# iQuantitator: A tool for protein expression inference using iTRAQ

**DOI:** 10.1186/1471-2105-10-342

**Published:** 2009-10-18

**Authors:** John H Schwacke, Elizabeth G Hill, Edward L Krug, Susana Comte-Walters, Kevin L Schey

**Affiliations:** 1 Department of Biochemistry, Medical University of South Carolina, Charleston, South Carolina, USA; 2 Department of Medicine, Medical University of South Carolina, Charleston, South Carolina, USA; 3 Department of Cell Biology and Anatomy, Medical University of South Carolina, Charleston, South Carolina, USA; 4 Department of Pharmacology, Medical University of South Carolina, Charleston, South Carolina, USA; 5 Mass Spectrometry Research Center, Vanderbilt University, Nashville, Tennessee, USA

## Abstract

**Background:**

Isobaric Tags for Relative and Absolute Quantitation (iTRAQ™) [Applied Biosystems] have seen increased application in differential protein expression analysis. To facilitate the growing need to analyze iTRAQ data, especially for cases involving multiple iTRAQ experiments, we have developed a modeling approach, statistical methods, and tools for estimating the relative changes in protein expression under various treatments and experimental conditions.

**Results:**

This modeling approach provides a unified analysis of data from multiple iTRAQ experiments and links the observed quantity (reporter ion peak area) to the experiment design and the calculated quantity of interest (treatment-dependent protein and peptide fold change) through an additive model under log transformation. Others have demonstrated, through a case study, this modeling approach and noted the computational challenges of parameter inference in the unbalanced data set typical of multiple iTRAQ experiments. Here we present the development of an inference approach, based on hierarchical regression with batching of regression coefficients and Markov Chain Monte Carlo (MCMC) methods that overcomes some of these challenges. In addition to our discussion of the underlying method, we also present our implementation of the software, simulation results, experimental results, and sample output from the resulting analysis report.

**Conclusion:**

iQuantitator's process-based modeling approach overcomes limitations in current methods and allows for application in a variety of experimental designs. Additionally, hypertext-linked documents produced by the tool aid in the interpretation and exploration of results.

## Background

Recent advances in instrumentation, reagents, and techniques for high throughput proteomics are making it possible to simultaneously identify and compare disease, development, and treatment-related changes to the level of protein expression [1]. In most cases, these techniques rely on two dimensional gel electrophoresis (2DGE) or liquid chromatography (LC) to separate proteins or peptides by charge, mass, or other chemical properties followed by identification using mass spectrometry (MS). For gel-based techniques, quantitation is performed by comparing the intensity of associated spots in gel images or by comparing the intensity of signals from appropriately excited Cyanine reactive dyes used to tag the samples being compared [2]. For techniques using liquid chromatography, methods for quantitating expression can be grouped into two broad classes: differential labeling, and label-free LC-MS [3]. Labeling methods such as SILAC, ICAT, and iTRAQ use isotopic or isobaric tags to differentially label the samples being compared and paired reporter ion or isotopic peaks provide estimates of the expression ratio for identified peptides. Label free methods estimate peptide abundance from a chromatographic elution profile, integrated across retention time or from the frequency with which a peptide is selected from an MS scan for MSMS analysis, termed spectral counting. In the former case, the integrated profile is taken to be indicative of the abundance of the associated peptide and thus a measure of associated protein abundance. In spectral counting, the methods compute protein abundance indices from the average of the associated peptide abundances based on these frequencies.

The isobaric Tag for Relative and Absolute Quantitation (iTRAQ™), has seen increased application in quantitative proteomics [4]. This technique uses four (or eight [5]) isobaric reagents to label and compare four (or eight) protein samples simultaneously. These reagents incorporate chemical tags consisting of a reporter group, a balance group, and a reactive group. The reactive group attaches the tag to free primary amino groups (e.g. N-terminal amines and lysines) where reporter groups, with masses of 114, 115, 116, and 117 Daltons, are linked to the reactive group by complementary balance groups with masses of 31, 30, 29, and 28 Daltons. The compensating masses of the reporter and balance groups yield a common mass of 145 Daltons. Therefore, a given peptide, modified by any of the individual iTRAQ reagents, appears at the same mass to charge in the MS spectrum. However, tandem MS spectra of these peptides yield, in addition to the identifying fragment ion peaks, reporter ion peaks at m/z 114, 115, 116, and 117 resulting from the singly charged reporter groups. The relative intensities of the reporter ion peaks are indicative of the relative quantities of the associated peptide across the four samples, from which relative expression of the cognate protein is inferred.

Each iTRAQ experiment produces tens of thousands of spectra (several gigabytes of data), several thousand identified peptides, and hundreds of identified proteins. Bioinformatic tools and statistical methods are essential to the interpretation of these data. Several data management and analysis tools have been developed to analyze these data, such as ProQuant, and ProteinPilot, software supplied by the manufacturer of the iTRAQ reagents, and a number of freely available tools. The software packages i-Tracker [6] and TandTRAQ [7] support the analysis of iTRAQ-generated quantitation data and the integration of that analysis with search results from Mascot, Sequest, and X!Tandem. The i-Tracker software performs reporter ion peak area calculations, isotopic impurity correction, threshold checking, and spectrum-level expression ratio calculations and links quantitations to peptide identities provided by Sequest or Mascot. TandTRAQ additionally provides a method for combining spectrum-level quantitation data from i-Tracker with peptide identifications from X!Tandem. Another iTRAQ data analysis tool, Multi-Q, provides instrument-independent processing, extracts reporter ion peak intensities, eliminates redundant peptides, and compensates for reporter ion saturation and variations in spectrum quality [8,9]. Multi-Q additionally provides both graphical- and web-based user interfaces. Quant, a MATLAB-based software package for iTRAQ data analysis, provides protein-level relative expression estimates and associated uncertainty measures using error propagation techniques [10]. Data management tools such as the Yale Protein Expression Database (YPED) facilitate biological interpretation through the capture, display, and linking of data from proteomic experiments using a variety of experimental techniques including iTRAQ [11]. Extensions to the Proteomics Identifications Database (PRIDE) and mzData standards have been proposed to permit the storage of iTRAQ reporter ion intensities [12,13]. Tools such as these for data manipulation, analysis, and storage are essential to the application of iTRAQ.

While early iTRAQ-based studies focused on comparisons to a common reference, a variety of experimental designs have now been suggested [14] and will likely grow in response to the introduction of higher throughput methods (e.g., 8-plex iTRAQ). In addition to increased complexity in experimental designs, pooling of information across experiments is needed to increase the power to detect small changes in expression and to improve estimates of the magnitude of those changes. Accommodating these advances will require statistical methods that support a variety of designs and permit inference across multiple iTRAQ experiments. Recently, we proposed a model for iTRAQ data analysis based on Analysis of Variance (ANOVA) [15]. The approach yields a linear model in log intensities from an iTRAQ experiment that can be analyzed using ANOVA methods. Oberg and colleagues apply this model in a study consisting of 6 iTRAQ experiments and discuss the computational challenges associated with fitting thousands of model parameters in the context of missing data typical of iTRAQ [14]. They emphasize the superiority of methods capable of simultaneously fitting all of the parameters to all of the data, describe the associated computational difficulties, and discuss strategies for dealing with these computational issues including subsetting, stagewise, and iterative regression. In the software described here, we attempt to address some of these difficulties using computational methods from the Bayesian statistical framework.

We report on the development and application of a new software tool, iQuantitator, designed to facilitate the analysis and reporting of results from iTRAQ experiments. This tool employs a model-based approach to describe sources of variation in the observed data and Bayesian inference to estimate the parameters of interest and their uncertainty, supports inference across multiple iTRAQ experiments addressing a common hypothesis, allows both protein and peptide-level treatment-effect analysis, and produces a hypertext-linked and searchable electronic report in the Portable Document Format (pdf).

## Implementation

### Reporter Ion Intensity Model

**Figure 1:**
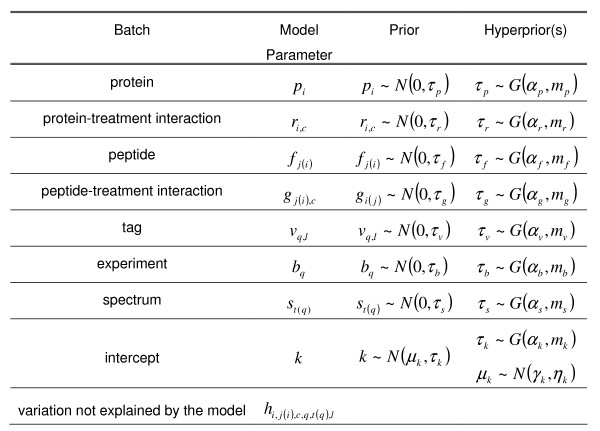
**Each source of variation included in the model is represented by a batch of parameters **(see [15]). The batches, prior distributions and associated hyperpriors are given here. Parameters are indexed as follows: protein: *i*, peptide: *j*(*i*), treatment: *c*, experiment: *q*, tag (or channel): *l*, spectrum: *t*(*q*). For the normal distribution *x *~ *N*(*μ*, *τ*), the form of the density is  and for the gamma distribution *τ *~ *G*(*α*, *m*), the form of the density is .

In the software described here, statistical inference employs a model that links experimental observations to the technical and experimental sources of variation and provides the basis for the inference of treatment-dependent changes in protein and peptide expression. We adapt our recently reported model for the analysis of iTRAQ data [15] in which the effects of the model are derived from the steps of the experimental process. The model is given by a multiplicative expression relating reporter ion peak area to biological and experimental factors associated with that peak. Log transformation of this multiplicative expression yields the following expression where the log reporter ion intensity is written as a linear combination of factors that decompose the sources of variation in the observed log reporter peak area (see Figure 1 for a definition of the terms).

(1)

This approach provides a framework for constructing models. For each study, the model must be tailored to capture the pertinent sources of variation and experimental effects of interest. In our previous efforts and those of Oberg, this model was analyzed using ANOVA, where the parameters are structured into batches that explain the sources of variation in the observed reporter ion peak area. All of the parameters associated with an effect of the model, for example protein effects (*p*_*i*_), are considered as a batch. While it is important to understand which sources of variation explain the observations, we are primarily interested in the inferred values of those effects that include an interaction with treatment, specifically, the protein by treatment interaction effect (*r*_*i*, *c*_). It is these parameter values that indicate treatment-dependent changes in protein expression. In Equation (1), we note that peptides are considered to be uniquely assigned to a protein as indicated by the notation *j*(*i*), indicating that peptide *j *is always associated with protein *i*. In cases where a peptide could be assigned to more than one protein, it is eliminated from consideration prior to analysis.

Examination of the model reveals the computational challenges discussed by Oberg and colleagues [14]. The model includes a number of parameters approximately equal to (*N*_*treatment *_-1) × (*N*_*protein *_+ *N*_*peptide*_) and these parameters must be estimated from the *N*_*spectra *_× *N*_*channel *_reporter ion intensities. In addition to the large number of parameters and the resulting computational costs, the inference method must contend with missing data. Within an experiment, many proteins are identified by a single peptide and the peptide and protein effects for that protein cannot, using traditional ANOVA techniques, be estimated independently. Additionally, the limited overlap in proteins identified in replicate iTRAQ experiments results in proteins that may not be observed in all experiments. In the experiments reported by Oberg [14], nearly 75% of the 992 identified proteins appeared in only one of six iTRAQ experiments and Liu, et. al [16] reported that 24% of 1751 identified proteins appeared in only one of nine iTRAQ experiments. To overcome these difficulties we employ a statistical model that allows information sharing across levels of a given effect and employ Markov Chain Monte Carlo methods to infer parameters from the data and parameter prior distributions.

### Model Parameter Inference

Parameter inference in iQuantitator employs computational methods developed using the Bayesian statistical framework. Applying Bayes theorem, the probability density of the model parameters conditioned on the observed data can be written as the product of the likelihood, the prior densities of the parameters, and integration constant. The joint posterior density of the parameters is obtained by integrating the likelihood over the prior densities. When a closed form solution of this integral is unavailable, computational methods such as Markov Chain Monte Carlo (MCMC) can be used [17]. The Markov chain constructed using these methods yields samples distributed according to the posterior distribution of the parameter vector. Statistical measures of center and spread estimated from these samples can be used to characterize the parameters of interest. Applying these methods to iTRAQ data requires the construction of a statistical model describing the prior distributions for each model parameter and, potentially, hyperprior distributions for distributional parameters of those priors. The statistical model structure used here follows the recommendation of Gelman [18] for the analysis of ANOVA using hierarchical regression. In this approach, all parameters of a given effect are considered as a batch and share a common prior distribution. Distributional assumptions of this model are given in Figure 1.

The Gibbs sampler implemented in iQuantitator was designed to exploit the structure of the model. Let *x*_*i *_be the *i*^th ^observed reporter ion peak area, assumed distributed





where *a*_*w *_are parameters of the model and *S*_*i *_are the set of parameter indices associated with peak area *i*. In our case, the set *S*_*i *_gives the indices of the parameters, one from each batch, that sum to give the mean of the distribution of *x*_*i *_and *a*_*w *_∈ {**p**|**r**|**f**|**g**|**v**|**b**|**s**|**h**}. The prior distributions for the *a*_*w *_are given by



where *B*(*w*) gives the batch to which parameter *w *is assigned (see Figure 1 for the prior specifications for each batch of parameters). Under these assumptions we can write closed form expressions for the full conditional distributions for parameters and hyperparameters of the model. For each parameter *a*_*w *_the full conditional distribution is given by

(2)

where *D*_*w *_is the set of indices of observed reporter ion peaks whose mean depends on *a*_*w *_and *n*() gives the number of members of the set. The hyperparameters for the prior distribution of batch *b *are updated using



where *H*_*b *_gives the set of indices of parameters assigned to batch *b*. Finally, the model precision parameter is updated using



Within a batch, the update of a parameter depends only on the observed reporter ion peak areas assigned to the associated factor level. Since each observation is assigned to only one parameter within a batch, during each iteration of the algorithm, all parameters assigned to a given batch are updated in parallel. For each batch, the software computes all of the sums in Equation (2) in one pass through the data, forming partial sums as needed. Once completed, all parameters in the batch are updated followed by updates to the batch hyperprior parameters *μ*_*b *_and *τ*_*b *_and this process is repeated for each batch. Finally, the model precision is updated. Throughout the batch updates, the means (*μ*_*i*_) are updated so that computational effort in the precision update is reduced.

### Software Description

**Figure 2:**
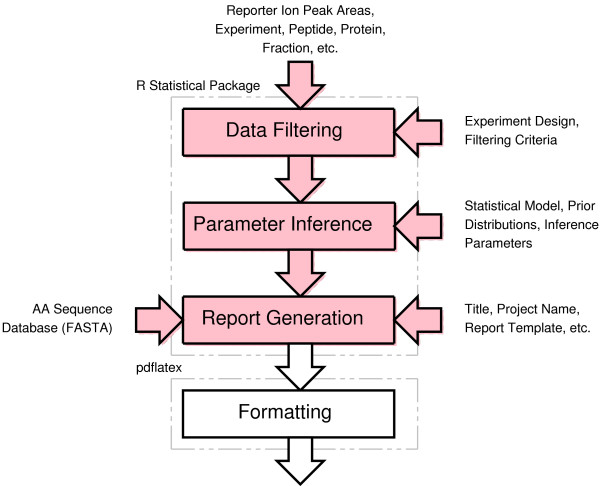
**The iQuantitor processing flow is divided into 3 main steps, data filtering, parameter inference, and report generation**. Each step corresponds to an R function as described in the text. The processing sequence can be customized to include additional analyses or diagnostics.

iQuantitator is available as an installable package for the freely-available R statistical computing environment and, through scripting, can be tailored to a variety of iTRAQ study designs. The package includes a collection of R functions for structuring input files, a Gibbs sampler designed for this application, and an R/latex script used to construct hypertext-linked reports. To make use of the package, users create an R script that specifies the input files, defines the experiment, gives the statistical model and the comparison of interest, and specifies the results file names. The software is intended for use by statisticians and analysts familiar with experimental design and statistical modeling. The processing flow of a typical iQuantitator application is illustrated in Figure 2

#### Data Import and Filtering

For each study, the user specifies the study design indicating the treatment group and sample identifier for each channel within each iTRAQ experiment (Figure 2). For each experiment, the user also provides a file giving the MS/MS reporter ion measurements, and peptide and protein identities for each MS/MS spectrum. iQuantitator relies on other external or vendor-supplied software to identify proteins from peptide-level tandem mass spectra (e.g. Mascot), measure iTRAQ reporter ion peak areas, and correct the peak areas for isotopic impurities in the iTRAQ reagents. The input file is typically a table containing one line for each observed MS/MS spectrum giving the name and accession number of the associated protein, spot or fraction identifier, best peptide sequence, list of modifications, and the reporter ion peak areas for each of the iTRAQ tags. These data are typical of that provided in the MS/MS summary reports generated by the Applied Biosystems software and is converted to a tab-delimited text file prior to processing. The default column names for each of these data items can be customized to match the tables produced by specific instruments and associated software.

Data import and filtering is implemented in the loadMSMSSummary function. In addition to the study design and a list of associated MS/MS summary files, the user may also specify contaminating proteins and protein modifications to be eliminated prior to analysis. The protein and modification filters are specified as lists of regular expressions [19], one expression for each protein or modification to be eliminated from the analysis. During loading, the protein name and modification fields in the MS/MS summary files are checked against the list of filtered proteins and modifications and are eliminated from the data set prior to analysis. Most often, this feature is used to eliminate experimental contaminants and peptides containing iTRAQ-modified tyrosines. In our applications, peptides without high confidence assignments (peptide assignment confidence below 70%) are also eliminated during the filtering step. The confidence threshold is specified using the confthreshold argument to loadMSMSSummary, and a count of spectra below threshold is provided in the summary report. Additionally, spectra assigned to more than one protein, unidentified spectra, and spectra with out-of-range data are also eliminated. The remaining data is restructured as a table with one row per reporter ion peak containing, in addition to the log transformed peak area, the descriptors of that peak (experiment, channel/tag, spectrum, protein, peptide, spot, treatment group, and sample). Additionally the software constructs a summary of the input data including: the number of spectra, unique proteins and unique peptides; the number of spectra eliminated during filtering or due to missing data; and the number of spectra observed for each reported peptide within each experiment.

#### Statistical Inference

To identify treatment-dependent, differentially expressed proteins, iQuantitator employs Bayesian inference using Gibbs Sampling as described above. This step is implemented within the processiTRAQ function. The user supplies a statistical model, specific to the experimental design, relating log-transformed reporter ion peak areas to the treatment effects of interest and other sources of variation. The user-supplied model, expressed in a restricted version of the R formula grammar, is specified so as to capture both the comparisons of interest and the sources of variation that interfere with those comparisons. The user may also specify parameters controlling the Gibbs sampler and prior distributions for the model parameters. The Gibbs sampler, implemented as a C library function, is used to draw samples of the model parameter vector from its joint posterior distribution. The sampling process produces a large table, with one row per monitored parameter (approximately twice the sum of the number of unique peptides and unique proteins) and number of columns equal to the number of samples retained (thousands of columns). The user-specified thinning and burn-in parameters control the number of MCMC iterations between stored samples and the number of samples discarded at the beginning of the sampling process, and can be used to reduce this table to a reasonable size. Monitoring flags for each factor allow data collection to be selectively bypassed for nuisance parameters. To assess convergence, MCMC variable trace plots can be generated and MCMC diagnostics can be applied to the sampled parameters using existing R packages [20] and functions.

#### Statistical Summary Preparation

For each parameter of interest, iQuantitator computes summary statistics giving a point estimate and credible interval from which the fold change estimate and its uncertainty can be determined. This step is performed in the summarizeiTRAQ function. Although the software draws samples from the joint posterior distribution for all of the parameters in the model, we focus our statistical summary only on those parameters associated with the treatment effects. In particular, we focus on the protein-treatment interaction factor, and the peptide-treatment interaction factor, typically specified in the model. For each parameter within these effects, the software estimates, using samples collected from the MCMC algorithm, the mean, median, standard deviation, and 2.5^th ^and 97.5^th ^percentiles. These values are exponentiated to give both point estimates (mean and median) and credible intervals (2.5^th ^and 97.5^th ^percentiles) for the protein and peptide fold change due to treatment. The protein and associated peptide summary statistics are organized into a hierarchical data structure. Additionally, the software builds a map of identified peptides along the associated protein sequence. The protein accession number is used to locate the protein sequence in a user-supplied protein database file (in FASTA format). The sequence is then included in the resulting data structure.

#### Report Preparation

**Figure 3:**
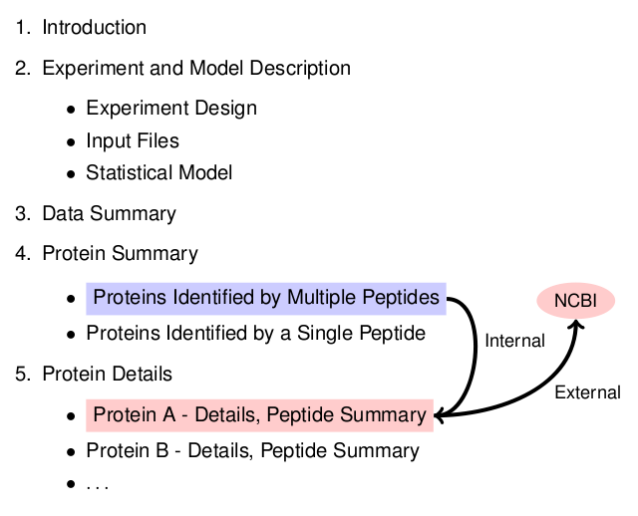
**The default report structure provides increasingly detailed summaries of the results with in-document hypertext links between single line protein summaries in Section 4 to subsections of Section 5 containing additional details for each protein and a summary of the supporting evidence from each peptide**. Within these details, the document provides external links to the Proteins database at NCBI.

**Figure 4:**
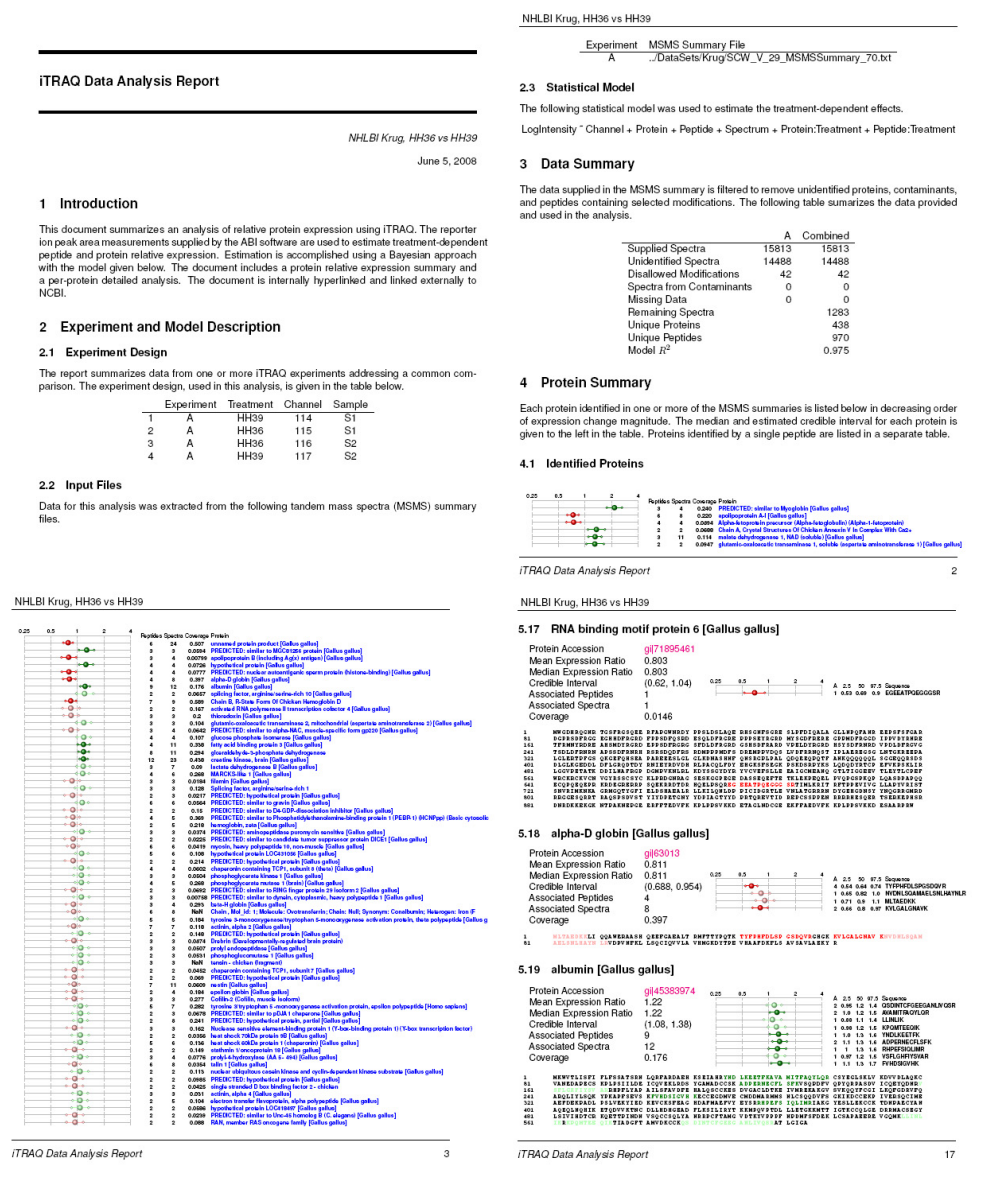
**Sample pages of the analysis report for the biological sample discussed here show the form and content of the report**. The experiment design, statistical model, and a summary of the raw data are given on the first two pages followed by a summary (one line per protein) of data for proteins identified by more than one peptide (pages 2 and 3 of this example). Each protein summary provides a hypertext link to detailed information (page 17 in this example) found later in the document. Clicking on the protein accession number links to NCBI.

The final step in the iQuantitator processing sequence is the construction of a latex document (.tex file) containing formatted versions of the experiment description, statistical model, data summary, and protein and peptide treatment effect summary data structures. This step utilizes the Sweave automatic report generator, a component of the R utils package. Sweave processes a user-supplied script containing both latex and R code and merges the output of the R code with the latex to create a .tex file suitable for document production. The resulting .tex file is then converted into a Portable Document Format (.pdf) file using the pdflatex processor. The iQuantitator package includes a default Sweave script that can be used as is or modified to change the form, content, or organization of the resulting report. The document is structured to provide both graphical and numerical summaries of the results with hypertext links providing quick access to document sections containing detailed analyses (see Figure 3). The protein summary section provides a graphical and numeric summary of all identified proteins with results sorted by decreasing magnitude of expression fold change. Protein names are hypertext linked to subsections within the Protein Details section where additional statistics, protein sequences showing peptide coverage, and peptide-level expression change summaries can be found. The protein accession number, given in each subsection of Protein Details, also provides a hypertext link to the NCBI Protein database (Figure 4). Clicking on this link will launch the user's web browser and direct it to the appropriate NCBI Protein web page on most platforms. Additionally, the summary report document can be navigated using bookmarks and searched using the search tools found in most Portable Document Format readers such as Adobe's Acrobat Reader [21].

Software was developed using version 2.6.2 of the R Statistical Computing Environment [22].

### Simulated Data Sets

A set of simulated data sets was produced to further investigate the performance of iQuantitator. Two sets of four tests simulating studies with single iTRAQ runs and one set of two iTRAQ experiments from a single study were generated using a simulator developed in our laboratory for testing inference methods. Biological, treatment, and technical variation can be set as desired to construct simulated protein expression profiles for individual samples. The simulator models the peptide composition of proteins, inserts post-translational modifications and treatment-dependent modifications, models loading errors, labeling efficiency, fractionation of peptides, MS intensities, peak selection logic, and reporter ion measurement noise. The resulting output is converted, via an R script, into files satisfying the input requirements of iQuantitator. For this study, the simulator was configured to draw from a pool of 5000 proteins composed of peptides from a pool of 850,000. The peptides were randomly assigned modifications with a probability of 0.05. Both loading error and tagging efficiency were set to model ideal conditions and the peptides were distributed across 14 first dimension fractions and 400 second dimension fractions. Peak selection was limited to peptides in the m/z range 800 to 3500 and only the 10 most intense peaks in the simulated mass spectrum were processed in simulated MSMS. A peak was not reinterogated if already appearing in any of the 30 previous fractions. The ion suppression model was set as to minimize the effects of suppression and treatment, biological and technical noise were added to the output. Each simulation run modeled samples from four subjects, two each from two treatment groups. Channels 1 and 3 were assigned to subjects from treatment group 1 and channels 2 and 4 to subjects from treatment group 2. Each experiment dataset contained approximately 1050 unique proteins, 4800 unique peptides, and 7000 spectra (see Table 1). Four additional single-run experiments of similar design were simulated, this time including treatment-dependent modifications. When treatment-dependent modifications are included and the modified form of the protein is not observed, datasets include peptides whose expression ratio differs significantly from other peptides of the same protein. Finally, one additional simulation dataset was constructed to examine the analysis of data from multi-iTRAQ studies. In this case, a common pooled control is included in the first channel of two experiments and simulated samples from six individuals of the treatment group are assigned to the three channels remaining in each of the two experiments. The simulated data set included 1321 proteins, 6762 peptides, and 13,524 spectra. Of the 1321 proteins, 47% were identified by a single peptide, and 40% were observed in only one of the simulated iTRAQ experiments. As in the first set of single experiment cases, no treatment-dependent modifications were included.

**Table 1:** Summary of simulated data sets produced as described in the text and results of analysis using both iQuantitator and log-space averaging.

						**iQuantitator**			**Mean of 116/117 and 114/115**		
						
**Simulated Data Set**	**Proteins**	**Peptides**	**Spectra**	**Peptides per Protein**	**Single Hit Proteins**	**Mean Error**	**Mean SE**	**Median SE**	**Mean Error**	**Mean SE**	**Median SE**
Case 1	1034	4776	6938	4.6	494	-0.004	0.009	0.004	0.043	0.012	0.006
Case 2	1049	4452	6359	4.2	530	0.002	0.009	0.004	-0.020	0.010	0.005
Case 3	1061	4958	7049	4.7	514	0.006	0.010	0.004	0.004	0.011	0.005
Case 4	1032	4791	6923	4.6	492	0.005	0.008	0.003	-0.004	0.009	0.004
Average							**0.009**	**0.004**		**0.011**	**0.005**
											
With Mods 1	1055	4784	6911	4.5	515	-0.002	0.037	0.011	-0.013	0.140	0.011
With Mods 2	1026	4744	6776	4.6	507	0.003	0.042	0.010	-0.026	0.194	0.011
With Mods 3	1052	4769	6830	4.5	511	0.003	0.038	0.010	-0.008	0.114	0.011
With Mods 4	1066	4770	6869	4.5	516	-0.001	0.042	0.010	-0.015	0.199	0.011
Average	1050	4756	6832	5	510		**0.040**	**0.010**		**0.162**	**0.011**
Multi-Experiment	1321	6762	13534	5.1	620	-0.009	**0.005**	**0.005**	-0.004	**0.005**	**0.005**

Simulated data from the two sets of four single-experiment studies and the multi-experiment study were processed with both iQuantitator and an R script that mimics the log-space averaging approach used in the ABI software. The log space averaging script computes raw ratios to a selected reference and normalizes the ratios to a common median. The normalized and log-transformed ratios for a given protein are averaged and then exponentiated to obtain the protein-level fold change estimates. Since each of the single experiment cases included two samples from both control and treatment groups, our log-space averaging function also computes averages of paired ratios to give a single estimate of the fold change. Point estimates for the protein expression fold change were taken from each approach and compared to the true expression ratio (recorded by the simulation).

### Biological Sample Preparation and Processing

In addition to the simulated cases, we applied iQuantitator to a biological sample collected as part of an on-going study of embryonic heart development in chickens. Fertile chicken eggs (Hubbard ISA Hatchery, Statesville, NC) were incubated at 37°C in a humidified atmosphere with hourly rotation for 10 to 14 days and staged according to the criteria of Hamburger and Hamilton (HH) (1951). Hearts from HH stage 36 (n = 72) and HH stage 39 (n = 24) were perfused *in situ *with warm phosphate buffered saline, excised from the embryo and cut in half, then rinse blotted dry, flash frozen in liquid nitrogen. Pooled hearts from the two stages were cryo-pulverized (Bio-Pulverizer, RPI Corp.), then homogenized in 5 mM bicine, pH 9.3 using a ground glass mortar and pestle (1:10; w:v), and centrifuged at 700 × g for 10 min at 4°C. The cytosolic fraction was obtained by centrifugation of the low-speed supernatant at 150,000 × g for 90 min at 4°C (Beckman Coulter TLX ultracentrifuge, TLS-55 rotor, 50 k rpm). Experiments were conducted in compliance with guidelines established by the Institutional Animal Care and Use Committee (IACUC) of the Medical University of South Carolina for the utilization of embryonated eggs prior to 18 days of age.

One hundred micrograms of total protein (BCA reagent; Pierce Chemical) from each aliquot were labeled with each of the 4-Plex iTRAQ reagents (Applied Biosystems, Foster City, CA) according to the manufacturer's standard protocol. Briefly, the aliquots were denatured with a sodium dodecyl sulfate solution, reduced with tris-2-carboxyethyl phosphine (TCEP), and alkylated by adding s-methyl methanethiosulfonate (MMTS). The aliquots were digested with 10 μg of trypsin (Applied Biosystems) each. Ten μL of 1 M tetraethyl ammonium bicarbonate buffer (pH 8.5) were added to ensure proper pH during labeling. After labeling, the four aliquots were combined and fractionated by strong cationic exchange (SCX) chromatography on a Waters 600-MS HPLC system connected to a Waters 484-MS UV detector. A PolySULFOETHYL A™ column (200 × 2.1 mm I. D., 5 μm, 200 Å) (PolyLC Inc., Columbia, MD) was used. Solvent A was 10 mM KH2PO4, 25% acetonitrile (ACN), pH 2.9; solvent B was similar to A but with the addition of 1 M KCl. A 45 minute gradient from 5% to 50% B, followed by 20 minutes at 50% B provided acceptable separation of the peptides. The flow rate used was 250 μL/minute, and the elution of peptides was monitored by UV at 220 nm. Fractions were collected every 5 minutes. Fractions were dried and stored at -20°C until further use. SCX fractions containing peptides were further fractionated by C18 nano-reversed phase chromatography on an Ultimate-Switchos- Probot system (LC Packings, Sunnyvale, CA). The peptides were first loaded using the Switchos system on a C18 PepMap 100 pre-column (5 mm × 300 μm I. D) (LC Packings) using 2% ACN, 0.1% trifluoroacetic acid (TFA) at 40 μL/minute. After 20 minutes of desalting, the peptides were eluted from the pre-column onto a C18 PepMap 100 column (150 mm × 75 μm I. D.) (LC Packings) using the Ultimate system at 200 nL/minute. Solvent A was 2% ACN, 0.1% TFA; solvent B was 85% ACN, 5% 2-propanol, and 0.1% TFA. An 80 minute gradient from 5% B to 50% B, followed by 30 minutes at 50% B was used. Peptide elution was monitored at 214 nm. The eluant from the reversed-phase HPLC separation was mixed at a 1:2 (eluant: matrix) ratio with a solution of α-cyano-4-hydroxy-cinnamic acid (Bruker Daltonics, Billerica, MA) being continuously delivered from the syringe pump of the Probot system. The mixture was spotted on stainless steel MALDI plates (Applied Biosystems) in a 24 × 24 pattern. Spots were collected every 10 seconds during peptide elution of the reversed phase chromatography run. Typically, two plates were collected for each SCX fraction. Six mass calibration spots were manually spotted on the perimeter of the plate, and two mass accuracy verification spots were manually placed on the top center and bottom center of each plate. Matrix-assisted laser desorption ionization mass spectrometry (MALDI-MS) analyses was performed on a 4700 Proteomics Analyzer tandem time-of flight (TOF) mass spectrometer (Applied Biosystems). One MS spectrum was acquired for each spot in positive reflector mode; subsequently the 15 most intense precursors from each spot were selected for tandem MS/MS sequencing. For peptides eluting in more than one spot, the interpretation method was set up to minimize repetition of the MS/MS data collection on the same precursor. MS/MS spectra were collected starting with the least abundant peptide in each fraction to maximize data quality. The raw MS/MS data were filtered using a signal-to-noise ratio of 15 and searched using GPS Explorer software (version 3.6) and Mascot (version 2.1). iTRAQ labeled N-terminal and Lysine, and MMTS labeled cysteine were selected as fixed modifications, while oxidation of methionine and iTRAQ labeled tyrosine were used as variable modifications. A 70% confidence cut-off was used at the peptide level. The Gallus gallus protein database used for the search was extracted from the NCBI non-redundant database downloaded from the NCBI website on May 4, 2005. The MS/MS summary report containing peptide sequence, Mascot scores, protein assignment to each peptide, iTRAQ reporter areas (corrected for isotopic impurity based on the certified purity supplied by the manufacturer with each batch of reagents), and other information used in this study were generated using the GPS Explorer software.

Tandem MS summary reports comparing stage 36 and stage 39 chicken hearts and containing 15,813 MSMS spectra were processed using iQuantitator. Of those spectra, 14,488 lacked a high confidence identification, and 42 were eliminated due to disallowed modifications (iTRAQ-modified tyrosines) leaving 1,283 spectra with high confidence identifications from 438 unique proteins and 970 peptides. Using iQuantitator, 200,000 samples were collected from the Markov chain following a burn-in period of 40,000 updates. The samples were thinned by a factor of 20 leaving 5,000 samples from which summary statistics were collected. For each protein and each associated peptide, mean, median, and 95% credible intervals were computed for each of the protein- and peptide-level treatment effects.

## Results

### Simulated Data

**Figure 5:**
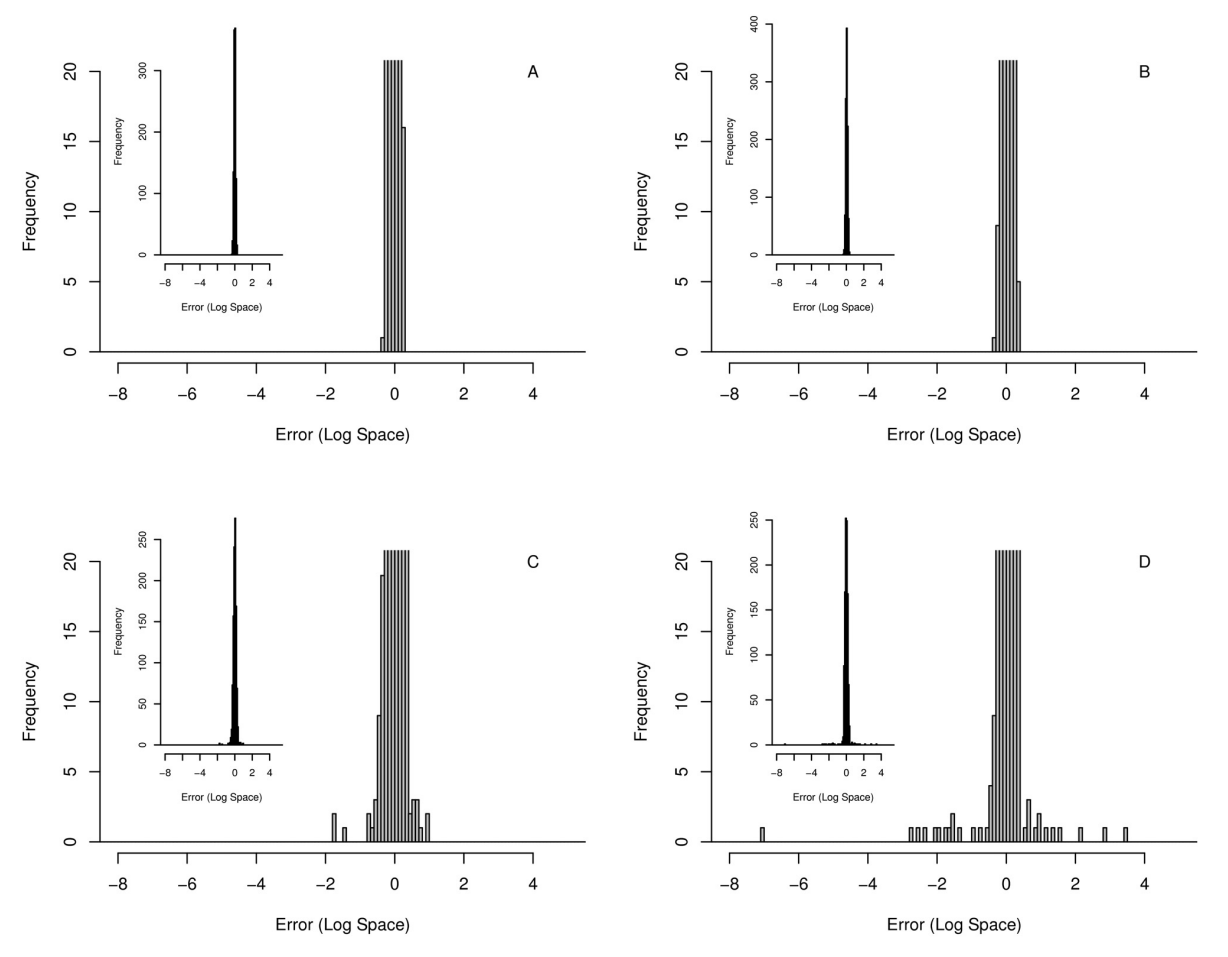
**Histograms showing errors in the log fold change estimate for a representative case without modifications (panels A and B) and a representative case with modifications (panels C and D) for iQuantitator (panels A and C) and the log-space averaging approach (panels B and D) provide a comparison of these methods**. Inset graphs show the complete histogram for each case and main plots limit the y axis to 20 to make the proteins with larger errors more visible. Without modifications, both methods give similar results, with modifications the mean square error for iQuantitator is noticeably lower due to a relatively few proteins with large fold change errors in the log space averaging approach.

We find that the model-based approach implemented here results in a smaller mean square error in the estimated log fold changes (see Table 1) when compared to individual paired estimates using the log averaging approach or when those paired estimates are averaged. This is especially evident in the second set of cases (simulated modifications) where the log-space mean square error was found to be 0.04 using iQuantitator and 0.19 using the log averaging approach. In these cases, a number of peptides exhibit fold changes that differ greatly from the associated protein when only one form of that peptide is observed (modified or unmodified) and the extent of modification varies across samples or treatment groups. The histograms of Figure 5 give error distributions for the two approaches and illustrate the presence of the few cases that greatly increase the mean error in the log-space averaging approach. When the median square error is used instead of the mean square error, the few cases resulting in large errors are less important and the difference between iQuantitator and the log-averaging approach is less noticable (0.01 vs 0.02). Many proteins identified in iTRAQ experiments are associated with only a few peptides. Using the log-space averaging approach, the fold change estimate for a protein makes no use of the data associated with other proteins/peptides and the overall distribution of fold changes seen in the data. The inclusion of a common prior distribution allows information to be shared across proteins and combines data across samples. In these examples, two samples from each treatment group were included in the experiment. Here, we used the model to combine information from samples from each treatment group to create a single estimate of the expression change across groups. In the simulated multi-experiment case, the results from iQuantitator and the log-space averaging approach were comparable. As in the first group of single-experiment cases, no treatment dependent modifications were included and, as before, the mean square errors for the two methods were similar (Table 1).

While the proposed method demonstrates a lower prediction error, we must also note that the method tends to produce estimates shifted towards the null hypothesis (no change in expression). Resulting estimates are, thus, conservative. In the model used in this study, the prior distribution is given a 0 mean with an unknown precision, thus indicating a prior preference for the null hypothesis. The prior specification can be adjusted to relax this preference as desired. We also note that in cases where the data is noisy and the resulting estimates in precision are small, the shift tends to increase. This effect is evident in Equation (2) which gives the rule for updating the model effects. The update mean is a sum of the prior mean and the mean estimated from the data, weighted by the prior precision and the model precision. With noisy data, the prior precision can tend to dominate, driving the estimate toward the prior mean (0 in this case) and thus underestimating the magnitude of the expression change. We find that with proper selection of prior parameters, the disadvantage of the null shift tends to be balanced by the improved accuracy in the expression change estimate. Adjustment of the prior specification can be used to control this balance.

### Chicken Heart Development

Embryonic development is an excellent model system for evaluating a continuum of protein expression dynamics as no extrinsic manipulations are required to affect change - it is a highly integrated melody of natural processes, which minimizes experimenter-related error. While early embryonic events proceed rapidly, the later phases of development are largely characterized by overt growth of organs and tissues that approximate their more mature counter parts Martinsen (2005). We chose to compare the chick heart cytosol proteome of HH stage 36 vs 39 embryos as this time difference coincides primarily with a 3-4 fold increase in muscle mass. There are also more subtle, but critical changes in the conduction system and valvular structure, with the potential for detecting tissue specific isoform expression that would allow for assessing the detection limits of the iTRAQ methodology. However, the results of the HH stage 36 vs. 39 comparison showed little difference in the heart cytosolic protein composition aside from constituents in the blood, possibly reflecting expansion of the coronary network.

**Figure 6:**
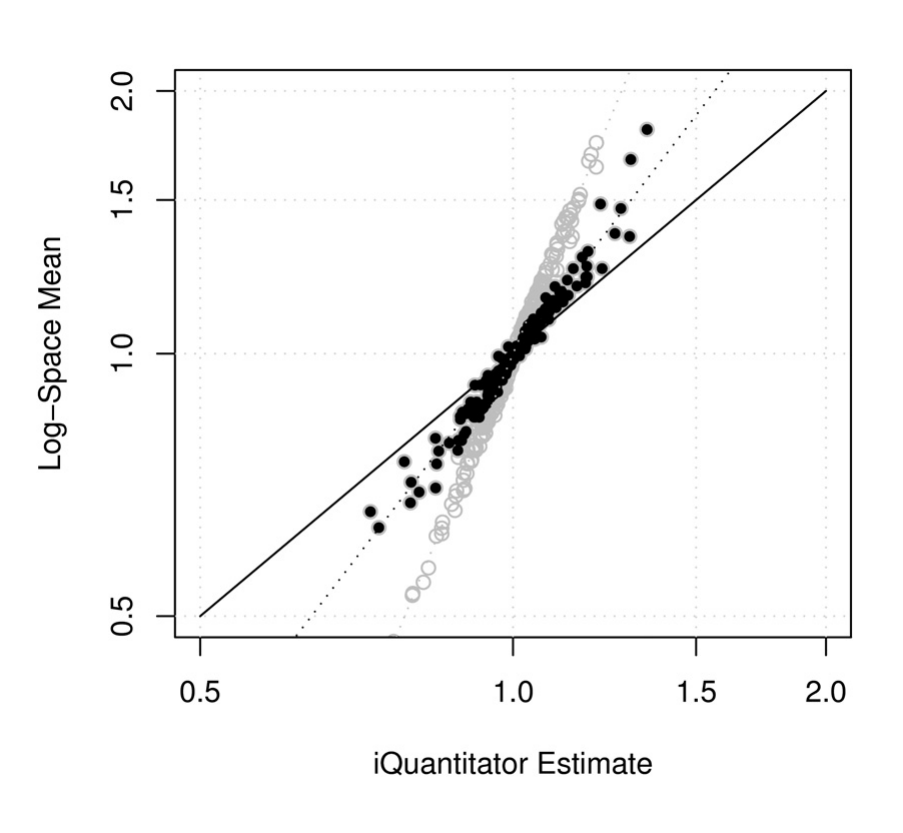
**Comparison of expression change estimates using iQuantitator and log-space averages for data from stage HH36 versus HH39 embryonic chicken hearts**. Solid circles give results for proteins identified by more than one peptide, open circles give results for proteins identified by a single peptide. Dotted lines give a linear fit in log coordinates and illustrate tendency toward conservative estimates of fold change.

To facilitate further understanding of the performance of the software described here, we also compared estimates derived using log space averaging to the estimates provided by iQuantitator. In this experiment, two samples from each of two treatment groups were processed, ratios for each of the two control treatment pairs were computed for each reported spectrum with identification meeting the search criteria (see Methods). Each set of ratios was normalized to the median of the set. For each protein the log-space average of the normalized ratios for all spectra assigned to a given protein is computed providing a single log-ratio average for each identified protein. Figure 6 compares the estimates provided by the two methods. From the figure we see the tendency toward conservative estimates when using iQuantitator discussed above. While proteins identified by a single peptide would not normally be reported, we plot them here (open circles) to illustrate how the shift toward more conservative estimates is affected by the available information.

## Discussion

We report here on the development and application of a new software tool, iQuantitator, designed to facilitate analysis and reporting of iTRAQ data. The tool's model-based approach, inference using the Bayesian framework, customized Gibbs Sampler, and R-based hypertext-linked report generator aid in the analysis of iTRAQ data for a variety of experimental designs.

Experiments involving multiple iTRAQ runs present a number of challenges including limited coverage overlap across runs, proteins identified by single peptides, and proteins appearing in a single experiment. The statistical and computational approach employed here attempts to address many of these problems without requiring special handling. As noted above, the approach used here allows for information sharing across levels of a given model effect (termed a "batch"). For example, the treatment-dependent protein relative expression estimate is based on both the information specific to that protein and the variation in relative expression over all proteins. This sharing of information is evident in Equation (2), the update rule for model parameters. Within the model, the term *g*_*j*(*i*), *c *_represents the log difference between protein *i*'s treatment-dependent change in expression and that of associated peptide *j(i)*. When a single peptide identifies a protein, the relative expression estimate is shared between *g*_*j*(*i*), *c *_and *r*_*i*, *c*_. When peptides within the sample provide consistent estimates of the associated protein, the protein-level estimate for proteins identified by a single peptide tend to follow the peptide estimate and the posterior estimate of *g*_*j*(*i*), *c *_tends toward 0. In cases where the peptide-level estimates of relative expression differ significantly from the associated protein-level estimates, as in our simulated cases involving protein modifications, the tendancy is for a more pronounced shift toward conservative estimates of relative expression. Difference in estimates for proteins identified by a single peptide and those identified by more than one peptide can be seen in Figure 6 where we see an increased tendency for estimates to shift toward the null hypothesis (more conservative estimate). In cases of increased noise or limited data, the software tends to produce more conservative estimates.

High-throughput proteomics is a complicated process involving chemical modification, instrumentation, and information processing. An objective of this effort has been the development of an approach that links the biological parameters of interest (protein expression levels) to the observed quantities (reporter ion peak areas) through a model that represents the sources of variation in the experimental process. The development of a model-based inference approach eases the adaptation of the software to changing experimental designs and processes. iQuantitator employs statistically-motivated inference for parameters of a model derived from the experimental process. As such, the sources of variation accounted for in the model are evident and the exponentiated value of each parameter has a clear meaning. The model includes both protein and peptide-level treatment effect estimates allowing for the identification of proteins whose expression changes in a treatment-dependent manner as well as for peptides differentially expressed (modifications, splice variants) relative to the associated proteins. The model can be adapted to a variety of experimental designs, can accommodate more than one treatment group, is not restricted to a single reference channel, and can merge information across experiments.

While a variety of web-based tools have been developed, we choose to consider presentation of results using an electronic document (e-Document) based on the Portable Document Format. Hypertext linking both within the document and to web resources provides many of the features of a web-based interface in a single file that can be easily distributed and viewed with a variety of document readers. The document structure chosen here allows the user to drill down from protein-level data with graphically depicted expression changes and associated uncertainty to the supporting peptide data with summaries across multiple iTRAQ experiments. The entire document is bookmarked and searchable using the capabilities of the user's PDF reader. We believe that many additional features could be included in the e-document report through the use of embedded scripting (Javascript) and forms. Additionally, the design of the package allows other reporting interfaces to be incorporated. The Sweave package used here can also generate HTML using a similar approach and existing interfaces between R and common database engines allow the possibility of pushing results directly to a data management system.

## Conclusion

Rapid advances in high-throughput proteomic technologies are requiring comparable advances in modeling, statistical methods, and visualization. iQuantitator's process-based modeling approach overcomes limitations in current methods and allows for application in a variety of experimental designs and meaningful integration of data across experiments. Additionally, inference in the Bayesian framework and an efficient Gibbs Sampler overcomes estimation problems noted by other researchers. iQuantitator is available from the authors as an installable R package.

## Availability and Requirements

Project name: iQuantitator

Operating system(s): Platform independent

Programming language: R, C

Other requirements: The R software for statistical computing (version 2.6.2 or later), pdflatex (for hypertext-linked report generation)

License: GNU GPL

Any restrictions to use by non-academics: None

An installable R package including all source code [see Additional File 1], sample output [see Additional File 2], a document describing the installation and application of the software [see Additional File 3], an input file containing the MSMS summary reports from our experiment [see Additional File 4], a sample R script demonstrating the analysis of experimental data described here [see Additional File 5], and a test-case specific protein sequence file in FASTA format [see Additional File 6] are provided.

## Competing interests

The authors declare that they have no competing interests.

## Authors' contributions

JHS designed and implemented iQuantitator. ELK proposed requirements for the reports, provided biological samples, and interpreted the results. EGH designed the statistical model. KLS supervised all MS sample processing and recommended features for the report. SCW collected MS data. JHS, ELK, KLS, and SCW contributed to writing the manuscript and all authors read and approved the manuscript.

## Supplementary Material

Additional File 1 **Installable R package file for iQuantitator**. This archive contains a single file that can be installed under the R statistical computing environment. Installation procedures and usage are described in Additional File 6. Click here for file

Additional File 2 **iQuantitator-generated output from the analysis of the chicken heart data**. This document provides an example of the iQuantitator output for the data collected from the comparison of stage 36 and stage 39 embryonic chicken hearts. Click here for file

Additional File 3 **iQuantitator Installation and Usage Manual**. A step-by-step introduction to the installation and use of iQuantitator based upon the sample data and scripts provided here. Click here for file

Additional File 4 **MSMS summary data from the embryonic chicken heart comparison**. A text file, exported from the MSMS summary report generated using the Applied Biosystems software that is the primary input for iQuantitator. Click here for file

Additional File 5 **R Script used to create **Additional File 1. This archive contains an R script that was used to generate the sample output (Additional File 1) using the data found in Additional File 4. Use of the script requires installation of the iQuantitator package. Click here for file

Additional File 6 **Example-specific FASTA file**. An archive containing a FASTA-formatted protein sequence file with a subset of the protein sequence database used by iQuantitator, sufficient to support the example provided here. Click here for file
