# Supplementary material for: iQuantitator: A tool for protein expression inference using iTRAQ
Source: BMC Bioinformatics. 2009 Oct 18;10:342. doi: 10.1186/1471-2105-10-342 (PMC2770557; doi:10.1186/1471-2105-10-342)
Supplement: Additional File 2 — iQuantitator-generated output from the analysis of the chicken heart data. This document provides an example of the iQuantitator output for the data collected from the comparison of stage 36 and stage 39 embryonic chicken hearts. [file 1471-2105-10-342-S2.PDF]

iTRAQ Data Analysis Report

NHLBI Krug, HH36 vs HH39

January 30, 2009

1 Introduction

This document summarizes an analysis of relative protein expression using iTRAQ. The reporter ion peak area measurements supplied by the ABI software are used to estimate treatment-dependent peptide and protein relative expression. Estimation is accomplished using a Bayesian approach with the model given below. The document includes a protein relative expression summary and a per-protein detailed analysis. The document is internally hyperlinked and linked externally to NCBI.

2 Experiment and Model Description

2.1 Experiment Design

The report summarizes data from one or more iTRAQ experiments addressing a common comparison. The experiment design, used in this analysis, is given in the table below.

	Experiment	Treatment	Channel	Sample
1	A	HH39	114	S1
2	A	HH36	115	S1
3	A	HH36	116	S2
4	A	HH39	117	S2

2.2 Input Files

Data for this analysis was extracted from the following tandem mass spectra (MSMS) summary files.

Experiment	MSMS Summary File
A	SCW_V_29_MSMSSummary_70.txt

2.3 Statistical Model

The following statistical model was used to estimate the treatment-dependent effects.

LogIntensity ˜ Channel + Spectrum + Protein:Treatment + Peptide:Treatment

3 Data Summary

The data supplied in the MSMS summary is filtered to remove unidentified proteins, contaminants, and peptides containing selected modifications. The following table sumarizes the data provided and used in the analysis.

	A	Combined
Supplied Spectra	15813	15813
Unidentified Spectra	14488	14488
Disallowed Modifications	42	42
Spectra from Contaminants	0	0
Missing Data	0	0
Low Confidence Spectra	0	0
Degenerate Peptides	0	0
Remaining Spectra		1283
Unique Proteins		438
Unique Peptides		970
Model R^2		0.975

4 Protein Summary

Each protein identified in one or more of the MSMS summaries is listed below in decreasing order of expression change magnitude. The median and estimated credible interval for each protein is given to the left in the table. Proteins identified by a single peptide are listed in a separate table.

4.1 Identified Proteins

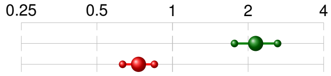

Peptides	Spectra	Coverage	Protein
**3**	**4**	**0.240**	**PREDICTED: similar to Myoglobin [Gallus gallus]**
**6**	**8**	**0.220**	**apolipoprotein A-I [Gallus gallus]**

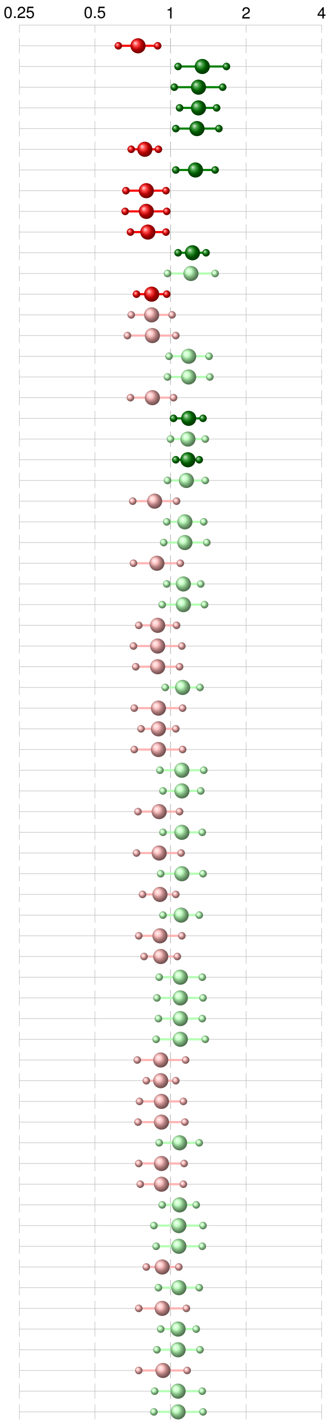

Peptides	Spectra	Coverage	Protein
4	4	0.0894	Alpha-fetoprotein precursor (Alpha-fetoglobulin) (Alpha-1-fetoprotein)
2	2	0.0688	Chain A, Crystal Structures Of Chicken Annexin V In Complex With Ca2+
2	2	0.0947	glutamic-oxaloacetic transaminase 1, soluble (aspartate aminotransferase 1) [Gallus gallus]
3	11	0.114	malate dehydrogenase 1, NAD (soluble) [Gallus gallus]
3	3	0.0594	PREDICTED: similar to MGC81256 protein [Gallus gallus]
6	24	0.507	unnamed protein product [Gallus gallus]
4	4	0.0726	hypothetical protein [Gallus gallus]
4	4	0.0777	PREDICTED: nuclear autoantigenic sperm protein (histone-binding) [Gallus gallus]
3	4	0.00799	apolipoprotein B (including Ag(x) antigen) [Gallus gallus]
4	8	0.397	alpha-D globin [Gallus gallus]
9	12	0.176	albumin [Gallus gallus]
2	2	0.0657	splicing factor, arginine/serine-rich 10 [Gallus gallus]
7	9	0.589	Chain B, R-State Form Of Chicken Hemoglobin D
3	4	0.0642	PREDICTED: similar to alpha-NAC, muscle-specific form gp220 [Gallus gallus]
2	2	0.167	activated RNA polymerase II transcription cofactor 4 [Gallus gallus]
4	4	0.107	glucose phosphate isomerase [Gallus gallus]
3	3	0.104	glutamic-oxaloacetic transaminase 2, mitochondrial (aspartate aminotransferase 2) [Gallus gallus]
3	3	0.2	thioredoxin [Gallus gallus]
8	11	0.294	glceraldehyde-3-phosphate dehydrogenase
4	11	0.338	fatty acid binding protein 3 [Gallus gallus]
12	23	0.438	creatine kinase, brain [Gallus gallus]
3	7	0.090	lactate dehydrogenase B [Gallus gallus]
3	3	0.0184	filamin [Gallus gallus]
4	6	0.268	MARCKS-like 1 [Gallus gallus]
3	3	0.128	Splicing factor, arginine/serine-rich 1
2	3	0.0217	PREDICTED: hypothetical protein [Gallus gallus]
6	6	0.0564	PREDICTED: similar to gravin [Gallus gallus]
3	3	0.0374	PREDICTED: aminopeptidase puromycin sensitive [Gallus gallus]
4	5	0.369	PREDICTED: similar to Phosphatidylethanolamine-binding protein 1 (PEBP-1) (HCNPpp) (Basic cytosolic
2	2	0.15	PREDICTED: similar to D4-GDP-dissociation inhibitor [Gallus gallus]
2	5	0.218	hemoglobin, zeta [Gallus gallus]
5	6	0.108	hypothetical protein LOC431056 [Gallus gallus]
2	2	0.0225	PREDICTED: similar to candidate tumor suppressor protein DICE1 [Gallus gallus]
6	6	0.0419	myosin, heavy polypeptide 10, non-muscle [Gallus gallus]
2	2	0.214	PREDICTED: hypothetical protein [Gallus gallus]
3	3	0.00758	PREDICTED: similar to dynein, cytoplasmic, heavy polypeptide 1 [Gallus gallus]
4	5	0.268	phosphoglycerate mutase 1 (brain) [Gallus gallus]
3	4	0.293	beta-H globin [Gallus gallus]
4	4	0.0602	chaperonin containing TCP1, subunit 8 (theta) [Gallus gallus]
2	3	0.0692	PREDICTED: similar to RING finger protein 29 isoform 2 [Gallus gallus]
3	3	0.0504	phosphoglycerate kinase 1 [Gallus gallus]
6	8	NaN	Chain , Mol_id: 1; Molecule: Ovotransferrin; Chain: Null; Synonym: Conalbumin; Heterogen: Iron (F
5	5	0.184	tyrosine 3-monooxygenase/tryptophan 5-monooxygenase activation protein, theta polypeptide [Gallus g
3	3	0.0874	Drebrin (Developmentally-regulated brain protein)
7	7	0.118	actinin, alpha 2 [Gallus gallus]
3	3	0.0507	prolyl endopeptidase [Gallus gallus]
2	3	0.0531	phosphoglucomutase 1 [Gallus gallus]
3	3	NaN	tensin - chicken (fragment)
2	2	0.148	PREDICTED: hypothetical protein [Gallus gallus]
2	2	0.0452	chaperonin containing TCP1, subunit 7 [Gallus gallus]
7	11	0.0609	nestin [Gallus gallus]
3	3	0.277	Cofilin-2 (Cofilin, muscle isoform)
2	2	0.069	PREDICTED: hypothetical protein [Gallus gallus]
2	8	0.241	PREDICTED: hypothetical protein, partial [Gallus gallus]
2	4	0.184	epsilon globin [Gallus gallus]
3	3	0.162	Nuclease sensitive element-binding protein 1 (Y-box-binding protein 1) (Y-box transcription factor)
5	7	0.282	tyrosine 3/tryptophan 5 -monooxygenase activation protein, epsilon polypeptide [Homo sapiens]
2	2	0.0356	heat shock 70kDa protein 9B [Gallus gallus]
2	3	0.0678	PREDICTED: similar to pDJA1 chaperone [Gallus gallus]
6	8	0.0354	talin 1 [Gallus gallus]
3	4	0.0776	prolyl-4-hydroxylase (AA 5 - 494) [Gallus gallus]
2	2	0.149	stathmin 1/oncoprotein 18 [Gallus gallus]
5	6	0.136	heat shock 60kDa protein 1 (chaperonin) [Gallus gallus]
3	3	0.031	actinin, alpha 4 [Gallus gallus]
2	2	0.0985	PREDICTED: hypothetical protein [Gallus gallus]
2	2	0.113	nuclear ubiquitous casein kinase and cyclin-dependent kinase substrate [Gallus gallus]
2	2	0.0586	hypothetical protein LOC418497 [Gallus gallus]

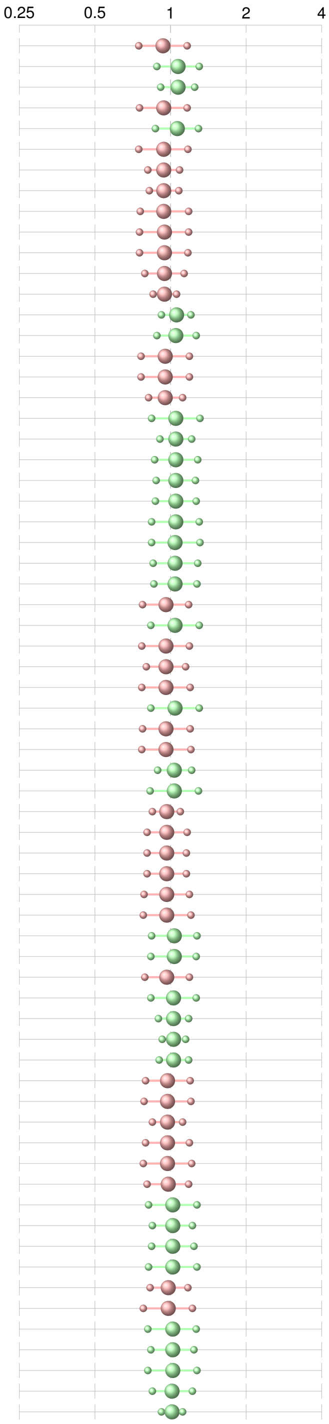

Peptides	Spectra	Coverage	Protein
2	2	0.0425	single stranded D box binding factor 2 - chicken
2	5	0.104	electron transfer flavoprotein, alpha polypeptide [Gallus gallus]
5	9	0.273	slow skeletal ventricular myosin alkali light chain 3 [Gallus gallus]
2	2	0.0239	PREDICTED: similar to Unc-45 homolog B (C. elegans) [Gallus gallus]
3	3	0.155	tyrosine 3-monooxygenase/tryptophan 5-monooxygenase activation protein, zeta polypeptide [Gallus ga
2	2	0.088	RAN, member RAS oncogene family [Gallus gallus]
7	8	0.0398	PREDICTED: similar to spectrin, beta, non-erythrocytic 1 [Gallus gallus]
8	9	0.143	valosin-containing protein [Gallus gallus]
2	2	0.0582	ATP synthase, H+ transporting, mitochondrial F1 complex, beta subunit [Gallus gallus]
2	2	0.0638	eukaryotic translation initiation factor 3, subunit 6 interacting protein [Gallus gallus]
2	2	0.188	hypothetical protein [Gallus gallus]
4	4	0.0254	PREDICTED: cardiomyopathy associated 3 [Gallus gallus]
12	25	NaN	tubulin alpha chain - chicken (fragment)
7	14	0.495	myristoylated alanine-rich C kinase substrate (MARCKS) [Gallus gallus]
4	4	0.0339	PREDICTED: similar to p167 [Gallus gallus]
2	2	0.0424	poly(A) binding protein, cytoplasmic 1 [Gallus gallus]
2	2	0.0595	PREDICTED: hypothetical protein [Gallus gallus]
6	6	0.0897	eukaryotic translation elongation factor 2 [Gallus gallus]
2	2	0.444	thymosin beta 4 [Gallus gallus]
5	10	0.136	pyruvate kinase, muscle [Gallus gallus]
3	3	0.170	peroxiredoxin 6 [Gallus gallus]
3	5	0.0464	PREDICTED: hypothetical protein [Gallus gallus]
3	4	0.067	PREDICTED: eukaryotic translation elongation factor 1 alpha 2 [Gallus gallus]
2	2	0.205	troponin C, slow [Gallus gallus]
2	2	0.248	cytochrome c, somatic [Gallus gallus]
2	4	0.047	calreticulin [Gallus gallus]
3	3	0.17	AK [Gallus gallus]
2	3	0.0269	suppressor of Ty 5 homolog [Gallus gallus]
2	2	0.00439	PREDICTED: similar to fat3 [Gallus gallus]
2	2	0.0193	PREDICTED: laminin, gamma 1 (formerly LAMB2) [Gallus gallus]
4	4	0.0424	clathrin heavy chain 1 [Gallus gallus]
2	2	0.0367	eukaryotic translation elongation factor 1 [Gallus gallus]
2	2	0.0497	hypothetical protein [Gallus gallus]
2	2	0.0274	radixin [Gallus gallus]
2	2	0.174	calmodulin 2 [Homo sapiens]
6	6	0.0637	myosin binding protein C, cardiac [Gallus gallus]
2	2	0.084	p47 protein [Gallus gallus]
9	11	0.206	heat shock 70kDa protein 8 [Gallus gallus]
3	5	0.333	hemoglobin, gamma A [Gallus gallus]
3	6	0.150	ras homolog gene family, member C [Gallus gallus]
4	4	0.179	PREDICTED: similar to natural killer cell enhancing factor isoform 3 [Gallus gallus]
2	3	0.27	Fructose-bisphosphate aldolase C (Brain-type aldolase)
2	2	0.0509	dynein, cytoplasmic, intermediate polypeptide 2 [Gallus gallus]
2	3	0.0809	protein phosphatase 2 (formerly 2A), catalytic subunit, alpha [Gallus gallus]
2	3	0.08	PREDICTED: hypothetical protein [Gallus gallus]
2	3	0.0607	Tubulin beta-3 chain (Beta-tubulin class-IV)
2	3	0.112	microtubule-associated protein, RP/EB family, member 1 [Gallus gallus]
7	9	0.282	triosephosphate isomerase 1 [Gallus gallus]
13	20	0.330	Desmin
8	9	0.27	hypothetical protein [Gallus gallus]
2	4	0.0638	nucleosome assembly protein 1-like 4 [Gallus gallus]
2	2	0.0246	PREDICTED: similar to Leucyl-tRNA synthetase, cytoplasmic (Leucine–tRNA ligase) (LeuRS) [Gallus ga
6	16	0.156	heat shock protein 70 [Gallus gallus]
2	4	0.0154	PREDICTED: hypothetical protein [Gallus gallus]
2	2	0.0277	matrin 3 [Gallus gallus]
3	4	0.126	high-mobility group box 1 [Gallus gallus]
2	2	0.0941	cathepsin B [Gallus gallus]
3	5	0.146	PREDICTED: hypothetical protein [Gallus gallus]
3	3	0.0913	heterogeneous nuclear ribonucleoprotein K [Gallus gallus]
2	2	NaN	tropomyosin alpha chain, skin fibroblast - Japanese quail
4	5	0.119	58kDa glucose regulated protein precursor [Gallus gallus]
2	2	0.0745	ARP1 actin-related protein 1 homolog A, centractin alpha [Gallus gallus]
2	2	0.0584	PREDICTED: hypothetical protein [Gallus gallus]
3	3	0.0706	heat shock 70kDa protein 5 precursor [Gallus gallus]
2	2	0.0554	zyxin [Gallus gallus]
3	5	0.142	PREDICTED: similar to hepatoma-derived growth factor (high-mobility group protein 1-like), partial
14	32	0.387	tubulin, beta 5 [Mus musculus]

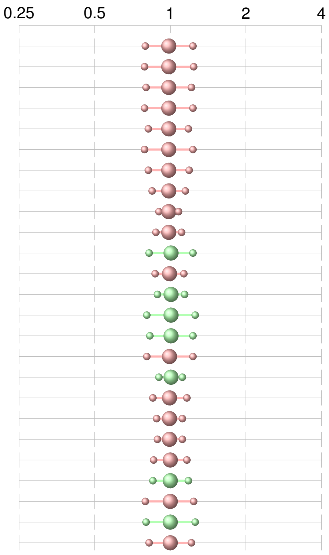

Peptides	Spectra	Coverage	Protein
2	2	0.0918	nucleophosmin 1 [Gallus gallus]
2	2	0.0231	nucleolin [Gallus gallus]
2	3	0.0886	heat shock 70kD protein binding protein [Gallus gallus]
2	2	0.129	glutathione S-transferase class-alpha [Gallus gallus]
3	5	0.0381	PREDICTED: similar to cellular apoptosis susceptibility protein [Gallus gallus]
2	2	0.0269	glutamine-rich 1 [Gallus gallus]
3	4	0.067	protein disulfide isomerase family A, member 4 [Gallus gallus]
6	7	0.101	PREDICTED: hypothetical protein [Gallus gallus]
17	36	0.228	Heat shock protein HSP 90-alpha
10	21	0.466	heat shock 27kDa protein 1 [Gallus gallus]
3	3	0.0492	hsp 108 [Gallus gallus]
9	9	0.048	spectrin, alpha, non-erythrocytic 1 (alpha-fodrin) [Gallus gallus]
9	18	0.215	vimentin [Gallus gallus]
2	2	0.0281	dihydropyrimidinase-like 2 [Gallus gallus]
3	3	0.0708	aminoimidazole-4-carboxamide ribonucleotidetransformylase/IMP cyclohydrolase [Gallus gallus]
2	3	0.0434	PREDICTED: similar to cytosolic NADP-dependent isocitrate dehydrogenase [Gallus gallus]
14	18	0.0854	cgABP260 [Gallus gallus]
4	9	0.0617	Vinculin (Metavinculin)
9	22	NaN	actin type 5, cytosolic - chicken
9	18	0.288	enolase 1 [Gallus gallus]
6	7	0.194	GDP dissociation inhibitor 2 [Gallus gallus]
5	6	0.205	troponin T type 2, cardiac [Gallus gallus]
2	2	0.101	capping protein (actin filament) muscle Z-line, beta [Gallus gallus]
2	2	0.0180	hypoxia up-regulated 1 [Gallus gallus]
3	3	0.0235	myosin, heavy polypeptide 9, non-muscle [Gallus gallus]

4.2 Proteins Identified by a Single Peptide

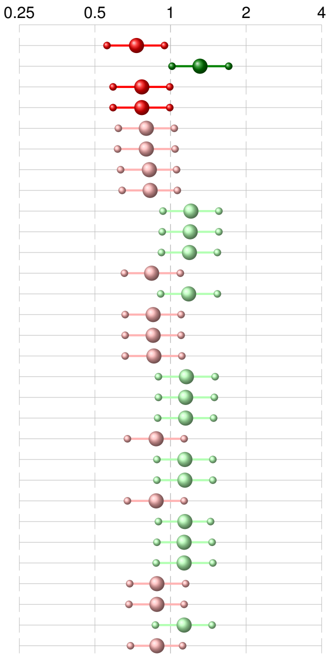

Peptides	Spectra	Coverage	Protein
1	1	0.0367	Fibrinogen beta chain precursor [Contains: Fibrinopeptide B]
1	1	0.104	fatty acid binding protein 5 [Gallus gallus]
1	1	0.0408	PREDICTED: similar to OTTHUMP00000016587, partial [Gallus gallus]
1	1	0.0599	acetyl-Coenzyme A acetyltransferase 2 (acetoacetyl Coenzyme A thiolase) [Gallus gallus]
1	1	0.0146	RNA binding motif protein 6 [Gallus gallus]
1	1	0.108	kinase related protein
1	1	0.0446	PREDICTED: similar to RAD23B protein [Gallus gallus]
1	1	0.0241	PREDICTED: hypothetical protein [Gallus gallus]
1	1	0.0498	PREDICTED: hypothetical protein, partial [Gallus gallus]
1	1	0.0355	fumarate hydratase [Gallus gallus]
1	1	0.03	proteasome (prosome, macropain) 26S subunit, ATPase, 2 [Gallus gallus]
1	1	0.0257	potassium channel tetramerisation domain containing 9 [Gallus gallus]
1	1	0.00978	PREDICTED: similar to Probable phospholipid-transporting ATPase IF (ATPase class I type 11B) (ATPas
1	1	0.0124	hemagglutinin [Influenza A virus (A/chicken/NJ/4236-18/1998(H11N3))]
1	1	0.055	PREDICTED: similar to LanC lantibiotic synthetase component C-like 2 (bacterial) [Gallus gallus]
1	1	0.0240	glutaminase C [Gallus gallus]
1	1	0.0476	PREDICTED: similar to L-3-hydroxyacyl-Coenzyme A dehydrogenase, short chain [Gallus gallus]
1	1	0.0206	gelsolin [Gallus gallus]
1	1	0.0415	Ovalbumin (Egg albumin) (Plakalbumin) (Allergen Gal d 2) (Gal d II)
1	1	0.0518	PREDICTED: hypothetical protein [Gallus gallus]
1	1	0.221	death-associated protein [Gallus gallus]
1	1	0.0905	PREDICTED: hypothetical protein [Gallus gallus]
1	1	0.0163	otokeratin [Gallus gallus]
1	2	0.037	PREDICTED: similar to Malate dehydrogenase 2, NAD (mitochondrial) [Gallus gallus]
1	1	0.2	PREDICTED: similar to Elongation factor Tu, mitochondrial precursor (EF-Tu), partial [Gallus gallus
1	1	0.0412	PREDICTED: similar to MGC83099 protein [Gallus gallus]
1	1	0.00855	PREDICTED: similar to Diacylglycerol kinase, theta [Gallus gallus]
1	1	0.00779	intraflagellar transport 140 homolog [Gallus gallus]
1	1	0.0166	PREDICTED: similar to kinesin heavy chain [Gallus gallus]
1	2	0.0246	PREDICTED: similar to human P5 isoform 2 [Gallus gallus]

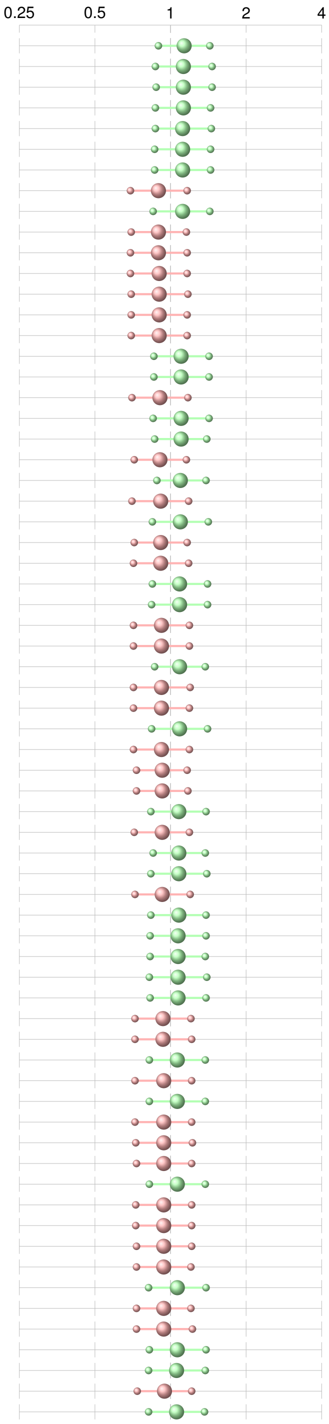

Peptides	Spectra	Coverage	Protein
1	2	0.067	PREDICTED: similar to Murr1 [Gallus gallus]
1	1	0.0337	PREDICTED: similar to aflatoxin aldehyde reductase [Gallus gallus]
1	1	0.00818	collagen, type XVIII, alpha 1 [Gallus gallus]
1	1	0.0784	heat shock 10kDa protein 1 (chaperonin 10) [Gallus gallus]
1	1	0.055	PREDICTED: hypothetical protein [Gallus gallus]
1	1	0.0114	coatomer protein complex, subunit gamma [Gallus gallus]
1	1	0.0942	PREDICTED: hypothetical protein [Gallus gallus]
1	1	0.0548	PREDICTED: hypothetical protein [Gallus gallus]
1	1	0.0125	PREDICTED: similar to Cullin-4B (CUL-4B) [Gallus gallus]
1	1	0.0283	PREDICTED: similar to stanniocalcin [Gallus gallus]
1	1	0.0623	PREDICTED: similar to thioredoxin-like protein [Gallus gallus]
1	1	0.0313	PREDICTED: similar to alpha-tubulin 8 [Gallus gallus]
1	1	0.00197	basement membrane-specific heparan sulfate proteoglycan core protein [Gallus gallus]
1	1	0.0265	vitronectin [Gallus gallus]
1	1	0.00545	filamin B, beta (actin binding protein 278) [Gallus gallus]
1	1	0.0058	PREDICTED: hypothetical protein [Gallus gallus]
1	1	0.0515	PREDICTED: hypothetical protein [Gallus gallus]
1	1	0.1	PREDICTED: similar to PDGF associated protein [Gallus gallus]
1	1	0.114	PREDICTED: hypothetical protein [Gallus gallus]
1	2	0.0363	Trypsin I-P38 precursor
1	2	0.058	ubiquitin carboxyl-terminal esterase L1 (ubiquitin thiolesterase) [Gallus gallus]
1	3	0.037	PREDICTED: similar to Aldehyde dehydrogenase 9 family, member A1 [Gallus gallus]
1	1	0.119	histidine triad nucleotide binding protein 1 [Gallus gallus]
1	1	0.0212	acyl-Coenzyme A dehydrogenase family, member 9 [Gallus gallus]
1	2	0.0575	small glutamine-rich tetratricopeptide [Gallus gallus]
1	1	0.0105	PREDICTED: similar to insulin-degrading enzyme [Gallus gallus]
1	1	0.0110	thrombospondin 2 [Gallus gallus]
1	1	0.0211	PREDICTED: similar to serotonin receptor [Gallus gallus]
1	1	0.0233	t-complex 1 [Gallus gallus]
1	1	0.0138	DEAD/H (Asp-Glu-Ala-Asp/His) box polypeptide 3 [Gallus gallus]
1	2	0.104	PREDICTED: similar to nuclear poly(C)-binding protein, splicevariant E, partial [Gallus gallus]
1	1	0.0292	seryl-tRNA synthetase [Gallus gallus]
1	1	0.0209	tyrosyl-tRNA synthetase [Gallus gallus]
1	1	0.1	Chain B, Smooth Muscle Myosin Motor Domain-Essential Light Chain Complex With Mgadp.Alf4 Bound At T
1	1	0.0277	PREDICTED: similar to leucine-rich repeat-containing F-box protein FBL3A [Gallus gallus]
1	2	0.103	PREDICTED: similar to ionized calcium binding adapter molecule 2 (Iba2) [Gallus gallus]
1	2	0.0179	PREDICTED: similar to cystathionine beta-synthase [Gallus gallus]
1	1	0.109	S100 calcium binding protein A11 [Gallus gallus]
1	1	0.0469	PREDICTED: hypothetical protein, partial [Gallus gallus]
1	2	0.0378	PREDICTED: similar to hepatocellular carcinoma susceptibility protein [Gallus gallus]
1	1	0.0159	SKI-like [Gallus gallus]
1	1	0.0422	PREDICTED: hypothetical protein [Gallus gallus]
1	1	0.0957	PREDICTED: hypothetical protein [Gallus gallus]
1	1	0.0326	pyruvate dehydrogenase (lipoamide) alpha 1 [Gallus gallus]
1	1	0.117	glutaredoxin 5 homolog [Gallus gallus]
1	1	0.0305	PREDICTED: similar to MGC97820 protein [Gallus gallus]
1	1	0.0675	protein phosphatase 1, regulatory (inhibitor) subunit 2 [Gallus gallus]
1	1	0.0140	PREDICTED: similar to acid alpha glucosidase [Gallus gallus]
1	1	0.0141	hypothetical protein [Gallus gallus]
1	1	0.0292	PREDICTED: similar to NIPA-like domain containing 3 [Gallus gallus]
1	1	0.0227	adenylosuccinate lyase [Gallus gallus]
1	1	0.0312	acyl-Coenzyme A dehydrogenase, C-2 to C-3 short chain [Gallus gallus]
1	1	0.0171	PREDICTED: similar to endo-beta-N-acetylglucosaminidase [Gallus gallus]
1	1	0.00914	PREDICTED: similar to rab effector MYRIP [Gallus gallus]
1	1	0.06	huntingtin interacting protein 2 [Gallus gallus]
1	1	0.0427	PREDICTED: hypothetical protein [Gallus gallus]
1	1	0.00993	myosin IG [Gallus gallus]
1	1	0.0187	PREDICTED: similar to cell adhesion molecule JCAM [Gallus gallus]
1	1	0.0212	Ras-GTPase-activating protein SH3-domain-binding protein [Gallus gallus]
1	1	0.116	myosin regulatory light chain 9 [Gallus gallus]
1	1	0.0389	PREDICTED: similar to Vacuolar protein sorting 36 (yeast) [Gallus gallus]
1	1	0.00745	PREDICTED: hypothetical protein [Gallus gallus]
1	1	0.00643	PREDICTED: similar to ELKS epsilon isoform 1 [Gallus gallus]
1	1	0.308	PREDICTED: similar to damaged-DNA binding protein DDB p127 subunit [Gallus gallus]
1	1	0.0442	osteoglycin [Gallus gallus]
1	1	0.00776	PREDICTED: similar to KIAA0835 protein [Gallus gallus]
1	1	0.0556	PREDICTED: similar to Phospholipase A2, membrane associated precursor (Phosphatidylcholine 2-acylhy

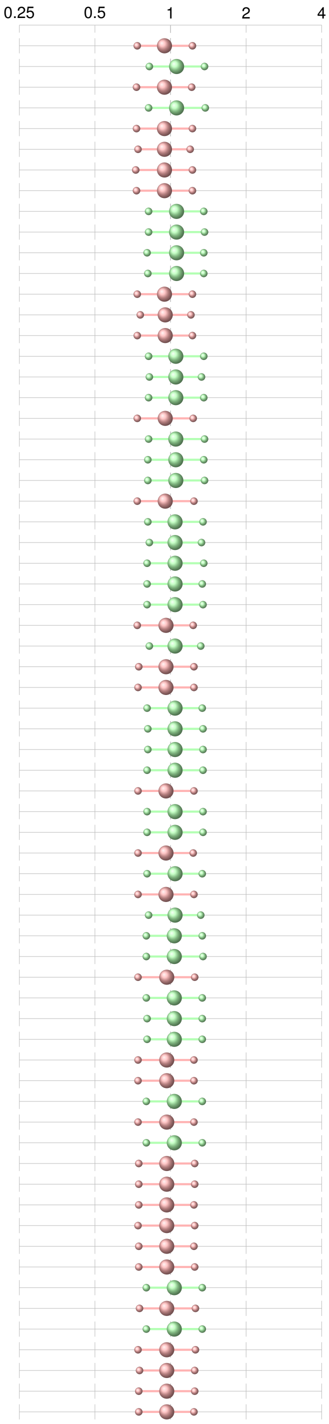

Peptides	Spectra	Coverage	Protein
1	1	0.0109	PREDICTED: similar to Ubiquitin carboxyl-terminal hydrolase 47 (Ubiquitin thioesterase 47) (Ubiquit
1	1	0.0095	PREDICTED: similar to KIAA1571 protein [Gallus gallus]
1	1	0.0355	PREDICTED: similar to taxilin [Gallus gallus]
1	1	0.0182	mitochondrial trifunctional protein, alpha subunit [Gallus gallus]
1	1	0.00718	PREDICTED: similar to Clathrin, heavy polypeptide (Hc) [Gallus gallus]
1	2	0.0736	S-phase kinase-associated protein 1A isoform b [Homo sapiens]
1	1	0.0319	putative coat protein [Chickpea yellow mosaic virus]
1	1	0.0229	PREDICTED: hypothetical protein [Gallus gallus]
1	1	0.0332	testin [Gallus gallus]
1	1	0.0224	zinc finger protein 85 (HPF4, HTF1) [Gallus gallus]
1	1	0.0162	kinesin heavy chain member 2A [Gallus gallus]
1	1	0.00586	PREDICTED: similar to RP11-163B6.3 [Gallus gallus]
1	1	0.114	acid phosphatase 1, soluble [Gallus gallus]
1	2	0.0543	mitogen-activated protein kinase kinase 3 [Gallus gallus]
1	1	0.00957	PREDICTED: similar to natural killer tumor recognition protein [Gallus gallus]
1	1	0.0142	PREDICTED: similar to KIAA0066 [Gallus gallus]
1	2	0.0366	PREDICTED: similar to 5-nucleotidase domain containing 2 [Gallus gallus]
1	1	0.0184	PREDICTED: similar to neuronal transmembrane protein Slitrk4 [Gallus gallus]
1	1	0.0394	pyruvate dehydrogenase kinase, isoenzyme 3 [Gallus gallus]
1	1	0.0186	Lysyl-tRNA synthetase [Gallus gallus]
1	1	0.0462	hypothetical protein LOC734509 [Xenopus laevis]
1	1	0.0155	heterogeneous nuclear ribonucleoprotein M [Gallus gallus]
1	1	0.0286	bleomycin hydrolase [Gallus gallus]
1	1	0.0915	non-metastatic cells 2, protein (NM23B) expressed in [Gallus gallus]
1	2	0.0228	connexin 45 [Gallus gallus]
1	1	0.0786	profilin 2 [Gallus gallus]
1	1	0.222	PREDICTED: hypothetical protein, partial [Gallus gallus]
1	1	0.101	p32 subunit of splicing factor SF2 [Gallus gallus]
1	1	0.0213	PREDICTED: hypothetical protein [Gallus gallus]
1	2	0.0365	DnaJ (Hsp40) homolog, subfamily A, member 2 [Gallus gallus]
1	1	0.0379	PREDICTED: similar to putative pre-mRNA splicing regulator female-lethal(2D) [Gallus gallus]
1	1	0.0242	PREDICTED: hypothetical protein [Gallus gallus]
1	1	0.0576	cell division cycle 42 (GTP binding protein, 25kDa) [Gallus gallus]
1	1	0.0314	tubulin, beta 6 [Gallus gallus]
1	1	0.088	hypothetical protein LOC420709 [Gallus gallus]
1	1	0.0985	fatty acid binding protein 7, brain [Gallus gallus]
1	1	0.037	sirtuin [Gallus gallus]
1	1	0.0116	PREDICTED: similar to basement membrane-specific heparan sulfate proteoglycan core protein, partial
1	1	0.00706	SMC4 structural maintenance of chromosomes 4-like 1 [Gallus gallus]
1	1	0.0773	proteasome (prosome, macropain) 26S subunit, non-ATPase, 9 [Gallus gallus]
1	1	0.066	PREDICTED: similar to GTP binding protein overexpressed in skeletal muscle [Gallus gallus]
1	1	0.0567	lectin-associated matrix protein [Gallus gallus]
1	2	0.0160	SAPS domain family, member 3 [Gallus gallus]
1	1	0.0183	vacuolar protein sorting 26 [Mus musculus]
1	1	0.0477	Chain A, Complex Between Rabbit Muscle Alpha-Actin: Human Gelsolin Domain 1
1	1	0.0239	glycinamide ribonucleotide synthetase-aminoimidazole ribonucleotide synthetase-glycinamide ribonucl
1	1	0.0186	PREDICTED: similar to Alanyl-tRNA synthetase domain containing 1 [Gallus gallus]
1	1	0.0152	PREDICTED: hypothetical protein [Gallus gallus]
1	1	0.0464	apolipoprotein A-IV [Gallus gallus]
1	1	0.0464	adipose differentiation-related protein [Gallus gallus]
1	1	0.00336	PREDICTED: similar to neuroblastoma-amplified protein [Gallus gallus]
1	1	0.0552	coatomer protein complex, subunit epsilon [Gallus gallus]
1	1	0.0308	hypothetical protein [Gallus gallus]
1	1	0.00723	myosin heavy chain 6 [Gallus gallus]
1	1	0.0138	PREDICTED: similar to partner of PIX 1 [Gallus gallus]
1	1	0.0348	PREDICTED: hypothetical protein [Gallus gallus]
1	1	0.00988	PREDICTED: similar to Cxorf5 (71-7A) protein [Gallus gallus]
1	1	0.05	RNA binding protein with multiple splicing 2 [Gallus gallus]
1	1	0.00952	PREDICTED: similar to Heat-shock protein 105 kDa (Heat shock 110 kDa protein) (Antigen NY-CO-25) [G
1	1	0.0105	hect domain and RLD 3 [Gallus gallus]
1	1	0.049	cytokine induced apoptosis inhibitor 1 [Gallus gallus]
1	1	0.0492	troponin I, skeletal, fast [Gallus gallus]
1	1	0.0181	PREDICTED: hypothetical protein [Gallus gallus]
1	1	0.0358	PREDICTED: hypothetical protein [Gallus gallus]
1	1	0.0638	Rous sarcoma virus transcription enhancer factor II [Gallus gallus]
1	1	0.0120	calpastatin [Gallus gallus]
1	1	0.0142	heat shock 70kDa protein 4-like [Gallus gallus]

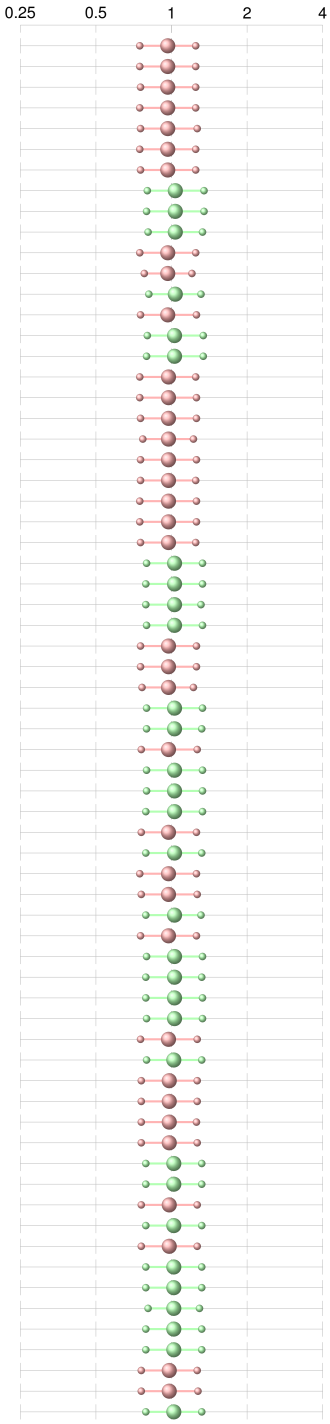

Peptides	Spectra	Coverage	Protein
1	1	0.0255	PREDICTED: hypothetical protein [Gallus gallus]
1	1	0.0098	PREDICTED: similar to KIBRA protein [Gallus gallus]
1	1	0.0164	adducin 1 (alpha) [Gallus gallus]
1	1	0.00872	PREDICTED: hypothetical protein [Gallus gallus]
1	1	0.0295	PREDICTED: similar to Misato homolog 1 (Drosophila) [Gallus gallus]
1	1	0.0334	calcium/calmodulin-dependent protein kinase (CaM kinase) II delta [Gallus gallus]
1	1	0.0174	heterogeneous nuclear ribonucleoprotein R [Gallus gallus]
1	1	0.0493	transcription elongation factor A 1 [Gallus gallus]
1	1	0.0649	chromobox homolog 1 (HP1 beta homolog Drosophila) [Gallus gallus]
1	1	0.0216	COBW domain containing protein1 [Gallus gallus]
1	1	0.00567	PREDICTED: similar to Rap guanine nucleotide exchange factor (GEF) 3 [Gallus gallus]
1	4	0.0202	tubulin, beta 2B [Gallus gallus]
1	2	1	heterogeneous nuclear ribonucleoprotein U, MARs binding protein p120, hnRNP U=matrix attachment reg
1	1	0.0146	PREDICTED: hypothetical protein [Gallus gallus]
1	1	0.0283	annexin A6 [Gallus gallus]
1	1	0.0463	FK506 binding protein 1A, 12kDa [Gallus gallus]
1	1	0.0153	PREDICTED: carnitine palmitoyltransferase II [Gallus gallus]
1	1	0.122	hematological and neurological expressed 1 [Gallus gallus]
1	1	0.111	hypothetical protein LOC427269 [Gallus gallus]
1	2	0.0242	PREDICTED: hypothetical protein [Gallus gallus]
1	1	0.0178	PREDICTED: similar to TBC1 (tre-2/USP6, BUB2, cdc16) domain family, member 1 [Gallus gallus]
1	1	0.0188	PREDICTED: hypothetical protein, partial [Gallus gallus]
1	1	0.0511	PREDICTED: similar to ragA [Gallus gallus]
1	1	0.0513	PREDICTED: similar to Fascin homolog 1, actin-bundling protein (Strongylocentrotus purpuratus) [Gal
1	1	0.0524	Chain A, Triosephosphate Isomerase From Gallus Gallus, Loop 6 Mutant T175v
1	1	0.0377	PREDICTED: similar to potassium channel tetramerization domain-containing 1 isoform 1 [Gallus gallu
1	1	0.0366	Chain A, Chicken Citrate Synthase Complex With Trifluoroacetonyl-Coa And Citrate
1	1	0.0093	PREDICTED: similar to phosphoinositol 3-phosphate-binding protein-2 [Gallus gallus]
1	1	0.0224	alpha-actinin [Gallus gallus]
1	1	0.0558	C-type lectin-like receptor [Gallus gallus]
1	1	0.0339	PREDICTED: similar to U-snRNP-associated cyclophilin [Gallus gallus]
1	2	0.0147	PREDICTED: similar to C219-reactive peptide (FLJ39207) [Gallus gallus]
1	1	0.0327	PREDICTED: similar to chaperonin-containing TCP-1 complex gamma chain [Gallus gallus]
1	1	0.0124	myomesin (M-protein) 2, 165kDa [Gallus gallus]
1	1	0.0314	chaperonin containing TCP1, subunit 5 (epsilon) [Gallus gallus]
1	1	0.0106	coatomer protein complex, subunit alpha [Gallus gallus]
1	1	0.0265	hypothetical protein LOC422936 [Gallus gallus]
1	1	0.00698	PREDICTED: similar to RSK-like protein [Gallus gallus]
1	1	0.0268	PREDICTED: similar to yeast sds22 homolog [Gallus gallus]
1	1	0.0783	PA polymerase protein [Influenza A virus (A/Chicken/Italy/5945/95(H3N2))]
1	1	0.00667	PREDICTED: similar to palladin [Gallus gallus]
1	1	0.0092	PREDICTED: similar to putative acetyltransferase [Gallus gallus]
1	1	0.00973	PREDICTED: similar to KIAA0316 protein [Gallus gallus]
1	1	0.105	PREDICTED: hypothetical protein [Gallus gallus]
1	1	0.034	PREDICTED: similar to FLJ39378 protein isoform 1 [Gallus gallus]
1	1	0.0110	PREDICTED: similar to Ogfr protein [Gallus gallus]
1	1	0.0196	PREDICTED: similar to caspase recruitment domain protein 9 [Gallus gallus]
1	1	0.0218	PREDICTED: similar to Pleckstrin homology domain containing, family C (with FERM domain) member 1 i
1	1	0.0164	signal recognition particle 72kDa [Gallus gallus]
1	1	0.0511	toll interacting protein [Gallus gallus]
1	1	0.0356	cell division cycle 37 protein [Gallus gallus]
1	1	0.0150	PREDICTED: similar to plasminogen [Gallus gallus]
1	1	0.068	PREDICTED: hypothetical protein [Gallus gallus]
1	1	0.00966	PREDICTED: similar to mKIAA0681 protein [Gallus gallus]
1	1	0.0348	3-oxoacid CoA transferase 1 [Gallus gallus]
1	1	0.00564	PREDICTED: similar to axonal transport of synaptic vesicles [Gallus gallus]
1	1	0.184	hypothetical protein [Gallus gallus]
1	1	0.101	COP9 constitutive photomorphogenic homolog subunit 8 [Gallus gallus]
1	1	0.0769	hypothetical protein [Gallus gallus]
1	1	0.0259	PREDICTED: hypothetical protein [Gallus gallus]
1	1	0.0755	PREDICTED: similar to MGC82793 protein isoform 2 [Gallus gallus]
1	2	0.00435	PREDICTED: similar to multiple ankyrin repeats single KH domain protein [Gallus gallus]
1	1	0.026	PREDICTED: similar to KDEL (Lys-Asp-Glu-Leu) containing 1 [Gallus gallus]
1	1	0.0274	dynamitin [Gallus gallus]
1	1	0.293	PREDICTED: hypothetical protein, partial [Gallus gallus]
1	1	0.0306	PREDICTED: similar to cyclophilin [Gallus gallus]
1	1	0.109	hypothetical protein [Gallus gallus]

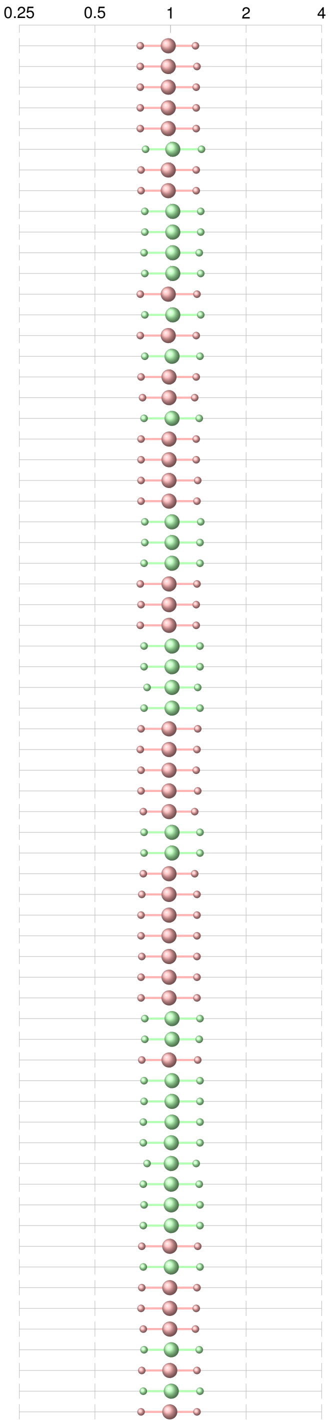

Peptides	Spectra	Coverage	Protein
1	1	0.0129	PREDICTED: similar to N-acetylglucosamine-phosphate mutase [Gallus gallus]
1	1	0.0208	hypothetical protein [Gallus gallus]
1	1	0.0113	hypothetical protein LOC424451 [Gallus gallus]
1	1	0.0114	PREDICTED: similar to MGC84609 protein [Gallus gallus]
1	1	0.0346	PREDICTED: hypothetical protein [Gallus gallus]
1	1	0.0252	PREDICTED: similar to glutathione synthetase [Gallus gallus]
1	1	0.00459	PREDICTED: similar to ATM [Gallus gallus]
1	1	0.0310	adenylosuccinate synthase [Gallus gallus]
1	1	0.0427	PREDICTED: similar to Acidic leucine-rich nuclear phosphoprotein 32 family member A (Leucine-rich a
1	1	0.0245	peptidylprolyl isomerase F [Gallus gallus]
1	1	0.066	PREDICTED: similar to Deoxythymidylate kinase (thymidylate kinase) [Gallus gallus]
1	1	0.157	hypothetical protein LOC426942 [Gallus gallus]
1	1	0.0205	nuclear distribution gene C homolog [Gallus gallus]
1	1	0.0902	PREDICTED: hypothetical protein [Gallus gallus]
1	1	0.0221	PREDICTED: similar to Phosphopantothenoylcysteine synthetase [Gallus gallus]
1	1	0.06	PREDICTED: hypothetical protein isoform 1 [Gallus gallus]
1	1	0.023	phosphofructokinase, platelet [Gallus gallus]
1	2	0.0526	PREDICTED: similar to CGI-83 protein, partial [Gallus gallus]
1	1	0.00514	PREDICTED: similar to Tsg24 protein [Gallus gallus]
1	1	0.0085	PREDICTED: hypothetical protein [Gallus gallus]
1	1	0.0232	Retinal dehydrogenase 2 (RalDH2) (RALDH 2) (RALDH(II)) (Retinaldehyde-specific dehydrogenase type 2
1	1	0.0608	PREDICTED: similar to TFG protein [Gallus gallus]
1	1	0.0553	PREDICTED: similar to cdc21p, partial [Gallus gallus]
1	1	0.122	cystatin C [Gallus gallus]
1	1	0.0199	aspartyl-tRNA synthetase [Gallus gallus]
1	1	0.171	Ubiquitin
1	1	0.0490	PREDICTED: similar to Prdx3 protein [Gallus gallus]
1	1	0.0139	PREDICTED: similar to Band 4.1-like protein 3 (4.1B) (Differentially expressed in adenocarcinoma of
1	1	0.0717	hypothetical protein LOC422173 [Gallus gallus]
1	1	0.0118	PREDICTED: similar to Eukaryotic translation elongation factor 1 delta (guanine nucleotide exchange
1	1	0.0153	hook homolog 1 [Gallus gallus]
1	2	0.0072	PREDICTED: similar to hephaestin [Gallus gallus]
1	1	0.00383	PREDICTED: similar to E3 ubiquitin-protein ligase HECTD1 (HECT domain-containing protein 1) (E3 lig
1	1	0.0203	signal transducer and activator of transcription 5B [Gallus gallus]
1	1	0.112	tumor protein D52-like 2 [Gallus gallus]
1	1	0.0206	PREDICTED: similar to polyglutamine-containing protein [Gallus gallus]
1	1	0.0443	acyl-Coenzyme A binding domain containing 3 [Gallus gallus]
1	2	0.0344	eukaryotic translation initiation factor 4A2 [Gallus gallus]
1	1	0.0194	leucine zipper protein [Gallus gallus]
1	1	0.0293	PREDICTED: similar to RP11-575L7.5 isoform 1 [Gallus gallus]
1	2	0.0112	aconitase 1, soluble [Gallus gallus]
1	1	0.0528	hypothetical protein [Gallus gallus]
1	1	0.0591	proteasome (prosome, macropain) subunit, beta type, 1 [Gallus gallus]
1	1	0.0917	histone H3 [Euperipatoides leuckartii]
1	1	0.0187	oxysterol binding protein-like 2 [Gallus gallus]
1	1	0.00504	PREDICTED: similar to putative protein kinase [Gallus gallus]
1	1	0.0327	fibulin 1 [Gallus gallus]
1	1	0.055	ras-related GTP-binding protein RAB10 [Gallus gallus]
1	1	0.0408	cytidylate kinase [Gallus gallus]
1	1	0.069	PREDICTED: similar to Oxysterol-binding protein 1, partial [Gallus gallus]
1	1	0.0463	PREDICTED: hypothetical protein [Gallus gallus]
1	1	0.0263	PREDICTED: similar to actin-related protein 3-beta [Gallus gallus]
1	1	0.0143	PREDICTED: similar to guanine nucleotide exchange factor Lbc [Gallus gallus]
1	1	0.0276	hypothetical protein [Gallus gallus]
1	3	0.105	SH3 domain binding glutamic acid-rich protein like [Gallus gallus]
1	1	0.0271	methionyl aminopeptidase 2 [Gallus gallus]
1	1	0.0272	PREDICTED: similar to Peptidylprolyl isomerase domain and WD repeat containing 1 [Gallus gallus]
1	1	0.0255	archain 1 [Gallus gallus]
1	1	0.140	PREDICTED: similar to P1725 [Gallus gallus]
1	1	0.0156	eukaryotic translation initiation factor 5B [Gallus gallus]
1	1	0.0435	non-histone chromosomal protein [Gallus gallus]
1	1	0.048	GTP-binding protein PTD004 [Gallus gallus]
1	2	0.0142	alpha 1 type II procollagen [Gallus gallus]
1	1	0.0280	taste receptor, type 2, member 7 [Gallus gallus]
1	1	0.0365	PREDICTED: hypothetical protein [Gallus gallus]
1	1	0.0413	PREDICTED: hypothetical protein [Gallus gallus]
1	1	0.0474	PREDICTED: hypothetical protein [Gallus gallus]

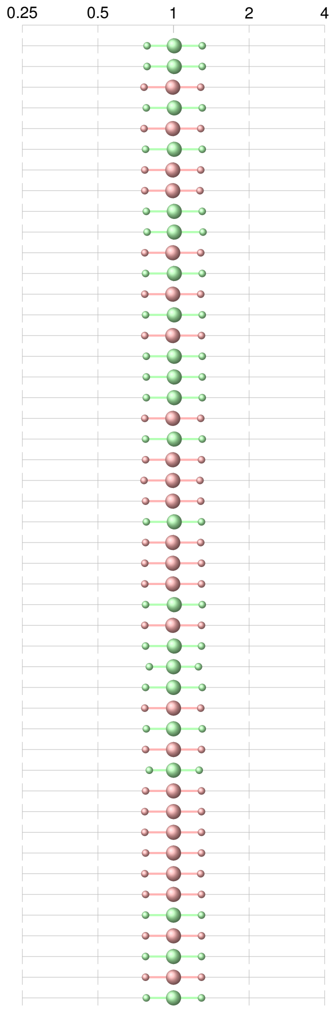

Peptides	Spectra	Coverage	Protein
1	1	0.0192	adaptor-related protein complex 2, alpha 2 subunit [Gallus gallus]
1	1	0.0198	nuclear calmodulin-binding protein [Gallus gallus]
1	1	0.0207	heat shock 90kDa protein 1, beta [Gallus gallus]
1	1	0.056	6-phosphogluconolactonase [Gallus gallus]
1	1	0.015	PREDICTED: similar to p80-coilin [Gallus gallus]
1	1	NaN	homeotic protein Hoxa-2 - chicken
1	1	0.0131	WD repeat domain 1 [Gallus gallus]
1	1	0.0127	PREDICTED: similar to ATP-dependent Lon protease [Gallus gallus]
1	1	0.00793	polymerase basic protein 1 [Influenza A virus (A/chicken/Thailand/PC-170/2006(H5N1))]
1	1	0.0359	trimethyllysine hydroxylase, epsilon [Gallus gallus]
1	1	0.0131	alanyl-tRNA synthetase [Gallus gallus]
1	1	0.00628	restin [Gallus gallus]
1	1	0.0235	PDZ and LIM domain 5 [Gallus gallus]
1	1	0.074	Parkinson disease (autosomal recessive, early onset) 7 [Gallus gallus]
1	1	0.00633	PREDICTED: similar to translocated promoter region (to activated MET oncogene) [Gallus gallus]
1	1	0.0273	PREDICTED: similar to MGC84017 protein [Gallus gallus]
1	1	0.146	PREDICTED: hypothetical protein, partial [Gallus gallus]
1	1	0.0149	PREDICTED: G elongation factor, mitochondrial 1 [Gallus gallus]
1	1	0.0157	PREDICTED: similar to Leucine rich repeat containing 33 [Gallus gallus]
1	1	0.00565	PREDICTED: hypothetical protein [Gallus gallus]
1	1	0.0321	PREDICTED: hypothetical protein [Gallus gallus]
1	1	0.0251	PREDICTED: similar to blood island enriched kruppel like factor [Gallus gallus]
1	1	0.0128	rabaptin, RAB GTPase binding effector protein 1 [Gallus gallus]
1	1	0.0261	hypothetical protein FLJ10656 [Gallus gallus]
1	1	0.0090	PREDICTED: similar to Zinc finger SWIM domain-containing protein 5 [Gallus gallus]
1	1	0.0175	PREDICTED: hypothetical protein [Gallus gallus]
1	1	NaN	PREDICTED: similar to cadherin-8 [Gallus gallus]
1	1	0.0192	UBP [Gallus gallus]
1	1	NaN	glutathione transferase (EC 2.5.1.18) mu2 - chicken
1	1	0.0335	Tubulin alpha-5 chain
1	4	0.0504	vimentin [Gallus gallus]
1	1	0.0424	PREDICTED: similar to Adprhl1-prov protein [Gallus gallus]
1	1	0.0478	PREDICTED: similar to Phosphoribosyl pyrophosphate synthetase-associated protein 1 [Gallus gallus]
1	1	0.0176	vacuolar protein sorting 35 [Gallus gallus]
1	1	0.0141	PREDICTED: similar to phosphofructokinase isoform 1 [Gallus gallus]
1	3	0.006	PREDICTED: similar to pleckstrin homology domain containing, family H (with MyTH4 domain) member 2
1	1	0.066	fibronectin [Gallus gallus]
1	1	0.0208	PREDICTED: similar to serine palmitoyltransferase, long chain base subunit 1 [Gallus gallus]
1	1	0.0116	PREDICTED: similar to Adaptor-related protein complex 2, beta 1 subunit [Gallus gallus]
1	1	0.0442	PREDICTED: similar to Human Diff6,H5,CDC10 homologue [Gallus gallus]
1	1	0.0194	PREDICTED: similar to KIAA1734 protein [Gallus gallus]
1	1	0.127	signal recognition particle 14kDa (homologous Alu RNA binding protein) [Gallus gallus]
1	1	0.00395	PREDICTED: polymerase (DNA directed), epsilon [Gallus gallus]
1	1	0.0208	PREDICTED: hypothetical protein [Gallus gallus]
1	1	0.0131	hexokinase 2 [Gallus gallus]
1	1	0.0342	PREDICTED: hypothetical protein, partial [Gallus gallus]
1	1	0.0974	ubiquitin-conjugating enzyme E2L 3 isoform 1 [Homo sapiens]

5 Protein Details

A detailed summary of each protein is given below. These sections include peptide relative expression estimates in addition to protein-level estimates.

5.1 PREDICTED: similar to Myoglobin [Gallus gallus]

Protein Accession	gi|50728806
Mean Expression Ratio	2.14
Median Expression Ratio	2.15
Credible Interval	(1.76, 2.61)
Associated Peptides	3
Associated Spectra	4
Coverage	0.240

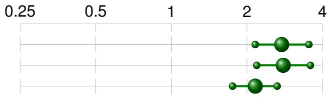

A	2.5	50	97.5	Sequence
1	2.2	2.8	3.5	EFGFQG
1	2.2	2.8	3.6	GNHESELKPLAQTHATK
2	1.8	2.2	2.6	LFHDHPETLDRFDK

```
1      MGLSDQEWQQ VLTIWGKVEA DIAGHGHEVL MRLFHDHPET LDRFDKFKGL KTPDQMKGSE DLKKHGATVL TQLGKILKQK
81     GNHESELKPL AQTHATKHKI PVKYLEFISE VIIKVIAEKH AADFGADSQA AMKKALELFR NDMASKYKEF GFQG
```

5.2 Fibrinogen beta chain precursor [Contains: Fibrinopeptide B]

Protein Accession	gi|399491
Mean Expression Ratio	0.731
Median Expression Ratio	0.731
Credible Interval	(0.559, 0.945)
Associated Peptides	1
Associated Spectra	1
Coverage	0.0367

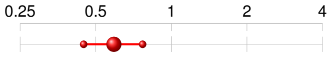

A	2.5	50	97.5	Sequence
1	0.45	0.59	0.77	ASVEYDNEEDSPQIDAR

```
1      ASVEYDNEED SPQIDARAHR PLDKRQEAAP TLRPVAPPIS GTGYQPRPPK QDKQAMKKGP IIYPDAGGCK HPLDELGVLC
81     PTGCELQTTL LKQEKTVKPV LRDLKDRVAK FSDTSTTMYQ YVNMIDNKLV KTQKQRKDND IILSEYNTEM ELHYNYIKDN
161    LDNNIPSSLR VLRAVIDSLH KKIQKLENAI ATQTDYCRSP CVASCNIPVV SGRECEDIYR KGGETSEMYI IQPDPFTTPY
241    RVYCDMETDN GGWTLIQNRQ DGSVNFGRAW DEYKRGFGNI AKSGGKKYCD TPGEYWLGND KISQLTKIGP TKVLIEMEDW
321    NGDKVSALYG GFTIHNEGNK YQLSVSNYKG NAGNALMEGA SQLYGENRTM TIHNGMYFST YDRDNDGWLT TDPRKQCSKE
401    DGGGWWYNRC HAANPNGRYY WGGTYSWDMA KHGTDDGIVW MNWKGSWYSM KKMSMKIKPY FPD
```

5.3 apolipoprotein A-I [Gallus gallus]

Protein Accession	gi|45382961
Mean Expression Ratio	0.732
Median Expression Ratio	0.732
Credible Interval	(0.631, 0.849)
Associated Peptides	6
Associated Spectra	8
Coverage	0.220

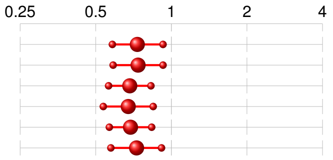

A	2.5	50	97.5	Sequence
1	0.58	0.73	0.92	NLAPYSDELR
1	0.59	0.74	0.93	LISFLDELQK
2	0.56	0.68	0.83	QKVELMQAK
1	0.54	0.68	0.85	LREDMAPYYK
2	0.57	0.69	0.83	IRPFLDQFSAK
1	0.58	0.73	0.91	DLEEVKEK

```
1      MRGVLVTLAV LFLTGTQARS FWQHDEPQTP LDRIRDMVDV YLETVKASGK DAIAQFESSA VGKQLDLKLA DNLDTLSAAA
81     AKLREDMAPY YKEVREMWLK DTEALRAELT KDLEEVKEKI RPFLDQFSAK WTEELEQYRQ RLTPVAQELK ELTKQKVELM
161    QAKLTPVAEE ARDRLRGHVE ELRKNLAPYS DELRQKLSQK LEEIREKGIP QASEYQAKVM EQLSNLREKM TPLVQEFRER
241    LTPYAENLKN RLISFLDELQ KSVA
```

5.4 Alpha-fetoprotein precursor (Alpha-fetoglobulin) (Alpha-1-fetoprotein)

Protein Accession	gi|59797643
Mean Expression Ratio	0.743
Median Expression Ratio	0.743
Credible Interval	(0.62, 0.89)
Associated Peptides	4
Associated Spectra	4
Coverage	0.0894

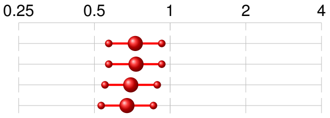

A	2.5	50	97.5	Sequence
1	0.57	0.72	0.93	SECIVNAENDDKPANLSPQVR
1	0.57	0.73	0.93	HIYETESVMK
1	0.55	0.7	0.89	KCTEVDRDNPNCR
1	0.53	0.67	0.86	VVKPIREDGLR

```
1     MAVLPLSGAI RLSRGDLVIE TQTQDPWAKK LITQGTDKAC ADLDVQQIQA LKMKCAMIMF AQYVQGNTFG QVVKMAEAVT
81    DLAKKCTEVD RDNPNCRKPL DWIFLNTICQ EDNLPRFTDC CAKKDPERND CFLSLKNSSR GFISPFERPN AEAACKNYSE
161   HRHSLPGYFI YEVSRRHPFL YAPTILSVAI HYDEMMKDCC RSAEDSTHNL EECFRRQAPK VVKPIREDGL RQEHTCGILK
241   KFGERTIKAL KLVQISQRFP KADFFTVTKL VSDIANMHKD CCRGDMLECM RDREEILHYV CTNQDVISSK IKKCCEKPLL
321   QRSECIVNAE NDDKPANLSP QVREFIEDKG ICERFAQEKD THLARFLYEY SRRHPEFSAQ MLLRIGKGYE DLLDECCKTG
401   SPDNCCSRGE EELKKHIYET ESVMKTSCDI YKEKGDYYFQ NEYIKFTKQM TTIGSKCCQL SQDKLLPCAE ENVSLLVDLV
481   LGEICRRHLT NPINPAVCHC CSSSYALRRP CMGKLEIDEN YVPLSLTPDL FTFHEDLCTT EEEKLQHRKQ EFGIPLLLSY
561   PMLINLIKYK PQITQEQLTS ITVAFTAMRE QCCKEENREA CFAKEVLVTL SPICS
```

5.5 Chain A, Crystal Structures Of Chicken Annexin V In Complex With Ca2+

Protein Accession	gi|62738641
Mean Expression Ratio	1.34
Median Expression Ratio	1.34
Credible Interval	(1.07, 1.67)
Associated Peptides	2
Associated Spectra	2
Coverage	0.0688

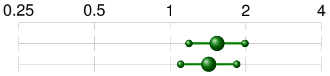

A	2.5	50	97.5	Sequence
1	1.2	1.5	2	ALLLLCGGDDE
1	1.1	1.4	1.8	GAGTDDDTLIR

```
1     AKYTRGTVTA FSPFDARADA EALRKAMKGM GTDEETILKI LTSRNNAQRQ EIASAFKTLF GRDLVDDLKS ELTGKFETLM
81    VSLMRPARIF DAHALKHAIK GAGTNEKVLT EILASRTPAE VQNIKQVYMQ EYEANLEDKI TGETSGHFQR LLVVLLQANR
161   DPDGRVDEAL VEKDAQVLFR AGELKWGTDE ETFITILGTR SVSHLRRVFD KYMTISGFQI EETIDRETSG DLEKLLLAVV
241   KCIRSVPAYF AETLYYSMKG AGTDDDTLIR VMVSRSEIDL LDIRHEFRKN FAKSLYQMIQ KDTSGDYRKA LLLLCGGDDE
```

5.6 fatty acid binding protein 5 [Gallus gallus]

Protein Accession	gi|57530631
Mean Expression Ratio	1.31
Median Expression Ratio	1.31
Credible Interval	(1.02, 1.71)
Associated Peptides	1
Associated Spectra	1
Coverage	0.104

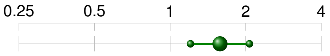

A	2.5	50	97.5	Sequence
1	1.2	1.6	2.1	DDGSLIQEQEWDGK

```
1     MAIDAFLGKW CLVSSEGFED YMKELGVGMA MRKMGSMAKP DVYIIKEGDT ITVKTESTFK TSQFSFKIGE KFEENTLDGR
81    KTQTLVSLKD DGSLIQEQEW DGKKTVITRK LVDGQLVVEC DMNGVKCVRV YQKA
```

5.7 PREDICTED: similar to OTTHUMP00000016587, partial [Gallus gallus]

Protein Accession	gi|118125386
Mean Expression Ratio	0.766
Median Expression Ratio	0.767
Credible Interval	(0.589, 0.993)
Associated Peptides	1
Associated Spectra	1
Coverage	0.0408

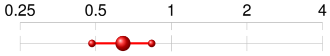

A	2.5	50	97.5	Sequence
**1**	**0.48**	**0.64**	**0.84**	**AAARSPREGR**

```
1      MRILTCPAHL QRLEAALRSS LPHALPVYGA VLNINRGNPG DFEVAVDAWP NFGAVLARRR GEAPVNDRYR NLCSAFYRDV
81     GAYRALVESP GCLRWDSAFH IFAPACRPPP GAARTRVGTE AEPRRPTARL PPARAPARPL AAPRSCCAGC HASAAVTRRP
161    VRTESRSVGT PRAPPLPPQG RRAAARSPRE GRGRSVPGPP YPPGVQDALV EVELAHEAGV GLHAGTAAPE VAQRPLQGPS
241    ELKEG
```

5.8 acetyl-Coenzyme A acetyltransferase 2 (acetoacetyl Coenzyme A thiolase) [Gallus gallus]

Protein Accession	gi|86129484
Mean Expression Ratio	0.767
Median Expression Ratio	0.768
Credible Interval	(0.59, 0.993)
Associated Peptides	1
Associated Spectra	1
Coverage	0.0599

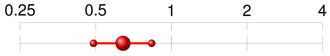

A	2.5	50	97.5	Sequence
**1**	**0.49**	**0.64**	**0.84**	**GEQDQLAVQSQNR**

```
1      MSADPIVFVA AVRTAIGSFN GALSALPAHE LGAAVIREAL RRAGLGPEEV SEVVLGQVLT AGAGQNPARQ ASVGAGIPYS
81     VPAWSCQMIC GSGLKAVCLG AQSIMTGDSS IVVAGGMESM SKAPHAIHMR AGVKMGEASL QDTIIHDGLT DAFYQYHMGI
161    TAENVANQWQ VSRGEQDQLA VQSQNRTEAA QKAGYFVKEI VPVLVPTKKG PIEVKTR
```

5.9 glutamic-oxaloacetic transaminase 1, soluble (aspartate aminotransferase 1) [Gallus gallus]

Protein Accession	gi|45384348
Mean Expression Ratio	1.29
Median Expression Ratio	1.29
Credible Interval	(1.04, 1.61)
Associated Peptides	2
Associated Spectra	2
Coverage	0.0947

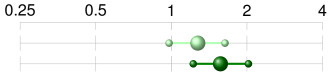

A	2.5	50	97.5	Sequence
**1**	**0.98**	**1.3**	**1.6**	**IALGDDSPAIAQK**
**1**	**1.2**	**1.6**	**2.0**	**KVEQLIAGDGSLNHEYLPILGLPEFR**

```
1      MAASIFAAVP RAPPVAVFKL TADFREDGDS RKVNLGVGAY RTDEGQPWVL PVVRKVEQLI AGDGSLNHEY LPILGLPEFR
81     ANASRIALGD DSPAIAQKRV GSVQGLGGTG ALRIGAEFLR RWYNGNNNTA TPVYVSSPTW ENHNSVFMDA GFKDIRTYRY
161    WDAAKRGLDL QGLLDDMEKA PEFSIFILHA CAHNPTGTDP TPDEWKQIAA VMKRRCLFPF FDSAYQGFAS GSLDKDAWAV
241    RYFVSEGFEL FCAQSFSKNF GLYNERVGNL SVVGKDEDNV QRVLSQMEKI VRTTWSNPPS QGARIVATTL TSPQLFAEWK
321    DNVKTMADRV LLMRSELRSR LESLGTPGTW NHITDQIGMF SFTGLNPKQV EYMIKEKHIY LMASGRINMC GLTTKNLDYV
401    AKSIHEAVTK IQ
```

5.10 malate dehydrogenase 1, NAD (soluble) [Gallus gallus]

Protein Accession	gi|57530355
Mean Expression Ratio	1.29
Median Expression Ratio	1.29
Credible Interval	(1.09, 1.53)
Associated Peptides	3
Associated Spectra	11
Coverage	0.114

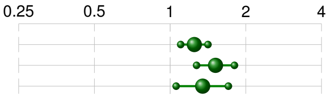

A	2.5	50	97.5	Sequence
7	1.1	1.2	1.4	FVEGLPINDFSR
3	1.3	1.5	1.8	DLDIAILVGSMPR
1	1.1	1.3	1.7	EVIPTDKEEVAFK

```
1     MGEPIRVLVT GAAGQIAYSL LYSIAKGDVF GKEQPLVLVL LDITPMMTVL EGVVMELQDC ALPLLREVIP TDKEEVAFKD
81    LDIAILVGSM PRREGMERKD LLKANVKIFK SQGAALDKYA KKTVKVVVVG NPANTNCLIA SKSAPSIPKE NFSCLTRLDH
161   NRAKSQIALK LGVTSNDVKN VIIWGNHSST QYPDVNHAKV NVKGKEVGVY EAIKDDSWLK GDFILTVQQR GAAVIKARKL
241   SSAMSAAKAI CDHVRDIWFG TPAGEFVSMG VISDGNSYGV PEDLLYSFPV VIKDKTWKFV EGLPINDFSR EKMDLTAKEL
321   TEEKETAVEF LSSA
```

5.11 PREDICTED: similar to MGC81256 protein [Gallus gallus]

Protein Accession	gi|118085057
Mean Expression Ratio	1.27
Median Expression Ratio	1.27
Credible Interval	(1.05, 1.56)
Associated Peptides	3
Associated Spectra	3
Coverage	0.0594

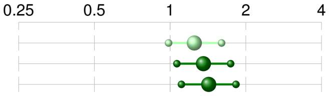

A	2.5	50	97.5	Sequence
1	0.99	1.3	1.6	EIVSVTVSK
1	1.1	1.4	1.7	GKPDTEVTEDEEYKR
1	1.1	1.4	1.8	KGKPDTEVTEDEEYKR

```
1     MAAVAMLGRR RAAAGLLRAL KYASRGYASQ RTLNEVVIAS AARTPIGSFQ GSLSSLPATK LGSIAIKGAI DRAGIPAEEV
81    KEAYMGNVLQ AGQGQAPARQ AVLGAGLPIS TDTTTVNKVC ASGMKSIMMA AQSLMCGSQD VMVAGGMESM SNVPYTMSRG
161   STPYGGVNLE DLIVKDGLTD VYNHIHMGNC AENTAKKFTI SREEQDTYAI GSYTKSKAAW DSGILKKEIV SVTVSKKGKP
241   DTEVTEDEEY KRVDFSKVPK LRAVFQKENG TVTAANASTL NDGAAALVLM TTEAAKRLKV KPLARIVAFA DAAVDPIDFP
321   IAPAHAVPKI LSETGLKKED IAMWEINEAF SVVVLANIKM LGIDPQKVNI NGGAVSLGHP IGMSGARIVV HMVHALKPGQ
401   YGLAGICNGG GGASAILIEK L
```

5.12 unnamed protein product [Gallus gallus]

Protein Accession	gi|63035
Mean Expression Ratio	0.791
Median Expression Ratio	0.79
Credible Interval	(0.699, 0.896)
Associated Peptides	6
Associated Spectra	24
Coverage	0.507

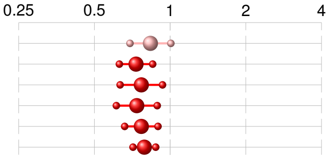

A	2.5	50	97.5	Sequence
2	0.69	0.83	1	IAGHAEEYGAETLER
4	0.63	0.73	0.85	VVAALIEAANHIDDIAGTLSK
2	0.63	0.77	0.93	VLSAADKNNVK
2	0.61	0.74	0.89	KVVAALIEAANHIDDIAGTLSK
4	0.66	0.77	0.9	TYFPHFDLSHGSAQIK
10	0.71	0.79	0.88	LSDLHAHK

```
1     MVLSAADKNN VKGIFTKIAG HAEEYGAETL ERMFTTYPPT KTYFPHFDLS HGSAQIKGHG KKVVAALIEA ANHIDDIAGT
81    LSKLSDLHAH KLTGGPVNFK LLGQCFLVVV AIHHPAALTP KVHASLDNFL CAVGTVLTAK YR
```

5.13 hypothetical protein [Gallus gallus]

Protein Accession	gi|53126140
Mean Expression Ratio	1.25
Median Expression Ratio	1.25
Credible Interval	(1.05, 1.50)
Associated Peptides	4
Associated Spectra	4
Coverage	0.0726

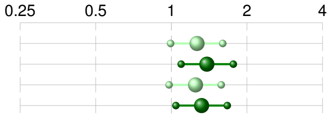

A	2.5	50	97.5	Sequence
1	1	1.3	1.6	FTITPGSEQIR
1	1.1	1.4	1.8	NALTQEFGPVPDTAR
1	0.98	1.2	1.6	FNPETDFLTGADGKK
1	1.0	1.3	1.7	IVYGHLDDPAKQEIER

```
1      MAPYCVLAAR LRHALNGGIR RYHVASVLCQ RAKVAMSHFE PNEYINYEKL EKNINIVRKR LDRPLTLSEK IVYGHLDDPA
81     KQEIERGKTY LRLRPDRVAM QDATAQMAML QFISSGLPKV AVPSTIHCDH LIEAQSGGEK DLRRAKDINQ EVYNFLSTAG
161    AKYGVGFWKP GSGIIHQIIL ENYSYPGVML IGTDSHTPNG GGLGGICIGV GGADAVDVMA GIPWELKCPK VIGVKLTGKL
241    SGWSSPKDVI LKVAGILTVK GGTGAIIEYH GPGVDSISCT GMATICNMGA EIGATTSVFP YNTRMKKYLG KTGRADIAAL
321    ADEFQQYLVP DAGCQYDQVI EINLSELKPH INGPFTPDLA HPVSDVGAVA EKEGWPVDIR VGLIGSCTNS SYEDMGRSAA
401    VAKQALAHGL KCKSKFTITP GSEQIRATIE RDGYAQILRD VGGLILANAC GPCIGQWDRK DIKKGEKNTI VTSYNRNFTG
481    RNDANPETHA FVTSPEIVTA LSIAGTLKFN PETDFLTGAD GKKFKLEAPD ADELPRLDFD PGQDTYQYPP KDGSGQHVDV
561    SPTSQRLQLL EPFDKWDGKD LEDMLILIKV KGKCTTDHIS AAGPWLKFRG HLDNISNNLL IGAINIENGK ANSVRNALTQ
641    EFGPVPDTAR YYKKMGVKWA VIGDENYGEG SSREHAALEP RHLGGRVIIT KSFARIHETN LKKQGLLPLT FADPADYNKI
721    HPVDKLSIVG LADFAPGKPL KCIIKHPNGS QETIMLNHTF NESQIEWFQA GSALNRMKEL QQKSS
```

5.14 PREDICTED: nuclear autoantigenic sperm protein (histone-binding) [Gallus gallus]

Protein Accession	gi|118094566
Mean Expression Ratio	0.798
Median Expression Ratio	0.798
Credible Interval	(0.664, 0.958)
Associated Peptides	4
Associated Spectra	4
Coverage	0.0777

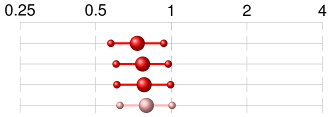

A	2.5	50	97.5	Sequence
1	0.58	0.73	0.93	AAVEEDEAAEAPAEK
1	0.6	0.77	0.97	ESAETAPEQKPDESK
1	0.61	0.78	1	EAQLHAAQAHLK
1	0.63	0.8	1	KPEEETHQGDNEAK

```
1      MEEDLAAPST SADRTDSMDV DAESKKLLGL GQKHLVMGNI PAAVNAFQEA ASLLGKKYGE TADECAEAFF YYGKSLLELA
81     RMENGVLGNA LEGVQVEEEG EKAEDDSALP AVDEEAREEL REQVYNAMGE KEEVKKSPVL TEKEIKGEDV EMKDVTEEKP
161    KEDTVTDMEV KLEGSEEKTE TSVEKVNSEE HGPQTAVEKN TEKAAVEEKA VEKEQKTKHE KAEATEGRNS MKEVLGEEAK
241    AAVEEDEAAE APAEKKGGAG EQTAEATETE TTVEKGESIE GQAEVAVEEK AMSQEGRAEG QAAVTAEQKE AAEEQAEAAE
321    KKVEEKESAE TAPEQKPDES KGMDTSKEPV SAVGEGPAND TGEKTEVAAK VEKEEKKDDL MEESEGAKVE KEEKDDQMEE
401    GEETEESEEE DKENDKAEDD KENELAVEDK ESEEEEIGNL ELAWDMLELA KVIYKRQETK EAQLHAAQAH LKLGEVSIES
481    ENYVQAIEEF QACLALQQKY LEAHDRLLAE SHYQLALAYH YNSQFDEAVL QFGKSVEVID KRLAMLTERI KNVESGSPED
561    EKEIEELKGL LPEIKEKIED SKESQKSARV AELALKATLV GTTSGFAQSE GSGSVSTIPV RKAADGASQC VTDISHLVRK
641    KRKPEEETHQ GDNEAKKSKP EPAVNGGGGD AAPSGNEVAE KMEEERKGHK QNQGLQLKAQ YDNSLALDTP LLQRKMVLFC
721    P
```

5.15 apolipoprotein B (including Ag(x) antigen) [Gallus gallus]

Protein Accession	gi|113206052
Mean Expression Ratio	0.8
Median Expression Ratio	0.8
Credible Interval	(0.661, 0.967)
Associated Peptides	3
Associated Spectra	4
Coverage	0.00799

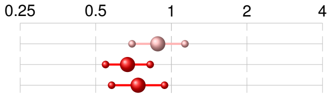

A	2.5	50	97.5	Sequence
1	0.7	0.88	1.1	EILNMAQYQPSR
2	0.55	0.67	0.82	TEEIPPLIENR
1	0.58	0.74	0.94	NSFLINIPLPFGGR

1	MGPVRLLLLL	LLLSSGVLTQ	EGTPENGNPG	CSKDAARFKS	LRKYVVLYEA	ETSSGITGTA	DSRSGSKITC	KVELEVPQLC
81	QFILRTMHCS	LRETFGVDSE	RRAMLRKSKN	SDDFANAMSK	HELRFSTQDG	TKVKLYPEKD	EPLNVLNLKR	GIISALLAPT
161	ETEENIKTIS	MDTVYGKCDS	EVEFKSRRGS	VAEDISINRN	LKACDNFSPI	RDYVSPVAIV	KGLNIPLSTL	LSSTQSCHYS
241	IDAKKKHIRD	VVCSEKHLFL	PSSYKNQYGM	MTEVNQTLKL	EDNQRMNNRN	PDGDELEEKG	LALESTDAKF	SRQGDAVLKI
321	LQELQKLTAS	QQNQQRAKLF	YKFVSGLRSL	HNSTLGSLVP	KMMETSSSIT	IQALIQCGTP	ECYSAVLQIL	RTGNVNPLVV
401	DLVTYTLGLL	PSPTPKRIRE	ILNMAQYQPS	RASFYGLSHA	VTKFYNEKMI	VTEEITDVAD	FMVSLLGTDC	SGDAELTYLT
481	LRAIGNMGAV	MEKAKPSLKA	SLKTCIRNQA	ASLSVQKAAI	QAFRKMTITE	EDRSALLKEF	QEGDAPTDKR	LATYLILMKN
561	PSPADLAKIM	RILTREKNEQ	VKSFVASHIA	NILDSDEVGI	EDLKSHVEEA	LKGNEVPTAK	DFRKFSQNYQ	VSKRVSVPGL
641	NPISAKVEGN	VIFDPSSYVP	KETMLKTTLN	LYGFGPSDIF	ELGLDGKGFE	PTLEALFGEK	GFFPDTASKA	LYWVDGKVPE
721	QVSKALFDYF	GYSHDGKQDQ	EITKAVILNL	EKLIKELSKK	EAPEGRAFLR	ILGEELGYMK	LSDFKLLGSV	ALECIKTLQR
801	IPEIIAQAIS	KGVDKDLFVH	YMFMDNEFEL	PTGAGLQLKF	ALSGIVTPGA	KVAVKLHQKS	MQAELIAKPS	VAVEFVTHLG
881	INMPEFARSG	VEMNSNIFHE	SGIEAHVSVK	AGQLKFSIPA	PKTPTKLLSI	SNTLHLVSPA	KTEEIPPLIE	NREFSTSCKP
961	FISGLNFCTK	LLYSNASSME	AAPYYPLTGE	TRFEVEIVST	GEVKEYSASA	NYDLQREGTD	LVDTLKFAVQ	AEGVKQHEAT
1041	LTFKYNRDRK	ILTSDVSIPD	VDVDFGTNFR	ITDESVSGKK	AYIFVIDFNN	KKISEVTLTG	QIRYAGIEEA	MLRGTVSIPR
1121	LQTELKTEAL	VNYSPTKGYL	QMSSSVGTHG	NTVSKRVLLR	YDSEKAELEW	NSGATAAVGR	MSSAFQVDFS	DYSKTLEKYA
1201	NELLDRKVAL	TDMTMRHIVS	QFIVATNTWL	QKASKDVPYA	QTLQAKLSGL	QELNIQKIKL	PVITIPEELF	LKSEGRIKYS
1281	FNKNSFLINI	PLPFGGRSSH	DIRVPQTVKT	PRLVIESMGI	NIPSQEYRMP	TFTVPESYPL	LVPLFGALEA	SASVHSNYYN
1361	WTAAYTLTNS	STEKTARIGT	TYAVNADSVF	ELLSYNMKGS	GEASSSRNGF	TCAYENHLKH	RLLTSDFKMS	RTKSYEPTSV
1441	SNCTIFLMAS	SALGPQLSFS	SDVVSEKTNN	MNINNVRIEG	QLEVASVFAR	SVYTMSSSYN	EKRRVLEGKS	NLRLDSSYLQ
1521	ATNHLSGRYT	DGVFSITSAS	DVQNGLLKNT	ASLKYENSQL	KITSETNGRY	LHLAAVNKLE	FLLSKKMAAL	RSEYQATYKQ
1601	TQCYALFAGS	LNSQDLVFNT	DFSLTDQRNR	AAHKSSLNVN	QYGLASSATT	NVQFSPLTMQ	SEMNAKLDTS	GGSVSLSSSG
1681	RYGKNNAKFN	VGGRVSLTEI	TLGSEYQSTI	LGMDNKHVLN	FRINKEGLKF	SNNLQGSLKE	IKLEYTNDLN	IPGLSLTFVS
1761	KLDNSFSFDK	FHKHVFDLQL	QPRSLTAKLN	NNIKYTKTEV	SNKAELLLEP	LKLNLGGNVR	AAYGTDEVRH	TYAITYADLT
1841	ANFKTDTVAN	VQGAAVSHRV	NLNVAGLASS	ITMNTNCDSK	SLRFSNALRS	TMAPFTITAD	VHTNGNGKLI	ALGEHTGDLY
1921	SKILFKAEPL	AFTFSHDYRG	STSHSFKSMR	RYSTQLDNKF	HMLFTPSEQS	SAWKLKSQLN	NNIYSQDINA	YNDAEKIGVE
2001	LSGRALADLS	VVDTAIRLPF	MSEEVNVIDV	LGLRDSVSEP	QEFSISGSVK	YDKNKDMHVI	NLPFLEHFPV	YFEQIRGAIL
2081	STLQAVQNYL	KNIDVDQYMK	KYKATLDEFP	QHLNDYMDKL	DLKGRASTIK	TNLIAFTKDY	RITSDDLEII	LEKALDNLQE
2161	ILLQLQVYLV	QIEQYIKENY	DQFDINALIA	QLLDKIVEKM	TALDEKYKIR	VTVVDTIQKL	QFFLNQYYPS	NIGSNTMTWI
2241	KNIDDEYRIT	GRIKENLEQL	KIQIQNIDIR	SFAENLKRKI	KIIDVKQLLE	KLKRSLPIKK	MKEVLEQIKD	FILSWMEEYE
2321	VSEKISAFRG	HMHKLIVKYE	IDKHVYFLLD	RMIELLNQYR	IRETVRKMTT	YLRKIDVKTC	FDKIVSLIDD	AVKKVQTFDY
2401	EMMIKKLNKF	LDMIIKKLKS	FDYNQFVDDT	NNKIQEIIQK	INEELRNLEL	PQKAEALKQY	MRDFNAVVSK	YVEQLRDTKL
2481	VAIINWLKEL	IDSTTFTNLK	AKVNEHLEGL	RERISDMDIA	KEFEWYLQKI	SQFYNSVVIY	ISEQWNIAFK	KIVTLAEKYD
2561	LKNWAENLNQ	FIETGFKVPE	IRTVIVTIPA	FEFSLRSLRE	ATFRTPDFIV	PLTDLKIPSY	EINIRRLKDM	KIPAKFTTPE
2641	FTVLNSFKVP	SYTIDLNEIK	FQIVRMIDQL	ISGEFQLPAI	DLYFKDLKMR	DMPFSEISFP	ELQMPQLEIP	ELLIPKLNLN
2721	EFQIPDLKIP	EFQLPRIPHT	VTAPTFGKLS	GAFRVASPFF	TLSTQAEVHN	TTASANSPEF	VTSLSAQATS	KLDFLVFSVI
2801	ADSHILAPEM	KQLKLKNSMK	VSHKFLKIDH	TNEVVFLGTS	VSGEAETRAK	FSAIKNSIEL	QNNLMVNLQR	KIQMQSGTAY
2881	SHRLNIPEAD	FSSQADLVNN	MTTEVEAGRI	SFTSNGKGNW	KWTSPNFSDE	GTHNSHATFR	VDGPILIFFA	DYRINDRYLK
2961	VSQSMRYECG	FLSYATLQVQ	SEIESQRVGR	SILNVKGTGQ	LGGMKVELTG	SHNARLNGRI	TGTVNNDFFF	LVQPFEIRLL
3041	TNNDGNVKIS	FPMKLTGKID	FLNNYGLSLS	SSVQQVSWQA	TGRFNQYRYS	HNMSAGNNDD	RIEAHVEMSG	DANLDFLNIP
3121	LTIPQLHIPY	TGIQTPQLKD	YSLWEQAGLK	NLLKTTRQSF	DLNLNAQYEK	NKDMHVIPLP	LATVHEALNK	YIIFFNKYFE
3201	RGRNTALDFL	TKSYNEAKTK	FDKYKIQTSL	NKLPRTFRIP	GYTIPIVNIE	VSPFTAEMPA	FGYVLPKEIS	TTGFTVPFIG
3281	FSVPSYTLVL	PSLELPVLHV	PQDLRTLKLP	RFRINSPSNQ	ILIPAMGNIT	YDFSFKSSVI	TLTANAGLFN	QSDIAGHLSI
3361	SSSSVIDALQ	FKLDGSTSLT	RKRGLKLATA	LSLNNNKFLG	GSHDNSISLT	KKNLEASMIT	NAKINTPVFK	MNFSQELSGN
3441	TKSKPTISSG	LKVTYDFTTP	KHGISAKGGV	AHKLALETLT	SYLSVETSTK	GNIDGAIYTG	NSFSGALDHE	ANTYLHANGV
3521	RSSLKLEANS	KVDGLWNSEM	KEILAVEAST	SRVYAVWEHN	GKNFARYTPL	FTTTGSQKCK	ATFELAPWTV	SADLQIQVTQ
3601	PNSFLDTASV	NQVVLMKVSP	TDQKVGWKGE	GQIQSLSLRH	DMQLSNEKSN	AKFDISGSLE	GYMDFLKRIN	CAISKKSLWD
3681	ILKLDVTTVA	DRKHYLNASA	SFIYRKSDDG	YFFPMPVIRL	SDGFTFSIPE	LHLKAPSPVL	STPEFRVPFS	TLQVPAYTID
3761	LRNIKIPQTL	NIMPFDVNLP	TLPKLRFPRV	DVGANYITLE	EYKIPYFEVT	VPEYQITVSQ	FTLPKSISLG	SFHVDLDEVA
3841	NKIADFDLPT	ITIPEQKIEI	PPLKVSLPAG	IYIPSFGALT	GSLKVASPLY	NVTWRTDLTN	KKESFEVSID	STCSSTLQFL
3921	EYDLNVVSNY	KYEEGKFVKK	TVGSFAHRDL	SANYIEDLAL	QGFGTVENTA	SLDVISPTFA	DVHVRYQMTD	NMISSTVSSP
4001	SAGILGFLVQ	IETDTLKEKF	YYRTLSAPQK	DIDILKSEIS	FRNSDIIQIK	LNWKEDAAKD	LLLGLKEKVP	KMTSAVYKCV
4081	NRYYKEHMGL	DISDATVIMK	NILQNNADKA	YMFAARQVDQ	MDVQLRTAAN	EASEKYQEMK	VKARQLYKRA	AEQAEQIDYQ
4161	RIKARLLDAT	VDLMEEYHSK	IRHLIDSVIE	FLKTTKFQIP	GLSEKYTGEE	LYLMTTEKAA	KTADICLSKL	QEYFDALIAA
4241	ISELEVRVPA	SETILRGRNV	LDQIKEMLKH	LQEKIRQTFV	TLQEADFAGK	LNQLKQVVQK	TFQKAGNMVR	SLQSKNFEDI
4321	KVQMQQLYKD	AMASDYAHKL	RSLAENVKKY	ISQMKNFSQK	TLQKVSENLQ	QLVLYIKALR	EEYFDPTTLG	WSVKYYEVED
4401	KVLGLLKNLM	DTLVIWYNEY	AKDLSDLVTR	LTDQVRELVE	NYRQEYYDLI	TDVEGKGRQK	VMELSSAAQE	KIRYWSAVAK
4481	RKINEHNRQV	KAKLQEIYGQ	LSDSQEKLIN	VAKMLIDLTV	EKYSTFMKYI	FELLRWFEQA	TADSIKPYIA	VREGELRIDV
4561	PFDWEYINQM	PQKSREALRN	KVELTRALIQ	QGVEQGTRKW	EEMQAFIDEQ	LATEQLSFQQ	IVENIQKRMK	T

5.16 RNA binding motif protein 6 [Gallus gallus]

Protein Accession	gi|71895461
Mean Expression Ratio	0.802
Median Expression Ratio	0.802
Credible Interval	(0.619, 1.04)
Associated Peptides	1
Associated Spectra	1
Coverage	0.0146

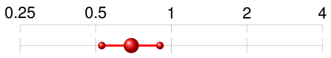

A	2.5	50	97.5	Sequence
1	0.53	0.69	0.9	EGEEATPQEGGGSR

```
1      MWGDHRQGNR TGSFRGSQEE RFAPGWNRDY PPSLDSLAQE RHSGNFSGRE SLPFDIQALA GLLNPQFANR EEPSFSFGAR
81     DGPRSDFRGG EGHHDFRGRD FPPSDFQSRD ESQLDFRGRE PPPSEYRGRD MYSGDFRERE GPPMDFRGGD IPPVDYRNRE
161    TFRMNYRDRE AHSMDYRGRD EPPSDFRGRG SFDLDFRGRD GSHSDFRARD VPELDYRGRD HSYSDFRNRD VPDLDFRGVG
241    TSDLDFRNRN APSSDFRNRH RSRSDQDFRS RDMPPPMDFS DREMPPVDQS LVDFRRNQST IPLAEREGSG LNTGKREEPA
321    LGLERTPFGS QKGEFQHSEA PAREEESLGL GLKDNASHNF QNSRGPLPAL QDQEEQPQTF ANKQQQQQQL SGGEQQRSDS
401    DLGLKGEDDL DFLGRQDTDY RNIEYRDVDH RLPACQLFDY EHGKSFSEGK PSKDSRPYKS LQDQDYRTCP EFVKPSKLIR
481    LGGVPETATK DDILNAFRGP DGMPVKNLRL KDYSSGYDYR YVCVEFSLLE EAIGCMEANQ GTLTIGGEEV TLEYTLCPEF
561    WRCKRCKVCN VGYRSSCSYC KLPRDGNRAG SESKGGPEGE DASSEQEFTE TKLEKPEQEL PVQPGSPKQP LQASDPAPQQ
641    ECQPQEQEPR KRDEGRERRP SQEKRRDTDR HQELPSQREG EEATPQEGGG SRTIMLKRIT RFTPPEVIVG LLAPYVRIST
721    SNVRIMKNKA GRMGQTYGFI ELDSHAEALR LLKILQNLDP PICIDGRTLE VNLATGRRRN DYGEHGDNSY YNQGRRGMRD
801    RRGGESQRRT RAQSPSDVST YIYDPETGNY YDPIAGTYYD PRTQREVTID REPCSSPPEN RRRRHESQER TSERKEPHSR
881    DNRDKKEKGK NTPAKNEPGE ERFFTEDVFK KPLPPSVKKD ETAGLNDCGE EKFFAEDVFK KPLPPSVKKD ESAARPRW
```

5.17 kinase related protein

Protein Accession	gi|211372
Mean Expression Ratio	0.803
Median Expression Ratio	0.803
Credible Interval	(0.616, 1.04)
Associated Peptides	1
Associated Spectra	1
Coverage	0.108

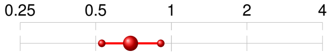

A	2.5	50	97.5	Sequence
1	0.53	0.69	0.9	EGEGEGEGEEDEEEEEE

```
1      MAMISGMSGR KASGSSPTSP INADKVENED AFLEEVAEEK PHVKPYFTKT ILDMEVVEGS AARFDCKIEG YPDPEVMWYK
81     DDQPVKESRH FQIDYDEEGN CSLTISEVCG DDDAKYTCKA VNSLGEATCT AELLVETMGK EGEGEGEGEE DEEEEEE
```

5.18 alpha-D globin [Gallus gallus]

Protein Accession	gi|63013
Mean Expression Ratio	0.815
Median Expression Ratio	0.814
Credible Interval	(0.694, 0.958)
Associated Peptides	4
Associated Spectra	8
Coverage	0.397

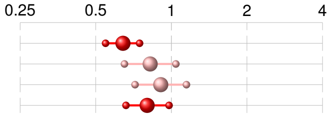

A	2.5	50	97.5	Sequence
4	0.55	0.64	0.75	TYFPHFDLSPGSDQVR
1	0.65	0.82	1.0	NVDNLSQAMAELSNLHAYNLR
1	0.72	0.9	1.1	MLTAEDKK
2	0.66	0.8	0.98	KVLGALGNAVK

```
1      MLTAEDKKLI QQAWERAASH QEEFGAEALT RMFTTYPQTK TYFPHFDLSP GSDQVRGHGK KVLGALGNAV KNVDNLSQAM
81     AELSNLHAYN LRVDPVNFKL LSQCIQVVLA VHMGKDYTPE VHAAFDKFLS AVSAVLAEKY R
```

5.19 albumin [Gallus gallus]

Protein Accession	gi|45383974
Mean Expression Ratio	1.22
Median Expression Ratio	1.22
Credible Interval	(1.07, 1.38)
Associated Peptides	9
Associated Spectra	12
Coverage	0.176

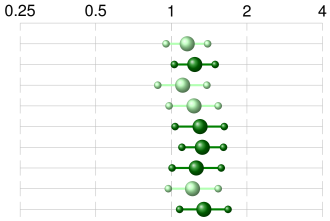

A	2.5	50	97.5	Sequence
2	0.96	1.2	1.4	QSDINTCFGEEGANLIVQSR
2	1.0	1.2	1.5	AVAMITFAQYLQR
1	0.88	1.1	1.4	LLINLIK
1	0.98	1.2	1.5	KPQMTEEQIK
1	1.0	1.3	1.6	YNDLKEETFK
2	1.1	1.3	1.6	ADPERNECFLSFK
1	1	1.3	1.6	RHPEFSIQLIMR
1	0.97	1.2	1.5	VSFLGHFIYSVAR
1	1.1	1.3	1.7	FVHDSIGVHK

```
1     MKWVTLISFI FLFSSATSRN LQRFARDAEH KSEIAHRYND LKEETFKAVA MITFAQYLQR CSYEGLSKLV KDVVDLAQKC
81    VANEDAPECS KPLPSIILDE ICQVEKLRDS YGAMADCCSK ADPERNECFL SFKVSQPDFV QPYQRPASDV ICQEYQDNRV
161   SFLGHFIYSV ARRHPFLYAP AILSFAVDFE HALQSCCKES DVGACLDTKE IVMREKAKGV SVKQQYFCGI LKQFGDRVFQ
241   ARQLIYLSQK YPKAPFSEVS KFVHDSIGVH KECCEGDMVE CMDDMARMMS NLCSQQDVFS GKIKDCCEKP IVERSQCIME
321   AEFDEKPADL PSLVEKYIED KEVCKSFEAG HDAFMAEFVY EYSRRHPEFS IQLIMRIAKG YESLLEKCCK TDNPAECYAN
401   AQEQLNQHIK ETQDVVKTNC DLLHDHGEAD FLKSILIRYT KKMPQVPTDL LLETGKKMTT IGTKCCQLGE DRRMACSEGY
481   LSIVIHDTCR KQETTPINDN VSQCCSQLYA NRRPCFTAMG VDTKYVPPPF NPDMFSFDEK LCSAPAEERE VGQMKLLINL
561   IKRKPQMTEE QIKTIADGFT AMVDKCCKQS DINTCFGEEG ANLIVQSRAT LGIGA
```

5.20 PREDICTED: similar to RAD23B protein [Gallus gallus]

Protein Accession	gi|118104328
Mean Expression Ratio	0.822
Median Expression Ratio	0.823
Credible Interval	(0.634, 1.06)
Associated Peptides	1
Associated Spectra	1
Coverage	0.0446

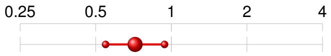

A	2.5	50	97.5	Sequence
1	0.55	0.72	0.94	QIIQQNPSLLPALLQQIGR

```
1     MASMGARAEK DHGGVAEMEH YGVTADPTLC SSAQLGGRDR RGYGWVKALK EKIESERGKD AFPVAGQKLI YAGKILNDDT
81    ALKEYKIDEK NFVVVMVTKP KAATAATTAT QQTNSTTGVS VTTAAPTPVS APTPAAAPVP APDPTTPAPA AVACESEPVS
161   TPKEEKPAEK PADAPTAVSL SSNESTTGDT SRSNLFEDAI SALVTGQSYE NMVTEIMSMG YEREQVIAAL RASFNNPDRT
241   VEYLLMGIPG DNQAVAEPTQ AASTGTSQSS AVAAAVATIP TTATSLGGHP LEFLRNQPQF QQMRQIIQQN PSLLPALLQQ
321   IGRENPQLLQ QISQHQEHFI HMLNEPVIES RQGLSGSDDS ASTGGVAEAG NGHMSYIQVT PQEKEAIERL KALGFPEGLV
401   IQAYFACEKN ENLAANFLLQ QNFDED
```

5.21 splicing factor, arginine/serine-rich 10 [Gallus gallus]

Protein Accession	gi|45382747
Mean Expression Ratio	1.21
Median Expression Ratio	1.21
Credible Interval	(0.97, 1.50)
Associated Peptides	2
Associated Spectra	2
Coverage	0.0657

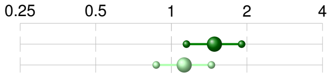

A	2.5	50	97.5	Sequence
1	1.2	1.5	1.9	GYDRGYDDR
1	0.87	1.1	1.4	AVQDRDQFYR

```
1     MSDSGEQNYG ERESRSASRS GSAHGSGKSG RHTPARSRSK EDSRRSRSKS RSRSESRSRS RRSSRRHYTR SRSRSRSHRR
81    SRSRSYSRDY RRRHSHSHSP MSTRRRHIGN RANPDPNCCL GVFGLSLYTT ERDLREVFSK YGPIADVSIV YDQQSRRSRG
161   FAFVYFENVE DAKEAKERAN GMELDGRRIR VDFSITKRPH TPTPGIYMGR PTYGSSRRRD YYDRGYDRGY DDRDYYSRSY
241   RGGGGGGGGG WRAVQDRDQF YRRRSPSPYY SRGGYRSRSR SRSYSPRRY
```

5.22 PREDICTED: hypothetical protein [Gallus gallus]

Protein Accession	gi|50758382
Mean Expression Ratio	0.827
Median Expression Ratio	0.829
Credible Interval	(0.641, 1.07)
Associated Peptides	1
Associated Spectra	1
Coverage	0.0241

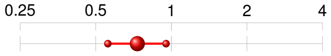

A	2.5	50	97.5	Sequence
1	0.56	0.73	0.95	GSAVPSCWRAVK

```
1     MSQKHLKEAF VSNLNGTSLL ELSLGMPLAP LCLLCRGLLL VLHCQHRGKP LRSRLPALLL DFGVLVAPLL FSCTVLSPVL
81    FLVPAILAAF CAGIFFTLYR QRQRDSRVHF RQIVKDFQNT YLDPDYVPAI TVFRVYVNVL TSISILAVDF PQFPRRYAKA
161   ETYGTGVMDL GVGAFIFSNA LVCPEVRQKA AVTQPKFSSL TKQLFAIWPL VVLGIGRFLS VKSIEYHEHI SEYGVHWNFF
241   FTLALVRIAA SLLLAIFPKN KSWIVAVTVA VLYQLILNLT SLKTFVLHGS DGEDSRTGFL NANREGLLSL FGYLAIYMAS
321   VQVGLYLLKC RDSVKEWMGA VCLLLLAALV LFVFLHISQA HTDPVSRRMA NLSYCLWVIA YCLTLFVSFV VADLMLVFTK
401   LLVKGSAVPS CWRAVKAPDS REQRQAEAVH ICLIAAINKN QLLFFLLANV MTGIVNVMID TIHSRAALTL CILHLYMFLN
481   CFIMYILHAK NIVLKFW
```

5.23 PREDICTED: hypothetical protein, partial [Gallus gallus]

Protein Accession	gi|118128960
Mean Expression Ratio	1.20
Median Expression Ratio	1.20
Credible Interval	(0.933, 1.56)
Associated Peptides	1
Associated Spectra	1
Coverage	0.0498

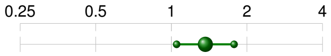

A	2.5	50	97.5	Sequence
1	1.0	1.4	1.8	GAVQGSVELTK

```
1     MRTLLYGPHG RAHPEVRGRS HVSSVDPRQS VVVRVANLPL VSSACSTVSS AYNCTKGSHP YVRSVCDAAE KGVRTLTAAA
81    VSGAQPILTK LEPQISTANE YACKGLDKLE EKLPILQQPT EKLISDTKQL VTSTVTGARD VLTSTVAGAK DAVSSRVTGV
161   MDMTKGAVQG SVELTKSAVS SGVTTVMGST VGQMVASGVG SMLEKSEELV DHYLPMTDEE L
```

5.24 fumarate hydratase [Gallus gallus]

Protein Accession	gi|57530433
Mean Expression Ratio	1.2
Median Expression Ratio	1.2
Credible Interval	(0.929, 1.55)
Associated Peptides	1
Associated Spectra	1
Coverage	0.0355

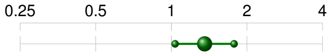

A	2.5	50	97.5	Sequence
1	1.0	1.4	1.8	VYQLAAGGTAVGTGLNTR

```
1     MHRSLRAFRR LGRSAGLLAS PPSPGTAAAN AARWAPLRAA MSTQESFRIE YDTFGELKVP SDKYYGAQTV RSTMNFKIGG
81    VSERMPVQVI RAFGILKRAA AEVNQDYGLD PKIANAIVKA ADEVAAGKLN DHFPLVVWQT GSGTQTNMNV NEVISNRAIE
161   ILGGKLGSKI PVHPNDHVNK SQSSNDTFPT AMHIAAAQEV NEVLLPGLKK LQNALEAKSK EFSQIIKIGR THTQDAVPLT
241   LGQEFSGYVQ QIKYGVARIE STMPRVYQLA AGGTAVGTGL NTRIGFAEKV AAKVAELTGL PFVTAPNKFE ALAAHDALVE
321   LSGAMNTVAC SLMKIANDIR FLGSGPRSGL GELILPENEP GSSIMPGKVN PTQCEAVTMV AAQVMGNHVA VTVGGSNGHF
401   ELNVFKPMMI KNVLNSARLL GDVCVSFTDN CVVGIQANTD RINKLMSESL MLVTALNPHI GYDKAAKIAK TAHKEGTTLK
481   EAAIKLGFLT SEQFDQWVKP KDMLGPQ
```

5.25 proteasome (prosome, macropain) 26S subunit, ATPase, 2 [Gallus gallus]

Protein Accession	gi|57525333
Mean Expression Ratio	1.19
Median Expression Ratio	1.19
Credible Interval	(0.918, 1.54)
Associated Peptides	1
Associated Spectra	1
Coverage	0.03

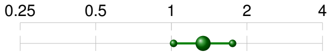

A	2.5	50	97.5	Sequence
1	1.0	1.3	1.8	FDDGAGGDNEVQR

```
1     MPDYLGADQR KTKEEEKEDK PIRALDEGDI ALLKTYGQST YSRQIKQVED DIQQLLKKIN ELTGIKESDT GLAPPALWDL
81    AADKQTLQSE QPLQVARCTK IINADSEDPK YIINVKQFAK FVVDLSDQVA PTDIEEGMRV GVDRNKYQIH IPLPPKIDPT
161   VTMMQVEEKP DVTYSDVGGC KEQIEKLREV VETPLLHPER FVNLGIEPPK GVLLFGPPGT GKTLCARAVA NRTDACFIRV
241   IGSELVQKYV GEGARMVREL FEMARTKKAC LIFFDEIDAI GGARFDDGAG GDNEVQRTML ELINQLDGFD PRGNIKVLMA
321   TNRPDTLDPA LMRPGRLDRK IEFSLPDLEG RTHIFKIHAR SMSVERDIRF ELLARLCPNS TGAEIRSVCT EAGMFAIRAR
401   RKIATEKDFL EAVNKVIKSY AKFSATPRYM TYN
```

5.26 Chain B, R-State Form Of Chicken Hemoglobin D

Protein Accession gi|4699641
Mean Expression Ratio 0.843
Median Expression Ratio 0.843
Credible Interval (0.733, 0.967)
Associated Peptides 7
Associated Spectra 9
Coverage 0.589

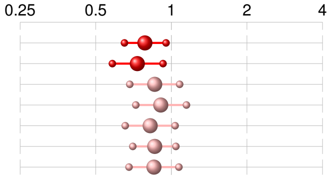

A	2.5	50	97.5	Sequence
2	0.65	0.79	0.95	LLIVYPWTQR
1	0.58	0.73	0.92	FFASFGNLSSPTAILGNPMVR
1	0.68	0.86	1.1	VNVAECGAEALAR
1	0.72	0.91	1.1	VLTSFGDAVK
1	0.65	0.82	1.0	QLITGLWGK
2	0.7	0.86	1.0	KVLTSFGDAVK
1	0.68	0.85	1.1	NTFSQLSELHCDKLHVDPENFR

```
1       VHWTAEEKQL ITGLWGKVNV AECGAEALAR LLIVYPWTQR FFASFGNLSS PTAILGNPMV RAHGKKVLTS FGDAVKNLDN
81      IKNTFSQLSE LHCDKLHVDP ENFRLLGDIL IIVLAAHFSK DFTPECQAAW QKLVRVVAHA LARKYH
```

5.27 potassium channel tetramerisation domain containing 9 [Gallus gallus]

Protein Accession gi|61098191
Mean Expression Ratio 0.843
Median Expression Ratio 0.843
Credible Interval (0.653, 1.10)
Associated Peptides 1
Associated Spectra 1
Coverage 0.0257

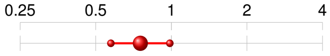

A	2.5	50	97.5	Sequence
1	0.58	0.75	0.98	VTLFVNGSPR

```
1       MRRVTLFVNG SPRNGKVVAV YGTLSDLLSV ASNKLGIKAT SVYNGKGGLI DDIALIRDDD VLFVCEGEPF IDPQTDARPH
81      EELTGAHTDW LTLNVGGRYF TTTRSTLVNK EPDSMLAHMF KDKDAWGNKQ DHRGAFLIDR SPEYFEPILN YLRHGQLIVN
161     DGINLLGVLE EARFFGIDSL IEHLEIAIKN SQPAEDHSPI SRKEFVRFLL ATPTKSELRC QGLNFSGADL SRLDLRYINF
241     KMANLSRCNL AHANLCCANL ERADLSGSVL DCANLQGVKM LCSNAEGASL KGCNFEDPSG IKANLEGANL KGVDMEGSQM
321     TGINLRVATL KNAKLKNCNL RGATLAGTDL ENCDLSGCDL QEANLRGSNV KGAIFEEMLT PLHMSQSVR
```

5.28 PREDICTED: similar to alpha-NAC, muscle-specific form gp220 [Gallus gallus]

Protein Accession gi|118129698
Mean Expression Ratio 0.843
Median Expression Ratio 0.843
Credible Interval (0.7, 1.01)
Associated Peptides 3
Associated Spectra 4
Coverage 0.0642

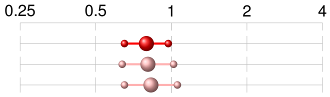

A	2.5	50	97.5	Sequence
2	0.65	0.8	0.97	SPTAAPSAPPAPAAPASSAAAK
1	0.64	0.81	1.0	AAPQSPVAATPAPAAAPATAAPAATK
1	0.65	0.83	1.1	AAPQSPVAATPAPPAAPAATK

```
1       MEATTSLLDA AAVGDLVMLD PLSEESLLRT LQERFSRGEI YVGTGMRGWG GEWGLGGGSP PAVPVHPAAP LPPPAAPLGP
81      ALAAAISPPI FLSAPMALAP LLPGGTVPCS AAASPRGPMS ATPVCPLAPA APSGAVTCPV GPALAASGTP AVPHSVARPP
161     PGSPIPVTAP VLPSPSPAAA LSPSGPPPAA AAPLKAAPVI PVSLPAAVTA APASSLPVTP AMAAPATPPA AKGAPQSPVP
241     APLSPSAPAA AAPVVPPAAP AATKAPPQSP VTTPSAPAAV VPAAAPAPAA NKAAPKSPVA ATPAPPAAPA AKKAAPQSPV
321     AAPSAPAAVV PAGPTNPAAP AATKAAPQSP VAATPAAPAA TKAAPQSPVA ATPAPPASPA ATKPAPESPI AATPAPAAAP
401     PATKAGPQSP IAATPAAPAA TKAAPQSPVA ATPAPATAPA TAAPAATKAA PQTAPAATKA APQSPVAATP APAFAPATAA
481     PAATKAAPQS PIAATPAAPA ATKAAPQSPV AATPAPPAAP AATKAAPQSP IAATPAAPAA AKAAPQSPVA ATPAPPAAPS
561     AAKAAPQSPV AATPAPAAAP ATAAPAATKA APQSPVAATP AAPAATKAAP QSPIAATPAP PAAPPATKAA PQSPIAATPA
641     APAATKAAPQ SPVAATPAPP AAPAATKAAP RTPASSAAAK APPKSPTAAP SAPPAPAAPA SSAAAKAPPK SPTAALAAPA
721     APSAPSAAKA APVSSAAPGT LPPAPGAAVS APGAPVALPK PAADRAAPTP QKTEATPPAS APGGNITPPA SSMPNAAPAK
801     KQPPTNKGSK QAPRPSPTKP AVKAPVPASA AVDDDDDLPP LIPPEVPAAE PPLQPILVDL SPRAAVAPAE APAPKQPVVK
881     NDKGSGTESD SDESVPELEE HDSTQATTQQ AQLAAAAEID EEPVSKAKQS RSEKKARKAM SKLGLRQVTG VTRVTIRKSK
961     NILFVITKPD VYKSPASDTY IVFGEAKIED LSQQAQLAAA EKFKVQGEAV SNIQENTQTP TVQEESEEEE VDETGVEVKD
1041    IELVMSQANV SRAKAVRALK NNSNDIVNAI MEMTM
```

5.29 activated RNA polymerase II transcription cofactor 4 [Gallus gallus]

Protein Accession	gi|56605972
Mean Expression Ratio	0.845
Median Expression Ratio	0.845
Credible Interval	(0.676, 1.05)
Associated Peptides	2
Associated Spectra	2
Coverage	0.167

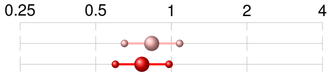

A	2.5	50	97.5	Sequence
1	0.65	0.83	1.1	EQISDIDDAVR
1	0.6	0.76	0.98	QAAPEKPVKK

```
1    MPKSKELVSS SSSASDSDSE VDKKAKRKKQ AAPEKPVKKQ KTGESSKGAA SSKQSSNRDE NMFQIGKMRY VSVRDFKGKV
81   LIDIREYWMD QEGEMKPGRK GISLNPEQWN QLKEQISDID DAVRKL
```

5.30 glucose phosphate isomerase [Gallus gallus]

Protein Accession	gi|57524920
Mean Expression Ratio	1.18
Median Expression Ratio	1.18
Credible Interval	(0.989, 1.42)
Associated Peptides	4
Associated Spectra	4
Coverage	0.107

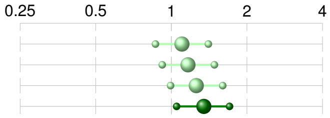

A	2.5	50	97.5	Sequence
1	0.87	1.1	1.4	ILLANFLAQTEALMK
1	0.92	1.2	1.5	MIPCDFMIPVQTQHPVR
1	1	1.3	1.6	KELQAAGLSGDALEK
1	1.0	1.3	1.7	QLFEADKDRFHK

```
1    MPLSADPHFK KLLEWHKANA SKLVLRQLFE ADKDRFHKFS LTLNTDHGDI LLDYSKNLVT EEVMKMLIEL AKSRGVESAR
81   ERMFSGEKIN FTENRAVLHI ALRNRSNTPI LVDGKDVVPE VNKVLDKMKH FCQKVRSGEW KGYTGKAITD VVNIGIGGSD
161  LGPLMVTEVL KPYSKGGPRV WFVSNIDGTH IAKTLAELHP ETTLFIIASK TFTTQETITN AVTAKEWFLH AAKDPSAVAK
241  HFVALSTNGP KVKEFGIDTE NMFEFWDWVG GRYSLWSAIG LSIALHIGFD NFESLLAGGH WMDKHFHTAP LEKNVPVLLA
321  MLGVWYINCY GCETHALLPY DQYMHRFAAY FQQGDMESNG KYITKKGSRV DYNTGPIVWG EPDTNGQHAF YQLIHQGTRM
401  IPCDFMIPVQ TQHPVRNGLH HKILLANFLA QTEALMKGKT ADEARKELQA AGLSGDALEK LLPHKVFEGN RPTNSIMFTK
481  LNPFTLGAII AMYEHKIFVQ GVVWDINSYD QWGVELGKQL AKKIEPELES DAPVTSHDSS TNGLISFIKK HRA
```

5.31 glutamic-oxaloacetic transaminase 2, mitochondrial (aspartate aminotransferase 2) [Gallus gallus]

Protein Accession	gi|45382953
Mean Expression Ratio	1.18
Median Expression Ratio	1.18
Credible Interval	(0.97, 1.43)
Associated Peptides	3
Associated Spectra	3
Coverage	0.104

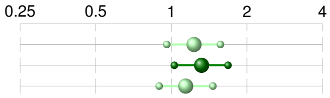

A	2.5	50	97.5	Sequence
1	0.96	1.2	1.6	YVTVQGISGTGSLR
1	1.0	1.3	1.7	IASLILNTPELR
1	0.9	1.1	1.5	SIILLHACAHNPTGVDPR

```
1    MALLQSRLLL SAPRRAAATA RASSWWSHVE MGPPDPILGV TEAFKRDTNS KKMNLGVGAY RDDNGKSYVL NCVRKAEAMI
81   AAKKMDKEYL PIAGLADFTR ASAELALGEN SEAFKSGRYV TVQGISGTGS LRVGANFLQR FFKFSRDVYL PKPSWGNHTP
161  IFRDAGLQLQ AYRYYDPKTC SLDFTGAMED ISKIPEKSII LLHACAHNPT GVDPRQEQWK ELASVVKKRN LLAYFDMAYQ
241  GFASGDINRD AWALRHFIEQ GIDVVLSQSY AKNMGLYGER AGAFTVICRD AEEAKRVESQ LKILIRPMYS NPPMNGARIA
321  SLILNTPELR KEWLVEVKGM ADRIISMRTQ LVSNLKKEGS SHNWQHITDQ IGMFCFTGLK PEQVERLTKE FSIYMTKDGR
401  ISVAGVASSN VGYLAHAIHQ VTK
```

5.32 thioredoxin [Gallus gallus]

Protein Accession	gi|45382053
Mean Expression Ratio	0.847
Median Expression Ratio	0.847
Credible Interval	(0.693, 1.03)
Associated Peptides	3
Associated Spectra	3
Coverage	0.2

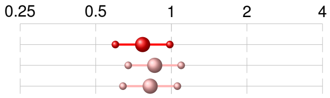

A	2.5	50	97.5	Sequence
1	0.6	0.77	0.98	CMPTFQFYK
1	0.67	0.86	1.1	KVQEFSGANK
1	0.64	0.82	1.1	VQEFSGANKEK

```
1       MVKSVGNLAD FEAELKAAGE KLVVVDFSAT WCGPCKMIKP FFHSLCDKFG DVVFIEIDVD DAQDVATHCD VKCMPTFQFY
81      KNGKKVQEFS GANKEKLEET IKSLV
```

5.33 PREDICTED: similar to Probable phospholipid-transporting ATPase IF (ATPase class I type 11B) (ATPas

Protein Accession	gi|118095259
Mean Expression Ratio	1.18
Median Expression Ratio	1.18
Credible Interval	(0.913, 1.53)
Associated Peptides	1
Associated Spectra	1
Coverage	0.00978

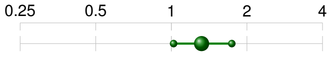

A	2.5	50	97.5	Sequence
1	1.0	1.3	1.7	MLERLMGRCSPTR

```
1       MRPEPPRALR GREPPPPPPA RGHRSLPALR PPIEARSRGS VLRDGMGWER EKAEPPLWPS SSPNNFPTQA MRAAPYVLQL
81      RSFVHTEASD AVAGTRAAQG EKQTIKTFTC LLPDRHLYKV ASKIDRDLVT LSNGGIASNL SKDLGRSDSF PLSVIGDSAN
161     SFGSEEGFDP PHQSDTRTIY IANRFPQHGH YIPQKFADNR IISSKYTVWN FVPKNLFEQF RRIANFYFLI IFLVQLMIDT
241     PTSPITSGLP LFFVITVTAI KQGYEDWLRH KADNEVNGAP VYVVRSGGLV KTRSKNIRVG DIVRVAKDET FPVDLVLLSS
321     DRVDGSCHVT TASLDGETNL KTHVAVPETA VLQSVANLDK LVAVIECQQP EADLYRFVGR ITISQQMEEI VRPLGPESLL
401     LRGARLKNTK EIFGVAVYTG METKMALNYK SKSQKRSAVE KSMNSFLIIY LIILLFEAIL STILKYAWQA EEKWDEPWYN
481     GKTEHERNSS KILRFISDFL AFLVLYNFII PISLYVTVEM QKFLGSFFIG WDLDLYHEET NQRAQVNTSD LNEELGQVEY
561     VFTDKTGTLT ENEMQFRECS INGIKYQEVN GKLTPEGFSE DSPDGNRHTL VRLLFFSPLG XYLNVTKHVS CFLQMKEEEL
641     FLKAVCLCHT VQISADQTDG ADGPWHANGI ASPLEYYASS PDEKALVEAA SRVGVVFMGT SGDSMEVKSL GKPERYKLLH
721     VLEFDPNRRR MSVIVESPSG EKLLFTKGAE SSILPRSKSG EIDKTRIHVD EFALKGLRTL CVAYRRFTPE EYQEIGKRLH
801     EARTALQQRE EKLADVFNFI ERDLELLGAT GVEDKLQEKV QETIEALRLA GIKVWVLTGD KHETAVSVSL SCGHFHRTMN
881     ILELVQHKSD STCAEQLRQL AKRIKEDHVI QHGLVVDGTS LSLALREHEK LFMEVCKNCS AVLCCRMAPL QKAKVVRLLK
961     TSPEKPITLA IGDGANDVSM IQEAHVGIGI MGKEGRQAVR NSDYAIARFK FLSKLLFVHG HFYYIRIATL VQYFFYKNVC
1041    FITPQFLYQF FCLFSQQTLY DSVYLTLYNI CFTSLPVLIY SLFEQHVHPH VLQSKPVLYR DISKNAHLGY KPFLYWTILG
1121    FLHAFVFFYG SYLLMGEDTS LLGNGQMFGN WTFGTLVFTV MVITVTMKMA LETHFWTWIN HFVTWGSIVF YFIFSLFYGG
1201    IIWPFLHTQD MYFVFVQLLS SGSAWFAIIL IVVACLFLDV VKKVLYRHLQ PTSTEKAQLT ETGSGINCMD SMCCFSDGET
1281    ACTSVRRMLE RLMGRCSPTR VNRSWSSTDP FYANDRSILT LSTMDSSTC
```

5.34 glceraldehyde-3-phosphate dehydrogenase

Protein Accession	gi|211801
Mean Expression Ratio	1.18
Median Expression Ratio	1.18
Credible Interval	(1.03, 1.34)
Associated Peptides	8
Associated Spectra	11
Coverage	0.294

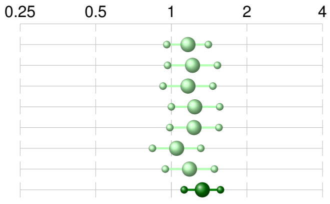

A	2.5	50	97.5	Sequence
2	0.96	1.2	1.4	VPTPNVSVVDLTCR
1	0.97	1.2	1.5	LVSWYDNEFGYSNR
1	0.93	1.2	1.5	GAAQNIIPASTGAAK
1	1	1.2	1.6	VIHDNFGIVEGLMTTVHAITATQK
1	0.99	1.2	1.5	LEKPAK
1	0.84	1.1	1.3	YDSTHGHFK
1	0.94	1.2	1.5	AGAHLKGGAK
3	1.1	1.3	1.6	LEKPAKYDDIKR

```
1       MVKVGVNGFG RIGRLVTRAA VLSGKVQVVA INDPFIDLNY MVYMFKYDST HGHFKGTVKA ENGKLVINGH AITIFQERDP
81      SNIKWADAGA EYVVESTGVF TTMEKAGAHL KGGAKRVIIS APSADAPMFV MGVNHEKYDK SLKIVSNASC TTNCLAPLAK
161     VIHDNFGIVE GLMTTVHAIT ATQKTVDGPS GKLWRDDRGA AQNIIPASTG AAKAVGKVIP ELNGKLTGMA FRVPTPNVSV
241     VDLTCRLEKP AKYDDIKRVV KAAADGPLKG ILGYTEDQVV SCDFNGDSHS STFDAGAGIA LNDHFVKLVS WYDNEFGYSN
321     RVVDLMVHMA SKE
```

5.35 hemagglutinin [Influenza A virus (A/chicken/NJ/4236-18/1998(H11N3))]

Protein Accession	gi|89275869
Mean Expression Ratio	0.852
Median Expression Ratio	0.851
Credible Interval	(0.66, 1.1)
Associated Peptides	1
Associated Spectra	1
Coverage	0.0124

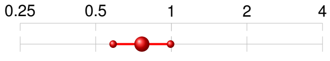

A	2.5	50	97.5	Sequence
1	0.59	0.76	0.99	SFHNVHR

```
1       MKKVLLFAAI IICIQADEIC IGYMSNNSTE KVDTIIESNV TVTSSVELVE NEHTGSFCSI DGKAPISLGD CSFAGWILGN
81      PMCDDLIGKT SWSYIVEKPN PVNGICYPGT LENEEELRLK FSGVLEFSKF ETFTSNGWGA VNSGAGVTAA CKFGSSNSFF
161     RNMVWLIHQS GTYPVIRRTF NNTKGRDVLM VWGVHHPATL KEHQDLYKKD SSYVAVGSES YNRRFTPEIN TRPKVNGQAG
241     RMTFYWTIVK PGESITFESN GAFLAPRYAF ELVSLGNGKL FRSDLNIESC STKCQSEIGG INTNRSFHNV HRNTIGDCPK
321     YVNVKSLKLA TGLRNVPAIA TRGLFGAIAG FIEGGWPGLI NGWYGFQHRN EEGTGIAADK ESTQKAIDQI TSKVNKIVDR
401     MNTNFESVQH EFSEIEERIN QLSKHVDDSV IDIWSYNAQL LVLLENEKTL DLHDSNVRNL HEKVRRMLKD NAKDEGNGCF
481     TFYHKCDNEC IEKVRNGTYD HKEFEEESKL NRQEIEGVKL NSNGNVYKIL SIYSCIASSL VLAAIIMGFI FWACSNGSCR
561     CTICI
```

5.36 fatty acid binding protein 3 [Gallus gallus]

Protein Accession	gi|71894843
Mean Expression Ratio	1.17
Median Expression Ratio	1.17
Credible Interval	(1, 1.38)
Associated Peptides	4
Associated Spectra	11
Coverage	0.338

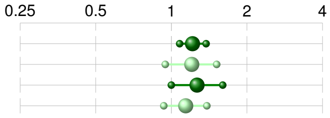

A	2.5	50	97.5	Sequence
7	1.1	1.2	1.4	LDEEFDETTADDR
1	0.95	1.2	1.5	LILTLTMGNVVSTR
1	1	1.3	1.6	LVDTANFDEYMK
2	0.93	1.1	1.4	LVHVQK

```
1       MVEAFVGTWK LVDTANFDEY MKALGVGFAT RQMAGLTKPT TIIEVDGDKA TVKTHSTFKN TEISFKLDEE FDETTADDRH
81      VKSLVKLDGG KLVHVQKWDG KETSLVRELK DGKLILTLTM GNVVSTRTYE KAS
```

5.37 PREDICTED: similar to LanC lantibiotic synthetase component C-like 2 (bacterial) [Gallus gallus]

Protein Accession	gi|118086105
Mean Expression Ratio	0.853
Median Expression Ratio	0.855
Credible Interval	(0.66, 1.10)
Associated Peptides	1
Associated Spectra	1
Coverage	0.055

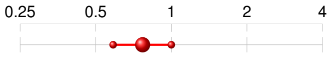

A	2.5	50	97.5	Sequence
1	0.59	0.77	1	AFANPYPDYQPQGAAAAQSAAEAQR

```
1       MGESMSKRLK LQLGGEAEME ERAFANPYPD YQPQGAAAAQ SAAEAQRDRV LLPPEEDPLP FDSDGKVSAQ FTKRIQTKIK
81      DLLQQMEEGL KTADPHDCSA YTGWTGIALL YLQLYRVTKN QSHLQRSLDY VKRILRNLSG RRVTFLCGDA GPLAVGAVVY
161     HVLKNESESK ECVARLLQLQ RTVISMDAEL PDELLYGRAG YLYALLYLNT EIASDTVPQS IIKEVIDAII ESGKNFSKEE
241     RKTERCPLLY QWHRKQYVGA AHGVAGIYYM LMQPIANVDQ ETLTELVKPS IDYVRHKKFR SGNYPSSLSN ETDRLVHWCH
321     GAPGVIHMLM QAYKTFKEDK YLKDAMECSD VIWQRGLLRK GYGICHGTAG NGYSFLSLYN LTQDKKYLYR ACKFAEWCLE
401     YGAHGCRIPD RPYSLFEGMA GAIHFLSDIS VPETSRFPAF ELGPQRREDK MEQDS
```

5.38 creatine kinase, brain [Gallus gallus]

Protein Accession	gi|45384340
Mean Expression Ratio	1.17
Median Expression Ratio	1.17
Credible Interval	(1.05, 1.30)
Associated Peptides	12
Associated Spectra	23
Coverage	0.438

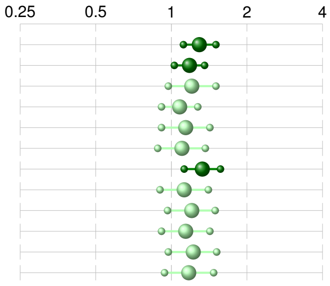

A	2.5	50	97.5	Sequence
4	1.1	1.3	1.5	GTGGVDTAAVGGVFDVSNADR
5	1.0	1.2	1.4	TDLNADNLQGGDDLDPNYVLSSR
1	0.97	1.2	1.5	ELFDPVIEDR
3	0.91	1.1	1.3	LGFSEVELVQMVVDGVK
1	0.91	1.1	1.4	FCTGLTQIETLFK
1	0.88	1.1	1.4	VLTLDLYK
3	1.1	1.3	1.6	LSVEALGSLGGDLK
1	0.9	1.1	1.4	TFLVWINEEDHLR
1	0.97	1.2	1.5	YSVDDEYPDLSVHNNHMAK
1	0.91	1.1	1.4	GFCLPPHCSR
1	0.97	1.2	1.5	PFSNSHNLLK
1	0.94	1.2	1.5	LPNLGKHEK

```
1      MPFSNSHNLL KMKYSVDDEY PDLSVHNNHM AKVLTLDLYK KLRDRQTSSG FTLDDVIQTG VDNPGHPFIM TVGCVAGDEE
81     SYEVFKELFD PVIEDRHGGY KPTDEHKTDL NADNLQGGDD LDPNYVLSSR VRTGRSIRGF CLPPHCSRGE RRAIEKLSVE
161    ALGSLGGDLK GKYYALRNMT DAEQQQLIDD HFLFDKPVSP LLLASGMARD WPDARGIWHN DNKTFLVWIN EEDHLRVISM
241    QKGGNMKEVF TRFCTGLTQI ETLFKSKNYE FMWNPHLGYI LTCPSNLGTG LRAGVHIKLP NLGKHEKFGE VLKRLRLQKR
321    GTGGVDTAAV GGVFDVSNAD RLGFSEVELV QMVVDGVKLL IEMEKRLEKG QSIDDLMPAQ K
```

5.39 glutaminase C [Gallus gallus]

Protein Accession	gi|71895159
Mean Expression Ratio	0.858
Median Expression Ratio	0.86
Credible Interval	(0.661, 1.11)
Associated Peptides	1
Associated Spectra	1
Coverage	0.0240

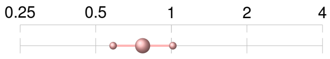

A	2.5	50	97.5	Sequence
1	0.59	0.77	1.0	GAQQPAAPSEPPDGGR

```
1      MMRLWRAALL RELLLLGPPG AAGGGGPGSG SRRLPPQLAA LGAGPATRRG PGPARGLCTR SEGALEEPGK GAQQPAAPSE
81     PPDGGRNNNH GSGDKELAGG GGGGENKVKQ GLLPSLEDLL FYTIAEGQEK IPVHKFITAL KSTGLRTSDP RLKECMDMLR
161    LTLQTTSDGV MLDKDLFKKC VQSNIVLLTQ AFRRKFVIPD FMSFTSHIDE LYESAKKQSG GKVADYIPQL AKFSPDLWGV
241    SLCTVDGQRH SVGDTKVPFC LQSCVKPLKY AIAVNDLGTE YVHRYVGKEP SGLRFNKLFL NEDDKPHNPM VNAGAIVVTS
321    LIKQGANNAE KFDYVMQFMN KMAGNEYVGF SNATFQSERE SGDRNFAIGY YLKEKKCFPE GTDMVAILDF YFQLCSIEVT
401    CESASVMAAT LANGGFCPII TGERVLSPEA VRNTLSLMHS CGMYDFSGQF AFHVGLPAKS GVAGGILVV PNVMGLMCWS
481    PPLDKMGNSV KGIHFCHDLV SLCNFHNYDN LRHFAKKLDP RREGGDQRVK SVINLLFAAY TGDVSALRRF ALSGMDMEQR
561    DYDSRTALHV AAAEGHVDVV KFLLEACKVN PFPKDRWNNT PMDEALHLDT MMCLKFSKSI RSHTHRQRIP VMARRTERFI
641    KTWMAYYNHS QLDPKNPISY LFNCGILK
```

5.40 PREDICTED: similar to L-3-hydroxyacyl-Coenzyme A dehydrogenase, short chain [Gallus gallus]

Protein Accession	gi|118090053
Mean Expression Ratio	1.16
Median Expression Ratio	1.16
Credible Interval	(0.893, 1.50)
Associated Peptides	1
Associated Spectra	1
Coverage	0.0476

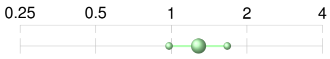

A	2.5	50	97.5	Sequence
1	0.98	1.3	1.7	KFADKPEAGAEFIEK

```
1      MAFATRPFVR AASSATAAAT AAAKKLIVKH VTVIGGGLMG AGIAQVAAAS GHTVVLVDQS DEILKKSTKG IEESLKRVTK
81     KKFADKPEAG AEFIEKTLKN LTTSTDAVAV VHSTDLVIEA IVENQEIKSE LFKRLDKFAP EHTIFTSNTS SLQITQLANS
161    TTRQDRFGGL HFFNPVPMMK LVEVVKTPMT SQKTYESLMD FSKAVGKSPV SCKDTPGFIV NRLLVPYMME AVRLFERGDA
241    SKEDIDVAMK LGAGYPMGPF ELLDYVGLDT SKYIIDGWHA IEPNNPLFLP SPLLDKLVEE KKLGRKTGEG FYKYN
```

5.41 lactate dehydrogenase B [Gallus gallus]

Protein Accession	gi|45383766
Mean Expression Ratio	1.16
Median Expression Ratio	1.16
Credible Interval	(0.976, 1.38)
Associated Peptides	3
Associated Spectra	7
Coverage	0.090

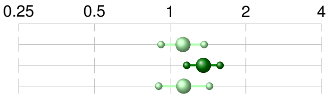

A	2.5	50	97.5	Sequence
2	0.92	1.1	1.4	QVVESAYEVIR
4	1.2	1.4	1.6	IVVVTAGVR
1	0.9	1.1	1.4	LKDDEVAQLK

```
1      MATLKEKLIT PVAAGSTVPS NKITVVGVGQ VGMACAISIL GKGLCDELAL VDVLEDKLKG EMMDLQHGSL FLQTHKIVAD
81     KDYAVTANSK IVVVTAGVRQ QEGESRLNLV QRNVNVFKFI IPQIVKYSPN CTILVVSNPV DILTYVTWKL SGLPKHRVIG
161    SGCNLDTARF RYLMAERLGI HPTSCHGWIL GEHGDSSVAV WSGVNVAGVS LQELNPAMGT DKDSENWKEV HKQVVESAYE
241    VIRLKGYTNW AIGLSVAELC ETMLKNLYRV HSVSTLVKGT YGIENDVFLS LPCVLSASGL TSVINQKLKD DEVAQLKKSA
321    DTLWSIQKDL KDL
```

5.42 filamin [Gallus gallus]

Protein Accession	gi|45383035
Mean Expression Ratio	0.867
Median Expression Ratio	0.867
Credible Interval	(0.71, 1.06)
Associated Peptides	3
Associated Spectra	3
Coverage	0.0184

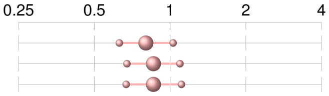

A	2.5	50	97.5	Sequence
1	0.63	0.8	1.0	SAPGVVGPAEADLDFDILR
1	0.67	0.86	1.1	VTAQGPGIEPTGNVANR
1	0.67	0.86	1.1	YGGDEIPYSPYR

```
1      MSAAGRAREP EAEMPATEKD LADDAPWKRI QQNTFTRWCN EHLKCVQKRV GNLQTDLGDG LRLIALLEVL SQKKLGRRYN
81     ARPTFRQMQL ENVSVALDFL ERENIKLVSI DSKAIVDGNL KLILGLVWTL ILHYSISMPV WEDEDEEEAR RQTPKQRLLG
161    WIQNRLPQLP VTNFSRDWSS GRALGALVDS CAPGLCPDWE SWDPERAVEN AREAMQQADE WLGIPQVITP EEIVDPNVDE
241    HSVMTYLSQF PKAKLKPGAP LRPKLNPKKA RAYGPGIEPT GNVVKQRAEF TVETISAGQG DVTVYVEDPE GHREEAKVVP
321    NNDKNRTFSV SYVPKVTGVH KVTVLFAGQH IAKSPFEVAV GRAAGDAGRV TAQGPGIEPT GNVANRTTHF DILTAGAGPG
401    SPDVVVTDPG GRRVEALLEG RGDGAFRCSY RPTAEGTHSV AVTFGGTPIP RSPFAVGVGQ ACNPSQCRAE GRGLQPKGLR
481    VKEAADFKVY TKGAGSGELK VAVKGPKGAK ALKQKDLGEG VFGCEYTPTV PGAHSVTFTY GGQPIPRSPF EVQVAPAGGA
561    PKVRAWGRGL QGGMVGCSAD FVVEALGDDV GTLGFSVEGP SQARIECSDG GDGCCAVGYR PLEAGLYAVH VLCDGDDIAG
641    SPFMATVRGP ERGCCPDKVK AQGPGLEPTG VAVNQPAEFT VDAKQAGKGP LTVQIQDSEG TPVEVAVKDR GDGTYSCSYT
721    PRKALKHTAV LAWGGVNIPH SPYRVSVGPG SHPNKVKVYG PGVGKTGLKA HEPTYFTVDC SEQGQGDVSI GIKSAPGVVG
801    PAEADLDFDI LRNADDTFTV RYTPRGAGMH TVMVLFADQP TPSSPIRVRV DPSHDASKVK AEGPGLSRTG LELGKPTQFT
881    VLSKGAGRAA LDVEVSGPGK GDAVRDLEVR DNRDGTHTVT YTPVQQGNLE VSVKYGGDPI PKSPFGVTVS PAVELGRIKI
961    SGLDDKLEVG KEQEFTVKAK GAGGQGKVGA RIVGPTHKAV PCRVEAGPGA ESSVVRWTAP HEGPYEVEVT YDGHPVPGSP
1041   FPVEAVPPTD PSKVRAFGPG LQGGQAGVPA PFTIDTKGAG TGGLGLTVEG PCEAPIECQD HGDGTCSVSY LPAVPGDYTI
1121   NILFAGAHVP GSPFRAPVAA PFDPTKVTCE GPGLEKGVVG QRSHFRVDCS RAGSAELSIG IASDGGAQAE VCVEDNGDGT
1201   YNIGYTALSP GPHSITVLYG GQPVPHFPAK VCVEPAGDAA AVKVYGPGVE GKGVFRGALT HFEVDARALG AAGGAAVTAR
1281   IRKPSGGGVE AAVEDRGDGT YGVQYTPYEE GVHTVEVSYG GSPLPAGPFR VAVTEGCDPS RVRVHGPGIA GGTARQPNCF
1361   TVETRGAGTG GLGLAMEGPS EAQLSCTDNK DGSCSVQYIP SAPGTYSLNV TYGGRRVPGS PFRVPVVETG GGGGAADAGM
1441   VKCAGPGLTK GEVRVNPVQR FDVDCSKAGE AALDVKVVGP KGVVEPVEVT DNGDGTRSVS YVPSREGPYS ISVRYGDQEV
1521   PRSPFKVKAL PSHDASKVRA SGPGLNTSGV PASLPVEFTI DAKDAGEGLL AVQITDPEGK PKKASIRDNG DGTYTVSYVP
1601   DTTGRYTILI KYGGDEIPYS PYRIRAVPAG DASKCTVTGP GLGPTIQLGE QTLLTVDAKA AGPGKVTCAV RAPDGAEADV
1681   DVVENADGTF DIFYTAPQPG KYIICVRFGG EHVPNSPFQV MATERPLLGV NGLDVGLRPF DLVIPLTIAK GEITGEVRMP
1761   SGKVSRPAIT DNKDGTVTVR FEPSEAGLHQ MDIRYDSVHI PGSPLQFYVD YVNSGHVTAY GPGLTHGTVN KAAQFTVNTK
1841   DAGEGGLSLA VEGPSKAEIS CTDHQDGTCT VSYLPVLPGD YSIVVKYNDK HIAGSPFTAR ITGDDSLRQS HLKVGASADI
1921   PLDIAESDLS QLAASVTAPS GRKEPCQLKR LRDGHVGISF VPQEVGEHLV HISRGGQPLP RSPITVSISQ AELGDAARVR
2001   VGGPGLHEGR TLTPTHFTID TRDAGYGGLS LSIEGPSKVD ISTEELPDGT CRVGYCPTEP GNYIIAVQFG EQHVPGSPFS
2081   VKVTGEGRVK ESITRRRRAP PEAHVGTACD LSLKMPEPTA HDVTAQVTAP SGTSMAAQVL EGERGAYSIR FVPEESGVHS
2161   VSVKDRGQHV PGSPFQFTVG PLGEGGAHKV RAGGPGLERA ETGVPAEFSI WTREAGAGGL SISVEGPSKA EIAFEDRKDG
2241   SCGVSYVVQE PGDYEVAVKF NEEHIPDSPF VVTAAPNSDA ARRLTVSSLR ESGLKVHQPA SFAVSLNGAK GALDAKVHSP
2321   SGAVEECHIS EVTEDKYAVR FIPRENGVYS IDVKFDGAHI PGSPFKVRVG DPGQAGDPGL VTAYGAGLEG GTTGSPAEFV
2401   VNTLHAGPGA LSVTIEGPSK VKMECVECPE GFRVVYTPMA PGSYLIAIKF GGPHHIAGSP FKAKVTGPRL VSTHSLRESS
2481   SVLVDSGMGG GVSVPPPSAT AAAPSSTSAP PGGVPQFSDP SKVVAKGLGL SKAFVGQRNS FTVDCSKAGS NMLLVGIQGP
2561   RAPCEELQVK HLGRQLYSVA YVVRERGEHL LVLKWGDHHV PHSPFRITVP
```

5.43 gelsolin [Gallus gallus]

Protein Accession	gi|45384386
Mean Expression Ratio	1.15
Median Expression Ratio	1.15
Credible Interval	(0.894, 1.49)
Associated Peptides	1
Associated Spectra	1
Coverage	0.0206

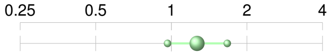

A	2.5	50	97.5	Sequence
**1**	**0.97**	**1.3**	**1.7**	**AMAAQHGMEDDGSGKK**

```
1     MGKQGFGYIF LTIFCTMALK LNCVSSVSVA GLGYVVTAAV VLSAVPVSMV EHAEFSKAGK EPGLQIWRIE KFDLVPVPKN
81    LYGDFFTGDS YLVLNTIRQR SGNLQYDLHF WLGDESSQDE RGAAAIFTVQ MDDYLQGKAV QHREVQGHES STFLGYFKSG
161   IKYKAGGVAS GFRHVVPNEV TVQRLLQVKG RRTVRATEVP VSWESFNTGD CFILDLGSNI YQWCGSNSNR QERLKATVLA
241   KGIRDNEKNG RAKVFVSEEG AEREEMLQVL GPKPSLPQGA SDDTKTDTAN RKLAKLYKVS NGAGNMAVSL VADENPFSQA
321   ALNTEDCFIL DHGTDGKIFV WKGRSANSDE RKAALKTATD FIDKMGYPKH TQVQVLPESG ETPLFKQFFK NWRDKDQTEG
401   LGEAYISGHV AKIEKVPFDA ATLHTSRAMA AQHGMEDDGS GKKQIWRIEG SEKVPVDPAT YGQFYGGDSY IILYDYRHAG
481   KQGQIIYTWQ GAHSTQDEIA TSAFLTVQLD EELGGSPVQK RVVQGKEPPH LMSMFGGKPL IVYKGGTSRE GGQTTPAQTR
561   LFQVRSSTSG ATRAVELDPA ASQLNSNDAF VLKTPSAAYL WVGRGSNSAE LSGAQELLKV LGARPVQVSE GREPDNFWVA
641   LGGKAPYRTS PRLKDKKMDA YPPRLFACSN KSGRFTIEEV PGDLTQDDLA TDDVMILDTW DQVFVWIGKD AQEEEKTEAL
721   KSAKRYIETD PASRDKRTPV TLVKQGLEPP TFSGWFLGWD DDYWSVDPLQ RAMADVDV
```

5.44 Ovalbumin (Egg albumin) (Plakalbumin) (Allergen Gal d 2) (Gal d II)

Protein Accession	gi|129293
Mean Expression Ratio	1.15
Median Expression Ratio	1.15
Credible Interval	(0.89, 1.48)
Associated Peptides	1
Associated Spectra	1
Coverage	0.0415

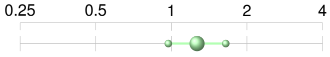

A	2.5	50	97.5	Sequence
**1**	**0.97**	**1.3**	**1.6**	**GGLEPINFQTAADQAR**

```
1     MGSIGAASME FCFDVFKELK VHHANENIFY CPIAIMSALA MVYLGAKDST RTQINKVVRF DKLPGFGDSI EAQCGTSVNV
81    HSSLRDILNQ ITKPNDVYSF SLASRLYAEE RYPILPEYLQ CVKELYRGGL EPINFQTAAD QARELINSWV ESQTNGIIRN
161   VLQPSSVDSQ TAMVLVNAIV FKGLWEKAFK DEDTQAMPFR VTEQESKPVQ MMYQIGLFRV ASMASEKMKI LELPFASGTM
241   SMLVLLPDEV SGLEQLESII NFEKLTEWTS SNVMEERKIK VYLPRMKMEE KYNLTSVLMA MGITDVFSSS ANLSGISSAE
321   SLKISQAVHA AHAEINEAGR EVVGSAEAGV DAASVSEEFR ADHPFLFCIK HIATNAVLFF GRCVSP
```

5.45 PREDICTED: hypothetical protein [Gallus gallus]

Protein Accession	gi|118089621
Mean Expression Ratio	0.873
Median Expression Ratio	0.874
Credible Interval	(0.675, 1.13)
Associated Peptides	1
Associated Spectra	1
Coverage	0.0518

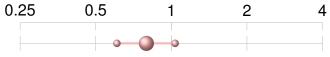

A	2.5	50	97.5	Sequence
**1**	**0.61**	**0.8**	**1.0**	**MPGCPRAGAAGPR**

```
1     MPGCPRAGAA GPRRSFVIRS APNAAGAGAP MPPRPRSAAR AAQPAFPSRC AAARPATPLP AARSRGAAGR QGRAGAASRS
81    SPRRRGGGGR RAELPDEPGG RSTASRSPRA AAGPGPERSF AKTRAAPSHS LAGARRPSRG YAAPHTRAPT PAPRTRAPHS
161   RLLRRPSRLS RLRPRPGPAA GRGPLEPRLT PLVTHRSEGP ASSSRISRHG VAHRRVTPGV NVPELGARSS SVLPRRFGTW
241   ASKPPVQWVA H
```

5.46 MARCKS-like 1 [Gallus gallus]

Protein Accession	gi|123959732
Mean Expression Ratio	1.14
Median Expression Ratio	1.14
Credible Interval	(0.965, 1.35)
Associated Peptides	4
Associated Spectra	6
Coverage	0.268

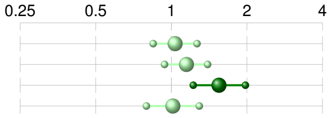

A	2.5	50	97.5	Sequence
2	0.85	1.0	1.3	DDGDSAASSPTEEQSK
2	0.94	1.1	1.4	AEEDPASGTAPQEER
1	1.2	1.5	2.0	GDAAAAQQPPEPTAAEPAAAEQKEE
1	0.8	1.0	1.3	KDDGDSAASSPTEEQSK

```
1       MGSQGSKAAK AEGSDPPGGN AAVTEPSKAN GQENGHVRLN GDMTPKAGGD PTPLNGAGSA EPPREDGTGG AGGEDTIEPA
81      PPADGGEAKP EGAAAPKDTP KKKKKFSFKK SFKLSGISFR KNKKDDGDSA ASSPTEEQSK AEPKAEEDPA SGTAPQEERS
161     GEGQSGAEPE GAAGGPGAEE EKPPAENRGD AAAAQQPPEP TAAEPAAAEQ KEE
```

5.47 death-associated protein [Gallus gallus]

Protein Accession	gi|71896843
Mean Expression Ratio	1.14
Median Expression Ratio	1.14
Credible Interval	(0.884, 1.48)
Associated Peptides	1
Associated Spectra	1
Coverage	0.221

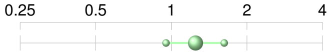

A	2.5	50	97.5	Sequence
1	0.95	1.3	1.6	GDKDFPPAAAQVAHQKPHPSVEK

```
1       MSSPPKEKAE TRAGHPPAVK AGGMRIVQKH PHSSDTKEEK DKDDQDWETS SPPKPTVFIS GVIARGDKDF PPAAAQVAHQ
81      KPHPSVEKLP HPQHVKQHIH QPRK
```

5.48 Splicing factor, arginine/serine-rich 1

Protein Accession	gi|82233969
Mean Expression Ratio	1.14
Median Expression Ratio	1.14
Credible Interval	(0.937, 1.40)
Associated Peptides	3
Associated Spectra	3
Coverage	0.128

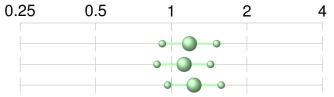

A	2.5	50	97.5	Sequence
1	0.92	1.2	1.5	DAEDAVYGR
1	0.88	1.1	1.4	DGTGVVEFVR
1	0.96	1.2	1.6	GGPPFAFVEFEDPR

```
1       MSGGGVIRGP AGNNDCRIYV GNLPPDIRTK DIEDVFYKYG AIRDIDLKNR RGGPPFAFVE FEDPRDAEDA VYGRDGYDYD
81      GYRLRVEFPR SGRGTGRGGG GGGGGGAPRG RYGPPSRRSE YRVIVSGLPP SGSWQDLKDH MREAGDVCYA DVFRDGTGVV
161     EFVRKEDMTY AVRKLDNTKF RSHEGETAYI RVKVDGPRSP SYGRSRSRSV VVAEAVVGAT AEAAVIPQEE AEDLHATLPA
241     TADPDLVHKR SLALIFL
```

5.49 PREDICTED: hypothetical protein [Gallus gallus]

Protein Accession	gi|50738728
Mean Expression Ratio	1.14
Median Expression Ratio	1.14
Credible Interval	(0.885, 1.47)
Associated Peptides	1
Associated Spectra	1
Coverage	0.0905

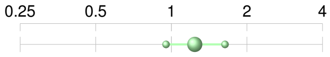

A	2.5	50	97.5	Sequence
**1**	**0.95**	**1.2**	**1.6**	**AEQAEDLQDDAEGGNSAEEK**

```
1       MGEFFPIEDN EETEISEETG LDPKGQPLSP TKCKAEQAED LQDDAEGGNS AEEKQNVEET EGRSQEADET KPSAVASLSS
81      EEAKPSEDKD SLNDSKGTRE SDPVHPMGCE QDQAQGQQED AVEEEMKTGA DSQSSCMEIE EPLLNQEDTT EHEKPLIPSV
161     EEKEKEGGRE TQEEEGGIQE QEETAAEPMK VELACEAAEA STEMDSQVES IKDPMETVPE A
```

5.50 otokeratin [Gallus gallus]

Protein Accession	gi|45384378
Mean Expression Ratio	0.878
Median Expression Ratio	0.879
Credible Interval	(0.676, 1.13)
Associated Peptides	1
Associated Spectra	1
Coverage	0.0163

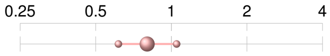

A	2.5	50	97.5	Sequence
**1**	**0.62**	**0.8**	**1.0**	**KEEKEQIK**

```
1       MSRSVSFSSR SVAVPGVSQV RVSSVSSVRG GGSLNKAGGF GSSSLYNLGS ANKRISLGGS SSYSVRSGYG YGGMGFGGAI
81      APSAGIQEVT VNQNLLTPLN LEIDPNIQRV RKEEKEQIKT LNNRFASFID KVRFLEQQNK MLETKWSLLQ DQKTTRSNIV
161     PMFEAYITNL RRQLDGLLND KGRLEGELRN MQDLVEDFKA KYEDEINKRT TAENEFVVLK KDVDAAYMNK VELEAKVDAL
241     TDEINFLRSL YEAELRELQA QISDTSVVLS MDNSRNLDLD SIIAEVKAQY EDIANRSRAE AESWYQSKFE ALQVTAGKHG
321     DDLRNTKNEI TEINRVIQRL QGEIENAKAQ RAKMEAAIAE AEERGELAVK DARAKLEELE AALQKAKQDM ARQLREYQEL
401     MNVKLALDIE IATYRKLLEG EESRLAGDGV GSVNISMVSS SGGGSSGFLG GGVRGGLALG AGMGSGALGF SSGGSTKSYT
481     VTTTSSTRSF RK
```

5.51 PREDICTED: similar to Malate dehydrogenase 2, NAD (mitochondrial) [Gallus gallus]

Protein Accession	gi|50758110
Mean Expression Ratio	1.14
Median Expression Ratio	1.14
Credible Interval	(0.895, 1.44)
Associated Peptides	1
Associated Spectra	2
Coverage	0.037

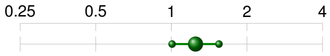

A	2.5	50	97.5	Sequence
**2**	**1**	**1.2**	**1.5**	**GCDVVVIPAGVPR**

```
1       MRGAVEVVGV TAAAMLSRLA RPAAVLCRGL ATSAQNNAKV AVLGASGGIG QPLSLLLKNS PLVSRLTLYD IAHTPGVAAD
81      LSHIETRANV KGFLGPEQLP ECLKGCDVVV IPAGVPRKPG MTRDDLFNTN ASIVATLTTA CAKHCPEAMI CIISNPVNST
161     IPITSEVFKK HGVYNPNRIF GVTTLDIVRA NTFVAELKGL DPARVSVPVI GGHAGKTIIP LISQCTPKVD FPQDQLEKLT
241     GRIQEAGTEV VKAKAGAGSA TLSMAYAGAR FVFSLVDAMN GKEGVIECSF VRSEETESPY FSTPLLLGKN GIEKNLGIGK
321     ITPFEEKMVA EAMAELKASI KKGEDFAKNF K
```

5.52 PREDICTED: hypothetical protein [Gallus gallus]

Protein Accession	gi|118082056
Mean Expression Ratio	0.88
Median Expression Ratio	0.88
Credible Interval	(0.712, 1.09)
Associated Peptides	2
Associated Spectra	3
Coverage	0.0217

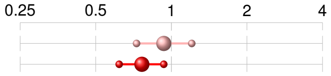

A	2.5	50	97.5	Sequence
**1**	**0.73**	**0.93**	**1.2**	**MHKEAAEARAR**
**2**	**0.62**	**0.76**	**0.93**	**GTEKILQER**

```
1     MRKYAKVEQL YKGSLKPLFS AGKLTSSRVA ELKAKYNLLH ETVRSLQESE IQLLQEAKRL SVDLEQQQHE LEKAEQFPEE
81    SSSEVCRIRQ QLLNCQNEYN AIKEREHEIQ FRIKCLQEEK RLLEKEYERI PKQGEADKKM RKMKEECDEL HKEVIQRKAE
161   VNAMKEDISS KQELMLIDKK EVEKLLEDQD SLKDELVKIL GVPVQLKKEA EKINQKKIDA EKKNEDLNNQ MQELNSTLND
241   IDKGTEKILQ EREDVMKELD GKRALLESKE QECAALARLL EIGREKQLAI LVERDSLNQK LNERIFEKKQ QQETLIYMQG
321   EKDKELRNLK KMELQMKMIH ESLQQEKSQH KTLKLEAEAI PKINGVLLER RRELQKEIEM KKRCLAEQEM VSDTDARRLE
401   ECIAEEGRLF KEQEKCRSEL SRLAHLTWLK VEEKEQKSRE VQKVQIQLQN IIKEIKRKDL EIEEYKKRKR RVHKQLQGVA
481   NMCDVIKNER NKCMHLVNVA QQKTAEIEDR IKVKASEIET LRNTLITQER ELQKQHMKNK NNAAIKESLK NDCSKVAQVM
561   YEMNEKKKQQ VLDLDGLTNA VTRIEEEIAQ LHKKYKRATE EQTESGLLLR SREEEICILY EKINAQEFLC RKGDIEMQAT
641   DEKISFLKMK VAEKERQIKF WLKALPMKRV LDAELVVLQI QYSQCKDRIK EMEEIFVDPT NESRKRDLGG KDPSAPELQK
721   KIKQLEVELV QKEEKLLETD IIYQHIARLT DRIRATAENG KQGTLLLATR INELQKKIKD RTQKMMALVA ELSMKQALAI
801   KLQQEMRDRE EFLMIVSSRI DQGLPPPKET EIEWLKVLRN EKMHKEAAEA RARQAAEEEQ AAVPGHVLTT AEPRPTAYVP
881   DDAYSLPVPR PYGALAPFKP SEPGSNIRHF RKPIIKPIEI
```

5.53 PREDICTED: similar to Elongation factor Tu, mitochondrial precursor (EF-Tu), partial [Gallus gallus

Protein Accession	gi|118127631
Mean Expression Ratio	1.14
Median Expression Ratio	1.14
Credible Interval	(0.882, 1.46)
Associated Peptides	1
Associated Spectra	1
Coverage	0.2

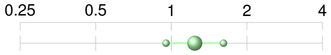

A	2.5	50	97.5	Sequence
**1**	**0.95**	**1.2**	**1.6**	**YEDIDKAPEER**

```
1     LSESGGARFQ RYEDIDKAPE ERARGITINA AHVEYSTARR HYAHTDCPGH ADYVK
```

5.54 PREDICTED: similar to MGC83099 protein [Gallus gallus]

Protein Accession	gi|118091687
Mean Expression Ratio	1.13
Median Expression Ratio	1.13
Credible Interval	(0.876, 1.47)
Associated Peptides	1
Associated Spectra	1
Coverage	0.0412

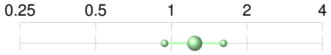

A	2.5	50	97.5	Sequence
**1**	**0.94**	**1.2**	**1.6**	**QHGKENFELLSYPGAGHR**

```
1     MWRAAVAAKA PARALCRAPA RRTVSVAVTP AAGLADERVD TRVAGLGPGQ PVTLRAVVSD ERGCLFQSCA HYRADGRGEL
81    HLGTDASHGG NYTGVQPMGL FWSLAPAGME RPYQRLVPRS TGTPMKVEML VHQGHSQPGA IHGPVMAKAE VERWFTAPGV
161   RRIRLKEGGV RGSLFLPPGD GPFPGVIDMY GDEGGLIEFR SSLLATRGFA ALSLPYFDFE DLPKVMKEFK LEYFQEAARF
241   LQRHPKVKGP GVGVIGTGKG AELALSMITF LPEVVAAVCI SGCSSNTVAD LHYGEITLPG LRFDMNKVSV SDTGVFDTFE
321   ALDDPANPAN SSCTIPIEKA EGHFLLVVGE ADRMWKSSLY AELAIGRLRQ HGKENFELLS YPGAGHRIDP PSTPFCQVAL
401   DRVLGVPVLG GGESKAHAHA QEHSWGKIQE FLHLHLG
```

5.55 PREDICTED: similar to Diacylglycerol kinase, theta [Gallus gallus]

Protein Accession	gi|118104544
Mean Expression Ratio	0.885
Median Expression Ratio	0.883
Credible Interval	(0.686, 1.15)
Associated Peptides	1
Associated Spectra	1
Coverage	0.00855

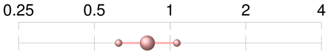

A	2.5	50	97.5	Sequence
1	0.62	0.81	1.1	SAVCTRDTKR

```
1       MAAPRPLAAR AGLRRHRPRP AGPGAARRKG GRLRAASSAK REGDGRARRP DRAPDSPWSS EERSAAASPP LPTASGEMGR
81      GKAGSARTLK GGRGPPSPGL PTDEAGRASR SCSAFFRPLA VSPRRTGASL ARPGARALLL GSQRCAAGRA RRSSRSAVCT
161     RDTKRSPRAV GKSSLSRLGR EEEQRTGKSC CAAFQTRGRF PAGPVAGAAW RPAGGSTAPL RKAPLGAASA RHPPAPPPLP
241     PPPPLRAEAI RAAAVAAVVA APRGAGMADE ARSLLGGSPA SSPVLAGRGR VSSSSSSSSS SSAIPPGHSF RRVTLTKPTF
321     CHYCTDFIWG LAGYLCEVCN FMSHEKCLKN VNIPCSCIAP SHVRVPVAHC FGPPGHYKRK FCTVCRKSLE SPAFRCEVCE
401     LHVHTDCILF VCSDCRQCHQ DGHQDHSVQC MHWYEGNLSS GARCEVCKKT CGSSEVLSGM RCEWCGMLAH AACYVVVTPE
481     CTFGRLRSMI LPPSCVQLFS RNFSKLHCFR ISENLQTESD EGDDVDGSTQ GPAKDVQIST DSSKQTLKIF DGSDGVKRNQ
561     FRLISVPRIA KNEEVVEAAL RAYYINDDQQ EYELQTFTQQ ALLSDDVINR NDAAEDSNLG SVFRESVPEA WIIRAKPKDE
641     EVIRIYPAWL QEGTAYISLR VNRDSTTQTV IKEVLPLLGR QAECLQNFRL VEVLMGGKQV QRMVLDSQKL ILNRLEDIKK
721     ISIRQMNQTR FYIVENSKST VQVNVFVGGL PPQLSPEEYM NILKDELAIK TNVVSLSHVY QAQGAVVLEI SCFSEAERIY
801     MLVKDTTIND KPLNAVVIPE VMASKIPQNC WPLLVFVNPK SGGLKGRDLL YCFRKLLNPH QVFELTNGGP LPGFHTFSKV
881     PSFRVLVCGG DGTVGWVLGA LEEIRHKLVC SEPSVAILPL GTGNDLGRVL RWGAGYSGED PYSILISVDE ADNVLMDRWT
961     ILLDAEEPAE GAENGVAEPE PPKIVQMNNY CGLGIDAELS LDFHHAREEE PGKFNSRFHN KGVYVKVGLQ KISHTRNLHK
1041    DIKLQVDQRE VELPNIEGLI FINIPSWGSG ADLWGTDSDD RFEKPRIDDG LLEVVGVTGV VHMGQVQSGF RSGIRIAQGS
1121    YFRVTLLKPI PVQVDGEPWI QAPGQIIISA AGPKVHMLKK SKQKKKKLEA
```

5.56 intraflagellar transport 140 homolog [Gallus gallus]

Protein Accession	gi|61098290
Mean Expression Ratio	0.882
Median Expression Ratio	0.884
Credible Interval	(0.681, 1.13)
Associated Peptides	1
Associated Spectra	1
Coverage	0.00779

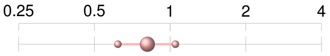

A	2.5	50	97.5	Sequence
1	0.62	0.81	1.1	EAEQEPEQEAR

```
1       MAVYVDHRTE APDSVASPSL ITWHSVHPLL AVASISTASG GCVDIYLEQG EHEPDAHIER SFQVTSLSWH PSRLILAAGW
81      ETGEVVILNK QDKELHTVPP NHNAKITILN WSSNGSRLVS GDRHGILFLW RMDQRGRVQG PPMLKHEYGK CLSHCIFRPP
161     PPGEDFLQLA KAAVSGDEKA LDMFNWRKAG TEAPLKMGLQ EGLSFFITLT DGSVHYVNEK GKTSHVLSAD NLVQKLLFME
241     KRDALVVVTE NLLLSLHTIT LEGDAKELMK VKLGGKTGNS ADIILIDHSL IVTAMGETVL RFWDLDRGEN YVLSPDVQFG
321     FEAGECMNCV SYCSAKGLLA AGTNKGRVAM WRSVSVSSQS TGALEGKEKW KLQASTELEG NITQIKWGSR KNLLAVNSIS
401     SVVILSEQAM SCHYNQQVAA VQVSPSLFNV TFFSTGTTHN LHVDMTVNGV FTTKDAVVFW NGKQVTVFEC SEDTLRNAGS
481     FLCDSPVLSM HGENLYTVEP HRVQVRTWQG TVKQLLVFSE AEGNPCLLDV CGNFLAVGTD LAHFKIFDLS RREAKVHCNS
561     KNFSELFPGL GNIASVKCNA NGNKVSILVS KANGSTDSKI CFYDVEMDKV TLFDFKAERG IGREKLSSGH GIDRSVVEYP
641     DLHNHIPVCH FWDCSEPRLF VCETIPETGV RSPSQKNNQT ESMLDVWVVS FFSTEEHGLL LQDSFPLPSL YEVLLGIDVP
721     HYYFAKKPGE TGKGQAESGS KVLQMASRQP MRDFIGLGDC DKATRDAMLN FSFYLTAGDM DEAFKSIKLI KSEAVWENMA
801     RMCVKTQRLD VAKICLGNMG HARGARALRE AEQEPEQEAR VAVLAVQLGM LEDAERLYKA CKRYDLLNKF YQATNQWQKA
881     IETAEAHDRV YLRTTYYNYA KHLEAVGDHA LALSHYEKSD THRFEVPRML SEDLQALENY VNKMKDKSLW KWWAQYLESQ
961     SDLESALKYY ALAQDYFSLV RVHCFQGNIQ KAAEIANETG NWAASYHLAR QYESQDEIKQ AVHFYTRAQA FNNAIRLCKE
1041    NNLDDQLMNL ALLSSPEDMI EACYYEEKG EQMDRAVMLY HKAGHFSKAL ELAFATQQFG ALQLIAEDLD EKSDPALLAR
1121    CSGFFIEHAQ YEKAVELLLT AKKYHEALQL CLKQNLTITE EMAERMTVSK DSKDLSEESR RELLEQIADC CMRQGNYHMA
1201    TKKYTQAGNK LKAMKALLRS GDTEKIVFFA GVSRQREIYI MAANYLQSLD WRKDPEIMKN IISFYTKGRA LDLLAGFYDV
1281    CAQVEIDEYQ NYEKAQGALT EAYKCLSKAK TKSPLEQESK LAHLQSKMAL IKRFIHARRV YSEDPKEAVR QCELLLAEQD
1361    LDNTIRQGDV LGFLVETLFA GGGIPYGLPV PGTDAEKNSM HKPDLLHQAA DD
```

5.57 PREDICTED: similar to kinesin heavy chain [Gallus gallus]

Protein Accession	gi|50732309
Mean Expression Ratio	1.13
Median Expression Ratio	1.13
Credible Interval	(0.873, 1.47)
Associated Peptides	1
Associated Spectra	1
Coverage	0.0166

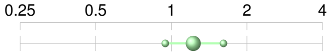

A	2.5	50	97.5	Sequence
**1**	**0.94**	**1.2**	**1.6**	**SAEIDSDDTGGSAAQK**

```
1     MADPAECNIK VMCRFRPLNE SEVTRGDKYV AKFQGEDTVV IASKPYIFDR VFQSNTSQEQ VYNDCAKKIV KDVLEGYNGT
81    IFAYGQTSSG KTHTMEGKLH DPDGMGIIPR IVQDIFNYIY SMDENLEFHI KVSYFEIYLD KIRDLLDVSK TNLSVHEDKN
161   RVPYVKGCTE RFVCSPEEVM DTIDEGKSNR HVAVTNMNEH SSRSHSIFLI NVKQENTQTE QKLSGKLYLV DLAGSEKVSK
241   TGAEGAVLDE AKNINKSLSA LGNVISALAE SSTYVPYRDS KMTRILQDSL GGNCRTTIVI CCSPSSYNES ETKSTLLFGQ
321   RAKTIKNTVC VNVELTAEQW KKKYEKEKEK NKTLRNTIQW LENELNRWRN GETVPVDEQF DKEKANLEAF AVDKDITVIN
401   DKPATTIGVT GNFTDAERRK CEEEIAKLYK QLDDKDEEIN QQSQLVEKLK TQMLDQEELL ASTRRDQDNL QAELNRLQAE
481   NDASKEEVKE VLQALEELAV NYDQKSQEVE DKAKEYELLS DELNQKSVTL ASIDAELQKL KEMTNHQKKR ATEMMASLLK
561   DLAEIGIAVG NNDVKQPEGT GMIDEEFTVA RLYISKMKSE VKTMVKRCKQ LEGTQAESNK KMEENEKELA ACQLRISQHE
641   AKIKSLTEYL QNVEQKKRQL EESVDSLNEE LVQLRAQEKV HEMEKEHLNK VQTANEVKQA VEQQIQSHRE THQKQISSLR
721   DEVDAKEKLI TELQDQNQKM MLEQERLRVE HEKLKATDQE KSRKLHELTV MQDRREQARQ DLKGLEETVA KELQTLHNLR
801   KLFVQDLATR VKKSAEIDSD DTGGSAAQKQ KISFLENNLE QLTKVHKQLV RDNADLRCEL PKLEKRLRAT AERVKALESA
881   LKEAKENASR DRKRYQQEVD RIKEAVRSKN MARRGHSAQI AKPIRPGQHP AASPTHPSAI RGGGAFTQNS QPVALRGGGG
961   RQDKVC
```

5.58 PREDICTED: similar to human P5 isoform 2 [Gallus gallus]

Protein Accession	gi|50745031
Mean Expression Ratio	0.884
Median Expression Ratio	0.884
Credible Interval	(0.693, 1.12)
Associated Peptides	1
Associated Spectra	2
Coverage	0.0246

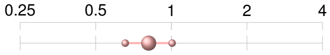

A	2.5	50	97.5	Sequence
**2**	**0.66**	**0.81**	**1**	**GEDPVDYDGGR**

```
1     MGVRGTGGLW WGTVSCTLFL AVNGLYSASD DVIELTPTNF NKEVIQSESL WLVEFYAPWC GHCQRLTPEW KKAATALKGV
81    VKVGAVDADK HQSLGGQYGV RGFPTIKIFG ANKNKAEDYQ GGRTSEAIVD AALSALRSLV KDRLSGRSGG YSSGRQSRES
161   GGGDKKDVIE LTDDSFDKNV INSDDVWMVE FYAPWCGHCK NLEPEWAAAA TEVKEQTKGK VKLAAVDATV NQMLANRYGI
241   RGFPTIKIFQ KGEDPVDYDG GRTRSDITAR ALDLFSDNAP PPELLEIINE DVLKTTCDAH QLCIISVLPH ILDTGASGRN
321   SYLDVMLKMA EKYKKKMWGW LWTEAGAQSD LESSLGIGGF GYPAMAAINA RKMKFALLKG SFSEQGINEF LRELSVGRGS
401   TAPVGGGAFP KIHAVEPWDG KDGELPVEDD IDLSDVDLDD IWDKDEL
```

5.59 PREDICTED: similar to Murr1 [Gallus gallus]

Protein Accession	gi|118087623
Mean Expression Ratio	1.13
Median Expression Ratio	1.13
Credible Interval	(0.896, 1.43)
Associated Peptides	1
Associated Spectra	2
Coverage	0.067

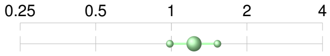

A	2.5	50	97.5	Sequence
**2**	**0.99**	**1.2**	**1.5**	**GQLYPEAAPEEFR**

```
1     MAAAEQQPPA VDGGKALSAL LGGIAQAAYY GKADITEELL RGQLYPEAAP EEFRALRAKM GGLLQSIASA DMDLNQLEAF
81    LTAQTKKQGG ITSDQAAVIA KFWKNHRVKI RESLVNQSCW DNVLKNMNWR VDLKSQSRHV DQINTPVAIV EMELGKYGQE
161   SEFLCLEFDE AKVSQMLKKL TEIEESMTAL TQTT
```

5.60 PREDICTED: similar to gravin [Gallus gallus]

Protein Accession	gi|118088380
Mean Expression Ratio	1.13
Median Expression Ratio	1.13
Credible Interval	(0.964, 1.32)
Associated Peptides	6
Associated Spectra	6
Coverage	0.0564

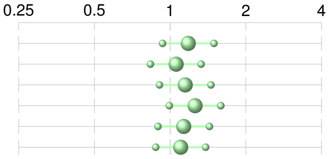

A	2.5	50	97.5	Sequence
1	0.93	1.2	1.5	EDDTQTMETSPSDSSTK
1	0.84	1.1	1.3	TTEEPSPQDSEAVTPR
1	0.9	1.1	1.4	NVTAVTIETQER
1	1	1.3	1.6	EDEQQSPTKEEQEK
1	0.9	1.1	1.4	EQTEDEKQESK
1	0.88	1.1	1.4	SDGKPEPTHLK

```
1     MGAGSSAEPS APPQEKGFAA AAAPPEPAKA LDASSPVPDI NEDNLELEVP PQEPQQPGAV TAPQELTGQQ PEVTSPLQEP
81    PEQHAEALMT SVEQTEPSSI TLKEDDTQTM ETSPSDSSTK DGVAAEKDAH TDKQLPSVEE DAEDAEHASE PQSYDLGFKK
161   VFKFVGFRFT VKKEKTGKSE PVQLLTVKKE TQVTEGADDQ KEASSEEAVM PEDAPSAEDS TKDALKNEKT EDESPKTPEA
241   NEICSQSSAS ATDTASPLRK FFSQGWTGFR KKKSFRKPKE DEQQSPTKEE QEKEGATLTT ETSEKEKSES EKQVEEKNVT
321   AVTIETQERE QTEDEKQESK TETTIGFDAK EEVTEHDEQD VAAAVATASA KEGKAEDQES KLVEVSEDLD KKEEKKGEKE
401   SEVTEKPLTA KSVMPVITTG VNGELKASSE VLPVGKDLES TEKCEINGRT EISSEEKLGA GPLLATESSI EDLKKSEGID
481   GSKPVLLEKE TVDEKTEIAE LEISPTTEVV TKKLETQGEA QDGTTEKKAS KEDEAKLALD AAGPKSFSTS EQSFDTEDDQ
561   QSVKPTDEGL QGKIGTDGIQ PGEITTEITL EEASGKRPPE GITNEAELLS SQEKTKLQGS PLKKLFTGTG LKKLSGKKHK
641   GKREESKLGE QGESTQHLSD SPDSPEEQKA ESSASSPEEL NEIPSLEKSV DGTQVSENED AAVSDVERKR ESVTPWASFK
721   KMVTPKKRVR RPSESDKEEE VDKTKSVATS VTESAAEESQ VELKENGMDQ KPEKVTEEPK RKVDTSVSWE AFICVGSSKK
801   RARKSSSSDE ESEHKLGQES QKIEESGQSK ETATDAILTS SQESDQGQGN SSPEQAGSPS EGEGISTWES FKRLVTPRRK
881   SKTRMEERTE DSVTGSSLEH STSDGEPGKD ESWVPFRKLM PGRRKKKSDG KPEPTHLKQA REDMAETAEE DSDIPAVVPL
961   SEYEAAEQEK IEAQQVKDAE ALKERSSEEE RAGKSEDSLS VKQASEGLVH AVTVSVVEGE RAVTSIEERS PSWISAALTE
1041  CIEQEEEEEK ETEKALESEV VVEEAMVVAK TMPEMRKDVS YDTTASELEL TSEAVTALEE TAEASCAEET MEVSFAEETT
1121  EMVSAVSQLI ETPDTTEEVT PVQEVEATEQ NLKELDKQTQ KVLHEVAERV KSSDVARVDS ERTVTATVIS TVQEIESEVK
1201  DDTKDDKIVG QGTILLEQSL KKGEHLEDDV QPLEGAGSVQ SENGIEERVH EGSERSEISA AVKESAEGYE NVDVLRDEGQ
1281  RRTCEGAVVE DHEEIGEVQR TTEEPSPQDS EAVTPREEQL AKQKSSEQEK LPQRDLTVDE WRDDRIPEVQ AAVQNEIEDE
1361  TSASGFTGEE PAELEGEGGT LPVAPVCTKA AVAEVPVNPE SQDEIPELDS QEQTCIEASI LSALISKVKV TDGTL
```

5.61 PREDICTED: aminopeptidase puromycin sensitive [Gallus gallus]

Protein Accession	gi|118102937
Mean Expression Ratio	1.13
Median Expression Ratio	1.12
Credible Interval	(0.924, 1.37)
Associated Peptides	3
Associated Spectra	3
Coverage	0.0374

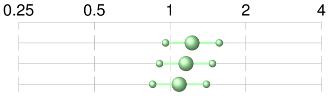

A	2.5	50	97.5	Sequence
1	0.96	1.2	1.6	AGIISTVEVLK
1	0.9	1.2	1.5	YAAVTQFEATDAR
1	0.85	1.1	1.4	FKDHVEGK

```
1     MLLVANYHPE MIIDGGPSQS VKHATNQIVM NCADIDIITA SYAPEGDEEV HATGFNYQNE DEKVTLSFPS TLQKGTGTLK
81    IDFVGELNDK MKGFYRSKYT TPTGDTRYAA VTQFEATDAR RAFPCWDEPA IKATFDISLV VPKDRVALSN MNVIDRKPYP
161   DDENLVEVKF ARTPIMSTYL VAFVVGEYDF VETRSLDGVL VRVYTPVGKA EQGKFALEVA AKTLPFYKDY FNVPYPLPKI
241   DLIAIADFAA GAMENWGLVT YRETALLIDP KNSCSSSRQW VALVVGHELA HQWFGNLVTM EWWTHLWLNE GFASWIEYLC
321   VDHCFPEYDI WTQFVSADYT RAQELDALDN SHPIEVSVGH PSEVDEIFDA ISYSKGASVI RMLHDYIGDE DFRKGMNLYL
401   TKFLQKNAAT EDLWESLEKA SGKPIAAVMN TWTKQMGFPL IYVEAEQQED DKVLKLVQKK FCASGPYAGE DFPMWMVPIS
481   ICTSDDPTSA KMQVLMDKPE LTLVLKDVKP DQWVKLNLGT VGFYRTQYSP DMLESLIPAI KDLSLPPVDR LGLQNDLFSL
561   ARAGIISTVE VLKVMEAFVN EPNYTVWSDL SCNLEILSTL LSHTDFYEEI QVFVKDVFSP IGERLGWDPK PGEGHLDALL
641   RGLVLGKLGK AGHKATLEEA RRRFKDHVEG KNILSADLRS PVYVTILKHG DSTTLDTMLK LHKQADMQEE KNRIERVLGA
721   ISQPELIQKV LTFALSEEVR PQDTVSVIGG VAGGSKQGRK AAWKFVRDNW EELYNRYQGG FLISRLIKLT VDGFANDKMA
801   AEVKAFFESH PAPSAERTVQ QCCENILLNA AWLKRDSEDI HQFFLQRKGP PPATA
```

5.62 PREDICTED: similar to Phosphatidylethanolamine-binding protein 1 (PEBP-1) (HCNPpp) (Basic cytosolic

Protein Accession	gi|50756703
Mean Expression Ratio	0.889
Median Expression Ratio	0.889
Credible Interval	(0.748, 1.06)
Associated Peptides	4
Associated Spectra	5
Coverage	0.369

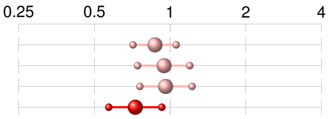

A	2.5	50	97.5	Sequence
2	0.71	0.87	1.1	LYTLVLTDPDAPSR
1	0.74	0.94	1.2	GNDVGSGTVLSDYVGSGPPK
1	0.76	0.96	1.2	YVWLVYEQPK
1	0.57	0.73	0.92	PVELGLWDGPLSLSEVEQKPAHPLR

```
1       MPVELGLWDG PLSLSEVEQK PAHPLRVKYG SVEIDELGKV LTPTQVQHRP TSIEWDGCDP QKLYTLVLTD PDAPSRKDPK
81      FREWHHFLVT NMKGNDVGSG TVLSDYVGSG PPKGTGLHRY VWLVYEQPKQ LTCNEPILSN RSGDKRGKFK VAAFRSKYGL
161     GVPVAGTCYQ AEWDDYVPKL YEQLSGK
```

5.63 PREDICTED: similar to aflatoxin aldehyde reductase [Gallus gallus]

Protein Accession	gi|118101125
Mean Expression Ratio	1.13
Median Expression Ratio	1.12
Credible Interval	(0.872, 1.47)
Associated Peptides	1
Associated Spectra	1
Coverage	0.0337

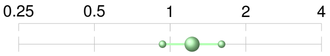

A	2.5	50	97.5	Sequence
1	0.93	1.2	1.6	QVELELFPCLR

```
1       MAAGGARPGV VLGAMEMGRR AGPEASSAML RAFLRRGHRL LDTAYIYAGG ESERILGTLL AGGEHSVEVA TKANPWEGNT
81      LKPDSVRSQL NTSLERLQRT SVELFYLHAP DHGTPVEETL RACNELHKEG KFKELGLSNY AAWEVAEICT ICKCNNWLMP
161     TVYQGMYNAT TRQVELELFP CLRYYGLRFY AYNPLAGGLL TGKYKYEDKD TRQPTGRFFG NDWAQAYRDR YWKKHNFEGI
241     ELVKKALKDA YGSNAPSLTS AALRWLYHHS KLQGSLGDAV IVGMSNMEQL EQNLNYSEEG PLLPPVVEAL DEAWKLTAHD
321     CPNYFR
```

5.64 collagen, type XVIII, alpha 1 [Gallus gallus]

Protein Accession	gi|45383788
Mean Expression Ratio	1.13
Median Expression Ratio	1.12
Credible Interval	(0.876, 1.46)
Associated Peptides	1
Associated Spectra	1
Coverage	0.00818

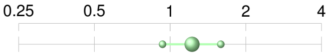

A	2.5	50	97.5	Sequence
1	0.93	1.2	1.6	AGARILSFDGR

```
1       MRPGCAPRRL LLLGLFVLLL LPAASQEPEN LSTEVSLLEL IGDPPPEEIL KIYGPDNNPG YVFGPSANTG QVARYHLPSP
81      FYRDFSLLFH IQPTTPRAGM LFAITDSTQS IIYVGVKLSD LQMGKQQIIF YYTEPGSQSS YAAATFTVPT LLNQWTRFAI
161     SVEEDEVILY LDCEEHERVR FERSPDEMEL EEGSGLFVAQ AGGADPDKYQ GVIADLRLRG DPRAAEHQCE EEEDDAEASG
241     DFGSGAEDRH HPSGKDKGIP GLLDAVPVTS PPVVEGSGTR SSAGSPQQAE RTRVEERLQV STGGTGPKGE KGEKGERGPK
321     GDSGTSGILG TGATKGEKGE KGELGIKGSA GFGYPGSKGQ KGEPGEPGPP GPLSRHTDSM SLEQVTGPPG PTGPPGKDGA
401     PGRDGEPGDP GEDGKPGEMG PQGFPGTPGE SGQKGEKGDP GVGPRGPPGP PGPPGPPGRS SKQDGLTFID MEGSGFGGDL
481     ETLRGPRGPP GPPGPPGVPG LPGEPGRFGM NSTDLPGPPG LPGRDGTPGA PGPEGPLGPP GKDGMPGPPG PKGERGDVGD
561     LGLPGAPGPK GSKGEAGPAG PPGETGLAGL PGPVGPRGPP GPPGPPGPPG PGYEAGFGDM EGSGLPLATG SPGPRGPSGP
641     QGVPGLPGIK GEVGSLGQPG PPGPKGDAGV PGVDGRPGLE GFPGPQGPKG NRGSPGEKGE RGQDGVGLPG PPGPPGPPGQ
721     VIYMSSEDRP LVALPGPEGR SGHAGFPGPV GPKGDPGSPG IQGAPGMKGE KGEPGVIISP DGTIVAANVK GQKGEPGLPG
801     PMGPSGPHGR AGMKGEIGFP GRPGRPGMNG LKGEKGDPVD ISSVLSLRGP PGPPGPPGPP GPPGSVVYDS NNGFSDASRP
```

```
881     AFPGFHQFPG QKGEKGDVGA PGPPGQFPYD LSRFSASLRG DKGEAGPKGE KGEPGSSTLY GPSVTGPPGP QGYPGPPGPK
961     GDSIVGPPGP PGPQGPPGIG YEGRQGPPGP PGPPGPPSFP GPHRQAISNP GPPGPPGPPG PPGTSGTSLG LRTMPTYQAM
1041    LSAAHELPEG GLIFLADRQE LYIRLRGGFR RVLLEEHTLV PSSALDNEVY DKLPSIHYGG AQQPVHPLRN HNPPPTARPW
1121    RGDEVVANQH HLPQPPLLQQ HELLNSYYIH RWPDPAPVAA HVHQDFQPAL HLVALNTPLS GGMRGIRGAD FQCFQQARQV
1201    GLAGTFRAFL SSRLQDLYSI VRRADRTAVP IVNLRDEVLF SNWEALFTGS EAPLRAGARI LSFDGRDILQ DSAWPQKSIW
1281    HGSDAKGRRL PESYCEAWRT DERGTSGQAS SLSSGKLLEQ SASSCQHAFV VLCIENSFMT AAKK
```

5.65 PREDICTED: similar to D4-GDP-dissociation inhibitor [Gallus gallus]

Protein Accession	gi|50728568
Mean Expression Ratio	0.89
Median Expression Ratio	0.89
Credible Interval	(0.712, 1.11)
Associated Peptides	2
Associated Spectra	2
Coverage	0.15

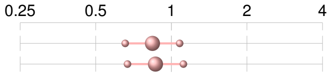

A	2.5	50	97.5	Sequence
1	0.65	0.84	1.1	SLLGDGPVVADPTAPNVVVTR
1	0.67	0.87	1.1	LNYKPPPQK

```
1       MTEKTQEPHV EEDDDELDGK LNYKPPPQKT LQELQELDKD DESLTKYKKS LLGDGPVVAD PTAPNVVVTR LTLVCDSAPG
81      PITMDLTGDL EALKKETFVL KEGVEYRVKI HFKVNRDIVS GLKYVQHTYR TGVKVDKATF MVGSYGPRPE EYEFLTPIEE
161     APKGMLARGT YHNKSFFTDD DKHDHLTWEW NLSIKKEWTE
```

5.66 hemoglobin, zeta [Gallus gallus]

Protein Accession	gi|52138651
Mean Expression Ratio	0.89
Median Expression Ratio	0.89
Credible Interval	(0.727, 1.09)
Associated Peptides	2
Associated Spectra	5
Coverage	0.218

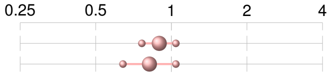

A	2.5	50	97.5	Sequence
4	0.76	0.9	1.0	VATQIESIGLESLER
1	0.64	0.82	1.0	TYFPHFDVSQGSVQLR

```
1       MALTQAEKAA VTTIWAKVAT QIESIGLESL ERLFASYPQT KTYFPHFDVS QGSVQLRGHG SKVLNAIGEA VKNIDDIRGA
81      LAKLSELHAY ILRVDPVNFK LLSHCILCSV AARYPSDFTP EVHAAWDKFL SSISSVLTEK YR
```

5.67 heat shock 10kDa protein 1 (chaperonin 10) [Gallus gallus]

Protein Accession	gi|45384204
Mean Expression Ratio	1.12
Median Expression Ratio	1.12
Credible Interval	(0.868, 1.45)
Associated Peptides	1
Associated Spectra	1
Coverage	0.0784

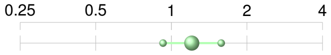

A	2.5	50	97.5	Sequence
1	0.92	1.2	1.6	KFLPLFDR

```
1       MAGKAFRKFL PLFDRVLVER CAAETVTKGG IMIPEKAQGK VLQATVVAVG SGARGKDGEI HPVSVKVGEK VLLPEYGGTK
81      IVLEDKDYYL FRDGDILGKY LD
```

5.68 PREDICTED: hypothetical protein [Gallus gallus]

Protein Accession	gi|118081867
Mean Expression Ratio	1.12
Median Expression Ratio	1.12
Credible Interval	(0.872, 1.45)
Associated Peptides	1
Associated Spectra	1
Coverage	0.055

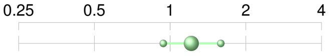

A	2.5	50	97.5	Sequence
**1**	**0.94**	**1.2**	**1.6**	**AMEPAAGGGPGAAXVAR**

```
1       MVRAAAAARR RRERSGARGG RRRAMEPAAG GGPGAAXVAR ARAAPRRSRS ARAGFPQTQA PVHEYAPHAH PAPAARPPNG
81      KLYSARAPAV SSSWWKSYAP QGEREIQLNR GEAVKVLSIG EGGFWEGTVK GRTGWFPAEC VEEVQMRQYD PRQETREDRT
161     KRLFRHYTVG SYDNFTSHSD YIIEEKTAVL QKREHEGFGF VLRGAKGLVQ DLLLLRALLN VVEHQSSTRV NGVNVVKVGH
241     KQVVSLIRQG GNHLVMKVVS VSRKPESEEV VRKKAACQRA VWAAEGHSEC REEADPTLSA AAASSLHRV
```

5.69 coatomer protein complex, subunit gamma [Gallus gallus]

Protein Accession	gi|71895411
Mean Expression Ratio	1.12
Median Expression Ratio	1.12
Credible Interval	(0.862, 1.45)
Associated Peptides	1
Associated Spectra	1
Coverage	0.0114

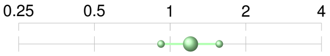

A	2.5	50	97.5	Sequence
**1**	**0.92**	**1.2**	**1.6**	**DDNYRGPAVR**

```
1       MLKKFDKKDE ESGGGSNPFQ HLEKSAVLQE ARVFNETPIN PRKCAHILTK ILYLINQGEH LGVMEATESF FAMTKLFQSN
81      DPTLRRMCYL TIKEMSSIAE DVIIVTSSLT KDMTGKDDNY RGPAVRALCQ ITDSTMLQAI ERYMKQAIVD KVPSVSSSAL
161     VSSLHLLKTS FDVVKRWVNE AQEAASSDNI MVQYHALGLL YHVRKNDRLA VNKMLSKFTR HGLKSPFAYC MMIRVASKLL
241     EEEAGSRDSP LFDFIESCLR NKHEMVVYEA ASAIVNLPNC TAKELAPAVS VLQLFCSSPK AALRYAAVRT LNKVAMKHPS
321     AVTACNLDLE NLVTDSNRSI ATLAITTLLK TGSESSIDRL MKQISSFMSE ISDEFKVVVV QAINALCQKY PRKHAVLMNF
401     LFTMLREEGG FEYKRAIVDC IISIIEENSE SKETGLSHLC EFIEDCEFTV LATRILHLLG QEGPKTNNPS KYIRFIYNRV
481     VLEHEEVRAG AVSALAKFGA QNEEMLPSIL VLLKRCVMDD DNEVRDRATF YLNVLEQKQK ALNAGYILNG LTVSIPGLER
561     ALHQYTLEPS EKPFDLKSVP LATAPIIEQR AENAPVAVAK QPEKVAATRQ EIFQEQLGAI PEFRGLGSLF KSSPEPVALT
641     ELETEYVVRC TKHTFVSHMV FQFDCTNTLN DQILENVTVQ MEPTEGYEVI GYIPAKSLVY NQPGTCYTLV ALSEEDPTAV
721     ACTFSCMMKF TVKDCDPNTG ETDDEGYEDE YVLEDLEVTV ADHIQRVLKP NFGAAWDEVG DEYEKEETFT LSAIKTLEEA
801     VSNIVKFLGM QPCERSDKVP DNKNSHTLYL AGVFRGGHDV LLRARLLLTD TVTMQVTARS AEELPVDVIM ASVG
```

5.70 hypothetical protein LOC431056 [Gallus gallus]

Protein Accession	gi|71896205
Mean Expression Ratio	1.12
Median Expression Ratio	1.12
Credible Interval	(0.95, 1.31)
Associated Peptides	5
Associated Spectra	6
Coverage	0.108

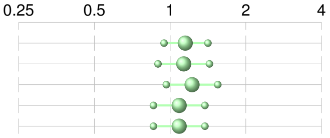

A	2.5	50	97.5	Sequence
**2**	**0.94**	**1.2**	**1.4**	**VANPVVEMDGDEMTR**
**1**	**0.9**	**1.1**	**1.4**	**IIWAFIK**
**1**	**0.97**	**1.2**	**1.5**	**FKDIFQEIFDK**
**1**	**0.86**	**1.1**	**1.4**	**KYSVAVK**
**1**	**0.86**	**1.1**	**1.4**	**YFDLGLPHR**

```
1       MAARCLRAVP ALSRAAGLSR CTPPAACTAL GPPGQRRHYA DRRIKVANPV VEMDGDEMTR IIWAFIKEKL ILPNVDVQLK
81      YFDLGLPHRD KTDDQVTIDS ALATKKYSVA VKCATITPDE ARVEEFKLKK MWKSPNGTIR NILGGTVFRE PIICKNIPRL
161     VPGWTQPITI GRHAHGDQYK ATDFVVNKSG TFKMVFTPKD GSGVKEWEVF NFPGGGVGMG MYNTDESISG FAHSCFQYAI
241     QKKWPLYMST KNTILKAYDG RFKDIFQEIF DKHYKTEFDK LKIWYEHRLI DDMVAQVLKS SGGFVWACKN YDGDVQSDIL
321     AQGFGSLGLM TSVLVCPDGK TIEAEAAHGT VTRHYREHQK GRPTSTNPIA SIFAWTRGLE HRGKLDSNPE LIKFAQTLEK
401     VCVETVESGT MTKDLAGCIH GLANVKLNEH FVNTTDFLDA IKNTLDKALG KK
```

5.71 PREDICTED: similar to candidate tumor suppressor protein DICE1 [Gallus gallus]

Protein Accession	gi|118084887
Mean Expression Ratio	0.894
Median Expression Ratio	0.895
Credible Interval	(0.719, 1.11)
Associated Peptides	2
Associated Spectra	2
Coverage	0.0225

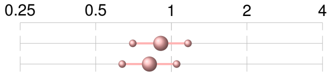

A	2.5	50	97.5	Sequence
1	0.7	0.9	1.2	THLGTTYLDIAK
1	0.64	0.82	1.1	AKIMKEIR

```
1     MPILLFLIDT SASMNQRTHL GTTYLDIAKG AVETFMKLRA RDPASRGDRY MLVTFEEPPY AIKAGWKENH ATFMNELKNL
81    QAEGLTTLGQ SLRTAFDLLN LNRLVTGIDN YGQGRNPFFL EPAIIITVTD GSKLTTTSGI QEELHLPLNS PLPGSELTKE
161   PFRWDQRLFA LVLRLPGVSS PESEQMTGVP VDDSAITPMC EVTGGRSYCV CSPRMLNQCL ESLVQKVQSG VVINFEKAGP
241   DPSPIDDGQV DITRPFGPQP WHSCHKLIYV RPNPKTGVPI GHWPVPESFW PDQNSPTLPP RTSHPVVKFS CTDCEPMVID
321   KLPFDKYELE PSPLTQFILE RKSPQTCWQV YVSNSAKYSE LGHPFGYLKA STALNCVNLF VMPYNYPVLL PLLDDLFKVH
401   KAKPTLKWLQ SFESYLKTMP PYYLGPLKKA VRMMGAPNLI ADNVEYGLSY SVISYLKKLS QQAKIESDRV IGSVGKKVAQ
481   ETGIKVRSRS HNLSMAHRND FQHLLQGITG EIPHRPLDFN MKEYAGFQIA LLNKDLKPQT FRNAYDIPRR NLLDQLTRMR
561   SNLLKSTRKL LKGQDEDQVH SVPIAQMGNY QEYLKQIPSP LRELDPDQPR RLHTFGNPFK LDKKGMMIDE ADEFVSGPQN
641   KHKRPGEPSM QGIPKRRRCM SPLLRGRPQT PPVVNNHIGG KGPPSPITQA QLDLVKPIPI HKTSEANNEI AMDDVVENHV
721   IDPLSSDDFP DAVDSEFSMS SSPFNSLDRS ATHIEGAGHE HLGNNLNVDG FLENHDESSS KEQNTEDSLP ISSTSKGKKT
801   VPCRSSREIN IELRAKIMKE IRKPGRKYER IFSLLKHVQG SLQTRLIFLQ NVIKEASRFK KRMLIEQLES FLEEIHRRSN
881   QVNHINSS
```

5.72 PREDICTED: hypothetical protein [Gallus gallus]

Protein Accession	gi|118091296
Mean Expression Ratio	1.12
Median Expression Ratio	1.12
Credible Interval	(0.866, 1.44)
Associated Peptides	1
Associated Spectra	1
Coverage	0.0942

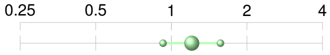

A	2.5	50	97.5	Sequence
1	0.93	1.2	1.6	ILDSVGIETDDER

```
1     MRYVAAYLLA VLGGNESPTS KDLKKILDSV GIETDDERLN KVVVPSGFRA YIPVGVEHWS CSLVFSVSLF PLLLAWYYFS
81    LIIYICDFQN AAEPIATILY SIFIEVWLHL CQLLLPSDLN PKNSKDNGMM VTDNIYCK
```

5.73 myosin, heavy polypeptide 10, non-muscle [Gallus gallus]

Protein Accession	gi|45382679
Mean Expression Ratio	0.895
Median Expression Ratio	0.895
Credible Interval	(0.763, 1.05)
Associated Peptides	6
Associated Spectra	6
Coverage	0.0419

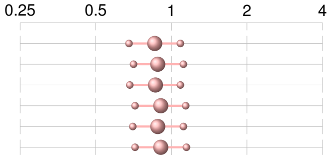

A	2.5	50	97.5	Sequence
1	0.68	0.86	1.1	GSDVNEAQPTPAE
1	0.7	0.88	1.1	ELDDATEANEGLSR
1	0.68	0.86	1.1	QLEEAEEEATR
1	0.72	0.9	1.1	GLEAEILQLQEEFAASER
1	0.7	0.88	1.1	KQELEEILHDLESR
1	0.72	0.9	1.1	KVIQYLAHVASSHK

```
1      MAQRSGQEDP ERYLFVDRAV IYNPATQADW TAKKLVWIPS ERHGFEAASI KEERGDEVLV ELAENGKKAL VNKDDIQKMN
81     PPKFSKVEDM AELTCLNEAS VLHNLKDRYY SGLIYTYSGL FCVVINPYKN LPIYSENIIE MYRGKKRHEM PPHIYAISES
161    AYRCMLQDRE DQSILCTGES GAGKTENTKK VIQYLAHVAS SHKGRKDHNI PPESPKPVKH QGELERQLLQ ANPILESFGN
241    AKTVKNDNSS RFGKFIRINF DVTGYIVGAN IETYLLEKSR AVRQAKDERT FHIFYQLLAG AGEHLKSDLL LEGFNNYRFL
321    SNGYIPIPGQ QDKDNFQETM EAMHIMGFSH DEILSMLKVV SSVLQFGNIS FKKERNTDQA SMPENTVAQK LCHLLGMNVM
401    EFTRAILTPR IKVGRDYVQK AQTKEQADFA VEALAKATYE RLFRWLVHRI NKALDRTKRQ GASFIGILDI AGFEIFELNS
481    FEQLCINYTN EKLQQLFNHT MFILEQEEYQ REGIEWNFID FGLDLQPCID LIERPANPPG VLALLDEECW FPKATDKTFV
561    EKLVQEQGTH SKFQKPRQLK DKADFCIIHY AGKVDYKADE WLMKNMDPLN DNVATLLHQS SDKFVAELWK DEIQNIQRAC
641    FYDNITGLHD PPVDRIVGLD QVTGITETAF GSAYKTKKGM FRTVGQLYKE SLTKLMATLR NTNPNFVRCI IPNHEKRAGK
721    LDPHLVLDQL RCNGVLEGIR ICRQGFPNRI VFQEFRQRYE ILTPNAIPKG FMDGKQACER MIRALELDPN LYRIGQSKIF
801    FRAGVLAHLE EERDLKITDI IIFFQAVCRG YLARKAFAKK QQQLSALKIL QRNCAAYLKL RHWQWWRVFT KVKPLLQVTR
881    QEEELQAKDE ELMKVKEKQT KVEAELEEME RKHQQLLEEK NILAEQLQAE TELFAEAEEM RARLAAKKQE LEEILHDLES
961    RVEEEEERNQ ILQNEKKKMQ GHIQDLEEQL DEEEGARQKL QLEKVTAEAK IKKMEEEILL LEDQNSKFLK EKKLMEDRIA
1041   ECTSQLAEEE EKAKNLAKLK NKQEMMITDL EERLKKEEKT RQELEKAKRK LDGETTDLQD QIAELQAQIE ELKIQLAKKE
1121   EELQAALARG DEEAVQKNNA LKVIRELQAQ IAELQEDLES EKASRNKAEK QKRDLSEELE ALKTELEDTL DTTAAQQELR
1201   TKREQEVAEL KKAIEEETKN HEAQIQEIRQ RHATALEELS EQLEQAKRFK ANLEKNKQGL ESDNKELACE VKVLQQVKAE
1281   SEHKRKKLDA QVQELTAKVT EGERLRVELA EKANKLQNEL DNVSSLLEEA EKKGIKFAKD AASLESQLQD TQELLQEETR
1361   QKLNLSSRIR QLEEEKNNLQ EQQEEEEEAR KNLEKQMLAL QAQLAEAKKK VDDDLGTIEG LEENKKKLLK DMESLSQRLE
1441   EKAMAYDKLE KTKNRLQQEL DDLMVDLDHQ RQIVSNLEKK QKKFDQMLAE EKNISARYAE ERDRAEAEAR EKETKALSLA
1521   RALEEALEAK EEFERQNKQL RADMEDLMSS KDDVGKNVHE LEKSKRTLEQ QVEEMRTQLE ELEDELQATE DAKLRLEVNM
1601   QAMKAQFERD LQARDEQNEE KKRMLVKQVR ELEAELEDER KQRALAVAAK KKMEMDLKDL EGQIEAANKA RDEAIKQLRK
1681   LQAQMKDYQR ELEEARASRD EIFAQSKESE KKLKGLEAEI LQLQEEFAAS ERARRHAEQE RDELADEIAN SASGKSALLD
1761   EKRRLEARIA QLEEELEEEQ SNMELLNERF RKTTLQVDTL NSELAGERSA AQKSENARQQ LERQNKELKA KLQELEGSVK
1841   SKFKATISTL EAKIAQLEEQ LEQEAKERAA ANKLVRRTEK KLKEVFMQVE DERRHADQYK EQMEKANARM KQLKRQLEEA
1921   EEEATRANAS RRKLQRELDD ATEANEGLSR EVSTLKNRLR RGGPITFSSS RSGRRQLHIE GASLELSDDD AESKGSDVNE
2001   AQPTPAE
```

5.74 PREDICTED: hypothetical protein [Gallus gallus]

Protein Accession	gi|50753780
Mean Expression Ratio	0.896
Median Expression Ratio	0.896
Credible Interval	(0.718, 1.12)
Associated Peptides	2
Associated Spectra	2
Coverage	0.214

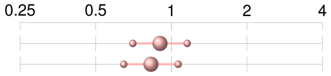

A	2.5	50	97.5	Sequence
1	0.7	0.9	1.2	NTILAQVLDQAAR
1	0.64	0.83	1.1	HGDTSADPSQQEAK

```
1      MADEELAALR QQRLAELQAK HGDTSADPSQ QEAKQREAEI RNTILAQVLD QAARARLSNL ALVKPDKAKA VENYLIQMAR
81     FGQLPGKVSE QGLIEILEKV SQQTEKKTTV KFNRRKVLDS DEEDDY
```

5.75 PREDICTED: hypothetical protein [Gallus gallus]

Protein Accession	gi|118083694
Mean Expression Ratio	0.895
Median Expression Ratio	0.896
Credible Interval	(0.69, 1.16)
Associated Peptides	1
Associated Spectra	1
Coverage	0.0548

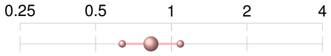

A	2.5	50	97.5	Sequence
1	0.64	0.83	1.1	KQTASDEKPPSR

```
1      MSAFPAFKVR TRGSIKRRPP SRKFRRSLSD YGDGEDLGNI IPSPENGTKE DGDEVFGHQG KTEEAETGNK EIKKQTASDE
81     KPPSRRGSSR SENEEKEEKQ AEEVTPCKSN AGDTEKEEEE VRCSAPGEEN STQSPSEDKE DKVCEASQEK EEEGGACEQE
161    KGDKEKEEGE EKETSESTVT RRMSDTAETP LAPQLLQEAV GLETASEPLK PPVQDQGTE
```

5.76 PREDICTED: similar to Cullin-4B (CUL-4B) [Gallus gallus]

Protein Accession	gi|118089688
Mean Expression Ratio	1.12
Median Expression Ratio	1.12
Credible Interval	(0.856, 1.44)
Associated Peptides	1
Associated Spectra	1
Coverage	0.0125

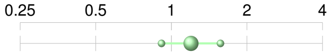

A	2.5	50	97.5	Sequence
**1**	**0.91**	**1.2**	**1.6**	**TIDGILLLIER**

```
1     MITSGFSSPN PSTAAQEVRP ATDGSSGSSS SCKKRKLNSS NSEREEFDSI SSCSSSPSKN NSSSSSLVIT TSSCSSSGVA
81    SSNHHLQKKL RFEDSLDFIG LDVKMAEESS SSSSPAASSQ QQHQQQLKNK SLLISSVAVG HHANGLTKAA STVSSFANSK
161   PGSAKKLVIK NFKDKPKLPE NYTDETWQKL KEAVEAIQNS TSIKYNLEEL YQAVENLCSY KISANLYKQL RQICEDHIKA
241   QIHQFREDSL DSVLFLKKID KCWQDHCRQM IMIRSIFLFL DRTYVLQNSM LPSIWDMGLE LFRTHIISDQ KVQNKTIDGI
321   LLLIERERNG EAIDRSLLRS LLSMLSDLQI YQDSFEHRFL EETNRLYAAE GQRLMQEREV PEYLHHVNKR LEEEADRIIT
401   YLDQSTQKPL IATVEKQLLG EHLTAILQKG LNHLLDENRI QDLSLLYQLF SRVRGGVQVL LQHWIEYIKA FGSTIVINPE
481   KDKTMVQELL DFKDKVDHII DVCFLKNEKF VNAMKEAFET FINKRPNKPA ELIAKYVDSK LRAGNKEATD EELEKMLDKI
561   MIIFRFIYGK DVFEAFYKKD LAKRLLVGKS ASVDAEKSML SKLKHECGAA FTSKLEGMFK DMELSKDIMI QFKQYMQNQN
641   VPGNIELTVN ILTMGYWPTY VPMEVHLPPE MVKLQEIFKT FYLGKHSGRK LQWQSTLGHC VLKAEFKEGK KELQVSLFQT
721   LVLLMFNEGE EFSLEEIKQA TGIEDGELRR TLQSLACGKA RVLTKSPKGK DVEDGDKFTC NDDFRHKLFR IKINQIQMKE
801   TVEEQASTTE RVFQDRQYQI DAAIVRIMKM RKTLSHNLLV SEVYNQLKFP VKPADLKKRI ESLIDRDYME RDKENPNQYN
881   YIA
```

5.77 PREDICTED: similar to stanniocalcin [Gallus gallus]

Protein Accession	gi|118101318
Mean Expression Ratio	0.896
Median Expression Ratio	0.896
Credible Interval	(0.697, 1.15)
Associated Peptides	1
Associated Spectra	1
Coverage	0.0283

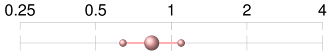

A	2.5	50	97.5	Sequence
**1**	**0.64**	**0.83**	**1.1**	**RCSTFQR**

```
1     MLLKSGLLLL LVISASASYE AEQNDSVSPR KPRVAAQNSA EVVRCLNSAL QVGCGAFACL ENSTCDTDGM YDICKSFLYS
81    AAKFDTQGKA FVKESLKCIA NGVTSKVFLA IRRCSTFQRM ISEVQEECYS KLDMCGIAKR NPEAITEVVQ LPNQFSNRYY
161   NKLVRSLLEC DEETVSTIKD SLMEKIGPNM ASLFHLLQTD HCAQGHPRTD FARRRITEPQ KLKLYFRNLR GEGSIPAHAK
241   RTSAESA
```

5.78 PREDICTED: similar to thioredoxin-like protein [Gallus gallus]

Protein Accession	gi|50806608
Mean Expression Ratio	0.896
Median Expression Ratio	0.898
Credible Interval	(0.692, 1.16)
Associated Peptides	1
Associated Spectra	1
Coverage	0.0623

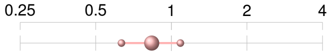

A	2.5	50	97.5	Sequence
**1**	**0.63**	**0.83**	**1.1**	**LIANDTEFQPELSAAGSR**

```
1     MVGVKLIAND TEFQPELSAA GSRLAVVKFT MRGCGPCLRI APAFNALSNK YPQATFLEVD VHQCQGTAAT NNISATPTFL
81    FFRNKVRIDQ YQGADAVGLE EKIKQHLEND PGNNEDTDIP KGYMDLMPFI NKAGCECLNE SDEHGFDNCL RKDSTYLESD
161   CDEQLLITVA FSQPVKLYSM KLQGPDNGQG PKYIKIFINL PRSMDFEEAE RSEPTQALEL TPDDIKEDGI IQLRYVKFQN
241   VNSVTLFVQS NHGDEETTRI TYFTFIGTPV QATNMNDFKR VVGKKGESH
```

5.79 PREDICTED: similar to dynein, cytoplasmic, heavy polypeptide 1 [Gallus gallus]

Protein Accession	gi|118092179
Mean Expression Ratio	1.11
Median Expression Ratio	1.11
Credible Interval	(0.91, 1.36)
Associated Peptides	3
Associated Spectra	3
Coverage	0.00758

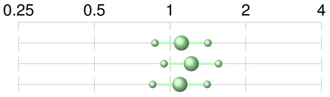

A	2.5	50	97.5	Sequence
1	0.87	1.1	1.4	DYIPVDQEELR
1	0.95	1.2	1.6	SVLVSAGNVKR
1	0.86	1.1	1.4	VKDDIESLHDKFK

```
1       MEVSGSAVFF LADVTVLHKH LHKLVPLLLE DGGKATSVLE ANLKEKGTMK QMQDVGDEGE EEKEFISYSI SNDIHYGIKS
81      NSLAFIKRTP VIDADKPVSL QLRVLTLSED SPYETLHSFI SSAVAPFFKS YIRESGKADR DGDKMAPSVE KKIAELEMGL
161     LHLQQNIEIP EISLPIHATI TNVAKQCYER GEKPKVTDFG EKAEDPLFLN QLQSGVNRWI REIQKVTKLD RDPASGTALQ
241     EISFWLNLER ALYRIQEKRE SPEVLLTLDI LKHGKRFHAT VSFDTDTGLK QALETVNDYN PLMKDFPLND LLSATELDKI
321     RQALVAIFTH LRKIRNTKYP IQRALRLVEA ISRDLSSQLL KVLGTRKLMH VAYEEFEKVM IACFEVFQTW DDEYEKLQVL
401     LRDIVKRKRE ENLKMVWRIS PAHRKLQSRL DQMRKFRRQH EQLRAVIVRV LRPQVTAVAQ QNQGDVPEPQ DMKVAEVLFD
481     AADANAIEEV NLAYENVKEV DGLDVSKEGT EAWEAAMKRY DERIDRVETR ITARLRDQLG TAKNANEMFR IFSRFNALFV
561     RPHIRGAIRE YQTQLIQRVK DDIESLHDKF KVQYPQSQAC KMSHVRDLPP VSGSIIWAKQ IDRQLTAYMK RVEDVLGKGW
641     ENHVEGQKLK QDGDSFRMKL NTQEIFDDWA RKVQQRNLGV SGRIFTIEST RVRGRSGNVL KLKVNFLPEI ITLSKEVRNL
721     KWLGFRVPLA IVNKAHQANQ LYPFAISLIE SVRTYERTCE KVEERNTISL LVAGLKKEVQ ALIAEGIALV WESYKLDPYV
801     QRLAETVFSF QEKVDDLLII EEKIDLEVRS LETCMYDHKT FSEILNRVQK AVDDLNLHSY SNLPIWVNKL DMEIERILGV
881     RLQAGLRAWT QVLLGQADDK AEVDMDTDAP QVSHKPGGEP KIKNIVHELR ITNQVIYLNP PIEECRYKLY QELFSWQTVI
961     LSLPRIQSQR YQVGVHYELS EEEKFYRNGL TRMPDGLAAL EEAYCAVEGI VTEVEQYVKV WLQYQCLWDM QAENIYNRLG
1041    EDLNKWQALL VQIRKARGTF DNAETRKEFG PVVIDYGKVQ SKVNLKYDSW HKEVLSKFGQ MLGQNMTEFH SQISKSRQEL
1121    EQHSVDTAST SDAVTFITYV QSLKRKIKQF EKQVELYRNG QRLLEKQRFQ FPSSWLYIDN IEGEWGAFND IMRRKDSAIQ
1201    QQVANLQMKI VQEDRAVESR TTDLLADWEK TKPVTGNLRP EEALQALTIY EGKFGRLKDD REKCAKAKEA LELTDTGLLS
1281    GSEERVQVAL EELQDLKGVW SELSKVWEQI DQMKEQPWVS VQPRKLRQNL DVLLNQLKNF PARLRQYASY EYVQRLLKGY
1361    MKINMLVIEL KSEALKDRHW KQLMKRLHVN WVVSELTLGQ IWDVDLQRNE AIVKDVLLVA QGEMALEEFL KQASINSSVY
1441    AHNIKSEPFC RIREVWNTYE LDLVNYQNKC RLIRGWDDLF NKVKEHINSV SAMKLSPYYK VFEEDALSWE DKLNRIMALF
1521    DVWIDVQRRW VYLEGIFTGS ADIKHLLPVE TQRFQRVAAT IREKTLEISW RKVREFTLKH CYFTRSISTE FLALMKKVSK
1601    SPLVMDVLNI QGVQRSLERL ADLLGKIQKA LGEYLERERS SFPRFYFVGD EDLLEIIGNS KNVAKLQKHF KKMFAGVSSI
1681    ILNEDNSVVL GISSREGEEV LFKTPVSITE HPKINEWLTL VEKEMRVTLA KLLAESVTEV EIFGKATSID PAVYISWIDK
1761    FQAQLVVLSA QIAWSENVES ALNGMSGGED SSPLQSVLAN VEVTLNVLAD SVLMEQPPLR RRKLEHLITE LVHQRDVTRS
1841    LLRSKVDNSK SFEWLSQMRF YFDPKQTDVL QQLSIQMANA KFNYGFEYLG VQDKLVQTPL TDRCYLTMTQ ALEARLGGSP
1921    FGPAGTGKTE SVKALGHQLG RFVLVFNCDE TFDFQAMGRI FVGLCQVGAW GCFDEFNRLE ERMLSAVSQQ VQCIQEALRE
2001    HSNPNYDKTA TPITCELLNK QVKVSPDMAI FITMNPGYAG RSNLPDNLKK LFRSLAMTKP DRQLIAQVML YSQGFRTAEV
2081    LANKIVPFFK LCDEQLSSQS HYDFGLRALK SVLVSAGNVK RERIQKIKRE KEERGEVVDE GEIAENLPEQ EILIQSVCET
2161    MVPKLVAEDI PLLFSLLSDV FPGVQYHRGE MTALREELKK VCQEMYLTYG DGEEVGGMWV EKVLQLYQIT QINHGLMMVG
2241    PSGSGKSMAW RVLLKALERL EGVEGVAHII DPKAISKDHL YGTLDPNTRE WTDGLFTHVL RKIIDNVRGE LQKRQWIIFD
2321    GDVDPEWVEN LNSVLDDNKL LTLPNGERLS LPPNVRIMFE VQDLKYATLA TVSRCGMVWF SEDVLSTDMI FNNFLARLKS
2401    IPLDEGEEEA QRRRKGKEDE GEEAASPMLQ IQRDAATIMQ PYFTSNGLVT KALEHAFKLE HIMDLTRLRC LGSLFSMLHQ
2481    ACRNVAQYNA NHPDFPMQID QLERYIQRYL VYAILWSLSG DSRLKMRAEL GEYIRRITAV PLPTAPNIPI IDYEVSITGE
2561    WVPWQTKVPQ IEVETHKVAA PDVVVPTLDT VRHEALLYTW LAEHKPLVLC GPPGSGKTMT LFSALRALPD MEVVGLNFSS
2641    ATTPELLLKT FDHYCEYRRT PNGVVLAPVQ LGKWLVLFCD EINLPDMDKY GTQRVISFIR QMVEHGGFYR TSDQTWVKLE
2721    RIQFVGACNP PTDPGRKPLS HRFLRHVPVV YVDYPGPASL TQIYGTFNRA MLRLIPSLRT YAEPLTAAMV EFYTMSQERF
2801    TQDTQPHYIY SPREMTRWVR GIFEALRPLE TLPVEGLIRI WAHEALRLFQ DRLVEDEERR WTDENIDMVA LKHFPNIDKE
2881    KAMSRPILYS NWLSKDYIPV DQEELRDYVK ARLKVFYEEE LDVPLVLFNE VLDHVLRIDR IFRQPQGHLL LIGVSGAGKT
2961    TLSRFVAWMN GLSVYQIKVH RKYTGEDFDE DLRTVLRRSG CKNEKIAFIM DESNVLDSGF LERMNTLLAN GEVPGLFEGD
3041    EYATLMTQCK EGAQKEGLML DSHEELYKWF TSQVIRNLHV VFTMNPSSEG LKDRAATSPA LFNRCVLNWF GDWSTEALYQ
3121    VGKEFTSKMD LEKPNYIVPD YMPVVYDKLP QPPSHREAIV NSCVFVHQTL HQANARLAKR GGRTMAITPR HYLDFINHYA
3201    NLFNEKRSEL EEQQMHLNVG LRKIKETVDQ VEELRRDLRI KSQELEVKNA AANDKLKKMV KDQQEAEKKK VMSQEIQEQL
3281    HKQQEVIADK QMSVKEDLDK VEPAVIEAQN AVKSIKKQHL VEVRSMANPP AAVKLALESI CLLLGESTTD WKQIRSIIMR
3361    ENFIPTIVNF SAEEISDAIR EKMKKNYLSN PSYNYEIVNR ASLACGPMVK WAIAQLNYAD MLKRVEPLRN ELQKLEDDAK
3441    DNQQKANEVE QMIRDLEASI ARYKEEYAVL ISEAQAIKAD LAAVEAKVNR STALLKSLSA ERERWEKTSE TFKNQMSTIA
3521    GDCLLSGAFI AYAGYFDQQM RQNLFTTWSH HLQQANIQFR TDIARTEYLS NADERLRWQA SSLPADDLCT ENAIMLKRFN
3601    RYPLIIDPSG QATEFIMNEY KDRKITRTSF LDDAFRKNLE SALRFGNPLL VQDVESYDPV LNPVLNREVR RTGGRVLITL
3681    GDQDIDLSPS FVIFLSTRDP TVEFPPDLCS RVTFVNFTVT RSSLQSQCLN EVLKAERPDV DEKRSDLLKL QGEFQLRLRQ
3761    LEKSLLQALN EVKGRILDDD TIITTLENLK KEAAEVTRKV EETDIVMQEV ETVSQQYLPL STACSSIYFT MESLKQVAFN
3841    RVARGMLHQD HITFAMLLAR IKLKGTIGEP TYDAEFQHFL RGKEIVLNTA SLPKINGLTV EQTEAMMRLS CLPAFKDLVS
3921    KVQADEQFCI WLDSSSPEQT VPYLWTEEKP ATPIGQAIHR LLLIQAFRPD RLLAMAHTFV STNLGESFMS IMEQPLDLTH
4001    IVDTEVKPNT PVLMCSVPGY DASGHVEDLA AEQNTQITSI AIGSAEGFNQ ADKAINTAVK SGRWVMLKNV HLAPGWLMQL
4081    EKKLHSLQPH ACFRLFLTME INPKVPVNLL RAGRIFVFEP PPGVKANMLR TFSSIPVSRM CKSPNERARL YFLLAWFHAI
4161    IQERLRYAPL GWSKKYEFGE SDLRSACDTV DTWLDDTAKG RQNISPDKIP WSALKTLMAQ SIYGGRIDNE FDQRLLNTFL
4241    ERLFTTLSFD SEFKLACKVD GHKDIQMPDG IRREEFVQWV EMLPDTQTPS WLGLPNNAEK VLLTTQGIDM ISKMLKMQML
4321    EDEDDLAYAE TEKKTRTDST SDGRPAWMRT LHTTASNWLH LIPQTLNHLK RTVENIKDPL FRFFEREVKM GAKLLQDVRQ
4401    DLADVVQVCE GKKKQTNYLR TLINELVKGI LPRSWSHYTV PAGMTVIQWV SDFSERIKQL QNISQAAASG GAKELKNIHV
4481    CLGGLFVPEA YITATRQYVA QANSWSLEEL CLEVNVTTTQ NAVLDACSFG VTGLKLQGAT CSNNKLSLSN AISTVLPITQ
4561    LRWIKQTNAD KKANVVTLPV YLNFTRADLI FTVDFEIATK EDPRSFYERG VAVLCTE
```

5.80 phosphoglycerate mutase 1 (brain) [Gallus gallus]

Protein Accession	gi|71895985
Mean Expression Ratio	1.11
Median Expression Ratio	1.11
Credible Interval	(0.935, 1.32)
Associated Peptides	4
Associated Spectra	5
Coverage	0.268

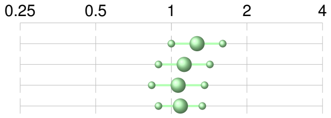

A	2.5	50	97.5	Sequence
1	1	1.3	1.6	HGESAWNLENR
1	0.89	1.1	1.4	FCGWYDADLSPAGQQEAR
1	0.84	1.1	1.4	SFDIPPPPMQSDHPFFSTISK
2	0.89	1.1	1.3	NLKPIKPMQFLGDEETVR

```
1       MAAYRLVLVR HGESAWNLEN RFCGWYDADL SPAGQQEARR GGEALRDAGY EFDICFTSVQ KRAIRTLWNV LDAIDQMWLP
81      VVRTWRLNER HYGALTGLNK AETAAKHGEA QVKIWRRSFD IPPPPMQSDH PFFSTISKDR RYADLTEDQL PTCESLKDTI
161     ARALPFWNEE IVPQIKEGKR VLIAAHGNSL RGIVKHLEGM SEEAIMELNL PTGIPIVYEL DKNLKPIKPM QFLGDEETVR
241     KAMEAVAAQG KVKK
```

5.81 PREDICTED: similar to alpha-tubulin 8 [Gallus gallus]

Protein Accession	gi|50729032
Mean Expression Ratio	0.9
Median Expression Ratio	0.9
Credible Interval	(0.695, 1.17)
Associated Peptides	1
Associated Spectra	1
Coverage	0.0313

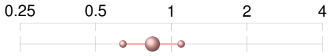

A	2.5	50	97.5	Sequence
1	0.64	0.84	1.1	LISQIVSSITASLR

```
1       MRECISVHVG QAGVQIGNAC WELFCLEHGI QPDGTFKDQH NQLNYDDSFT TFFNETVTGK HVPRAVIVDL EPTVVDEVRA
81      GTFRQLFHPE QLITGKEDAA NNYARGHYTV GKESIDIVID RVRKLADACS GLQGFLIFHS FGGGTGSGFT SLLMERLSVD
161     YGKKSKLEFA IYPAPQVSTA VVEPYNSILT THTTLEHSDC AFMVDNEAIY DICRRNLDIE RPTYTNLNRL ISQIVSSITA
241     SLRFDGALNV DLTEFQTNLV PYPRIHFPLV TYAPIISSER AHHEQLSVAE ITSSCFEPNN QMVKCDPRHG KYMACCMLYR
321     GDVVPKDVNV AIAAIKTKRN IQFVDWCPTG FKVGINYQPP TVVPGGDLAQ VQRAVCMLSN TTAIAEAWAR LDHKFDLMYA
401     KRAFVHWYVS EGMEEGEFAE AREDLAALEK DYEEVGTDSF EDENDEE
```

5.82 beta-H globin [Gallus gallus]

Protein Accession	gi|408500
Mean Expression Ratio	0.9
Median Expression Ratio	0.9
Credible Interval	(0.743, 1.08)
Associated Peptides	3
Associated Spectra	4
Coverage	0.293

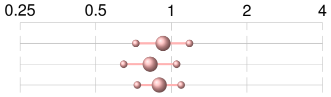

A	2.5	50	97.5	Sequence
1	0.72	0.93	1.2	FFASFGNLSSATAIIGNPMVR
1	0.65	0.82	1.0	KVLSSFGEAVK
2	0.73	0.9	1.1	VVAHALAHEYH

```
1       MVHWTAEEKQ LITGLWGKVN VAECGAEALA RLLIVYPWTQ RFFASFGNLS SATAIIGNPM VRAHGKKVLS SFGEAVKNLD
81      NIKKSFAQLS KLHCDKLHVD PENFRLLGDI LIIVLASHFS KDFTPASQAA WQKMVRVVAH ALAHEYH
```

5.83 chaperonin containing TCP1, subunit 8 (theta) [Gallus gallus]

Protein Accession	gi|52138673
Mean Expression Ratio	1.11
Median Expression Ratio	1.11
Credible Interval	(0.93, 1.33)
Associated Peptides	4
Associated Spectra	4
Coverage	0.0602

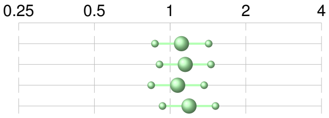

A	2.5	50	97.5	Sequence
1	0.87	1.1	1.4	FAEAFEAIPR
1	0.9	1.1	1.5	LFVTNDAATILR
1	0.84	1.1	1.4	LYAVHQEGNK
1	0.93	1.2	1.5	KFAEAFEAIPR

```
1     MALHVPKAPG FAQMLKEGAK HYSGLEEAVY RNIQACKELA QTTRTAYGPN GMNKMVINHL EKLFVTNDAA TILRELEVQH
81    PAAKMLVMAS HMQEQEVGDG TNFVLVFAGV LLELAEDLLR MGLSVSEVIE GYEKACKKAL EILPDLVCCS AKNLRDVDEV
161   ASLLHTSVMS KQYGNESFLS KLIAQACVSI LPDSGHFNVD NIRVCKIVGA GVSASSVLHG MVFNKETEGD VTSVKDAKXA
241   VYSCPFDGMI TETKGTVLIK NAEELMNFSK GEENLMDLQV KAIADSGANV VVTGGKVADM ALHYANKYNL MIVRLNSKWD
321   LRRLCKTVGA TALPRLTPPT LEEMGHCNSV YLSEVGDTQV VVFKHEKEDG AISTILIRGS TDNLMDDIER AVDDGVNTFK
401   VLTRDKRLVP GGGATEIELA KQITSYGETC PGLDQYAIKK FAEAFEAIPR ALAENSGVKA NEVISKLYAV HQEGNKNVGF
481   DIEAEAAAVK DMLEAGILDT YLGKYWGIKL ATNAAVTVLR VDQIIMAKPA GGPKPPSGKK DWDEDQND
```

5.84 PREDICTED: similar to RING finger protein 29 isoform 2 [Gallus gallus]

Protein Accession	gi|118087030
Mean Expression Ratio	0.9
Median Expression Ratio	0.901
Credible Interval	(0.732, 1.10)
Associated Peptides	2
Associated Spectra	3
Coverage	0.0692

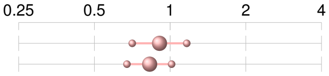

A	2.5	50	97.5	Sequence
1	0.7	0.9	1.2	AASEPSPAGQAEAAAGSSEQSTEPTR
2	0.67	0.83	1.0	FDYLYSVLEER

```
1     MSTSLGYKSF SKEQQTMDNL EKQLICPICL EMFTKPVVIL PCQHNLCRKC ASDIFQASNP YLPTRGGTTV ASGGRFRCPS
81    CRHEVVLDRH GVYGLQRNLL VENIIDIYKQ ESTRPERKCD LPMCEEHEDE RINIYCLNCE IPTCSLCKVF GAHKDCQVAP
161   LTNVYQQQKS ELSDGIAVLV GSNDRVQGIV TQLEETCKTV EECSRRQKEQ LCEKFDYLYS VLEERKNEMT QIITRTQEEK
241   LEHVRSLMKK YADHLEAVSK LVESGIQFME EPEMAVFLQN AKTLLQKITE ASKGFQMEKI EDGYENMNQF TVNLSREEKI
321   IREIDFDREE EAEEEEEETV EGEDLDEVHT ESSGEEGEEE EKEEEEGAER APQPPQQDPE QQNAVGEPPA DPVPVPLPAA
401   PAGQDEVVTP SGSQQTAESD SQVPPAAETI DPLFYPSWYK AQPRPASSPS SAPESGQGKV SPETNAKKAE APEVAAVEES
481   APGSGKESNA TAATSKAASE PSPAGQAEAA AGSSEQSTEP TRHIFSFSWL NSLTE
```

5.85 phosphoglycerate kinase 1 [Gallus gallus]

Protein Accession	gi|45384486
Mean Expression Ratio	1.11
Median Expression Ratio	1.11
Credible Interval	(0.913, 1.35)
Associated Peptides	3
Associated Spectra	3
Coverage	0.0504

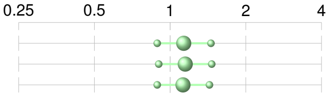

A	2.5	50	97.5	Sequence
1	0.9	1.1	1.4	VLPGVDALSSV
1	0.9	1.1	1.5	FHVEEEGK
1	0.89	1.1	1.4	FHVEEEGKGK

```
1     MSLSNKLTLD KVDVKGKRVV MRVDFNVPMK DHKITNNQRI KAAVPTIKHC LDHGAKSVVL MSHLGRPDGV PMPDKFSFSP
81    VAVELKALLG REVSFLKDCV GPEVEKACAN PANGSVILLE NLRFHVEEEG KGKDASGNKI KADAAKVEAF RASLSKLGDV
161   YVNDAFGTAH RAHSSMVGVH LPQKAAGFLM KKELDYFAKA LESPERPFLA ILGGAKVQDK IQLISNMLDK VNEMIIGGGM
241   AFTFLKVLNN MQIGNSLFDE EGSKIVKDLM AKAEKNGVKI TLPVDFITAD KFDEHAQTGE ATVASGIPAG WMGLDCGPES
321   VKKFVEVVGR AKQIVWNGPV GVFEWDKFSK GTKALMDKVV EVTGKGCITI IGGGDTATCC AKWNTEDKVS HVSTGGGASL
401   ELLEGKVLPG VDALSSV
```

5.86 basement membrane-specific heparan sulfate proteoglycan core protein [Gallus gallus]

Protein Accession	gi|94536813
Mean Expression Ratio	0.902
Median Expression Ratio	0.902
Credible Interval	(0.697, 1.17)
Associated Peptides	1
Associated Spectra	1
Coverage	0.00197

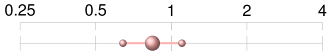

A	2.5	50	97.5	Sequence
**1**	**0.64**	**0.84**	**1.1**	**AWRRADGR**

```
1       MERPVPAALL LLAALGVCHA AGVPAESSFP EDTVADHVGS TWRRRYYAQL SDDEDLLADE ASADGSGELG SGDVALVALA
81      PTVYFRALVN FTRSIDFSPR LEDPNSEEFR EVSEAVVDTL ESEYYKIPGE QMVSVVFIKE LEGSVFVELD VGSEGNGDEA
161     QIGAVLRSVV TAGSIASFVT SPVGFQFRRL GAVTPHLRPC TPLEFSCGSG ECIAREYRCD RRPDCRDASD EQGCAEPPPS
241     TAPPSTARPA TTARPALTTP PGTRRPPPPP HGCRPAETAC ADGRCVPRDY LCDGERDCAD GSDEEGCGTP SPCEPNEFKC
321     RNGHCALKLW RCDGDNDCGD GSDETGCPTK VPGMPCGPDQ FSCVVSGACI PASYHCDEEP DCPDRSDEVG CMPPQVVTPP
401     QESVRAVPGQ TVSFTCVATG VPTPIITWRL NWGSIPSSRR VSIVSEGGQG TLTIRDVKES DQGAYTCEAI NTRGMVFGIP
481     DSILTVTPRP GPCPEGHFQV MGTSRCLPCF CFGVTSSCRA TTRHRHRIHL SFNRPDDFKG VNVTVPSAPS VPALSATQLH
561     VDMATEEFQL LDLSRRFLAL DAFWALPAQF LGDKVDAYGG ALSYGVRYRL GRGPPEPAPR PDVLLRGHGR QLRAHTRATQ
641     PDVLNRRRIP FTEDNWEDET GAPVSRELLL MVLQRLEGIL VRAVYDGRMA SVGLSDVAMD VTGPGDTGLE PAGDVEECRC
721     PVGYTGLSCQ RCAANFERVP QGPYLGTCSG CSCHGHSSTC DQVYGHCLNC QHNTEGPQCE KCKPGFFGDA TQGTATACRP
801     CPCPYTETVR RFSESCFLDT DGQATCDACA PGYAGRRCER CAPGYEGDPI QPGGKCTRIG EELIKCDARG GKDAVGGTCR
881     CKPNVTGRQC DECAVGTFHL SDANPDGCLK CFCMGVSRQC ASSSWSRDQV RVTSEEVAPL HLANLAGTRT AIEGSRFAAT
961     RELVFSDFHT LPRDVYFWVL PSSFTGDKVT SYGGELKYTV THHAPAGRQL LQHQPDVLLQ GNGISLKYFS DASPMLGVPT
1041    TVTVPFRERA WRRADGRDAT REHLLMALAD IDVLMIRASY SEQPAESRLA DVRLDVAVPH ATGRPLALEV EDCTCPPGYR
1121    GPSCQDCDVG YTRSTSGLYL GTCERCQCHG HATECHPETG ECQGCRDHTE GAQCDKCQPG YYGDATHGTP GDCRPCPCHG
1201    PRTDGQSCFE DTDGQPTCSA CAPGHTGRLC ERCLPGYVGD PLRGELCHEP GTAGGQCQCD MQGSISERCD ASGLCQCKAN
1281    VEGPHCATCR PHHFHLSAEN PVGCQPCFCM GIVQHCTSTS YSRDTVRTPF AAGSWQGFAL VNRQRSTRVA TGFSVEMGSQ
1361    GPQLTYGRFG ELSPESYYWQ LPQPYLGDKV GSYGGRLRYA LTYTPGGRGG PLPDADIQIT GNDITLVAYQ PELPPRTLRA
1441    FEVVFREHHW QRPDGQPATR EHLMMALADL DEILIRATYA SSTAWAAIAA LSMDTAVPPR PGLPPAPEVE ECRCPPGYRG
1521    LSCQDCAPGY TRTGGGLYLG HCELCECNGH SDTCHPESGL CSGCLHNTAG DFCDQCAPGF YGDATAGTHE DCQPCACPLL
1601    HPENQFSRTC ESLGGGGYRC TACAPGYAGQ YCERCAPGYT GDPSVRGQTC VPLGSPSLLA VRVHPPRTAV PQGSAVTLRC
1681    QASGEPPLYY HWSREDGRPL PSSAQSRRQG EELHFANIQP SEAGVYVCSC RNLQHSNTSR AEVIVTETPT KPITVTVEEK
1761    RVQRVKPGAD VTFICTAKSK SPAYTLVWTR QNHGTLPSRA MDFNGILTIR NVQPEDAGVY VCTGSNMLDM DEGTATLYVQ
1841    APSKTQMYYG PVEFMEGHRP AATATAPTAS VEPAQLSVVV GQPAEFRCVA TGSPTPTLEW LGALPPRAVV QGDTLRFSAV
1921    EPSDEGHYAC RARSSAGQDM ARGYLRVQGA DEPRVQVSPE RTEVQEGSTV RLYCRVSGSP TATISWEKQG GTLPPQSRSE
2001    RTDIATLLIP SITVADGGIY LCVGTSAAGT ARASIEVVVV PGAAPPVRIE SSSPSVTEGQ TLDLDCVVAG PGHVTVTWYR
2081    RGGSLPAGHQ VSGSRLRVPH VTMADSGEYV CRASSGTSVR EASVIVTVVS SSGLSYGPPA SGGQAPVRIE ASSSAVAEGH
2161    TLDLNCVIAS GLQATVTWYK RGGSLPARHQ VSGSRLRLLQ VTAADSGEYV CRVSSGATTK EASVMVTIQP SGASSYPPGG
2241    TTPLHIEPSS STVTEGQTLD LNCVIASPAQ ATVTWYKRGG SLPARHQVSG SRLRLLQVTA ADSGEYVCRV SSGATTKEAS
2321    VMVTIQPSGA SSYPSGVTPP VRIESSSSSV SEGQTLDLDC IVASQGQATV TWYKRGGSLP AKHQVSGTRL RIPQVSAADS
2401    GEYVCRVTTG SVTHETSLIV TIQTGAGSSY AVGVTPPVRI ETSSASVTEG QTLDLNCMVA GQGHPQVTWY RRGGALPPSS
2481    QVSGTHLRLT QVSVADSGEY VCRVTLGTAT QEASVIVTVP SSAGTYYSPG ASQPVHIESS SSAIAEGQTL DLNCVVAGSG
2561    PTTVTWYKRG GSLPAGHQVS GSRLRIPQVS AADSGEYVCR VTTGGVTQET SLVVTIDDDA GRSHPSSVTP PIRIESSASS
2641    VTEGQTLDLD CVVAGQGQAT FTWYKRSGSL PAKHQMSGSR LRLSQLSVAD SGEYVCRADL GSTSREATVV VTVTSRDSSS
2721    YRLQSPIISI DPHSMAVAPG EDATFKCRIH DGAQPINVTW RMGPGQHLQD NVKISANGSV ISITGAHVGN QGAYHCVASN
2801    RFGVASSVVN LLVQSAPTVS VMPPGPVTVK EGKSLSLECL GRGEPRPLVR WNRLGSRQKV EHQTLLHMDS QAVLQLFPAK
2881    PEHAGTYICT AQSALGSAQA RVDVSVETAQ RHPGAPRVTA PPTVTVVAGD TATLHCSATG EPEPRIEWSK LRAPLPWQHR
2961    VVNGTLVIPR AAQQDSGQYI CNASSPAGFT EVFVTLDVET PPYATSLPED SAVRAGDTVQ LQCLAHGTPP LFYTWDKLNG
3041    SLSPRAVPRA GLLRISPALP SDAGTYRCMV TNRVGGRAET FARVTVHGDG GGDSGGPLAV RVTPGSLVRG VGSTAEFACT
3121    ASDPRVHLEW LKDGGELPPR HSVQDGVLRI AELAQGAQGV YYCRASTLHG QVEDRATLTV QALPRALINI RTAVQTVLAG
3201    MEVELECLGL GEPQPHVTWS KVGGRIRPGV LVRAGTLTIE RVERADAGQY RCTATNSVGT VQSHVILHVQ AAPHIAGQPE
3281    VKEVSVGSAA VLPCLASGFP VPEISWSKLE GELPEGARVD GTALMLPAVR LEDAGVYACA ASNRRGQETA FYVLKVQERL
3361    VPYFTQTPRS FLPLPTIKDA YKTFEIQITF RPDAADGMLL YNGQRKSSGA DFISFGLVGG RPEFRFDAGS GMATIRDPTP
3441    LRLGQYHTVR LFRNLTRGSL QVDGQPPVNG TSQGKFQGLD LNEELYLGGY PDYTIVAKTG LSRGFVGCVR QLRIQNEEVA
3521    FGELDLQAHG VSNCPTCQDQ PCQNGGICED AESSTYICRC PQGFTGSNCE YSQALHCHPE ACGPDATCIS RPDGQGYSCR
3601    CHLGKMGERC TEGEAVSVPS FDEAGAFISY PPLTNVHHEL RVEAEFLPRA PDGLLLFSAG KASPVEDFVA LAMVSGHLEF
3681    HYELGSGTAV LRSVEPVALG RWHRVTAERV HKDGTMTVDG GALVQRSSPG KSQGLNLRSP LYLGGVEPPL RPPTNASFQG
3761    CIGEVSINGK KVDLSYSFLR SRGVGQCGQS SPCLHAPCLH GGRCLPLPAS SPPFRCLCTP GFSGPRCERA ADRCLEHNPC
3841    LHGGTCKDNG CICPKGYAGP YCQHGAALSE LDQDWQEGSG GSDTPGQFSA MFREGSYLAL PGHLFPRGAP DLQDTIELEL
3921    RTSSTEGLLL WHGTESGKAK DFVGLGLKDG HLVFSYQLGS GEATIVSEDP VNDGEWHHVM AARQGRRGWL QVDGEEPVFG
4001    ESPGTNVMAN TQGNVYVGGA PDPRSLTAGK FLTGVSGCVR GLVLAPAGTP RHPTDLRHGA VGGSAVVPCP S
```

5.87 vitronectin [Gallus gallus]

Protein Accession	gi|46048795
Mean Expression Ratio	0.902
Median Expression Ratio	0.903
Credible Interval	(0.696, 1.17)
Associated Peptides	1
Associated Spectra	1
Coverage	0.0265

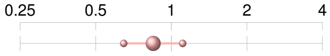

A	2.5	50	97.5	Sequence
**1**	**0.65**	**0.84**	**1.1**	**FDDGALDPGYPR**

```
1       MRLLLPVLVL ALLALTRAAE DSCEGRCDEG FNAMKKCQCD TLCNYYQSCC SDYSTVCKAK VTRGDVFALP EDDYLDYDLS
81      IDTGTVGPTE APAPTEHPTE PHSSPSPTRV IDTATEETPE EPEVPTLHPT TTTTTTTSDE TRNPSLDDEP EELCSRKPFN
161     AFTDLKNGSI YAFRGKYFYE LDKSSVRPGY PKLISDVWGI EGPIDAAFTR INCQGKTYLF KGSQYWRFDD GALDPGYPRD
241     ISEGFEGIPN DIDAAFALPA HSYHGNERVY FFKGKYYWSY DFAHQPTQAE CEKSSPSTVF NHYAFMNRDS WEDIFLSLFG
321     SRMVGASSQR LISRDWRGVP NQLDAAMAGR IYVSSRQPRR RSSRRHRKRY RNHRTLNLGL WSWLNSDSES TDTESDWLSG
401     SQCETLQSVY FFVGDKYYRV NLRTKRVDLV QPPYPRSIAQ YWLDCPQPDE EST
```

5.88 filamin B, beta (actin binding protein 278) [Gallus gallus]

Protein Accession	gi|71896431
Mean Expression Ratio	0.903
Median Expression Ratio	0.903
Credible Interval	(0.697, 1.17)
Associated Peptides	1
Associated Spectra	1
Coverage	0.00545

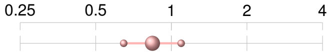

A	2.5	50	97.5	Sequence
**1**	**0.64**	**0.84**	**1.1**	**RLPNNHIGISFIPR**

```
1       MSTQARGQGG ARAADPDGEM PATEKDLAED APWKRIQQNT FTRWCNEHLR CVNKRIGNLQ HDLSDGLRLI ALLEVLSQKR
81      MYRKYHQRPT FRQMQLENVS VALEFWSGRA SSWLSIDSKA IVDGNLKLIL GLIWTLILHY LISMPVWEDE GDDDAKKQTP
161     KQRLLGWIQN KIPYLPITNF NQNWQDGKAL GALVDSCAPG LCPDWETWDP SKPVDNAREA MQQADDWLGV PQLLPPEEII
241     HPDVDEHSVM TYLYTFPKAK LKPGAPLKPK LNPKKARAYG RGIEPHGNMV KQPAIFTVDT ISAGQGDLMV FVEDPEGNRE
321     EAKIMPSSDK NKTYSVQYVP KVTGPHKVSV LFAGQHISKS PFEVNVDKAQ GDASKVTAKG PGLEATGNIA NKPTYFDLYT
401     AGAGVGDVIV EVEDPQGRCL AEVAVEDKGN QVYRCVYKPV QAGPHVVKVT FAGEAIPKTP CSVLIGEACN PNACRATGRG
481     LQPKGVRIRE TATQLIREQQ AVETLGTIKT RLEELVKQKG FMDGVYAFEY YPATPGKYVV TITWGGHNIP KSPFEVHIGH
561     EAGPQKVRAW GPGLHEGIVG RSADFVVESI GTEVGSLGFA IEGPSQAKIE CDDKNDGSCD VKYWPKEPGE YAVHIMCDDE
641     DIKDSPYMAF IRPASGDFNP DKVRAYGPGL ERSGCIVNNP AEFTVETKDA GKAPLKIYSQ DGEGNPIDIQ MKSKPDGVFA
721     CSYVPVKPIK HTISIVWGGA NVPSSPFRVL IGQGSHPQKV KVFGPGVERT GLKASEPTHF TVDCTDAGEG DVSVGIKCDA
801     RVVSDEEEDI DFDIIHNAND TFTVKYAPPA AGRYTIKVLF AGEEIPASPF RVKVDPSHDA SKVKVKLPGL SQNWCGKCKP
881     THFTVFTKGA GKAPLDVQFS SPVPGEVVTD VDIIDNIDYS HTVRYTPIQQ GPMKVLVTYG GDPIPKSPFT VGVAAPLDLS
961     KVKVNGLENR VEVGKDQEFV IDTRGAGGQG KLDVNISSPM RKAVPCLVEP VLGKECSTAK YIPREEGLYV VDVSYDGNPI
1041    QAALYCGATL PPDPSKVKAH GPGLSRRPRW KTSSIHNIDT KGAGTGGLGL TVEGPCEAKI ECSDNGDGTC SVSYLPTKPG
1121    EYFVNILFEE VHIPGSPFKA DIEMPFDVSK VIATGPGLER GKVGEAGLLN VDCTEAGPGN LRVDMVSDTV SKAEIQIDDN
1201    KDGTYVVTYV PLSAGMYTIK MKYGGEQVPK FPARVKVEPA VDTSRVKVFG PGVEGKDVFR EATTDFTVDA RPLTKAGGDH
1281    IRTQITSPSG SPTDCQIQDN ADGTYAVEYT PFEKGPHTVN VTYDGVPVPN SPFRVNVTEG CHPSRVKAQG PGLKEAFTNQ
1361    PNAFSVVTRG AGIGGPGITV EGPSESKISC KDNKDGSCSA EYVPYVPGDY DVNITYGGEH IPGSPFKVPV KDVVDPSKVK
1441    IAGPGLGTAV RAKVPQSFTV DTSKAGVAPL EVVVAGPRGI VEPVNVVDNG DGTHTVCTPL HRRDHTDLCQ ISDEEIPRSP
1521    FKVKVLPTYD ASKVTASGPG LSSYGIPASL PVEFAVDAKD AGQGLLTVQI TDQEGKPKRV DIHDNKDGTY TVTYVPDKTG
1601    RYTIGVKYGG DDIHPLPTES ASPAGDAAQC LATGLGIAPT VRTGEEVGFV VDAKSAGKGK VTCTVLTPDG TEAEADVVEN
1681    EDGTYDIFYT AAKPGTYVIY VRFGGVDIPN SPFTVMVTEE AYVPVGDMNG MGFRPFDMVI PFAVRKGEIT GEVHMPSGKT
1761    DTPDIVDNKD GTVTVRYAPT EVGLHEMHIK YMGNHIPESP LQFYVNYPNS GSVSAYGPGL IYGVANKPAT FTIVTEDAEE
1841    GGLDLAIEGP SKAEISCIDN KDGTCTVTYL PTLPGNYSIL VKYNDKHIPG SPFTAKITDD NRRRSQVKLG SAAEFLLDIN
1921    ETDLSLLTAS IKAPSGRDEP CLLKRLPNNH IGISFIPREV GEHLVSIKKN GNHVPNSPVT IMVVQSEIGV ARRARVYGRG
2001    LVEGRTFEMC DFIVDTRDAG YGGISLAVEG PSKVDIQTED LEDGTCKVSY FPTVPGVYIV STKFADEHIP GSPFTVKISG
2081    EGRVKESITR TRRAPSVATV GSICDLNLKI PQIDCGDMTA QVTSPSGRNS DAEIVEVDKN TYCVRFVPQE MGVTRWMSNT
2161    EGPVPGSPFQ FTVGPLGEGG AHKVRAGAQG GAWREGVPAE FSIWTREAGA GGLSIAVEGP SKAEIAFEDH KDGSCGVSYI
2241    VQEPGNYEVS IKFNDEHIPE SPYLVPIIAP SDDARRLTVT SLQESGLKVN QPASFAIRLN GAKGKIDAKV PTPSGAVEEC
2321    HVSELEPDKY AVRFIPHENG IHSIDVKFNG STWWAAPSRC VLVTGQAGNP TLVTAYGPGL ESGTTGLQSE FFINTTKAGP
2401    GTLSVTIEGP SKVKMDCQET PEGYKVMYTP MAPGNYLIGV KYGGPNHIVA ALQGKGYRKR LVTLGSANET SSIMVESVTR
2481    SSTETCYSAI PKSTSDASKV VSRGAGLSKA FVGQKRLLLC GLQKAGSNML LVGVHGPTIP CEEVSIKHLG NHQYNVCGEG
2561    EGHYVWR
```

5.89 PREDICTED: hypothetical protein [Gallus gallus]

Protein Accession	gi|118085349
Mean Expression Ratio	1.11
Median Expression Ratio	1.11
Credible Interval	(0.86, 1.42)
Associated Peptides	1
Associated Spectra	1
Coverage	0.0058

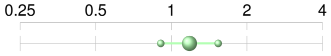

A	2.5	50	97.5	Sequence
**1**	**0.9**	**1.2**	**1.5**	**LALLGRIHLAR**

```
1       MQQRRPRASA PAPGVSRRRR APGDIPASTS LPPLVEGPPR CVLCCTVGRI VWTAAKPPSA AVLVRLRWWG ETSDGTVFHP
81      SSKPGQAAGS SRYTVRCGPR QLTAYLTDMG MLVLEVLTKP DRLPVGRVQI TELSQLSPSH PISGFFTIVS PTADKLGELQ
161     VSLVLEPLSD TYKTSSSGPT TATSSDMTPA PSQQMQTTPP GQEPVNKSRV TTPSTDPEAP LSTNTHVFSL HNPTTKDLLS
241     ALLDQGNKLR DAMVASAMKS SPDMEIELNE VPPFVKPDGF RAAAKSPKGL NSTFMPPPED THLFTSEVTG QQLDLNSEER
321     AIQLLLGSAD LSPVHFWDQP GSLLDSLSVG SEVYDSELND PHYDQSLLES LFYTTPKSDS STSDFLSDED VNPSKKVSKT
401     KTVKKADSVN AQKDSTASDQ DQKVTEIKPS CSPTRQLAPP DVDTEEAHVT SLSVDRLALL GRIHLARVII ESLRIPLESS
481     QITPRKKGLM GKPPRPASVN KCTFFVEYHF PVGASKNEKG QVSLTTELTR IASSKITDGV VKFQQRFVFP VRFGGTMIEH
561     WWSSDLAFKI YMRKSTQRKP VAIGSAVLQL CKVIQSELLT FSCEIPVEKE GDQMQVGPLK LSVELAADNK DFTCTTARCS
641     VAVQQQVPPR AIRSPQMELW EPNRDRVNAE SCLKFRNHVD SEKTGAACTN VVQPPRSVST PVARNLMTQL PAAEDYGLLL
721     HVLLMVPDGK DFVAENSGLH TSCNVYLNCK LLSTEEATRS AVVWGTTQPT FNFSQVMPFS LTSKHLERLK NNVMVIEAWH
801     KMGNPGNDRL LGLVKLPLHQ FYISFKDPEI SHLLLQAHYP VVAVDSYMPV IDVFTGSKSG SLRVILAMGS ADQIVALQRL
881     KNEEGMEPPI TQRPAHFLDP PPTKLTMQLE REGEELMEHV FEIHVESVKG LTPLQSTVWG EADCYVQYYF PVQEAGSGAL
961     PGTELHTDGI ELKPFRTATT LCVPDPVFND EHHHSLLVPA DVPVQRLLVS AFMAPGAVGG GIQFEVWCRY YYPNVRDQMV
1041    AKGTLPLSRL CAMITMQHRE EIGIQTFNLP LVPRTDSSEE FHLRSSGLLD VSVRYQRSVK MAARRTAQTV SLSVQIHRAA
1121    GLQAAARAVA EKNPSVQYYA GVGVNAYVSV HLSFLPETER HSTRAVARSF CPEFEHHVEI PCNLIVQKSS GEASCLGELL
1201    QSANITFSVY HQSIKSATDT MAARTSRDYL LGTVTIPTRD LLRRRSGITG WYPITLSEDV MPSHCTNIMQ TVVGGLELSV
1281    TFAHHNDRER ILKAAQLLGW NGEESLEDSM DESDEWEPSD SPMVVTISTP RVWLPLHCVL LAGQMHLSKS TCCYLRYKLY
1361    DQEAMWTLLK RPKLCEDKKN VTVNFKKGHK VTLRKSQGLI WFFREEKLEI QVWWAYGKEN GMERPLDTDR LIGSAYVDLR
1441    ALAERSRRTL SVGGVYPVFR CNAADLAGAA VRVHIALTST SAAPTLTPHC TEEHSNSEDE STERAPDPSQ QISESQSQNL
1521    DVRSSEITDG KRQEDDVVFL ESTIAVSILV ERAMHLSLKG SPLTEREVAA PSSCVSFAVA GADAPITTPV IENTESPVWD
1601    FQQQVRLSKE LLLDPQQTLV FKVWHKAETE RVIGFASVDL SPLLSGFQLV CGWYNITDFS GQCRGQIKVA VSPLQSITNL
1681    KEERQARIRT QTPSSAVKAI LPALPSNVPS SSKPVLPKEI RSSSQELPAP SRTSPSKAHT PRHEEHMQNV RRFHESLQQA
1761    EGNAQRAARM DSLSLSSRAS LLTALRKNLS ELDEVQRYFS QKLTRSLPDF SDGNRSQPSH REQESDRQGM RSREVDPNRC
1841    HLLRKSSQLV SQSGRGAARG AIPYRRPQQR PALVPAELSE EEARRIARIF SARLREKE
```

5.90 PREDICTED: hypothetical protein [Gallus gallus]

Protein Accession	gi|118086446
Mean Expression Ratio	1.11
Median Expression Ratio	1.11
Credible Interval	(0.857, 1.43)
Associated Peptides	1
Associated Spectra	1
Coverage	0.0515

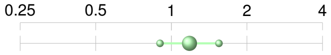

A	2.5	50	97.5	Sequence
**1**	**0.9**	**1.2**	**1.5**	**FSFALCVPPSLR**

```
1       MASPARPRPE PAAPGAWGSG AAGERLLLLE LGCARPPSPG SEEEEEEEAG EETAAGEDCC PKCKKRVQFA DSLGQRVASV
81      KHFSDAEDLR LEPDFPDGGE PSAERLRRQR VCLERLGRPA LLSDVRGTVR ALSGSGPGEV TVRYTFNEWL SFMDVPASPL
161     PPAPADGSEP PAERFSFALC VPPSLREGSA LHFAIRYRGA QGEHWDNNDG RNYTLRCLRD PEGCPEAAAA PRD
```

5.91 Chain , Mol_id: 1; Molecule: Ovotransferrin; Chain: Null; Synonym: Conalbumin; Heterogen: Iron (F

Protein Accession	gi|1127086
Mean Expression Ratio	0.905
Median Expression Ratio	0.905
Credible Interval	(0.776, 1.05)
Associated Peptides	6
Associated Spectra	8
Coverage	NaN

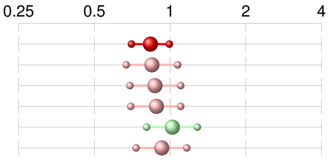

A	2.5	50	97.5	Sequence
3	0.7	0.84	1	NLQMDDFELLCTDGR
1	0.67	0.85	1.1	TGTCNFDEYFSEGCAPGSPPNSR
1	0.7	0.87	1.1	ECNLAEVPTHAVVVRPEK
1	0.7	0.88	1.1	SDFHLFGPPGK
1	0.81	1.0	1.3	HTTVNENAPDQKDEYELLCLDGSR
1	0.73	0.92	1.2	SDFHLFGPPGKK

5.92 tyrosine 3-monooxygenase/tryptophan 5-monooxygenase activation protein, theta polypeptide [Gallus g

Protein Accession	gi|55741594
Mean Expression Ratio	1.10
Median Expression Ratio	1.10
Credible Interval	(0.933, 1.30)
Associated Peptides	5
Associated Spectra	5
Coverage	0.184

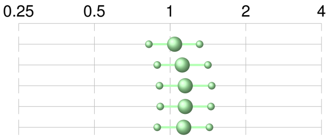

A	2.5	50	97.5	Sequence
1	0.82	1.0	1.3	YLAEVACGDDR
1	0.89	1.1	1.4	AVTEQGAELSNEER
1	0.9	1.2	1.5	QTIENSQGAYQEAFDISKK
1	0.91	1.2	1.5	YLAEVACGDDRK
1	0.89	1.1	1.4	KQTIENSQGAYQEAFDISKK

```
1       MDKTELIQKA KLAEQAERYD DMATCMKAVT EQGAELSNEE RNLLSVAYKN VVGGRRSAWR VISSIEQKTD TSDKKMQLIK
81      DYREKVESEL RSICTTVLEL LDKYLIANAT NPESKVFYLK MKGDYFRYLA EVACGDDRKQ TIENSQGAYQ EAFDISKKEM
161     QPTHPIRLGL ALNFSVFYYE ILNNPELACT LAKTAFDEAI AELDTLNEDS YKDSTLIMQL LRDNLTLWTS DSAGEECDAA
241     EGAEN
```

5.93 PREDICTED: similar to PDGF associated protein [Gallus gallus]

Protein Accession	gi|50755563
Mean Expression Ratio	0.907
Median Expression Ratio	0.906
Credible Interval	(0.701, 1.18)
Associated Peptides	1
Associated Spectra	1
Coverage	0.1

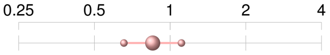

A	2.5	50	97.5	Sequence
1	0.65	0.85	1.1	AREEEEQEEGEGATGDPK

```
1       MPKGGRKGGH KGRARQYTSP EEIDAQLQAE KQKAREEEEQ EEGEGATGDP KKDKKSLDSD ESDEDDEDYQ QKRKGVEGLI
81      DIENPNRVIQ TTKKVTQLDL DGPKELSRRE REEIEKQKAK ERYMKMHLAG KTEQAKADLA RLAIIRKQRE EAARKKEEER
161     KAKDEAAMAG KRLQSLSLNK
```

5.94 PREDICTED: hypothetical protein [Gallus gallus]

Protein Accession	gi|118084200
Mean Expression Ratio	1.10
Median Expression Ratio	1.10
Credible Interval	(0.853, 1.43)
Associated Peptides	1
Associated Spectra	1
Coverage	0.114

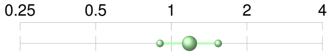

A	2.5	50	97.5	Sequence
1	0.9	1.2	1.5	EGGGGGGSSGGNGEGDSVSNK

1 MRHSGLLLAA LLALLAPVRG ATVKPVSPQK PPSDDYGFNL EDALQPSSKP DLPAKPKDSD NPRPATPVKP RESDTFDDSD
81 LFDGDLPREG GGGGSSGGNG EGDSVSNKGG DGEASQGAIA GIVSAVAATA IGAVSSFIAY QKKKLCFKQS DEENVNMDSH
161 RGAQSEPPVQ RTLLEN

5.95 Trypsin I-P38 precursor

Protein Accession	gi|2499863
Mean Expression Ratio	1.1
Median Expression Ratio	1.1
Credible Interval	(0.865, 1.39)
Associated Peptides	1
Associated Spectra	2
Coverage	0.0363

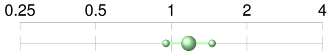

A	2.5	50	97.5	Sequence
2	0.95	1.2	1.5	VCNYVSWIK

1 MKFLVLVAFL GVAVAFPISD EDDDKIVGGY SCARSAAPYQ VSLNSGYHFC GGSLISSQWV LSAAHCYKSS IQVKLGEYNL
81 AAQDGSEQTI SSSKVIRHSG YNANTLNNDI MLIKLSKAAT LNSYVNTVPL PTSCVTAGTT CLISGWGNTL SSGSLYPDVL
161 QCLNAPVLSS SQCSSAYPGR ITSNMICIGY LNGGKDSCQG DSGGPVVCNG QLQGFVSWGI GCAQKGYPGV YTKVCNYVSW
241 IKTTMSSN

5.96 ubiquitin carboxyl-terminal esterase L1 (ubiquitin thiolesterase) [Gallus gallus]

Protein Accession	gi|122692295
Mean Expression Ratio	0.91
Median Expression Ratio	0.91
Credible Interval	(0.718, 1.15)
Associated Peptides	1
Associated Spectra	2
Coverage	0.058

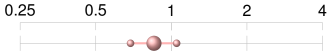

A	2.5	50	97.5	Sequence
2	0.69	0.85	1.1	FLDETADLSPEER

1 MAWQPMEINP EMLNKVLSRL GVSPGWRFVD VLGFEEEALG AVPSPACALL LLFPLTEQHE NFRKQQTEKI KDQEISSKVY
81 FLKQTVSNSC GTIGLIHAVA NNKDKVKLDE GSALKKFLDE TADLSPEERA KRFANNKAIQ EVHNSVAQEG QCRVEDNSVN
161 FHFILFANVD GHLYELDGRL PFPVNHGTSS DDLLLKDSAK ICRQFTEREK GEVRFSAVAF CKSA

5.97 Drebrin (Developmentally-regulated brain protein)

Protein Accession	gi|2507011
Mean Expression Ratio	0.91
Median Expression Ratio	0.91
Credible Interval	(0.745, 1.11)
Associated Peptides	3
Associated Spectra	3
Coverage	0.0874

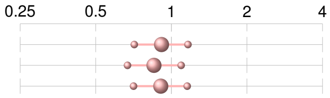

A	2.5	50	97.5	Sequence
1	0.71	0.91	1.2	GEQASEGYFSQSQEEEAPPPEEPSAK
1	0.67	0.86	1.1	YVLVNWVGEDVPDAR
1	0.71	0.9	1.2	LREDENAEPVGTTYQK

```
1       MAGVGFAAHR LELLASYQDV IGEDSPTDWA LYTYEDGSDD LKLAASGGGG LLELSGHFEI QKVMYGFCSV KEPQAVLPKY
81      VLVNWVGEDV PDARKCACAS HVAKIAEFFQ GVDVIVNASS VEDIDPGAIG QRLSNGLARV SSPVLHRLRL REDENAEPVG
161     TTYQKTDATV EMKRLNREQF WEQAKKEEEL RKEEERKKAL DARLRFEQER MEQERLEQEE RERRYREREE QIEEHRRKQQ
241     SMEAEEARQR LKEQSIFGEQ QEEDDRQQLR KSESEVEEAA AIIAQRPDNP REFFKQQERV ASGSGDAISP GSHRTGRLHC
321     PFIKTADSGP PSSSSSSSSP PRTPFPYITC HRTPNLSSFF PCSQSDYRKV SAAGCSPCES SPASTPLGEQ RTRAPAEETP
401     ATPKDSPSPS TQVAEPAATE QHWPFPGPED KAAEPPGDEP DPDPRPAWTA GADVLGDLVT LEPSEPSPAP AASEPQPVET
481     PGVAEPLIEL WQSDGAAPAA TSTWPLPDTP AGPPVPPEEG TLLGLDELPE PPATFCDAEQ HEEVEEEEEE EEATAGEPHP
561     TGLGYQEGYQ EGPEVPPITN GEMGPKDGTA GRGEQASEGY FSQSQEEEAP PPEEPSAKAP QPVFYNKPPE IDITCWDTDP
641     LPEEEESFGG GL
```

5.98 PREDICTED: similar to Aldehyde dehydrogenase 9 family, member A1 [Gallus gallus]

Protein Accession	gi|118094103
Mean Expression Ratio	1.1
Median Expression Ratio	1.10
Credible Interval	(0.882, 1.38)
Associated Peptides	1
Associated Spectra	3
Coverage	0.037

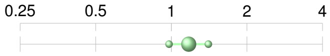

A	2.5	50	97.5	Sequence
3	0.98	1.2	1.4	VQPVDGGQTEDVYEPATGR

```
1       MVLAFLLPRL RRLQPGLAMS TATGTFSLQQ PLNYRAGGRV QPVDGGQTED VYEPATGRVI TKLLCSGEKE VDLAVQSAKA
81      AFQTWSRTSG MERCRVLLEA ARLIRERRDE IATLETINNG KSIFEARVDI DISWQCLEYY AGLAGSLAGE HIQLPGGSFG
161     YTRREPLGVC VGIGAWNYPF QIACWKSAPA LACGNAMVFK PSPFTPISVL KLAEIFTEAG VPKGLFNVVQ GGAATGQFLC
241     HHPDVAKISF TGSVPTGVKI MEMAAKGIKP VTLELGGKSP LIIFSDCSLE NAVNGALMAN FLTQGEVCCN GTRVFVERKI
321     LDTFTKEVVK RTQKIKIGDP LQEDTRMGAL INRPHLERVQ RFIKQAKEQG AQVLCGGDLY VPEDPKLKNG FYMQPCVLGN
401     CRDDMTCVQE EIFGPVMSIL PFDTEEEVVE RANNTKFGLA GGVFTRDIQK AHRVVAALKA GMCFINNYNV SPVELPFGGY
481     KSSGFGRENG RAAIEYYSQL KTVCVEMGDV ESVF
```

5.99 histidine triad nucleotide binding protein 1 [Gallus gallus]

Protein Accession	gi|45382717
Mean Expression Ratio	0.912
Median Expression Ratio	0.912
Credible Interval	(0.704, 1.18)
Associated Peptides	1
Associated Spectra	1
Coverage	0.119

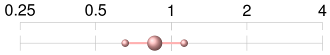

A	2.5	50	97.5	Sequence
1	0.66	0.86	1.1	KAQAARPGGDTIFGK

```
1       MADEIRKAQA ARPGGDTIFG KIIRKEIPAN IIYEDEQCLA FHDISPQAPT HFLVIPKKPI VRLSEAEDSD ESLLGHLMIV
81      GKKCAANLGL TNGFRMVLNE GPEGGQSVYH VHLHILGGRQ LGWPPG
```

5.100 acyl-Coenzyme A dehydrogenase family, member 9 [Gallus gallus]

Protein Accession	gi|57524955
Mean Expression Ratio	1.10
Median Expression Ratio	1.10
Credible Interval	(0.848, 1.42)
Associated Peptides	1
Associated Spectra	1
Coverage	0.0212

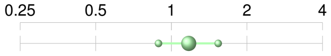

A	2.5	50	97.5	Sequence
1	0.89	1.2	1.5	ILLIFEGTNEILR

```
1       MSGLLVRALR AALPGPAAVR RLRTAAPRPA YAKELFLGAL RKEEVFPYPE ISNEELQEIN QFVGPIEKFF AEEVDSKKID
81      QDAKIPPETL KGLRELGLFG MQIPEEYGGL GLSHTMYARL GEITSLDGSI AVTLAAHQAI GLKGILIAGT DEQKAKYLPK
161     LASGEHIAAF CLTEPGSGSD AASIQTRATL SEDGKYFLLN GSKVWISNGG LASIFTVFAR TEIIEKDGQV KDKITAFIVE
241     RDFGGVTSGK PEDKLGIRGS NTCEVDFENT KVPIENVLGQ IGGGFKVAMN ILNSGRFSMG SASAGMIKKL IEMTAEHACT
321     RKQFNKKLSE FGLIQEKFCF MAVKAYVMES MAYLTAGMMD RPGFPDCSVE AAMVKVFSSE GAWACVSEAL QILGGLGYMK
401     DYPYERYLRD SRILLIFEGT NEILRMYIAL TGMQHAGKIL TDKIKEIKKG NVGLALQELV NKVRDTTGRK VDYGIMEERG
481     GLVHPSLQDS GKKLEENVYY FGTTVRGLLT RFGKTIVDEQ LALKRVADIV INLYAMTAAI SRASRSISIG LRNHDHDVLL
561     TNIFCSEAYF KNNYAMAQLQ KYADENLDDH IKKAAKQILE KRAYICSHPL DRTF
```

5.101 small glutamine-rich tetratricopeptide [Gallus gallus]

Protein Accession	gi|71897305
Mean Expression Ratio	0.913
Median Expression Ratio	0.912
Credible Interval	(0.718, 1.16)
Associated Peptides	1
Associated Spectra	2
Coverage	0.0575

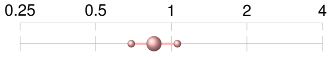

A	2.5	50	97.5	Sequence
2	0.69	0.86	1.1	ANSEPVTPSEDDIAEAER

```
1       MADQKRLAYS IIQFLHDQLQ NGGLSPDAQE SLEVAIQCLE TAFGVSLEDQ GLAVSRTLPE IFEAAAGKEP EHIRANSEPV
81      TPSEDDIAEA ERLKTEGNEQ MKAENFEAAV SFYGKAIELN PSNAVYFCNR AAAYSKLGNY AGAVRDCERA IGIDPNYSKA
161     YGRMGLALSS LNKHTEAVVY YKKALELDPD NDTYKSNLKI AEQKMKETPS PTGGPGGFDL AGLLNNPGFM SMASTLMEQS
241     TSTTSSLSGM ISGGHNPMGA AGTSPSTNDL ASLIQAGQQF AQQMQQQNPE LIEQLRSQIR SRTPSASNED QQE
```

5.102 actinin, alpha 2 [Gallus gallus]

Protein Accession	gi|46048687
Mean Expression Ratio	0.913
Median Expression Ratio	0.913
Credible Interval	(0.785, 1.06)
Associated Peptides	7
Associated Spectra	7
Coverage	0.118

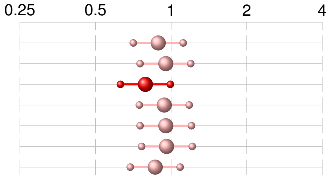

A	2.5	50	97.5	Sequence
1	0.7	0.89	1.1	ETADTDTAEQVIASFR
1	0.75	0.95	1.2	DYESASLTEVR
1	0.63	0.79	1	ISASNPYSTVTVEEIR
1	0.75	0.94	1.2	ACLISMGYDLGEAEFAR
1	0.75	0.95	1.2	VGWELLLTTIAR
1	0.76	0.96	1.2	LMLLLEVISGER
1	0.69	0.86	1.1	HNIDKLEGDHQLIQEALVFDNK

```
1       MNSMNQIETN MQYTYNYEED EYMTQEEEWD RDLLLDPAWE KQQRKTFTAW CNSHLRKAGT QIENIEEDFR NGLKLMLLLE
81      VISGERLPKP DRGKMRFHKI ANVNKALDYI ASKGVKLVSI GAEEIVDGNV KMTLGMIWTI ILRFAIQDIS VEETSAKEGL
161     LLWCQRKTAP YRNVNIQNFH LSWKDGLGLC ALIHRHRPDL IDYSKLNKDD PIGNINLAME IAEKHLDIPK MLDAEDIVNT
241     PKPDERAIMT YVSCFYHAFA GAEQAETAAN RICKVLAVNQ ENERLMEEYE RLASELLEWI RRTIPWLENR TPEKTMQAMQ
321     KKLEDFRDYR RKHKPPKVQE KCQLEINFNT LQTKLRISNR PAFMPSEGKM VSDIAGAWQR LEQAEKGYEE WLLNEIRRLE
401     RLEHLAEKFR QKASTHEQWA YGKEQILLQK DYESASLTEV RAMLRKHEAF ESDLAAHQDR VEQIAAIAQE LNELDYHDAA
481     SVNDRCQKIC DQWDSLGTLT QKRREALERT EKLLETIDQL HLEFAKRAAP FNNWMEGAME DLQDMFIVHS IEEIQSLISA
561     HDQFKATLPE ADGERQAILS IQNEVEKVIQ SYSMRISASN PYSTVTVEEI RTKWEKVKQL VPQRDQSLQE ELARQHANER
641     LRRQFAAQAN VIGPWIQTKM EEIARSSIEM TGPLEDQMNQ LKQYEQNIIN YKHNIDKLEG DHQLIQEALV FDNKHTNYTM
721     EHIRVGWELL LTTIARTINE VETQILTRDA KGITQEQMND FRASFNHFDR RKNGLMDHDD FRACLISMGY DLGEAEFARI
801     MSLVDPNGQG TVTFQSFIDF MTRETADTDT AEQVIASFRI LASDKPYILA DELRRELPPE QAQYCIKRMP QYTGPGSVPG
881     ALDYTSFSSA LYGESDL
```

5.103 prolyl endopeptidase [Gallus gallus]

Protein Accession	gi|57530288
Mean Expression Ratio	1.10
Median Expression Ratio	1.09
Credible Interval	(0.9, 1.33)
Associated Peptides	3
Associated Spectra	3
Coverage	0.0507

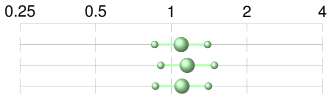

A	2.5	50	97.5	Sequence
**1**	**0.86**	**1.1**	**1.4**	**FIATLQYVVGR**
**1**	**0.9**	**1.2**	**1.5**	**VIEEVSDMFAFIAR**
**1**	**0.86**	**1.1**	**1.4**	**AGHGAGKPTAK**

```
1     MQAFQYPEVY RDEAAVLDYH GHQISDPYCW LEDPDSEQTK AFVEAQNKLT VPFLEQCPVR GLFKERMTEL YDYPKYSCHF
81    KKGKRYFHFY NTGLQNQRVL YVQDSLDADA KVFLDPNKLS DDGTVALRGY AFSEDGEYFA YGLSSSGSDW VTIKFMKVEG
161   AEELPDTLER VKFSCMAWTH DGKGMFYNCY PKQDGKSDGT ETSTNLHQKL HYHVLGTNQS EDILCAEFPD EPKWMGGAEI
241   SDDGRYVLLS IREGCDPVNR LWYCDLQKES QGISGILQWV KLIDNFEAEY EYVTNEGTVF TFKTNRHSPN YRLINIDFSD
321   PEESKWKVLI PEHERDVLEW VACVRSNFLV LCYLHDVKNI LQLHDLATGA HLKTFPLDVG SIVGYSGQKK DNEIFYQFTS
401   FLSPGIIYHC DLTKEELEPR VFREVTVKGF DPSVYQTIQV FYPSKDGTKI PMFIIHKKGI KLDGSHPAFL YGYGGFNISI
481   TPSYSVSRLI FVRHLGGVLA VANIRGGGEY GETWHKGGIL ANKQNCFDDF QYAAKYLIRE GYTAPKKLTI NGGSNGGLLV
561   AACANQRPDL FGCVIAQVGA MDMLKFHKYT IGHAWTTDYG CSDHKEQFEW LCKYSPLHNV KLPEEDGIQY PATLLLTADH
641   DDRVVPLHSL KFIATLQYVV GRSRKQTNPL LIHVDTKAGH GAGKPTAKVI EEVSDMFAFI ARCLNLDWIE
```

5.104 phosphoglucomutase 1 [Gallus gallus]

Protein Accession	gi|84619526
Mean Expression Ratio	1.09
Median Expression Ratio	1.09
Credible Interval	(0.883, 1.35)
Associated Peptides	2
Associated Spectra	3
Coverage	0.0531

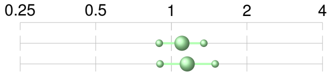

A	2.5	50	97.5	Sequence
**2**	**0.9**	**1.1**	**1.3**	**TGEYDFGAAFDGDGDR**
**1**	**0.9**	**1.2**	**1.5**	**ADNFEYNDPVDGSVSR**

```
1     MVHIETVKTK AYADQKPGTS GLRKRVTVFQ NNAHYAENFI QSILATVPPA ERQEATLVVG GDGRFYMKDA IQIIVRIAAA
81    NGIGRLVIGQ NGILSTPAVS CIIRKIKAIG GIILTASHNP GGPNGDFGIK FNTANGGPAP EGITDKIFQI SKKIEEYAIC
161   PDLKVDLGTI GKQQFDLENK FKPFTVEIVD SVEAYANMLR NIFDFNALKE LLSGKNHLKI RIDAMHGVVG PYVKKILCEE
241   LGAPANSAVN CTPLEDFGGH HPDPNLTYAA DLVQTMKTGE YDFGAAFDGD GDRNMILGKH GFFVNPSDSV AVIAANIFSI
321   PYFQQTGVRG FARSMPTSGA LDRVAHATKI ALYETPTGWK FFGNLMDANK LSLCGEESFG TGSDHIREKD GLWAVLAWLS
401   ILAARKQSVE DIMKDHWQKY GRNFFTRYDY EEVDADAAGK MMKDLETVMF DRSFVGKQLS AGDKVYTVEK ADNFEYNDPV
481   DGSVSRNQGL RLIFSDGSRI IFRLSGTGSA GATVRLYIDS YEKDAKKIHE DPQVMLAPLI SIALKLSQLH ERTGRTGPTC
561   YNIKARPGTK GKKPARRLFY CRVCRYKRNI NFICVFKNTA RYR
```

5.105 tensin - chicken (fragment)

Protein Accession	gi|2134419
Mean Expression Ratio	1.09
Median Expression Ratio	1.09
Credible Interval	(0.892, 1.34)
Associated Peptides	3
Associated Spectra	3
Coverage	NaN

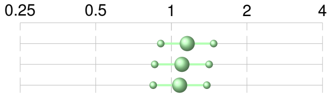

A	2.5	50	97.5	Sequence
**1**	**0.9**	**1.2**	**1.5**	**QVDTPDATR**
**1**	**0.86**	**1.1**	**1.4**	**AGNLPAAQPVGLEVPAR**
**1**	**0.84**	**1.1**	**1.4**	**YEANLALPQAPAR**

5.106 PREDICTED: hypothetical protein [Gallus gallus]

Protein Accession	gi|118083300
Mean Expression Ratio	1.09
Median Expression Ratio	1.09
Credible Interval	(0.877, 1.37)
Associated Peptides	2
Associated Spectra	2
Coverage	0.148

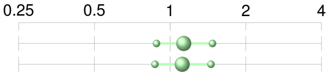

A	2.5	50	97.5	Sequence
**1**	**0.88**	**1.1**	**1.5**	**SAEEEEDEVDPK**
**1**	**0.87**	**1.1**	**1.5**	**SAEEEEDEVDPKR**

```
1       MKEKKEKLEE KAVHKEKKKE VVEDEENGAE EDEEENPEDV DEEEGGDEDD EGDENGQEQD GHAEKRSAEE EEDEVDPKRQ
81      KTENGSSA
```

5.107 PREDICTED: similar to insulin-degrading enzyme [Gallus gallus]

Protein Accession	gi|118092865
Mean Expression Ratio	0.917
Median Expression Ratio	0.915
Credible Interval	(0.71, 1.18)
Associated Peptides	1
Associated Spectra	1
Coverage	0.0105

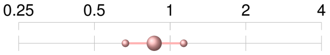

A	2.5	50	97.5	Sequence
**1**	**0.66**	**0.87**	**1.1**	**QEAISDEVIK**

```
1       MRQRLLTELR RALLGCARPW AAPTSQFAPQ RGFLGYGACV RRGNRAEGRA LREGPEVRPR LPRRVRTGAG ARCAAGAALT
81      LWRTRAARNV GLWGNREGSG GRPACVRVPC AAPPVPAGRA SAALLGRFFA LQKETNNKMN NPAIKRITNE IIKSPEDKRE
161     YRGLELANGI KALLISDPTT DKSSAALDVH IGSLSDPPNI AGLSHFCEHM LFLGTKKYPK ENEYSQFLSE HAGSSNAFTS
241     GEHTNYYFDV SHEHLEGALD RFAQFFLCPL FDESCKDREV NAVDSEHEKN LMNDAWRLFQ LEKATGNPNH PFSKFGTGNK
321     LTLETRPTKE GIDVRQELLK FHSTYYSSNL MAICVLGRES LDELTSLVVK LFSEVENKNV PVPEFPEHPF QEEHLRQLYK
401     VVPIKDIRNL YVTFPIPDLQ KYYKSNPGHY LGHLIGHEGP GSLLSELKAK GWVYTLVGGQ KEGARGFMFF IINVDLTEEG
481     LLHVEDIILH MFQYIQKLRI EGPQEWVFQE CKDLNAVAFR FKDKERPRGY TSKLGGMLHD LNAVAVRFKD KERPRGYTSK
561     LGGMLHYYPI EEVLAAEYLL EEFRPDLIEM VLDKLRPENI RVAIVSKSFE GKTDRTEDWY GTQYKQEAIS DEVIKKWQNA
641     DLNGKFKLPM KNEFIPTNFE ILPLEKDATQ YPALVKVRNY VQGNAVLELQ FQKYLLEKQH ASRGWFVYQQ RNEVHNNCGI
721     EIYYQTDMQS TSENMFLELF CQIISEPCFN TLRTKEQLGY IVFSGPRRAN GIQGLRFIIQ SEKPPHYLES RVEAFLKTME
801     KCIEDMTEEA FQKHIQALAI RRLDKPKKLS AECAKYWGEI ISQQYNFDRD NIEVAYLKTL TKDDIIQFYK VLLAVDAPRR
881     HKVSVHVLAR EMDSCPVVGE FPCQNDVNLA PAPPLPQPSV IENMTEFKRS LPLFPLVKPH INFMAAKL
```

5.108 chaperonin containing TCP1, subunit 7 [Gallus gallus]

Protein Accession	gi|71895883
Mean Expression Ratio	0.917
Median Expression Ratio	0.916
Credible Interval	(0.736, 1.15)
Associated Peptides	2
Associated Spectra	2
Coverage	0.0452

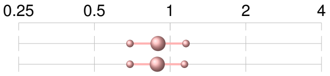

A	2.5	50	97.5	Sequence
**1**	**0.7**	**0.9**	**1.2**	**QVKPYVEEGLHPQIIIR**
**1**	**0.69**	**0.89**	**1.1**	**KEDKDEQR**

```
1       MMPTPVILLK EGTDTSQGIP QLVSNINACQ VIAEAVRTTL GPRGMDKLIV DDRGKATISN DGATILKLLD VVHPAAKTLV
81      DIAKSQDAEV GDGTTSVTLL AAEFLKQVKP YVEEGLHPQI IIRAFRTATQ LAVNKIKDIA VSVKKEDKDE QRSLLEKCAA
161     TALSSKLISQ SKEFFSKMVV DAVMMLDDLL QLKMIGIKKV QGGALEDSQL VAGVAFKKTF SYAGFEMQPK KYQSPKIALL
241     NVELELKAEK DNAEVRVNTV EDYQAIVDAE WNILYDKLDK IHKSGAKVVL SKLPIGDVAT QYFADRDMFC AGRVPEEDLK
321     RTMMACGGSI QTSVNALSDD VLGRCELFEE IQIGGDRYNF FTGCPKAKTC TIILRGGAEQ FMEETERSLH DAIMIVRRAI
401     KNDSVVAGGG AIEMELSKYL RDYSRTIPGK QQLLIGAYAK ALEIIPRQLC DNAGFDATNI LNKLRAKHAQ GGMWYGVDVN
481     NEDIADNFEA CVWEPAIVRI NALTAASEAA CLIVSVDETI KNPRSTVDAP PGGRGRGRGQ TPQPLRPRSV ALS
```

5.109 nestin [Gallus gallus]

Protein Accession	gi|45384298
Mean Expression Ratio	0.917
Median Expression Ratio	0.917
Credible Interval	(0.802, 1.05)
Associated Peptides	7
Associated Spectra	11
Coverage	0.0609

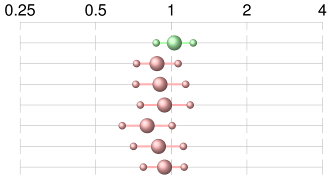

A	2.5	50	97.5	Sequence
3	0.87	1.0	1.2	ASSIEEAMPAAEGSSGSGEGTTGR
2	0.72	0.88	1.1	EALGEDDLQAGEALGAK
1	0.72	0.9	1.1	CAPVAFQPVEVEDYSK
1	0.75	0.94	1.2	QSLQVQIAQVLEDR
1	0.64	0.8	1	AGLDQELASFSQSLEGFR
1	0.71	0.89	1.1	FLEEENEGLR
2	0.77	0.94	1.1	ARDDEGEEEDKGR

```
1     MLSMEGFVGA RALGEESLQM WDLNKRLEAY LARVKFLEEE NEGLRAEIQS TKENPAGTHA GPGMRRSCGR SGCAAPRLHR
81    EVCGRAGRDN LYEEVQHVRS RCQKEQAARE EAKRQLSSSK KELEEERRAQ IWLKERAVQL EKEVEALLEV HEEEKAGLDQ
161   ELASFSQSLE GFRCAPVAFQ PVEVEDYSKR LSEIWRGAVE TYKAEVSQLE RALGQAKENL WQVAEDNQQS QLQLRHLEKE
241   LVGLKVRKEM LEESLGQQWQ EQHGEAEKFQ LAIEALEQEK QSLQVQIAQV LEDRQQLMHL KMSLSLEVAT YRTLLEAEST
321   RLQMPPGEFK LANSLRDVKL EASSSKHRAS LAAFPRPEGV AQLCRTPGDA LKVLTPKSKS SSALEFQKIS SVLQAPRTGE
401   PAAPSPTVPV LSPEPGSGGA ESPVHECGAG KESPMLSPLS PEQLVSHALQ DALKEMQDDA EAKEVPTLGA TQSTRDGDLE
481   ATMEEEEAAG TQGVGAEGET VSPPGLCLCS NEPTLLSATQ SDVESQEEMW EEERSKEEML NPLSSMESQE PGGERWGGVT
561   RGSRLQVGKE DMEATSTEAL HVSEKKEQRE IWSPSREDEE CEFPDEEREM QEEGSLQMEI EAACAVPVGS HPVLPTGIHL
641   QEDFLEREQE SEHQEMSLGE LGAAAGEERE QEVCQELKAS SIEEAMPAAE GSSGSGEGTT GRESTGRARD DEGEEEDKGR
721   EALGEDDLQA GEEREQEVCQ ELKASSIEEA MPAAEGSSGS GEGTTGREST GRARDDEGEE EDKGREALGE DDLQAGEALG
801   AKELGKESMG LEEAEGMWEE SVDLREEHRD LQEGHGDLQV EHEDLWEEQG DIQEEHGDTQ EEYGDTQEEH GDLQVEGGDL
881   QEEHGDTQEE HGDLQEEHGD TQEEHGDLQV EHEDLQVEHG DLQEEHGDLQ VEHEDLQVEH GDLQVEHEDL WEEHRDVQEE
961   HGDTQEEYGD TQEEHGDLQV EGGDLQEEHG DTQEEHEDLQ EEHGDTQEEH GDLQVEHGDL QEEHRDVQEG HGDLQVEHGD
1041  LQVEHGDLQE EHGDTQEEHG DLQEEHGDLQ EEHGDLQVEH EDLQVEHGDL QVEHGDLQEE HGDTQEEHRD LQEVHGDQQE
1121  EHRDLQEEHG DLQEGHGDLQ VEHGDLQVEH GDLQDEYGDT QEEHRDLQEV HGDQQEEHRD LQEGHGDLQE GHGDLWEEHG
1201  DLKEEHGDLQ EGHGDLQEES GDPQEEPGEP WVQHGEQGSA GDGLEQDMVL QPGEGAWGRE DNDISQKQQA QDWEGTAEDE
1281  EETGVNTITS QEPTQVDDNP HAEAAENEER DVTSPTAMEE TQEGEDEGDA GSEVQSQQQP QDTAGQEAEP APGQKEVRYG
1361  DTGEAPGDPQ EPAEALEVED EELSSEPIEL ERGSPDTAVM QQDLGNGAES DEPMEEDFQS EDAQLEEPKA CRMELEDTLL
1441  NSTPLCAYSG EMLESDPNPP SSGGDGEAAP EMAQEEEGDL RGSDEAAVHA EPESCEELSP APQCTEEEEG YFIVSAPSQE
1521  GSSMEEAENS EEFEEIKVEA AAERKDELTA PGVASLVPED EGHSEPVVGE AEDVKMPLGE FEMPKEEDEE DAGGFAAELE
1601  EGLTVPVAEG LSEGHIDKTT LGDEGLGEED VQDGDNPPAT ETSDTDPSST DPPPGTMLEH GAGMEAAEYL PDVPTQLPVD
1681  IMKDSDILEI VEQALEFNQE LVLGAKLAKD GQEEDGDAQP PQEEEGGSSP TSSCDEQPTV QEAVAEPERT KNGEQNGLHR
1761  QASLEDLAEF TEEGLNGITH PGEAPAAHTL PLPSKHSGAE PVPELSPLQT TSCARSRSPG PLGRSDAVGP HSPATIGRLP
```

5.110 thrombospondin 2 [Gallus gallus]

Protein Accession	gi|48976107
Mean Expression Ratio	1.09
Median Expression Ratio	1.09
Credible Interval	(0.846, 1.40)
Associated Peptides	1
Associated Spectra	1
Coverage	0.0110

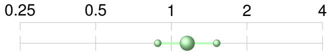

A	2.5	50	97.5	Sequence
1	0.88	1.2	1.5	YNPEQEDSDGDGR

```
1     MLQRSRLLWL AVFITLWVSS DAQDDAKEEE NTFDLLQISN INRKTIGAKL FRGPDPAIPA YRFIRFDHIP PFKPEKLKKI
81    VKLIRQNEGF ILSATLRQDR QSRGTILALE GPGISERQFE IISNGRANTL DLIYWVDGFQ NVISLEDVDL ADSQWKNLTV
161   QVTGENYNLY VGCDLIDSFI LEEPFYEQLK AENSRMYVAK GSIRENHFRG LLQNIHLIFD TSIEDVLRKK GCQRSQSTEV
241   NTINESTEIL HLSPAVTTEY VGEKTEKKAE FCDRSCEELG TMFTELTGLR IVVNNLADNL QKVSEENQIM WELIGPNKTL
321   KNQSVCWQDG RVFADSESWI VDSCTKCTCQ DSKIVCHQIT CPPVSCADPS FIEGECCPVC SHSDDSEEGW SPWSDWTKCS
401   VTCGSGTQMR GRSCDVTRSA CTGPHIQTRM CSFKKCDHRI RQDGGWSHWS PWSSCSVTCG VGNITRIRLC NSPIPQMGGK
481   NCVGNGRETE KCEKAPCPVN GQWGPWSPWS ACTVTCGGGI RERSRLCNSP EPQYGGKPCV GDTKQHDMCN KRDCPIDGCL
561   SNPCFPGAEC NSYPDGSWSC GPCPAGFLGN GTVCEDLDEC IAVSDVCFKV NQVHRCVNTN PGFHCLPCPP RYKGSQPYGV
641   GLEVAKTEKQ VCEPENPCKD KTHSCHKSAE CIYLGHFSDP MYKCECRTGY AGDGRICGED SDLDGWPNNN LVCAANATYH
721   CVKDNCPLLP NSGQEDFDKD GKGDACDEDD DNDGVEDDKD NCPLLFNPRQ FDYDKDEVGD RCDNCPYVHN PAQIDTDNNG
801   EGDSCAVDID GDDIFNERDN CPYVYNTDQS DTDGDGVGDQ CDNCPLMHNP DQTDADNDLV GDQCDNNEDI DEDGHQNNQD
881   NCPYIPNANQ ADHDKDGKGD ACDPDDDNDG IPDDRDNCRL RYNPEQEDSD GDGRGDICKD DFDDDNVPDI FDVCPENNAI
961   SETDFRKFQM VPLDPKGTAQ IDPNWVIRHQ GKELVQTANS DPGIAVGYDE FSSVDFSGTF YVNTDRDDDY AGFVFGYQSS
1041  SRFYVLMWKQ VTQTYWEDKP TRAYGYSGVS LKVVNSTTGT GEHLRNALWH TGNTPGQVRT LWHDPKNIGW KDYTAYRWHL
1121  IHRPKTGLIK VLVYEGKQVM VDSGPIYDTT FAGGRLGLFV FSQEMVYFSD LKYECRDA
```

5.111 PREDICTED: similar to serotonin receptor [Gallus gallus]

Protein Accession	gi|118090882
Mean Expression Ratio	1.09
Median Expression Ratio	1.09
Credible Interval	(0.844, 1.41)
Associated Peptides	1
Associated Spectra	1
Coverage	0.0211

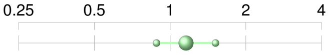

A	2.5	50	97.5	Sequence
**1**	**0.88**	**1.2**	**1.5**	**MLLRGSPRR**

```
1       MLLRGSPRRF LEHHLLFVEN AEQQYPARES LPNPFMTEEP SVPAEPDLPS SNLTNATDCG EEILLYGDTE KIVIGAVLSI
81      IILMTIAGNG LVIISVCIVK KLRQPSNYLV VSLAAADLSV AFAVMPFVTI TDLVGGEWLF GKVFCNVFIA MDVMCCTASI
161     MTLCIISVDR YLGITRPLTY PVRQNGKLMA KMVFIVWLLS ASITLPPLFG WAKNVTVERV CLISQDFGYT VYSTGVAFYI
241     PMAVMLVMYS RIYKAAKVSA EKHRFMNFSK HYEEEGVYCL EASSRGHPSS KRTKAVEECA TLSKLLRQDR KNISIFKREQ
321     KAARTLGIIV GAFTFCWFPF FLMSTARPFI CGIHCSCLPL RLERTLLWLG YTNSLINPLI YAFFNRDLRT TFWNLLRCRY
401     RNINRRLSAA SMHEALKATE RHECIL
```

5.112 t-complex 1 [Gallus gallus]

Protein Accession	gi|57530301
Mean Expression Ratio	0.92
Median Expression Ratio	0.918
Credible Interval	(0.711, 1.19)
Associated Peptides	1
Associated Spectra	1
Coverage	0.0233

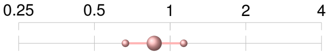

A	2.5	50	97.5	Sequence
**1**	**0.66**	**0.87**	**1.1**	**AFHNEAQVNPDRK**

```
1       MAAMEGPLAV FGERTSGDTI RTQNVTAASA IANIVKSSLG PVGLDKMLVD EIGDVTITND GATILKLLEV EHPAAKVLCE
81      LADLQDKEVG DGTTSVVIIA AELLKNADEL VKQKIHPTSI IGGYRLACKE AVRYINENLI INTDELGREC LINAAKTSMS
161     SKIIGIDGDF FANMVVDAAM AVKYTDQKGQ ARYPINSVNV LKAHGRSQKE SILVNGYALN CVVGSQGMTK RIVNAKIACL
241     DFSLQKAKMK LGVQVVITDP EKLDQIRQRE SDITKERIQK ILATGANVIL TTGGIDDMCL KYFVDAGAMA VRRVVKKDLK
321     RIAKASGATI CSTLANLEGE ESFEALMLGQ AEEVIQERIC DDELILIKNT KARTSASIIL RGANDFMCDE MERSIHDALC
401     VVKRVLESKS VVPGGAVEA ALSIYLENYA TSMGSREQLA IAEFARSLLI IPNTLAVNAA QDATDLVAKL RAFHNEAQVN
481     PDRKNLKWIG LDLVNGKPRD NKQAGVFEPT MVKTKSLKFA TEAAITILRI DDLIKLHPEP KEDRGCYEDA VHSGALEE
```

5.113 DEAD/H (Asp-Glu-Ala-Asp/His) box polypeptide 3 [Gallus gallus]

Protein Accession	gi|71895253
Mean Expression Ratio	0.919
Median Expression Ratio	0.918
Credible Interval	(0.713, 1.19)
Associated Peptides	1
Associated Spectra	1
Coverage	0.0138

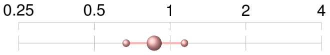

A	2.5	50	97.5	Sequence
**1**	**0.67**	**0.87**	**1.1**	**YLVLDEADR**

```
1       MSHVAVENAL SLDQQFSGLD LNSSDSQSEG SATSKGRYIP PHLRNREASK QGFDSGGWST SRDKDAYSSF GARSDRGAKS
81      SFFDRGNGSR GGRYEERGRG SDYDRSGFGR FDRGGNSRWC DKSDEDDWSK PLPPSERLEQ ELFSGSNTGI NFEKYDDIPV
161     EATGSNCPPH IESFSDVDMG EIIMGNIELT RYTRPTPVQK HAIPIIKEKR DLMACAQTGS GKTAAFLLPI LSQIYADGPG
241     DALRAMKENG RYGRRKQYPI SLVLAPTREL AVQIYEEARK FAYRSRVRPC VVYGGADIGQ QIRDLERGCH LLVATPGRLV
321     DMMERGKIGL DFCKYLVLDE ADRMLDMGFE PQIRRIVEQD TMPPKGVRHT MMFSATFPKE IQMLARDFLD EYIFLAVGRV
401     GSTSENITQK VVWVEELDKR SFLLDLLNAT GKDSLTLVFV ETKKGADALE DFLYHEGYAC TSIHGDRSQR DREEALHQFR
481     SGKSPILVAT AVAARGLDIS NVKHVINFDL PSDIEEYVHR IGRTGRVGNL GLATSFFNER NINITKDLLD LLVEAKQEVP
561     SWLENMAYEQ HHKGGGSRGR SKSRFTGGFG ARDYRTSSGA GSSSFSSSRP ASGRTGGSGS RGFGGGGYGG FYNSDGYGGN
641     YNSQGVDWWG N
```

5.114 PREDICTED: similar to nuclear poly(C)-binding protein, splicevariant E, partial [Gallus gallus]

Protein Accession	gi|118116443
Mean Expression Ratio	1.09
Median Expression Ratio	1.09
Credible Interval	(0.862, 1.38)
Associated Peptides	1
Associated Spectra	2
Coverage	0.104

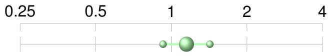

A	2.5	50	97.5	Sequence
2	0.93	1.2	1.4	ESTGAQVQVAGDMLPNSTER

```
1       MDTGVIEGGL NVTLTIRLLM HGKEVGSIIG KKGESVKKMR EESGARINIS EGNCPERIIT LAGPTNAIFK AFAMIIDKLE
81      EDISSSMTNS TAASRPPVTL RLVVPASQCG SLIGKGGCKI KEIRESTGAQ VQVAGDMLPN STERAITIAG IPQSIIECVK
161     QICVVMLESP PKGVTIPYRP KPSSSPVIFA GGQ
```

5.115 seryl-tRNA synthetase [Gallus gallus]

Protein Accession	gi|71897227
Mean Expression Ratio	0.92
Median Expression Ratio	0.92
Credible Interval	(0.711, 1.19)
Associated Peptides	1
Associated Spectra	1
Coverage	0.0292

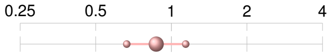

A	2.5	50	97.5	Sequence
1	0.66	0.87	1.1	KLDLEAWFPGSGAFR

```
1       MVLDLDLFRA DKGGDPAAVR EMQRKRFKDP ALVDALVRAD GAWRRCRFRA DNLNKLKNLC SKTIGDKMKK KEPVGSDESV
81      PESAQNLDEL TADVLGGLQV SQIKKVRLLI DEAILECDAE RVRLEAERFE SLREIGNLLH PSVPISNDED ADNKVERIWG
161     DCSCRKKYSH VDLVVMVDGY EGEKGAVVAG SRGYFLKGPL VFLEQALIQY ALQSLRAKGY TPVYTPFFMR KEVMQEVAQL
241     SQFDEELYKV IGKGSEKAED SSVDEKYLIA TSEQPIAALH RDEWLKPEDL PIKYAGLSTC FRQEVGSHGR DTRGIFRVHQ
321     FEKIEQFVYA SPHDNKSWEM FDEMIATAEE FYQSLGIPYH IVNIVSGALN HAASKKLDLE AWFPGSGAFR ELVSCSNCTD
401     YQARRLRIRF GQTKKMMDKV EFVHMLNATM CATTRTICAI LENYQTEEGI VVPERLRDFM PPDLRQIIRF VKPAPIEQEL
481     SKKQKKQQEG GRKKAAGGER VLEEQMQNMG VSSA
```

5.116 Cofilin-2 (Cofilin, muscle isoform)

Protein Accession	gi|17433708
Mean Expression Ratio	0.92
Median Expression Ratio	0.92
Credible Interval	(0.753, 1.12)
Associated Peptides	3
Associated Spectra	3
Coverage	0.277

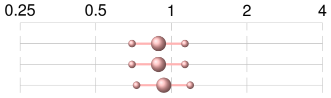

A	2.5	50	97.5	Sequence
1	0.7	0.89	1.1	QILVGDIGDTVEDPYTAFVK
1	0.7	0.89	1.1	KEDLVFIFWAPESAPLK
1	0.73	0.93	1.2	KSSTPEEIK

```
1       MASGVTVNDE VIKVFNDMKV RKSSTPEEIK KRKKAVLFCL SDDKKQIIVE EAKQILVGDI GDTVEDPYTA FVKLLPLNDC
81      RYALYDATYE TKESKKEDLV FIFWAPESAP LKSKMIYASS KDAIKKKFTG IKHEWQVNGL DDIKDRSTLG EKLGGNVVVS
161     LEGKPL
```

5.117 PREDICTED: hypothetical protein [Gallus gallus]

Protein Accession	gi|118092623
Mean Expression Ratio	0.92
Median Expression Ratio	0.92
Credible Interval	(0.741, 1.14)
Associated Peptides	2
Associated Spectra	2
Coverage	0.069

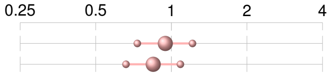

A	2.5	50	97.5	Sequence
1	0.73	0.94	1.2	NVFNMVVEVPR
1	0.66	0.85	1.1	YVANVFPHK

```
1      MAGYSVEERA APNSLEYRLF FKDAAGRYIS PFHDIPLYAD AGKNVFNMVV EVPRWTNAKM EIATKDPLNP IKQDVKKGKL
81     RYVANVFPHK GYIWNYGAIP QTWEDPGHKD ENTGCCGDND PIDVCEIGSK VCSRGEVIKV KVLGTLALID EGETDWKIIA
161    INVEDPEAEN YNDINDVRRM KPGYLEATVD WFRRYKVPDG KPENQFAFNG EFKDKDFAVN VIKSTHEHWK ALIAKKTDGG
241    EISCTNLTVS GSPFCCSQEC AKATVDAAPP CKAANPIPPE VDRWFYYQKN
```

5.118 tyrosyl-tRNA synthetase [Gallus gallus]

Protein Accession	gi|57530465
Mean Expression Ratio	0.92
Median Expression Ratio	0.921
Credible Interval	(0.711, 1.19)
Associated Peptides	1
Associated Spectra	1
Coverage	0.0209

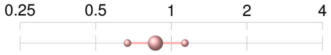

A	2.5	50	97.5	Sequence
1	0.67	0.87	1.1	NSEPETIVPSR

```
1      METASGPQEK YQLITRNLQE VLGEDKLMAI LKEREVKIYW GTATTGKPHV AYFVPMSKIA DFLKAGCEVT ILFADLHAYL
81     DNMKAPWELL ELRTRYYEHV IKAMLESIGV PLEKLKFIRG TDYQLSKEYT LDVYRLSSVV TQHDAKKAGA EVVKQVEHPL
161    LSGLLYPGLQ ALDEEYLKVD AQFGGVDQRK IFTFAEKYLP SLGYAKRIHL MNPMVPGLTG SKMSSSEEDS KIDLLDRKED
241    VKKKLKKAFC EPGNIENNGV LSFIKHVLFP LKSEFVILRE EKWGGNKTYT AYETLEKDFA EQVVHPGDLK NSVEAALNKL
321    LDPIREKFNS PELKKLTNAA YPNPSKAKPA EKGTKNSEPE TIVPSRLDIR VGKVVSVEKH PDADSLYVEK IDVGEPEPRT
401    VVSGLVQFVP KEQLQDRLVV LLCNLKPQKM RGVESQGMVL CASSVGEPRQ VEPLDPPAEC CAGERVYVEG YEDGEPDDEL
481    KPKKKVFEKL QADFRISEDC IAQWKERNFL TKLGSISCKS LKGGSIS
```

5.119 Chain B, Smooth Muscle Myosin Motor Domain-Essential Light Chain Complex With Mgadp.Alf4 Bound At T

Protein Accession	gi|5542589
Mean Expression Ratio	1.09
Median Expression Ratio	1.09
Credible Interval	(0.839, 1.40)
Associated Peptides	1
Associated Spectra	1
Coverage	0.1

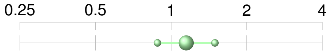

A	2.5	50	97.5	Sequence
1	0.88	1.1	1.5	NKDQGCFEDYVEGLR

```
1      CDFSEEQTAE FKEAFQLFDR TGDGKILYSQ CGDVMRALGQ NPTNAEVMKV LGNPKSDEMN LKTLKFEQFL PMMQTIAKNK
81     DQGCFEDYVE GLRVFDKEGN GTVMGAEIRH VLVTLGEKMT EEEVEQLVAG HEDSNGCINY EELVRMVLSG
```

5.120 PREDICTED: similar to leucine-rich repeat-containing F-box protein FBL3A [Gallus gallus]

Protein Accession	gi|118084729
Mean Expression Ratio	0.922
Median Expression Ratio	0.922
Credible Interval	(0.714, 1.19)
Associated Peptides	1
Associated Spectra	1
Coverage	0.0277

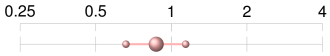

A	2.5	50	97.5	Sequence
**1**	**0.66**	**0.87**	**1.1**	**DVLGRVGMTCPR**

```
1       MKRGRKTNEA NNSSSEETAE KESKRHKSVD ERTTVVRSPD WGNLLQDIIL QVFQYLPLLD RAHASQVCRS WNQVFHMPDL
81      WRCFEFELNQ PATSYLRATH PELIKQIIKR HSNHLQYVSF KVDSSKESAE AACDILSQLV NCSLKTLGLI STARPSFMDL
161     PKSHFISALT VVFVNSKSLS SLKIDDTPVD DPSLKVLVAN NSDTLKLLKM SSCPHVSPAG ILCVADQCHG LRELALNYHL
241     LSDELLLALS SEKHVRLEHL RIDVVSENPG QTQFHTIQKS SWDAFIKHSP KVNLVMYFFL YEEEFDPFFR YEIPVTHLYF
321     GRSVSKDVLG RVGMTCPRLV ELVVCANGLR PLDEELIRIA ERCKYLSAVG LGECEVSCSA FVEFVKMCGG RLSQLSIMEE
401     VLIPDQKYSL EQIHWEVSKH LGRVWFPDMM PTW
```

5.121 PREDICTED: hypothetical protein, partial [Gallus gallus]

Protein Accession	gi|118114292
Mean Expression Ratio	1.09
Median Expression Ratio	1.08
Credible Interval	(0.903, 1.3)
Associated Peptides	2
Associated Spectra	8
Coverage	0.241

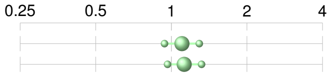

A	2.5	50	97.5	Sequence
**4**	**0.94**	**1.1**	**1.3**	**QITISNCGQLS**
**4**	**0.97**	**1.1**	**1.3**	**FADENFILK**

```
1       FADENFILKH TGPGILSMAN AGPNTNGSQF FICTAKTEWL DGKHVVFGRV KEGMNVVEAM ERCGSKDGKT SKQITISNCG
81      QLS
```

5.122 epsilon globin [Gallus gallus]

Protein Accession	gi|52138683
Mean Expression Ratio	0.921
Median Expression Ratio	0.922
Credible Interval	(0.749, 1.13)
Associated Peptides	2
Associated Spectra	4
Coverage	0.184

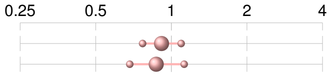

A	2.5	50	97.5	Sequence
**3**	**0.77**	**0.92**	**1.1**	**FFDNFGNLSSPTAIIGNPK**
**1**	**0.68**	**0.87**	**1.1**	**LSELHCEK**

```
1       MVHWSAEEKQ LITSVWSKVN VEECGAEALA RLLIVYPWTQ RFFDNFGNLS SPTAIIGNPK VRAHGKKVLS SFGEAVKNLD
81      NIKNTYAKLS ELHCEKLHVD PENFRLLGNI LIIVLAAHFT KDFTPTCQAV WQKLVSVVAH ALAYKYH
```

5.123 Nuclease sensitive element-binding protein 1 (Y-box-binding protein 1) (Y-box transcription factor)

Protein Accession	gi|465508
Mean Expression Ratio	0.923
Median Expression Ratio	0.922
Credible Interval	(0.757, 1.12)
Associated Peptides	3
Associated Spectra	3
Coverage	0.162

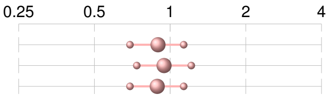

A	2.5	50	97.5	Sequence
1	0.7	0.9	1.1	TAEPPAENTSAPEAEQGGAE
1	0.74	0.95	1.2	SVGDGETVEFDVVEGEK
1	0.7	0.89	1.1	NEGAENIPEGQAQQR

```
1     MSSEAETQPP AAPVPAAPAA APADSKPNGG SGNGSSGLAS AAPPAGGDKK VIATKVLGTV KWFNVRNGYG FINRNDTKED
81    VFVHQTAIKK NNPRKYLRSV GDGETVEFDV VEGEKGAEAA NVTGPGGVPV QGSKYAADRN HYRRYPRRRG PPRNYQQNYQ
161   NSESGEKNEG AENIPEGQAQ QRRPYRRRRY PPYYMRRPYG RRPQYSNPPV QGEIVEGADN QGAGEQGRPV RQNMYRGYRP
241   RFRRGPPRQR QPREDGNEED KENQGDETQG QQPPQRRYRR NFNYRRRRPE NPKPQDGKET KTAEPPAENT SAPEAEQGGA
321   E
```

5.124 tyrosine 3/tryptophan 5 -monooxygenase activation protein, epsilon polypeptide [Homo sapiens]

Protein Accession	gi|5803225
Mean Expression Ratio	1.08
Median Expression Ratio	1.08
Credible Interval	(0.925, 1.27)
Associated Peptides	5
Associated Spectra	7
Coverage	0.282

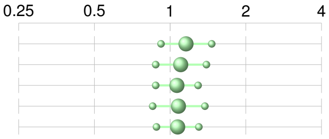

A	2.5	50	97.5	Sequence
1	0.92	1.2	1.5	EALQDVEDENQ
1	0.88	1.1	1.4	DNLTLWTSDMQGDGEEQNK
2	0.87	1.1	1.3	AAFDDAIAELDTLSEESYK
1	0.85	1.1	1.4	YLAEFATGNDRK
2	0.88	1.1	1.3	EENKGGEDKLK

```
1     MDDREDLVYQ AKLAEQAERY DEMVESMKKV AGMDVELTVE ERNLLSVAYK NVIGARRASW RIISSIEQKE ENKGGEDKLK
81    MIREYRQMVE TELKLICCDI LDVLDKHLIP AANTGESKVF YYKMKGDYHR YLAEFATGND RKEAAENSLV AYKAASDIAM
161   TELPPTHPIR LGLALNFSVF YYEILNSPDR ACRLAKAAFD DAIAELDTLS EESYKDSTLI MQLLRDNLTL WTSDMQGDGE
241   EQNKEALQDV EDENQ
```

5.125 PREDICTED: similar to ionized calcium binding adapter molecule 2 (Iba2) [Gallus gallus]

Protein Accession	gi|50757279
Mean Expression Ratio	0.924
Median Expression Ratio	0.924
Credible Interval	(0.73, 1.17)
Associated Peptides	1
Associated Spectra	2
Coverage	0.103

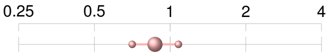

A	2.5	50	97.5	Sequence
2	0.71	0.87	1.1	ANESNPKPSGPPPER

```
1     MAAPRRPSGG GVRRAPQDGR LEEINKEFLC DPKFSDEEDL EEKLAVFKEK YMEFDLNNQG EIDLMSVKRM MEKMGVPKTH
81    LELKKMISEV TGGVSETISY QDFVNVMLGK RSAVLKLVMM FEGKANESNP KPSGPPPERD IASLP
```

5.126 PREDICTED: similar to cystathionine beta-synthase [Gallus gallus]

Protein Accession	gi|118083958
Mean Expression Ratio	0.926
Median Expression Ratio	0.926
Credible Interval	(0.732, 1.17)
Associated Peptides	1
Associated Spectra	2
Coverage	0.0179

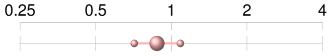

A	2.5	50	97.5	Sequence
2	0.71	0.88	1.1	CVVIFPDSIR

```
1     MEKLMSEKPV PVSKAKPDTN SCPHASGKYF LPGGTDTEDC KTNDKERKWI RPDTPSKCTW KLGKPISASP HRHMSLPEPP
81    NILPNILNKV GNTPLVRINK IGKYFGLKCE LLAKCEYFNA GGSVKDRISL RMVEDAERAG ILKPGDTIIE PTSGNTGIGL
161   ALAAAVKGYR CIIVMPEKMS MEKVDVLRAL GAEIVRTPTT ARFDSPESHV GVAWRLKNEI PNAHILDQYR NASNPLTHYD
241   TTAEEILQQC EGKIDMLVAT AGTGGTITGI SRKLKEKCPG CKIIGVDPEG SILAQPDELN KTDKTMYEVE GIGYDFVPTV
321   LDRSLVDQWY KSNDEESFAL ARMLIREEGL LCGGSSGSAM SVAVKAAKEL KEGQRCVVIF PDSIRNYMSK FLSDKWMIQK
401   GFMTEEDLVK KPWWWNVSVQ ELSLSAPLTV LPTVTCAKTV EILREKGFDQ VPVVDESGVI LGMVTLGNML SSLLAGKVQP
481   SDEVSKVIYK QFKQINLQDN LGRLSHILET DHFALVVHEQ IQYHTDGSSS KRQMVFGIVT AIDLLNFVTA RERERKIN
```

5.127 heat shock 70kDa protein 9B [Gallus gallus]

Protein Accession	gi|57524986
Mean Expression Ratio	1.08
Median Expression Ratio	1.08
Credible Interval	(0.86, 1.35)
Associated Peptides	2
Associated Spectra	2
Coverage	0.0356

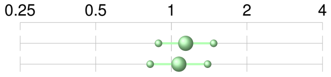

A	2.5	50	97.5	Sequence
1	0.89	1.1	1.5	LLGQFTLVGIPPAPR
1	0.83	1.1	1.4	RFDDSEVKK

```
1     MISASRAAAR LPLLLPRGGP VPAVPGLAQT FWNGLSQNVL RAASSRKYAS EAIKGAVIGI DLGTTNSCVA VMEGKQAKVL
81    ENSEGARTTP SVVAFTADGE RLVGMPAKRQ AVTNPHNTFY ATKRLIGRRF DDSEVKKDIK NVPFKIVRAS NGDAWVEAHG
161   KLYSPSQIGA FVLMKMKETA ENYLGHPAKN AVITVPAYFN DSQRQATKDA GQISGLNVLR VINEPTAAAL AYGLDKSEDK
241   IIAVYDLGGG TFDISILEIQ KGVFEVKSTN GDTFLGGEDF DQALLQYIVK EFKRETSVDL TKDNMALQRV REASEKAKCE
321   LSSSVQTDIN LPYLTMDASG PKHLNMKLSR SQFEGIVADL IKRTVAPCQK AMQDAEVSKS DIGEVILVGG MTRMPKVQQT
401   VQDLFGRAPS KAVNPDEAVA IGAAIQGGVL AGDVTDVLLL DVTPLSLGIE TLGGVFTKLI NRNTTIPTKK SQVFSTAADG
481   QTQVEIKVCQ GEREMASDNK LLGQFTLVGI PPAPRGVPQI EVTFDIDANG IVHVSAKDKG TGREQQIVIQ SSGGLSKDEI
561   ENMVKNAEKY AEEDRRRKER VEAVNLAEGI IHDTESKMEE FKDQLPADEC NKLKEEIAKM RELLARKDTE TGENIRQAAT
641   SLQQASLKLF EMAYKKMASE RESSGSSGDQ KEEKQ
```

5.128 PREDICTED: similar to pDJA1 chaperone [Gallus gallus]

Protein Accession	gi|118095620
Mean Expression Ratio	1.08
Median Expression Ratio	1.08
Credible Interval	(0.874, 1.34)
Associated Peptides	2
Associated Spectra	3
Coverage	0.0678

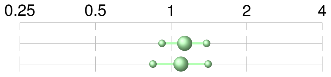

A	2.5	50	97.5	Sequence
2	0.92	1.1	1.4	NSGGEAYEEDEEGPR
1	0.84	1.1	1.4	YHPDKNPSEGER

```
1     MVKETEYYDI LQVKPTASSE EIKRAYRKLA LKYHPDKNPS EGERFKLISQ AYEVLSDPKK RDLYDQGGEQ AIKEGGLSGG
81    SFSSPMDIFD MFFGGGGRMN RERRGKNVVH QLGVSLEDLY NGVTRKLALQ KNVICGKCEG YGGKRGAVEK CPVCKGRGMQ
161   VLVQQIGPGM VQQIQTVCPE CKGQGERINP KDRCDNCNGC KVVREKKIIE VHVDKGMKDG QKIVFHGEGD QEPDLEPGDV
241   IIVLDQKDHG VFQRRGHDLV TKMRIQLSEA LCGFKKTIET LDNRVLVISS RPGEVIKHGD LKCIYNEGMP IYKSPMDKGS
321   LIIQFLVQFP EQHWLPREKL NMLEALLPPR EDVMITDEMD QVDLEDFDPS EQTYRNSGGE AYEEDEEGPR TGVQCQTS
```

5.129 S100 calcium binding protein A11 [Gallus gallus]

Protein Accession	gi|45384028
Mean Expression Ratio	1.08
Median Expression Ratio	1.08
Credible Interval	(0.837, 1.39)
Associated Peptides	1
Associated Spectra	1
Coverage	0.109

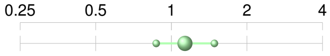

A	2.5	50	97.5	Sequence
**1**	**0.87**	**1.1**	**1.5**	**CIESLLAVFQR**

```
1       MSKVSPTETE RCIESLLAVF QRYAGREGDN LKLSKKEFRT FMNTELASFT KNQKDPAVVD RMMKRLDINS DGQLDFQEFL
81      NLIGGIAVAC HDALLVQPPH P
```

5.130 PREDICTED: hypothetical protein, partial [Gallus gallus]

Protein Accession	gi|118115605
Mean Expression Ratio	0.926
Median Expression Ratio	0.927
Credible Interval	(0.718, 1.19)
Associated Peptides	1
Associated Spectra	1
Coverage	0.0469

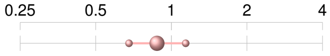

A	2.5	50	97.5	Sequence
**1**	**0.68**	**0.88**	**1.1**	**KEFLHAQEEVKR**

```
1       MAPHAEDLPA LPRPHPALPF LGPEAEDLED LYSRYKKLQQ ELEFLEVQEE YIKDEQKNLK KEFLHAQEEV KRIQSIPLVI
81      GQFLEAVDQN TAIVGSTTGS NYYVRILSTI DRELLKPLWG SNYYVRILST IDRELLKPLW GPYGVPLGSL WGTFYTGPFW
161     GRYGVLMGSV CGSGSNYYVR ILSTIDRELL KPLWGPYGVL MGSLWGTFYT GPFWGRYGVL MGSVCGSGSN YYVRILSTID
241     RELLKPLWGP YGVLMG
```

5.131 PREDICTED: similar to hepatocellular carcinoma susceptibility protein [Gallus gallus]

Protein Accession	gi|118086767
Mean Expression Ratio	1.08
Median Expression Ratio	1.08
Credible Interval	(0.854, 1.38)
Associated Peptides	1
Associated Spectra	2
Coverage	0.0378

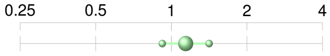

A	2.5	50	97.5	Sequence
**2**	**0.92**	**1.1**	**1.4**	**DDEQLLGTPLR**

```
1       MRAELRGAAS ISPCGLAGMF VPCDGGGLDF EGFTLLMPAV SVGNVGQLAV DLVISTLRMP KVGYFYTDCL VPMVGNNPYA
81      TTKENSMELS INAEVYSLPS KKLVVLQIRS PFIKNKYRPF CQTLLSWVES SKCARVILLS SSHAYQRDDE QLLGTPLRYL
161     LTPALEKAVE GHIQELKWKE MEKVAAYPGI SDAEKVLHIP GGGITKLLFT ESCSKGIHMA VLLKFCSEGD NIPDAFGLVN
241     YLNEWLQLIK IRRNSSTDAS PEREIPDPSS LWKIPSSWRL LFGNGLPPAL F
```

5.132 talin 1 [Gallus gallus]

Protein Accession	gi|45383127
Mean Expression Ratio	0.927
Median Expression Ratio	0.927
Credible Interval	(0.798, 1.08)
Associated Peptides	6
Associated Spectra	8
Coverage	0.0354

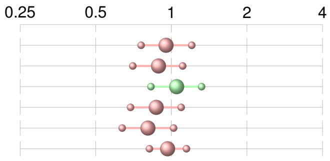

A	2.5	50	97.5	Sequence
1	0.76	0.96	1.2	EQGIDDNETLLLR
1	0.7	0.89	1.1	EVDQASLAAISQQLAPR
1	0.83	1.0	1.3	QELAVFSSPVPPAQVSTPEDFIR
1	0.69	0.87	1.1	FFYSDQNVDSR
1	0.64	0.8	1.0	IGITNYDEYSLVR
3	0.82	0.97	1.1	AVAKPGDPDSQQR

```
1     MVALSLKISI GNVVKTMQFE PSTMVYDACR MIRERVPEAQ MGQPNDFGLF LSDEDPKKGI WLEAGKALDY YMLRNGDTME
81    YKKKQRPLKI RMLDGTVKTV MVDDSKTVTD MLTTICARIG ITNYDEYSLV REIMEEKKEE VTGTLKKDKT LLRDEKKMEK
161   LKQKLHTDDE LNWLDHGRTL REQGIDDNET LLLRKFFYS DQNVDSRDPV QLNLLYVQAR DDILNGSHPV SFDKACEFAG
241   YQCQIQFGPH NEQKHKPGFL ELKDFLPKEY IKQKGERKIF MAHKNCGNMS EIEAKVRYVK LARSLKTYGV SFFLVKEKMK
321   GKNKLVPRLL GITKECVMRV DEKTKEVIQE WSLTNIKRWA ASPKSFTLDF GDYQDGYYSV QTTEGEQIAQ LIAGYIDIIL
401   KKKKSKDHFG LEGDEESTML EDSVSPKKST VLQQQFNRVG KAELGSVALP AIMRTGAGGP ENFQVGTMPQ AQMQITSGQM
481   HRGHMPPLTS AQQALTGTIN SSMQAVNAAQ ATLDDFETLP PLGQDAASKA WRKNKMDESK HEIHSQADAI TAGTASVVNL
561   TAGDPADTDY TAVGCAVTTI SSNLTEMSKG VKLLAALMED EGGNGRQLLQ AAKNLASAVS DLLKTAQPAS AEPRQNLLQA
641   AGLVGQTSGE LLQQIGESDT DPRFQDMLMQ LAKAVASAAA ALVLKAKNVA QKTEDSALQT QVIAAATQCA LSTSQLVACT
721   KVVAPTISSP VCQEQLIEAG KLVAKSAEGC VEASKAATND DQLLKQVGVA ATAVTQALND LLQHIKQHAT GGQPIGRYDQ
801   ATDTILNVTE NIFSSMGDAG EMVRQARILA QATSDLVNAI KADAEGETDL ENSRKLLSAA KILADATAKM VEAAKGAAAH
881   PDSEEQQQRL REAAEGLRMA TNAAAQNAIK KKLVHKLEHA AKQAAASATQ TIAAAQHAAA SNKNPAAQQQ LVQSCKVVAD
961   QIPMLVQGVR GSQSQPDSPS AQLALIAASQ NFLQPGGKMV AAAKATVPTI TDQASAMQLS QCAKNLAAAL AELRTAAQKA
1041  QEACGPLEID SALGLVQSLE RDLKEAKAAA RDGKLKPLPG ETMEKCAQDL GNSTKAVTSA IAHLLGEVAQ GNENYTGIAA
1121  REVAQALRSL SQAARGVAAN SSDPQAQNAM LECASDVMDK ANNLIEEARK AVAKPGDPDS QQRLVQVAKA VSQALNRCVN
1201  CLPGQRDVDA AIRMVGEASK RLLSDSFPPS NKTFQEAQSQ LNRAAAGLNQ SANELVQASR GTPQDLAKSS GKFGQDFNEF
1281  LQAGVEMASL SPTKEDQAQV VSNLKSISMS SSKLLLAAKA LSADPTSPNL KSQLAAAARA VTDSINQLIT MCTQQAPGQK
1361  ECDNALRELE TVKELLENPT QTVNDMSYFS CLDSVMENSK VLGESMAGIS QNAKNSKLPE FGESISAASK ALCGLTEAAA
1441  QAAYLVGVSD PNSQAGQQGL VDPTQFARAN QAIQMACQNL VDPACTQSQV LSAATIVAKH TSALCNTCRL ASSRTANPVA
1521  KRQFVQPAKE VANSTANLVK TIKALDGAFN EENRERCRAA TAPLIEAVDN LTAFASNPEF ATVPAQISPE GRRAMEPIVT
1601  SAKTMLESSA GLIQTARSLA VNPKDPPQWS VLAGHSRTVS DSIKKLITNM RDKAPGQREC DEAIDVLNRC MREVDQASLA
1681  AISQQLAPRE GISQEALHNQ MITAVQEINN LIEPVASAAR AEASQLGHKV SQMAQYFEPL ILAAIGAASK TPNHQQQMNL
1761  LDQTKTLAES ALQMLYTAKE AGGNPKQAAH TQEALEEAVQ MMKEAVEDLT TTLNEAASAA GVVGGMVDSI TQAINQLDEG
1841  PMGEPEGTFV DYQTTMVKTA KAIAVTVQEM VTKSTTNPDE LGILANQLTN DYGQLAQQAK PAALTAENEE IGSHIKRRVQ
1921  ELGHGCAALV TKAGALQCSP SDAYTKKELI ESARKVSEKV SHVLAALQAG NRGTQACITA ASAVSGIIAD LDTTIMFATA
2001  GTLNRENSET FADHREGILK TAKALVEDTK VLVQNATASQ EKLAQAAQSS VSTITRLAEV VKLGAASLGS EDPETQVVLI
2081  NAVKDVAKAL GDLIGATKAA AGKAGDDPAV YQLKNSAKVM VTNVTSLLKT VKAVEDEATK GTRALEATIE HIRQELAVFS
2161  SPVPPAQVST PEDFIRMTKG ITMATAKAVA AGNSCRQEDV IATANLSRRA IADMLRACKE AAYHPEVSAD VRQRALRFGK
2241  ECADGYLELL EHVLVILQKP THELKQQLAG YSKRVASSVT ELIQAAEAMK GTEWVDPEDP TVIAENELLG AAAAIEAAAK
2321  KLEQLKPRAK PKQADESLDF EEQILEAAKS IAAATSALVK AASAAQRELV AQGKVGVIPA NAVDDGQWSQ GLISAARMVA
2401  AATNNLCEAA NAAVQGHASE EKLISSAKQV AASTAQLLVA CKVKADHDSE AMKRLQAAGN AVKRASDNLV KAAQKAAAFQ
2481  DHDETVVVKE KMVGGIAQII AAQEEMLRKE RELEEARKKL AMIRQQQYKF LPTELRDEEQ N
```

5.133 prolyl-4-hydroxylase (AA 5 - 494) [Gallus gallus]

Protein Accession	gi|63739
Mean Expression Ratio	1.08
Median Expression Ratio	1.08
Credible Interval	(0.897, 1.30)
Associated Peptides	3
Associated Spectra	4
Coverage	0.0776

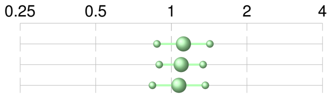

A	2.5	50	97.5	Sequence
1	0.88	1.1	1.4	VDATEEAELAQQFGVR
2	0.9	1.1	1.3	ILEFFGLK
1	0.84	1.1	1.4	YKPESDDLTADKIK

```
1     EEEDGVLVLR AANFEQALAA HRHLLVEFYA PWCGHCKALA PEYAKAAAQL KAEGSEIRLA KVDATEEAEL AQQFGVRGYP
81    TIKFFRNGDK AAPREYTAGR EADDIVSWLK KRTGPAATTL TDAAAAETLV DSSEVVVIGF FKDVTSDAAK EFLLAAESVD
161   DIPFGISSSA DVFSKYQLSQ DGVVLFKKFD EGRNNFEGDL TKDNLLNFIK SNQLPLVIEF TEQTAPKIFG GEIKTHILLF
241   LPKSVSDYEG KLDNFKTAAG NFKGKILFIF IDSDHSDNQR ILEFFGLKKE ECPAVRLITL EEEMTKYKPE SDDLTADKIK
321   EFCNKFLEGK IKPHLMSQDL PEDWDKQPVK VLVGKNFEEV AFDENKNVFV EFYAPWCGHC KQLAPIWDKL GETYRDHENI
401   VIAKMDSTAN EVEAVKIHSF PTLKFFPAGS GRNVIDYNGE RTLEGFKKFL ESGGQDGAAA DDDLEDLETD EETDLEEGDD
481   DEQKIQKDEL
```

5.134 SKI-like [Gallus gallus]

Protein Accession	gi|45642712
Mean Expression Ratio	1.08
Median Expression Ratio	1.08
Credible Interval	(0.835, 1.39)
Associated Peptides	1
Associated Spectra	1
Coverage	0.0159

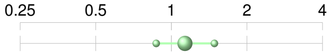

A	2.5	50	97.5	Sequence
1	0.87	1.1	1.5	QQEKLNTILRR

```
1     MESPQANFPL GLVSEQKRSG MQEDGSPPLK KLMTEMHVNS PVQVVINKLP TIKKENLDEY DETSVEADGE SAKPNSSSVS
81    EPLNLNPGLK HTLAQFHLSS QSSLGGPAAF SARYSQESMS PTVFLPLPSP QILSLPLLIP PDSSTELTQT VLEGESISCF
161   KVGGEKRLCL PQVLNSVLRD FSLQQINTVC DELYIYCSRC TSDQLHILKV LGILPFNAPS CGLITLTDAQ RLCNALLRPR
241   TFPQNGGFLP GKNTLAQLKE TGSAFEVEHE CLGKCQGLFA PQFYLAPDDP CIQCLECYGM FSPQTFVMHS HRSPDKRTCH
321   WGFESAKWHC YLHINQKYLG TSEERELKHL LEEMKEKFSE KNQKRTRSKV DSQQSLELSQ WYPVIKQEAE ADPQPPSFFH
401   PSYYLYMCDK VVAPNVSLAS QYKDVTKTTG KASEVIKSSS GTSEKKLSSG KHKKPASYPE LSLEEQEKID LKTGLEQHKR
481   LDPLVSAHSA RGGRSERISS KSIRGSRCVE VGNDGRTLSP TLMKDISCED DKGRIMEEVM KTYIKQQEKL NTILRRKQQL
561   QMEVEMLNNS KAMKELSEEQ QNLQKELESL QTEHAQRMEE FYFEQRDLEK KLDQMMKQKC TCDSSSEKDK EAEYAAQLAE
641   LRQRLDHAEA DRQELQDELR QEREAREKLE MMIKELKLQI LKSSKNGNGK
```

5.135 PREDICTED: hypothetical protein [Gallus gallus]

Protein Accession	gi|118089404
Mean Expression Ratio	0.93
Median Expression Ratio	0.929
Credible Interval	(0.722, 1.20)
Associated Peptides	1
Associated Spectra	1
Coverage	0.0422

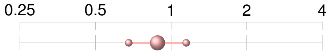

A	2.5	50	97.5	Sequence
1	0.68	0.88	1.1	EKPPEPSQEETSDKPK

```
1     MALLCYNKGC GQRFDPEHNS QDSCLYHPGD PIFHDALKGW SCCKKRTTDF SEFLSIKGCT KGFHSKEKPP EPSQEETSDK
81    PKAKPMAEII VQGPKSAEKM QRERPSSDEP RQLLPIKVSR SLEQALEKLS LSPNNKEPKG DCTGEAAAQV RAGTTCKNAT
161   CKAIYQGPES NTEVCTFHPG VPVFHEGMKY WSCCGIRTTD FSAFLEQPGC STGRHCWMGK ADKKAVSCRQ DWHQTSSQVV
241   VTIYGKNPLP TLSSVKANRT VLEVHVIFEG NKIFQAELDL WGVRSSEKSF VSMVPSKVEI TLRKANPGAW ARLEHPQSKA
321   CAQGEPEKAA VSTEEPEDDS DDSLSWSEED EEVEAADGAA PLRGFGESSA RADGALSSP
```

5.136 stathmin 1/oncoprotein 18 [Gallus gallus]

Protein Accession	gi|50053682
Mean Expression Ratio	0.928
Median Expression Ratio	0.93
Credible Interval	(0.746, 1.16)
Associated Peptides	2
Associated Spectra	2
Coverage	0.149

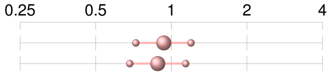

A	2.5	50	97.5	Sequence
1	0.72	0.93	1.2	ASGQAFELILGPR
1	0.68	0.88	1.1	KSHEAEVLK

```
1     MATSDIQVKE LEKRASGQAF ELILGPRSKE AAPEFPLSPP KKKDLSLEEI QKKLEAAEER RKSHEAEVLK QLAEKREHEK
81    EVLQKAIEEN NNFSKMAEEK LTHKMEANKE NREAQMAAKL ERLREKDKHI EEVRKNKEGK DPGEAETN
```

5.137 PREDICTED: hypothetical protein [Gallus gallus]

Protein Accession	gi|50751071
Mean Expression Ratio	1.08
Median Expression Ratio	1.08
Credible Interval	(0.832, 1.39)
Associated Peptides	1
Associated Spectra	1
Coverage	0.0957

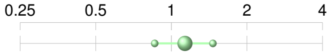

A	2.5	50	97.5	Sequence
1	0.86	1.1	1.5	AAEFHNHIFEK

```
1     MAAVRDVEID PDGTFKYILV RLQRPGGGEQ RDIVRGTKAA EFHNHIFEKV NPEMEKLGYE CKCLGGGKID HNSKEKKIRV
81    FGLSTGYGKA DHSVTVEILK KVYTDYEITW SDEKK
```

5.138 heat shock 60kDa protein 1 (chaperonin) [Gallus gallus]

Protein Accession	gi|61098372
Mean Expression Ratio	1.08
Median Expression Ratio	1.08
Credible Interval	(0.915, 1.26)
Associated Peptides	5
Associated Spectra	6
Coverage	0.136

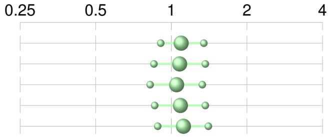

A	2.5	50	97.5	Sequence
2	0.9	1.1	1.3	LVQDVANNTNEEAGDGTTTATVLAR
1	0.85	1.1	1.4	AAVEEGIVPGGGCALLR
1	0.82	1.0	1.3	VGGTSDVEVNEK
1	0.86	1.1	1.4	NAGVEGSLIVEK
1	0.89	1.1	1.4	TVIIEQSWGSPK

```
1     MLRLPAVLRQ IRPVSRALAP HLTRAYAKDV KFGADARALM LQGVDLLADA VAVTMGPKGR TVIIEQSWGS PKVTKDGVTV
81    AKAIDLKDKY KNIGAKLVQD VANNTNEEAG DGTTTATVLA RAIAKEGFEK ISKGANPVEI RRGVMLAVDA ITAELKKLSK
161   PVTTPEEIAQ VATISANGDQ EIGNIISDAM KKVGRKGVIT VKDGKTLNDE LEIIEGMKFD RGYISPYFIN TAKGQKCEFQ
241   DAYVLISEKK ISSVQSIVPA LEIANSHRKP LVIIAEDVDG EALSTLVLNR LKVGLQVVAV KAPGFGDNRK NQLKDMAIAT
321   GGAVFGEEGL SLNVEDIQPH DFGKVGEVIV TKDDTMLLKG KGEKAQIEKR IQEIIEQLEV TTSEYEKEKL NERLAKLSDG
401   VAVLKVGGTS DVEVNEKKDR VTDALNATRA AVEEGIVPGG GCALLRCIPA LDALKPANED QKIGIEIIKR TLKIPAMTIA
481   KNAGVEGSLI VEKILQSSSE VGYDAMLGEF VNMVEKGIID PTKVVRTALM DAAGVASLLS TAEAVVTEVP KEEKEPAMGG
561   MGGMGGGMGG GMF
```

5.139 actinin, alpha 4 [Gallus gallus]

Protein Accession	gi|45384104
Mean Expression Ratio	1.08
Median Expression Ratio	1.07
Credible Interval	(0.884, 1.31)
Associated Peptides	3
Associated Spectra	3
Coverage	0.031

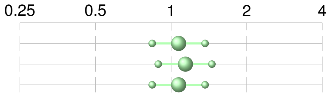

A	2.5	50	97.5	Sequence
1	0.84	1.1	1.4	LASDLLEWIR
1	0.9	1.1	1.5	VHKPPK
1	0.84	1.1	1.4	HRPELIEYDKLR

```
1     MVDYHSAGQP YPYGGNGPGP NGDYMAQEDD WDRDLLLDPA WEKQQRKTFT AWCNSHLRKA GTQIENIDED FRDGLKLMLL
81    LEVISGERLP KPERGKMRVH KINNVNKALD FIASKGVNVV SIGAEEIVDG NAKMTLGMIW TIILRFAIQD ISVEETSAKE
161   GLLLWCQRKT APYKNVNVQN FHISWKDGLA FNALIHRHRP ELIEYDKLRK DDPVTNLNNA FEVAEKYLDI PKMLDAEDIV
241   NTARPDEKAI MTYVSSFYHA FSGAQKAETA ANRICKVLAV NQENEHLMED YEKLASDLLE WIRRTIPWLE DRSPQKTIQE
321   MQQKLEDFRD YRRVHKPPKV QEKCQLEINF NTLQTKLRLS NRPAFMPSEG RMVSDINTGW QHLEQAEKGY EEWLLNEIRR
401   LEPLDHLAEK FRQKASIHEA WTEGKEAMLK QKDYETATLS DIKALIRKHE AFESDLAAHQ DRVEQIAAIA QELNELDYYD
481   SPSVNARCQK ICDQWDVLGS LTHSRREALE KTEKQLETID ELHLEYAKRA APFNNWMESA MEDLQDMFIV HTIEEIEGLI
561   AAHDQFKATL PDADREREAI LGIQREAQRI ADLHSIKLSG NNPYTSVTPQ VINSKWERVQ QLVPTRDRAL QDEQSRQQCN
641   ERLRRQFAGQ ANIVGPWMQT KMEEIGRISI EMHGTLEDQL QHLKHYEQSI VDYKPNLELL EHEHQLVEEA LIFDNKHTNY
721   TMEHIRVGWE QLLTTIARTI NEVENQILTR DAKGISQEQM QEFRASFNHF DKDHCGALGP EEFKACLISL GYDVENDRQG
801   DAEFNRIMSL VDPNGSGSVT FQAFIDFMSR ETTDTDTADQ VIASFKVLAG DKNYITAEEL RRELPPEQAE YCIARMAPYR
881   GPDAAPGALD YKSFSTALYG ESDL
```

5.140 PREDICTED: hypothetical protein [Gallus gallus]

Protein Accession	gi|118091140
Mean Expression Ratio	0.93
Median Expression Ratio	0.93
Credible Interval	(0.748, 1.16)
Associated Peptides	2
Associated Spectra	2
Coverage	0.0985

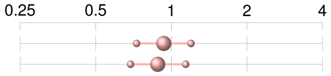

A	2.5	50	97.5	Sequence
1	0.73	0.93	1.2	TADLDGDSAATR
1	0.69	0.88	1.1	QATYGYYLENPEEFQDATDR

```
1     MGGGAGRCPA ALLLLALPLL ALPGGLGKPT AWQERARPGT AQQHEDAPSF QYDHEAFLGK EEARSFDQLS PEESQERLGK
81    IVDRIDENKD GYLTTEELKN WIKRVQKRYI YENVAKVWKD YDTNKDNKIT WEEYKQATYG YYLENPEEFQ DATDRHSFKK
161   MLPRDERRFK TADLDGDSAA TREEFTAFLH PEEFEHMKDI VVLETLEDID KNEDGFVDQD EYIADMFANE EGGPEPDWVT
241   TEREQFSDFR DLNKDGKMDK EEIQHWILPQ DYDHALAEAR HLVYESDVDK DQKLTKEEVL DNWNMFVGSQ ATNYGEDLTR
321   NHDEL
```

5.141 nuclear ubiquitous casein kinase and cyclin-dependent kinase substrate [Gallus gallus]

Protein Accession	gi|47087173
Mean Expression Ratio	1.08
Median Expression Ratio	1.07
Credible Interval	(0.863, 1.34)
Associated Peptides	2
Associated Spectra	2
Coverage	0.113

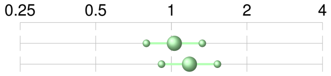

A	2.5	50	97.5	Sequence
1	0.8	1.0	1.3	VVDYSQFQESDDADEDYGR
1	0.91	1.2	1.5	RPAEDSEDEKEDHK

```
1     MSRPVRNRKV VDYSQFQESD DADEDYGRDS GPPSKKIRSS PREAKNKRRS GKNSQEDSED SEEKDVKTKK DDSHSADEDF
81    GSEDDDLGAD DGKADSDYES SQKSKKGKKA KPDKNKRASK SRKRPAEDSE DEKEDHKNVR QQRQAASKAA SKQREMLMDD
161   VGSEEEEQED DEAQFQENSG SDEDFLMEDD DDSDYGSSKK KNKKASKKSK PERKEKKMPK PRLKATVTPS PVKGKGKAGR
241   PTASKTTKEK TPSPKEEDEE PESPPEKKKS ASPPPEKSGD EGSEEEAPSG ED
```

5.142 hypothetical protein LOC418497 [Gallus gallus]

Protein Accession	gi|57525473
Mean Expression Ratio	1.07
Median Expression Ratio	1.07
Credible Interval	(0.861, 1.34)
Associated Peptides	2
Associated Spectra	2
Coverage	0.0586

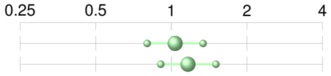

A	2.5	50	97.5	Sequence
1	0.8	1.0	1.3	ETKLLSDYVGR
1	0.9	1.2	1.5	QEELKK

```
1     MVRLHVKRGD ESQFLLEAAC GARLAELAPL VARIYNGRLK VQRLCAEMQE LAEHGAYLPL NMQGLTDEQI EELKLKDEWA
81    EKCVPSGGSV FKKDEIGRRN GHAPNEKMQQ VIRKTIEEAK ALISKKQVQA NVCVNLEMVK DALDQLRGAV MIVYPMGLPP
161   HDPVRMELED KEDLSGTHAG LEVIEEGEAQ LWWAGKELKE TKLLSDYVGR NEKTTIIVKI QKKGQGAPGR EPLISHEEQK
241   QMMLYYYKKQ EELKKLEEDD DDSFLNAEWA DSHALKRQFH GVKDIKWGPR
```

5.143 single stranded D box binding factor 2 - chicken

Protein Accession	gi|1362732
Mean Expression Ratio	0.932
Median Expression Ratio	0.932
Credible Interval	(0.747, 1.16)
Associated Peptides	2
Associated Spectra	2
Coverage	0.0425

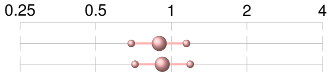

A	2.5	50	97.5	Sequence
**1**	**0.69**	**0.9**	**1.2**	**KDPVKK**
**1**	**0.72**	**0.92**	**1.2**	**KFHNVSGSK**

```
1      MSEAEQQLAA AAGATQNGHE AAESSGEQQQ ADTGGAPAAS AGAATAAAAT AGTAAAAGAG PAAGTAGVAA SQNGAEGDQI
81     NASKNEEDAG KMFVGGLSWD TSKKDLKDYF TKFGEVTDCT IKMDPNTGRS RGFGFILFKE PGSVEKVLEQ KEHRLDGRLI
161    DPKKAMAMKK DPVKKIFVGG LNPEATEEKI REYFGEFGEI EAIELPMDPK TNKRRGFVFI TFKEEDPVKK VLEKKFHNVS
241    GSKCEIKVAQ PKEVYQQQQF SSGGGRGSYG GRGRGGRGGA QSQNWNQGYG NYWNQGYGNQ GYGYQQGYGG YGGYDYSGYG
321    YYGYGPGYDY SQGSANYGKT PRRGGHQNNY KPY
```

5.144 pyruvate dehydrogenase (lipoamide) alpha 1 [Gallus gallus]

Protein Accession	gi|60302740
Mean Expression Ratio	1.07
Median Expression Ratio	1.07
Credible Interval	(0.827, 1.38)
Associated Peptides	1
Associated Spectra	1
Coverage	0.0326

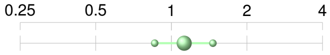

A	2.5	50	97.5	Sequence
**1**	**0.86**	**1.1**	**1.5**	**LEEGPATTAVLTR**

```
1      MRKMLLAALS RVLQGPVAAA GRTASRVMVA SRNYADFANE ATFEIKPCDL HRLEEGPATT AVLTREEGLH YYKTMQTIRR
81     MELKSDQLYK QKIIRGFCHL YDGQEACCVG LEVAIKPTDH VITAYRAHGF TYARGVPVRE ILAELTGRKG GCAKGKGGSM
161    HMYTKNFYGG NGIVGAQVPL GAGIALACKY FGKNEVCLTL YGDGAANQGQ IFETYNMAAL WKLPCIFICE NNRYGMGTSV
241    ERAAASTDYY KRGDFIPGLR VDGMDVLCVR EAAKFAAEYC RAGKGPIVME LQTYRYHGHS MSDPGISYRT REEIQEVRSK
321    SDPITLLKDR MINNNLASVE ELKEIDVAVR KEIEEAAQFA TTDPEPPLEE LGNHIYFNEP PFEVRGPNQW IKYKSLSHS
```

5.145 electron transfer flavoprotein, alpha polypeptide [Gallus gallus]

Protein Accession	gi|71895681
Mean Expression Ratio	1.07
Median Expression Ratio	1.07
Credible Interval	(0.883, 1.30)
Associated Peptides	2
Associated Spectra	5
Coverage	0.104

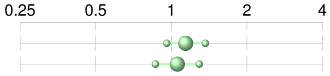

A	2.5	50	97.5	Sequence
**3**	**0.96**	**1.1**	**1.4**	**LLYDLADQLNAAVGASR**
**2**	**0.86**	**1.1**	**1.3**	**AAVDAGFVPNDMQVGQTGK**

```
1      MLRAAAARGV RRAASLLHRF QSTLVIAEHN NETLTPITLN AVTAAKRLGG EVSCLVAGTS CDKVAQELSK VQGVAKVLVA
81     QHDVYKGFLA EELTPLVLET QKQFKYTHIC AGVSAFGKNL IPRVAAKLDV APVSDIIEIK SPNTFVRTIY AGNVLCTVQC
161    DEAVKVFSVR GTSFEAAPTS GGSASVEKVT PPPPVGLSEW IEQKLSKSDR PELTSARVVV SGGRGLKSGE NFKLLYDLAD
241    QLNAAVGASR AAVDAGFVPN DMQVGQTGKI VAPELYIAVG ISGAIQHLAG MKDSKTIVAI NKDPEAPIFQ VADYGLVADL
321    FQVRLSWGTG ERKLSNVMTL QIGNRI
```

5.146 glutaredoxin 5 homolog [Gallus gallus]

Protein Accession	gi|56605958
Mean Expression Ratio	1.07
Median Expression Ratio	1.07
Credible Interval	(0.827, 1.38)
Associated Peptides	1
Associated Spectra	1
Coverage	0.117

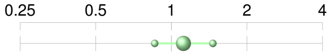

A	2.5	50	97.5	Sequence
**1**	**0.86**	**1.1**	**1.5**	**GSPAQPLCGFSNAVVQILR**

```
1       MSGSLRAALR LGVWAARGRP GRRHLSEAAS GSAGGSGGAD GGGSGSREAV ERLVREHPVV VFMKGSPAQP LCGFSNAVVQ
81      ILRLHGVEDY RAHDVLQDPD LRQGIKNYSN WPTIPQVYLN GEFVGGCDIL LQMHQNGDLV EELKKLGIRS ALLDAEKDQE
161     KK
```

5.147 PREDICTED: similar to MGC97820 protein [Gallus gallus]

Protein Accession	gi|50756041
Mean Expression Ratio	1.07
Median Expression Ratio	1.07
Credible Interval	(0.826, 1.39)
Associated Peptides	1
Associated Spectra	1
Coverage	0.0305

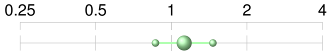

A	2.5	50	97.5	Sequence
**1**	**0.86**	**1.1**	**1.5**	**LIGQIVSSVTASLR**

```
1       MRECISIHIG QAGVQMGNAC WELYCLEHGI QADGTIPGSK LVKPVEPQSE QVDSSFETFF CETASGKHVP RAVFIDLEPT
81      VIDEIRTGTY HGLFHPEQLI SGKEDAANNY ARGHYTIGKE IIDTVLSRIR KMADQCSGLQ GFLVFHSFGG GTGSGFTSLL
161     MERLSVEYSK KSKLEFSVYP APQVSTAVVE PYNSILTTHT TLEHSDCSFM VDNEAIYDIC NRNLDIERPT YTNLNRLIGQ
241     IVSSVTASLR FNGALNVDLI EFQTNLVPYP RIHFPLTTYA PIISAEKAYH EQLSVPEITN ACFEFSNQMV KCDPRRGKYM
321     ACCLLYRGDV VPKDVNAAIA AIKTRRSIQF VDWCPTGFKV GINYQPPTVV PGGDLAKVQR AVCMLSNTTA IAEAWARLDH
401     KFDLMYAKRA FVHWYVGEGM EEGEFSEARE DLAALEKDYE EVGRDSADGE EDEVDEDEY
```

5.148 protein phosphatase 1, regulatory (inhibitor) subunit 2 [Gallus gallus]

Protein Accession	gi|71896917
Mean Expression Ratio	1.07
Median Expression Ratio	1.07
Credible Interval	(0.83, 1.38)
Associated Peptides	1
Associated Spectra	1
Coverage	0.0675

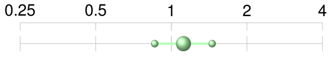

A	2.5	50	97.5	Sequence
**1**	**0.86**	**1.1**	**1.5**	**KGGGKQPAGGAGSGAR**

```
1       MEAPSVPAVS TAGTAGRAPI KGILKKGGGK QPAGGAGSGA RQASPARDDD EHGKKSQKWD EMNIIATYHP AGKDYGLMKI
81      DEPSTPYHSM VGDDDEDAVS DSESNEPLRA DVLSKKLAAA AEGKGPKVIA RQEESSEEEE EEEELTPEER EKKKQFEMKR
161     KMHYNEGRNI KLARQLIAKE LHGEDEEEEE EDEEMRDAPD VETMNTEDIE HGERSTPVGN IMTFLHGLKL PSVCTVL
```

5.149 PREDICTED: similar to acid alpha glucosidase [Gallus gallus]

Protein Accession	gi|118097771
Mean Expression Ratio	0.934
Median Expression Ratio	0.935
Credible Interval	(0.722, 1.21)
Associated Peptides	1
Associated Spectra	1
Coverage	0.0140

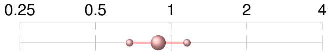

A	2.5	50	97.5	Sequence
1	0.68	0.89	1.2	EAEATASALIHIR

```
1      MRSYQKLRTA VPQLVLSQEE EEESAATPTP AGRSKLAPWW VGSGLLITAV LLSTITVWVL RQVSRGWHGP APPSQCHLVP
81     ESHRYDCYPE RSVVVTQELC ESRGCCFIET PPAVGGKRGV PWCFYPPSFP SYVVQSLNQT ALGMTGLLVR REKAYYPKDI
161    QVLRMDVEFQ TNTRLRIKIT DAAKPRYEVP LEVPRVMKRA ENPIYSLEFS QDPFGVLLRR QGTGTVLLNT TVAPLIFADQ
241    FLQISTTLPS RFLYGLGEHR STLLHSLDWN TLTLWARDVA PTESFNLYGA HPFYLLMEEG GDAHGVFLLN SNAMEVALQP
321    APGLTWRTIG GVLDFYIFLG PDPNMVIQQY QEVIGFPAMP PLWALGFHLC RWGYGSSNET WQTARAMRNF QIPQDAQWND
401    IDYMDGYRDF TFDPQKFASL PSLVEDLHKH GQHYVMILDP GISSTSPHGS YWPFDEGLRR ALFLNTTQGQ PLIGQVWPGY
481    TAFPDFSNRD THQWWLENLQ RFHTRVPFDG LWIDMNEPSN FMDGSEEGCP PGELDSPPYT PAVLGNSLSA KTVCASAKQN
561    ASVHYNLHNL YGLKEAEATA SALIHIRGKR PFVISRSTFP SQGRYSGHWL GDNRSQWKDM YYSIPGMLSF SLFGIPLVGA
641    DICGFSGSTS EELCTRWMQL GAFYPFSRNH NTQNEKAQDP TAFSPSARTA MKDVLLTRYS LLPFLYTLFH RAHLQGETVA
721    RPLFFEFPWD VATYGLDRQF LWGQSLLVTP VLEPGADSVL GYFPRGVWYD FYTGSSVNSS GEMLKLSAPL DHLNLHLREG
801    SILPTQKPGT TSKATRGNPL RLIVALSPRA TAWGDLFWDD GESLDTFERG NYSYLVFNAT ENIFTSNVLH ASTEATYVTI
881    DTVSFYGMQE PPSKVLLDGQ EKPFSYLDNQ VLTVSGLGLR LSQGFSLQWL
```

5.150 slow skeletal ventricular myosin alkali light chain 3 [Gallus gallus]

Protein Accession	gi|45384044
Mean Expression Ratio	1.07
Median Expression Ratio	1.07
Credible Interval	(0.91, 1.25)
Associated Peptides	5
Associated Spectra	9
Coverage	0.273

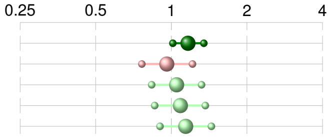

A	2.5	50	97.5	Sequence
5	1.0	1.2	1.3	DTGTYEDFVEGLR
1	0.76	0.96	1.2	ITYAQCGDVLR
1	0.83	1.0	1.3	KEVEFNPASIK
1	0.86	1.1	1.4	TKDTGTYEDFVEGLR
1	0.9	1.1	1.4	KEEPKPAPKPAEPEPK

```
1      MPPKKPEPKK APEPKKEEPK PAPKPAEPEP KKEVEFNPAS IKVEFTPDQI EEFKEAFSLF DRTPKSEMKI TYAQCGDVLR
81     ALGQNPTQAE VMKVLGRPKQ EEMNSKMIDF ETFLPMLQHI SKTKDTGTYE DFVEGLRVFD KEGNGTVMGA ELRHVLRTLG
161    ERLTEEEVDK LMAGQEDANG CINYEAFVKH IMAN
```

5.151 hypothetical protein [Gallus gallus]

Protein Accession	gi|60098993
Mean Expression Ratio	0.935
Median Expression Ratio	0.936
Credible Interval	(0.722, 1.21)
Associated Peptides	1
Associated Spectra	1
Coverage	0.0141

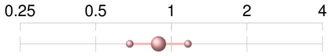

A	2.5	50	97.5	Sequence
1	0.68	0.9	1.2	NIEMVDGEQGR

```
1      MEKQQIKDKK LPVGFTFSFP CRQSKLDEGI LITWTKRFKA SGVEGADVVK LLNKAIKKRG DYDADIMAVV NDTVGTMMTC
81     GFDDQRCEVG LIIGTGTNAC YMEEMRHIDL VEGDEGRMCI NTEWGAFGDD GSLEDIRTEF DREIDRGSLN PGKQLFEKMV
161    SGMYMGELVR LILVKMAKEG LLFEGRITPE LLTKGKFETK HVSAIEKSKE GLNKAKEILT RLGVEPSHED CIAVQHVCTI
241    VSFRSANLVA STLGAILNQL RDNKGVGRLR TTVGVDGSLY KMHPQYARRL HKTTRRLVPD SDVRFLLSES GSGKGAAMVT
321    AVAYRLSEQH RLIDETLAEF KLTHEQLLQV KKRMRTEMEA GLKKKSHETA KVKMLPTFVR STPDGTENGD FLALDLGGTN
401    FRVLLVKIRS GKRRTVEMHN KIYAIPIEVM QGTGEELFDH IVTCISDFLD YMGIKGARLP LGFTFSFPCK QTSLDAGILL
481    NWTKGFKATD CEGEDVVYLL REGIKRREEF DLDVVAVVND TVGTMMTCAY EDPNCEIGLI VGTGSNACYM EEMRNIEMVD
561    GEQGRMCVNM EWGAFGDNGC LDDIRTIYDK AVDDYSLNAG KQRYEKMISG MYLGEIVRNI LIDFTKRGFL FRGQISETLK
641    TRHIFETKFL SQIESDRLAL LQVRTILQQL GLNSTCDDSI IVKTVCGAVS KRAAQLCGAG MVAVVDKIRE NRGLEHLEIT
721    VGVDGTLYKL HPHFSRIMHQ TVKDLAPKCD VTFLLSEDGS GKGAALITAV GCRLRDAEQN
```

5.152 PREDICTED: similar to NIPA-like domain containing 3 [Gallus gallus]

Protein Accession	gi|118101770
Mean Expression Ratio	1.07
Median Expression Ratio	1.07
Credible Interval	(0.824, 1.38)
Associated Peptides	1
Associated Spectra	1
Coverage	0.0292

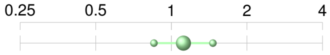

A	2.5	50	97.5	Sequence
**1**	**0.85**	**1.1**	**1.5**	**AVAGMIVVSIR**

```
1      MMENLIGALL AIFGHLVISI ALNLQKYSHI RLAGSKDSRA YFKTKTWWCG LFLLVLGELG VFSSYAFAPL SLIVPLSAVS
81     VIASAIIGII FIKEKWKPKE FLRRYVLSFV GCGLAIVGTY LLITFGPNSH EKMTGENITR HLVSWPFLLY MLVEIIIFCL
161    LLYFYKEKNA NYIVIILLLV ALLGSMTVVT VKAVAGMIVV SIRGNLQLNY PIFYIMLVCM IATAVFQATF LAQASQLYDS
241    SQIASIGYIL STTVAITAGA TFYLDFTGED VLHICMFALG CLIAFLGVFL ITRNRKKSVP FEPYISMDAM PGMQNMHDKG
321    IAVQPDLKAS FSYGALENND NMPEIYTPAT LPIVQEQHGS RGVSAPPYRV LEHSKKE
```

5.153 adenylosuccinate lyase [Gallus gallus]

Protein Accession	gi|51874220
Mean Expression Ratio	0.938
Median Expression Ratio	0.937
Credible Interval	(0.724, 1.22)
Associated Peptides	1
Associated Spectra	1
Coverage	0.0227

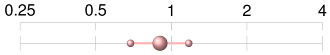

A	2.5	50	97.5	Sequence
**1**	**0.69**	**0.9**	**1.2**	**QEGGDNDFIAR**

```
1      MATPCAEEDP LARYRSPLVS RYASAEMGFN FSERKKFGTW RRLWLYLAQA EKSLGLPITD EQIKEMEANL DNIDFKMAAE
81     EEKKLRHDVM AHVHTFAHCC PKAAAIIHLG ATSCYVGDNT DLIVLRDGFN LLLPKLARVI SRLADFAETH ADLPTLGFTH
161    YQPAQLTTVG KRCCLWIQDL CMDLQNLERA RDDLRLRGVK GTTGTQASFL QLFEGDHSKV EELDRLVTAK AGFKRSYMVT
241    GQTYSRKVDI EVLSVLASLG ASVHKICTDI RLLANLKEIE EPFEKDQIGS SAMPYKRNPM RSERCCSLAR HLMTLVLDPL
321    QTASVQWFER TLDDSANRRV CLAEAFLTAD IILSTLQNIS EGLVVYPKVI DRRIRQELPF MATENIIMAM VKAGGNQQDC
401    HEKIRVLSQQ AAAVVKQEGG DNDFIARVRA DPYFSPIHEH LDSLLDPSSF TGRAPQQVAK FLKEEARPAL IPCQSMMGGK
481    IELTL
```

5.154 acyl-Coenzyme A dehydrogenase, C-2 to C-3 short chain [Gallus gallus]

Protein Accession	gi|57525201
Mean Expression Ratio	1.07
Median Expression Ratio	1.07
Credible Interval	(0.824, 1.38)
Associated Peptides	1
Associated Spectra	1
Coverage	0.0312

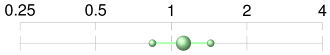

A	2.5	50	97.5	Sequence
**1**	**0.84**	**1.1**	**1.4**	**ITEIYEGTSEIQR**

```
1      MAAAAALALL VTRGGGVPRA RALGSLRRLH TVYQTVELPE THQMLRQTCR DFAEKELMPL AAELDREHRF PAEQVKKMGS
81     LGLLAVEVPE QFKGAGLDYL AYSIAVEEIS RGCASTGVIA SVNNSLYLGP ILKFGSEEQK HKWISPFTSG DKIGCFALSE
161    PGNGSDAGAA STVARLDGDE WVLNGTKAWI TNAWDASATV VFATTDKSLK HKGISAFLVP MPTAGLSLGK KEDKLGIRAS
241    STANLIFEDC RIPKANLLGQ LGMGFKIAMQ TLDGGRIGIA SQALGIAQAA LDCAVDYAEK RMAFGSPITK LQAIQFKLAD
321    MAVALESARL LTWRAAMLKD NGKPFTKEAA MAKLAASEAA TNIAHQAIQI LGGMGYVTEM PAERHYRDAR ITEIYEGTSE
401    IQRLVIAGQL LKAYRS
```

5.155 PREDICTED: similar to Unc-45 homolog B (C. elegans) [Gallus gallus]

Protein Accession	gi|118100114
Mean Expression Ratio	0.937
Median Expression Ratio	0.937
Credible Interval	(0.753, 1.17)
Associated Peptides	2
Associated Spectra	2
Coverage	0.0239

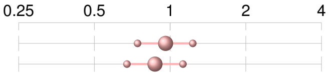

A	2.5	50	97.5	Sequence
1	0.74	0.96	1.2	IAAISNPDIAFPGER
1	0.67	0.87	1.1	QHVPEEHPK

```
1      MAGNTRDGDF LGVAFGQTYR GVQMEDPIQL KEEGNKYFQA SDYERAAQSY TQAMKLNKDR ALQAVLYRNR AACFLKREEY
81     AKAASDASRA IDINSSDIKA LYRRSQALEK LGKLDQAFKD AQKCATMEPH NKNFQETLRR LGADIQEKAA NNLIVLGREE
161    AGAERIFQNN GVSLLLQLIE TKNAELVLAA VRTLSGMCTG HKARATAILH YLGIDNICMW MSVDNEEISL AVCNLLQTIT
241    DCLLGQGKEE HHGKEEAVVL DTKKDLKMIT MRLLDMLVSK NVSGQGRDRA LNLLNKNIPR KDLKDQDNSR TTFVIDNGLK
321    KILKVVGQIP EMPGCLPLTE NTQLTASILL NKLYDDLRCD PERDNYRVIC EEYIKSKIDP QNMDKTLHAI QIVSGVLQGP
401    FDLGNKLLGM KGVMEMMVAL CGSEREIDQL VAVEALIHAS TKLSRATFII SNGITLLKEI YKKTKNEKIK IRALVGLCKL
481    GSAGGTDYGL RQFAEGSTEK LARQCRKWLC NTSIDARTRK WAVEGLAYLT LDADVKDDFV EDEQALQAMF ELAKTSDKTI
561    LYSVASALVN CTNSYDTKEL VPELVQLAKF SKQHVPEEHP KDKKDFVVKR VKRLLKAGVV SALACMVKAD SAILTDQSKE
641    LIARVFLALC DDPKDRGTIV AQGGGKALIP LAVEGTDVGK IKASHALAKI AAISNPDIAF PGERVYEVVR PLVSLLNTER
721    DGLQNYEALL GLTNFSGRSD KLRMKIVKEG ALPDIENYMF ENHDQLRQAA TECMCNLVVS KEVQERFVAD GNDRLKLVVL
801    LCGEDDEKVQ KAAAGALAML TAAQKKLCSK MTEVTTQWLE ILQRLCLHDN MEVQHRGLVI AFNLISASKE LAKKLVESEL
881    LEILTYVGKQ EDDPKKQHII NAARDCLTKC MDYGLIKPLS RACYFTVFIQ GNTTAHRFHF LRKSCYATAG PPHRAAPPPQ
961    PRRAGNSRAG ASPSYWRAPE SVLVIALYSF LSACGEGSFY GALF
```

5.156 PREDICTED: similar to endo-beta-N-acetylglucosaminidase [Gallus gallus]

Protein Accession	gi|118099855
Mean Expression Ratio	0.938
Median Expression Ratio	0.939
Credible Interval	(0.723, 1.22)
Associated Peptides	1
Associated Spectra	1
Coverage	0.0171

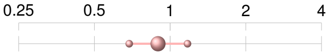

A	2.5	50	97.5	Sequence
1	0.69	0.9	1.2	LLLVYSEPAGGSS

```
1      MAEAAAQDGT ARRGKRAAEE GEEERGGRRR RRLGRGWGLG AGRGARSGRS GRSGRSEARC GGRWPRCCPA RRGGRTRSGA
81     RGAGRPRASP LSPGSFQPAA EPLGTTVLHA AVDTRPQPLP ARYFDTGTTE PVSFFLSGLE ELLAWHPSSD DEFNVCAVPL
161    AQRQPPLHSR RPRTLLCHDM RGGYLEDRFI QGSATRNPYV FYHWRYVDIF VYFSHHTVTI PPVCWTNAAH RNGVPVLGTF
241    ITEWADGEKL CEAFLAGGED AYRAVSHQLA RIAQHYRFDG WLINIENALS AAAVGNLSPF LRHLTAEVHG AVPGGLVIWY
321    DSILESGTLR WQNELNQQNR VFFDACDGLF VNYNWKEEHL ERTRELAGQR HADVYIGVDV FARGDVVGGG FDTNKSLSLI
401    RKHGLSAAIF APGWVYKHLG EENFLLNEDK FWGLLEDYLP THSICTLPLA TSFSVGMGTG MFLAGKEEEA GPWYNLSAQE
481    IQPLYPERRG WLSTSCCLQD AWCGGSSLRV QGTIPPGEER VAIRGPHRDD FTVALELTTW HSSRCHDGTV TVLPSEDEPH
561    GRHHPHLLPA PPPALSRMLA ACSHGAQGWT SRCYEQELRG CSLRDLSLLV SRQQASPQET SFSCLLGELR VLDAGSMAAS
641    PPQVQSLTAS QLWWQDGPSA EQLSLSLTLR WAFPPGRAAC FRVLSQGARC HRAQPAQPQL LGLAHGCQYR AVGLAVPRPA
721    PGQSCQLELL VEPVLPSELP VGPERWGRLL LVYSEPAGGS S
```

5.157 PREDICTED: similar to rab effector MYRIP [Gallus gallus]

Protein Accession	gi|118086154
Mean Expression Ratio	0.94
Median Expression Ratio	0.939
Credible Interval	(0.727, 1.22)
Associated Peptides	1
Associated Spectra	1
Coverage	0.00914

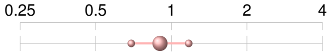

A	2.5	50	97.5	Sequence
**1**	**0.69**	**0.9**	**1.2**	**FNEHCCIR**

```
1       MGRKLDLSGL TDEEAEHVLQ VVQRDFSLRK KEEERLSEMK QKLDEEGNKC SILSKQQKFN EHCCIRCCSP FTFLINSKRQ
81      CQDCKYNICK SCSSYQKKEK AWICSVCQQA RLLRTQSLDW YYNNVKSRFK CFGSAKVLKN LYKKHRLESG VCPDVIEGGC
161     FEGSFENDGS ICGSDSTFYR ETEGHSMMDT LAVALRVAEE AVEEAISKAE TYSDSLDKQN EACYLQEHKE DLIEELATTI
241     VQKIIKKQKG KTEQAEADFE WPPSRSSGLA SVAVSDQSML TFPGSRRGSY TLWRSQSAFS LASEDAPSKG LDPASSVTEA
321     LWRQQKGQFK KQKERKLSAL PSWKSVDRLN ETNPPPVLQS TDGNWVALQN ISLPRPRMLA KPKSQVFEAL ENESSVVSAY
401     DEMGSDSEDD YDWNVALNKL RRRPRQLPDD FYCTNSQYDS QWVYGNDQYQ AVTSPSSGLY TNTETLFSDS ETSSVNSSQE
481     AKGPSKLLWL QSRTQSDVPR MEKKHFHGEL DVNFNPQATS LEYSDSSETE EVQYDLEKRS RRWRKNKTIS EESCEGKNHT
561     KANVKNLSQV IDLDDLSETD GSSEDQHHNI KPELMEEELK SRLFQLAAKM SDKETSSGEE QESEPRTDPE NQKESLSSEE
641     NGKSIQEELK KKYSAVSLCN ISTEVLKVIN ATEELIAESG SPCDFPDDVQ DKGRGTFPLG TDPIRLDEQL TTLEENVYLT
721     ASTVYGLEGQ LTTLEDAARQ ISSVTAESEL AELEDQVATA AAQVHHAELQ ISDIESRISA LTVAGLNVAP CVRLTRKRDQ
801     KQTNQMHTID TSRQQRRKLP APPVKGEKMD GSPITTVRTF NRNFMLQGSL TQRTKERKST AKDLMEPAIG SAVMY
```

5.158 tyrosine 3-monooxygenase/tryptophan 5-monooxygenase activation protein, zeta polypeptide [Gallus ga

Protein Accession	gi|71897035
Mean Expression Ratio	1.07
Median Expression Ratio	1.07
Credible Interval	(0.873, 1.30)
Associated Peptides	3
Associated Spectra	3
Coverage	0.155

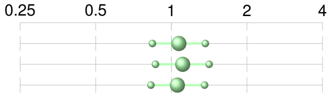

A	2.5	50	97.5	Sequence
**1**	**0.84**	**1.1**	**1.4**	**SVTEQGAELSNEER**
**1**	**0.87**	**1.1**	**1.4**	**DICNDVLSLLEK**
**1**	**0.83**	**1.1**	**1.4**	**YLAEVAAGDDKK**

```
1       MDKNELVQKA KLAEQAERYD DMASCMKSVT EQGAELSNEE RNLLSVAYKN VVGARRSSWR VVSSIEQKTE GAEKKQQMAR
81      EYREKIETEL RDICNDVLSL LEKFLIPNAS QAESKVFYLK MKGDYYRYLA EVAAGDDKKG IVEQSQQAYQ EAFEISKKEM
161     QPTHPIRLGL ALNFSVFYYE ILNSPEKACS LAKTAFDEAI AELDTLSEES YKDSTLIMQL LRDNLTLWTS DTQGDEAEAG
241     EGGEN
```

5.159 RAN, member RAS oncogene family [Gallus gallus]

Protein Accession	gi|45383966
Mean Expression Ratio	0.937
Median Expression Ratio	0.94
Credible Interval	(0.75, 1.17)
Associated Peptides	2
Associated Spectra	2
Coverage	0.088

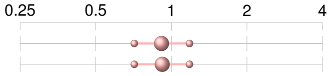

A	2.5	50	97.5	Sequence
**1**	**0.71**	**0.91**	**1.2**	**YVATLGVEVHPLVFHTNR**
**1**	**0.71**	**0.92**	**1.2**	**KYVATLGVEVHPLVFHTNR**

```
1       MAAQGEPQVQ FKLVLVGDGG TGKTTFVKRH LTGEFEKKYV ATLGVEVHPL VFHTNRGPIK FNVWDTAGQE KFGGLRDGYY
81      IQAQCAIIMF DVTSRVTYKN VPNWHRDLVR VCENIPIVLC GNKVDIKDRK VKAKSIVFHR KKNLQYYDIS AKSNYNFEKP
161     FLWLARKLIG DPNLEFVAMP ALAPPEVVMD PALAAQYEQD LQIAQTTALP DEDDDL
```

5.160 huntingtin interacting protein 2 [Gallus gallus]

Protein Accession	gi|71895881
Mean Expression Ratio	0.939
Median Expression Ratio	0.94
Credible Interval	(0.73, 1.22)
Associated Peptides	1
Associated Spectra	1
Coverage	0.06

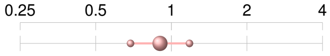

A	2.5	50	97.5	Sequence
**1**	**0.69**	**0.9**	**1.2**	**VDLVDENFTELR**

```
1       MANIAVQRIK REFKEVLKSE ETSKNQIKVD LVDENFTELR GEIAGPPDTP YEGGRYQLEI KIPETYPFNP PKVRFITKIW
81      HPNISSVTGA ICLDILKDQW AAAMTLRTVL LSLQALLAAA EPDDPQDAVV ANQYKQNPEM FKQTARLWAH VYAGAPVSSP
161     EYTRKIENLC AMGFDRNAVI VALSSKSWDV ETATELLLSN
```

5.161 PREDICTED: hypothetical protein [Gallus gallus]

Protein Accession	gi|50751398
Mean Expression Ratio	1.06
Median Expression Ratio	1.06
Credible Interval	(0.825, 1.38)
Associated Peptides	1
Associated Spectra	1
Coverage	0.0427

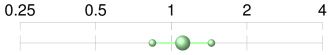

A	2.5	50	97.5	Sequence
**1**	**0.84**	**1.1**	**1.4**	**GPGDTSNFDDYEEEDIR**

```
1       MAAQKEVPCA QCTGTATALQ RLEGFANRLF HRHSKGGAHD LRVPLENESH HVSELAVLWD KSMKEFLAKA KEDFLRKWES
81      PPQNTAGLDD FERQKTLGTG SFGRVMMVKH KATEQYYAMK ILDKQKVVKL KQIEHTLNEK RILQAVNFPF LVKLEYSFKD
161     NSNLYMVMEY VPGGEMFSHL RRIGRFSEPH ARFYAAQIVL TFEYLHSLDL IYRDLKPENL LIDQQGYIQV TDFGFAKRVK
241     GRTWTLCGTP EYLAPEIILS KGYNKAVDWW ALGVLIYEMA AGYPPFFADQ PIQIYEKIVS GKVRFPSHFS SDLKDLLRNL
321     LQVDLTKRYG NLKNGVNDIK NHKWFATTDW IAIYQRKVEA PFIPKCRGPG DTSNFDDYEE EDIRASLTEK CAKEFADF
```

5.162 myosin IG [Gallus gallus]

Protein Accession	gi|71896596
Mean Expression Ratio	0.94
Median Expression Ratio	0.94
Credible Interval	(0.726, 1.21)
Associated Peptides	1
Associated Spectra	1
Coverage	0.00993

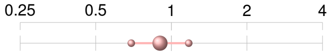

A	2.5	50	97.5	Sequence
**1**	**0.69**	**0.9**	**1.2**	**VKAYLLELIR**

```
1       MTELEGPEFG KADFVLLDEV TMEHFMENLR LRFSKGRIYT YIGEVVVAMN PYQPLELYGP SVVEQYRGRE LYERPPHLFA
81      LADAAYKAMK RRAKDTCIVI SGESGAGKTE ASKYIMQYIA AITNPTQRAE VERVKNGLLK SNCVLEAFGN AKTNRNDNSS
161     RFGKYMDINF DFKGDPTGGH IYNYLLEKSR VLQQQPGERN FHSFYQLLLG APDALLASLH LQRDPTAYCY TQQGTQGSAG
241     GDDARGYRAV EEAMAVIGFT PEEVGAVQRI LAAILHLGNV QFVAEGEVAA LEAVEQLAVL AQLTGTTPEQ LRQALLARTV
321     ATGGGELIEK GHSPTEAAYG RDACAKAIYE RLFGWIVGRI NASITARGYD VRQHGKSTVI GVLDIYGFEI FDTNSFEQFC
401     INYCNEKLQQ LFIELILRQE QAEYQREGIT WQNIEYFSNE PIVELVEQPH RGILALLDEA CLAVGTVTDA LFLANMDARL
481     GHHPHYSSRK LCPTDKTMEF DRDFRIKHYA GDVTYSVEGF LDKNKDTLFQ DFKRLLYNSM DPVLRAMWPD GEQSITEVTK
561     RPLTAATLFK NSIVALVENL ASKEPYYVRC IKPNDQKSPV LFDEERCRHQ VAYLGLLENV RVLRAGFAYR QPYDRFLQRY
641     KMTCEYTWPN HLMATDREAT QTLLEQHGFQ DDVAYGHTKV FIRTPRTLFC LEQERAQLIP IIVLLLQKAW RGALARRWCR
721     YLRAAYAIMG YYKRHKVKAY LLELIRRFQG VRSMPDFGKS LAWPEPPAVL SRFQENSQQL FRRWRARQIV KNIPPSDMAQ
801     IRAKVAAMGA LHGLRKDWGC QRGWVRDYLS SASENPGLAL PFAHRVQALR DKVHFGAVLF SSHVRKINRF NKSRDRAILI
881     TDQHLYKLEP RKQYRVMREL PLSMVTGLSV TSCRAQLVVF HTQNHDDLAV CLHKTQPRGD ERVGELVGVL LEHCRTTKRE
961     LQVHVSDRIQ LSLRGRKRLL TVETQPDVAA PDFRKSRDGF VLYWPGS
```

5.163 PREDICTED: similar to cell adhesion molecule JCAM [Gallus gallus]

Protein Accession	gi|118083908
Mean Expression Ratio	0.94
Median Expression Ratio	0.94
Credible Interval	(0.725, 1.22)
Associated Peptides	1
Associated Spectra	1
Coverage	0.0187

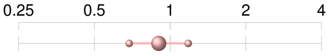

A	2.5	50	97.5	Sequence
1	0.69	0.9	1.2	CCCLKK

```
1       MERFQKFILL PFILAASLTG LGFCYSILNG PTNATVLAGS EARFNCTVAN GWAILIWLLN TNPVLTVINS HGPIETSDRF
81      TSQSYNNSQS FTSELIIHNT QLNDSGRIEC SIQKIDGSSF AFLSVQVNGT LLIKNSTLIV KENTTVEIVC EALGWAPAPE
161     ITWMTNDSLI DKSRYVTQQS QGSNSLYNAL SVLTLTPMDT EILTCLADIE ALPSPQNATI AVIVGNSTLE NNYSEDSTST
241     WVIVLAVVFS IVGFILLIIL IWVVVRCCCL KKGSTYENEV RKISVKKKTD DRLGSRQRSG SENQGYVPEE PPYTGQIPSE
```

5.164 PREDICTED: similar to spectrin, beta, non-erythrocytic 1 [Gallus gallus]

Protein Accession	gi|118087525
Mean Expression Ratio	0.94
Median Expression Ratio	0.94
Credible Interval	(0.814, 1.09)
Associated Peptides	7
Associated Spectra	8
Coverage	0.0398

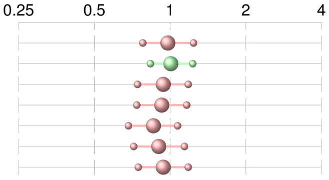

A	2.5	50	97.5	Sequence
1	0.78	0.98	1.2	WDVDDWDNENSSAR
2	0.84	1.0	1.2	DVEDEILWVGER
1	0.74	0.94	1.2	DLDDFQSWLSR
1	0.74	0.93	1.2	EIEELQSQAR
1	0.68	0.86	1.1	SQNIITDSSPNAEAIQQR
1	0.72	0.9	1.1	QLWNLLIEETEKR
1	0.75	0.94	1.2	LQAAYAGDKADDIQKR

```
1       MTTTVATDYD NIEIQQQYSD VNNRWDVDDW DNENSSARLF ERSRIKALAD EREAVQKKTF TKWVNSHLAR VSCRITDLYT
81      DLRDGRMLIK LLEVLSGERL PKPTKGRMRI HCLENVDKAL QFLKEQRVHL ENMGSHDIVD GNHRLTLGLI WTIILRFQIQ
161     DISVETEDNK EKKSAKDALL LWCQMKTAGY PNVNIHNFTT SWRDGMAFNA LIHKHRPDLI DFDKLKKSNA HYNLQNAFNL
241     AEQHLGLTKL LDPEDISVDH PDEKSIITYV VTYYHYFSKM KALAVEGKRI GKVLDNAIET EKMIEKYESL ASDLLEWIEQ
321     TIIILNNRKF ANSLVGVQQQ LQAFNTYRTV EKPPKFTEKG NLEVLLFTIQ SKMRANNQKV YMPREGKLIS DINKAWERLE
401     KAEHERELAL RNELIRQEKL EQLARRFDRK AAMRETWLSE NQRLVSQDNF GFDLPAVEAA TKKHEAIETD IAAYEERVQA
481     VVAVAKELET ENYHDIKRIT ARKDNVIRLW EYLLELLRAR RQRLEMNLGL QKIFQEMLYI MDWMDEMKVL LLSQDYGKHL
561     LGVEDLLQKH ALVEADIAIQ AERVRGVNAS AQKFATDGEG YKPCDPQVIR DRVAHMEFCY QELCQLSAER RARLEESRRL
641     WKFFWEMAEE EGWIREKEQI LSSDDYGKDL TSVVRLMSKH KAFEDEMSGR SGHFQQAIKE GEDMIAEENF GSEKIRERIK
721     DIREQWANLE QLSAIRKKRL EEASLLHQFQ ADADDIDAWM LDILKIVSSN DVGHDEYSTQ SLVKKHKDVA EEIASYRPTI
801     DSLHEQAKAL PQEHAGSPDV QGRLSGIEER YKEVAELTRL RKQALQDTLA LYKMFSEADA CELWIDEKEK WLNNMQIPEK
881     LEDLEVIQHR FESLEPEMNN QASRVAVVNQ IARQLMHSGH PSEKEIKAQQ DKLNTRWSQF RELVDRKKDA LISALSIQNY
961     HLECNETKSW IREKTKVIES TQDLGNDLAG VMALQRKLTG MERDLVAIEA KLSDLQKEAE KLESEHPDQA QAILSRLAEI
1041    NDVWEEMKTT LKNREESLGE ASKLQQFLRD LDDFQSWLSR TQTAIASEDM PNTLTEAEKL LTQHENIKNE INNYEEDYQK
1121    MRDMGEMVTQ GQTDAQYMFL RQRLQALDTG WNELHKMWEN RQNLLSQSHA YQLFLRDTKQ AEAFLNNQEY VLAHTEMPTT
1201    LEGAEAAIKK QEDFMTTMDA NEEKINAVVE TGRRLVSDGN INSDKIQEKV DSIDDRHRKN REAASELLMR LKDNRDLQKF
1281    LQDCQELSLW INEKMLTAQD MSYDEARNLH SKWLKHQAFM AELASNKEWL EKIEKEGMQL IAEKPETEAV VKEKLTGLHQ
1361    MWEELESTTQ TKAQRLFDAN KAELFTQSCA DLDKWLNGLE SQIQSDDYGK DLTSVNILLK KQQMLENQMD VRKKEIEELQ
1441    SQARALSQEG KSTDEVDGKR LTVEKKFLEL LEPLNERKAN LLASKEIHQF NRDVEDEILW VGERMPIATS TDHGHNLQTV
1521    QLLIKKNQTL QKEIQGHQPR IDDIFERSQN IITDSSPNAE AIQQRLADLK QLWNLLIEET EKRHKRLEES HRAQQYYFDA
1601    AEAEAWMSEQ ELYMMSEEKA KDEQSAVSML KKHQILEQAV EDYAETVHQL SKTSRTLVAD NHPESERISM RQSKVDKLYA
1681    GLKDLAEERR GKLDERHRLF QLNREVDDLE QWIAEREVVA GSHELGQDYE HVTMLQERFR EFARDTGNIG QERVDTVNHL
1761    ADELINSGHS DAATIAEWKD GLNEAWADLL ELIDTRTQIL AASYELHKFY HDAKEILGRI QDKHKKLPEE LGRDQNTVET
1841    LQRMHTTFEH DIQALGTQVR QLQEDAARLQ AAYAGDKADD IQKRENEVLE AWKALLDACE GRRVRLVDTG DKFRFFSMVR
1921    DLMLWMEDVI RQIEAQEKPR DVSSVELLMN NHQGIKAEID ARNDSFTTCI ELGKSLLARK HYASEEIKEK LLQLTEKRKE
2001    MIDKWEDRWE WLRLILEVHQ FSRDASVAEA WLLGQEPYLS SREIGQSVDE VEKLIKRHEA FEKSAATWDE RFAALERLTT
2081    LELLEVRRQQ EEEERKRKPP TPEPSPKVAE DGGSQQQWDG TKGEQVSQNG LPSDQESPRV AETAETNEMV NGAAEQRTSS
2161    KESSPVPSPT ADRKAKAAIQ AQTAATLPAK TQEIPSAQME GFLHRKHEWE THNKKASSRS WHNVYCVINN QEMGFYKDSK
2241    AAASGIPYHN EIPVSLKEAV CEIAVDYKKK KHVFKLRLTD GNEYLFQAKD DEEMNTWIQA ITSAISSDKI EVSPTTQSTP
2321    ASSRAQTLPA SVTITSESSP GKREKDKEKD KEKRFSLFGK KK
```

5.165 Ras-GTPase-activating protein SH3-domain-binding protein [Gallus gallus]

Protein Accession	gi|57525015
Mean Expression Ratio	0.941
Median Expression Ratio	0.94
Credible Interval	(0.73, 1.21)
Associated Peptides	1
Associated Spectra	1
Coverage	0.0212

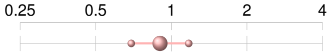

A	2.5	50	97.5	Sequence
1	0.69	0.9	1.2	EGEQGDVETR

```
1     MVMEKPSPLL VGREFVRQYY TLLNQAPDYL HRFYGKNSSY VHGGLDSNGK PADAVYGQSD IHKKVLSLNF KDCHTKIRHV
81    DAHATLNDGV VVQVMGELSN NMQPVRRFMQ TFVLAPEGSV ANKFYVHNDI FRYQDEVFGD SDTEPPEESE EEGEEPEERQ
161   QTPEAVPDDT GAYYEQTVSN DIEEHLEETT AEAEPEPEPE PEQEPEPEAQ EEKSEPVLEE SAPEETVEKS PSPAPADPAP
241   AVQEDSRTFS WASVTSKNLP PSGAVPVSGI PPHVVKVPVS QPRPEAKPES QTPPQRPQRD QRVREQRTSI PPQRGPRPIR
321   EGEQGDVETR RIVRYPDSHQ LFVGNLPHDV DKSELKDFFQ KLGSSLAGYG NVVELRINSG GKLPNFGFVV FDDPDPVQKI
401   LSNRPIMFRG EVRLNVEEKK TRAAREGDRR DNRPRGPGGT RGGLGGGIRG PPRGGMSQKP GFGAGRGIGQ RQ
```

5.166 myosin regulatory light chain 9 [Gallus gallus]

Protein Accession	gi|45384118
Mean Expression Ratio	0.94
Median Expression Ratio	0.94
Credible Interval	(0.732, 1.21)
Associated Peptides	1
Associated Spectra	1
Coverage	0.116

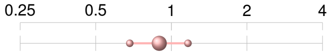

A	2.5	50	97.5	Sequence
1	0.68	0.9	1.2	NAFACFDEEASGFIHEDHLR

```
1     MSSKRAKAKT TKKRPQRATS NVFAMFDQSQ IQEFKEAFNM IDQNRDGFID KEDLHDMLAS MGKNPTDEYL EGMMSEAPGP
81    INFTMFLTMF GEKLNGTDPE DVIRNAFACF DEEASGFIHE DHLRELLTTM GDRFTDEEVD EMYREAPIDK KGNFNYVEFT
161   RILKHGAKDK DD
```

5.167 PREDICTED: similar to Vacuolar protein sorting 36 (yeast) [Gallus gallus]

Protein Accession	gi|50730915
Mean Expression Ratio	1.06
Median Expression Ratio	1.06
Credible Interval	(0.817, 1.38)
Associated Peptides	1
Associated Spectra	1
Coverage	0.0389

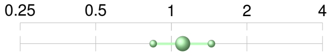

A	2.5	50	97.5	Sequence
1	0.85	1.1	1.4	ETDKNISEAFEDLSK

```
1     MDRFSWTSGL LELGETLVLQ QRGVRLYDGE EKVKFDSGVL LLSTHRLIWR DQKNHECCIA IPLSQVVFIE EQAAGIGKSA
81    KIVAHLHPAS PNKEPGPFQS SKYSYIKLSF KEHGQIEFFR RFSEEITQRR WENMPAGQTI QVNKDPQAGR IRAVGIVGIE
161   RKLEAKRKET DKNISEAFED LSKLMEKAKE MVELSKSIAN KIKEKQGDIT EDETIRFKSY LLSMGIANPV TRETHGSGTH
241   YHMQLAKQLA GMLQTPLEER GGIMSLTEVY CLVNRARGLE LLSPEDLVNA CKMLEPLKLP LRLQIFDSGV MVIELQSHNE
321   EEMVASALET VSEKGSLTAD EFAKLVGMSV LLAKERLLLA EKMGHLCRDD SVEGLRFYPN LFLTQS
```

5.168 valosin-containing protein [Gallus gallus]

Protein Accession	gi|113206112
Mean Expression Ratio	0.942
Median Expression Ratio	0.941
Credible Interval	(0.821, 1.08)
Associated Peptides	8
Associated Spectra	9
Coverage	0.143

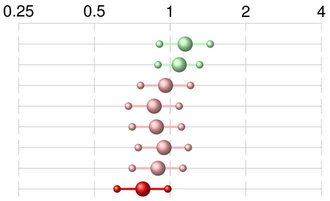

A	2.5	50	97.5	Sequence
1	0.9	1.1	1.4	EVDIGIPDATGR
2	0.9	1.1	1.3	QTNPSAMEVEEDDPVPEIR
1	0.76	0.96	1.2	GPELLTMWFGESEANVR
1	0.68	0.86	1.1	NAPAIIFIDELDAIAPK
1	0.7	0.88	1.1	GVLFYGPPGCGK
1	0.75	0.95	1.2	KYEMFAQTLQQSR
1	0.71	0.9	1.1	KQLAQIK
1	0.61	0.78	0.98	ELQELVQYPVEHPDKFLK

```
1      MASGSDSKAD DLSTAILKQK NRPNRLIVDE AINEDNSVVS LSQAKMDELQ LFRGDTVLLK GKKRREAVCI VLSDDTCSDE
81     KIRMNRVVRN NLRVRLGDVI SIQPCPDVKY GKRIHVLPID DTVEGITGNL FEVYLKPYFL EAYRPIRKGD IFLVRGGMRA
161    VEFKVVETDP SPYCIVAPDT VIHCEGEPIK REDEEESLNE VGYDDIGGCR KQLAQIKEMV ELPLRHPALF KAIGVKPPRG
241    ILLYGPPGTG KTLIARAVAN ETGAFFFLIN GPEIMSKLAG ESESNLRKAF EEAEKNAPAI IFIDELDAIA PKREKTHGEV
321    ERRIVSQLLT LMDGLKQRAH VIVMAATNRP NSIDPALRRF GRFDREVDIG IPDATGRLEI LQIHTKNMKL ADDVDLEQVA
401    NETHGHVGAD LAALCSEAAL QAIRKKMDLI DLEDETIDAE VMNSLAVTMD DFRWALSQSN PSALRETVVE VPQVTWEDIG
481    GLEDVKRELQ ELVQYPVEHP DKFLKFGMTP SKGVLFYGPP GCGKTLLAKA IANECQANFI SIKGPELLTM WFGESEANVR
561    EIFDKARQAA PCVLFFDELD SIAKARGGNI GDGGGAADRV INQILTEMDG MSTKKNVFII GATNRPDIID PAILRPGRLD
641    QLIYIPLPDE KSRVAILKAN LRKSPVAKDV DLDFLAKMTN GFSGADLTEI CQRACKLAIR ESIESEIRRE RERQTNPSAM
721    EVEEDDPVPE IRRDHFEEAM RFARRSVSDN DIRKYEMFAQ TLQQSRGFGS FRFPSGNQGG AGPSQGTGGG SGGNVYSEDN
801    DDDLYG
```

5.169 PREDICTED: hypothetical protein [Gallus gallus]

Protein Accession	gi|50747212
Mean Expression Ratio	0.942
Median Expression Ratio	0.942
Credible Interval	(0.734, 1.21)
Associated Peptides	1
Associated Spectra	1
Coverage	0.00745

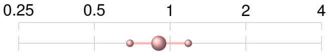

A	2.5	50	97.5	Sequence
1	0.69	0.9	1.2	RLSNTGIK

```
1      MREILPRLYP GQVNDKNNSL TTSPKQSFED KSNPSNGDDD QNYCYQGTEA ENNKLSLISC MKCRNIQKIS KQDLEKHKKL
81     GWAEDKNFIC KMCSHITSPA FHFVPEGANA IDFEKHEKTY QRKKQKAFKV KNFLPGKYYC DKCRFSTKDP LQYKKHVSQH
161    EEIKFICSYC NYISYTKGEF QRHLVKHTGT FPYQCEYCDY GAVRHDYIVK HTRRVHEAPK KRLSNTGIKR QQKKSCLPSQ
241    STLFEKQKYE EITFQNELSN SSSNITCEIP DKATKIVSSS HDIECSISAA SIQNKTILEP SEISMCENQS VEVEVYSPKM
321    EPLQPGMPLT VIAPSELVVP SNCLAQIVEI KIVNGAQQLV LKLIPMKEAA CRPVNCAALG LENQGTERTA EGKKPSVSSN
401    ELLTMEVNVD KLSSISNQLN LDSKYDKNSD YLYSSDSQYS DCNSVSIQKE DSSKLRLHLV KGIDVRSGVI ELCSQSLVTN
481    STEKKCDLKS PKWEADGKNN LHYDLYCYEE GVNTSRPKTA AVSEDKSSKN NSVKTTQGSK NFYSSAVLKQ KDVFPLKRDD
561    KECRSPIVNS AETPLAAKGF DRKPVICSEA EKNFHVTKTS PWEDMTFSFS KVKKAETVSQ KNNLLLESLE LQKMENKNNP
641    FEGPVISSVF SLSSGAANVP ESIRWDDTTR NKKSTTLLCR KIAQLMSAAE SNMKSMPLRC QASSKKMHFP QENSASCATG
721    LEQAMSFPQV HDGSNVIGSN EHSNEQLLTF ARTSKSRVTK NSNVASPVFI PKGTVLRVLN AASSQNSRGI EKMSEASAPS
801    SYCNEMLLPR PVPVSVSEKL SSNLPCLPNQ SDPHTQSRNI SLRQRPKREM NAKTSSKRTV VPYQKNGDVS KQSKHYSKSQ
881    QGPKNKVKQV GYRELPKRKT RTQSETSSGS DMSYLLTARR LRLVPLRMNQ LIKCPRRNQP VVVLNHPDVD TPEIINVMKT
961    INKYKGHVLK VVLSERTSSC LGVKRYRKRL TLQNAETGSQ AKKQSMLKMK LKKTHKNNYQ VVEASPAEPL QCMFKCWFCG
1041   RVYADQEEWI SHGQRHLIEA TKGWDVLSLP VTKH
```

5.170 ATP synthase, H+ transporting, mitochondrial F1 complex, beta subunit [Gallus gallus]

Protein Accession	gi|71897237
Mean Expression Ratio	0.943
Median Expression Ratio	0.943
Credible Interval	(0.756, 1.18)
Associated Peptides	2
Associated Spectra	2
Coverage	0.0582

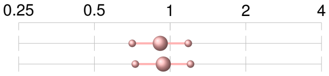

A	2.5	50	97.5	Sequence
1	0.71	0.91	1.2	AIAELGIYPAVDPLDSTSR
1	0.73	0.94	1.2	IMNVIGEPIDER

```
1       MLGLAGRCSA AAASAARPAL RRAAGPSHGF LPLLLSRGAG PAAAVGARRD HAAQAAPAAK AGSATGRIVA VIGAVVDVQF
81      DEGLPPILNA LEVQGRETRL VLEVAQHLGE NTVRTIAMDG TEGLVRGQKV LDSGAPIRIP VGPETLGRIM NVIGEPIDER
161     GPITTKQFAA IHAEAPEFVE MSVEQKILVT GIKVVDLLAP YAKGGKIGLF GGAGVGKTVL IMELINNVAK AHGGYSVFAG
241     VGERTREGND LYHEMIESGV INLKDATSKV ALVYGQMNEP PGARARVALT GLTVAEYFRD QEGQDVLLFI DNIFRFTQAG
321     SEVSALLGRI PSAVGYQPTL ATDMGTMQER ITTTRKGSIT SVQAIYVPAD DLTDPAPATT FAHLDATTVL SRAIAELGIY
401     PAVDPLDSTS RIMDPNIVGP EHYDVARGVQ KILQDYKSLQ DIIAILGMDE LSEEDKLTVA RARKIQRFLS QPFQVAEVFT
481     GHMGKLVPLK ETIKGFKQIL AGEYDHLPEQ AFYMVGPIEE AVAKAEKLAE EHA
```

5.171 PREDICTED: similar to ELKS epsilon isoform 1 [Gallus gallus]

Protein Accession	gi|118083050
Mean Expression Ratio	0.944
Median Expression Ratio	0.943
Credible Interval	(0.73, 1.23)
Associated Peptides	1
Associated Spectra	1
Coverage	0.00643

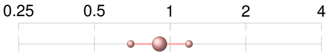

A	2.5	50	97.5	Sequence
1	0.7	0.9	1.2	KIAELER

```
1       MYGSARSVGK AEPSNQSPGR SPRLPRSPRL GHRRTNSTGG SSGSSTGGGS GKTLSMENIQ SLNAAYATSG PMYLSDHENV
81      GADTPKSTMT LGRSGGRLPY GVRMTAMGSS PNIASSGVAS DTIAFGEHHL PPVSMASTVP HSLRQARDNT IMDLQTQLKE
161     VLRENDLLRK DVEVKESKLS SSMNSIKTFW SPELKKERAL RKDEASKITI WKEQYRVLQE ENQHMQMTIQ ALQDELRIQR
241     DLNQLFQQDS SSRTSEPFVA ELTEENFQRL HTEHERQAKE LFLLRKTLEE MELRIETQKQ TLNARDESIK KLLEMLQSKG
321     LSAKATEEDH ERTRRLAEAE MHVHHLESLL EQKEKENNML REEIHRRFEN APDSAKTKAL QTVIEMKDSK ISSMERGLRD
401     LEEEIQMLKS NGALSTEERE EEMKQMEVYR SHSKFMKNKI GQVKQELSRK DTELLALQTK LETLTNQFSD SKQHIEVLKE
481     SLTAKEQRAA ILQTEVDALR LRLEEKETML NKKTKQIQEI AEEKGTQAGE IHDLKDMLEV KERKVNVLQK KIENLQEQLR
561     DKEKQMSSLK DRVKSLQADT TNTDTALTTL EEALAEKERT IERLKEQRDR DEREKQEEID TYKKDIKDLK EKVGILQGDL
641     TEKESSLLDL KEHASSLASS GLKKDSRLKT LEIALEQKKE ECLKMETQLK KAHEATLEAR ASPELSDRMQ QLEREVTRYR
721     EESSKAQAEV DRLLEILKEM ENEKNDKDKK IAELERQVKD QNKKVANLKH KEQVEKKKSA QMLEEARRRE DNLNDSSQQL
801     QDNLRKKDDR IEELEEALRE SVQITAEREM VLAQEESARI NAEKQVEELM MAMEKVKQEL ESMKAKLSST QQSLAEKETH
881     LTNLRAERRK HLEEVLEMKQ EALLAAISEK DANIALLELS SSKKKTQDEV AALKREKDRL VQQLKQQTQN RMKLMADNYE
961     DDHLKSSSHS NQTNHKPSPD QIIQPLLELD QNRSKLKLYI GHLTALCHDR DPLILRGLTP PASYHLDDDR AAWEKELQKM
1041    TQEQLRDELE KGEKESAKLQ EFANAILQQI ADHCPDILEQ VVNALEESS
```

5.172 PREDICTED: similar to damaged-DNA binding protein DDB p127 subunit [Gallus gallus]

Protein Accession	gi|118090978
Mean Expression Ratio	1.06
Median Expression Ratio	1.06
Credible Interval	(0.823, 1.38)
Associated Peptides	1
Associated Spectra	1
Coverage	0.308

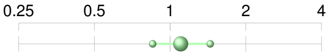

A	2.5	50	97.5	Sequence
1	0.85	1.1	1.4	KTEPATGFIDGDLIESFLDISRPK

```
1       MWIWLLDSAT WRSFHTERKT EPATGFIDGD LIESFLDISR PKMQEVVANL QIDDGSGMKR EATVDDLIKI VEELTRIH
```

5.173 osteoglycin [Gallus gallus]

Protein Accession	gi|45383706
Mean Expression Ratio	1.06
Median Expression Ratio	1.06
Credible Interval	(0.818, 1.38)
Associated Peptides	1
Associated Spectra	1
Coverage	0.0442

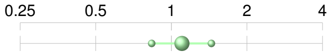

A	2.5	50	97.5	Sequence
**1**	**0.84**	**1.1**	**1.4**	**LLLLEELSLAENR**

```
1       MKTLQAAFFL VAFVPLVKPA PPIQQDSPKF YEYVDADFAT GSLIQQDYEM LPKDTIKDGT NVSLDTALRL QADDSELSAR
81      PTKDTNLPTC LLCVCLSGSV YCEEIDIEAV PPLPKETAYL YARFNKIKRI AVSDFADITT LRRIDFSGNM IEEIEDGAFS
161     KLLLLEELSL AENRLVKLPV LPPKLTTFNA NQNRIKSRGI KNNAFKKLTN LAYLYLGHNA LESVPLNLPE SLRILHLQHN
241     NITTINDDTF CKSNNTRYIR TRMDEIRMEG NPILLAKHVN AFSCLRTLPV GTYY
```

5.174 PREDICTED: similar to KIAA0835 protein [Gallus gallus]

Protein Accession	gi|118100721
Mean Expression Ratio	0.945
Median Expression Ratio	0.943
Credible Interval	(0.738, 1.22)
Associated Peptides	1
Associated Spectra	1
Coverage	0.00776

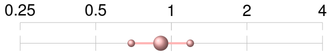

A	2.5	50	97.5	Sequence
**1**	**0.7**	**0.9**	**1.2**	**QSKPLDSIR**

```
1       MPIISMKGEN EFRGELLNQR WTCGEKISSC EGAASLHGTC IKMSLENDDK RARTRSKSVR VPTELISQEV SCPTPGCNGS
81      GHIRGKYARH RSLQSCPLAK KRKLQDAEAE HLVSKRKSHP LKLALDEGYN VDSDGSEEAE VKEESGSEES EGTLEEDEAE
161     VVEQEETRSP EPAEGRSPAK SAHYGQKPAT STSGPSKANY SNYQEIIANS LLNLGQIAEE TLAIEGQLPE AELQVSKPPS
241     NVVHLVHEDA GEEIVEEECD KEIIIQTEDA EEVIEVTSER SSELCPEHQD DTNCEESCKL QKAMQTEAEE EDEEDEDEDE
321     DEDEEEEEEE EEEEEEEEEE EEEEMAPEVI CEEAPHTSQD TQKSHCEGQF SPKPEYSVIV EVRSDDDKDD DAHSQKSAVT
401     DESEMYDMMT RGNLGLLEQA IALKAEQVKI VREPSRLPGE HVKHFQVDEK QSKPLDSIRK SYYGKDPSRP EKREIKCPTP
481     GCDGTGHVTG LYPHHRSLSG CPHKDRIPPE ILAMHENVLK CPTPGCTGQG HVNSNRNTHR SLSGCPIAAA EKLAKSHEKQ
561     QTQSGDPSKP SSNSDRILRP MCFVKQLEIP QYGSYRPNMV PATPRANLAK ELEKYSKVTF DYASFDAQVF GKRMLAPKIQ
641     TSETSPKAFK CFDYSHDAEA AHMAATAILN LSTRCWEMPE NLSTKQQEAP GKSMDIEVDE NGTLDLSMNK NRKRESTFPS
721     SSSCSSSPSV KSPDVSQRQN STSATSSTMT SPQSSQTSRQ DEWDGPIDYT KPNRQREEEP EESEPAAHSF ASSEADEQEV
801     SEENFEERKY PGEVTLTNFK LKFLSKDIKK ELLTCPTPGC DGSGHITGNY ASHRSLSGCP LADKSLRNLM AAHSADLKCP
881     TPGCDGSGHI TGNYASHRSL SGCPRAKKSG IKITPTKDDK EDPELMKCPV PGCVGLGHIS GKYASHRSAS GCPLAARRQK
961     EGSLNGSSFS WKSLKNEGPT CPTPGCDGSG HANGSFLTHR SLSGCPRATF AGKKGKLSGD EILNTKFKTS DVLENDEEIK
1041    QLNKEISELN ESNSEMEAAM VKLQSQISSM EKNLKNIEEE NKIIEEQNEA LFLELSGLSQ ALIQSLANIR LPHMEPISEQ
1121    NFDAYVNTLT DMYTNQECYQ NPENKDLLES IKQAVKGIQV
```

5.175 eukaryotic translation initiation factor 3, subunit 6 interacting protein [Gallus gallus]

Protein Accession	gi|60302722
Mean Expression Ratio	0.943
Median Expression Ratio	0.943
Credible Interval	(0.753, 1.18)
Associated Peptides	2
Associated Spectra	2
Coverage	0.0638

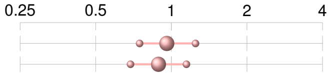

A	2.5	50	97.5	Sequence
**1**	**0.75**	**0.96**	**1.2**	**QLEVYTSGGDPESVAGEYGR**
**1**	**0.69**	**0.89**	**1.1**	**VFADEVQQQAQLSTIR**

```
1      MAYPGEDYDN DAAYDPYAYS NDYDMHTGDP KQDLAYERQY EQQTYQVIPE VIKNFIQYFH KTVSDLIDQK VYELQASRVS
81     SDVIDQKVYE IQDIYENSWT KLTERFFKNT PWPEAEAIAP QVGNDAVFLI LYKELYYRHI YAKVSGGPTL EQRFESYYNY
161    CNLFNYILNA DGPAPLELPN QWLWDIIDEF IYQFQSFSQY RCKTAKKSEE EIDFLRSNPK IWNVHSVLNV LHSLVDKSNI
241    NRQLEVYTSG GDPESVAGEY GRHSLYKMLG YFSLVGLLRL HSLLGDYYQA IKVLENIELN KKSMYSRVPE CQVTTYYYVG
321    FAYLMMRRYQ DAIRVFANIL LYIQRTKSMF QRTTYKYEMI NKQNEQMHAL LAIALTMYPM RIDESIHLQL REKYGDKMLR
401    MQKGDAQVYE ELFSYACPKF LSPVVPNYDN VHPNYHKEPF LQQLKVFADE VQQQAQLSTI RSFLKLYTTM PVAKLAGFLD
481    LTEQEFRIQL LVFKHKMKNL VWTSGISALD GEFQSASEVD FYIDKDMIHI ADTKVARRYG DFFIRQIHKF EELNRTLKKM
561    GQRP
```

5.176 PREDICTED: similar to Phospholipase A2, membrane associated precursor (Phosphatidylcholine 2-acylhy

Protein Accession	gi|50805872
Mean Expression Ratio	1.06
Median Expression Ratio	1.06
Credible Interval	(0.817, 1.36)
Associated Peptides	1
Associated Spectra	1
Coverage	0.0556

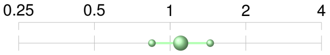

A	2.5	50	97.5	Sequence
1	0.84	1.1	1.4	MIRVTTGK

```
1      MRNLLLALLL ACGLPPARGS MLELERMIRV TTGKSALLSY SWYGCFCGIG GRGTPVDPTD TCCHAHDCCY RRLRGGSCSP
81     LITPYHFDVI DGNITCGDEQ SWCKRETCLC DRAVASCFAS ALPSYNVSYR FYFKLRCRGS KLQC
```

5.177 PREDICTED: similar to Ubiquitin carboxyl-terminal hydrolase 47 (Ubiquitin thioesterase 47) (Ubiquit

Protein Accession	gi|118091184
Mean Expression Ratio	0.946
Median Expression Ratio	0.944
Credible Interval	(0.735, 1.22)
Associated Peptides	1
Associated Spectra	1
Coverage	0.0109

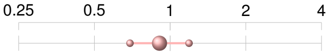

A	2.5	50	97.5	Sequence
1	0.7	0.91	1.2	LYVSLPEQSPGSQFR

```
1      MVPSEENQLV PKEDMFWSCR QSTLAEMKKK FAQVESAAEE PRVLCIIQDT TNSKTVNERV TLNVPASTPL KKLFEDVASK
81     VGYVNGTFDL VWGNGDNVTD MTPIDQNSDK TILDAGFEPG KKNFLHLTDK DGEQPHIMQE ESGTTEDSAQ DRFIGPLPRE
161    GSVGCTNDYV SQSYSYSSVL SKSETGYVGL VNQAMTCYLN SLLQTLFMTP EFRNALYKWE FEESEEDPVT SIPYQLQRLF
241    VLLQTSKKRA IETTDVTRSF GWDSSEAWQQ HDVQELCRVM FDALEQKWKQ TEQADLINQL YQGKLKDYVR CLECGYEGWR
321    IDTYLDIPLV IRPYGSNQAF ASVEEALHAF IQPEILDGPN QYFCERCKKK CDARKGLRFL HFPYLLTLQL KRFDFDYTTM
401    HRIKLNDRMT FPEELDMSIF IDVEDEKSPQ TESCTDSGAE NEGSCHSDQM SNDFSNDDGV DEGICLESNS AAERIAKVGS
481    EKNSLLYELF SVMVHSGSAA GGHYYACIKS FSDDQWYSFN DQHVSKITQE DIKKTYGGSS GSRGYYSSAF ASSTNAYMLI
561    YRLKDPARNA KFLESHEYPD HIKQLVQKER ELEEQEKRQR EIERNTCKIK LFCMHPTKQI MMENKLEVHK DRTLKEAVGI
641    AYKLMDLEEA VPLDCCRLVK YDEFHDYLER SYEGEEDTPM GLLLGGVKST YMFDLLLETR RPDQIFQCYK PGEVMVKVHV
721    VDLKTESVAP PISVRAYLNQ TVSEFKQLIS KATHLPAETM RVVLERCYND LRLLTVSSKT LKAEGFFRSN KVFIESSESL
801    DRHVAYTDSH LWKLLDRHAN TIRLYVSLPE QSPGSQFRRS IYQKPSGDLG NLDEACERVK GPAGNMKSVE AILEESTEKL
881    KSLSLQQQQQ EGDNGDSSKS TEASDFENIE SPSNEIDSSA SVENRELENQ IQISDPENLQ SEERSDSDVN NDRSTSSVDS
961    DILSSSHSSD TLCNVDNAPI PLANGLDSHS ITSSRRSKAN QGKKETWDTA EEDSGTDSEY DESGKSRGET QYMYFKSEPY
1041   TADEGSGEGQ KWLMVHVDKR ITLSAFKQQL EPFVGVPSSH FKVFRVYASN QEFESVRLNE TLSSFSDDNK ITIRLGRALK
1121   KGEYRVKVYQ LLVNEPEPCK FLLDAVFAKG MTVRQSKEEL LPQLREQCGL DLTIDRFRLR KKTWKNPGTV FLDYHIYEED
1201   INISSNWEVF LEILDGVEKM KSMSQLAVLS RRWRPSEMKL DSFQEVVLES SSVEELKEKL SELSGIPLEN IEFAKGRGTF
1281   PCDISVLEIH QDLDWNPKVS TLNVWPLYIC DDGAVIFYRD KTEELMELTD EQRNELMKKE SSRLQKTGHR VTYSPRKEKA
1361   LKIYLDGAPN KDLTQD
```

5.178 PREDICTED: similar to KIAA1571 protein [Gallus gallus]

Protein Accession	gi|118093494
Mean Expression Ratio	1.06
Median Expression Ratio	1.06
Credible Interval	(0.821, 1.36)
Associated Peptides	1
Associated Spectra	1
Coverage	0.0095

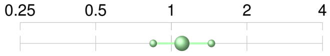

A	2.5	50	97.5	Sequence
**1**	**0.84**	**1.1**	**1.4**	**TPLTSIRGDR**

```
1      MFDRIKPSDE ASASSVQGME RHGVEGSLQQ DSNSSLHSRR QEHPRPGVST MQNRQGYCNC CHVHYSNLEQ HIFSSQHRHF
81     TTYCRNRMGT SSLMERFLQD VLQHHPHRYH DNRPTYDDMP LPVTPVSART ASSAEVEKKK NRGRQVASSK DEESIDSVRS
161    SAPYLSHEHT KETSVTQTFT QKLETEKERV TGTSQMSMGN CSNEKCSVLK DAQVANHSHE GQFTAVSPVP QHFSVNTLVH
241    SSSVNRKIEK NIKTSSSSNR ILHSGCDNTG MEVCSRNELK PALAPVHPKC PSVLHQSPVC SHRKSLHVTS GQSLLKRDGL
321    QTQDETLVSD FCLRDTVGIS RSLGLGTSPP VARCKNVKMN RGDASSVDET IENVILKYCH GTAYEELHFK EENNLCLTFS
401    SLLDNADLEG SEMSFDCDAP IQSGTDLPKA AVKDIELLKE VQINLQDKDY GTQLSSVLKS ESVKQIETVK KNMGVHSEEA
481    VLPALPHVPP SFVGKTWSQI MYEDDIKIEE LVRDFREGRF RCYFDSESSA NCIGKKMKKK KQKDAKRNNT AEGNRTETAS
561    VKALPEFNDA LSGGSDFDNP SLASERVCNP QILKTPRKRT WRLASRCQVV KVSHGTQTSL LNYPVTKRKM TRRESDPSDQ
641    KGSIIWPENE RTPNMKTRLC ALKLPESYSK IMSPVQPKTV VYVLSCPEMK QCKGKPVDIP KMRKNRNSTD SKDSIRYKYK
721    QCSFKYYDPL TNRILKMPPK GSVGEKAKKP SHVRQLFRSL SFDANMRKIA DAQREGTPTK SFSRSDFYSS SSATCLPESG
801    KGNDTVSSQK ADGSSVSTER TDCLVSSHSE NSFKPLVISP LNSHQSVVEG DGRLTPFNSR VTKTPLTSIR GDRLERENPK
881    AIWKRKESTN KEPFFSKKAA GAMSVRCAVA RRGNRVTTGK QTSRTKKQQK EGMRRKLSPC AQKSSAFPIH RHQTRKTTVG
961    KHLKKEKPDA KKLNVRRKSK RTFLNSTVTS IPEKRQKGTS GSFPKKPERA SSKVRSWEVS GDRGHPSTVN RPSRRTSALP
1041   LLRNCIVLPG ES
```

5.179 PREDICTED: similar to taxilin [Gallus gallus]

Protein Accession	gi|118101716
Mean Expression Ratio	0.943
Median Expression Ratio	0.944
Credible Interval	(0.732, 1.22)
Associated Peptides	1
Associated Spectra	1
Coverage	0.0355

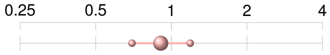

A	2.5	50	97.5	Sequence
**1**	**0.7**	**0.9**	**1.2**	**EGSEAAAGGAPAEQR**

```
1      MKNQGGDTKA APRPAAKTSG ALGEEGDPSE PTAALEVPKS LLLLFHNVEL ILTWKRWLEE HRNSQKQMKI LQKKQTQLVQ
81     EKDHLQSEHS KAILARSKLE SLCRELQRHN RTLKEEGVQR AREEEEKRKE VTSHFQVTLN DIQLQMEQHN ERNSKLRQEN
161    MELAERLKKL IEQYELREEH IDKVFKHKDL QQQLVDAKLQ QAQEMLKEAE ERHQREKDFL LKEAVESQRM CELMKQQETH
241    LKQQLALYTE KFEEFQNTLS KSSEVFTTFK QEMEKMTKKI KKLEKETTMY RSRWESSNKA LLEMAEEKTL RDKELEGLQV
321    KIQRLEKLCR ALQTERNDLN KKVQDLCAHV PRADMDLTEP LKDPSREGSE AAAGGAPAEQ RLAEDCLHSG KPTHSTDPAE
401    SLGELSIGAL GQSGTEEGTQ GAD
```

5.180 hypothetical protein [Gallus gallus]

Protein Accession	gi|53128989
Mean Expression Ratio	0.943
Median Expression Ratio	0.945
Credible Interval	(0.752, 1.18)
Associated Peptides	2
Associated Spectra	2
Coverage	0.188

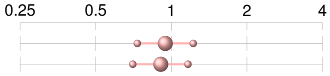

A	2.5	50	97.5	Sequence
**1**	**0.73**	**0.94**	**1.2**	**LGGSLVVAFEGSPV**
**1**	**0.7**	**0.9**	**1.2**	**KEELMFFLWAPEQAPLK**

```
1      MASGVQVADE VCRIFYDMKV RKCSTPEEVK KRKKAVIFCL SPDKKCIIVE EGKEILVGDV GVTVTDPFKH FVQMLPEKDC
81     RYALYDASFE TKESKKEELM FFLWAPEQAP LKSKMIYASS KDAIKKKFQG IKHECQANGP EDLNRACIAE KLGGSLVVAF
161    EGSPV
```

5.181 PREDICTED: cardiomyopathy associated 3 [Gallus gallus]

Protein Accession	gi|118085527
Mean Expression Ratio	0.945
Median Expression Ratio	0.945
Credible Interval	(0.788, 1.13)
Associated Peptides	4
Associated Spectra	4
Coverage	0.0254

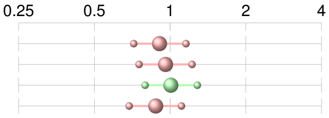

A	2.5	50	97.5	Sequence
1	0.72	0.91	1.2	NLQQESEIQSLISAQAEQGAAESAPR
1	0.75	0.96	1.2	GGFPGYCSLSTPSPQNPR
1	0.8	1	1.3	WIFETQPLDAIR
1	0.69	0.88	1.1	NIQHHVQSK

```
1     MAEPQKSSKV AIKKMEDDLP PPPIPGSIQV IAPASQDPNP LPVPPPKQAF SKFYQQRQVN ELKRLYRHMH PELRKNLEEA
81    VTEDLAEMLN TEDPNAQGSV NLDKVLPGEV QSMRWIFENW ALDSIGDHQV TKKMMEDEII PGGDVKSTSL RFENQSVNGD
161   YLSTTAKVSE TDLARGDVHT ARWLFETQPL DSLNKLYSDE TEMQEAVLKE PVQGGDVKGA KELFEAQSLD AIGRCCSVEE
241   KSILQLKSEI QELKGDVKKT IRLFQTEPLC AIRDKTGNIH EIKSVCREEI QSNAVRTARW LFETQPLDTI NKDTSKVQII
321   RGISLEEIGR PDVSGARWIF ETQPLDAIRE ITVEEQDFKA STDFVTGADV TKQRLLFETQ ALDSLKGEAS ESVAAKEQVI
401   GGDVKSTLWL FETQPMETLK DNVEVGHLKK VELSAEEKGD VKQRKHVFET CPLGSISKAF EEEVSAASTE EVVKGDVKSF
481   KTLFETLPLD SIKEVDAEPI TKEEEKIPPG NVKANQILFE TTPLYAIKDS FGNFHEVTSV SREQVISGDV KKYKWMFETR
561   PLDQFDESTK KVDIIRGITK QEVVAGDVRT AKWLFETQPM DVIHHQATQG EEHPSMKREI SQRGDVKTCR WLFETQPMHT
641   LYEKAEKKQE EDVSVPQADV KSYTWMFETQ PLDSLKGQEE QYLRVSKAYS QDELQGVDVK TVRHLFETEP LGSSVVSEAD
721   QKKTLRYSSR VEIQSGEVSR VKEFFEAKPL DTTTKPTAVI KDDGTIEAGS VHKFTWLFEN YPMDTLKDSS EGIQEIPPEK
801   DIKGGDVGGK RFIFETYSLD QIHDKVDETE LHKIQKDTMS KANVKSCTML FESQPLYAIQ DKEGGYHEVT SVQKEEIMKG
881   DVKGARWLFE TKPLDQIKKE EEVFVIRAVT QEDIKKGDVQ AARWRFETEP LDSFPGGKIS VPRTVDDVQK GDVQSNKQLF
961   ESQQVGQKKY VRMVSVSDVQ RGDVRTSTWL FENQPVDSLY GDADRSSSIS TVQREDSQKG DVKRCTWLFE TQPMDTLKDP
1041  EVTVSTGTQE PIPRADVKST TWLFESTPLD KFSASECSRE TELKERTMRE TLETLCTCQA IQHDGILIEA NDTESVKMVK
1121  YQLSSPGAPE ILKEEIVRGH LQGIMLQLLH RTNVEPQSVL VEEDREGKIK VSSLQLLDQS EAIKGKEDLS GNVAKALQSL
1201  LSQDASIKKG MVIQETKSES VKMTLYSLLF HSVQQKVVKG DVKSTIGNLM ASSQEQRATV TVKREDNEKG NVQLFASCIE
1281  KGDLDYLKNL QQESEIQSLI SAQAEQGAAE SAPRALQSTN THVLANKEQV EKVMAEAKSG ALEGAKMVFA CESTGKEGAL
1361  EREVVHAVGV TGTTVQCLGK PQNLPTGMEK EEIMSGGLKV TTKSIQRVAD VSKNTEKEES ISACLKEPKA TMQGIAQAKV
1441  TAERNEVVGE QQSLVTEQAS QKQSEEKVLG NDLQAAMQSL RLATAEARNI QHHVQSKLQR NREEVHMACR QQVASKQETK
1521  TLQSTIHQQE SASTMRENTS TAIRTSTTRV QEASRTHTSV SQKSIASHKK VSASEEVQGG QLLSQENQVV PSRDVSIKDG
1601  LYTATPVKTY INPFVESDYK EQSVQEERDV IIRGDVQTAI RALQSAATEQ RLVEKEDIVR GNLKATLQSL EKSNVNVSKG
1681  DFKAAMIYRN AGQSYSVCKK KSETQVNNNQ TAVVASGSQA DNDFPPPPPV AVMKAEHCPP STKATREGAP PLLTSKDEAP
1761  GCFSPLQTPL PPPPSLSCKP SDQNSTEKPK IPPKPEITAP LRKKPVPPPK PEHLLHEAYS ASTNNSTNRS TKSVPPPVPP
1841  KPPGLREISM PKPPPAELQL SCTEVCEQSD HRESQDKCCT LESSMDKSIT VQGPERKLPK YTAKTPLQMA EERYKARKGG
1921  QGKFELDRAK PSKPVKNGEV GFEAKQGMLN GKAAASISCS HEVVQRHSEM CQQEERFSSV SYPACPGRAQ TPKMPGQTES
2001  STSSVGHVSP PKRGDDIAKN ASAKVERECV YNAYSSWESQ RVMQEVNERK QTSHSMSFHQ QPKNASKEEQ QENSEQPKYP
2081  DVDAEASAQE KPAVIMREKS KRETEDERRK RLSVHKEEIM KGNVKEAMEI FENLRRQEEL QEILTRVKEF EEETSKVDVK
2161  SLRSLFEKVP DWVVRQKALQ AKQQDGAETQ AKDDTDSISS VEMVFGDLER ASAEIIHLKE QTLARLLDIE EAIKKALYSV
2241  SSLKSESDIA GLSGLFKESL GSTQSSTSSS NIRKISIVSS KARQEGTTVE TGEAASAEGG KVAEKTEVTK SELEVPRQVH
2321  PRVSSPSSPS YITIESAARK PESPRTAHSP RDIPSPDCPD TPGKKDAFAQ ESSNSVNHPL AGSPGRDTSP FEKRPEPTQT
2401  NAGDNSVKQH TPANTNNQVN EKEQCPPDAS KGSCQCGVKG GFPGYCSLST PSPQNPRRQK SILELKTGPD GSKLYGATRT
2481  VTEQYEEMDQ FGNKIITSST TVTKQSETQT SSTCGVASHP QYEVSASPVF PSYLKSPGEN IHYGIFQESG VVFVTFGNSR
2561  LKK
```

5.182 mitochondrial trifunctional protein, alpha subunit [Gallus gallus]

Protein Accession	gi|45384238
Mean Expression Ratio	1.06
Median Expression Ratio	1.06
Credible Interval	(0.819, 1.37)
Associated Peptides	1
Associated Spectra	1
Coverage	0.0182

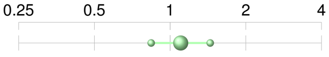

A	2.5	50	97.5	Sequence
1	0.84	1.1	1.4	TIEYLEEVAIGFAR

```
1     MAAAVRAYRQ LGALRAAAKY CPPRRPALEG YACRNVSTST ALQARTHVSY DIKGDVAVVR FNTPNSKVNT LSKQLNAEFT
81    EVMNEIWTNE AVKSAVLISS KPGSFIAGAD IDMIAACKTS QEVTQLSQEG QKMLEKIEQS PKPIVAAISG SCLGGGLEVA
161   IACHYRIATK DRKTILGTPE VLLGLLPGAG ATQRLPKMVG LPAAFDMMLT GRNINADRAK KMGLVDQLVD PLGPGVKPPE
241   ARTIEYLEEV AIGFARGLAN KTVSAKRSKG LVQRITDYAM ALPFVRQQVY KTVENKVQKQ TKGLYPAPLK IIEVVKAGLD
321   QGHDTGYLTE SQSFGHLAMT NESKALIGLY HGQVRCKKNK FGTPQREVKT LAVLGAGLMG AGIAQVSVDK GLKTILKDTT
401   QQGLDRGQQQ VFKGLNGKVK KKSLTSFERD SILSNMTAQL DYKGFEKADM VIEAVFEDIS IKHKVLKEVE AVIPPHCIFA
481   SNTSALPINQ IAAVSQRPEK VIGMHYFSPV DKMQLLEIIT TDKTSQDTAA SAVAVGLKQG KVVIVVKDGP GFYTTRCLGP
561   MLAEVGRVLQ EGIDPKKVDA ISTAFGFPVG AATLIDEVGV DVATHVAEDL GKAFGERFGG GSIGLFKLMV QKGFLGRKAG
641   KGFYIYQEGV KNRSLNSGMD EILAQFKLPA KPEVCTDEDI QMRLVTRFVN EAAMCLQEGI LSNPMEGDIG AVFGLGFPPC
721   LGGPFRYADS YGAKQLVDKL RKYEAVYGSQ FTPCQLLLDY ANSPGKKFYQ
```

5.183 PREDICTED: similar to Clathrin, heavy polypeptide (Hc) [Gallus gallus]

Protein Accession	gi|118098353
Mean Expression Ratio	0.946
Median Expression Ratio	0.946
Credible Interval	(0.733, 1.22)
Associated Peptides	1
Associated Spectra	1
Coverage	0.00718

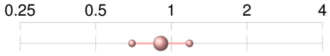

A	2.5	50	97.5	Sequence
**1**	**0.7**	**0.91**	**1.2**	**VNQLLLVKPYLR**

```
1       MAQILPIRFQ EHFQLQSLGI NPANIGFSTL TMESDKFICI REKVGEQAQV VIIDMSDPAT PIRRPISAES AIMNPASKVI
81      ALKAGKTLQI FNIEMKSKMK AHTMAEEVIF WKWISVNTVA LVTETAVYHW SMEGESQPQK MFDRHASLAG CQIINYRTDE
161     HQKWLLLIGI SAQQNRVVGA MQLYSVDRKV SQPIEGHAAA FAEFKIEGNA KPSTLFCFAV RSPAGGKLHI IEVGQPATGN
241     QPFVKKAVDV FFPPEAQTDF PVAMQIGIKH GVIYLITKYG YIHVYDLESG VCIYMNRISA DTIFVTASHE PTSGIIGVNK
321     KGQVLSVCVE EDNIVNYATN VLQNPDLGLR MAIRSNLAGA EELFARKFNT LFAQGSYADA AKVAASAPKG ILRTSDTIRK
401     FQSVPAQPGH ASPLLQYFGI LLDQGQLNKF ESLELCRPVL QQGRKQLLEK WLKEDKLECS EELGDLVKTA DPTLALSVYL
481     RANVPNKVIQ CFAETGQFQK IVLYAKKVGY TPDWIFLLRS VMRVSPEQGL QFSQMLVQDE EPLANINQIV DVFMENSLIQ
561     QCTSFLLDAL KNNRPAEGHL QTRLLEMNLI HAPQVADAIL GNQMFTHYDR AHIAQLCEKA GLLQRALEHY TDLYDIKRAV
641     VHTHLLNPEW LVNFFGSLSV EDSVECLRAM LSANIRQNLQ LCVQVASKYH EQLGTQSLVE LFESFKSYEG LFYFLGSIVN
721     FSQDPDVHFK YIQAACKTGQ IKEVERICRE SNCYNPERVK NFLKEAKLTD QLPLIIVCDR FDFVHDLVLY LYRNSLQKYI
801     EIYVQKVNPS RIPAVVGGLL DVDCSEDVIK NLIMVVRGQF STDELVAEVE KRNRLKLLLP WLESRIHEGC EEPATHNALA
881     KIYIDSNNNP ERFLRENPYY DSRVVGKYCE KRDPHLACVA YERGQCDLEL IKVCNENSLF KSEARYLVRR KDPELWANVL
961     EENNPFRRQL IDQVVQTALS ETQDPEEVSV TVKAFMTADL PNELIELLEK IVLDNSVFSE HRNLQNLLIL TAIKADRTRV
1041    MEYINRLDNY DAPDIANIAI SNELYEEAFA IFRKFDVNTS AVQVLIEHIG NLDRAYEFAE RCNEPAVWSQ LARAQLQKDL
1121    VKEAIDSYIK ADDPSAYMEV VQAANRNDNW EDLVKFLQMA RKKARESYVE TELIFAFAKT NRLSELEEFI SGPNNAHIQQ
1201    VGDRCYEEGM YEAAKLLYNN VSNFARLAST LVHLGEYQAA VDSGRKANST RTWKEVCFAC VDGREFRLAQ ICGLHIVIHA
1281    DELEELISYY QDRGYFEELI ALLEAALGLE RAHMGMFTEL AILYSKFKPQ KMREHLELFW SRVNIPKVLR AAEQAHLWAE
1361    LVFLYDKYEE YDNAIITMMN HPTDAWREGQ FKDIIAKVAN VELYYKALQF YLDYKPLLIN DLLLVLSPRL DHTRTVNFFS
1441    KVNQLLLVKP YLRSVQNHNN KGVNEALNNL LTEEEDYQGL RASIDAYNNF DNITLAQRLE KHELIEFRRI AAYLYKGNNR
1521    WKQSVELCKK DHLYKDAMQY AAESKDVELA EKLLQWFLEE GKQECFAACL FTCYDLLHPD VVLELAWRHN IMDFAMPYFI
1601    QVMREYLTKV DNLDASESLR KEEEQVTEPT PIVFGQQLML TAGPSAVPPQ TNFPYGFTAP GFTQPPVYGF NV
```

5.184 S-phase kinase-associated protein 1A isoform b [Homo sapiens]

Protein Accession	gi|25777713
Mean Expression Ratio	0.947
Median Expression Ratio	0.947
Credible Interval	(0.743, 1.20)
Associated Peptides	1
Associated Spectra	2
Coverage	0.0736

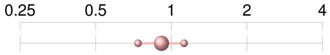

A	2.5	50	97.5	Sequence
**2**	**0.74**	**0.91**	**1.1**	**NDFTEEEEAQVR**

```
1       MPSIKLQSSD GEIFEVDVEI AKQSVTIKTM LEDLGMDDEG DDDPVPLPNV NAAILKKVIQ WCTHHKDDPP PPEDDENKEK
81      RTDDIPVWDQ EFLKVDQGTL FELILAANYL DIKGLLDVTC KTVANMIKGK TPEEIRKTFN IKNDFTEEEE AQVRKENQWC
161     EEK
```

5.185 tubulin alpha chain - chicken (fragment)

Protein Accession	gi|71575
Mean Expression Ratio	0.947
Median Expression Ratio	0.947
Credible Interval	(0.85, 1.06)
Associated Peptides	12
Associated Spectra	25
Coverage	NaN

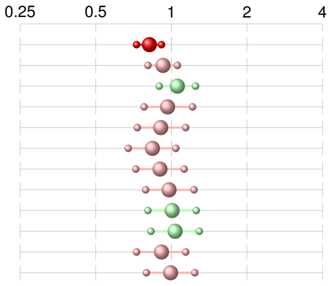

A	2.5	50	97.5	Sequence
8	0.73	0.82	0.92	AVFVDLEPTVIDEVR
5	0.8	0.93	1.1	FDGALNVDLTEFQTNLVPYPR
3	0.9	1.1	1.2	TIGGGDDSFNTFFSETGAGK
1	0.78	0.97	1.2	EDAANNYAR
1	0.73	0.91	1.1	AVCMLSNTTAIAEAWAR
1	0.67	0.84	1.0	LIGQIVSSITASLR
1	0.72	0.9	1.1	VGINYQPPTVVPGGDLAK
1	0.79	0.98	1.2	YMACCLLYR
1	0.81	1	1.3	AFVHWYVGEGMEEGEFSEAR
1	0.83	1.0	1.3	QLFHPEQLITGK
1	0.73	0.91	1.1	QLFHPEQLITGKEDAANNYAR
1	0.8	1	1.2	LDHKFDLMYAK

5.186 myristoylated alanine-rich C kinase substrate (MARCKS) [Gallus gallus]

Protein Accession	gi|45382723
Mean Expression Ratio	1.06
Median Expression Ratio	1.06
Credible Interval	(0.923, 1.21)
Associated Peptides	7
Associated Spectra	14
Coverage	0.495

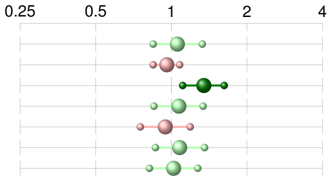

A	2.5	50	97.5	Sequence
1	0.85	1.1	1.3	QEAPSESSPEGPAEPAE
7	0.85	0.96	1.1	AAEEAGAAATSEAGSGEQEAAPAEEPAAAR
2	1.1	1.3	1.6	EAAEAESTEPASPAEGEASPK
1	0.85	1.1	1.3	ATGEEAPAAEEQQQQQQEK
1	0.75	0.95	1.2	EEAAAAAPEAAGGEEGK
1	0.86	1.1	1.4	TEEGATPSSSSETPKK
1	0.82	1.0	1.3	GEAAAEKPGEAVAASPSK

```
1       MGAQFSKTAA KGEAAAEKPG EAVAASPSKA NGQENGHVKV NGDASPAAAE AGKEEVQANG SAPAEETGKE EAASSEPASE
81      KEAAEAESTE PASPAEGEAS PKTEEGATPS SSSETPKKKK KRFSFKKSFK LSGFSFKKNK KEAGEGAESE GGAAAAAEGG
161     KEEAAAAAPE AAGGEEGKAA AEEASAAAAG SREAAKEEAG DSQEAKSDEA APEKATGEEA PAAEEQQQQQ QQEKAAEEAG
241     AAATSEAGSG EQEAAPAEEP AAARQEAPSE SSPEGPAEPA E
```

5.187 putative coat protein [Chickpea yellow mosaic virus]

Protein Accession	gi|33327347
Mean Expression Ratio	0.947
Median Expression Ratio	0.947
Credible Interval	(0.729, 1.22)
Associated Peptides	1
Associated Spectra	1
Coverage	0.0319

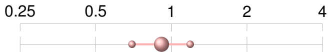

A	2.5	50	97.5	Sequence
1	0.7	0.91	1.2	NAKESYMPR

```
1       ADQQRAELDA GAQREKKEKD KDKDKNTSTQ SGSSDQGKAL TTTNRDRDVN AGTSGPFSIP RIKAMTSKLN IPRIKGKEVV
81      NLDHLLIYEP KQVDLSNTRA THSQFEFWHT AVCEGYGIQE SDSQVIMNGL MVWCIENGTS PNINGNWTMM DGEEQVEYPL
161     KIVIENAKPT FRQIMAWFSD LAEAYIEKRN AKESYMPRYG QIRNLRDKSL ARYAFDFYEI TSKTPDRARE AHLQMKAAAL
241     KNTQNKMFGL DGSVGNAEES TERHTVEDVT SNMHSMLGVR NF
```

5.188 PREDICTED: hypothetical protein [Gallus gallus]

Protein Accession	gi|50728360
Mean Expression Ratio	0.947
Median Expression Ratio	0.948
Credible Interval	(0.731, 1.22)
Associated Peptides	1
Associated Spectra	1
Coverage	0.0229

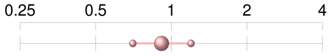

A	2.5	50	97.5	Sequence
1	0.7	0.92	1.2	KDEEKEDPK

```
1      MADIDNKEQS ELDQQDMEDV EDVEEEETGE DANSKARQLT VQMMQNPQIL AALQERLDGL VGTSTGYIES LPKVVKRRVN
81     ALKNLQVQCA QIEAKFYEEV HELERKYAAL YQPLFDKRSE IINAIYEPTE EECEWKADAE EEISEEMKEK AKLEEEKKDE
161    EKEDPKGIPE FWLTVFKNVD LLSDMVQEHD EPILKHLKDI KVKFSEVGQP MSFTLEFHFE PNDYFTNEVL TKTYRMRSEP
241    DDSDPFSFDG PEIMGCTGCQ IDWKKGKNVT LKTIKKKQKH KGRGTVRTVT KTVSNDSFFN FFSPPEVPES GDLDDDAEAI
321    LAADFEIGHF LRERIVPRSV LYFTGEAIED DDDDYDEEGE EADDEEGEEE ADEENDPDFD PKKDQNPAEC KQQ
```

5.189 testin [Gallus gallus]

Protein Accession	gi|45382899
Mean Expression Ratio	1.05
Median Expression Ratio	1.06
Credible Interval	(0.816, 1.36)
Associated Peptides	1
Associated Spectra	1
Coverage	0.0332

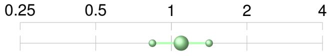

A	2.5	50	97.5	Sequence
1	0.84	1.1	1.4	EKQPVAGSEGAQYR

```
1      MDLESKVKKM GLGHEQGFGA PCLKCKDKCE GFELHFWRKI CRNCKCGQEE HDVLTSNEED RKVGKLFEDT KYTTLIAKLK
81     NDGIPMYKRN VMILTNPVPA KKNISINTVT YEWAPPVQNQ TLARQYMQML PKEKQPVAGS EGAQYRKKQL AKQLPAHDQD
161    PSKCHELSPN EVKQMEQFVK KYKNEALGVG DVKLPGELET KATDKNNVNS GDRSTSAAVG AMEDKSADQK ASQYSCYRCK
241    LNMKEGDPAV YAERAGYDKL WHPACFVCCT CSELLVDMIY FWKNGNLYCG RHYCDSEKPR CAGCDELIFS NEYTQAEGQN
321    WHLKHFCCFD CDCVLAGEIY VMVNDKPVCR PCYVKKHAAI CQGCHNAIDP EVQRVTYNNF NWHATQECFL CSCCSKCLIG
401    QKFMPVEGMV FCSVECKKKM MS
```

5.190 zinc finger protein 85 (HPF4, HTF1) [Gallus gallus]

Protein Accession	gi|71897351
Mean Expression Ratio	1.06
Median Expression Ratio	1.05
Credible Interval	(0.815, 1.37)
Associated Peptides	1
Associated Spectra	1
Coverage	0.0224

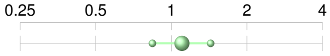

A	2.5	50	97.5	Sequence
1	0.84	1.1	1.4	QSSCVSEGESR

```
1      MAKGHPFQEP VSFAEVAVYF SREEWALLDP AQRALYRDVM LETYHCVASL VPLPVHKPLV ITLLEGGKEP WIPDVCSPEK
81     MVEELGPADV TTAMKEDVQK SKVSERQSSC VSEGESRRDV QGALEQGEHF KKPLGNHTGR TARNPLDLST AQKQHEEVRS
161    KEVCQKRRQN PCDECGKSCE SSSDLIVHQH IQTGERPYKC SECEKSFKRS SHLSYHQRIH TGERPYKCPE CGKGFTGSSD
241    LSRHQLIHTA ERPFKCHECG KTFKSRSDLS RHQRIHTGET PYSCPQCLQS FRSSYDLTHH QCIHKGERPY KCPECGKSFT
321    SNSVLVRHQR IHTGERAFKC PECGKSFKSS SGLTYHQRIH TGERPFKCSE CGKGFTSSSD LTHHQRIHTG ETPFKCPKCG
401    KSFTRSSALR CHQRIHRGER PFKCSDCGKG FTSSSDLIYH QCFHIGEKTY RCPECGKGFR SSSHLSVLQL ILTQERPFMC
481    SECERTSKAV
```

5.191 kinesin heavy chain member 2A [Gallus gallus]

Protein Accession	gi|86129530
Mean Expression Ratio	1.05
Median Expression Ratio	1.05
Credible Interval	(0.809, 1.36)
Associated Peptides	1
Associated Spectra	1
Coverage	0.0162

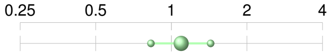

A	2.5	50	97.5	Sequence
**1**	**0.83**	**1.1**	**1.4**	**RPLNRKETLMK**

```
1      MVTSLNEDNE SVTVEWIENG DTKGKEIDLE SIFSLNPDLA PDEDIEPSPE TQPLPAPSAK VNKIVKGRRT VAPVKNDTPA
81     RDNRVASSAR ARPTQLPEQS SSSQQNGTVS GISPVQAAKK EFGPPSRRKS NCVKEVEKLQ EKREKRRLQQ QELREKRAQD
161    VDATNPNYEI MCMIRDFRGN LDYRPLTTAD PIDEHRICVC VRKRPLNRKE TLMKDLDVIT IPSKDVVMVH EPKQKVDLTR
241    YLENQTFRFD YAFDETAPNE MVYRFTARPL VETIFERGMA TCFAYGQTGS GKTHTMGGDF SGKNQDCSKG IYALAARDVF
321    LMLKKPNYKK LELQVYATFF EIYSGKVFDL LNRKTKLRVL EDGKQQVQVV GLQEREVKCV EDVLKLIEIG NSCRTSGQTS
401    ANAHSSRSHA VFQIILRRKG KLHGKFSLID LAGNERGADT SSADRQTRLE GAEINKSLLA LKECIRALGR NKPHTPFRAS
481    KLTQVLRDSF IGENSRTCMI ATISPGMASC ENTLNTLRYA NRVKELTIDP SAAGDIRPII HHTPSQIDDL DTQWGVGSSP
561    QRDDLKLLCE QNEEEVSPQL FTFHEAVSQM VEMEEQVVED HRTVFQESIR WLEDEKALLE MTEEVDYDVD SYATQLEAIL
641    DQKIDILTEL RDKVKSFRAA LQEEEQASKQ INPKRPRAV
```

5.192 PREDICTED: similar to RP11-163B6.3 [Gallus gallus]

Protein Accession	gi|118099494
Mean Expression Ratio	1.05
Median Expression Ratio	1.05
Credible Interval	(0.814, 1.36)
Associated Peptides	1
Associated Spectra	1
Coverage	0.00586

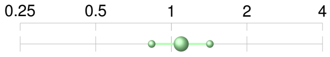

A	2.5	50	97.5	Sequence
**1**	**0.83**	**1.1**	**1.4**	**SSQFVPR**

```
1      MPVQAAQWTE FLSCPICYNE FDENVHKPIS LGCSHTVCKT CLNKLHRKAC PFDQTAINTD IDVLPVNFAL LQLVGAQVPD
81     HQTVKLSNVG ENKHYEVAKK CVEDLALYLK PLSGGKGVAS LNQSALSRPM QRKLVTLVNC QLVEEEGRVR AMRAARSLGE
161    RTVTELILQH QNPQQLSANL WAAVRARGCQ FLGPAMQEEA LKLVLLALED GSALSRKVLV LFVVQRLEPR FPQASKTSIG
241    HVVQLLYRAS CFKVTKRDED SSLMQLKEEF RSYEALRREH DAQIVHIAME AGLRISPEQW SSLLYGDLAH KSHMQSIIDK
321    LQSPESFAKS VQELTIVLQR TGDPANLNRL RPHLELLANI DPNPDAASPT WEQLENAMVA VKTVVHGLVD FIQNYSRKGH
401    ETPQPQPNSK YKTSMCRDLR QQGGCPRGTN CTFAHSQEEL EKYRLRNKKI SATVRTFPLL NKVGVNSTVS TTTGSVISVI
481    GSPEATVKMV PSTNGIANLE SGVPQLIPRC ADTSLRALEN TKKAGKSGAN GQNVSGSPTE SLPENKIGSP PKTPVSQAAA
561    TSAGPPNIGT EVNSVPPKSS QFVPRVPVYP AHSDNVQYFQ DPRTQLSYEV PQYPQTGYYP PPPTVPAGVA PCVPRFVRSN
641    NVPESSLPPA SVPYADHYST FPPRDRLNSP YQPPPPQPYG PVPPVPSGMY APVYDSRRIW RPQMYPRDDI IRSNSLPPMD
721    VMHSSVYQTS LRERYNSLDG YYSVACQPPN EQRTVPLPRE PCGHLKTGYD EQLRRKPEQW AQYHTQKTPL VSSTLPMATP
801    SPTPPSPLFS VDFNTEFSES VNDLSGTKFE EDHLSHYSPW SCGTIGSCIN AIDSEPKDVI ANSNAVLMDL DSGDVKRRVH
881    LFETQRRAKE EDPIIPFSDG PIISKWGAIS RSSRTGYHTT DPIQATASQG SATKPISVSD YVPYVNAVDS RWSSYGSDSA
961    SSARYAERDR FIVTDLSGHR KHSSTGDLLS IELQQAKSNS LLLQREANAL AMQQKWNSLD EGSRLTLNLL SKDIDLRNGE
1041   VKQGTDYSED CADTKPDRDI ELELSALDTD EPDGQGEQIE EILDIQLGIS SQDDQLLNGT TVENGHPLKQ HQKESMEQKR
1121   QSLGEDLVIL EEQKTILPVT SCFSQPITTS VSNASCLPIS TSVSVGSLIL KTAHIMSEDK NDFLKPVANG RMVNS
```

5.193 acid phosphatase 1, soluble [Gallus gallus]

Protein Accession	gi|86129490
Mean Expression Ratio	0.95
Median Expression Ratio	0.95
Credible Interval	(0.737, 1.22)
Associated Peptides	1
Associated Spectra	1
Coverage	0.114

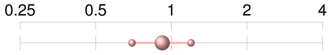

A	2.5	50	97.5	Sequence
**1**	**0.7**	**0.92**	**1.2**	**IDSAATSTYEIGNPPDYR**

```
1       MAAGEVKSVL FVCLGNICRS PIAEAVFRKL VTDEKVENKW RIDSAATSTY EIGNPPDYRG QTCMKKHGIT MNHIARQVTK
81      DDFQTFDYIL CMDESNLRDL KRKSNQVKDC KAKIELLGAY DPQKQLIIED PYYGNEKDFE TVYEQCVRCC KAFLEKPH
```

5.194 mitogen-activated protein kinase kinase 3 [Gallus gallus]

Protein Accession	gi|61098276
Mean Expression Ratio	0.95
Median Expression Ratio	0.95
Credible Interval	(0.756, 1.20)
Associated Peptides	1
Associated Spectra	2
Coverage	0.0543

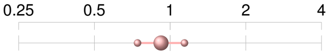

A	2.5	50	97.5	Sequence
2	0.74	0.92	1.1	QVVEEPSPQLPPER

```
1       MSVKRIRATV NTQEQKRLLM DLDISMRTVD CFYTVTFYGA LFREGDVWIC MELMDTSLDK FYKKVLEKKK TIPEDILGKM
81      AVSIVRALEH LHSKLSVIHR DVKPSNVLIN KEGHVKMCDF GISGYLVDSV AKTMDAGCKP YMAPERINPE LNQKGYNVKS
161     DVWSLGITMI ELAILRFPYE SWGTPFQQLK QVVEEPSPQL PPERFSKEFV DFTAQCLRKN PAERMNYLEL MEHPFFTLHD
241     TKETDMASFV TEILGDDS
```

5.195 PREDICTED: similar to p167 [Gallus gallus]

Protein Accession	gi|118093099
Mean Expression Ratio	1.05
Median Expression Ratio	1.05
Credible Interval	(0.88, 1.26)
Associated Peptides	4
Associated Spectra	4
Coverage	0.0339

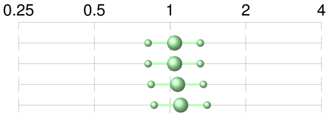

A	2.5	50	97.5	Sequence
1	0.82	1.0	1.3	EDAPLGPQLQSMPSEQIR
1	0.82	1.0	1.3	LATLVPFVDAFQLER
1	0.84	1.1	1.4	EKEELEQR
1	0.87	1.1	1.4	ALDDDRPPRR

```
1       MPVYFQRPEN ALKRANEAAR ERSGAARPRG TGPGLPPHGA ARREEAQAAA RRGPGSAYRP RRGPRGRRRR AGSERRREAA
81      AAVGPGPAEG GGRRGDGLRL RSARRLVPSS ASGLASPAAG ESNQSGPRRC FSLLAARLAI PNRVSAPEFL EVGKKQPALD
161     VLYDVMKSKK HRTWQKIHEP IMLKYLELCV DLRKSHLAKE GLYQYKNICQ QVNIKSLEDV VRAYLKLAEE KTEAAKEESQ
241     QMVLDIEDLD NIQTPESVLL SAVSGEDTQD RTDRLLLTPW VKFLWESYRQ CLDLLRNNSR VERLYHDIAQ QAFKFCLQYT
321     RKAEFRKLCD NLRMHLGQIQ RHHNQSTAIN LNNPDSQSMH LETRLVQLDS AISMELWQEA FKAVEDIHGL FALSKKPPKP
401     QLMANYYHKV STVFWKSGNA LFHASTLHRL YHLSREMRKN LTQEEMQRMS TRVLLATLSI PITPERTDIA RLLDMDGIIV
481     EKQRRLATLL GLQAPPTRGS LINDMVRFNV VQYVVPEVKE LYNWLEVDFH PLKLCSRVSK VLNWVKDQAE KEPELQLYVP
561     HLQNNTILRL LQQVAQIYQS IEFSRLATLV PFVDAFQLER SIVDAARHCD LQVRLDHTTR TLSFGSDLNY STREDAPLGP
641     QLQSMPSEQI RNQLTAMSSA LAKALAVIKP PHLLQEKEEQ HQLAVTAFLK NSRKEHQRIL ARRQTIEERK ERLESLNIQR
721     EKEELEQREA ELQKVRKAEE ERLRQEAKER EKERILQEHE QIKKKTVRER LEQIKKTELG AKAFKDIDIE DLEELDPDFI
801     MAKQVEQLEK EKKELQERLK NQEKKIDYFE RAKRLEEIPL IKTAYEEQRV RDMELWEQQE EERITTLQLE REKALEHKNR
881     LSRMLEDRDL FEARLKALRR TVYEDKLKQF QERLAEERRN RLEERKKQRK EERRITYYRE KEEEEQRLRE EQLLKEREEK
961     ERIEREKREQ EQREYQERVK KLEELEKKKR QREMEIEERE RRREEERRGL DDPFSRKESR WGDRGESESS WRRGGETESE
1041    WRRAPVERDW RRGEARDDER PFRRGDDLPR RSDDLPRRGD DLPRRGPAEE KERPSVESSE DRPPRREGDE DRPPRREGDE
1121    DRPLRRGLDE DRPPRRGLDD DRGSWRAADD DRGPRRGMDD DRPPRRALDD DRPPRRALDD DRPPRRALDD DRPPRRSLDD
1201    DRGSWRAADD DRGPRRGLDD DRPPRRALDD DRPPRRGLDD DRGSWRAADD DRGPRRGLDD DRGPRRGLDD DRGPWRNTDD
1281    DRLSRRDDDR GPWRSSEDSR PGPWRPFGKP GGWREREKAR EDSWGPPRDS RPPGDREWDR DKDRDDNEKD REFDRERDFD
1361    RDDRFRRPRD DAGWRRGPAE ETSSWRDSSR REEWDRGGRD MRDRRADDRE PPLRRGPPLR SDREEPSSWR RADDRREERG
1441    EERETVRRSA PAPAAPPASA PPSASKDRER DGEKEKGSWK TEKEREPARR TKNETDEEGW TTVRR
```

5.196 PREDICTED: similar to natural killer tumor recognition protein [Gallus gallus]

Protein Accession	gi|118085464
Mean Expression Ratio	0.95
Median Expression Ratio	0.95
Credible Interval	(0.736, 1.22)
Associated Peptides	1
Associated Spectra	1
Coverage	0.00957

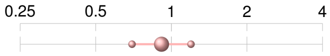

A	2.5	50	97.5	Sequence
**1**	**0.7**	**0.92**	**1.2**	**SQECGFSTRGXASR**

```
1       MGVQDRPQCF FDIEINREPV GRIMFQLFSD ICPKTCKNFL CLCSGEKGIG KTTGKKLCYK GTTFHRVVKN FMIQGGDFSE
81      GNGKGGESIY GGYFKDENFI LKHDRAFLLS MANRGKHTNG SQFFITTKPA PHLDGVHVVF GLVISGFEVI EQIENLKTDT
161     ASRPYADVRV IDCGVLVTKP AKDALEKKKK VCSDSEASDS SSSGSDPSET SSESEAEDER SRRRKRKRRA KTKQSRKRRK
241     EERKKEDLRS KRASNQRCLS DKSDVTEKAV DVSTKRDKPV VRPEEIPPVP ENRFLLRRDV PVVSTEPEPK LLDATPVLTD
321     QKPSVSKSGR KIKGRGTIRY HTPPRSRSCS ESDDDESSET PPHWKEEMQR LRTYRPPSGE KWSKGDKLSD PCTSRWDERS
401     ASRRSRSWSH NGYSDLSTVR YSSHHKKHRK KKVKHKKKSK KQKHYKKHKQ AKKKKASASS EVESSRSFRK KKSSCDRERK
481     SRSSSLSSRR SSRRDWSKSD KEDQSSSTFS SRESRSYYRS RSRSRSRSKS RSYSQRSSRS RSASKSSHSR SRSRSSSNPR
561     HQKTVSNSPR NISTRLNENK LNKPADPVRA VILPSDKVVI PPVVPESLPV IPLSDSPPPS RWKPGQKPWK PSYERIQEMK
641     AKATHLIPPQ TNYNLVVIKE ANTSSYQKQQ GSSDSDRSGY SKYRSDKSSD SWQRSRSRSS RSRSYSRSLT TEEYXKSVXL
721     KDKISFFWQI TITEQIPQXQ VRVXXVNILF FPXXXXXTQK QKKIYIRXSE HSFSXSEARN KLXKYPSLXG YERLRXIFSR
801     SEREXQFIIF RLLYXQRAFW XSKGSPRKGR PFSIRRXCSA TGXKYLPFCE RGGEIQAXMG CXSFXKESSX RAIFXATXRW
881     CKKXTKILFM XXMGLXIKLR KGRGSSWXGR FQALVXXRGR RGHIRIXYRT XCYQKEKNKI QFLRRRSGFX XCMEDKQTVI
961     IFRIXEFSFX LNTYQRKVKK TQTXIEKNSX KITFQKSKRK IKREKGEETQ SSEKKRSVSL ATPTGVWXRR XRRYKXKASY
1041    QGXQRKAAYQ GXXNSQRQNR KWXKAXCRXK PASXKYYSXC LTKSKXAXXR XHQSKHFNQY IKLRNKCDYL QXXDXACXRK
1121    QPEWIGCNSD RXQHGDLYSR SXLTREGGCG HFVSCHSHXX TSEFRCXYKQ RFTDLXTRCC QTRKQPCRLH XYXRRNRKAR
1201    KXLCSCIXCX RQQFEKXNCX KYTKQFNXXX MEALARCWXF ATSYSKYCHR SQECGFSTRG XASRFKNXNK SXKXSKAWIS
1281    VXXSXEDSTT KSKAKEPRKF QRGGVSKXRX QXSIQEPQXV SKXIRIXIQT PNTVXIVQSL KKSIPKLYIF LQVEKLYEKQ
1361    KXRLVQQRPV KKPEXFLPQL QKSXSNLXXK QIQKXFLWSP QSIQVIHLXX LLXQKSKXKE XQLSKIXKLX QKIQDLXILW
1441    LXXRKRSQLL QQPKSQXEQQ IQL
```

5.197 poly(A) binding protein, cytoplasmic 1 [Gallus gallus]

Protein Accession	gi|71896197
Mean Expression Ratio	0.95
Median Expression Ratio	0.95
Credible Interval	(0.762, 1.19)
Associated Peptides	2
Associated Spectra	2
Coverage	0.0424

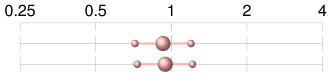

A	2.5	50	97.5	Sequence
**1**	**0.72**	**0.93**	**1.2**	**NLDDGIDDER**
**1**	**0.73**	**0.95**	**1.2**	**SLGYAYVNFQQPADAER**

```
1       MNPSAPSYPM ASLYVGDLHP DVTEAMLYEK FSPAGPILSI RVCRDMITRR SLGYAYVNFQ QPADAERALD TMNFDVIKGK
81      PVRIMWSQRD PSLRKSGVGN IFIKNLDKSI DNKALYDTFS AFGNILSCKV VCDENGSKGY GFVHFETQEA AERAIEKMNG
161     MLLNDRKVFV GRFKSRKERE AELGARAKEF TNVYIKNFGE DMDDERLKEL FGKFGPALSV KVMTDESGKS KGFGFVSFER
241     HEDAQKAVDE MNGKELNGKQ IYVGRAQKRV ERQTELKRKF EQMKQDRITR YQGVNLYVKN LDDGIDDERL RKEFSPFGTI
321     TSAKVMMEGG RSKGFGFVCF SSPEEATKAV TEMNGRIVAT KPLYVVALAQR KEERQAHLTN QYMQRMASVR AVPNPVINPY
401     QPAPPSGYFM AAIPQTQNRA AYYHTNQLAQ LARPSPRWTA QGARPHPFQN MPGAIRPAAP RPPFSTMRPA SSQVPRVMST
481     QRVANTSTQT MGPRPAAAAT AATPAVRTVP QYKYAAGVRN PQQHLNTQPQ VAMQQPAVHV QGQEPLTASM LASAPPQEQK
561     QMLGERLFPL IQSMHPTLAG KITGMLLEID NSELLHMLES PESLRSKVDE AVAVLQAHQA KEAAQKAVNN PTGVPSV
```

5.198 PREDICTED: similar to KIAA0066 [Gallus gallus]

Protein Accession	gi|118093847
Mean Expression Ratio	1.05
Median Expression Ratio	1.05
Credible Interval	(0.817, 1.36)
Associated Peptides	1
Associated Spectra	1
Coverage	0.0142

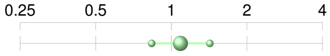

A	2.5	50	97.5	Sequence
**1**	**0.83**	**1.1**	**1.4**	**EGNKEPVNLKPEGR**

```
1      MAADSEPESE VFEITDFTTA SEWERFISKI EEVLNDWKLI GISSGKPLEK GVYTTGVWEE KSDEISFADF KFSVTHHYLV
81     QEPGDRDGRE EGVEDALPLP MQDLLCMNND FPPRAHCLVR WYGLREFVVI APAANNDAVL SESKCNLLLS SVSIALGNTG
161    CQVPLFVQIH QKWRRMYVGE CQGPGVRTDF EMVHLRKVPS QYTHLSGLLD VFKSKIGCPL TPLPPVSMAI RLTYVLQDWQ
241    QYFWPQQPPD IDALVGGEVG GLEFGKLPFG ACEDPISELH LATTWPHLTE GIIVDNDVYS DLDPIQAPQW SVRVRKADNP
321    QCLLGDFLNE LFKLCRRKES TDEILGRSAF EEDGKEVADI THALSKLTEP APVPIHKLSV TNMVHSAKKK IRKHRGVDES
401    PLNNEVLNTI LLFLFPDAAD KLAEGFESRA STSSGSNPPP ENEDYNLFSQ FKSAPSDSLT YKLALCLCMI NFYHGGVKGV
481    AHLWQEFVLE MRYRWENNYL IPGLANGPPD LRCCLLHQKL QMLNCCIERK KARDEGKKGN MSDRSPGGSS GDTGKVADHS
561    GDNLKETDKE VGKSWESWSD SEEEFFECLS DTEDLKGNGQ ENGKKGGTKE GNKEPVNLKP EGRLHPHGKL TLLHPGEPLY
641    IPITQEPAPM TEDLLEEQSE VLAKLGTSAE GAHLRARMQS ACLLSDMESF KAANPGCCLE DFVRWYSPRD YIEEEVVDEK
721    GNVVIKGELS ARMKIPSNMW VEAWETAKPV PARRQKRLFD DTREAEKVLH YLAVQKPADL ARHLLPCIIH AAVLKVKEEE
801    VLENISSVKK IIKQIISHSS KVLRFPNPED KKLEEIIAQI MSVEAIIARA RSLKAKFGVE KCENEEEKED LQRFVNCLLE
881    QPEVSVLGAG RGPAGSVIHK LFVNAQRISA VPPLDEELRR SGPSDERRLN AGSVSDFPPP TGREIILRTT VPRPAPYSKP
961    LPQRMYSVLT KEDFRLAGAF SADTTFF
```

5.199 PREDICTED: hypothetical protein [Gallus gallus]

Protein Accession	gi|118091572
Mean Expression Ratio	0.952
Median Expression Ratio	0.952
Credible Interval	(0.765, 1.19)
Associated Peptides	2
Associated Spectra	2
Coverage	0.0595

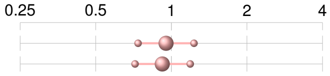

A	2.5	50	97.5	Sequence
**1**	**0.74**	**0.95**	**1.2**	**SPDQSPVPLVVR**
**1**	**0.71**	**0.92**	**1.2**	**HHIVEVAGDDKYGR**

```
1      MAADPLSELQ DDLNLDDTNQ SLSQLKLASI DDKNWPADED PAFPKSEDSK GSPEAVTHLR WDDPYYDIAR HHIVEVAGDD
81     KYGRKVILFS ACRMPPSHQL DHVKLLGYLK FTLDQYVESD YTLVYLHHGL TSENKPSLSW LRDAYREFDR KYKKNIKALY
161    IVHPTMFIKT LLILFKPLIS FKFGRKIFYV NFLSELEEYV KLEQLGIPSQ VLKYDEYLRS LQKPSQVPQK PTPPRPPLPN
241    QQFGVSLQHL REKSPDQSPV PLVVRETIAH LQEHALATEG IFRRSANTQV VKEVQQKYNM GVPVDFQQYE DVHLPAVILK
321    TFLRELPEPL LTFGLYSHIV SFQSVEEVNR VDVVRKTLQN LPEENYHVLR LLTAFLVQVS AHSDRNKMTN TNLAVVFGPN
401    LLWAKDAAIT LKAINPINTF TKFLLDHQKE LFEDVEA
```

5.200 PREDICTED: similar to 5-nucleotidase domain containing 2 [Gallus gallus]

Protein Accession	gi|118096688
Mean Expression Ratio	1.05
Median Expression Ratio	1.05
Credible Interval	(0.823, 1.33)
Associated Peptides	1
Associated Spectra	2
Coverage	0.0366

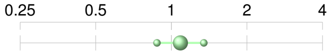

A	2.5	50	97.5	Sequence
**2**	**0.88**	**1.1**	**1.3**	**GAEPGPVAGGQPAPSEGADTR**

```
1       MAALGIAALR RGGGAAPRLL AVAVSCHGCR ARSGSGPADG GHGQPREPLP GGRARRGAEP GPVAGGQPAP SEGADTRSYL
81      WARYHEMKKL VYDLLSPQGF ANLLNPAAIN ANNEISLGDV EIYGFDYDYT LAQYSNLLHS MIFNTARDIL IEQFKYPEGL
161     GKYDYIPGFA IRGLHYDVQK SLLMKIDAFH YVQLGTAYRG LKPVPDEEVI ELYGGTQHIP LYQMSDFYGK GPSLKQFMDI
241     FSLPEMTLLS SVIDYFITHG IEFDQVHLYK DISDAIRDVH VKGVMYKWIE KDMEQYILHG DEIYAVLNRL VSHKKKLFLI
321     TNSPFSFVDK GMKHMVGKNW RDLFDMVIVQ ADKPNFFTDR RKPFRKLDDN GSLQWDKINQ LEKGKIYKEG NLFDFLRLTG
401     WRGSKVLYFG DHLYSDLADL MLRHGWRTGA IVPELETEIR IINTEQYMHS LTWQQALTGL LERMQMYQDA ESKQVLLEWM
481     KERQEIRSLT KNLFNPQFGS IFRTFHNPTY FSRRLVRFSD IYMASISCLL NYDVNFTFYP RRTPLQHEAP LWMDQLCTGC
561     MKTPFLEEMV HIR
```

5.201 eukaryotic translation elongation factor 2 [Gallus gallus]

Protein Accession	gi|45382453
Mean Expression Ratio	0.953
Median Expression Ratio	0.952
Credible Interval	(0.816, 1.12)
Associated Peptides	6
Associated Spectra	6
Coverage	0.0897

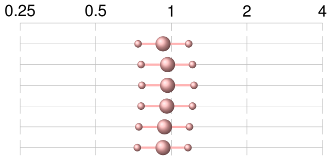

A	2.5	50	97.5	Sequence
1	0.74	0.93	1.2	AYLPVNESFGFTADLR
1	0.76	0.96	1.2	IMGPNYTPGKK
1	0.76	0.97	1.2	NMSVIAHVDHGK
1	0.76	0.96	1.2	DLEEDHACIPIKK
1	0.74	0.94	1.2	KEDLYLKPIQR
1	0.73	0.93	1.2	LDSEDKDKEGKPLLK

```
1       MVNFTVDQIR AIMDKKANIR NMSVIAHVDH GKSTLTDSLV CKAGIIASAR AGETRFTDTR KDEQERCITI KSTAISLFYE
81      LSENDLAFIK QSKDGSGFLI NLIDSPGHVD FSSEVTAALR VTDGALVVVD CVSGVCVQTE TVLRQAIAER IKPVLMMNKM
161     DRALLELQLD PEELYQTFQR IVENVNVIIS TYGEGESGPM GNIMIDPVLG TVGFGSGLHG WAFTLKQFAE MYVAKFAAKG
241     DAQMNPTERA KKVEDMMKKL WGDRYFDPAT GKFSKSATGP DGKKLPRTFC QLILDPIFKV FDAIMTFKKE EAAKLIEKLD
321     IKLDSEDKDK EGKPLLKAVM RRWLPAGDAL LQMITIHLPS PVTAQKYRCE LLYEGPPDDE AAIGIKNCDP RGSLMMYISK
401     MVPTSDKGRF YAFGRVFSGL VSTGLKVRIM GPNYTPGKKE DLYLKPIQRT ILMMGRYVEP IEDVPCGNIV GLVGVDQFLV
481     KTGTITTFEH AHNMRVMKFS VSPVVRVAVE AKNPADLPKL VEGLKRLAKS DPMVQCIIEE SGEHIIAGAG ELHLEICLKD
561     LEEDHACIPI KKSDPVVSYR ETVSEESNVM CLSKSPNKHN RLYMKARPFP DGLAEDIDKG EVSARQELKQ RARYLAEKYE
641     WDVTEARKIW CFGPDGTGPN ILTDITKGVQ YLNEIKDSVV AGFQWATKEG VLCEENMRGV RFDVHDVTLH ADAIHRGGGQ
721     IIPTARRCLY ACVLTAQPRL MEPIYLVEIQ CPEQVVGGIY GVLNRKRGHV FEESQVAGTP MFVVKAYLPV NESFGFTADL
801     RSNTGGQAFP QCVFDHWQIL PGDPFDSASR PSQVVAETRK RKGLKEGIPA LDNFLDKL
```

5.202 thymosin beta 4 [Gallus gallus]

Protein Accession	gi|47604946
Mean Expression Ratio	1.05
Median Expression Ratio	1.05
Credible Interval	(0.842, 1.31)
Associated Peptides	2
Associated Spectra	2
Coverage	0.444

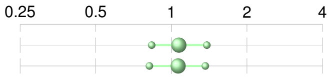

A	2.5	50	97.5	Sequence
1	0.83	1.1	1.4	NPLPSKETIEQEK
1	0.82	1.1	1.4	KTETQEK

```
1       MSDKPDMAEI EKFDKSKLKK TETQEKNPLP SKETIEQEKQ AGESK
```

5.203 pyruvate kinase, muscle [Gallus gallus]

Protein Accession	gi|45382651
Mean Expression Ratio	1.05
Median Expression Ratio	1.05
Credible Interval	(0.905, 1.22)
Associated Peptides	5
Associated Spectra	10
Coverage	0.136

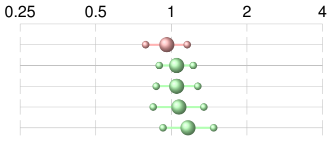

A	2.5	50	97.5	Sequence
2	0.79	0.96	1.2	NTGIICTIGPASR
4	0.9	1.0	1.2	FGVEQNVDMVFASFIR
2	0.87	1.1	1.3	GSGTAEVELK
1	0.85	1.1	1.4	AGKPIICATQMLESMIK
1	0.92	1.2	1.5	LNFSHGTHEYHEGTIK

```
1      MSKHHDAGTA FIQTQQLHAA MADTFLEHMC RLDIDSEPTI ARNTGIICTI GPASRSVDKL KEMIKSGMNV ARLNFSHGTH
81     EYHEGTIKNV REATESFASD PITYRPVAIA LDTKGPEIRT GLIKGSGTAE VELKKGAALK VTLDNAFMEN CDENVLWVDY
161    KNLIKVIDVG SKIYVDDGLI SLLVKEKGKD FVMTEVENGG MLGSKKGVNL PGAAVDLPAV SEKDIQDLKF GVEQNVDMVF
241    ASFIRKAADV HAVRKVLGEK GKHIKIISKI ENHEGVRRFD EIMEASDGIM VARGDLGIEI PAEKVFLAQK MMIGRCNRAG
321    KPIICATQML ESMIKKPRPT RAEGSDVANA VLDGADCIML SGETAKGDYP LEAVRMQHAI AREAEAAMFH RQQFEEILRH
401    SVHHREPADA MAAGAVEASF KCLAAALIVM TESGRSAHLV SRYRPRAPII AVTRNDQTAR QAHLYRGVFP VLCKQPAHDA
481    WAEDVDLRVN LGMNVGKARG FFKTGDLVIV LTGWRPGSGY TNTMRVVPVP
```

5.204 peroxiredoxin 6 [Gallus gallus]

Protein Accession	gi|86129578
Mean Expression Ratio	1.05
Median Expression Ratio	1.05
Credible Interval	(0.862, 1.28)
Associated Peptides	3
Associated Spectra	3
Coverage	0.170

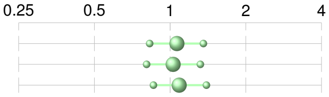

A	2.5	50	97.5	Sequence
1	0.83	1.1	1.4	VVFIFGPDKK
1	0.8	1.0	1.3	LPFPIIADKDR
1	0.86	1.1	1.4	FHDFLGDSWGILFSHPR

```
1      MPGLLLGDEA PNFEADTTQG GIRFHDFLGD SWGILFSHPR DFTPVCTTEL GRAAKLAPEF SKRNVKMIAL SIDSVPDHLA
81     WSKDINAYNG DQPVEKLPFP IIADKDRELA VKLGMLDPDE RDKDGMPLTA RVVFIFGPDK KLKLSILYPA TTGRNFDEIL
161    RVVDSLQLTA YKKVATPVDW KCGDSVMVVP TLPDEEAKKL FPKGVFTKDL PSGKKYLRYT PQPE
```

5.205 PREDICTED: similar to neuronal transmembrane protein Slitrk4 [Gallus gallus]

Protein Accession	gi|118089517
Mean Expression Ratio	1.05
Median Expression Ratio	1.05
Credible Interval	(0.82, 1.36)
Associated Peptides	1
Associated Spectra	1
Coverage	0.0184

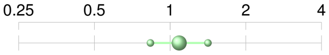

A	2.5	50	97.5	Sequence
1	0.83	1.1	1.4	ISAIRGEVFRNLTNLR

```
1      MTTEKHFLFS FLLSSLDTLS LFRSLRLLAD HKKMFLWLFL VLSSPVSSTT ADADISVEIC SVCSCVSVEN VLYVNCEKVA
81     VYRPNQLKPP WSNFYHLNFQ NNLLIILYPN TFLNFTHAVS LQLGNNKLQN IEGGAFLGLS ALKQLHLNNN ELKILRADTF
161    LGIENLEYLQ ADYNLIKSIE RGAFNKLHKL KVLILNDNLI SFLPDNIFRF ASLTHLDIRG NRIQKLPYIG VLEHIGRIVE
241    LQLEDNPWNC TCDLLPLKAW LENMPYNIYI GEAICETPSD LYGRLLKETN KQELCSMGTG SDFDVRILPP SQLEPGYSTP
321    NGHTTQTSVH RLVTKPPKTT NPSKISGIVA GKALSNRNLS QIISYQTRVP PLTPCPVPCV CKTHPSDLGL SVNCQERNIE
401    SMAELLPKPL NAKKLHVNGN YIKDVDTADF TEFEGLDLLH LGSNRISAIR GEVFRNLTNL RRLYLNGNQI ERLSPEMFAG
481    LHNLQYLYLE YNVIKEILAG TFDLMPNLQL LYLNNNLLRS LPAYIFAGAP LARLNLRNNH FMYLPVSGVL DQLKSLTQID
561    LEGNPWDCTC DLVALKLWLE KLSEGIVVKE LKCETPVQFA NIELRSLKNE ILCPKLLNKP SALLTSPVPA VSFTTPLGPV
641    RSHPGGPVPL SILILSILVV LILTVFVAFC LLVFVLRRNK KPTVKHEGIG NQECSTMQLQ LRKHDHKSNK KDGLGAEAFI
721    PQTIEQMSKS HTCGLKESET GFTFPDPPGQ KVILRNVNDK EKDLLHVDSR KRLSTIDELD ELFPGRDSNV FIQNFLESKK
801    EYNSIGVSGF EIRYPEKQQD KKNKKSLIGG NHSKIVVEQR KSEYFELKAK LQGSPDYLQV LEEQTALNKI
```

5.206 pyruvate dehydrogenase kinase, isoenzyme 3 [Gallus gallus]

Protein Accession	gi|57525478
Mean Expression Ratio	0.952
Median Expression Ratio	0.953
Credible Interval	(0.738, 1.23)
Associated Peptides	1
Associated Spectra	1
Coverage	0.0394

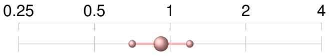

A	2.5	50	97.5	Sequence
1	0.71	0.92	1.2	TTPEADDWSNPSSEPR

```
1       MRLCGALLKS PIPKQIEYYS RFSPSPLSIK QFLDFGRDNA CEKTSYMFLR KELPVRLANT MREVNLLPDN LLNRPSVGLV
81      QSWYMQSFLE LLEYENKSPE DPHVLDDFLD VLIKVRNRHN DVVPTMAQGV IEYKEKYGFD PFVSSNIQYF LDRFYTNRIS
161     FRMLINQHTL LFGGDINPAH PKHIGSIDPN CNVAEVVKDA YETAKMLCEQ YYLVAPDLEV EEFNAKAPNK PIQVVYVPSH
241     LFHMLFELFK NSMRATVELH EGKREGYPSI KTLVTLGKED LSIKISDQGG GVPLRKIDRL FNYMYSTAPR PSLEPTRAVP
321     LAGFGYGLPI SRLYARYFQG DLKLYSMEGV GSDAVIYLKA LSSESFERLP VFNKSAWRHY KTTPEADDWS NPSSEPRDAS
401     KYKANR
```

5.207 Lysyl-tRNA synthetase [Gallus gallus]

Protein Accession	gi|71895483
Mean Expression Ratio	1.05
Median Expression Ratio	1.05
Credible Interval	(0.816, 1.36)
Associated Peptides	1
Associated Spectra	1
Coverage	0.0186

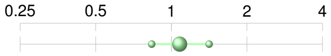

A	2.5	50	97.5	Sequence
**1**	**0.83**	**1.1**	**1.4**	**YLDLILNDYVR**

```
1       MAAAAEREFD AADAESHLSK NELKRRLKAE RKIAEKEAKH KEQSEKHPNK PSLATDSEDN IGADEESLDP NQYYKIRSHA
81      IQQLKGTNED PYPHKFHVDL SLSDFIERYS HLQPGDHLTD ITVSVAGRIH AKRASGGKLI FYDLRGEGVK LQVMANSRLY
161     KSEEEYFRIN NKLRRGDIIG VVGNPGKTKK GELSIIPYEI TLLSPCLHML PHLHFGLKDK ETRYRQRYLD LILNDYVRQK
241     FITRAKIVTY IRSFLDELGF LEIETPMMNI IPGGAMAKPF ITYHNELDMK LYMRIAPELY HKMLVVGGLD RVYEIGRQFR
321     NEGIDLTHNP EFTTCEFYMA YADYRDLMEI TEKLLSGMVK HITGSYKITY HPDGQDGQAY EIDFTPPFRR ISMVDELEKT
401     LGVKFPSTES FETEETRRFF DDLCAVRNVE CPPPRTTARL LDKLVGEFLE VTCINPTFIC DHPQIMSPLA KWHRIHRGLT
481     ERFELFVMKK EVCNAYTELN DPFQQRQLFE DQAKAKAAGD DEAMFIDENF CTALEYGLPP TAGWGMGIDR FTMFLTDSSN
561     IKEVLLFPAM KPEDKKKEGH LEQPAEGTSV
```

5.208 PREDICTED: hypothetical protein [Gallus gallus]

Protein Accession	gi|118097631
Mean Expression Ratio	1.05
Median Expression Ratio	1.05
Credible Interval	(0.877, 1.26)
Associated Peptides	3
Associated Spectra	5
Coverage	0.0464

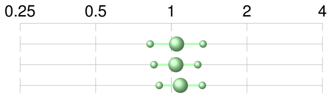

A	2.5	50	97.5	Sequence
**1**	**0.82**	**1.0**	**1.3**	**FLEMCDGLLAR**
**2**	**0.85**	**1.0**	**1.3**	**FQESEERPK**
**2**	**0.9**	**1.1**	**1.3**	**AKDEQYDHLDEADVAKVEK**

```
1       MSVVGIDLGF QSCYVAVARA GGIETIANEY SDRSTPSCIA FGPKNRSIGA AAKSQVISNA KNTVQSFKRF HGRAFSDPFV
81      QAEKTSLAYE LVQLPTGSTG IKVMYMEEER NFTIEQMTGM LLTKLKETAE NALKKPVVDC VVSVPCFYTD AERRSVMDAT
161     QIAGLNCLRL INESTAVALA YGIYKQDLPA LEEKPRNVVF VDMGHSAYQV SICAFNKGKL KVLATAFDTT LGGRKFDEML
241     VEYFCEEFGK KYKLDIKSKI RALLRLYQEC EKLKKLMSAN ASDLPMNIEC FMNDIDVSGT MNRSKFLEMC DGLLARVEAP
321     LRSVLEQAKL KKEDIYAVEI VGGTTRIPAV KEKISKFFGK EVSTTLNADE AVARGCALQC AILSPAFKVR EFSITDLIPY
401     PISLRWNSPA EEGLSDCEVF PKNHAAPFSK VLTFYRKEPF TLEAYYSSPK ELPYPDPAIA HFLVQKVTPQ TDGSSSKVKV
481     KVRVNIHGIF SVSSASLVEV HKSDENEEPM ETDQHAKEEE KMQVDQEEQQ KTEEQQQAQA ENKAESEEME TSQGDSKDKK
561     VDQPPQAKKA KVKTTTVDLP IENQLVWQIG KDMLNLFIEN EGKMIMQDKL EKERNDAKNA VEEYVYDMRD KLCSIYEKFV
641     SEDDRNSFTL KLEDTENWLY EDGEDQPKQI YIDKLTELKA LGQPIQARFQ ESEERPKAFE DLGKQIQQYM KTVHAFKAKD
721     EQYDHLDEAD VAKVEKSANE AMEWMNNKLN LQNKRSLTLD PVIKAKDIQA KTKELTSICN PIVTKPKPKV ELPKEEQKPT
801     EPNGPVEGQG DGSSGSQTAD PSTAPAPAAA EKKLPEMDID
```

5.209 hypothetical protein LOC734509 [Xenopus laevis]

Protein Accession	gi|147901081
Mean Expression Ratio	1.05
Median Expression Ratio	1.05
Credible Interval	(0.814, 1.36)
Associated Peptides	1
Associated Spectra	1
Coverage	0.0462

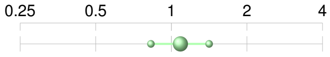

A	2.5	50	97.5	Sequence
1	0.83	1.1	1.4	ILGFFF

```
1      MVRMNVLADA LKSINNAEKR GKRQVLIRPC SKVIVRFLTV MMKHGYIGEF EIIDDHRAGK IVVNLTGRLN KCGVISPRFD
81     VQLKDLEKWQ NNLLPSRQFG YIVLTTSAGI MDHEEARRKH TGGKILGFFF
```

5.210 heterogeneous nuclear ribonucleoprotein M [Gallus gallus]

Protein Accession	gi|71896353
Mean Expression Ratio	1.05
Median Expression Ratio	1.05
Credible Interval	(0.812, 1.36)
Associated Peptides	1
Associated Spectra	1
Coverage	0.0155

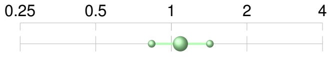

A	2.5	50	97.5	Sequence
1	0.83	1.1	1.4	AFITNIPFDVK

```
1      MAAAAAVAAA TESAGGKMEE PPAAAAPPPP NGAGGAGDPP AKSEGERPSQ NEKRKEKSVK RGGNRFEPYA NPAKRYRAFI
81     TNIPFDVKWQ SLKDLVKEKV GEVTYVELLM DAEGKSRGCA VVEFKMEESM KKAAEVLNKH SLGGRPLKVK EDPDGEHARR
161    AMQKVMAAGG MGIGPGPGGP GMINIPPSIL NNPNIPNEII HALQAGRLGS TVFVANLDYK VGWKKLKEVF SMAGAVVRAD
241    ILEDKDGKSR GIGTVTFEQA IEAVQAISMF NGQLLFDRPM HVKMDERAFP KGDFFPPERP QQLPRMGMEG MGFGMNKMGG
321    MEGPFGGMEN IGRFPAGMNM GRMSEMDRAM GGGFEREFGR NEMGMSRSFG ETLERGIGGG NASIPGIERM APGIDRMGSG
401    IERIPSGMGH GMERVGSEID RMGLVLDRMS SNVDRIGSGI DRMAPLGIDH IAPNIERMGP AIERMGSGIE RMGSGIGFGI
481    ERMGAAIDRV GTTMDRMGSG VERMGSGMDR MGIGMERMVP AGMGTGMGQV IERMPAGLDR IGATPMERIG IDRMGAASME
561    RMGLERIGAT NMERMGPAMG QGMGAGIDRM GLAMGSNFER TMEMERGNFA GNFAGSLGGT GGPAAGVARK ACQIFVRNLP
641    FDFTWKMLKD KFNECGHVLY ADIKMENGKS KGCGVVRFES PEVAERACRM MNGIQLRGRE IDVRIDRNA
```

5.211 PREDICTED: eukaryotic translation elongation factor 1 alpha 2 [Gallus gallus]

Protein Accession	gi|118100661
Mean Expression Ratio	1.05
Median Expression Ratio	1.05
Credible Interval	(0.871, 1.27)
Associated Peptides	3
Associated Spectra	4
Coverage	0.067

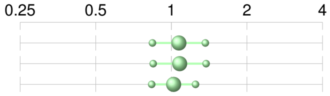

A	2.5	50	97.5	Sequence
1	0.84	1.1	1.4	QTVAVGVIK
1	0.85	1.1	1.4	KLEDNPK
2	0.84	1.0	1.2	THINIVVIGHVDSGK

```
1      MGKEKTHINI VVIGHVDSGK STTTGHLIYK CGGIDKRTIE KFEKEAAEMG KGSFKYAWVL DKLKAERERG ITIDISLWKF
81     ETTKYYITII DAPGHRDFIK NMITGTSQAD CAVLIVAAGV GEFEAGISKN GQTREHALLA YTLGVKQLIV GINKMDSTEP
161    AYSEKRYDEI VKEVSAYIKK IGYNPATVPF VPISGWHGDN MLEPSPNMPW FKGWKVERKE GNASGVSLLE ALDTILPPTR
241    PTDKPLRLPL QDVYKIGGIG TVPVGRVETG ILRPGMVVTF APVNITTEVK SVEMHHEALS EALPGDNVGF NVKNVSVKDI
321    RRGNVCGDSK SDPPQEAAQF TSQVIILNHP GQISAGYSPV IDCHTAHIAC KFAELKEKID RRSGKKLEDN PKSLKSGDAA
401    IVEMIPGKPM CVESFSQYPP LGRFAVRDMR QTVAVGVIKN VEKKSGGAGK VTKSAQKAQK AGK
```

5.212 troponin C, slow [Gallus gallus]

Protein Accession	gi|45384092
Mean Expression Ratio	1.05
Median Expression Ratio	1.05
Credible Interval	(0.84, 1.31)
Associated Peptides	2
Associated Spectra	2
Coverage	0.205

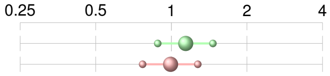

A	2.5	50	97.5	Sequence
**1**	**0.88**	**1.1**	**1.5**	**AAFDIFVLGAEDGCISTK**
**1**	**0.77**	**0.99**	**1.3**	**AAVEQLTEEQKNEFK**

```
1      MDDIYKAAVE QLTEEQKNEF KAAFDIFVLG AEDGCISTKE LGKVMRMLGQ NPTPEELQEM IDEVDEDGSG TVDFDEFLVM
81     MVRCMKDDSK GKTEEELSDL FRMFDKNADG YIDLEELKIM LQATGETITE DDIEELMKDG DKNNDGRIDY DEFLEFMKGV
161    E
```

5.213 bleomycin hydrolase [Gallus gallus]

Protein Accession	gi|45384138
Mean Expression Ratio	0.956
Median Expression Ratio	0.955
Credible Interval	(0.739, 1.24)
Associated Peptides	1
Associated Spectra	1
Coverage	0.0286

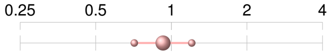

A	2.5	50	97.5	Sequence
**1**	**0.71**	**0.93**	**1.2**	**KYFPESHTTEATR**

```
1      MNAHGLSTEK AAAFTRRLRA EPQFLLAQNV ATCSDPLEVC LQRQVVQDTI QVFQHAVPAE GKPVTNQKNS GRCWIFSCLN
81     AMRLPFMKKY NIEEFEFSQS YLFFWDKVER CYYFLNAFVE TAQKKEPIDG RLVQFLLTNP TNDGGQWDML VNIVEKYGVV
161    PKKYFPESHT TEATRRMNEI LNHKMREYCL RLRNMVATGT NKEELCAAMD TMIEEVFRIV STCLGNPPET FCWEFRDKEK
241    NYHKFGPMTP VQFYNEHVKP YFNMEDKVCL VNDPRPQNPY CQLYTVEYLG NMAGGRKTLY NNQPIEVLKK LAATSIKDGE
321    AVWFGCDVAK HFYSKLGIND LNIFNHELVF GVSVKNMNKA ERLIFGDSLM THAMVLTAVS EKDGQEDCYE KWRVENSWGE
401    DRGNKGYLIM TDDWFSEYVY EVVVDKKYVP EDVLAVMEQE PIVLPAWDPM GALAK
```

5.214 non-metastatic cells 2, protein (NM23B) expressed in [Gallus gallus]

Protein Accession	gi|45384260
Mean Expression Ratio	1.05
Median Expression Ratio	1.05
Credible Interval	(0.81, 1.34)
Associated Peptides	1
Associated Spectra	1
Coverage	0.0915

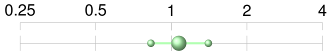

A	2.5	50	97.5	Sequence
**1**	**0.83**	**1.1**	**1.4**	**NIIHGSDSVESAQK**

```
1      MAANCERTFI AIKPDGVQRG LVGEIIKRFE QKGFRLVAMK FVHASEDLLK QHYIDLKDRP FYPGLVKYMN SGPVVAMVWE
81     GLNVVKTGRV MLGETNPADS KPGTIRGDFC IQVGRNIIHG SDSVESAQKE ISLWFKPAEL IDYRSCAHDW VYE
```

5.215 cytochrome c, somatic [Gallus gallus]

Protein Accession	gi|118405198
Mean Expression Ratio	1.05
Median Expression Ratio	1.05
Credible Interval	(0.842, 1.31)
Associated Peptides	2
Associated Spectra	2
Coverage	0.248

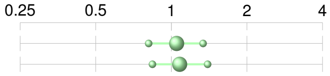

A	2.5	50	97.5	Sequence
**1**	**0.81**	**1.0**	**1.3**	**KTGQAEGFSYTDANK**
**1**	**0.84**	**1.1**	**1.4**	**TGPNLHGLFGR**

```
1      MGDIEKGKKI FVQKCSQCHT VEKGGKHKTG PNLHGLFGRK TGQAEGFSYT DANKNKGITW GEDTLMEYLE NPKKYIPGTK
81     MIFAGIKKKS ERVDLIAYLK DATSK
```

5.216 connexin 45 [Gallus gallus]

Protein Accession	gi|45382839
Mean Expression Ratio	1.05
Median Expression Ratio	1.05
Credible Interval	(0.822, 1.32)
Associated Peptides	1
Associated Spectra	2
Coverage	0.0228

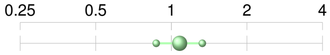

A	2.5	50	97.5	Sequence
**2**	**0.87**	**1.1**	**1.3**	**MVEHSDVDR**

```
1      MSWSFLTRLL EEIHNHSTFV GKIWLSVLIV FRIVLTAVGG ESIYYDEQSK FVCNTEQPGC ENVCYDAFAP LSHVRFWVFQ
81     IILVATPSVM YLGYAIHKIA RMVEHSDVDR RFRSKSFSTR WKQHRGLEEA EDDHEEDPMM YPEIELESER ENKEQQPPAK
161    AKHDGRRRIR EDGLMRIYVL QLLVRATFEV GFLIGQYLLY GFEVSPVFVC SRKPCPHKID CFISRPTEKT IFLLIMYGVS
241    CMCLLLNVWE MLHLGFGTIR DTLNNKRKEL EDSGTYNYPF TWNTPSAPPG YNIAVKPDQM QYTELSNAKM AYKQNKANIA
321    QEQQYGSNEE NIPADLENLQ REIKVAQERL DMAIQAYNNQ NNPGSSSREK KSKAGSNKSS ASSKSGDGKN SVWI
```

5.217 calreticulin [Gallus gallus]

Protein Accession	gi|44969651
Mean Expression Ratio	1.05
Median Expression Ratio	1.05
Credible Interval	(0.852, 1.28)
Associated Peptides	2
Associated Spectra	4
Coverage	0.047

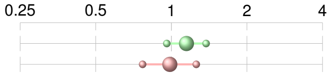

A	2.5	50	97.5	Sequence
**3**	**0.96**	**1.1**	**1.4**	**QKQEEEDKQR**
**1**	**0.77**	**0.99**	**1.3**	**KVHVIFNYK**

```
1      MSRLCLPLLL GAVLAVTAAG PAQFFREEFL DGDSWTQRWV ESKHKSDYGR FVLTAGKFYG DAEKDKGIQT SQDARFYALS
81     SRFEPFSNRD KTLVVQFTVK HEQNIDCGGG YVKLFPASLN QEDMHGDSEY NIMFGPDICG PGTKKVHVIF NYKGKNVLIN
161    KDIRCKDDEF THLYTLIVRP DNTYEVKIDN SKVESGSLED DWDFLPPKKI KDPEAKKPDD WDERAKIDDP EDSKPEDWDK
241    PEHIPDPDAK KPEDWDEEMD GEWEPPVIQN PEYKGEWRPR QIDNPDYKGK WVHPEIDNPE YTPDPNLYAY DSFGVIGLDL
321    WQVKSGTIFD NFLITDDEKF AEEFGNETWG ATKEAERKMK EQQDEEQRQK QEEEDKQRKE EEGDEDGDGD DEEDEEDEED
401    EAEK
```

5.218 AK [Gallus gallus]

Protein Accession	gi|46048771
Mean Expression Ratio	1.05
Median Expression Ratio	1.05
Credible Interval	(0.858, 1.28)
Associated Peptides	3
Associated Spectra	3
Coverage	0.17

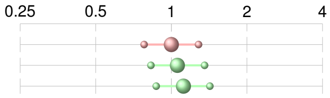

A	2.5	50	97.5	Sequence
1	0.78	1	1.3	GELVPLDTVLDMLR
1	0.83	1.1	1.4	GFLIDGYPR
1	0.87	1.1	1.4	VDDNEETIKK

```
1       MSTEKLKHHK IIFVVGGPGS GKGTQCEKIV HKYGYTHLST GDLLRAEVSS GSERGKKLQA IMEKGELVPL DTVLDMLRDA
81      MLAKADTSKG FLIDGYPREV KQGEEFEKKI APPTLLLYVD AGKETMVKRL LKRGETSGRV DDNEETIKKR LETYYKATEP
161     VIAFYKGRGI VRQLNAEGTV DEVFQQVCSY LDKL
```

5.219 suppressor of Ty 5 homolog [Gallus gallus]

Protein Accession	gi|71897283
Mean Expression Ratio	0.956
Median Expression Ratio	0.957
Credible Interval	(0.774, 1.18)
Associated Peptides	2
Associated Spectra	3
Coverage	0.0269

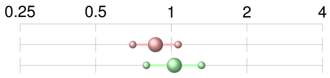

A	2.5	50	97.5	Sequence
2	0.7	0.86	1.1	TPAQSGAWDPNNPNTPSR
1	0.8	1.0	1.3	HLVLAGGSKPR

```
1       MSDSDDSNFS EEESEHSSEA EEAEEAEAEE ERASAAGSEK EEVEEEEEEE YDEEEEEEDD DRPAKKPRHG GFILDEADVD
81      DEYEDEDQWE DGAEDILEKE EIEASNIDNV VLDEDRSGAR RLQNLWRDQR EEELGEYYMK KYAKSSVGET VYGGSDELSD
161     DITQQQLLPG VKDPNLWTVK CKIGEERATA IALMRKFIAY QFTDTPLQIK SVVAPEHVKG YIYVEAYKQT HVKQAIEGVG
241     NLRMGYWNQQ MVPIKEMTDV LKVVKEVTNL KPKSWVRLKR GIYKDDIAQV DYVEPSQNQI SLKMIPRIDF DRIKARMSLK
321     DWFAKRKKFK RPPQRLFDAE KIRSLGGDVA SDGDFLIFEG NRYSRKGFLF KSFAMSAVIT EGVKPTLSEL EKFEDQPEGI
401     DLEVVTESTG KEREHNFQPG DNVEVCEGEL INLQGKILSV DGNKITIMPK HEDLKDMLEF PAQELRKYFK MGDHVKVIAG
481     RFEGDTGLIV RVEENFVILF SDLTMHELKV LPRDLQLCSE TASGVDVGGQ HEWGELVQLD PQTVGVIVRL ERETFQVLNM
561     YGKVVTVRHQ AVTRKKDNRF AVALDSEQNN IHVKDIVKVI DGPHSGREGE IRHLFRGFAF LHCKKLVENG GMFVCKTRHL
641     VLAGGSKPRD VTNFTVGSFA PMSPRISSPM HPSGAGQRGG FGGGGMSRGR GRRDNDLIGQ TVRISQGPYK GYIGVVKDAT
721     ESTARVELHS TCQTISVDRQ RLTTVGSRRP GGMTSTYGRT PMYGSQTPMY GSGSRTPMYG SQTPLHDGSR TPHYGSQTPL
801     HDGSRTPAQS GAWDPNNPNT PSRADEDFEY GFDDEPTPSP QGYGGTPNPQ TPGYPDPSSP QVTQPYNPQT PGTPAMYNTD
881     QFSPYAVPSP QGSYQPSPSP QSYHQVAPSP VGYQNTHSPA SYHPTPSPMA YQASPSPSPV GYSPMTPGAP SPGGYNPHTP
961     GSGIEQSSSD WVTTDIQVKV RDTYLDSQAV GQTGVIRSVT GGMCSVYLKD SEKVVSISSE HLEPVTPTKS NKVKVILGED
1041    REATGILLSI DGEDGIVRMD LDEQLKILNL RFLGKLLEA
```

5.220 profilin 2 [Gallus gallus]

Protein Accession	gi|119331154
Mean Expression Ratio	1.05
Median Expression Ratio	1.04
Credible Interval	(0.808, 1.35)
Associated Peptides	1
Associated Spectra	1
Coverage	0.0786

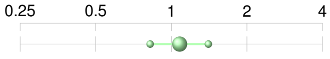

A	2.5	50	97.5	Sequence
1	0.82	1.1	1.4	EGVHGGGLNKK

```
1       MAGWQSYVDN LMCDGCCQEA AIVGYCDAKY VWAATAGGIF QSITPVEIDM IVGKDREGFF TNGLTLGAKK CSVIRDSLYV
81      DGDCTMDIRT KSQGGEPTYN VAVGRAGRVL VFVMGKEGVH GGGLNKKAYS MAKYLRDSGF
```

5.221 PREDICTED: hypothetical protein, partial [Gallus gallus]

Protein Accession	gi|118123643
Mean Expression Ratio	1.04
Median Expression Ratio	1.04
Credible Interval	(0.805, 1.34)
Associated Peptides	1
Associated Spectra	1
Coverage	0.222

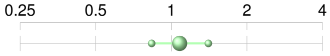

A	2.5	50	97.5	Sequence
**1**	**0.83**	**1.1**	**1.4**	**SYLEFVEDFIQVPR**

```
1       TADAVKKARS YLEFVEDFIQ VPRNLVGKVI GKNGKVIQEI VDKSGVVRVR IEGDNENKLP RED
```

5.222 p32 subunit of splicing factor SF2 [Gallus gallus]

Protein Accession	gi|5509946
Mean Expression Ratio	1.04
Median Expression Ratio	1.04
Credible Interval	(0.807, 1.35)
Associated Peptides	1
Associated Spectra	1
Coverage	0.101

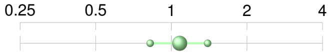

A	2.5	50	97.5	Sequence
**1**	**0.82**	**1.1**	**1.4**	**IHTEGDKAFAQFLTDEIKEER**

```
1       IHTEGDKAFA QFLTDEIKEE RKIQKHKALP KVSGGWELEV HGTEARLVRK VAGEKITVTF NINNSIPPAV DDEAPEEQKP
81      DEQEEPDLTS TPNFVVEVIK DDTKQTLVLD CHYPQDEVGH QGEEESDIFT IREVSFQPTG ESDWKDTNYT LNTDSLDWAL
161     YDHLMDFLAD RGVDNTFADE LIELSTALEH QEYIKFLEDL KSFVKCQ
```

5.223 PREDICTED: hypothetical protein [Gallus gallus]

Protein Accession	gi|118101322
Mean Expression Ratio	0.957
Median Expression Ratio	0.957
Credible Interval	(0.737, 1.23)
Associated Peptides	1
Associated Spectra	1
Coverage	0.0213

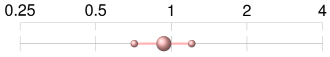

A	2.5	50	97.5	Sequence
**1**	**0.71**	**0.93**	**1.2**	**EEPTQQQTPLEAR**

```
1       MDGVFLSPNE DEFVGRIAEE LEHFMLQGQH HRVLLFPPLS SRLRYLIHRT VENVDLLSSF SVGEGWRRRT VICHSAVRIP
81      SETSEQNPAT TAPRPPRPTQ PWGRGARGAR LRHGGDGHGD TSRACVGSGR IKRPPRRKPD KALYVPKAMH KKVEWGEQEV
161     GADGAREGEI CPKAALEGEA EGGPGPNGAI GGVCLVLDEQ HKDGEESGKS SHGTNGDRPP RCTDVPPLES SNSSSGQHSQ
241     DKDCSDSLSS VPNENPAEPE EQSKSCDDTV GPEGSKTPCQ DQKSRDADSK SSHLLGEHCS DATLVLENPK KSCAMSLESS
321     GNVSVQGEDV VSPVLELGEG PSAPESEDQE SFSSAREEPT QQQTPLEARG EAVLAPEPIG GAGPSGRSEQ EEHREDVPVA
401     KDGVREPMGL ADALQRELRL PKGDQEENAA TQSRELAFED DCTAELLQEI MGHLTVKDIS VERITLDYSS YGDAQVHEGD
481     FGHVTEIYDF SPSLKTEHLM EVFSDFHESG FKIQWVDDTH ALGIFSSLSA ASQALGRRYP SLKIRPLIHA TKQSKIKALQ
561     RPKLLQLAKE RPQTDTVVAR RLVTRALGLK HQQQCGSGPE GLLPEGLDQE E
```

5.224 PREDICTED: similar to fat3 [Gallus gallus]

Protein Accession	gi|118085157
Mean Expression Ratio	1.05
Median Expression Ratio	1.04
Credible Interval	(0.837, 1.31)
Associated Peptides	2
Associated Spectra	2
Coverage	0.00439

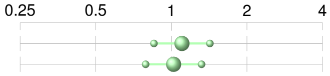

A	2.5	50	97.5	Sequence
**1**	**0.85**	**1.1**	**1.4**	**QQFYNLTVR**
**1**	**0.8**	**1.0**	**1.3**	**DADIGSNGDIR**

```
1       MEMNMGPWSG TRPPPPCLAL LLAELLASLF QVAHGATPLG FHFTHSTYNA TVYENSAART YVNSQTRMGI TLSDLSWDIK
81      YRIVSGDDEG FFKAEEVVIA DFCFLRIRTK GGNSAILNRE IQDNYLLVVK GSVRGEDLEA WTKVNIQVLD MNDLRPLFSP
161     TTYSVTIAES TPLRTSIAQV TATDADIGSN GEFYYYFKSK VDLFSVHPTS GVISLSGRLN YDEKNRYDLE ILAVDRGMKL
241     YGNNGVSSTA KLYVHIERIN EHAPTISVVT HIPFPSDKEP TYAVVNIDDL DEGANGEIES VSIVAGDPLE QFFLIKEGKW
321     MNEYKIKERK PIDWDSFPYG YNLTLQAKDK GSPQKFSAVK LVHIASPKKE SIPLKFEKEA YDVSVSEFSP PGVVVAVVKL
401     MPEPLGVEYR ISPSEGAENF KINPITGLIT TARSLKIIEK DLYELEVYTN KGDDLKAQVT IRLEDANDHT PEFQQTSYSS
481     FVNESVPVGF SVLAVSAVDK DRGENGYITY SIASLNSLPF IINQFTGVIS TSEELDFESS PESYRFIVRA SDWGSPYRHE
561     SEVNVTIYIG NVNDNKPLFE KVACQGVISS DFPVGGHITA VSAIDIDELE LVKYKIISGN ELGIFYLNPD SGVLQLKKSL
641     INSGIKNSNF GLKITATDGE NFADAMFVNI SVVHGKVSSK TFSCRETRVA QRLAEKLLKK AKANVKLNLE DGFLDYYSVN
721     RQAPHFDKTF PTDVMVSEDL PVGATILRIK AYDADSGFNG KVVYTISDGN ADSCFNIDIE TGVLKVLMPL DREKTDLYIL
801     NITIYDLGNP QKSAWRLLTV TVGDANDNKP IFLQDSYSVN ILESTSLGTE IIQVEARDKD LGSNGEVTYS VLTDTQQFAI
881     NSSTGVVYVA DQLDRETKAN YTLKIEARDK AESGHQQFSV VPLKVFLDDV NDCSPAFIPS SYNVKVLEDL PVGTVIAWLE
961     THDPDLGLGG QVRYSLVNDY NGRFEIDKAS GAIRLSKELD YEKQQFYNLT VRAKDKGRPV SLSSVSFVEV EVVDVNENLY
1041    TPYFPDFAVI GSVKENSRIG TSVLQVVARD EDSGRDGEIQ YSIRDGSGLG RFSIDEETGV IYTADVLDRE TTKSYWLTVY
1121    ATDHGVVPLY ATIEVYIEVE DVNDNAPLTS EPIYYPSVME NSPKDVSVIQ IQAQDPDSST NEKLAYRITS GNPQNFFVIN
1201    PKTGLITTTS RKLDREQQAE HFLEVTVSDG GTSPKQSSVW VVVQVLDEND NKPQFPEKVY QIKLPERDRK KRGEPIYRAF
1281    AFDKDEGPNA EISYSIVDGN DDGKFFIDPK TGMVSSRKQF TAGSYDILTI KAVDNGRPQK SSTARLHIEW IKKPTPSPVP
1361    LTFDEPFYNF TVMESDKVTE IVGVVSVQPS NIPLWFDIVG GNYDSSFDAE KGVGTIVIAK PLDAEQRSIY NMTVEVTDGT
1441    NVATTQVFIK VLDNNDNGPE FSQPSYDVTI SEDILPDTEI LQIEAIDRDE KHKLSYTIHS SIDAISMRKF RIDPSAGVLY
1521    TAERLDHEAQ DKHILNVMVR DQEFPYRRNL ARVIINVEDS NDHSPYFTSP LYEASVYESA AVGSAVLQVT ALDKDKGENA
1601    ELVYTIEAGN TGNTFKIEPV LGIITLLKEP DLTTMGQFVL SVKVADQGSP PLSATAIVRI SVTMADNSHP KFTLKEYQAE
1681    VNENVDIGTS VISLSAISQS TLVYEIKDGN VDGLFTINPY SGVITSQKAL DYEHASFYQL TVQATNMAGM ASNATVNIQI
1761    VDENDNPPVF LFSQYSGSIS EAAPINSIVR STNNSPLVIR ATDADSNQNA LLVYQIVEST AKKYFTVDSS TGAIRTIANL
1841    DHETIAHFHF HVHVRDSGNP QLTAESPVEV TIEVTDVNDN PPVFSQAVFE TVLLLPTYVG VEVLKVKATD PDSEIPPELT
1921    YSLIEGNMDH FLIDSNTGVL TIKNNSLSKD HYMLIVRVSD GKFYSTAMVT IMVKEAMDSG LHFTQNFYST SISENTTNIT
2001    KIAVVNAVGN RLNEPLKYSI LNPGNKFKIK STSGVIQTTG ISFDREEQEL YELVVEASRE LDHLRVARVV VRVYIEDIND
2081    NAPVFVGLPY YAAVQVDAEP GTLIYRVTAI DKDKDENGDV SYLLKEDYGH FEIDRGTGSV TLKEAFNSDL SNIEYLVVIL
2161    AKDGGNPSLS ASVELPITIV NKAMPVFDKP FYTASVNEDV EIHTPILSIN ATSPEGQGII YIIVEGDPHN QFNIDFDTGV
2241    LSVISPLDYE INSLFKLMVR ASDALTGARA EVTVDLVIND VNDNPPVFDQ SAYNATLSEA SLIGTPVLQV VATDADSDNN
2321    KIVQYQIVQD TFNSTDYFHI DSTSGLILTA RMLDHELIQQ CSLKVRATDN GFPPLSSEVL VSISITDMND NPPVFNQLIY
2401    ESYVSELAPR GHFVTCVQAS DADSSDFDRL EYSILSGNDR TSFIMDSKSG VITLSNHRKQ RMEPMYSLNV SVSDGLFTST
2481    AQVHIQILGA NLYSPVFSQS VYVAEVRENA APGTKVLHVK ATDGDSGVYG QISYSIINDF AKDRFLIDSN GQILTTERLD
2561    RENLLESDIG IFLRALDGGG RTTFCTVRVI VVDENDNAPQ FMTVEYRASV KADVGKGYLV TQVQAVDPDD GANSRITYSL
2641    YSEASVSVAD LLEIDPDNGW MVTKGSFNQL KNTVLSFFVK AVDGGIPVKH SLIPVYIHVL PPETILPSFS QPQYSFTLAE
2721    DTLIGSTIDI VHIVPNQGAL YSTVNGELTE NNKDGIFIIE QDTGLIKLDK RLDHETNSAF HFKVAATIQL DKVDIVLTVD
2801    VEVKVLDVND NKPMFETASY DAIIMEGMPI GTKLMQVKAV DADSSANGQV TYTLAVESEL EKITEAFTID SNSGWISTLK
2881    DLDHEKDPTF TFAVVASDLG ETLSLSSTTL VSVAVTDIND NAPVFEHEVY RGSVKESDPP GEVVAVLSTW DEDTSDVNRQ
2961    VSYHITGGNP KGKFALGLVQ NEWKVYVKRP LDREEQDVYY LNITATDGLF VTQAAVEVTV TDVNDNSPVC EQVAYTALFP
3041    EDIPSNKVIL KISAKDADIG SNGDIRYSLY GPGNNKFFLD PENGELKSLA PLDREKIPVY NLVARATDGG GRFCQSEIHL
3121    ILEDVNDNPP VFSSDHYTAC VYENTATKAL LTRVQATDPD VGVNRKVTYS LADSADGYFS VDRSSGIIIL EHPLDRELQS
3201    SYNISVKASD QSIVLTLSSF ATVTITVLDI NDNPPVFERR DYLVTVPEDT SPGTEVLSVF ATSQDIGTNA EITYLIRSGN
3281    EKGKFNINSK TGAISISESL DYESCKDFYL VVEAKDGGTP ALSAVTTVNV NVTDVNDNAP KFSQVVYSAV ISEDAAIGDS
3361    VIMLIAEDLD SPSNGQIHFS IVSGDQDNEF SVDPGSGLVK VKKKLDRERI SGYSLVIQAR DSGTPPLSSS VTVNVDISDV
3441    NDNSPVFTPA NYTAVIQENK PVGTSILQLV VTDKDSFHNG PPFTFTILTG NEEEEFTLDP NGVLRSAVIF KHMVATEYVL
3521    CVQAKDSGKP QQVSHTYIQV RVIEESIHKP TAIPLEIFIV TMEDDFPGGV IGKIHATDQD VYDVLTYTLK SEQKSLFKVN
3601    NHDGKIIALG GLDNGKYILN VSVSDGRFQV PIDVVVQVEQ LLQEMLQNTV TIRFDNVSPE DFVGLHMHGF RRTLRNAVLS
3681    QKQDSLHIIS IQPVAGSNQL DMLFAVQMHS GGFYKPAFLI QKLTNARRHL ENVIRISAIL EKNCSGLDCQ EQHCEQSLSI
3761    DSHSLMTYST ARISFVCPRF YRNVRCMCNG GLCPGSNDPC LEKPCPGDMQ CVGYEANRRP FICQCPPGKL GECSGHTSLS
3841    FAGNSYIKYR VSENSKKEEF KLALRLRTLQ SNGIIMYTRA NPCIILKIVD GKLWFQLDCG TGPGILGISG RAVNDGSWHS
3921    VFLELNRNFT SLSLDDSYVE RRKAPLYFQT LSTDSSVYFG AQVQVDNVRS LTDKRTTQVL SGFQGCLDSV VLNNNELPLQ
4001    NKRSSFAEVV GLTELKLGCV LYPDACERHP CQNGGTCTSV PSGGYQCNCL SQFTGRNCES EITACFPNPC RNGGSCDPIG
4081    NAFICNCKNG LTGVTCEEDI NECEREECEN GGSCVNVFGS FLCNCTPGYV GQYCGLRPVV VPNIQAGHSY VGKEELIGIA
4161    VVLFVIFVLI VLFIIFRKKV FRKNYSRNNI TLVQDPATAA LLNKSNGIQF KNIRNSGDSR NIYQEVGPPQ VPVRPMAYTP
4241    CFQSDSRNNL DKIVDGLGVE HQEMTTFHPE SPRILTARRG VVVCSVAPNL PAVSPCRSDC DSIRKSTWDA GTESKGVDDA
4321    EEVTCFAGSN KGSNSEVQSL SSFQSDSGDD NAYHWDTSDW MPSARLSDIE EVPNFEAADG GSAHHGSTRE LETDYYLGGY
4401    DIDSDYPPPH EEEFLSQDQL PPPLPEDYPD QYETLPPSQP VSMANTLSPD CRRRPHFHPS QYLPPHQFPH ETDTTGAQTG
4481    NEFSTFAVGP SQNTENVSAG KMPLTLHNSL DASSSDVSAS CGFDDSEVAM SDFESVEELK LENVHIPFVE TQHQTQV
```

5.225 PREDICTED: laminin, gamma 1 (formerly LAMB2) [Gallus gallus]

Protein Accession	gi|118094226
Mean Expression Ratio	0.958
Median Expression Ratio	0.958
Credible Interval	(0.767, 1.19)
Associated Peptides	2
Associated Spectra	2
Coverage	0.0193

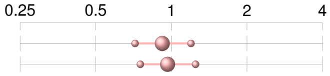

A	2.5	50	97.5	Sequence
1	0.72	0.92	1.2	AATKXRGSASASPTSPR
1	0.75	0.97	1.3	ISISGXTTTRQQLR

```
1       MGGVGGRRAL LRVLALLAVL ALPRGGGAAM DECTEERSGR PQRCMPEFVN AAFNAHGGGH QHVRHAGRGV LRADRRHRRN
81      PSRATCATPP SPTCGTAPPS SPTTTDRPTA PGGRARACWP ACSTPPPSTS PCTSERPLTS PTCGXSSTPA GQRASPSTNA
161     RGRMARGCPT SITAGRVRAP TTKSTGALFG LGRTSSRRCA RMSSVTSPPS LGATWLSQRW RGVPAPTTLT TAPCCRNGXR
241     PPILGXASTA STPSEMKFSM TPKSSSRITT RFLILPWVED ANAMAMRASA XGTSLGSWSA TASTTPLGMT VRSAFPSSMT
321     GRGGEQQQKV PTSACLVTAM GGHRSATSTP SCTAPPATAG TAWAAGTTPT APTARGAGTA STASGARRAA CPAAATPWAL
401     SAHSVIATAS AAASLVSWGR NVTAASQAST LSLRLGAGPV LAILLAAQVN ATWKQDGVLA RTTLRASTVR DANLGFSIWI
481     PQIQEAVPPA SVLDTLRFAP MPLATACTAS PPAFSLERTS GTLSNVTAPR SLCSGVQRPR TSLSSQTATS PSILLHHANS
561     WATKFXATVR TXPSPSVWTD GTHVSQQRTW YWKGPGCVCR CLSLPRATRT PVRAHXPTPS GSMRLLITRG GLLSLHLTSR
641     SFSTIXLPSR SVEHTAREVP AIWMTLPSPV LSQGPACPQR GWRAAPVQQA TRGSSVSAAP LGTAEKPPAW GPTAPACLAP
721     AMGTARPVIL RRACATAGIT RQALTARSAA MGTTGMRQRA QPWTASPAPA PAAPAVPSCP AQRRLCAPAA RQAPPARGVS
801     CVMMPILETH WVKMGLXGRA ACASATTTLI PTLWATATGR RGSASSASTT QPASTATAAR TASLGTPWPP TLLTSAGLVT
881     VIPTAPXISR RAATKXRGSA SASPTSPRGT AAPASPASST CRAAEAVRGV TATRWAPPMA NVTYGQGSVS ASPASPASAA
961     TAARPTTLDS ALRAANHATV TQRAHVPCSA VRMGAVSARR ALWGAAATSV RRTTSTTGRG RAARSALRVT DXXKIRWQSN
1041    ERGYGSWKTX XQIWAQEKKL SLIKPSRRGX SRQKEMLWSC SEKHRTAKTX IRVSWIVSET STALXQVSSA GXGTSRTRCR
1121    RQRTWQSKPG AGWRTQRTXL QWLLICWRRQ RWQLATCPLH PRSRAVTPTT XLYWQKRRAG WLRGTNKKLM RSFALPRQPT
1201    ILPQKRISCC XRPWLGKTKQ HVTLMSSTRN ITKQETFLEI XRDRPTVCLR RQRKLEIRPS RSMLISPICP LWTPRGWRMK
1281    QIRSRRKQRS XISXXQGSXR TMKTXGKTXR ERSWKSRTSW RKGRRSSRLQ TSSWLEQMQQ KPWLRKLLGR AMAHCRRPTP
1361    FSATXKISIS GXTTTRQQLR RRXRRSLPLP RPLLKPTTRP ARQSWRLATL LLMPVKPKLE QMMLRKLPAL FRRVPLLPEQ
1441    KRTRPSLTXR GWPERWMTXX SSSRRLKKSX SVNKMMLIKI XXWQAWPHRQ PRRQKTTQEK RKTLXTACLL SLMTFXTNXG
1521    NWRRWTXTNX MRLRVPXTVP KTRXKIATWT RRXLSLREKP GSKMMRYSPT TGTLRKSXKT LATWRTSGRP CHLAALTPRP
1601    LRNP
```

5.226 DnaJ (Hsp40) homolog, subfamily A, member 2 [Gallus gallus]

Protein Accession	gi|57524857
Mean Expression Ratio	1.04
Median Expression Ratio	1.04
Credible Interval	(0.826, 1.32)
Associated Peptides	1
Associated Spectra	2
Coverage	0.0365

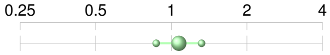

A	2.5	50	97.5	Sequence
2	0.87	1.1	1.3	EYHPDKNPNAGDKFK

```
1       MANVADTKLY DILGVPPGAS DNELKKAYRK LAKEYHPDKN PNAGDKFKEI SFAYEVLSNP EKRELYDRYG EQGLREGSGG
81      SSGMDDIFSH IFGGGLFNFM GGQSRSRNGR RRGEDMMHPL KVSLEDLYNG KTTKLQLSKN VLCSACNGQG GKAGAVQKCN
161     ACRGRGVRIM IRQLAPGMVQ QMQSVCSDCN GEGEVINEKD RCKKCEGKKV IKEVKILEVH VDKGMKHGQR ITFSGEADQA
241     PGVEPGDIVL LLQEKENEVF QRDGNDLHMT HKIGLVEALC GFQFTFKHLD GRQIVVKYPP GKVIEPGCVR VVRGEGMPQY
321     RNPFEKGDLY IKFDVQFPEN NWISPEKLSE LEDLLPARPE FPNVIGDAEE VDLQEFDTTR GSGGGQRREA YNDSSDEESS
401     HHGPGVQCAH Q
```

5.227 PREDICTED: similar to putative pre-mRNA splicing regulator female-lethal(2D) [Gallus gallus]

Protein Accession	gi|50741755
Mean Expression Ratio	0.96
Median Expression Ratio	0.959
Credible Interval	(0.746, 1.24)
Associated Peptides	1
Associated Spectra	1
Coverage	0.0379

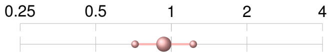

A	2.5	50	97.5	Sequence
1	0.71	0.93	1.2	TPSSEPTDQGEAVGK

```
1       MTNEEPLPKK VRLSEADFKV LPRDELILRW KQYEAYVQAL EGKYTDLNSN DVTGLRESEE KLKQQQQESA RRENILVMRL
81      ATKEQEMQEC TNQIQYLKQV QQPSVAQLRS TMVDPAINLF FLKMKGELEQ TKDKLEQAQN ELSAWKFTPD SQTGKKLMAK
161     CRMLIQENQE LGRQLSQGRI AQLEAELALQ KKYSEELKSS QDELNDFIIQ LDEEVEGMQS TILVLQQQLK ETRQQLAQYQ
241     QQQSQASNPG TSRTPSSEPT DQGEAVGKDC SRLANGPSNG SSSHQRTSGP GFYREGSSTE DDFPASPGNG NKLSNHSEDR
321     TGRGGGSYIN QLSTGYESVD SPTGSENSLT HHSNDTDSNH DPQEEKTVSM KGNRTAGSRH VQNGLDSNVN VQGSVL
```

5.228 PREDICTED: hypothetical protein [Gallus gallus]

Protein Accession	gi|50748470
Mean Expression Ratio	0.96
Median Expression Ratio	0.959
Credible Interval	(0.745, 1.24)
Associated Peptides	1
Associated Spectra	1
Coverage	0.0242

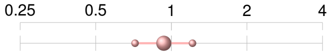

A	2.5	50	97.5	Sequence
1	0.72	0.94	1.2	GVEEGASLLLDGR

```
1       MAAAAAARGV WGGRRRVALR LPRRAGVPWI ASAAASSSSV PTTKLFIDGK FVESKTTEWI DIHNPATNEV VARVPKATTS
81      EMEAAVASCK KAFWNWSETS VLSRQQIFLR YQQLIKDNLK EISKLITFEQ GKTLADAEGD VFRGLQVVEH ACSVTSLILG
161     ETMPSITKDM DTCTYRLPLG VCAGIAPFNF PAMIPLWMFP MAMVCGNTFL MKPSERVPGA LMFLAKLFQD AGAPDGTLNI
241     IHGQHEAVNF ICDHPDIRAI SFVGSNQAGE YIYERGSRNG KRVQANMGAK NHGVVMPDAN KENTLNQLVG AAFGAAGQRC
321     MALSTAILVG EAQKWLPELV ERAKNLRVNA GDQPGADLGP LISPQAKERV CHLIQKGVEE GASLLLDGRN IKVKGYENGN
401     FVGPTILANV KPNMTCYKEE IFGPVLVVLE ADTLDDAIEV VNNNPYGNGT AIFTTNGATA RKYSHLVDVG QVGVNVPIPV
481     PLPMFSFTGS RASFRGDANF YGKQGVQFYT QLKTIISQWK EEDATVTKPA VVMPTMGN
```

5.229 clathrin heavy chain 1 [Gallus gallus]

Protein Accession	gi|124339781
Mean Expression Ratio	0.96
Median Expression Ratio	0.96
Credible Interval	(0.803, 1.15)
Associated Peptides	4
Associated Spectra	4
Coverage	0.0424

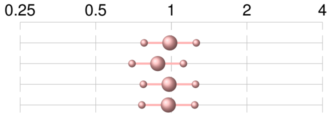

A	2.5	50	97.5	Sequence
1	0.78	0.99	1.3	TSIDAYDNFDNISLAQR
1	0.7	0.88	1.1	SVNESLNNLFIIEEDYQALR
1	0.77	0.98	1.3	KVGYTPDWIFLLR
1	0.76	0.97	1.2	KFDVNTSAVQVLIEHIGNLDR

```
1       MAQILPIRFQ EHLQLQNLGI NPANIGFSTL TMESDKFICI REKVGEQAQV VIIDMNDPSN PIRRPISADS AIMNPASKVI
81      ALKAGKTLQI FNIEMKSKMK AHTMTDDVTF WKWISLNTVA LVTDNAVYHW SMEGESQPVK MFDRHSSLAG CQIINYRTDA
161     KQKWLLLTGI SAQQNRVVGA MQLYSVDRKV SQPIEGHAAS FAQFKMEGNA EESTLFCFAV RGQAGGKLHI IEVGTPPTGN
241     QPFPKKAVDV FFPPEAQNDF PVAMQISDKH DVVFLITKYG YIHLYDLETG TCIYMNRISG ETIFVTAQHE ATAGIIGVNR
321     KGQVLSVCVE EENIIPYITN VLQNPDLALR MAVRNNLAGA EELFARKFNA LFAQGNYSEA AKVAANAPKG ILRTPDTIRR
401     FQSVPAQPGQ TSPLLQYFGI LLDQGQLNKY ESLELCRPVL QQGRKQLLEK WLKEDKLECS EELGDLVKSV DPTLALSVYL
481     RANVPNKVIQ CFAETGQVQK IVLYAKKVGY TPDWIFLLRN VMRISPDQGQ QFAQMLVQDE EPLADITQIV DVFMEYNLIQ
561     QCTAFLLDAL KNNRPSEGPL QTRLLEMNLM HAPQVADAIL GNQMFTHYDR AHIAQLCEKA GLLQRALEHF TDLYDIKRAV
641     VHTHLLNPEW LVNYFGSLSV EDSLECLRAM LSANIRQNLQ ICVQVASKYH EQLSTQSLIE LFESFKSFEG LFYFLGSIVN
721     FSQDPDVHFK YIQAACKTGQ IKEVERICRE SNCYDPERVK NFLKEAKLTD QLPLIIVCDR FDFVHDLVLY LYRNNLQKYI
801     EIYVQKVNPS RLPVVIGGLL DVDCSEDVIK NLILVVRGQF STDELVAEVE KRNRLKLLLP WLEARIHEGC EEPATHNALA
```

```
881     KIYIDSNNNP ERFLRENPYY DSRVVGKYCE KRDPHLACVA YERGQCDLEL INVCNENSLF KSLSRYLVRR KDPELWASVL
961     LESNPYRRPL IDQVVQTALS ETQDPEEVSV TVKAFMTADL PNELIELLEK IVLDNSVFSE HRNLQNLLIL TAIKADRTRV
1041    MEYINRLDNY DAPDIANIAI SNELFEEAFA IFRKFDVNTS AVQVLIEHIG NLDRAYEFAE RCNEPAVWSQ LAKAQLQKGM
1121    VKEAIDSYIK ADDPSSYMEV VQAANASGNW EELVKYLQMA RKKARESYVE TELIFALAKT NRLAELEEFI NGPNNAHIQQ
1201    VGDRCYDEKM YEAAKLLYNN VSNFGRLAST LVHLGEYQAA VDGARKANST RTWKEVCFAC VDGKEFRLAQ MCGLHIVVHA
1281    DELEELINYY QDRGYFEELI TMLEAALGLE RAHMGMFTEL AILYSKFKPQ KMREHLELFW SRVNIPKVLR AAEQAHLWAE
1361    LVFLYDKYEE YDNAIITMMN HPTDAWKEGQ FKDIITKVAN VELYYKAVQF YLEFKPLLLN DLLMVLSPRL DHTRAVTFFT
1441    KVKQLPLVKP YLRSVQNHNN KSVNESLNNL FIIEEDYQAL RTSIDAYDNF DNISLAQRLE KHELIEFRRI AAYLFKGNNR
1521    WKQSVELCKK DRLYKDAMQY ASESKDTELA EELLQWFLQE NKKECFGACL FTCYDLLRPD VVLETAWRHN IMDFAMPYFI
1601    QVMKEYLTKV DKLDASESLR KEEEQATETQ PIVYGQPQLM LTAGPSVAVP PQAPFGYGYT APPYGQPQPG FGYSM
```

5.230 cell division cycle 42 (GTP binding protein, 25kDa) [Gallus gallus]

Protein Accession	gi|45384262
Mean Expression Ratio	1.04
Median Expression Ratio	1.04
Credible Interval	(0.805, 1.34)
Associated Peptides	1
Associated Spectra	1
Coverage	0.0576

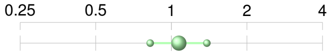

A	2.5	50	97.5	Sequence
1	0.82	1.1	1.4	QKPITPETAEK

```
1       MQTIKCVVVG DGAVGKTCLL ISYTTNKFPS EYVPTVFDNY AVTVMIGGEP YTLGLFDTAG QEDYDRLRPL SYPQTDVFLV
81      CFSVVSPSSF ENVKEKWVPE ITHHCPKTPF LLVGTQIDLR DDPSTIEKLA KNKQKPITPE TAEKLARDLK AVKYVECSAL
161     TQKGLKNVFD EAILAALEPP EPKKTRRCVL L
```

5.231 tubulin, beta 6 [Gallus gallus]

Protein Accession	gi|71896411
Mean Expression Ratio	1.04
Median Expression Ratio	1.04
Credible Interval	(0.81, 1.35)
Associated Peptides	1
Associated Spectra	1
Coverage	0.0314

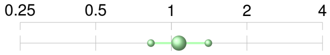

A	2.5	50	97.5	Sequence
1	0.83	1.1	1.4	LHFFMPGFAPLTAR

```
1       MREIVHIQAG QCGNQIGTKF WEVISDEHGI DPAGGYVGDS ALQLERINVY YNESSSQKYV PRAVLVDLEP GTMDSVRSGP
81      FGQLFRPDNF IFGQTGAGNN WAKGHYTEGA ELVDSVLDVV RKECEHCDCL QGFQLTHSLG GGTGSGMGTL LISKIREEYP
161     DRIMNTFSVM PSPKVSDTVV EPYNATLSVH QLVENTDETY CIDNEALYDI CFRTLKLTTP TYGDLNHLVS ATMSGVTTSL
241     RFPGQLNADL RKLAVNMVPF PRLHFFMPGF APLTARGSQQ YRALTVPELT QQMFDAKNMM AACDPRHGRY LTVATVFRGP
321     MSMKEVDEQM LAIQNKNSSY FVEWIPNNVK VAVCDIPPRG LKMASTFIGN STAIQELFKR ISEQFSAMFR RKAFLHWFTG
401     EGMDEMEFTE AESNMNDLVS EYQQYQEATA NDGEEAFEDD EEEINE
```

5.232 hypothetical protein LOC420709 [Gallus gallus]

Protein Accession	gi|57530763
Mean Expression Ratio	1.04
Median Expression Ratio	1.04
Credible Interval	(0.813, 1.35)
Associated Peptides	1
Associated Spectra	1
Coverage	0.088

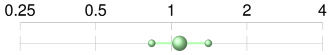

A	2.5	50	97.5	Sequence
1	0.83	1.1	1.4	SSEQDGEPESADGK

```
1       MSKRNQVSYV RPAEPAFLSR FKRQVGYREG PTVETKREQL PLADSDSENG SDNEDEQPQV VTLKKGDLTA EEAMKIKQQI
81      KEALKSSEQD GEPESADGKI MFRKPAKRSS EKVLDFNVSS SKKMKKTKKT DREATSSQST AKQVKNSSLL SFDDEENDD
```

5.233 fatty acid binding protein 7, brain [Gallus gallus]

Protein Accession	gi|45384320
Mean Expression Ratio	1.04
Median Expression Ratio	1.04
Credible Interval	(0.805, 1.35)
Associated Peptides	1
Associated Spectra	1
Coverage	0.0985

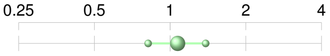

A	2.5	50	97.5	Sequence
1	0.82	1.1	1.4	LGEEFDETTPDDR

```
1       MVEAFCATWK LADSHNFDEY MKALGVGFAM RQVGNVTKPT VIISSEGDKV VIRTQSTFKN TEISFKLGEE FDETTPDDRN
81      CKSVVTLDGD KLVHVQKWDG KETNFVREIK DGRMVMTLTF GDVVAVRHYE KA
```

5.234 eukaryotic translation elongation factor 1 [Gallus gallus]

Protein Accession	gi|44969758
Mean Expression Ratio	0.96
Median Expression Ratio	0.96
Credible Interval	(0.767, 1.20)
Associated Peptides	2
Associated Spectra	2
Coverage	0.0367

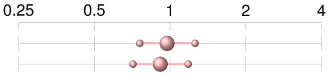

A	2.5	50	97.5	Sequence
1	0.76	0.98	1.3	AKDPFAHLPK
1	0.71	0.92	1.2	EKEKEK

```
1       MPVSGTLYTF PDNWRSFQAP IPAQYIGAQF RLRCSPPHFR FGTTNRSPDF LRQFPAGKVP AFEGDDGFCV FESNALAYYV
81      SHEELRGAPR EGAAQVLQWV NFADSDIVPP ASTWVFPPLG IMHYNKQATE QAKSEVRRAL SVLDAHLQSR TFLVGERVSL
161     ADISVVCALL WLYKQVLEPS FRQPFGNVTR WFLTCLHQPQ FKAVLGDVPL CPRMAQFDAQ KFAENPPQKD GVKKEKKEKE
241     KEKEKEKEKE KEAHGAAPEE ELDECEQALL AEPKAKDPFA HLPKSPFVLD EFKRKYSNED TAAVALPHLW QHFDPQGWSL
321     WYAEYRYPAE LSQTFMSCNL ITGMFQRLDK LRKNAFASVI LFGTNNDSSI SGVXLFRGQE LAFQLCPDWQ VDYESYTWRK
401     LDPGSAECRT LVTEYFSWEG EFAHVGKPFN QGKVFK
```

5.235 sirtuin [Gallus gallus]

Protein Accession	gi|52345464
Mean Expression Ratio	0.96
Median Expression Ratio	0.96
Credible Interval	(0.745, 1.24)
Associated Peptides	1
Associated Spectra	1
Coverage	0.037

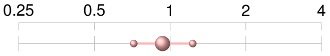

A	2.5	50	97.5	Sequence
1	0.72	0.94	1.2	GAGPPPAAAAATEPPGDAAAVSADGDVR

```
1       MADGEAPLLR PRDGGPGAAA ESVEPAPKRQ RLNSEDGVCG RGAPPAHRPD RGAGPPPAAA AATEPPGDAA AVSADGDVRA
81      REEDGGATTE GRSGADNRAA QRGLARAEPP PQPRRQGRGE GAEAAPGEDA AEAAIGCERA QRSNGAAGAP APQPDNFLLS
161     DEIIANGFHS CDSDEDDRAS HASSSDWTPR PRIGPYTFVQ QHLMLGTDPR TILKDLLPET IPPPELDDMT LWQIVINILS
241     EPPKRKKRKD VNTIDDAVKL LQECKKIMVL TGAGVSVSCG IPDFRSRDGI YARLAVDFPD LPDPQAMFDI EYFRKDPRPF
321     FKFAKEIYPG QFQPSLCHKF IALMDKEGKL LRNYTQNIDT LEQVAGIQRI IQCHGSFATA SCLICKYKVD CEVVRGDIFN
401     QVVPRCPRCL PDEPLAIMKP DIVFFGENLP EQFHRAMKYD KNEVDLLIVI GSSLKVRPVA LIPSSIPHEV PQILINREPL
481     PHLHFDVELL GDCDVIISEL CQRLGSEYTK LCYNSVKLSE ITEKPPRMHK ELEMHSSELP PTPLDISEDS GSPEQMTPPG
561     TSVVPSEHAA ECKVENSDPA SETKGICTEE KLQDTQASSE NPENPASELM NSETMKENGT NNGESKEKNE IVKKCWVNRS
641     AKEQISKRLD GTQYLFLPPN RYIFHGAEVY SDSEDDMISS SSCGSSSESG SCHSQSLDVE DESEIEEFYN GIEDEDAPER
721     EVEAAFEEDG VEQDAADESA YTNEAAGNDH PTSNKL
```

5.236 PREDICTED: similar to basement membrane-specific heparan sulfate proteoglycan core protein, partial

Protein Accession	gi|118101256
Mean Expression Ratio	1.04
Median Expression Ratio	1.04
Credible Interval	(0.807, 1.35)
Associated Peptides	1
Associated Spectra	1
Coverage	0.0116

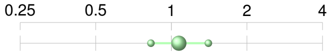

A	2.5	50	97.5	Sequence
1	0.83	1.1	1.4	DGTMTVDGGAPVQR

```
1      MSGSRLRLSQ LSVADSGEYV CRADLGSTSR EATVVVTVTS RDSSSYRLQS PIISIDPHSM AVAPGEDATF KCRIHDGAQP
81     INVTWRMGPG QHLQDNVKIS ANGSVISITG AHVGNQGAYH CVASNRFGVA SSVVNLLVQG APTVSVMPPG PVTVKEGKSL
161    SLECLGRGEP RPLVRWSRLG SRQKVEHQTL LHMDSQAVLQ LSPAKPEHAG TYICTAQSAL GSAQARVDVS VETAQRHPGA
241    PRVTAPPTVT VVAGDTATLH CSATGEPEPR IEWSKLRAPL PWQHRVVNGT LVIPRAAQQD SGQYICNASS PAGFTEVFVT
321    LDVETPPYAT SLPEDSAVRA GDTVQLQCLA HGTPPLLYTW DKLNGSLSPR AVPRAGLLRI SPALPSDAGT YRCMVTNRVG
401    RAETFARVTV HGDGGGDSGG PLAVRVTPGS LVRGVGSTAE FACTASDPRV HLEWLKDGGE LPPRHSVQDG VLRIAELAQG
481    AQGVYVCRAS TLHGQVEDRA TLTVQALPRA LINIRTAVQT VLAGMEVELE CLGLGEPQPH VTWSKVGGRI RPGVLVRAGT
561    LTIERVERAD AGQYRCTATN SVGTVQSHVI LHVQAAPHIA GQPEVKEVSV GSAAVLPCLA SGFPVPEISW SKLEGELPEG
641    TRVEGTALML PAVRLEDAGV YACAASNRRG QETAFYVLKV QERLVPYFTQ TPRSFLPLPT IKDAYKTFEI QITFRPDAAD
721    GMLLYNGQRK SSGADFISFG LVGGRPEFRF DAGSGMATIR DPTPLRLGQY HTVRLFRNLT RGSLQVDGQP PVNGTSQGKF
801    QGLDLNEELY LGGYPDYTIV AKTGLSRGFV GCVRQLRIQN EEVAFGELDL QAHGVSNCPT CQDQPCQNGG ICEDAESSTY
881    ICRCPQGFTG SNCEYSQALH CHPEACGPDA TCISRPDGHG YSCRCHLGKM GERCTEGEAV SVPSFDEAGA FISYPPLTNV
961    HHELRVEAEF LPRAPDGLLL FSAGKASPVE DFVALAMVSG HLEFHYELGS GTAVLRSVEP VALGRWHRVT AERVHKDGTM
1041   TVDGGAPVQR SSPGKSQGLN LRSPLYLGGV EPPLRPPTNA SFQGCIGEVS INGKKVDLSY SFLRSRGVGQ CGQSSPCLHA
1121   PCLHGGRCLP LPASSPPFRC LCTPGFSGPR CERAADRCLE HNPCLHGGTC KDNGCICPKG YAGPYCQHGA ALSELDQDWQ
1201   EGSGGS
```

5.237 SMC4 structural maintenance of chromosomes 4-like 1 [Gallus gallus]

Protein Accession	gi|45383133
Mean Expression Ratio	1.04
Median Expression Ratio	1.04
Credible Interval	(0.804, 1.35)
Associated Peptides	1
Associated Spectra	1
Coverage	0.00706

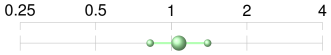

A	2.5	50	97.5	Sequence
1	0.82	1.1	1.4	AAIKDIDIK

```
1      MQVEKQDGRQ LGEGPAEAVS NEESDNRSLE EILGSISPPP PPAMTNEAGA PRLMITHIVN QNFKSYAGEQ TLGPFHKRFS
81     CIIGPNGSGK SNVIDAMLFV FGYRAQKIRS KKLSVLIHNS EEHTDIQSCS VEVHFQKIID KEGDDYEVIP NSNFCVSRTA
161    YRDNSSVYHI NGKKSTFKDV GILLRSHGID LDHNRFLILQ GEVEQIAMMK PKGQTEHDEG MLEYLEDLIG SARLRAPIQT
241    LCRRVETLNE QRGEKLNRVK MVEKEKDALE GDKNKAIEFL SLENKMFKEK NHICQYYIYD LQKRINDCET QKEKIHEDTK
321    EITEKSNMLS NEMKAKNKAV KDVEKKLNKI TKFIEENKEK FTQLDLQDVK VREKLKHAKS KAKKLEKQLQ KDKEKVEELK
401    NVPSSSEKNI SDATSKKELL EKAKDKEEEK LKQVMSSLQE ETREIQKEKE GKEKELMEFC KTVNDARSKM DVAQAELDIY
481    LTRYNTAVSQ LDEAKEALMT TSETLKQMKA AIKDIDIKLP TAEQELKEKE NKLEKLKKEE LNAQDLVRNL RQKVEEAKSS
561    LAQHRSRGKV LEALLEQKRS GSISGIHGRL GDLGAIDEKY DVAISSSCAA LDYIVVDTID IAQQCVEFLK KTEIGAATFI
641    ALDKMAVWKK NLQKIPTPEN APRLIDLVKV EDKSFSPAFY FALRDTLVVK DLEDATRIAF QRDKRWRVVT LKGEIIEQSG
721    TMTGGGGKVM KGRMGSSVVT DVSPEEVNRL ESELQRDSQR AVQCEEEKFQ LEEDITKLQK NVREMRNTLE KYTASIQSFL
801    EQEIRLKGQV KELEANVTAA APDKNKQKEL EKVLNSYKKD YERVSEQAGK MESEVKRLHN LIIDFNNRKL KTQQDKVDKI
881    NQEIDECTSA ITKAQVAIKT AHRNLKKSED SVLRTEKEIG DMRTEIKDLT EELTTLEDKA TEVLNDCRQA EEALPGVQEE
961    HRNLLQEMRA IQDDEHELRK EALNIKFKIE QIDSHISTHQ SKIKYWQKEI SKIIMHPIED KPPEELPVLS QEELEAIKDP
1041   DIITNQIALL EAQCHEMKPN LGAIAEYRRK EELYLKRVAE LDDITNERDR FRQAFEDLRK QRLNEFMAGF NVITNKLKEN
1121   YQMLTLGGDA ELELVDSLDP FSEGIMFSVR PPKKSWKKIF NLSGGEKTLS SLALVFALHH YKPTPLYFMD EIDAALDFKN
1201   VSIVAFYIYE QTKNAQFIII SLRNNMFEIA DRLIGIYKTH NTTKSVATNP KVIAVKGLTE LGIAEHSIAQ NQNA
```

5.238 proteasome (prosome, macropain) 26S subunit, non-ATPase, 9 [Gallus gallus]

Protein Accession	gi|57525182
Mean Expression Ratio	0.96
Median Expression Ratio	0.96
Credible Interval	(0.742, 1.23)
Associated Peptides	1
Associated Spectra	1
Coverage	0.0773

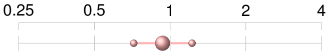

A	2.5	50	97.5	Sequence
**1**	**0.72**	**0.94**	**1.2**	**GVGTDGPLVDAEGFPR**

```
1      MSEDGSGRAM TLGEVQQLVR RKDELEAQIR ACYQLLEDQK GVGTDGPLVD AEGFPRADID LYQVRAARHS IACLQNDHKA
81     LMKQVEEALH QLHAREKEKH ARDEAEARAE AMSQSLPPAF AKVNAVTPES PASTSGLQVD DEIVEFGSVN VHNFKSLQNI
161    ATVVQHSEGR PLSVTVIRNG KKVHLGLTPK RWAGKGLLGC DIIPLQR
```

5.239 hypothetical protein [Gallus gallus]

Protein Accession	gi|53134115
Mean Expression Ratio	1.04
Median Expression Ratio	1.04
Credible Interval	(0.835, 1.30)
Associated Peptides	2
Associated Spectra	2
Coverage	0.0497

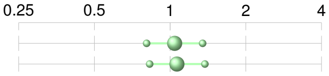

A	2.5	50	97.5	Sequence
**1**	**0.8**	**1.0**	**1.3**	**GLLFVGSGVSGGEEGAR**
**1**	**0.83**	**1.1**	**1.4**	**FKDKDGK**

```
1      MAEADIALIG LAVMGQNLIL NMNDHGFVVC AFNRTVSKVD DFLANEAKGT KVIGAHSLEE MVSKLKKPRR IILLVKAGSA
81     VDDFINKLVP LLETGDIIID GGNSEYRDTT RRCKELLQKG LLFVGSGVSG GEEGARYGPS LMPGGSKEAW PHIKTIFQSI
161    AAKVGSGEPC CDWVGEEGAG HFVKMVHNGI EYGDMQLICE AYHLMKDVVG MDHDEMSQVF EEWNNTELDS FLIEITANIL
241    KFKDKDGKYL LPKIRDSAGQ KGTGKWTAIS ALEYGVPVTL IGEAVFARCL SSLKDERVQA SKLLNGPKLT QFSGNKKAFL
321    EDIRKALYAS KIISYAQGFM LLRQAAKEFG WTLNYGGIAL MWRGGCIIRS VFLGKIKDAF DRNPELQNLL LDDFFKTAVE
401    KCQDSWRHVI STGVQHGIPM PCFTTALSFY DGYRHEVLPA NLIQAQRDYF GAHTYELLSK PGVFIHTNWT GHGGNVSSSA
481    YNV
```

5.240 PREDICTED: similar to GTP binding protein overexpressed in skeletal muscle [Gallus gallus]

Protein Accession	gi|50753839
Mean Expression Ratio	1.04
Median Expression Ratio	1.04
Credible Interval	(0.807, 1.34)
Associated Peptides	1
Associated Spectra	1
Coverage	0.066

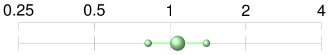

A	2.5	50	97.5	Sequence
**1**	**0.82**	**1.1**	**1.4**	**IFGGVEDGAEAEEAGNTYDR**

```
1      MTLNRGDKLR YLDKRRGSMP FSAHQHLHRR SMPVDERDLR ASLPQDELSG LVRCTSYSPG EAHRESWASD SSDSVISSGS
81     DSDGSLYKVI LLGEHGVGKT SLARIFGGVE DGAEAEEAGN TYDRSIMVDG EEASLVVFDI WEQDDSQWLQ NHCMKMGDAY
161    VIVYSVTDKV SFEKASELRI QLRRARQTED IPIILVGNKS DLVRSREVSV DEGRACAVVF DCKFIETSAA LHHNVKDLFE
241    GIVRQIRLRK DSKEDNARRM ANTKRRESIG KKAKRFLGRI VAKNNKKMAF KAKSKSCHDL SVL
```

5.241 radixin [Gallus gallus]

Protein Accession	gi|45382077
Mean Expression Ratio	0.961
Median Expression Ratio	0.961
Credible Interval	(0.773, 1.20)
Associated Peptides	2
Associated Spectra	2
Coverage	0.0274

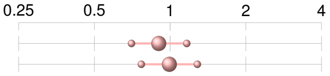

A	2.5	50	97.5	Sequence
1	0.7	0.9	1.2	LFFLQVK
1	0.77	1	1.3	FVIKPIDKK

```
1     MPKPINVRVT TMDAELEFAI QPNTTGKQLF DQVVKTVGLR EVWFFGLQYV DSKGYSTWLK LNKKVTQQDV RKENPLQFKF
81    RAKFFPEDVS EELIQEITQR LFFLQVKEAI LNDEIYCPPE TAVLLASYAV QSKYGDYNKE IHKLGYLAND RLLPQRVLEQ
161   HKLTKEQWEE RIQNWHEEHR GMLREDSMME YLKIAQDLEM YGVNYFEIKN KKGTELWLGV DALGLNIYEH DDKLTPKIGF
241   PWSEIRNISF NDKKFVIKPI DKKAPDFVFY APRLRINKRI LALCMGNHEL YMRRRKPDTI EVQQMKAQAR EEKHQKQLER
321   AQLENEKKKR EIAEKEKERI EREKEELMER LRQIEEQTMK AQKELEEQTR RALELDQERK RAKEEAERLE KERRAAEEAK
401   AALAKQAADQ MKNQEQLAAE LAEFTAKIAL LEEAKKKKEE EASEWQHKAF AAQEDLEKTK EELKSVMSAP PPPPPPPVIP
481   PTENEHDEHD ENNAEASAEL SSDGVMNHRS EEERVTETQK NERVKKQLQA LSSELAQARD ETKKTQNDVL HAENVKAGRG
561   KYKTLRQIRQ GNTKQRIDEF EAM
```

5.242 calmodulin 2 [Homo sapiens]

Protein Accession	gi|4502549
Mean Expression Ratio	0.961
Median Expression Ratio	0.961
Credible Interval	(0.77, 1.20)
Associated Peptides	2
Associated Spectra	2
Coverage	0.174

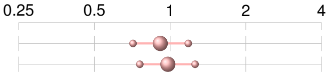

A	2.5	50	97.5	Sequence
1	0.71	0.92	1.2	DTDSEEEIR
1	0.76	0.98	1.3	EAFSLFDKDGDGTITTK

```
1     MADQLTEEQI AEFKEAFSLF DKDGDGTITT KELGTVMRSL GQNPTEAELQ DMINEVDADG NGTIDFPEFL TMMARKMKDT
81    DSEEEIREAF RVFDKDGNGY ISAAELRHVM TNLGEKLTDE EVDEMIREAD IDGDGQVNYE EFVQMMTAK
```

5.243 lectin-associated matrix protein [Gallus gallus]

Protein Accession	gi|126256509
Mean Expression Ratio	0.961
Median Expression Ratio	0.962
Credible Interval	(0.743, 1.24)
Associated Peptides	1
Associated Spectra	1
Coverage	0.0567

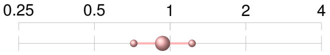

A	2.5	50	97.5	Sequence
1	0.72	0.94	1.2	NKKPGEVK

```
1     ELRNKKPGEV KELVLDNCRS DDGKIVGLSS DFENLEFLSM INVNLLSISN LPKLNKLRKL ELSDNRISGG LEVLAERTPN
81    LTHLNLSGNK IKDINTLEPL KKLPNLHSLD LFNCEVTMLI NYRESVFTLL PQLTYLDGFD A
```

5.244 SAPS domain family, member 3 [Gallus gallus]

Protein Accession	gi|71894719
Mean Expression Ratio	1.04
Median Expression Ratio	1.04
Credible Interval	(0.815, 1.32)
Associated Peptides	1
Associated Spectra	2
Coverage	0.0160

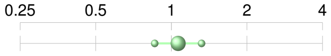

A	2.5	50	97.5	Sequence
**2**	**0.86**	**1.1**	**1.3**	**FADQDDIGNVSFDR**

```
1      MFWKFDLHSS SHIDTLLERE DVTLKELMDE EDVLQECKAQ NRKLIEFLLK SECLEDLVSF IIEEPPQDMD EKIRYKYPNI
81     SCELLTSDVS QINDRLGEEE SLLMKLYSFL LNESPLNPLL ASFFSKVLSI LISRKPEQIV DFLKKKHDFV DLVIKHIGTS
161    AIMDLLLRLL TCIEPPQPRQ EVLNWLNEER IIQRLVEIVH PSQDEDRHSN ASQSLCEIIR LSRDQMLQVQ NSSEPDPLLA
241    SLEKREIIEQ LLSNIFHKEK NESAIVSAIQ ILLTLLETRR QTFEGHIEIC PPGMSNSTYS VNKSVLEAIK ARLSSFHELL
321    LEPPKKSVMK TTWGVLDPPV GNTRLNVIRL ISSLLQTNTS SVNQELIELN SIGVILDMFF KYTWNNFLHT QVEICIALIL
401    ASPLESTENG TITDQDSTGD NLLLKHLFLK CQLIERILEA WEMNEKKQAE GGRRHGYMGH LTRIANCIVH STDKGPNSTL
481    VQQLIKELPE EVRERWETFC TSSLGETNKR NTVDLVTTCH IHSSSDDEID FKETGFSQDS SLQQAFSDYQ MQQMTSNFID
561    QFGFNDEKFA DQDDIGNVSF DRVSDINFTL NTNESGNIAL FEACCKERIQ QFDDGGSDEE DIWEEKHIAF TPESQRRSSS
641    GSTDSEESTD SEEEDGTKQD LFESHANTED KMEVDLNEPP NWSANFDVPM ETAHGTNLDS VGSDVWSTEE PMPAKETGWA
721    SFSEFTSSLS STDSLRSNSP VEMETNTEPM DPLSANATGL ATQLETPGSV AMEASSDGEE DAENADKVTE TVMNGSMKET
801    LSLTVDAKTE TAVFKSEEGK LSTSQDASCK YVVEENAEVA EEAPSALQPA NSSPEQRTDQ RTLLGETSVN GPV
```

5.245 vacuolar protein sorting 26 [Mus musculus]

Protein Accession	gi|19526826
Mean Expression Ratio	1.04
Median Expression Ratio	1.04
Credible Interval	(0.8, 1.34)
Associated Peptides	1
Associated Spectra	1
Coverage	0.0183

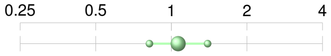

A	2.5	50	97.5	Sequence
**1**	**0.82**	**1.1**	**1.4**	**VNLAFK**

```
1      MSFLGGFFGP ICEIDVALND GETRKMAEMK TEDGKVEKHY LFYDGESVSG KVNLAFKQPG KRLEHQGIRI EFVGQIELFN
81     DKSNTHEFVN LVKELALPGE LTQSRSYDFE FMQVEKPYES YIGANVRLRY FLKVTIVRRL TDLVKEYDLI VHQLATYPDV
161    NNSIKMEVGI EDCLHIEFEY NKSKYHLKDV IVGKIYFLLV RIKIQHMELQ LIKKEITGIG PSTTTETETI AKYEIMDGAP
241    VKGESIPIRL FLAGYDPTPT MRDVNKKFSV RYFLNLVLVD EEDRRYFKQQ EIILWRKAPE KLRKQRTNFH QRFESPDSQA
321    SAEQPEM
```

5.246 Chain A, Complex Between Rabbit Muscle Alpha-Actin: Human Gelsolin Domain 1

Protein Accession	gi|7766848
Mean Expression Ratio	1.04
Median Expression Ratio	1.04
Credible Interval	(0.803, 1.34)
Associated Peptides	1
Associated Spectra	1
Coverage	0.0477

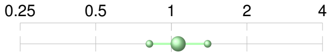

A	2.5	50	97.5	Sequence
**1**	**0.82**	**1.1**	**1.4**	**VAPEEHPTLLTEAPLNPK**

```
1      MCDEDETTAL VCDNGSGLVK AGFAGDDAPR AVFPSIVGRP RHQGVMVGMG QKDSYVGDEA QSKRGILTLK YPIEXGIITN
81     WDDMEKIWHH TFYNELRVAP EEHPTLLTEA PLNPKANREK MTQIMFETFN VPAMYVAIQA VLSLYASGRT TGIVLDSGDG
161    VTHNVPIYEG YALPHAIMRL DLAGRDLTDY LMKILTERGY SFVTTAEREI VRDIKEKLCY VALDFENEMA TAASSSSLEK
241    SYELPDGQVI TIGNERFRCP ETLFQPSFIG MESAGIHETT YNSIMKCDID IRKDLYANNV MSGGTTMYPG IADRMQKEIT
321    ALAPSTMKIK IIAPPERKYS VWIGGSILAS LSTFQQMWIT KQEYDEAGPS IVHRKCF
```

5.247 glycinamide ribonucleotide synthetase-aminoimidazole ribonucleotide synthetase-glycinamide ribonucl

Protein Accession	gi|68299602
Mean Expression Ratio	0.963
Median Expression Ratio	0.963
Credible Interval	(0.743, 1.24)
Associated Peptides	1
Associated Spectra	1
Coverage	0.0239

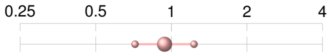

A	2.5	50	97.5	Sequence
1	0.72	0.94	1.2	LVVVGPEVPLAAGIVDDLTAAGVR

```
1       MADRVLVIGS GGREHALAWK LAQSPHVKQV FVAPGNAGTA NSGKISNSAV SVSNHAALAQ FCRDQEIRLV VVGPEVPLAA
81      GIVDDLTAAG VRCFGPTARA AQLESSKSFT KSFLDRHGIP TARWKSFTDP KAACSFINSA NFPALVVKAS GLAAGKGVIV
161     ASNKEEACKA VNEIMQDKTF GTAGETVVVE ELLEGEEVSC LCFTDGVTIA PMPPAQDHKR LKDGDEGPNT GGMGAYSPAP
241     QISKDLLLKI RETVLQKTVD GMRKEGIPYL GVLYAGLMLT KDGPKVLEFN CRFGDPECQV ILPLLKSDLY EVMQAVINKK
321     LSSSMPIWYE DSAAVTVVMA SEGYPGTYPK GLEITGLSKA KELGLEVFHA GTALKDGKVV TNGGRVLTVT AIKEDLMTAL
401     QEANKGVAAI NFKGSIYRKD IGYRAIAFLS QSRGLTYKNS GVDIAAGNIL VQKIKPLAAA TSRSGCNAEL GGFAGLFDLK
481     AAGYKDPILV SGTDGVGTKL KIAQVCKKHD TIGQDLVAMC VNDILAQGAE PLFFLDYFAC GKLDVEVAQG VIAGIAEACQ
561     KAGCALLGGE TAEMPGMYPP GEYDLAGFAV GAVERGQMLP QLERIADGDV VIGVASSGVH SNGYSLVRRI VEKSSLDFSS
641     QVGVSGDQTL GDLLLTPTKI YSKTLLPVLR SGHVKAYAHI TGGGLLENIP RVLPDSFGVV LDALSWKIPE IFCWLHKEGN
721     LSEEEMARTF NCGIGAVLVV QKELAQQVLK DVQKHEAAWL IGKVVPLQKG SAHVKVHNLL QALQANRSLS VHSHIQGKIQ
801     TNKVKVAVLI SGTGTNLEAL INSTKKPTSF AEIVLVVSNK AGVEGLRKAE RAGIPTRVID HKLYGSRTEF DSAVDRVLEE
881     FSVELICLAG FMRILSGPFV KKWEGKILNI HPSLLPSFKG ANAHKLVLEA GVRVTGCTVH FVAEEVDAGA IIFQEAVPVK
961     IGDTVETLSE RVKEAEHRAF PAALQLVASG AVQVGEAGKI CWK
```

5.248 myosin binding protein C, cardiac [Gallus gallus]

Protein Accession	gi|45384120
Mean Expression Ratio	1.04
Median Expression Ratio	1.04
Credible Interval	(0.887, 1.21)
Associated Peptides	6
Associated Spectra	6
Coverage	0.0637

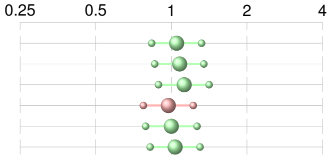

A	2.5	50	97.5	Sequence
1	0.83	1.1	1.3	DGSAEWTPALPGLTER
1	0.86	1.1	1.3	SDSQPDVDVWEILR
1	0.89	1.1	1.4	KAPPSEYEK
1	0.77	0.97	1.2	TGTTYKPPSYK
1	0.79	1	1.3	KLDPAYQVDK
1	0.82	1.0	1.3	HEDHCVFIIEGAEKEDEGVYR

```
1       MPEPAKKAVS AFTKKPKTTE VAAGSTAVFE AETEKTGIKV KWQRAGTEIT DSEKYAIKAE GNKHSLTISN VGKDDEVTYA
81      VIAGTSKVKF ELKVKEPEKS EPVAPAEASA PAASELPAPP VESNQNPEVP PAETQPEEPV DPIGLFVTRP QDGEVTVGGN
161     ITFTAKVAGE SLLKKPSVKW FKGKWMDLAS KVGKHLQLHD NYDRNNKVYT FEMEIIEANM TFAGGYRCEV STKDKFDSSN
241     FNLIVNEAPV SGEMDIRAAF RRTSLAGGGR RMTSAFLSTE GLEESGELNF SALLKKRDSF LRTANRGDGK SDSQPDVDVW
321     EILRKAPPSE YEKIAFQYGI TDLRGMLKRL KRIKKEEKKS TAFLKKLDPA YQVDKGQKIK LMVEVANPDA DVKWLKNGQE
401     IQVSGSKYIF EAIGNKRILT INHCSLADDA AYECVVAEEK SFTELFVKEP PILITHPLED QMVMVGERVE FECEVSEEGA
481     TVKWEKDGVE LTREETFKYR FKKDGKKQYL IINESTKEDS GHYTVKTNGG VSVAELIVQE KKLEVYQSIA DLTVKARDQA
561     VFKCEVSDEN VKGIWLKNGK EVVPDERIKI SHIGRIHKLT IEDVTPGDEA DYSFIPQGFA YNLSAKLQFL EVKIDFVPRE
641     EPPKIHLDCL GQSPDTIVVV AGNKLRLDVP ISGDPTPTVS GRKVNKKGEL VHQSNEDSLT PSENSSDLST DSKLLFESEG
721     RVRVEKHEDH CVFIIEGAEK EDEGVYRVIV KNPVGEDKAD ITVKVIDVPD PPEAPKISNI GEDYCTVQWQ PPTYDGGQPV
801     LGYILERKKK KSYRWMRLNF DLLKELTYEA KRMIEGVVYE MRIYAVNSIG MSRPSPASQP FMPIAPPSEP THFTVEDVSD
881     TTVALKWRPP ERIGAGGLDG YIVEYCKDGS AEWTPALPGL TERTSALIKD LVTGDKLYFR VKAINLAGES GAAIIKEPVT
961     VQEIMQRPKI CVPRHLRQTL VKKVGETINI MIPFQGKPRP KISWMKDGQT LDSKDVGIRN SSTDTILFIR KAELHHSGAY
1041    EVTLQIENMT DTVAITIQII DKPGPPQNIK LADVWGFNVA LEWTPPQDDG NAQILGYTVQ KADKKTMEWY TVYDHYRRTN
1121    CVVSDLIMGN EYFFRVFSEN LCGLSETAAT TKNPAYIQKT GTTYKPPSYK EHDFSEPPKF THPLVNRSVI AGYNTTLSCA
1201    VRGIPKPKIF WYKNKVDLSG DAKYRMFSKQ GVLTLEIRKP TPFDGGFYTC KAVNERGEAE IECRLDVRVP Q
```

5.249 PREDICTED: similar to Alanyl-tRNA synthetase domain containing 1 [Gallus gallus]

Protein Accession	gi|118103027
Mean Expression Ratio	1.04
Median Expression Ratio	1.04
Credible Interval	(0.802, 1.34)
Associated Peptides	1
Associated Spectra	1
Coverage	0.0186

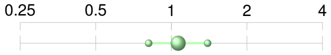

A	2.5	50	97.5	Sequence
**1**	**0.81**	**1.1**	**1.4**	**DWEGDEEVER**

```
1     MARQHAKTLW YDRPRYVFLE FCVEDSTDVQ VVIEDHRLVF SCKNADGVEF YNEINLYARV NSKDSREKRS DRSITCFMRK
81    WKEKVAWPRI TKENIKPAWL SVDFDNWRDW EGDEEVERAM VEEYAELLQK VTDKAPPPTM DDLDFATKVV SCQEAELRPE
161   SGGEPRARSG AARPGPDSGS LSPQPDDRGL IGAVPVLRVT RRGAEAVHFV ETALEPGSAV LLSLDWERRF DHMQQHSGQH
241   LITAIAEQMF GFKTTSWELG RQQSVIELDT PSMTTEQMEA LEQSVNEKIR ERIPVTVREL AADDPEVETV RSRGLPGDHT
321   GPVRVVDIEG IDSNMCCGTH VSNLSDLQVI KLICTEKGKK NKTNLVFLAG NRVLKSVEQS HRTEKALTSL LKNGPGEHVE
401   AVKRLQSSVK LLQKNNLNLL RDIAVLIARD FKSKPVQSPL FVLHRKEGDS EFMNIIANEI GKEETLLFLT VGDEKEAGLF
481   LLAGSVEAVE NLGPRVAELL EGKGAGKRGR YQGKATKMSR RGEVQALLQE FISQQTDEA
```

5.250 PREDICTED: hypothetical protein [Gallus gallus]

Protein Accession	gi|118087631
Mean Expression Ratio	1.04
Median Expression Ratio	1.04
Credible Interval	(0.804, 1.34)
Associated Peptides	1
Associated Spectra	1
Coverage	0.0152

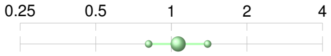

A	2.5	50	97.5	Sequence
**1**	**0.81**	**1.1**	**1.4**	**LNLVEAFVVDTELR**

```
1     MRPLRDKNQP CSFCERIGKR ALKAFSGQAG RSSGKLAGRA QWQRKENGTG IRFVLLGNQK LESVVLSKMN FESFVRDLLL
81    VRHYRVEVYK NKAGSKSVKE NDWYLAYKGS PGNLAQFEEV LFANNDMSMA IGVVGVKLSS ADGQRVIGVG YVDTTLRKLS
161   VCEFPDNDQF SNLEALLVQL GPKECVLPGG DTAGEMGKLR QVVQRGGILI TDRKKADFTT KDIVQDLNRL LKSRKGEQMN
241   SAALPEMEKQ VAVSSLSAVI KFLELLSDDS NFGQYELTTF DLSQYMVLDN AAVQALNLFQ SSVENANNTQ SLAGLLNKCR
321   TPQGQRLVNQ WIKQPLMDKN RIEERLNLVE AFVVDTELRQ GLQEDLLRRF PDLNRLAKKF QRQAATLQDC YRMYQAINQL
401   PNVVQALEKH EGAHQMLLLA VFITPLNDIH SDFSKFLEMI ETTLDMDKVE NHEFLVKASF DPNLTELREK MNELEESMQT
481   LLKSAAKELG LEAGKSIKLE SNSQFGHHFR ITCKEEKVLR NNMKYKITDT QKNGVKFTNS KLSAINEEYI KNREEYEEAQ
561   DAIVKEIINI ASGYAEPIQT MNDVIAQLDA IVSFAHVSNG APVPYVRPVI LEKGQGRIVL KGARHPCIEV QDEVAFIPND
641   VTFEKGKQMF HIITGPNMGG KSTYIRQTGV IVLMAQIGCF VPCNSAEITI VDCILARVGA GDSQLKGVST FMAEMLETAS
721   ILRTASENSL IIIDELGRGT STYDGFGLAW AISEYIASKI CAFCMFATHF HELTALADQV PTVNNLHVTA LTSDDTLTML
801   YRVKAGVCDQ SFGIHVAELA AFPKHVIESA REKALELEEF QDIGRPKESE GEPAAKRCYR EREEGEKIIQ DFLSQVKALP
881   LTDMSEEDIK TKLKQLRADV LAKNNGFVNE IISRTKVTP
```

5.251 apolipoprotein A-IV [Gallus gallus]

Protein Accession	gi|45384392
Mean Expression Ratio	1.04
Median Expression Ratio	1.04
Credible Interval	(0.804, 1.34)
Associated Peptides	1
Associated Spectra	1
Coverage	0.0464

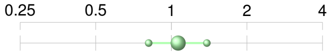

A	2.5	50	97.5	Sequence
**1**	**0.81**	**1.1**	**1.4**	**LAPYADEVHQQIGTNIR**

```
1      MSPKAALLLV LLAVAGTRAD VSPDQVATVL WRYFTELGSS AKETVDQLQQ AEITKQLNTL LQSNLQSANS YAEELQRRLV
81     PFATELQAQL VQDSQRLKKQ IQQELAELQA KLAPYADEVH QQIGTNIREL QAKLSPYADE LRSQVDRGTG ELRRALEPFA
161    TELREKLQDN ADSIQASLGP YAERLQQQID SSVEGLKGQL TPLADELKEQ VAQSVEGLRK GLSPYAQEVQ DGLNRQLQSL
241    TAQMERAAEE LRSRLAASSE EMRAQLSPLA QELQEALRGD AEAMQQRLAP LAQQLDERLA QTVEAFRQQA APISETFRQQ
321    LVQRLEEMKQ KLESGTAGVE DHLDLLEKEV REKVATFLST TEQAES
```

5.252 adipose differentiation-related protein [Gallus gallus]

Protein Accession	gi|71894785
Mean Expression Ratio	0.963
Median Expression Ratio	0.964
Credible Interval	(0.743, 1.24)
Associated Peptides	1
Associated Spectra	1
Coverage	0.0464

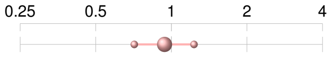

A	2.5	50	97.5	Sequence
**1**	**0.71**	**0.94**	**1.2**	**KNTGQNDGDESHSAEHIESR**

```
1      MALAAIDPQQ NIVSRVVNLP LVSSTYDMVS TAYITTKDNH PYLKSVCEIA EKGVKTITSV AMTSAMPIIQ KLEPQIVVAN
81     NYACIGLDKI EERLPILNQP TDKVVANAKG VVVGAREAVT TTVSGAKETV AHKITGVVGK TKEAVQDSVE ITKSVVNGGI
161    NTVLGSRVVQ MMSSGMDSAL TKSETLVDQY LPLTEAELER EAAKVEGFEV GVQKPSYYVR LGSLSSKFRA RAYQQALNKV
241    RDAKQKSQET ISQLHNTVSL IEYARKNMNS ANQKLLGAQE KLYQSWVEWK KNTGQNDGDE SHSAEHIESR TLAIAQSLTQ
321    QLQTTCLTLV TSIQGLPQSV QDQVYSVRSM AGDVYEIFRS ASSFQELSDS FLTTSKGQLK KMKESLDDVM DYLVNNTPLN
401    WLVGPFYPQL PGTQHAENEG EGEKNSSQED K
```

5.253 PREDICTED: similar to neuroblastoma-amplified protein [Gallus gallus]

Protein Accession	gi|118089036
Mean Expression Ratio	0.963
Median Expression Ratio	0.964
Credible Interval	(0.742, 1.24)
Associated Peptides	1
Associated Spectra	1
Coverage	0.00336

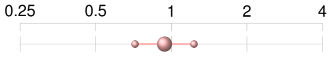

A	2.5	50	97.5	Sequence
**1**	**0.72**	**0.94**	**1.2**	**EDLTLPLK**

```
1      MATAGGEGAP RAEGMVAVNE EEDGEENILY DLLVNAEWPP ETELQPRGNR KHGASFIITR AITGPALLLL RYIWYSPAKF
81     SLPSGLVRLV NKQINWHLVL ASNGKLLAVV QDQRVEMRSA KDDFGSTVGI CQVPKDPNPQ WRRVAWSHDC TLLAYAESSG
161    TVRVFDLMGS ELLVISPTAS FSGDLSYAIA GLIFLEYKAS AQWSAELLVI NYRGELRSYL VSVGTNQSFQ ESHSFSFSSH
241    YAHGITAVIY HPGHRLLLVG GCETDEDGVS KATACGISAW RVLSGSPHYK QVTNSFLGMF LQAQRRGLLR IMNLRFYSRR
321    GTEQDGIFKM NLSPDGTLLA AIHFSGKLTI WSIPSLRQQG EWDQTDQPGY DEVNPDWKLS SEKRKKIKDK ESYYPLIDVN
401    WWANSSVILA RCSGALTVSS VKTLRNLLGK SCEWFESSPQ VTSAHDGGFL SLECEIKLVP KRLRLESRPG DEDEGEDDSD
481    SDDENSAKDR YFSYLKQGLY FVTEMERFAP PRKRPRTITK NFRLVSLRST TPEELYQRKI DNEEYDEALS LAQTYGLDSD
561    LVYQRQWRKS AVNVASIQDY LSKIKKRAWV LHECLERVPE NVDAAKKLLQ YGLKGTDLEA LVAIGKGEDG GRFILPGEAD
641    IGIPYENFLS PDEETDTKKE KEARKHQELL LSLNFSKLTL EQKELCRSRL KLLTYLDRLE TYEEILGGPH SAEQHYDGEF
721    FKKFRNQNIV LSARTYARES NVRALEILFT FHGSALLPHR QAILSNFPET TSPHEYAFLL PEACYEQGTL KIIPWNEQKH
801    REEDWCEKAD CRTIVEPTLQ DESEFLYESQ PELLKYRTTE LSVELVTDWY LTRAQEIEKY AMQVDCALSL VRLGMERNIP
881    GLQVLCDNLI TLETVVYETD GDRTLTLKEL VEMKDIEKLR LLMKNSSDEK YVKNVYQWMI PFLHRCENQS PGLANSLFKE
961    YLVTLAKEDL TLPLKIFQNS KPACQQKIIP EQDQLMITAL ECIYSCERDD QLALCYDILE CLPQRGYGPE TDKTSSLHDA
1041   VDELERILSV SELLEKHGLQ KPVSFVKDTK DNAEEARKLM IRLTRHTGRK QPSVSEMQWK ELLQDMLDMQ QKVYICLQSD
1121   ACYEIFTESL LCSSSIDNIH LAGQMMHCSI WSVDQPVSSK GKPQYRVSYT RSIELVLAAS REYFNSSTSL TDSCMDLARC
1201   CLQLIVDCPS AIQEELDLIR ALGYLEEFGV KILPLQVRLC SDRLGLIKDC LSQLPTNYKQ SAKLLGLANL LRVAGDDQME
1281   RKGQVLILLV EQALSFQDYK AASMHCQELM TAGYAKSWEV CSQLGQSEGF NDLGMRQELM AFALTHCPPS AIEALLGVSS
1361   SLQTQILYQA VNYQLLPSEG GENVNTSGPS SLMSKDTEDD TNISASQSAD LLYWTTAKTM KVLSNTTMTT KAMLHAVSDG
1441   QWWKRSLTYL RPLHGQELGD VLKSGLGENV TVEKQGCHPF YESLIADPYV AESEISYGTY QNNSLESFAE VLLRTEKLTE
1521   TKSEAKDLLP TTEVLLQLAS DALPKDMTLA LAYLLALPQV VDANKCFEKQ LHSAISLQLA SYYYSLQIYA RLAPCFKDKC
1601   HPLYRADPEE LIKMVTQHVT RYAYADWPEE IATLINQLHY YNERLLDFTQ AQILQGLGKG VDVQRFTADG QYKRETILGL
1681   AETLEENVYK IALSLAQRYS VPLWEVYMIH LEFLFTDSGL STVEIEERAQ SLGLFETLKT SPETLYEHMV KYVYPSIEGR
1761   DHQRLLYYFT LLENCGCSEV VKHTVKPETH IRLLKKFKAV APGLNYKKLM DENENPLETL EPILTSQNIL SISKLAPKIP
1841   KKDGSMLSPS SLYAVWLQNL FWNGDHHLIK KIPETMDEWL HAYDVCSKYL DRLDPDDVIT FIDAITFSSK AVTKLPVEAR
1921   IEVTKKAVEA VKHLSEKSRK KPSENDMGDA KDPAVAYEKT LNHLQQSLAH LKTLTCSFIT YLKNSQQDIL QKYGYLYDLS
2001   RSERDQIHEQ AVTMCIDGQP LNMIQQLIEV AVGDLCLSTK DIVQCAIKRI ICMLSGNDGS TSVKDPLGIL EGIVSAVHAS
2081   VEKGEEVVSS DDLLEWLRPF CGDDSLPVKP RIRVLQILEQ DFHLNDEDSK LLVYFRTQAV LRACWPETKV DTADIESEEK
2161   RCELFLRLLE SSHKPCEFQH LVLLLQAWPP MEMSSRSCAD DNPWVKLVTV MLQRYPPEEK DSMGNEILKI CRSLYGTKYK
2241   LSVECIKELC LLLLNHSLLL PSLKLLVESM DQDLHTMALK QITAVTKVDD SNCDAEILSL LLNAKLVVKC ISTAFYPRLI
2321   DHLLANQAEG GWDVEEIAKQ LKEAGFNAEA GSLLLSYRGT HPALRTFTSA LQTIQHWI
```

5.254 p47 protein [Gallus gallus]

Protein Accession	gi|71894957
Mean Expression Ratio	1.04
Median Expression Ratio	1.04
Credible Interval	(0.831, 1.29)
Associated Peptides	2
Associated Spectra	2
Coverage	0.084

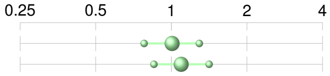

A	2.5	50	97.5	Sequence
1	0.78	1	1.3	SYQDPSNAQFLDDIR
1	0.85	1.1	1.4	LGATPEEESAYVAGER

```
1       MADREEALRE FVAVTGAEEE RARFFLESAG WDLQIALASF YEDGGDEDIL TLPQPTPSSV SRGTAASDHR VTSFRDLVHA
81      QEDDDEEEEG QRFYAGGSER SGQQIVGPPR KKSPNELVED LFKGAKEHGA VAVDRTAKSS GESSKPKPFA GGGYRLGATP
161     EEESAYVAGE RRHNSVQDVH VVLKLWKTGF SLDSGELRSY QDPSNAQFLD DIRRGEVPAE LRRLARGGQV NLDMEDHRDE
241     EYVKPKSVFK AFTGEGQKLG STAPQVLSTS SPAQQAENEA KASSAIAIDE SEPVTNIQIR LADGGRLVQK FNHNHRIRDI
321     RLFIVDARPA MAATSFVLMT TFPNKELTDE NQTLKEANLL NAVIVQRLT
```

5.255 coatomer protein complex, subunit epsilon [Gallus gallus]

Protein Accession	gi|57530593
Mean Expression Ratio	1.04
Median Expression Ratio	1.04
Credible Interval	(0.8, 1.33)
Associated Peptides	1
Associated Spectra	1
Coverage	0.0552

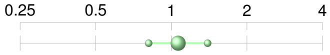

A	2.5	50	97.5	Sequence
1	0.81	1.1	1.4	NSFYIGAYQAAINEAQR

```
1       MASGAGAGPA GQGGEADELF DVKNSFYIGA YQAAINEAQR IKPSNPEKET ERDVFLFRSY IAQRKYGVVL DEIKANASPE
81      LQAVRMFAEY LSNESQRDAI VADLDKKMAK SVDVANTTFL LMAASIYFHD KNPDAALRTL HQGESLECMA MMIQILLKLD
161     RLDLARKELK KMQEQDEDAT LTQLATAWVN LAIGGEKLQD AYYIFQEMAD KCSSTLLLLN GQAACYMAQG KWDDAEGVLQ
241     EALDKDSGHP ETLINFVVLS QHLGKPPEVT NRYLSQLKDA HKNHPFIKEY QAKENDFDRL AMQYAPSA
```

5.256 hypothetical protein [Gallus gallus]

Protein Accession	gi|53136666
Mean Expression Ratio	0.963
Median Expression Ratio	0.964
Credible Interval	(0.743, 1.24)
Associated Peptides	1
Associated Spectra	1
Coverage	0.0308

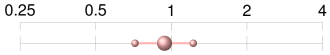

A	2.5	50	97.5	Sequence
1	0.72	0.94	1.2	KPAVVAK

```
1       MGFGDLKSAA GLRVLNDFLA DKSYIEGYVP SQADIAVFEA VGAPPPAELF HALRWYNHIK SYEKQKASLP GIKKALGKYG
81      PADVEDTTGA ATDSKDDDDI DLFGSDDEEE SEEAKKLREE RLAQYESKKS KKPAVVAKSS ILLDVKPWDD ETDMAKLEEC
161     VRSIQADGLV WGSSKLVPVG YGIKKLQIQC VVEDDKVGTD MLEERITAFE DYVQSMDVAA FNKILSS
```

5.257 myosin heavy chain 6 [Gallus gallus]

Protein Accession	gi|45382109
Mean Expression Ratio	1.04
Median Expression Ratio	1.04
Credible Interval	(0.8, 1.34)
Associated Peptides	1
Associated Spectra	1
Coverage	0.00723

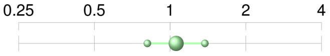

A	2.5	50	97.5	Sequence
**1**	**0.81**	**1.1**	**1.4**	**AYVEAEITESSGGK**

```
1       MMDMTEFGEA APFLRKSEKE LMMLQTVAFD GKKKCWVPDD KKAYVEAEIT ESSGGKVTVE TTDGRTMTIK EDDVQSMNPP
81      KFDMIEDMAM LTHLNEASVL YNLRKRYSNW MIYTYSGLFC VTINPYKWLP VYKSEVVAAY KGKRRSEAPP HIFSIADNAY
161     HDMLRNRENQ SMLITGESGA GKTVNTKRVI QYFATVAALG EPGKKSQPAT KTGGTLEDQI IQANPALEAF GNAKTLRNDN
241     SSRFGKFIRI HFGTTGKLSS ADIEIYLLEK SRVIFQQPGE RDYHIFYQIL SGKKPELLDM LLVSTNPYDY HFCSQGVVTV
321     DNLDDGEELM ATDQAMDILG FVPDEKYGAY KLTGAIMHFG NMKFKQRPRE EQAEADGTES ADKAAYLMGI NSSDLVKGLL
401     HPRVKVGNEY VTKGQSVEQV LYAVGALSKA VYDRMFKWLV VRINKTLDTK LPRQFFIGVL DIAGFEIFDF NSFEQLCINY
481     TNEKLQQFFN HHMFVLEQEE YKKEGIEWVF IDFGMDLQAC IDLIEKPLGI LSILEEECMF PKATDMTFKA KLYDNHLGKS
561     PNLQKPRPDK KRKYEAHFEL IHYAGSVPYN IIGWLEKNKD PLNETVVGIF QKSSNKLLAS LFESYVGADS ADQGGEKKRK
641     KGASFQTVSS LHKENLNKLM TNLRSTAPHF VRCIIPNESK TPGEMDAFLV LHQLRCNGVL EGIRICRKGF PNRVLYADFK
721     QRYRILNPGA IPEDKFVDSR KAAEKLLASL DIDHNQYRFG HTKVFFKAGL LGHLEEMRDE RLAKILTMIQ ARARGRLMRI
801     EFQKIVERRD ALLVIQWNIR AFMAVKNWPW MKLFFKIKPL LKSAETEKEM ANMKEEFLKL KEALEKSEAR RKELEEKQVS
881     LVQEKNDLLL QLQAEQDTLA DAEERCDLLI KSKIQLEAKV KELTERVEDE EEMNSELTSK KRKLEDECSE LKKDIDDLEI
961     TLAKVEKEKH ATENKVKNLT EEMATLDENI SKLTKEKKSL QEAHQQVLDD LQAEEDKVNT LSKAKVKLEQ QVDDLEGSLE
1041    QEKKVRMDLE RAKRKLEGDL KLTQESVMDL ENDKLQMEEK LKKKEFEMSQ LNSKIEDEQA IVMQLQKKIK ELQARIEELE
1121    EELEAERAAR AKVEKQRSDL ARELEVLSER LEEAGGATAA QLEMNKKREA EFLKLARDLE EATLHYEATA AALRKKHADS
1201    VAEMGEQLDN LQRVKQKLEK EKSELKMEVD DLTSNMEQTV KGKANAEKLC RTYEDHLNET KTKLDEMTRL MNDLTTQKTK
1281    LQSENGEFVR QLEEKESLIS QLSRGKTSFT QQIEELRRQL EEETKSKNAL AHALQAARHD CDLLREQYEE EQEAKAELQR
1361    ALSKGNAEVA QWRTKYETDA IQRTEELEDA KKKLAARLQE AEEAIEAANA KCSSLEKTKH RLQNELEDMM IDLEKANSAA
1441    ASLDKKQRGF DKIINDWKQK YEESQAELEA SQKEARSLST ELFKLKNAYE ETLDHLETLK RENKNLQEEI SDLTNQISEG
1521    NKNLHEIEKV KKQVEQEKSE VQLALEEAEG ALEHEESKTL RFQLELSQLK ADFERKLAEK DEEMENIRRN QQRTIDSLQS
1601    TLDSEARSRN EAIRLKKKME GDLNEMEIQL SHANRHAAEA TKSARGLQTQ IKELQVQLDD LGHLNEDLKE QLAVSDRRNN
1681    LLQSELDELR ALLDQTERAR KLAEHELLEA TERVNLLHTQ NTSLINQKKK LEGDISQMQN EVEESIQECR NAEEKAKKAI
1761    TDAAMMAEEL KKEQDTSAHL ERMKKNMEQT IKDLQKRLDE AEQIALKGGK KQIQKLESRV RELENELENE LRRNSDAQKG
1841    ARKFERRIKE VTYQSEEDKK NLARMQDLID KLQLKVKSYK HQAEEAEAQA NLYLSKYRKQ QHDLDDAEER AEIAESQVNK
1921    LRSKSRDIGM KKVHEEE
```

5.258 PREDICTED: similar to partner of PIX 1 [Gallus gallus]

Protein Accession	gi|118100298
Mean Expression Ratio	0.965
Median Expression Ratio	0.965
Credible Interval	(0.75, 1.25)
Associated Peptides	1
Associated Spectra	1
Coverage	0.0138

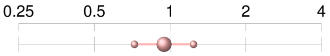

A	2.5	50	97.5	Sequence
**1**	**0.72**	**0.95**	**1.2**	**FLELFLGEFR**

```
1       MAAGGSGSSG GGGSAEEKNY RRFLELFLGE FRGPFGAEPA PAPPPPAADT VDTAASEEDP AAEGEAAAAE AEEGDSADPR
81      PPTPPPPPPP LPPLPRPLSE YITEEEVEGE CLDLCLQQLY KYNCPSFLAA ALARATSDEV LQSDLSAHYL PKHVDNADGI
161     IQIETVKLAR LVFSKLHEIC SNWVKEFPLQ PKPHRYYETS IHAIKNMRRK MEDKHVCIPD FNMLFNLEDQ EEQAYFAVFD
241     GHGGVDAAIY ASIHLHVNMV HQEMFQHDPA EALCRAFRVT DERFVQKAAR ESLRCGTTGV VTFIRGNMLH VAWLGDSQVM
321     LVRKGQAVEL MKPHKPDRED EKKRIEALGG CVVWFGAWRV NGSLSVSRAI GDAEHKPYIC GDADSASTVL DGSEDYLILA
401     CDGFYDTVNP DEAVKVVADH LKENNGDSSM VAHKLVASAR DAGSSDNITV IVVFLRDMNA AVSVSEESDW TENSFQGGQE
481     DNGEDKENHG ECKRPWPQHQ CSAPADLGYE GRVDSFTDRT SLSIGSGINP FDDQGYLDLT KTETSMPNSA KYLPPIQVFS
561     PGIPKNASLI NGLTVKNESP ESTSSICGQS NPREYSAPVS LHAAGQSIYR VKGLTPISFG LEDELFKSLG KQVAFFHFRF
641     CNGKRRRGAR LKPKFHTPLL AREPSHREGS GLPLPVRSRG STRLLVRHSP WWRLPRPNAY SESMFLMRRQ SHCIPDAYLH
721     QCCEM
```

5.259 PREDICTED: hypothetical protein [Gallus gallus]

Protein Accession	gi|50731161
Mean Expression Ratio	0.965
Median Expression Ratio	0.965
Credible Interval	(0.748, 1.25)
Associated Peptides	1
Associated Spectra	1
Coverage	0.0348

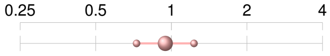

A	2.5	50	97.5	Sequence
**1**	**0.73**	**0.94**	**1.2**	**AYLVPAELLFR**

```
1     MAKVEKFAFH VPSLEELAGV LQKGLKENFA DAQVSVVDCP DLTQEPFNFP AKGICGKPRI ADVGGVPYLI PLAQKEKVYD
81    LNTVAKDIEL PGAFILGAGA ASSRVLGVNA ELIPIVQTKN EKKPAINGSY VAQINPADKG CLLEKYSNKY SDCEFGLLAN
161   LYASEGQPGK VVEVKANGRT GELNFVSCLR QILEKHYGEK PVGMGGTFII QKGKAKIHIM PPEFSACPLN TDEDVNNWLK
241   FFEMKAPLIC QPVIVSRDPG FDLRLEHTHC FSQHGEGGHY HTDTTPDAVQ YRAYLVPAEL LFRIDRPKET HSVGRD
```

5.260 PREDICTED: similar to Cxorf5 (71-7A) protein [Gallus gallus]

Protein Accession	gi|118084129
Mean Expression Ratio	0.964
Median Expression Ratio	0.965
Credible Interval	(0.746, 1.24)
Associated Peptides	1
Associated Spectra	1
Coverage	0.00988

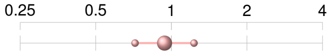

A	2.5	50	97.5	Sequence
**1**	**0.72**	**0.94**	**1.2**	**RIMQDTAASR**

```
1     MAEGQREALT QDELRKRLYQ TFKNRGVLDT LKTQLRKQLI HELMHPILSG ELQPRTVPSD DCSLLITASN SLVADHLQRC
81    GYEYSLSVFF PESGLEKKKL LSVQDLLQLL RISPKSSLYK SLTSGTQKEN KGFLMWILMG LTEHHLSKEC NDRETQTISI
161   PPYRESLAEK LQLIDEQFAD SYPQHHKYEP LEGKLHEYRK EIEKQLQAEM SQKLQHFKEV EVAKIKMEEK AQTQKEISEL
241   RYELERTHQA KAEALVSREK NAIERLQKQQ EIEAKEVYAQ RQSLLKDIEV MRTREAELKQ RIEAFEITQK LQEEKNKSID
321   DALRRREVAV KNIEETYDQK LKTELLKYQL ELKEEYIART NKVTEDEKKN KEKAMLLREE AVAVNSKKEE LKQAVSRTKE
401   LELDLESVKA QVLLVNKQNQ LLTEKLKEVS DYPLLKEEKL ELQVQNKLLR QQLDETRTEN QHLRDKLSQP SAEHLACQAE
481   LRKVEHSRRL VMDEFESHKQ FLEKQLQSEV ERSAQLKTQL LDSEATVRKL NVQVEDLKLQ LKQTQAALEN EVYRNPKPSL
561   VDRSVIDLID DRIVPHDVYT DSVFLKNPIV SDVVKGNAVP RAGYHQQLQT RSASPDSDLE CMAQTRARIK ELEKEAEYLE
641   EAYRNYQRRI MQDTAASRTP RKMQSPLLLQ CAVSGVPLTT QCELLEDNPR SQHSPLSSPR SEKYVGRHGQ TDAFKNPSTP
721   PHRGVPPSKC LSSTPVHKLE RVLSNKNRSD DASDSYLASS QRSVDQVLSP ISKPLILSRS ESLSSSPCSH REKIRLQKQR
801   IDNQDFIDSP KPKKLAYEDL EEHISPLEYQ GDIPEQCESD VLHPSGDTVN GNHVTATVRA TATSPQDLTV LDRRETEEQN
881   TEDQHWEERK AGEERREGEQ QGTGEREQNE VEELKNETLI QDSMKINEEE GDKGEKCESN NSGVEEIIKD STPNPLEKYM
961   KIIQQRREQE LAHEDSTKEE IRDVSFIEGI TSSEKDDSAA GISHGDDDDN FW
```

5.261 RNA binding protein with multiple splicing 2 [Gallus gallus]

Protein Accession	gi|45382377
Mean Expression Ratio	0.965
Median Expression Ratio	0.965
Credible Interval	(0.745, 1.24)
Associated Peptides	1
Associated Spectra	1
Coverage	0.05

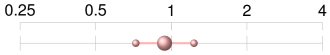

A	2.5	50	97.5	Sequence
**1**	**0.72**	**0.94**	**1.2**	**FDPENPQTLR**

```
1     MSNLNKDTEH TNGGGNVEEE VRTLFVSGLP VDIKPRELYL LFRPFKGYEG SLIKLTSKQP VGFVTFDSRA GAEAAKNALN
81    GIRFDPENPQ TLRLEFAKAN TKMAKSKLMA TPNPTNIHPA LGAHFIARDP YDLTGAALIP ASPEAWAPYP LYTTELTPAI
161   PHAAFTYPAA AAAAAALHAQ MRWYPPSEAT QQGWKSRQFC
```

5.262 PREDICTED: similar to Heat-shock protein 105 kDa (Heat shock 110 kDa protein) (Antigen NY-CO-25) [G

Protein Accession	gi|118084991
Mean Expression Ratio	0.966
Median Expression Ratio	0.965
Credible Interval	(0.749, 1.24)
Associated Peptides	1
Associated Spectra	1
Coverage	0.00952

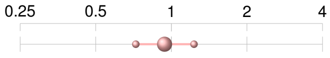

A	2.5	50	97.5	Sequence
1	0.72	0.94	1.2	KGDQPPEAK

```
1     MRTGDVTGFW RRRLVGIVRE RRRRRKRKPH VEPVSGCRSF WKLRLIVIFK AAAPAARPCA PIRRQWDRGG GGGAGSGLGG
81    GWSVGGGGAM AVVGFDLGFQ SCYIAVARAG GIETVANEFS DRCTPSVVSF GSKNRAIGVS AKNQQITHAH NTVSNFKRFH
161   GRAFNDPFVQ KEKEKLSYDL VPMKNGGVGV KVMYMDEEHI FSVEQISAML LTKLKETAES NLKKPVTDCV ISVPSFFTDA
241   ERRSVLDAAQ IVGLNCLRLM NDMTAVALNY GIYKQDLPAP EEKPRIVVFV DMGHSAFQVS ACAFNKSKLK VLGTAFDPFL
321   GGRNFDGKLV DYFCAEIKAK YKLDPKSKVR ALLRLYQECE KLKKLMSSNS TDIPLNIECF MNDTDVSGKM NRSQFEELCA
401   DLLQRIEMPL LSLMEQTQLK VEDVTAVEIV GGATRIPAVK ERIAKFFGKD VSTTLNADEA IARGCALQCA ILSPAFKVRE
481   FSVTDATPFP ISLLWNTEAE DTEGVHEVFS RNHAAPFSKV LTFYRKGPFE LEAFYSDPNG VPYPESKIGR YIIQNVAAQK
561   DGEKSKVKVK VRVNTHGIFS VSTASMVEPV KSEDSEDVGV ETELETQDQM PAENSSDKNN QQENSEAGTQ SQVQTDGQQT
641   SQSPPSSEPP SEENKIPDVK KTSEKKGDQP PEAKKPKIKV KNVELPIEAN LVWQLGKDLL NMYIETEGKM IMQDKLEKER
721   NDAKNAVEEY VYEFRDKLSG PYEKFVCEKD LQGFSALLTE TEGWLYEEGE DEAKQVYVDK LEDLKKLGTP IEMRYQEAEE
801   RPKLLEELGH RLQYYAAIAG EFRNKDEKYI HIDEMEMMKV EKCVSEVIEW MNNAVSAQAK KSLDQDPAVR SFEIKAKLQE
881   LNNVCEPIVT QPKPKVDSPK EENPLNEQGD YKTEDMGEDD KNSDMPQQNG ECHPGDQNTV NMDLD
```

5.263 heat shock 70kDa protein 8 [Gallus gallus]

Protein Accession	gi|45384370
Mean Expression Ratio	0.966
Median Expression Ratio	0.965
Credible Interval	(0.848, 1.10)
Associated Peptides	9
Associated Spectra	11
Coverage	0.206

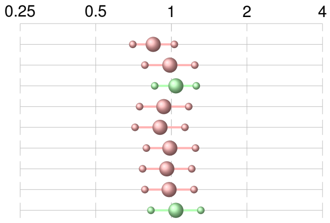

A	2.5	50	97.5	Sequence
2	0.7	0.85	1.0	QTQTFTTYSDNQPGVLIQVYEGER
1	0.78	0.98	1.2	FDDSVVQSDMK
2	0.86	1.0	1.3	TTPSYVAFTDTER
1	0.75	0.94	1.2	TVTNAVVTVPAYFNDSQR
1	0.72	0.9	1.1	DAGTIAGLNVLR
1	0.8	0.99	1.2	IINEPTAAAIAYGLDKK
1	0.77	0.96	1.2	YKAEDEK
1	0.78	0.98	1.2	LDKSQIHDIVLVGGSTR
1	0.83	1.0	1.3	NQTAEKEEFEHQQK

```
1     MSKGPAVGID LGTTYSCVGV FQHGKVEIIA NDQGNRTTPS YVAFTDTERL IGDAAKNQVA MNPTNTVFDA KRLIGRRFDD
81    SVVQSDMKHW PFTVVNDAGR PKVQVEYKGE TKSFYPEEIS SMVLTKMKEI AEAYLGKTVT NAVVTVPAYF NDSQRQGTKD
161   AGTIAGLNVL RIINEPTAAA IAYGLDKKVG AERNLLIFDL GGGTFDVSIL TIENGIFEVK STAGDTHLGG EDFDNRLVNH
241   FIAEFKRKHK KDISENKRAV RRLRTACERA KRTLSSSTQA SIEIDSLYEG IDFYTSITRA RFEKLNADLF RGTLDPVEKA
321   LRDAKLDKSQ IHDIVLVGGS TRIPKIQKLL QDFFNGKELN KSINPDEAVA YGAAVQAAIL SGDKSENVQD LLLLDVTPLS
401   LGIETAGGVM TVLIKRNTTI PTKQTQTFTT YSDNQPGVLI QVYEGERAMT KDNNLLGKFE LTGIPPAPRG VPQIEVTFDI
481   DANGILNVSA VDKSTGKENK ITITNDKGRL SKEDIERMVQ EAEKYKAEDE KQRDKVSSKN SLDSYAFNMK ATVEDEKLPG
561   KILDEDHQNI LDKCNEIINW LDKNQTAEKE EFEHQQKELE KVCNPIITKL YQSAGGMPGG MPGGFPGGGA PPSGGASSGP
641   TIEEVD
```

5.264 hemoglobin, gamma A [Gallus gallus]

Protein Accession	gi|126165290
Mean Expression Ratio	0.966
Median Expression Ratio	0.966
Credible Interval	(0.805, 1.16)
Associated Peptides	3
Associated Spectra	5
Coverage	0.333

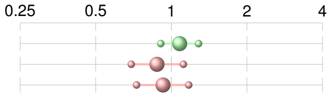

A	2.5	50	97.5	Sequence
3	0.9	1.1	1.3	VNVEECGAEALAR
1	0.69	0.88	1.1	FFASFGNLSSPTAIMGNPR
1	0.73	0.93	1.2	LSELHCDKLHVDPENFR

1 MVHWSAEEKQ LITSVWSKVN VEECGAEALA RLLIVYPWTQ RFFASFGNLS SPTAIMGNPR VRAHGKKVLS SFGEAVKNLD
81 NIKNTYAKLS ELHCDKLHVD PENFRLLGDI LIIVLASHFA RDFTPACQFA WQKLVNVVAH ALARKYH

5.265 hect domain and RLD 3 [Gallus gallus]

Protein Accession	gi|86129504
Mean Expression Ratio	0.967
Median Expression Ratio	0.966
Credible Interval	(0.748, 1.25)
Associated Peptides	1
Associated Spectra	1
Coverage	0.0105

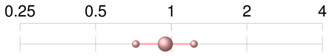

A	2.5	50	97.5	Sequence
**1**	**0.72**	**0.95**	**1.2**	**ELSIHSDIDLK**

1 MLCWGYSSFG QPGIGSNLQV IIPEPQVYGF IHDRNVKEVA CGGNHSVFLL EDGEVYTCGL NTKGQLGHES EGSKPEPIGA
81 LAGQHIVHVA CGESHSVALS DQGQVFSWGA GSDGQLGLTT IEDAVTVPRL IKKLNQQTIL QISCGNWHCL ALAADGQFFT
161 WGQNSYGQLG LGKECPSQAS PQRVKSLDGI PLAQVAAGGA HSFALSLSGA VFGWGKNSSG QLGLSDERDR ESPCHVKLLR
241 SQKVVYISCG EEHTAVLTKS GGVFTFGAGS CGQLGHDSMN DEVNPRRVLE LMGSEVSQIA CGRHHTLAFV PSSGMIYAFG
321 CGTRGQLGTG HTCNVKCPSP VKGHWAAHNG QLSGKPDACK YHIVKHIFSG GDQTFVLCSK YENSLPADDF RTINGTRYTC
401 LINDETIDVW RQKLLEKNSS NSVNNVVQIL SSAACWNGSF LEKKIDEHFK TSPKIPGIDL NSTRVLFEKL MNSQHSILLD
481 QILKSFESFL IPQLSSSPPD VEAMRIYLIL PEFPPFQDSK YYITLTLPLA MAILRLDTNP SKVLDNWWSQ VCPRYFLRLV
561 DLYKGAVVYL LSGRKTLLIP VLFSSYITAA LRLLEKLHKV NQKVKHIEYD KFYIPEISSL VDIQEDYLMW FLHQAGMKVR
641 PSIMQDAVTL CSYPFIFDAQ AKTKMLQTDA ELQMQVAING ANLQNVFMLL TLEPLLARSP FLVLHVRRSN LVGDALRELS
721 IHSDIDLKKP LKVIFDGEEA VDAGGVTKEF FLLLLKELLN PIYGMFTYYP ESNLLWFSDT CFVEHNWFHL IGIICGLAIY
801 NFTVVDLHFP LALYKKLLNV KPCLEDLKEL SPTEGRSLQQ LLDYPGEDIE ETFCLNFTIC RESYGVTEHK NLIEDGDKIQ
881 VQKDNREKFV EAYVNYIFNC SIHEWYTAFS TGFLKVCGGK VLELFQPTEL RAMIVGNSNY NWEELEESAV YKGDYTATHP
961 TVRMFWETFH AFPLEKKKKF LLFLTGSDRI PIYGMSSLRI VIQSTANGEQ YLPVAHTCYN LLDLPKYSSK EILSARLMQA
1041 IDHYEGFSLA

5.266 cytokine induced apoptosis inhibitor 1 [Gallus gallus]

Protein Accession	gi|57524844
Mean Expression Ratio	1.03
Median Expression Ratio	1.04
Credible Interval	(0.8, 1.34)
Associated Peptides	1
Associated Spectra	1
Coverage	0.049

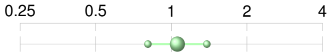

A	2.5	50	97.5	Sequence
**1**	**0.8**	**1.1**	**1.4**	**GLVDAVQASVGADSR**

1 MGEYGIAPGQ RVAVIWDSSS PVEALKGLVD AVQASVGADS RVSVENINQL CQSAHRESSF DVILSGVVPG STAQHSAEVL
81 AEIARILKPG GRVLLKEPVV TESENNSQIK TAAKLPAALT LSGLVEVKGL QKEPLTAEEA QSVREHLGYQ GNDLLIVQIE
161 GRKPNFEVGS SSQLKLSFAK KTSPSGKPSV DPATAKLWTL SASDMNDEEM DLLDSDELLD SEDLKKPDPA SLRAPSCKEK
241 GKKKACKNCT CGLAEELEQE KKSSQPKSAC GNCYLGDAFR CASCPYLGDA CLQAWREDPA EREPAA

5.267 troponin I, skeletal, fast [Gallus gallus]

Protein Accession	gi|45382253
Mean Expression Ratio	0.967
Median Expression Ratio	0.966
Credible Interval	(0.753, 1.26)
Associated Peptides	1
Associated Spectra	1
Coverage	0.0492

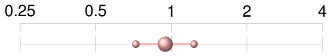

A	2.5	50	97.5	Sequence
**1**	**0.72**	**0.94**	**1.2**	**EAAAKEVEK**

```
1       MSDEEKKRRA ATARRQHLKS AMLQLAVTEI EKEAAAKEVE KQNYLAEHCP PLSLPGSMQE LQELCKKLHA KIDSVDEERY
81      DTEVKLQKTN KELEDLSQKL FDLRGKFKRP PLRRVRMSAD AMLRALLGSK HKVNMDLRAN LKQVKKEDTE KEKDLRDVGD
161     WRKNIEEKSG MEGRKKMFEA GES
```

5.268 PREDICTED: hypothetical protein [Gallus gallus]

Protein Accession	gi|118103330
Mean Expression Ratio	1.03
Median Expression Ratio	1.03
Credible Interval	(0.802, 1.34)
Associated Peptides	1
Associated Spectra	1
Coverage	0.0181

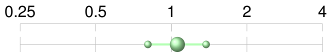

A	2.5	50	97.5	Sequence
**1**	**0.8**	**1.1**	**1.4**	**DNTVKMTLLRR**

```
1       MAVRVPAGGE QLRGDGAELE GARRAPLGRS RFLKVCSGEA RGHQRGPAAR GGEATSALLS AAHARSNSAL RKVAQLQSKI
81      LDRKKQLELQ STELGRKPLD EDSSSASSLE HHTRGKKYLK GCAAVGRNVA ASRGCSEEEE STQSPKRNVT VTQQLGLGTY
161     EKPMREFMEN SLEFSSGREN QRCVTTDSRW GGKRETPVAP GTPPPSHKEI PLTEVSAVPS LHSKGSEKNI LGGSNLLRPS
241     PASRNRTARH DMRSQSLSSS MKDNTVKMTL LRRGNAKQSQ VSNGSGGSEI KSLDDLFSKA DDVEDSTSIS SNDFRQNILS
321     LDDLASGISE MAELKQQGTD IQISRETNRN PKKDTFLLEK DQTFPKISAE IDATDTSERD TENVTEAEIP EHLEVSADFS
401     RPRQDYPDQD DRTVNSEYSE DFEQSPSTTD KETVSKMSEE HSESYAYSGK DPSSSASSPL LTRERHKRIH RVAVKETAAQ
481     TVDLPFTYCW SKTNSSAVLG LPVGNSYVDP VPIASHVISM DTVEALTAYN PSVLVLNAML KQHLMLTQQF VENIQHLHLS
561     LVESLENEKF HYHTLEEAKE YIKNHRSPPL TIEQAFEEIQ RAEEILPSS
```

5.269 ras homolog gene family, member C [Gallus gallus]

Protein Accession	gi|71143154
Mean Expression Ratio	0.967
Median Expression Ratio	0.967
Credible Interval	(0.81, 1.15)
Associated Peptides	3
Associated Spectra	6
Coverage	0.150

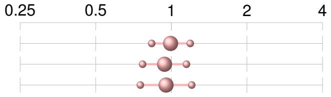

A	2.5	50	97.5	Sequence
**3**	**0.84**	**1**	**1.2**	**QVELALWDTAGQEDYDR**
**2**	**0.77**	**0.94**	**1.1**	**QEPVKPEEGR**
**1**	**0.75**	**0.95**	**1.2**	**MKQEPVKPEEGR**

```
1       MAAIRKKLVI VGDGACGKTC LLIVFSKDQF PEVYVPTVFE NYIADIEVDG KQVELALWDT AGQEDYDRLR PLSYPDTDVI
81      LMCFSIDSPD SLENIPEKWT PEVKHFCPNV PIILVGNKKD LRNDEHTRRE LAKMKQEPVK PEEGRDMANR INAFGYLECS
161     AKTKEGVREV FEMATRAGLQ VRKNKKRRGC PLL
```

5.270 PREDICTED: hypothetical protein [Gallus gallus]

Protein Accession	gi|118097545
Mean Expression Ratio	0.967
Median Expression Ratio	0.967
Credible Interval	(0.744, 1.26)
Associated Peptides	1
Associated Spectra	1
Coverage	0.0358

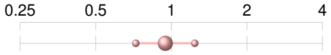

A	2.5	50	97.5	Sequence
**1**	**0.72**	**0.94**	**1.2**	**EDSAKPGAHAK**

```
1       MENSQLCKLF IGGLNVQTTE AGLREHFAAY GTLTDCVVVL NPQTKRSRCF GFVTYSAVEE ADAAMAASPH AVDGNSVELK
81      RAVSREDSAK PGAHAKVKKL FVGGLKGDVG EGDLVQHFSQ FGPVEKAEII ADKQSGKKRG FGFVYFQNHD AADKAAVVKF
161     HPIQGHRVEV KKAVPKEDIQ AGGGGSARPS RGGRGGGRGR GGGGSGNRDH NGLSKGGGGY NSYGGYGGGG GGGYGGYGGG
241     SYGGGGGGGD YGNGYGGFGS YSQHQSSYGP MKSGGGGGGG GGNWGGRSNS GPYRGGYGGG GYGGGSF
```

5.271 Rous sarcoma virus transcription enhancer factor II [Gallus gallus]

Protein Accession	gi|45382293
Mean Expression Ratio	0.968
Median Expression Ratio	0.967
Credible Interval	(0.752, 1.25)
Associated Peptides	1
Associated Spectra	1
Coverage	0.0638

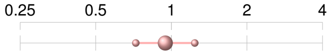

A	2.5	50	97.5	Sequence
**1**	**0.72**	**0.95**	**1.2**	**GAEAANVTGPDGVPVEGSR**

```
1       MSEAAETAPL AHGARQLPPR RRRRPLLPPS GRQAQEPRQR AAPGASAARW HAAPELRRRG GTPASAPGPL PPCRRRRGDG
81      EEVLATKVLG TVKWFNVRNG YGFINRNDTK EDVFVHQTAI KKNNPRKYLA SVGDGETVEF DVVEGEKGAE AANVTGPDGV
161     PVEGSRYAAD RRRYRRGYFG RRRGPPRSGV EGEIKDGVTE GGQIHQQVQR NPTYRPRYRR GPLRPRPAPV AGEAENKENQ
241     HEAGAVSQQP LRRGYRRPYN YRRRPRSPNT PAQDGKETKV TETPAENPAP VTEQSGAE
```

5.272 calpastatin [Gallus gallus]

Protein Accession	gi|145411437
Mean Expression Ratio	0.967
Median Expression Ratio	0.967
Credible Interval	(0.746, 1.25)
Associated Peptides	1
Associated Spectra	1
Coverage	0.0120

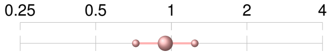

A	2.5	50	97.5	Sequence
**1**	**0.72**	**0.94**	**1.2**	**KPSAPASTK**

```
1       TSSKSGEKKE ATEKPAGANV SQLPKTQTSG VLPAAKKQSP SADAPKPPIM KPSEAKSTPT IQTEPEKTAL STKPSNSQNN
81      QPAEEKPKES SGAKSPIAPS VVTASKPNNM GKETTSQAER PSQATSADTA QPKTEKTSTA GGAGAAGAGA VASMVAAADK
161     PNSEPAMDES ALDSLIDTLG GSEEDVATRP VYTGPEITEN ISSEYLEELG KREGSLPPEY LALLKSKVDG KDGGLPKVDE
241     HSEKPMTDDE LADALSSDFT CSIAAAEEKK TTEKQTKEGE IIQAQVTSSV KTSVPPKEKK TKSKEEIKED AMEALMATIG
321     GPEPEPEPKD VSPIVEVSEA KAKEKKEKKA GERDDTIPPE YRLTPELDKD GKPVLPKPEE KPKPLSESDL VDEFSKDFAS
401     PAQPAIQSKP SKPSNTSKKP SAPASTKTAK DEVVPRATAC SVQSSAPTPV SSVGHVADEA VEALSSSLGE REPDPEEKKP
481     AVDKVKEKAK RKQHKKLGED EETIPPEYRL TDAKDKDGRP LLIKPEEESQ PMSENDLLEG LTKGFSPAQS APLPTQTVKK
561     KTKGGKKTAD SSDVISASTI SSVHSAAPLA STSGGKEMDD ALDLLSDSLG QREPDPDENK PVVDKVKEKA KSEHRDKLGE
641     RDDTIPPDYR KLLESSDEGK PVKPTPKDSS KNKEQKKPTD ESAAIDALSG DFDTSPKAPA TPQHSKEKSG KETVTTKATP
721     KDERKPKDHK KARGQSSSSK SEKQKTS
```

5.273 heat shock 70kDa protein 4-like [Gallus gallus]

Protein Accession	gi|60302800
Mean Expression Ratio	0.966
Median Expression Ratio	0.967
Credible Interval	(0.747, 1.24)
Associated Peptides	1
Associated Spectra	1
Coverage	0.0142

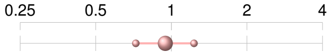

A	2.5	50	97.5	Sequence
**1**	**0.72**	**0.94**	**1.2**	**SIDLPIQASLYR**

```
1       MSVVGIDLGF LNCYYGVARS GGIETIANEY SDRCTPACIS LGSQTRAIGN AAKSQIVTNV KNTLHGFEKL HGRAFEDSYI
81      QAERAKLPYE LQKMPNGSVG VKVRYLDEER LFAVEQITGM LLAKLKETSE SALKKPVADC VISVPSFFTD AERRSVMAAA
161     QIAGLNCLKL MNETTAVALA YGIYKQDLPA LEEKPRNVVF VDMGHSAYQV SICAFNKGKL KVLATTFDPF LGGRNFDEAL
241     VDYFSEEFRT KYKLNVKENP RALLRLYQEC EKLKKLMSAN ASDLPLNIEC FMNDLDVSSK MNRAQFEQLC AALLSRVEPP
321     LRAAMEQAKL QREDIYSIEI VGGATRIPAV KEQISSFFCK EISTTLNADE AVARGCALQC AILSPAFKVR EFSITDVVPY
401     SITLRWKSSY EEGTGECEVF SKNHAAPFSK VITFHKKEPF DLEAFYTHPH EVPYPDSRIG RFTIQNVGPQ HDGDNSKVKV
481     KVRVNIHGLF SVANASIIEK QNIDGDHNDA AMDTESSSKN QGREDELDKM QVDQDEGVQK SQAEQQSQAD EEAENTGIET
561     KASSGDKQDH PTLPRAKTKV KSIDLPIQAS LYRQLGQDLI NCYIENEGKM MMQDKLEKER NDAKNAVEEY VYDFRDKLCG
641     VFEKFITEED TNKLTLMLED TENWLYEDGE DQPKQVYMDK LQELRKFGQP IQERYMEHEE RPKVLNELGK KIQLLMKAVE
721     AYKNKDEKYD HLDPAEMEKV EKYISEAMNW LNTKMNAQNK LSLTQDPVVK VAEIISKSKE LDSFCNPIIY KPKPKIEPPN
801     DGQSKANGEH NGPVNGQSST ETGPDPAKDN SQQTKPPGEM EVD
```

5.274 PREDICTED: hypothetical protein [Gallus gallus]

Protein Accession	gi|118103387
Mean Expression Ratio	0.965
Median Expression Ratio	0.967
Credible Interval	(0.747, 1.25)
Associated Peptides	1
Associated Spectra	1
Coverage	0.0255

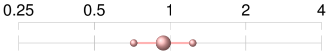

A	2.5	50	97.5	Sequence
1	0.72	0.94	1.2	ETEAQAEAQEVEPGAGSR

```
1      MVANVLTVME TGTLGQTEAR YESYNYGYGY GQDNSGNYGY GMAASNSWDM GNSDMDMNPD GAGSADTVIA KMNQRLDMVS
81     HLDADSMQGG HYGSGGDRYD SYESYDSRSS MNDRDVYRSG YDYNENDNDN AYDSHYDGHY DDHYESHYDS FYGSRRDQYQ
161    HRARDGFGQR GQNWARDGRN TRPMASPYSG RMGGQWNDAP RGMGPHGSSR LPSLFSHNII PELGMFQGMR GFSGSMRFGG
241    GMMKQRMRRN WKMWDSDFKP QKKKMKKDLT GKKRKQTSSS DEPDSKAAKT DGSDNSDSDN EEGTEGESGE KEEKEGSRGE
321    GEDEEGRDSE KGALTIQEEI SQIKRKLQAG KKTQERQKKR HRDRMVERIQ YVCSLCKYRT FYDDEMNSHL ESKFHKEHFK
401    FVGTKLPQQT ADFLQEYVAN KTRKTEERRK AIEDINAVIQ QIYRDQDLTQ DIGMEHFIKK VEAAHCAACD LFIPMQYGII
481    QKHLKSLDHN HNRRAMMEQS KKSSLVVARS ILNNKLISKK LERYLKGENP FTDDPEEKEE HEEGEGGVAG NVEEGTAEGG
561    DENKDEEENL EEENADDENK EENVGDENKE EGNLDNENKG DEMLDDENKE EGNLRDESND PKENPEGNEN EEEEEKGTER
641    ETEAQAEAQE VEPGAGSRGG EEEEEEEEVW QPAGESLPED EEQQPAEGEE EEESEETTAA PEDEDVA
```

5.275 PREDICTED: similar to natural killer cell enhancing factor isoform 3 [Gallus gallus]

Protein Accession	gi|118094468
Mean Expression Ratio	0.967
Median Expression Ratio	0.967
Credible Interval	(0.809, 1.16)
Associated Peptides	4
Associated Spectra	4
Coverage	0.179

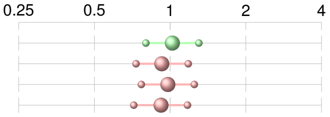

A	2.5	50	97.5	Sequence
1	0.8	1.0	1.3	QITINDLPVGR
1	0.73	0.93	1.2	LVQAFQFTDK
1	0.77	0.98	1.2	KQGGLGTMK
1	0.72	0.92	1.2	SKEYFSK

```
1      MSSGKAFIGK PAPDFTATAV MPDGQFKDIK LSDYRGKYVV FFFYPLDFTF VCPTEIIAYS DRADEFKKIN CEIIGASVDS
81     HFCHLAWINT PKKQGGLGTM KIPLVSDTKR VIAKDYGVLK EDEGIAYRGL FIIDEKGILR QITINDLPVG RSVDETLRLV
161    QAFQFTDKHG EGLHVLVVSV CPAGWKPGSD TIKPDVQKSK EYFSKQK
```

5.276 PREDICTED: similar to KIBRA protein [Gallus gallus]

Protein Accession	gi|118097238
Mean Expression Ratio	0.967
Median Expression Ratio	0.967
Credible Interval	(0.746, 1.25)
Associated Peptides	1
Associated Spectra	1
Coverage	0.0098

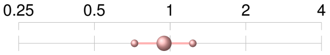

A	2.5	50	97.5	Sequence
1	0.72	0.94	1.2	EKQDLIQSLAR

```
1      MPRKELPLPA GWEEARDYDG KVYYIDHGSR TTSWIDPRDR YTKPLTFADC ISDELPLGWE EAYDPQVGVY YIDHNTKTTQ
81     IEDPRVQWRR EQEHMLKDYL VLAQEAIAAQ KEIYQVKQQR LELAQQEYRQ LHDVWEHKLG SQTSLLSGSS SSSKYDPEIL
161    KAEIATTKSR VNKLKREVAH MKQEIQYKEH GFQTLKNIDM KMSDTQGGYK LDEAQAILSE MKALKKAITS GEKEKQDLIQ
241    SLARLKDSFV NDRGSQSDLW ASSVSLESSS PSLPRQYLDV SSQTDVSGNV SINSNNQLAE KVRLRLRYEE AKRRIGNLKI
321    QLAKLDSEAW PGVLDSERDR LILINEKEEL LKEMRFISPR KWTRGEVERL ETERKRLEED LQAARDTQSK ALTERLKLNS
401    KRNQLVRELE ETTRLVAMLH TQLKSLSASM LSLSSSSSPG SLASSRGSLA TSSQDSSTSA SFTDLYCEHM EQLEQLEQLD
481    SDYQNKLDLL LEGATGFRPS GCITTIHENE VAKTHKTDAT SRIQALRSLS GTPKSMTSLS PRSSLSSPSP PCSPLVIDPL
561    LGGDAFSSHM DFDDTEISTN LSELTLNIES GNCRLEEPRA GDKHLDQGVN TAGGTALKVA CVSAAVFDES VAGDSGVYEA
641    SVQRPCASEV MVFDSDDTEA AGTAKVQIAM RYDEKNKQFA ILVIQLSNVQ ALLLQQDQKV NIRVAVLPCL ESTSCLFRTR
721    PLEVSDNLMY NEVFWVSISY PALRQKTLRV DVCTVDKSRL EECLGGAQIS LAEICRSGEK STRWYNLLSS KYLQKQNRKS
801    KQGTIHSEIA CTDKTDSVSA LLEQTAVELE AVEKKLEESR TTLTSGESWR DEEVAEQEEL DEISENEAEE EEEFCAGKLL
```

```
881     WETEESLNSH PHVEMAVKVD KETNTESLAQ SSAVVRPKDK RAANPPQTQF VRGSTIIRSK TFSPGPQSQY VCRLNRSDSD
961     SSTLSKKTPF VRNAMERRSV RVKRPSIKSA GTERLIRTSL DLELDLQASK TWHDRLVQEI SVLRELKEQL EQAQSQGEKE
1041    LPQWVKDDER FRLLLRLVEK RVEKTEHRCE LKADKMMRAA AKDVHRLRGQ SRKEPLEVQS FREKMAFFTR PRINIPTLSA
1121    DDV
```

5.277 adducin 1 (alpha) [Gallus gallus]

Protein Accession	gi|119331100
Mean Expression Ratio	0.967
Median Expression Ratio	0.967
Credible Interval	(0.75, 1.25)
Associated Peptides	1
Associated Spectra	1
Coverage	0.0164

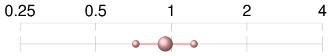

A	2.5	50	97.5	Sequence
1	0.72	0.94	1.2	AEEETQQDQTYR

```
1       MNGDSGVAVV TSPPPTTAPH KERYFDRVDE NNPDYLRERN MAPDLRQDFN MMEQKKRVSM ILQSPAFCEE LESMIQEQFK
81      KGKNPTGLLA LQQIADFMTT NVPNVYPAAP QGGMAALNMS LGMVTPVNDL RGSDSIAYEK GEKLLRCKLA AFYRLADLFG
161     WSQLIYNHIT ARVNSEQEHF LIVPFGLLYS EVTASSLVKI NIQGDVVDRG STNLGVNQAG FTLHSAIYAA RPDVKCIVHI
241     HTPAGAAVSA MKCGLLPISP EALSLGEVAY HDYHGILVDD EEKVVIQKNL GPKSKVLILR NHGLVSVGET VEEAFYYIHN
321     LVLACEIQVR TLASAGGPDN LVLLDPGKYK AKSRSSESPS GDGSVSHPKW QIGEQEFEAL MRMLDNLGYR TGYPYRCPAL
401     REKSKKYSDV EIPASVTGYS FTSDGESGTC SPLRHSFQKQ QREKTRWLNS GRGDDASEEG QNGGSPKSKT KWTKEDGHRT
481     ATSAVPNLFV PLNTNPKEVQ EMRNKIREQN LQDIKTAGPQ SQVLSGVVVD RSLVQGELVT ASKAIIEKEY QPKVIVSTTG
561     PNPFNKLTDR ELEEYRKEVE RKQKGSEEPS EDGRQQKERS PPEHTSACTP PSTPMKAEEE TQQDQTYRDD SDAATLKQTL
641     PDLTPDEPSE ALSFPPLGKE EGRCDEDVSK SQTESPAAAD KEPPPQPAEE AATPTAEEGT AADAGSDESP GKSPSKEKEV
721     SHSFLPEEEQ KEE
```

5.278 Fructose-bisphosphate aldolase C (Brain-type aldolase)

Protein Accession	gi|1703245
Mean Expression Ratio	0.967
Median Expression Ratio	0.967
Credible Interval	(0.784, 1.19)
Associated Peptides	2
Associated Spectra	3
Coverage	0.27

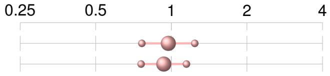

A	2.5	50	97.5	Sequence
1	0.76	0.97	1.2	YEGSGDDSGAAGQSLYVANHAY
2	0.76	0.93	1.2	YSPEEIAMATVTALR

```
1       LLKPNMVTPG HSCPTKYSPE EIAMATVTAL RRTVPPAVPG VTFLSGGQSE EEASINLNAI NTCPLVRPWA LTFSYGRALQ
81      ASALSAWRGQ RDNANAATEE FVKRAEVNGL AALGKYEGSG DDSGAAGQSL YVANHAY
```

5.279 PREDICTED: hypothetical protein [Gallus gallus]

Protein Accession	gi|50749392
Mean Expression Ratio	0.968
Median Expression Ratio	0.967
Credible Interval	(0.749, 1.25)
Associated Peptides	1
Associated Spectra	1
Coverage	0.00872

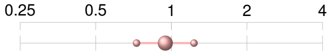

A	2.5	50	97.5	Sequence
1	0.72	0.95	1.2	EGNMEESAIR

```
1       MSVNQHTHAG PPYGQPQPGY QGYQQPAYGG QPLPGVPHTQ YGAYNGPMPG YQQPVPPQGY FPSLGFPSKG SPVSLNSDTS
81      LAPNFIGSVR ALPTSGAPPP ASGTSLPSGH QGYSQFGQGD VQNGIPTSTA PMQRPPASQP FLPGSAPAPV SQPSTFQQYG
161     PPPCSVQQLS NHMAGMTIGS TSVSAPPPAG LGYGPPTSVP PVSGSFSATG SGLYTPYTAS PGPPPPSVPQ GLPLAQPPFS
241     GQPVPTQRLP TEVPGFAPPP PATGIGASSY PPPTGAPRPP PMPGPPLSGQ TVAGPPMSQP NHVSSPPPPL TLSGPHPGPP
321     MSGPPPPTHP PQPGYQMQQN GSFGQVRGPQ PNYGGAYPGT PNYGSQPGPP PKRLDPDSIP SPIQVIEDDR NNRGSEPFVT
401     GVRGQVPPLV TTNFLVKDQG NASPRYIRCT SYNIPCTSDM AKQSQVPLAA VIKPLATLPP EETLPYLVDH GESGPVRCNR
481     CKAYMCPFMQ FIEGGRRFQC CFCSCVTEVP AHYFQHLDHT GKRVDFYDRP ELSLGSYEFL ATVDYCKNNK FPSPPAFIFM
561     IDVSYNAVKS GLVRLICEEL KSILDYLPRE GNMEESAIRV GFVTYNKVLH FYNVKSSLAQ PQMMVVSDVA DMFVPLLDGF
641     LVNVNESRTV ITSLLDQIPE MFADTRETET VFGPVIQAGL EALKAAECAG KLFIFHTSLP IAEAPGKLKN RDDKKLINTD
721     KEKTLFQPQT SFYSNLAKDC VAQGCCVDLF LFPNQYLDVA TLGVVTYQTG GSIYKYAYFQ LEADQDRFLN DLRRDVQKEV
801     GFDAVMRVRT STGIRATDFF GAFYMSNTTD VEMAGLDCDK TITVEFKHDD KLSEDSGALL QCALLYTSCA GQRRLRIHNL
881     SLNCCTQLAD LYRNCETDTL INYLAKYAYR GVLNSPVKSV RDSLINQCAQ ILACYRKNCA SPSSAGQLIL PECMKLLPVY
961     LNCVLKSDVL QPGPEVTTDD RAYIRQLVTS MDVAETNVFF YPRLLPLTKA DVDSDSLPAA IRNSEERLSK GDIYLLENGL
1041    NIFVWVGVNV QQGLIQNLFG VSSFSQISST LSTLPVLENP FSKKVRSIID MLHLQRSRYM KLIIVKQEDK LEMLFKHFLV
1121    EDKSLTGGAS YVDFLCHMHK EIRQLLS
```

5.280 PREDICTED: similar to Misato homolog 1 (Drosophila) [Gallus gallus]

Protein Accession	gi|118102188
Mean Expression Ratio	0.97
Median Expression Ratio	0.968
Credible Interval	(0.751, 1.26)
Associated Peptides	1
Associated Spectra	1
Coverage	0.0295

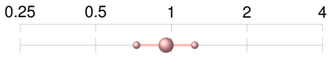

A	2.5	50	97.5	Sequence
**1**	**0.73**	**0.95**	**1.2**	**GDVANYAEPGTGTALR**

```
1       MAGEVVTLQL GHYAGCVGAH WWGLQRCPPE GTELSHAALL RAGRDGCTPR LIALELKGGV GPLGRGGARP EPLAAWRGDV
81      ANYAEPGTGT ALRDRGQRPG DASADTDCGS GAPGEGAAPS AGSSTQLWSD YLSVQLHPRS IYVLQHYNHD GDSGRLEAFG
161     QGEKLLQDAG CMEELEDRLH FYAEECDYLQ GFQVLCDLHD GFSGVGAKVT EVLQDEYSRK GILTWGLTPV THTVADSQKN
241     FYRVLNAALG IVHLSAHSSL FCPLSLSGSL GIKPQPPIEF PYVNYDASLN YHSSAVLAAA LDTLTVPYRL CSSRGSMMHL
321     AEMLSFSGRK VVAAWAAVPF PAVRGHSLPD ALCAHQQDVP WKLLSSCKEQ KVSRCFAQSV VLRGVCKESP TSCPGQQPPS
401     PLHACETAEQ VLQYYLHTAF PGAFSASHVL EQPCVTLPPY PRIFSPLLSR QGFLPDDLSS YSAAAVESIP VLTSLQSSPV
481     LRTLLCDLCK DLQKLGTRRC ASFFAAGVEE DDFHEALQEL RTLSQCYQMG FEADNSEDES DSD
```

5.281 calcium/calmodulin-dependent protein kinase (CaM kinase) II delta [Gallus gallus]

Protein Accession	gi|86129508
Mean Expression Ratio	0.968
Median Expression Ratio	0.968
Credible Interval	(0.748, 1.25)
Associated Peptides	1
Associated Spectra	1
Coverage	0.0334

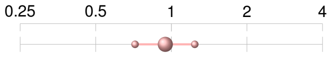

A	2.5	50	97.5	Sequence
**1**	**0.72**	**0.95**	**1.2**	**ESTESSNTTIEDEDVK**

```
1       MASTATCTRF TDEYQLFEEL GKGAFSVVRR CMKITTGQEY AAKIINTKKL SARDHQKLER EARICRLLKH PNIVRLHDSI
81      SEEGFHYLVF DLVTGGELFE DIVAREYYSE ADASHCIQQI LESVNHCHLN GIVHRDLKPE NLLLASKSKG AAVKLADFGL
161     AIEVQGEQQA WFGFAGTPGY LSPEVLRKDP YGKPVDMWAC GVILYILLVG YPPFWDEDQH RLYQQIKAGA YDFPSPEWDT
241     VTPEAKDLIN KMLTINPAKR ITASEALKHP WICQRSTVAS MMHRQETVDC LKKFNARRKL KGAILTTMLA TRNFSAAKSL
321     LKKPDGVKES TESSNTTIED EDVKARKQEI IKVTEQLIEA INNGDFEAYT KICDPGLTSF EPEALGNLVE GMDFHRFYFE
401     NALSKSNKPI HTIILNPHVH LVGDDAACIA YIRLTQYMDG TGMPKTMQSE ETRVWHRRDG KWQNVHFHRS GSPTVPINA
```

5.282 heterogeneous nuclear ribonucleoprotein R [Gallus gallus]

Protein Accession	gi|132626770
Mean Expression Ratio	0.968
Median Expression Ratio	0.968
Credible Interval	(0.753, 1.25)
Associated Peptides	1
Associated Spectra	1
Coverage	0.0174

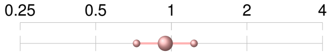

A	2.5	50	97.5	Sequence
1	0.73	0.95	1.2	KADGYNQPDSK

```
1      MANQVNGNAV QLKEEEEPMD TSSVTHTEHY KTLIEAGLPQ KVAERLDEIF QTGLVAYVDL DERAIDALRE FNEEGALSVL
81     QQFKESDLSH VQNKSAFLCG VMKTYRQREK QGSKVQESTK GPDEAKIKAL LERTGYTLDV TTGQRKYGGP PPDTVYSGVQ
161    PGIGTEVFVG KIPRDLYEDE LVPLFEKAGP IWDLRLMMDP LSGQNRGYAF ITFCSKDAAQ EAVKLCDNYE IRPGKHLGVC
241    ISVANNRLFV GSIPKNKTKE NILEEFNKVT EGLVDVILYH QPDDKKKNRG FCFLEYEDHK SAAQARRRLM SGKVKVWGNV
321    VTVEWADPVE EPDPEVMAKV KVLFVRNLAT TVTEEILEKS FSEFGKLERV KKLKDYAFVH FEDRGAAVKA MNEMNGKEIE
401    GEEIEIVLAK PPDKKRKERQ AARQASRSTA YEDYYYYPPP RMPPPIRGRG RGGRGGYGYP PDYYGYEDYY DDYYGYDYHD
481    YRGGYEDPYY GYDDGYAIRG RGGGGRGGRG APPPPRGRGA PPPRGRAGYS QRGAPMGPPR GARGGRGGPA QQQRGRGARG
561    ARGNRGGNVG GKRKADGYNQ PDSKRRQTNN QQNWGSQPIA QQPLQQGGDY AGNYGYNNDN QEFYQDTYGQ QWK
```

5.283 dynein, cytoplasmic, intermediate polypeptide 2 [Gallus gallus]

Protein Accession	gi|57529758
Mean Expression Ratio	0.968
Median Expression Ratio	0.968
Credible Interval	(0.778, 1.20)
Associated Peptides	2
Associated Spectra	2
Coverage	0.0509

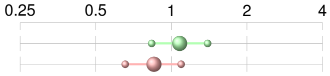

A	2.5	50	97.5	Sequence
1	0.83	1.1	1.4	SVSTPSEAGSQDSGDGAVGSR
1	0.66	0.85	1.1	ADAEEEAATR

```
1      MSDKSELKAE LERKKQRLAQ IREEKKRKEE ERKKKETDQK KDVLPVQEES DLEKKRREAE ALLQSMGLTP ESPVVPPPTS
81     PSSKSVSTPS EAGSQDSGDG AVGSRRGPVK LGMAKITQVD FPPREIVTYT KETQTPVMTQ PKEDEEEEDD VVAPKPLVEP
161    EEVKTFKKGR GGEAAPHELT EEEKQQILHS EEFLSFFDHS TRIVERALSE QINIFFDYSG RDLEDKEGEI QAGAKLSLNR
241    QFFDERWSKH RVVCCLDWSS QYPELLVASY NNNEDAPHEP DGVALVWNMK YKKTTPEYVF HCQSAVMSAT FAKFHPNLVV
321    GGTYSGQIVL WDNRSNKRTP VQRTPLSAAA HTHPVYCVNV VGTQNAHNLI SISTDGKICS WSLDMLSQPQ DSMELVHKQS
401    KAVAVTCMSF PIGDVNNFVV GSEEGSVYTA CRHGSKAGIS EMFEGHQGPI TGIHCHAAVG PVDFSHLFVT SSFDWTVKLW
481    TTKNNKPLYS FEDNSDYVYD VMWSPTHPAL FACVDGMGRL DLWNLNNDTE VPTASITVEG NPALNRVRWT HTGREIAVGD
561    SEGQIVIYDV GEQIAVPRSD EWTRFGRTLA EINANRADAE EEAATRIPA
```

5.284 transcription elongation factor A 1 [Gallus gallus]

Protein Accession	gi|57530393
Mean Expression Ratio	1.03
Median Expression Ratio	1.03
Credible Interval	(0.802, 1.34)
Associated Peptides	1
Associated Spectra	1
Coverage	0.0493

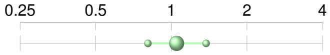

A	2.5	50	97.5	Sequence
1	0.8	1.1	1.4	KKEPASSSQNSPEAR

```
1      MTTEDEIIRI AKKMDKMVQK KNAAGALDLL KELKNIPMTL ELLQTTRIGM SVNAIRKQST DEEVTSLAKS LIKSWKKLLD
81     GPSTDKDSEE KKKEPASSSQ NSPEAREESS SSSNSSSRKE EGSAPSNSFI PSFPRAPSTS DSVRVKCREM LSAALRTGDD
161    YIAIGADEEE LGSQIEEAIF QELKNTDMKY KNRVRSRIAN LKDAKNPNLR KNVLCGNIPP DKFAKMTAEE MASDELKEMR
241    KNLTKEAIRE HQMAKTGGTQ TDLFTCGKCK KKNCTYTQVQ TRSADEPMTT FVVCNECGNR WKFC
```

5.285 chromobox homolog 1 (HP1 beta homolog Drosophila) [Gallus gallus]

Protein Accession	gi|45383494
Mean Expression Ratio	1.03
Median Expression Ratio	1.03
Credible Interval	(0.798, 1.34)
Associated Peptides	1
Associated Spectra	1
Coverage	0.0649

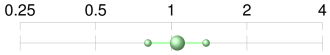

A	2.5	50	97.5	Sequence
1	0.8	1.1	1.4	CPQVVISFYEER

```
1       MGKKQNKKKV EEVLEEEEEE YVVEKVLDRR VVKGKVEYLL KWKGFSDEDN TWEPEENLDC PDLIAEFLQS QKTAHESEKS
81      EGSKRKAESD TEDKGEESKP KKKKEESEKP RGFARGFEPE RIIGATDSSG ELMFLMKWKN SDEADLVPAK EANIKCPQVV
161     ISFYEERLTW HSYPSEDDDK KEDKN
```

5.286 protein phosphatase 2 (formerly 2A), catalytic subunit, alpha [Gallus gallus]

Protein Accession	gi|57525024
Mean Expression Ratio	1.03
Median Expression Ratio	1.03
Credible Interval	(0.838, 1.28)
Associated Peptides	2
Associated Spectra	3
Coverage	0.0809

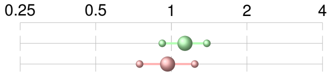

A	2.5	50	97.5	Sequence
2	0.92	1.1	1.4	YSFLQFDPAPR
1	0.75	0.96	1.2	QITQVYGFYDECLR

```
1       MEEKVFTKEL DQWVEQLNEC KQLSEGQVKS LCEKAKEILT KESNVQEVRC PVTVCGDVHG QFHDLMELFR IGGKSPDTNY
81      LFMGDYVDRG YYSVETVTLL VALKVRYRER ITILRGNHES RQITQVYGFY DECLRKYGNA NVWKYFTDLF DYLPLTALVD
161     GQIFCLHGGL SPSIDTLDHI RALDRLQEVP HEGPMCDLLW SDPDDRGGWG ISPRGAGYTF GQDISETFNH ANGLTLVSRA
241     HQLVMEGYNW CHDRNVVTIF SAPNYCYRCG NQAAIMELDD TLKYSFLQFD PAPRRGEPHV TRRTPDYFL
```

5.287 COBW domain containing protein1 [Gallus gallus]

Protein Accession	gi|71895319
Mean Expression Ratio	1.03
Median Expression Ratio	1.03
Credible Interval	(0.804, 1.33)
Associated Peptides	1
Associated Spectra	1
Coverage	0.0216

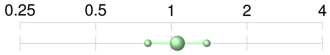

A	2.5	50	97.5	Sequence
1	0.81	1.1	1.4	AIENLMQK

```
1       MEDEDCPDLV PIDVGIVQDS EPGSGRKIPV TIITGYLGAG KTTLLNYILT EQHSKRIAVI LNEFGEGSAL EKSLAISQGG
81      ELYEEWLELR NGCLCCSVKD NGVKAIENLM QKRGKFDYIL LETTGLADPG AVASMFWVDS ELGSDIYLDG IVSVVDAKHG
161     LQHLTEEKPE GLVNEAARQV ALADLIIINK TDLVSGEELN KVRASVRSIN GLVKILETQR SRVDLSNVLD LHAFDSLSGI
241     SLQKKLENMK TAHAHLDKGI VTVTFEVLGN IKEENLNLFI QNLLWEKNVK DKTGRTMDVI RLKGLVSIQG KSHQVIVQGV
321     HELYDLEETA VAWKEDEKRT NRLVLIGRNL NKEIIKEVFI ETVSEKHESS
```

5.288 PREDICTED: similar to Rap guanine nucleotide exchange factor (GEF) 3 [Gallus gallus]

Protein Accession	gi|118129647
Mean Expression Ratio	0.967
Median Expression Ratio	0.969
Credible Interval	(0.746, 1.25)
Associated Peptides	1
Associated Spectra	1
Coverage	0.00567

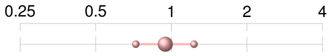

A	2.5	50	97.5	Sequence
1	0.72	0.94	1.2	LAAHLK

```
1       MPGCREPREE EDEEEDEEEE GFGVQEGLQG MEQPEPCRAP SGGRPPPAPV CTRRCQQTPL LDAESSLDYG HSLTQIWRAG
81      KLLFTYLTST SPSLIRDHKH HLRHHRRCCS GKELVDWMLN AELGIQTRSQ AIGVGQVLVD GGVLTHVKQE WHFQDKDTQF
161     YRFAELELSP EPRVGLRDAE ELLEAVTFLA QLGPDALLTM ALRKPPAQRT EDELELIFEE LLHIKAVAHL SNSVKRELAS
241     VLMFESHQRA GTVLFSQGDK GTSWYIVWKG SVNVVTHGKG LVATLHEGDD FGQLALVNDA PRAASIILRE DNCHFLRVDK
321     QDFNRILKDV EANTMRLKEH GKVVLVLQKN LQGGSSQPAA TRSSRMENGD GSISPQPKSR SSMSWLSSQD EAALSSSCAL
401     RAHDKVPYEV YRADHSCLVT VLPVNASVRD VLQSLTPQLG WDGEHLLVKV NSAGDKVGLQ LDAVGVFTSL GLNERLFAVS
481     VEELGGLTPP PERLGPHVGS SDTLDLISSK DLASHLTDHD WNLFKSIHQV EMIQYIMGPQ KFHEVTTANL ERMMRRFNEL
561     QYWVATELCL CPELGRRAQL LRKFIKLAAH LKEQKNLNSF FAVMFGVSNT AVSRLAKTWE RLPHKIRKLH AALERMLDPS
641     WNHRVYRLAV AKLSPPIIPF VPLLLKDMTF IHEGNRTLAE NLINFEKMHM MAKTVRILQR CRGQAHAPLS PLRNRSPHRP
721     EDARAIRIST SLYTDPDSCR GRCEEPYSHE DECHCDVGCR SRNSCCWDYP EHCGAEHFYS SHNAISDEEL LHVSEQLYGA
801     DHNKAQPNDI TINPQHRAAP DQTGLQEDRS PEPLYAYVNE KLFSKPTYSS FIRLLDNYQR ATGREEEVTA EELREQDVFL
881     REVMETELMK KLFAFLHTKN RYGSEEEFLQ DLKEMWFGLY SRGDGEKDSS GFEHVFSGEI KKGKVSGFHN WIRFYLLEKQ
961     GLVNYFSHNF DGPWDTYPDV LGLQFSWDGF YKEVGSAFIG CSPEFEFGIY TLCFIARPGR ACHLSLGGHG VSIQTYTWTK
1041    STYGHGKKYI AAAYVISP
```

5.289 tubulin, beta 2B [Gallus gallus]

Protein Accession	gi|52138699
Mean Expression Ratio	0.969
Median Expression Ratio	0.969
Credible Interval	(0.779, 1.20)
Associated Peptides	1
Associated Spectra	4
Coverage	0.0202

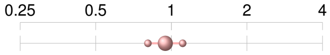

A	2.5	50	97.5	Sequence
4	0.8	0.95	1.1	YLTVAAIFR

```
1       MREIVHIQAG QCGNQIGAKF WEVISDEHGI DPTGSYHGDS DLQLERINVY YNEATGNKYV PRAILVDLEP GTMDSVRSGP
81      FGQIFRPDNF VFGQSGAGNN WAKGHYTEGA ELVDSVLDVV RKESESCDCL QGFQLTHSLG GGTGSGMGTL LISKIREEYP
161     DRIMNTFSVM PSPKVSDTVV EPYNATLSVH QLVENTDETY CIDNEALYDI CFRTLKLTTP TYGDLNHLVS ATMSGVTTCL
241     RFPGQLNADL RKLAVNMVPF PRLHFFMPGF APLTSRGSQQ YRALTVPELT QQMFDSKNMM AACDPRHGRY LTVAAIFRGR
321     MSMKEVDEQM LNVQNKNSSY FVEWIPNNVK TAVCDIPPRG LKMSATFIGN STAIQELFKR ISEQFTAMFR RKAFLHWYTG
401     EGMDEMEFTE AESNMNDLVS EYQQYQDATA DEQGEFEEEG EEDEA
```

5.290 heterogeneous nuclear ribonucleoprotein U, MARs binding protein p120, hnRNP U=matrix attachment reg

Protein Accession	gi|546484
Mean Expression Ratio	1.03
Median Expression Ratio	1.03
Credible Interval	(0.812, 1.31)
Associated Peptides	1
Associated Spectra	2
Coverage	1

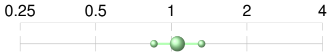

A	2.5	50	97.5	Sequence
2	0.85	1.1	1.3	NFILDQTNVSAAAQR

```
1        NFILDQTNVS AAAQR
```

5.291 PREDICTED: hypothetical protein [Gallus gallus]

Protein Accession	gi|118102310
Mean Expression Ratio	1.03
Median Expression Ratio	1.03
Credible Interval	(0.837, 1.27)
Associated Peptides	2
Associated Spectra	3
Coverage	0.08

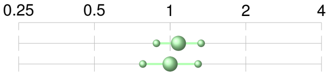

A	2.5	50	97.5	Sequence
2	0.88	1.1	1.3	AVFVDLEPAVIDEVR
1	0.78	1	1.3	QLFHPEQLISGKEDAANNYAR

```
1        MTRECISVHV GQAGVQIGNA CWELYCLEHG IQPNGTMPSD KTIGGGDDSF NTFFSETGAG KHVPRAVFVD LEPAVIDEVR
81       NGTYRQLFHP EQLISGKEDA ANNYARGHYT VGKEIIDLVL ERIRKLSDQC TGLQGFLIFH SFGGGTGSGF TSLLMERLSV
161      DYGKKSKLEF AIYPAPQVST AVVEPYNSIL TTHTTLEHSD CAFMVDNEAI YDICRRNLDI ERPTYTNLNR LIGQIVSSIT
241      ASLRFDGALN VDLTEFQTNL VPYPRIHFPL VTYSPIISAE KAYHEQLSVS EITNACFEPS NQMVKCDPRH GKYMACCMLY
321      RGDVVPKDVN AAIAAIKTKR TIQFVDWCPT GFKVGINYQP PTVVPGGDLA KVQRAVCMLS NTTAIAEAWA RLDHKFDLMY
401      AKRAFVHWYV GEGMEEGEFS EAREDLAALE KDYEEVGTDS MDGEDEGEEY
```

5.292 Tubulin beta-3 chain (Beta-tubulin class-IV)

Protein Accession	gi|135464
Mean Expression Ratio	0.968
Median Expression Ratio	0.97
Credible Interval	(0.787, 1.19)
Associated Peptides	2
Associated Spectra	3
Coverage	0.0607

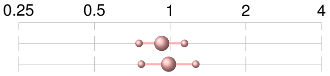

A	2.5	50	97.5	Sequence
2	0.75	0.93	1.1	AVLVDLEPGTMDSVR
1	0.77	0.99	1.3	INVYYNEATGGK

```
1        MREIVHLQAG QCGNQIGAKF WEVISDEHGI DPTGTYHGDS DLQLERINVY YNEATGGKYV PRAVLVDLEP GTMDSVRSGP
81       FRQIFRPDNF VFGQSGAGNN WAKGHYTEGA ELVDSVLDVV RKEAESCDCL QGFQLTHSLG GGTGSGMGTL LISKIREEYP
161      DRIMNTFSVV PSPKVSDTVV EPYNATLSVH QLVENTDETY CIDNEALYDI CFRTLKLTTP TYGDLNHLVS ATMSGVTTCL
241      RFPGQLNADL RKLAVNMVPF PRLHFFMPGF APLTSRGSQQ YRALTVPDLT QQMFDAKNMM AACEPGHGRY LTVAAVFRGR
321      MSMKEVDEQM LNVQNKNSSY FVEWIPNNVK TAVCDIPPRG LKMSATFIGN STAIQELFKR ISEQFTAMFR RKAFLHWYTG
401      EGMDEMEFTE AESNMNDLVS EYQQYQDATA EEEGEFEEEA EEEAE
```

5.293 PREDICTED: hypothetical protein [Gallus gallus]

Protein Accession	gi|118087564
Mean Expression Ratio	0.97
Median Expression Ratio	0.97
Credible Interval	(0.755, 1.25)
Associated Peptides	1
Associated Spectra	1
Coverage	0.0146

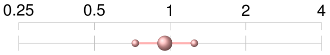

A	2.5	50	97.5	Sequence
1	0.73	0.95	1.2	GYIELELQLR

```
1      MASTAAGKQR IPKVAKVKNK APAEVQITAE QLLREAKERE LELLPPPPQQ KITDVEELND YKLRKRKTFE DNIRKNRTVI
81     SNWIKYAQWE ESLKEIQRAR SIYERALDVD YRNVTLWLKY AEMEMKNRQV NHARNIWDRA ITTLPRVNQF WYKYTYMEEM
161    LGNVAGSRQV FERWMEWQPE EQAWHSYINF ELRYKEVDRA RTIYERFVIV HPDVKNWIKY ARFEEKHCYF AHARKVYERA
241    VEFFGEEHMD EHLYVAFAKF EENQKEFERV RVIYKYALDR IPKQDAQNLF KNYTIFEKKF GDRRGIEDII VSKRRFQYEE
321    EVKANPHNYD AWFDYLRLVE SDADAETVRE VYERAIANVP PIQEKRYWKR YIYLWINYAL YEELEAKDPE RTRQVYQACI
401    ELLPHKKFTF AKIWLLYAQF EIRQKNLPLA RRALGTSIGK CPKNKLFKGY IELELQLREF DRCRKLYEKF LEFAPENCTS
481    WIKFAELETI LGDIDRARAI YELAISQPRL DMPEVLWKSY IDFEIEQEEY EKTRNLYRRL LQRTQHVKVW ISFAQFELSA
561    GKEESLSKCR QIYEEANKAM RNCEEKEERV MLLESWKTFE EEFGTDSTKE RIEKLMPEKI KKRRKLQAED GSDAGWEEYY
641    DYIFPEDTAN QPNLKLLAMA KLWKKQQQEI EAAEMDPDKD IDESQS
```

5.294 microtubule-associated protein, RP/EB family, member 1 [Gallus gallus]

Protein Accession	gi|71894959
Mean Expression Ratio	1.03
Median Expression Ratio	1.03
Credible Interval	(0.838, 1.27)
Associated Peptides	2
Associated Spectra	3
Coverage	0.112

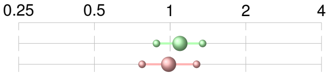

A	2.5	50	97.5	Sequence
**2**	**0.89**	**1.1**	**1.3**	**NIELICQENEGENDPVLQR**
**1**	**0.77**	**0.99**	**1.3**	**KFFDANYDGK**

```
1      MAVNVYSTSV TSDNLSRHDM LAWINESLQL TLTKIEQLCS GAAYCQFMDM LFPGSVALKK VKFQAKLEHE YIQNFKVLQA
81     GFKRMGVDKI IPVDKLVKGK FQDNFEFVQW FKKFFDANYD GKEYDPVAAR QGQETVAPNL VAPVVNKPKK PLAPQRPIVA
161    QRTPATPKGS TGMVKKAAGD DESAGLIEQI NVLKLTVEDL EKERDFYFGK LRNIELICQE NEGENDPVLQ RIVEILYATD
241    EGFVIPDEGA PQEEQEEY
```

5.295 annexin A6 [Gallus gallus]

Protein Accession	gi|50982399
Mean Expression Ratio	1.03
Median Expression Ratio	1.03
Credible Interval	(0.799, 1.33)
Associated Peptides	1
Associated Spectra	1
Coverage	0.0283

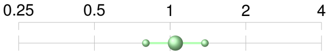

A	2.5	50	97.5	Sequence
**1**	**0.8**	**1.1**	**1.4**	**GTVQPASNFNDDGDAQVLR**

```
1      MAPKGKVYRG SVKDFPGFNA SQDADALYNA MKGFGSDKDA ILDLITSRSN KQRVEICQAY KSQYGKDLIA DLKYELTGKF
81     ERLIVSLMRP PAYSDAKEIK DAIAGIGTDE KCLIEILASR TNQEIHDLVA AYKDAYERDL EADVVGDTSG HFKKMLVVLL
161    QGAREEDDVV SEDLVEQDAK DLLEAGELKW GTDEAQFIYI LGRRSKQHLR MVFDEYLKIS GKPIERSIRA ELSGDFEKLM
241    LAVVKCVRST AEYFAERLYK AMKGLGTRDN TLIRIMVSRS EIDMLDIREV FRTKYDKSLH NMIKEDTSGE YKKALLKLCG
321    GDDDAAGEFF PEAAQVAYRM WELSAVAKVE LRGTVQPASN FNDDGDAQVL RKAMKGLGTD EGAIIEVLTQ RSNAQRQQIL
401    KAYKAHYGRD LLADLKSELS GSLAKLILGL MLTPAQYDAK QLRKAVEGAG TDESTLIEIM ATRNNQEIAA INEAYQQAYH
481    KSLEDDLSSD TSGHFKRILV SLALGNRDEG PENLTQAHED AKVVAETLKL ADVASNDSSD SLETRFLSIL CTRSYPHLRR
561    VFQEFVKMTN HDVEHAIRKR MSGDVRDAFV AIVRSVKNKP AFFADKLYKS MKGAGTDERT LTRIMISRSE IDLLNIRGEF
641    IDLFDKSLYH MIEKDTSGDY CKALLALCGG DD
```

5.296 FK506 binding protein 1A, 12kDa [Gallus gallus]

Protein Accession	gi|45383498
Mean Expression Ratio	1.03
Median Expression Ratio	1.03
Credible Interval	(0.797, 1.34)
Associated Peptides	1
Associated Spectra	1
Coverage	0.0463

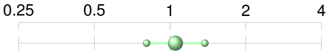

A	2.5	50	97.5	Sequence
**1**	**0.8**	**1.1**	**1.4**	**NKPFK**

```
1       MGVHVETIAP GDGRTFPKRG QTCVVHYTGM LEDGKKFDSS RDRNKPFKFV MGKQEVIRGW EEGVAQMSVG QRAKMTISPD
81      YAYGSTGHPG IIPPNATLIF DVELMKLE
```

5.297 triosephosphate isomerase 1 [Gallus gallus]

Protein Accession	gi|45382061
Mean Expression Ratio	1.03
Median Expression Ratio	1.03
Credible Interval	(0.895, 1.18)
Associated Peptides	7
Associated Spectra	9
Coverage	0.282

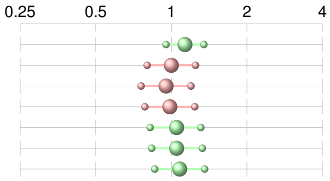

A	2.5	50	97.5	Sequence
3	0.96	1.1	1.3	LSADTEVVCGAPSIYLDFAR
1	0.8	1	1.3	FFVGGNWK
1	0.76	0.95	1.2	DIGAAWVILGHSER
1	0.78	0.99	1.2	TATPQQAQEVHEK
1	0.83	1.0	1.3	HVFGESDELIGQK
1	0.83	1.1	1.3	KFFVGGNWK
1	0.86	1.1	1.4	RHVFGESDELIGQK

```
1       MAPRKFFVGG NWKMNGDKKS LGELIHTLNG AKLSADTEVV CGAPSIYLDF ARQKLDAKIG VAAQNCYKVP KGAFTGEISP
81      AMIKDIGAAW VILGHSERRH VFGESDELIG QKVAHALAEG LGVIACIGEK LDEREAGITE KVVFEQTKAI ADNVKDWSKV
161     VLAYEPVWAI GTGKTATPQQ AQEVHEKLRG WLKSHVSDAV AQSTRIIYGG SVTGGNCKEL ASQHDVDGFL VGGASLKPEF
241     VDIINAKH
```

5.298 PREDICTED: carnitine palmitoyltransferase II [Gallus gallus]

Protein Accession	gi|118094580
Mean Expression Ratio	0.97
Median Expression Ratio	0.97
Credible Interval	(0.749, 1.25)
Associated Peptides	1
Associated Spectra	1
Coverage	0.0153

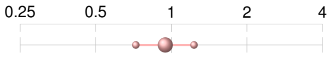

A	2.5	50	97.5	Sequence
1	0.72	0.95	1.2	LEFKLNDALK

```
1       MAARQLAVGR VVLGCWRRAY SSAGAAEFLH RSIVPTMHYQ KSLPRLPVPK LEDTIRRYLN AQKPLLNDDQ FRKTEELAHQ
81      FEKGIGRELH EQLVAQDNQN KHTSYITGPW FDMYLKAREP VVLNFNAFMS FNPDPKSEYN DQLIRATNMT VSAIRFMKTF
161     RAGYLEPEVF HLNPEKSDTQ LFKKIIRFVP SSLSWFGAYM VNAYPLDMSQ YFRLFNSTRL PKLNRDELYT DEKAKHLLVL
241     RKGNFYIFDV LDKDGNMLKP SEIQAHLKYI LSDNSPASAF PLGYLSSENR DTWALLRKDL LDNGNEEALR KIDSAVFCLS
321     LDDFPIKDFV HLSHTMLHGD AANRWYDKSF NLIIAKDGTA GINFEHSWGD GVAVLRFQNE VFKDSTEIPA VSPQSQPASV
401     DSSTAVQKLE FKLNDALKAG ITKAKQNFDA SVEGLSLNMI QFHEGGKELL KQKKVSPDAV AQLAFQMAFL RQYNQTVATY
481     ESCSTAAFKH GRTETIRPAS IHTKKCSEAF VREPSKHSTE ELQELIVECS KYHGRLTKEA AMGQGFDRHL FGLRYLALSK
561     GIALPDFYQD QAYVRLNYNI ISTSTLVSPA VQLGGFGPVV PDGFGLGYQV HDDWIGCNVS SYPTRNGKEF LQCVYKSLED
641     IFNVLKGKKV GS
```

5.299 Desmin

Protein Accession	gi|118453
Mean Expression Ratio	1.03
Median Expression Ratio	1.03
Credible Interval	(0.927, 1.15)
Associated Peptides	13
Associated Spectra	20
Coverage	0.330

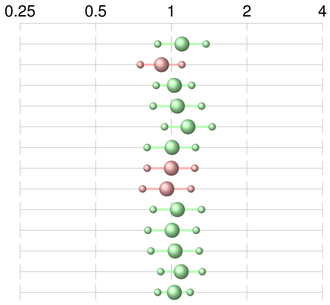

A	2.5	50	97.5	Sequence
1	0.88	1.1	1.4	DGEVVSEATQQQHEVL
2	0.75	0.91	1.1	ADVDAATLAR
3	0.87	1.0	1.2	FAGEAGGYQDTIAR
1	0.85	1.1	1.3	FLEQQNALMVAEVNR
1	0.94	1.2	1.5	TFGGGTSPVFPR
1	0.8	1	1.2	VAEMYEEELR
1	0.8	1	1.2	NIAEAEEWYK
1	0.77	0.96	1.2	EYQDLLNVK
1	0.85	1.1	1.3	KLLEGEENR
1	0.8	1	1.3	TNEKVELQELNDR
1	0.83	1.0	1.3	HQIQSYTCEIDALK
2	0.9	1.1	1.3	RIESLQEEIAFLK
4	0.88	1.0	1.2	KVHEEEIR

```
1       SQSYSSSQRV SSYRRTFGGG TSPVFPRASF GSRGSGSSVT SRVYQVSRTS AVPTLSTFRT TRVTPLRTYG SAYQGAGELL
81      DFSLADAMNQ EFLQTRTNEK VELQELNDRF ANYIEKVRFL EQQNALMVAE VNRLRGKQPT RVAEMYEEEL RELRRQVDAL
161     TGQRARVEVE RDNLLDNLQK LKQKLQEEIQ LKQEAENNLA AFRADVDAAT LARIDLERRI ESLQEEIAFL KKVHEEEIRE
241     LQAQLQEQHI QVEMDISKPD LTAALRDIRA QYESIAAKNI AEAEEWYKSK VSDLTQAANK NNDALRQAKQ EMLEYRHQIQ
321     SYTCEIDALK GTNDSLMRQM REMEERFAGE AGGYQDTIAR LEEEIRHLKD EMARHLREYQ DLLNVKMALD VEIATYRKLL
401     EGEENRISIP MHQTFASALN FRETSPDQRG SEVHTKKTVM IKTIETRDGE VVSEATQQQH EVL
```

5.300 hematological and neurological expressed 1 [Gallus gallus]

Protein Accession	gi|57530211
Mean Expression Ratio	0.971
Median Expression Ratio	0.97
Credible Interval	(0.748, 1.26)
Associated Peptides	1
Associated Spectra	1
Coverage	0.122

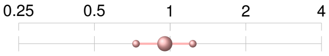

A	2.5	50	97.5	Sequence
1	0.73	0.95	1.2	SLPAAPVPSPVAPAPAPSR

```
1       MTTTTIFSGM DPSGRNSSRV LRPPGGGSNF SLGFDEPKEQ PMRKNKMASS IFETPEENPP SWAKSSGTKP GEIREDTELS
81      GPQRNSADAN CGDVIDPKGG DGGGETYENT EADVEAAPGQ NEEKSLPAAP VPSPVAPAPA PSRRNPPGGK SSLVLG
```

5.301 hypothetical protein LOC427269 [Gallus gallus]

Protein Accession	gi|71894835
Mean Expression Ratio	0.97
Median Expression Ratio	0.97
Credible Interval	(0.75, 1.25)
Associated Peptides	1
Associated Spectra	1
Coverage	0.111

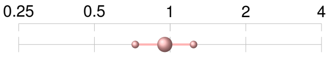

A	2.5	50	97.5	Sequence
1	0.73	0.95	1.2	LAAAAAATSSALSPR

```
1       MSAGSSMAAA LGGLGLLHGR FRLAAAAAAT SSALSPRAPV RGGAATFPSA GRNYGSEAKE EEELRVQYLD EEHKGIVVLG
81      LNRSHAKNAL NKNLLKMMSK AVDALKSDKK VRTVIFRSEV PGIFCAGEDE LRRGI
```

5.302 hypothetical protein [Gallus gallus]

Protein Accession	gi|53129586
Mean Expression Ratio	1.03
Median Expression Ratio	1.03
Credible Interval	(0.9, 1.18)
Associated Peptides	8
Associated Spectra	9
Coverage	0.27

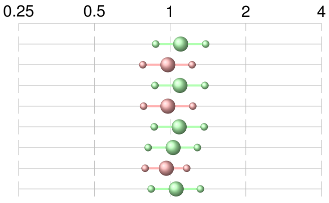

A	2.5	50	97.5	Sequence
1	0.88	1.1	1.4	LVIIEGDLER
1	0.78	0.98	1.2	IQLVEEELDR
1	0.87	1.1	1.4	KIQVLQQQADDAEER
1	0.78	0.98	1.2	KLVIIEGDLER
1	0.86	1.1	1.4	ALKDEEK
1	0.82	1.0	1.3	KYEEVAR
2	0.8	0.96	1.2	EDKYEEEIK
1	0.84	1.1	1.3	HIAEEADRK

```
1       MAGATTIEAV KRKIQVLQQQ ADDAEERADR LQREVEAERR NREQAEAEVA SLNRRIQLVE EELDRAQERL ATALQKLEEA
81      EKAADESERG MKVIENRALK DEEKMELQEI QLKEAKHIAE EADRKYEEVA RKLVIIEGDL ERTEERAELA ESKCSELEEE
161     LKNVTNNLKS LEAQAEKYSQ KEDKYEEEIK ILTDKLKEAE TRAEFAERSV AKLEKTIDDL EDELYAQKLK YKAISEELDH
241     ALNDMTSI
```

5.303 PREDICTED: hypothetical protein [Gallus gallus]

Protein Accession	gi|118085600
Mean Expression Ratio	0.971
Median Expression Ratio	0.972
Credible Interval	(0.766, 1.22)
Associated Peptides	1
Associated Spectra	2
Coverage	0.0242

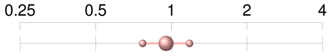

A	2.5	50	97.5	Sequence
**2**	**0.77**	**0.95**	**1.2**	**NTLLELFGQIER**

```
1       MPIESGSCAS ARQAKQKRKS HSLSIRRTNS SEQERAGLQR DMLEGQDSKL PSSVRNTLLE LFGQIEREFE NLYIENLELR
81      REIDTLNERL AAEGQTIDGA ELSKGQLKTK ASHSTSQLSQ KLKTTYKAST SKIVSSFKTT TSRAICQLVK EYVGHRDGIW
161     DVSVAKTQPV VLGTASADHT ALLWSIETGK CLVKYVGHVG SVNSIKFHPT EQVALTASGD QTAHIWRYIV QLPTPQPTAD
241     CNQVSGEDEF EFSDKDEPDG DGDGSSDCPT VRAPLTSLKS HQGVVIAADW LVGGKQAVTA SWDRTANLYD VETSELVHSL
321     TGHDQELTHC CTHPTQRLVV TSSRDTTFRL WDFRDPSIHS VNVFQGHTDT VTSAVFTVGD NVVSGSDDRT VKVWDLKNMR
401     SPIATIRTDS AVNRINVCVG QRIIALPHDN RQVRLFDMSG VRLARLPRSN RQGHRRMVCC SAWSEDHPIC NLFTCGFDRQ
481     AIGWNINIPA LLQEK
```

5.304 PREDICTED: similar to TBC1 (tre-2/USP6, BUB2, cdc16) domain family, member 1 [Gallus gallus]

Protein Accession	gi|118090617
Mean Expression Ratio	0.973
Median Expression Ratio	0.972
Credible Interval	(0.75, 1.26)
Associated Peptides	1
Associated Spectra	1
Coverage	0.0178

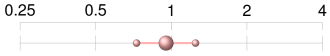

A	2.5	50	97.5	Sequence
**1**	**0.73**	**0.95**	**1.2**	**QTSQPSAENAVNEVPSSAPR**

```
1       MSPLFSWVAK VGGGHGPARG GGTRPVGIRG GSGALLLARL GASSEGKLVR FRGWLGGLEA VRCGGEGSGE SCRKVWYVPE
81      SPPACSGIRV PEIISSIRQA GKIARQEEFQ NNLSDIEDPF AKKFEVLFCG RVTVAHKNAP PALIDECIEK FNHASCTKKS
161     DSDSSSQQHE TADNSGEEEK RPIRKSFSQP GLRSLAFKKD FQEGSHRSNS FVRSFEEDAI SLNLKSQLIY GHSVVQPTDI
241     AENRTMLFTI GQSEVYLISP DTKKVAIEKS FKEISFCSQG IRHVDHFGFI CRESSENGGF HFVCYVFQCT DEALVDEIMM
321     TLKQAFTVAA VQQTSKAQPQ LCETCPLQSL HKLCEKIEGL HPSKTKLELQ KHLTTLNSQE QASVFEEVKK LRPRNDQKEN
401     ELIISILRNM YEEKQKNHVH IGEAKQTSQP SAENAVNEVP SSAPRFRLDM LKNKAKRSLT ESFESILSRG SKARGPLDSS
481     SGSVDLEASV SSNLSSTNKV SKACDAHRVP TLPPENAVKS SGSVDDLSSD PDSYAIDHSV TLPQQSFRRR ANTLSHLPSE
561     SRESLVPVET SPSVPQRKLM RYHSVSTETP HKRNDYESKH SQQAAGADSP VRTRRHSWRQ QIFLRVATPQ KACDSPSRYD
641     DYTELGELPP RSPLEPVCED GPFGPVKEER KRTSRELREL WKKAIIQQIL LLRMEKENQK LQASENNLQN RRLKLDYEEI
721     TPCLKEVTLI WEKMLSTPGR SKIKFDMEKI HSAVGQGVPR HHRGEIWKFL AEQYHLKHQF PNKQQPKDTP YKELLKQLTS
801     QQHAILIDLG RTFPTHPYFS AQLGAGQLSL YNILKAYSLL DQEVGYCQGL SFVAGVLLLH MSEEDAFKML KFLMFDVGLR
881     KQYRPDMTIL QIQMYQLSRL LHDYHRDLYN HLEEHEIGPS LYAAPWFLTM FASQFPLGFV ARVFDMLFLQ GSEAIFKVAL
961     SLLGSHKPLI LQHENLETIV DFIKNTLPNL GLVQMEKTIS QVFEMDIAKQ LQAYEVEYHV LQDELIDSSL NDNQRLDKLE
1041    KANSSLRKQN FELLEELQVA NGKIQNLEAT VELLLTNEGK LKESILTLEQ ERTALQKAVE EMRRKIGDTN EKPLLTRQEH
1121    LDAD
```

5.305 PREDICTED: hypothetical protein, partial [Gallus gallus]

Protein Accession	gi|118121115
Mean Expression Ratio	0.972
Median Expression Ratio	0.972
Credible Interval	(0.754, 1.25)
Associated Peptides	1
Associated Spectra	1
Coverage	0.0188

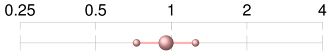

A	2.5	50	97.5	Sequence
**1**	**0.72**	**0.95**	**1.2**	**IILANDPDADR**

```
1       NPKTSAIVKE LVAEGNVEEL QKYFGSRMEF GTAGLRAAMG AGISHMNDLT IIQTTQGFCR YLEKNFSDLK NRGVVIGFDA
81      RAHLSSGGSS KRFARLAANT FISQGVPVYL FSDVTPTPFV PYTVTHLKLC AGIMVTASHN PKQDNGYKVY WENGAQIISP
161     HDKGISQAIE ENKEPWPQAW DDKQIDSSAL LHDPYATINK EYFKDIQKQC FHRNINKETN LKFVHTSVHG VGHKFVQLAF
241     KAFDLRPPFA VPEQKDPDPE FQRVKYPNPE EGKGVLTLSF ALAEKDGAKI ILANDPDADR LAVAEKQESG EWKVFSGNEL
321     GALLGWWIFT CWKNQNRDAC AIKDVYMLSS TVSSKILRAI ALKEGFHFEE TLTGFKWMGN RAKQLMDQGK AVLFAFEEAI
401     GYMCCPAVLD KDGVSAAVIT AEMASFLATR NLSLSQQLKA VYDEYGFHIT KASYFICHDP KVIQQLFDNL RNYDGKDTYP
481     KSCGRFKVSG IRDLTTGYDS SQPDLKAILP TSKSSQMITF TFANGGVATM RTSGTEPKIK YYSELCAPPG NSDVEQLKKE
561     LDELVSALEK HFFQPEKNNL QRKTE
```

5.306 PREDICTED: similar to ragA [Gallus gallus]

Protein Accession	gi|50745896
Mean Expression Ratio	0.971
Median Expression Ratio	0.972
Credible Interval	(0.749, 1.26)
Associated Peptides	1
Associated Spectra	1
Coverage	0.0511

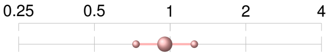

A	2.5	50	97.5	Sequence
**1**	**0.73**	**0.96**	**1.2**	**NFAQIIEADEVLLFER**

```
1       MPSTAMKKKV LLMGKSGSGK TSMRSIIFAN YIARDTRRLG ATIDVEHSHV RFLGNLVLNL WDCGGQDTFM ENYFTSQRDN
81      IFRNVEVLIY VFDVESRELE KDMHYYQSCL EAILQNSPDA KIFCLVHKMD LVQEDQRDLI FKEREEDLRR LSRPLECACF
161     RTSIWDETLY KAWSSIVYQL IPNVQQLEMN LRNFAQIIEA DEVLLFERAT FLVISHYQCK EQRDIHRFEK ISNIIKQFKL
241     SCSKLAASFQ SMEVRNSNFA AFIDIFTSNT YVMVVMSDPS IPSAATLINI RNARKHFEKL ERVDGPKHSL LMR
```

5.307 nucleosome assembly protein 1-like 4 [Gallus gallus]

Protein Accession	gi|57530009
Mean Expression Ratio	0.973
Median Expression Ratio	0.972
Credible Interval	(0.794, 1.20)
Associated Peptides	2
Associated Spectra	4
Coverage	0.0638

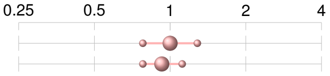

A	2.5	50	97.5	Sequence
**1**	**0.78**	**1**	**1.3**	**GIPDFWFTIFR**
**3**	**0.78**	**0.93**	**1.1**	**KYAALYQPLFDKR**

```
1       MADNSSSKAE DTASDSVEAA KNASDKKEKL ADQVMQNPQV LAALQERLDN TALTPSSYIE TLPKAVKRRI DALKQLQVKC
81      AHIEAKFYEE VHDLERKYAA LYQPLFDKRR EFINGEAEPT DAESEWHSEN EEEEKLAGDL KNVVVIEEKA EAEEINVKGI
161     PDFWFTIFRN VDMLSELVQE YDEPILKHLQ DIKVKFSEPG QPMSFSLEFP LGPNDYFPNT VLTKTYKMKS EPDKTDPFSF
241     EGPEIVDCDG CTIDWKKGKN VTVKTIKKKQ KHKGRGTVRT ITKQVPNDSF FNFFNPIKVS GDGESLDEDS EFTLAADFEI
321     GHFFRERIVP RAVLYFTGEA IEDDDNFEEG EEGEEEELEG EEEGEEEEDA ESDPKV
```

5.308 PREDICTED: similar to Fascin homolog 1, actin-bundling protein (Strongylocentrotus purpuratus) [Gal

Protein Accession	gi|118097808
Mean Expression Ratio	0.971
Median Expression Ratio	0.972
Credible Interval	(0.75, 1.26)
Associated Peptides	1
Associated Spectra	1
Coverage	0.0513

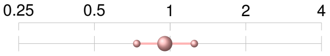

A	2.5	50	97.5	Sequence
**1**	**0.74**	**0.96**	**1.2**	**KVTGTLDSNR**

```
1       MGGIALRGYC SDGYRSAPGG PYVPAEPHSA HRNASCYFDI EWCDKRITLR AANGKYVTAK KNGQLAASME TAGETEHFVM
81      KLINRPIIVL RGEHGFIGCR KVTGTLDSNR SSYDVFQLEF NDGAYNIKDT TGKYWMVGSE SSVTSSSDTP VDFFFEFCDY
161     NKVAIKINGK YLKGDHAGVL KASADAIDAS TLWEY
```

5.309 Chain A, Triosephosphate Isomerase From Gallus Gallus, Loop 6 Mutant T175v

Protein Accession	gi|52695585
Mean Expression Ratio	0.971
Median Expression Ratio	0.972
Credible Interval	(0.752, 1.25)
Associated Peptides	1
Associated Spectra	1
Coverage	0.0524

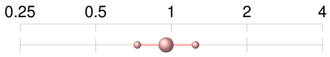

A	2.5	50	97.5	Sequence
1	0.73	0.96	1.2	VATPQQAQEVHEK

```
1       MAPRKFFVGG NWKMNGDKKS LGELIHTLNG AKLSADTEVV CGAPSIYLDF ARQKLDAKIG VAAQNCYKVP KGAFTGEISP
81      AMIKDIGAAW VILGHSERRH VFGESDELIG QKVAHALAEG LGVIACIGEK LDEREAGITE KVVFEQTKAI ADNVKDWSKV
161     VLAYEPVWAI GTGKVATPQQ AQEVHEKLRG WLKSHVSDAV AQSTRIIYGG SVTGGNCKEL ASQHDVDGFL VGGASLKPEF
241     VDIINAKH
```

5.310 PREDICTED: similar to potassium channel tetramerization domain-containing 1 isoform 1 [Gallus gallu

Protein Accession	gi|118086901
Mean Expression Ratio	1.03
Median Expression Ratio	1.03
Credible Interval	(0.794, 1.33)
Associated Peptides	1
Associated Spectra	1
Coverage	0.0377

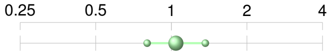

A	2.5	50	97.5	Sequence
1	0.8	1.0	1.4	RTSRAPSVIR

```
1       MFQDSRPNMS RPLITRSPAS PLNNQGIPTP AQLTKSNAPV HIDVGGHMYT SSLATLTKYP DSRIGRLFDG TEPIVLDSLK
81      QHYFIDRDGQ MFRYILNFLR TSKLLIPDDF KDYSLLYEEA KYFQLQPMLG EMERWKQDRE SGRFSKSCEC LVVRVAPDLG
161     ERITLSGDKS LIEEVFPEIG DVMCNSVNAG WNHDSTHVIR FPLNGYCHLN SVQVLERLQQ RGFEIVGSCG GGVDSSQFSE
241     YVLRRELRRT SRAPSVIRIK QEPLD
```

5.311 PREDICTED: similar to Leucyl-tRNA synthetase, cytoplasmic (Leucine–tRNA ligase) (LeuRS) [Gallus ga

Protein Accession	gi|118097622
Mean Expression Ratio	0.973
Median Expression Ratio	0.973
Credible Interval	(0.786, 1.21)
Associated Peptides	2
Associated Spectra	2
Coverage	0.0246

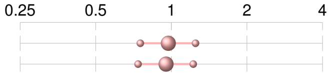

A	2.5	50	97.5	Sequence
1	0.75	0.97	1.3	GTGVVTSVPSDSPDDIAALR
1	0.74	0.95	1.2	FDDPILGPR

```
1       MAERKGTAKV DFLKRIEKEI QQKWADERVF EAGTGDGHNP RSKGKYFVTF PYPYMNGRLH LGHTFSLSKC EFAVGYQRLK
81      GKSCLFPFGL HCTGMPIKAC ADKLKREMEL YGCPPEFPDE EEEEENSAK KDEEIIIKDK AKGKKSKAAA KTGSSKYQWG
161     IMKSLGLSDE EVVSFSEAEH WLDYFPPLAV QDLKSMGLKV DWRRSFITTD VNPYYDSFVR WQFLTLKERN KIKFGKRYTI
241     YSPKDGQPCM DHDRQTGEGV GPQEYTLIKM KVLDPYPAKL SGLRGKNIFL VAATLRPETM FGQTNCWVRP DMKYIGFETV
321     NGDIFICTQR AARNMSYQGF TKDNGVVPVV KELMGEEILG AALSAPLTSY KVVYALPMLT IKEDKGTGVV TSVPSDSPDD
401     IAALRDLKKK QALRGKYGIR DEMVLPFEPV PIIEIPGYGN LCAPFICDEL KIQSQNDREK LAEAKERVYL KAFYEGVMLV
481     DGFKGQKVQD VKKCIQKMMV DNDEAMIYME PEKQVMSRSA DECVVALCDQ WYLDYGEVDW KKQTSECLKQ LETFCEETRR
561     NFEATLGWLQ EHACSRTYGL GTRLPWDEQW LIESLSDSTI YMAYYTVAHL LQGGNLRGQG ESPLGIRAHQ MSKEVWDYIF
641     FKAAPFPKTE IPKEKLDKLK EEFEFWYPVD LRVSGKDLVP NHLSYYLYNH VAMWSDQKEK WPVAVRANGH LLLNSEKMSK
721     STGNFLTLSQ AVDKFSADGM RLALADAGDT VEDANFVEAM ADAGILRLYT WVEWVKEMIA NRDSLRSGPA NTFNDRVFAS
801     EMNAGIKKTD QNYERMLFKE ALKTGFFEFQ AAKDKYRELA IEGMHRELVF RFIEVQTLLL APICPHLCEH IWSLLGKPDS
```

```
881     IMKASWPEAG PVDEILIGSS QYLMEAAHDL RLRLKGYMAP VKGKKGTKEP SQKPSHCTIY VAKSYPPWQH TTLSVLRQHY
961     QVTGGQLPDN KVIASELNAL PELKKYMKKV MPFVAMIKEN LEKNGSRVLD LELEFDERAV LMENIVYLTN SLELDHIEVK
1041    FASEAEDKIK EECCPGKPFS VFRTEPSVSV FLVNPQPSNG HFSTKIEIRQ GDNRETVIRR LMKMDRGIKD LSKVKLMRFD
1121    DPILGPRRVP VLGKEEAEKT AIPEQAVFHI NLAERRVCVT ESGLTRDIGD TIVYLVH
```

5.312 Chain A, Chicken Citrate Synthase Complex With Trifluoroacetonyl-Coa And Citrate

Protein Accession	gi|2780941
Mean Expression Ratio	1.03
Median Expression Ratio	1.03
Credible Interval	(0.793, 1.33)
Associated Peptides	1
Associated Spectra	1
Coverage	0.0366

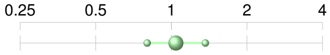

A	2.5	50	97.5	Sequence
**1**	**0.8**	**1.0**	**1.4**	**GLIYETSVLDPDEGIR**

```
1       ASSTNLKDVL ASLIPKEQAR IKTFRQQHGN TAVGQITVDM SYGGMRGMKG LIYETSVLDP DEGIRFRGFS IPECQKLLPK
81      AGGGEEPLPE GLFWLLVTGQ IPTPEQVSWV SKEWAKRAAL PSHVVTMLDN FPTNLHPMSQ LSAAITALNS ESNFARAYAE
161     GINRTKYWEF VYEDAMDLIA KLPCVAAKIY RNLYRAGSSI GAIDSKLDWS HNFTNMLGYT DPQFTELMRL YLTIHSDHEG
241     GNVSAHTSHL VGSALSDPYL SFAAAMNGLA GPLHGLANQE VLLWLSQLQK DLGADASDEK LRDYIWNTLN SGRVVPGYGH
321     AVLRKTDPRY TCQREFALKH LPSDPMFKLV AQLYKIVPNV LLEQGKAKNP WPNVDAHSGV LLQYYGMTEM NYYTVLFGVS
401     RALGVLAQLI WSRALGFPLE RPKSMSTAGL EKLSAGG
```

5.313 PREDICTED: similar to phosphoinositol 3-phosphate-binding protein-2 [Gallus gallus]

Protein Accession	gi|118083033
Mean Expression Ratio	1.03
Median Expression Ratio	1.03
Credible Interval	(0.792, 1.31)
Associated Peptides	1
Associated Spectra	1
Coverage	0.0093

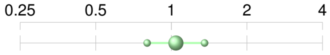

A	2.5	50	97.5	Sequence
**1**	**0.8**	**1.0**	**1.4**	**AIGPVTAEEK**

```
1       MLGELLSDLP TGWEEAYTFE GARYYVNHNE RKVTCKHPVT GQPSQDNCIF VVNEQTAAAM TSEEKKERPV SMICEATNYN
81      LTSDYAAHPM SPVGRTSRSS KKVHNFGKRS NSIKRNPNAP VVRRGWLYKQ DSTGMKLWKK RWFVLSDLCL FYYRDEKEEG
161     ILGSILLPSF QISVLSPEDH INRKYAFKAA HPNMRTYYFC TDTGKEMELW MKAMTDAALV QTEPVKRVEK FTTENTPPRE
241     ANNISNHRVL IKPEAQNNQK NREVNKNEEK KALEAEKYGF QKVGQDKPLT KINSVKLNPL GSEYRTLPIT TVQAGHFRPV
321     HLNGAENKAV GIVLADTGDG GQHNAMQSHM ESERVMQRTN SMLQLEQWIK IQRGKGQEEE ARGVISYQTL PRNMPSHRPQ
401     ALPRYPEGYR TLPRNSKARP ESLCSVTPSA YDRAIGPVTA EEKRRSMRDD TMWQLYEWQQ RQFYNKQTTL TRHSTLSSPK
481     TMINISDQTM HSIPTSPSHG SLAGFQGYSP QRTYRSEVSS PVQRGDVTID RRHRAHHTKH VYMPDRRSMP AVLSLQPVTP
561     QSLQGKTLTQ EECRGTLYKY RTEELDIDAK LSRLCEQDKV VQALEEKLQQ LHKEKYTLEQ ALLSASQEIE MNADNPAAIQ
641     NVVLQRDDLQ NGLLGTCREV SRATAELERA WREYDKLEYD VTATRNHMQE QLDRLGEIQT ESAGIQRAQI QKELWRIQDV
721     MEGLSKHKQQ RSSSDAGIMG SRTFSAIKYK NEGPDYRLYK SEPELTTVAE VDESNGEEKT EQMLESESAA KGSHFPVGVV
801     PPRTKSPTPE SSTIASYVTL RKNKKIDLRT ERPRSAVEQL CLAESTRPRM TVEEQMERIK RHQQACLREK RKGLNIIGVS
881     DQSPSQSLSF VRDNPFKSAQ NRKKDDTVSS HIKDLESTTK DNNLKQNNES PEEEIVQVKH DGEQEHKSGF TKELTKTDDF
961     LSDTHVVSDS KEVNNEEKAE KEKNNKVEET HDGDISLQSV TTVANHKPKT SSEESEAVSV QEQKGIIASF ELATQSSKGN
1041    QATAVKSLPS SPESSLSPAS STQPQLTEGS HFMCV
```

5.314 alpha-actinin [Gallus gallus]

Protein Accession	gi|211083
Mean Expression Ratio	1.03
Median Expression Ratio	1.03
Credible Interval	(0.794, 1.33)
Associated Peptides	1
Associated Spectra	1
Coverage	0.0224

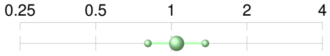

A	2.5	50	97.5	Sequence
**1**	**0.8**	**1.0**	**1.4**	**ASFNHFDRDHSGTLGPEEFK**

```
1     MDHHYDPQQT NDYMQPEEDW DRDLLLDPAW EKQQRKTFTA WCNSHLRKAG TQIENIEEDF RDGLKLMLLL EVISGERLAK
81    PERGKMRVHK ISNVNKALDF IASKGVKLVS IGAEEIVDGN VKMTLGMIWT IILRFAIQDI SVEETSAKEG LLLWCQRKTA
161   PYKNVNIQNF HISWKDGLGF CALIHRHRPE LIDYGKLRKD DPLTNLNTAF DVAEKYLDIP KMLDAEDIVG TARPDEKAIM
241   TYVSSFYHAF SGAQKAETAA NRICKVLAVN QENEQLMEDY EKLASDLLEW IRRTIPWLEN RAPENTMQAM QQKLEDFRDY
321   RRLHKPPKVQ EKCQLEINFN TLQTKLRLSN RPAFMPSEGK MVSDINNAWG GLEQAEKGYE EWLLNEIRRL ERLDHLAEKF
401   RQKASIHESW TDGKEAMLQQ KDYETATLSE IKALLKKHEA FESDLAAHQD RVEQIAAIAQ ELNELDYYDS PSVNARCQKI
481   CDQWDNLGAL TQKRREALER SEKLLETIDQ LYLEYAKRAA PFNNWMEGAM EDLQDTFIVH TIEEIQGLTT AHEQFKATLP
561   DADKERQAIL GIHNEVSKIV QTYHVNMAGT NPYTTITPQE INGKWEHVRQ LVPRRDQALM EEHARQQQNE RLRKQFGAQA
641   NVIGPWIQTK MEEIGRISIE MHGTLEDQLN HLRQYEKSIV NYKPKIDQLE GDHQQIQEAL IFDNKHTNYT MEHIRVGWEQ
721   LLTTIARTIN EVENQILTRD AKGISQEQMN EFRASFNHFD RDHSGTLGPE EFKACLISLG YDIGNDAQGE AEFARIMSIV
801   DPNRMGVVTF QAFIDFMSRE TADTDTADQV MASFKILAGD KNYITVDELR RELPPDQAEY CIARMAPYNG RDAVPGALDY
881   MSFSTALYGE SDL
```

5.315 C-type lectin-like receptor [Gallus gallus]

Protein Accession	gi|77745589
Mean Expression Ratio	0.973
Median Expression Ratio	0.973
Credible Interval	(0.75, 1.26)
Associated Peptides	1
Associated Spectra	1
Coverage	0.0558

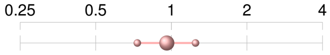

A	2.5	50	97.5	Sequence
**1**	**0.73**	**0.96**	**1.2**	**MEDEEGYTALNLR**

```
1     MEDEEGYTAL NLRTPASDIT DGYLSNKKCP TFSTPANCVT VDRVSASSAI RQPATLAFFA LSLVLLTGLV VLLSLFFQTY
81    KDPEEGKKLQ AMREALCFER RANNETECAL CPARWKSSEA GSCFYVSKQK KTWKESQEFC STRNSTLLVL KDKVKMVSLP
161   YDSQFYWVGL SYISERNGWF WEDGTALSTE AKTWTVLREH TFCASLYGQI IYASNSCSTK QSWICEKGAV QFA
```

5.316 PREDICTED: similar to U-snRNP-associated cyclophilin [Gallus gallus]

Protein Accession	gi|50759473
Mean Expression Ratio	0.971
Median Expression Ratio	0.973
Credible Interval	(0.754, 1.25)
Associated Peptides	1
Associated Spectra	1
Coverage	0.0339

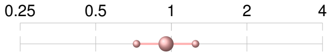

A	2.5	50	97.5	Sequence
**1**	**0.73**	**0.95**	**1.2**	**HVVFGK**

```
1     MAVLAANPNN PVVFFDVTIG GQEVGRMKIE LFADVVPKTA ENFRQFCTGE FRKDGVPIGY KGSTFHRVIK DFMIQGGDFV
81    NGDGTGVASI YRGPFADENF KLKHSAPGLL SMANSGPSTN GCQFFITCSK CDWLDGKHVV FGKIVDGLLV MRKIENVPTG
161   PNNKPKLPVV ISQCGEM
```

5.317 PREDICTED: similar to C219-reactive peptide (FLJ39207) [Gallus gallus]

Protein Accession	gi|118087853
Mean Expression Ratio	0.972
Median Expression Ratio	0.973
Credible Interval	(0.765, 1.23)
Associated Peptides	1
Associated Spectra	2
Coverage	0.0147

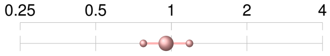

A	2.5	50	97.5	Sequence
2	0.77	0.95	1.2	MSVPDLGPAVASLISSGPR

```
1       MTIFEERIEK SSPEEKSKSA AQNTNAENST QQGTAHTDNE DSDRNLGTSS ARETTLRLEL QSEEPDAEED PDLKQEEELL
81      EDENAASAKL LQARLANIQG NAQTTRSTNP DLGVLSEAVS GTVNPTYETG EKTNSFSEDT KEISRVQDAH EVGNKEVDKP
161     VSEDTKLDEI EHVIEDNKES SEAEEPSAVE EYDFKSPDRE DSNDFNQRKG HLPESILLGD SEEIQNSEDT RNAHQHSAHF
241     PSPADMSAAT NDTVTDYSES VKRLTITRDF LDEKRVIRLQ KYLGLQNVIR IEAMFQDMKI EMELARKASQ NNEEIEKALD
321     QILKSSDSSI MDVVGKVLDS RVAENQEEVV KEMDLYDEES ALMDDIQELI YSLRSKYSSA SESVPLGSFL EQEEDQLHVQ
401     GAAKEAENDA APGRNLHGID ESSQKFQQLE DKRPFLPMEE EAEEEETAVN IPPEDEEATF SDNKEAEDGY GSERGLPLAD
481     PFGAADAGES AKGDTAPDHV PGVPPSLPPD PGAVPPGGGA SEGALSGAVR RLLRQLAATL PEDIRPGPDF HGLPWEPVIV
561     TALVGMATLA IIFWRTCLSV KSRMYQVTEK QLAEKIQNLL QEKTEMLDKF SECDEKIKQA KESMKVAQEQ KSILSDEIAG
641     LKDTVKELEE TNHQLDDKIK SLRTMLDTER KQNAKKQKKL SETQKSLEKF EEAFSMHSAE LSEVQIALNE SKLSEEKVKA
721     ELQHVQEENA RLKKSKEQLL KEAEGWSERH TELTEQIKLY RKSQKDIEEA LAYKENEIEV LTNCIMQLKQ LDTDSASEAK
801     DGEGLEWSTK DDLANGELPD NEKMKTQIKQ MMDASRVKTM LSLVEEDRNS LQSKLSDEVA ARHELEEQIK KLEHDSSSLQ
881     SAKARLENEC KTLQQKVEIL GELYQQKEMA LQKKLTQEEY ERQEKEQKLS AADEKAVLAI EEVKVYKQRI QDMEEELQKT
961     ERSYKNQIAA HEKKAHDNWL IARSAERALA EEKREAANLR QKLMEVNQKT IMLQRPLIVK PTPGRPDRQV PPRRVPLSRD
1041    GSFGPSPVSG GNPSPTQMME VPSRPLSAPQ REGSRAEFGT VVDGPPAPRR PPELPGRMSV PDLGPAVASL ISSGPRTSSP
1121    ATAKDRAVNV GPKGPPSFPG TPVMTSPVLG PPPPPPVNYG PPPAPFPGHY GPGPRPLPVP LVCGAPLPPP AARDFLPGPS
1201    LGIRDLPPGP LPPLPDPRSY RRGHPHFRPP GPPGPRDYPP GPPLPPPASR DYAPSRSRDL PPAGPRDYPA GPAPPPAGSK
1281    DYAQPPVQKP
```

5.318 PREDICTED: similar to chaperonin-containing TCP-1 complex gamma chain [Gallus gallus]

Protein Accession	gi|118122977
Mean Expression Ratio	1.03
Median Expression Ratio	1.03
Credible Interval	(0.798, 1.32)
Associated Peptides	1
Associated Spectra	1
Coverage	0.0327

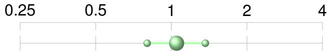

A	2.5	50	97.5	Sequence
1	0.8	1.0	1.4	TAVETAVLLLR

```
1       MVTEVLSNPG HAMSLPQLFV PSSVLSSREL IWCSVRAELL LSEQRGSLWE RSPRLLVEHV VPLTSPFSLC QIPGGFSEDS
81      CVLRGIMVNK DVTHPRMRRL IKNPRIVLLD CSLEYKKGES QTDIEITREE DFARILQMEE EYIQQICEDL LRVKPDLVIT
161     EKGISDLAQH YLMRANISAV RRVRKTDNNR IARIQPFVLI TNPRGWAEPK PSSEWEIPLL VVALGVMALC GGAGLYQRWG
241     EVSAQPPLCS AISLSSLQAK HTQEGSQTWG VNGESGALAD MKELGVWEPL AVKLQTYKTA VETAVLLLRI DDIVSGHKKK
321     GEEHSKAPAP TEAAQE
```

5.319 myomesin (M-protein) 2, 165kDa [Gallus gallus]

Protein Accession	gi|45384088
Mean Expression Ratio	1.03
Median Expression Ratio	1.03
Credible Interval	(0.797, 1.32)
Associated Peptides	1
Associated Spectra	1
Coverage	0.0124

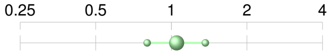

A	2.5	50	97.5	Sequence
1	0.8	1.0	1.4	TLDLSGQAFDDALTEFQR

```
1     MSSVAVPFYQ RRHKHFDQSY RNIQTRYVLE EYAARKAASR QAAHYESTGL GKTTCRLCAR RARSLAHEAM QESRKRTHEQ
81    KSHASDEKRI KFASELSSLE REIHMARHHA REQLDRLAIQ RMVEENMALE RHVVEEKISR APEILVRLRS HTVWEKMSVR
161   LCFTVQGFPS PVVQWYKNEE LITPASDPAK YSVENKYGVH VLHINRADFD DSATYSAVAT NIHGQASTNC AVVVRRFRES
241   EEPHPAGIMP FHLPLSYDVC FTHFDVQFLE KFGVTFATEG ETLTLKCSVL VTPELKRLRP RAEWYRDDVL IKDSKWTKLY
321   FGEGQAALSF THLNKDDEGL YTLRMVTKGG VNECSAFLFV RDADALIAGA PGAPMDVKCH DANRDYVIVT WKPPNTTSQN
401   PVIGYFVDKC EVGLENWVQC NDAPVKICKY PVTGLYEGRS YIFRVRAVNS AGISRPSRVS EPVAALDPVD LERTQTVHVD
481   EGRKIVISKD DLEGDIQIPG PPTNVHASEI SKTYVVLSWD PPVPRGREPL TYFIEKSMVG SGSWQRVNAQ VAVKSPRYAV
561   FDLAEGKPYV FRVLSANKHG ISDPSEITEP IQPQDIVVVP SAPGRVVATR NTKTSVVVQW DKPKHEENLY GYYIDYSVVG
641   SNQWEPANHK PINYNRFVVH GLETGEQYIF RVKAVNAVGF SENSQESEAI KVQAALTCPS YPHGITLLNC DGHSMTLGWK
721   APKYSGGSPI LGYYIDKREA NHKNWHEVNS SVISRTIYTV EDLTEDAFYE FKIAAANVVG IGHPSDPSEH FKCKAWTMPE
801   PGPAYDLTVC EVRNTSLVLL WKAPVYEGKS PITGYLVDYK EVDTEDWITA NEKPTSHRYF KVTDLHQGHT YVFKVRAVND
881   AGVGKSSEIS EPVFVEASPG TKEIFSGVDE EGNIYLGFEC KEATDASHFL WGKSYEEIED SDKFKIETKG DHSKLYFKHP
961   DKSDLGTYCI SVSDTDGVSS SFVLDEEELE RLMTLSNEIK NPTIPLKSEL AYEVLDKGEV RFWIQAESLS PNSTYRFVIN
1041  DKEVENGDRH KISCDHSNGI IEMVMDKFTI DNEGTYTVQI QDGKAKNQSS LVLIGDAFKA ILAESELQRK EFLRKQGPHF
1121  SEFLYWEVTE ECEVLLACKI ANTKKETVFK WYRNGSGIDV DEAPDLQKGE CHLTVPKLSR KDEGVYKATL SDDRGHDVST
1201  LELSGKVYND IILALSRVSG KTASPLKILC TEEGIRLQCF LKYYNEEMKV TWSHRESKIS SGEKMKIGGG EDVAWLQITE
1281  PTEKDKGNYT FEIFSDKESF KRTLDLSGQA FDDALTEFQR LKAAAFAEKN RGKVIGGLPD VVTIMDGKTL NLTCTVFGNP
1361  DPEVVWFKND KALELNEHYL VSLEQGKYAS LTIKGVTSED SGKYSIYVKN KYGGETVDVT VSVYRHGEKI PEVNQGQLAK
1441  PRLIPPSSST
```

5.320 chaperonin containing TCP1, subunit 5 (epsilon) [Gallus gallus]

Protein Accession	gi|60302774
Mean Expression Ratio	0.975
Median Expression Ratio	0.974
Credible Interval	(0.757, 1.26)
Associated Peptides	1
Associated Spectra	1
Coverage	0.0314

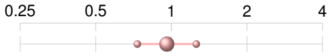

A	2.5	50	97.5	Sequence
1	0.73	0.96	1.3	WVGGPEIELIAIATGGR

```
1     MSAMGTLAFD EYGRPFLILK DQERKTRLMG LEALKSHIMA AKAVASTLRT SLGPNGLDKM MVDKDGEVTV TNDGATILNM
81    MDVDHQIAKL MVELAKSQDD EIGDGTTGVV VLAGALLEQA EQLLDRGIHP IRIADGYEQA ARIAVEHLDK ISDSFPVDPQ
161   NIEPLIQTAK TTLGSKVVNR CHRQMAEIAV NAVLTVADME RKDVDFELIK VQGKVGGRLE DTQLVKGVIV DKDFSHPQMP
241   KELKDAKIAI LTCPFEPPKP KTKHKLDVTS VDDYKALQKY EKEKFEEMVK QIKDTGANLA ICQWGFDDEA NHLLLQNELP
321   AVRWVGGPEI ELIAIATGGR IVPRFCELTP EKLGFAGIVR EISFGTTKDR MLVIEQCQNS RAVTIFIRGG NKMIIEEAKR
401   SLHDALCVIR NLVRDNRIVY GGGAAEISCA LAVSEAADKC PSLEQYAMRA FADALEVIPM ALSENSGMNP IQTMTEVRAR
481   QVKENNPALG IDCLQKGTND MKQQHVIETL IGKKQQISLA TQVVRMILKI DDIRRPGESE E
```

5.321 coatomer protein complex, subunit alpha [Gallus gallus]

Protein Accession	gi|71897175
Mean Expression Ratio	1.03
Median Expression Ratio	1.03
Credible Interval	(0.794, 1.33)
Associated Peptides	1
Associated Spectra	1
Coverage	0.0106

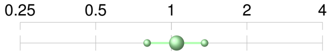

A	2.5	50	97.5	Sequence
1	0.8	1.0	1.4	DADSQNPDAPEGK

```
1     MLTKFETKSA RVKGLSFHPK RPWILTSLHN GVIQLWDYRM CTLIDKFDEH DGPVRGIDFH KQQPLFVSGG DDYKIKVWNY
81    KLRRCLFTLL GHLDYIRTTF FHHEYPWILS ASDDQTIRVW NWQSRTCVCV LTGHNHYVMC AQFHPSEDLV VSASLDQTVR
161   VWDISGLRKK NLSPGAVESD VRGITGVDLF GTTDAVVKHV LEGHDRGVNW AAFHPTMPLI VSGADDRQVK IWRMNESKAW
241   EVDTCRGHYN NVSCAVFHPR QELILSNSED KSIRVWDMSK RTGVQTFRRD HDRFWVLAAH PNLNLFAAGH DGGMIVFKLE
321   RERPAYAVHG NMLYYVKDRF LRQLDFNSSK DVAVMQLRSG SKFPVFNMSY NPAENAVLLC TRASNLENST YDLYTIPKDA
401   DSQNPDAPEG KRSSGLTAVW VARNRFAVLD RMHSILIKNL KNEITKKVQV PNCDEIFYAG TGNLLLRDAD SITLFDVQQK
481   RTLASVKISK VKYVIWSADM SHVALLAKHA IMICNRKLES LCNIHENIRV KSSAWDESGV FIYTTSNHIK YAVTTGDHGI
561   IRTLDLPIYV TRVKGNNVYC LDRECRPRVL TIDPTEFKFK LALINRKYDE VLHMVRNAKL VGQSIIAYLQ KKGYPEVALH
641   FVKDEKTRFS LALECGNIEI ALEAAKALDD KNCWEKLGEV ALLQGNHQIV EMCYQRTKNF DRLSFLYLIT GNLEKLRKMM
721   KIAEIRKDMS GHYQNALYLG DVAERVRILR NCGQKSLAYL TAATHGLDEE AENLKETFDP EKETIPDIDP NAKLLQPPAP
801   VMPLDTNWPL LTVSKGFFEG TIASKGKGGA LAADIDIDNV GTEGWGEDAE LQLDEDGFVD AGEGFGEEGL GKGQEEGGGW
```

```
881     EVEEDLDLPP ELDVPAGPAG GAEDGFFVPP TKGTSPAQVW CNNSQLPVDH ILAGSFETAM RLLHDQVGVT NFGPYKQLFL
961     QTYARGRTTY QALPCLPTMY GYPHRNWKEA GLKNALPAVG LKLNDLIQRL QLCYQLTTAG KFEEAVEKFR SILLSVPLLV
1041    VDNKQEIAEA QQLIAICREY IVGLSMEIER KKLPKETLEH QKRICEMAAY FTHSNLQPVH MILVLRTALN LFFKLKNFKT
1121    AATFARRLLE LGPKPEVAQQ TRKILSACEK NPTDTYQLNY DMHNPFDICA ASYRPIYRGK PVEKCPLSGA CYCPEFQGQI
1201    CRVTTVTEIG KDVIGLRISP LQFR
```

5.322 heat shock protein 70 [Gallus gallus]

Protein Accession	gi|30962014
Mean Expression Ratio	0.974
Median Expression Ratio	0.974
Credible Interval	(0.847, 1.12)
Associated Peptides	6
Associated Spectra	16
Coverage	0.156

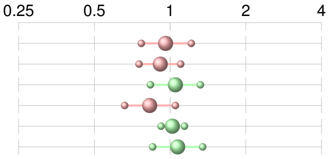

A	2.5	50	97.5	Sequence
1	0.77	0.96	1.2	LYQGAGGAGAGGSGGPTIEEVD
2	0.75	0.91	1.1	QTQTFTTYSDNQSSVLVQVYEGER
1	0.83	1.1	1.3	GQIQEIVLVGGSTR
1	0.66	0.83	1.1	VQNAVITVPAYFNDSQR
10	0.92	1.0	1.1	FEELNADLFR
1	0.85	1.1	1.3	NQMAEKEEYEHK

```
1       MSGKGPAIGI DLGTTYSCVG VFQHGKVEII ANDQGNRTTP SYVAFTDTER LIGDAAKNQV AMNPTNTIFD AKRLIGRKYD
81      DPTVQSDMKH WPFRVVNEGG KPKVQVEYKG EMKTFFPEEI SSMVLTKMKE IAEAYLGKKV QNAVITVPAY FNDSQRQATK
161     DAGTITGLNV MRIINEPTAA AIAYGLDKKG TRAGEKNVLI FDLGGGTFDV SILTIEDGIF EVKSTAGDTH LGGEDFDNRM
241     VNHFVEEFKR KHKRDIAGNK RAVRRLRTAC ERAKRTLSSS TQASIEIDSL FEGIDFYTSI TRARFEELNA DLFRGTLEPV
321     EKALRDAKLD KGQIQEIVLV GGSTRIPKIQ KLLQDFFNGK ELNKSINPDE AVAYGAAVQA AILMGDKSEN VQDLLLLDVT
401     PLSLGIETAG GVMTALIKRN TTIPTKQTQT FTTYSDNQSS VLVQVYEGER AMTKDNNLLG KFDLTGIPPA PRGVPQIEVT
481     FDIDANGILN VSAVDKSTGK ENKITITNDK GRLSKDDIDR MVQEAEKYKA EDEANRDRVG AKNSLESYTY NMKQTVEDEK
561     LKGKISDQDK QKVLDKCQEV ISWLDRNQMA EKEEYEHKQK ELEKLCNPIV TKLYQGAGGA GAGGSGGPTI EEVD
```

5.323 hypothetical protein LOC422936 [Gallus gallus]

Protein Accession	gi|61097983
Mean Expression Ratio	1.03
Median Expression Ratio	1.03
Credible Interval	(0.798, 1.33)
Associated Peptides	1
Associated Spectra	1
Coverage	0.0265

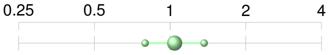

A	2.5	50	97.5	Sequence
1	0.8	1.0	1.4	LVETTDGGGPQPPDSVR

```
1       MGFAEDKVYN HILRNMSRFC DIHVASLVDS LSCLTDADRD ELHTRQDMRG IRATAYKFYQ HLKCRKGWVM DLINALHQNN
81      AGHLAEELQR VYDCYQAPPP GASAPPSASA PAAWPAVSSS SVPKPFPGPN SAAEAPMAEP PRYNPSAGGR PPLSPAAATT
161     ATSAVSSDVS STELDARAPV QETPPERKSP QPPLMTSTVC DGKEKPLPYP TSEQQEAVET PGTSSTVLPS VSPEQGQEWL
241     SHRHPVCVDN GCFGNANHLQ RGMPNLDLSR SLPPRDLSAA PGPEQTRNEP QEDVYISSEL PVRLVETTDG GGPQPPDSVR
321     TQEERAVHSF KHDGPPSSFV DVRNPLLIQQ QFDAEQKQIE REHEGGGDVL KETTTSVSTS APQDISPFCD TSLKPPVREK
401     MLPEEKAASS TPSMPGKEKV LSASVASLSG MNAAGSFEGA AGRTSSQVSS AASIWASHDN EEEDVELSKP GALQSVVGES
481     PKAAARYPGS PSSNTSSHLG LSSDPIMVST DSLRPGEAQS TANSGLWSAT PAVHADPGGE EAAGVSPYPP LSWADPSVGT
561     HEVHVEHHPS APLTAGNDVP DEAVPYGDSP DSNKGSNAAN NSSHAEVPTS GDSNGPSLLY ILPAVGIALI SVFLVYTRLQ
641     K
```

5.324 PREDICTED: similar to RSK-like protein [Gallus gallus]

Protein Accession	gi|118087900
Mean Expression Ratio	1.03
Median Expression Ratio	1.03
Credible Interval	(0.79, 1.33)
Associated Peptides	1
Associated Spectra	1
Coverage	0.00698

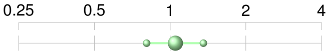

A	2.5	50	97.5	Sequence
1	0.8	1.0	1.4	TQQTFILK

```
1       MLLSEDKWVW QEIYGHVIRF LSDQSREGYI SRPRASFSAG RTSLALLGRG SPKRRAGGRG QARHRPGGAG RSGAPLPPRS
81      GPVAAAGSML SPRERGGELA RFYTVTEPRR HPRGHTVYKV TARIVSRKNP EDVQEIVVWK RYSDFKKLHK DLWQIHKNLC
161     RHTELFPPFA KAIVFGRFDE TVIEERRQCA EDLLQFSANI PALYNSKQLE EFFKGGEVHD GSELIGPVEP LSDSLTDNLS
241     DCSSEGFSSD SDLISLTVDV DSLAELDDGM ASNHSSPSRA VGLCLTSEPP IQSTVASEQE WSKPEGERES HGLFSGSLKP
321     KPGKQDYLEK AGELIKLALK KEEEEDYETA FSFYRKGVDL LLEGVQGESS PTRREAVKRK TAEYLMRAEK ISTLYHKSSE
401     DASVSMPPGS LSSRPSWNLR SPAEELKAFR VLGVIDKVLL VMDTRTQQTF ILKGLRKSSE YSRSRTTIIP RCVPNMVCLH
481     KYIISEESVF LVLQHAEGGK LWSYVSKFLN TSPEQSFEIP EPKTFSSTKI HLERPTPSPK EISSSTDSRE SYGENMLKVV
561     PLRSSLTPSS QDDSSNQEDG QESSKWMDSA SSSEEECTTS YLTLCNEYGQ EKIDSGSLNE EPVVKGESQS LNIKEKSSLQ
641     KHAVVSSASF SSQVPSQELK LFIDDAESEI ASPTRILDSL TKSKNSPMEL FRIDSKDSTS ELLGLDLGEK LYNLKSEPLK
721     PLFSVPGHDR SLDALESKMG VRAQDTVSRG SNDSVPVISF KDAAFDDVSS VDEGRPDLLI NLPSMSDGTE EAAASRPAKT
801     DMDVLEVKLL ETPDVLQLNS SAEQCKAFNQ EPNHVTPQVG DPSPKEVNGQ SGVTQGALGT LFTSDPEVGS SEDGALFQGP
881     GACSINMTSK EESVFVSNLL SGAQDVSLDR DTLRNEAVLL FSDQTEDFGK EETNLSLLPA AESEQTAPKR EDKIAVAGEK
961     DIHQIFQDLD ERLAITSRFY IPEDCIQRWA AEMVVALDAL HREGIVCRDL NPNNILLNDR GHIQLTYFSR WREVEDSCDN
1041    DAIERMYCAP EVGAILEETE ACDWWSLGAI LFELLTGKTL VECHPSGINT HTSLSIPDHV SKEARSLIQQ LLQFNPAERL
1121    GAGVAGVEDI KSHPFFALIE WADLVK
```

5.325 PREDICTED: similar to yeast sds22 homolog [Gallus gallus]

Protein Accession	gi|118094949
Mean Expression Ratio	0.974
Median Expression Ratio	0.974
Credible Interval	(0.755, 1.25)
Associated Peptides	1
Associated Spectra	1
Coverage	0.0268

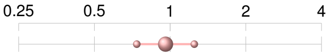

A	2.5	50	97.5	Sequence
**1**	**0.74**	**0.96**	**1.2**	**ELDLYDNQIR**

```
1       MAAGSGEGRT EMMEVDKRIE SEESEDEGKK QAVRLVTDLS QQSLRDEQNG EAETPVDMET ISLDPEAEDV DLNHFRIGKI
81      EGFEVLKKVK TLCLRQNLVK RIENLEQLQT LRELDLYDNQ IRKIENLEAL VDLEVLDISF NVLRHIEGLD QLTQLKKLFL
161     VNNKISKIEN LSNLQLLQML ELGSNRIRAI ENIDTLTNLD SLFLGKNKIT KLQNLDALTN LTVLSIQSNR LTKIEGLQSL
241     VNLRELYLSN NGIEVIEGLE NNNKLTMLDI ASNRIKKIEN ISHLTELQEF WMNDNLVESW SDLDELKGAK NLETVYLERN
321     PLYRAFRDRD GNLTLTYWYL LAVRLGFLIM CQRQHSLHWS WLSPGKLDLL TAG
```

5.326 PA polymerase protein [Influenza A virus (A/Chicken/Italy/5945/95(H3N2))]

Protein Accession	gi|18091834
Mean Expression Ratio	1.03
Median Expression Ratio	1.03
Credible Interval	(0.791, 1.32)
Associated Peptides	1
Associated Spectra	1
Coverage	0.0783

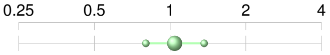

A	2.5	50	97.5	Sequence
**1**	**0.8**	**1.0**	**1.4**	**XRFEIIEGR**

```
1       NPXIVELAEK AMKEYGENPK IETNKFAAIC THLEVCFMYS DFHFIDEKGE SIIVESDDPN ALLKXRFEII EGRDRTMAWT
81      MVNSICNXTR IEKPKFLPDL YDYKENRFXE IGVTR
```

5.327 PREDICTED: similar to palladin [Gallus gallus]

Protein Accession	gi|118089852
Mean Expression Ratio	0.975
Median Expression Ratio	0.974
Credible Interval	(0.75, 1.26)
Associated Peptides	1
Associated Spectra	1
Coverage	0.00667

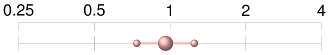

A	2.5	50	97.5	Sequence
**1**	**0.74**	**0.96**	**1.2**	**VTGNPKPK**

```
1     MQDRSCGQSL PLSLMQKETY CAERGIKKGH PKKMSEASSR HDFFYGSLSG IQNGEFSNEL SAFLTQEEIC KSLDIARESI
81    ANSMKEDQNI APELTHFNTS SASEHASFQD AKLHEQDALQ RSQASSLTSS SASHIPPVNQ KEQFTSPKMA SGFVTVSRKQ
161   ESTSPLLAAS PSFIRSLKHS GKRASGVPTT KSESASQGEV PFRNKLCDKA ATFIEELSSI FREAAKTRGR SPDGDSSPDS
241   GYLSPKKKQS TLSTSKNKDF NKPHQETEPG PKLSGVDFNG EPCSEKESIT QSEMVLCHPF INAEDNQSSP PQFTQKLRSQ
321   EVAEGNKVLL ECRVAGNPVP DVRWFCEGKE LQNSPDIQIH SGSGGLHSLI IAEAFEDDTG RYTCLASNSL GSDSTSAEIF
401   IEGASSTDSE SESLIFKSKS GAMPQAQKKT TSVSLTIGSS SPKSGVTTAV IQPISVPSQQ VQSPTSYLHH LDGSKPINSA
481   LIFTKELQNS TASEGQVVVL ECRVRGPPPI HVKWFRQGIE IQDSPDFRIL QKKPRSATEP EEICTLVIAE TFPEDSGLFT
561   CTATNEYGSV TSSAQLTVCS ANSENSSHES LTRKSSSDDF HHFPPLPPTE FSSLELSPTK HMETHQINNT ELRSGVTALQ
641   LDLSSSEEKT NGIHPIHGVN GMINSKPNGD KPISSPAVLL SPAKEPPPVL AKPKLGFPKK TGRTARIASD EEIQGSKDAV
721   IQDLERKLRF KEDLLNNGQP RLTYEEKMAR RLLGADSAAT VFNIQEPEEE PAIQEYKVSS FEQRLISEIE YRLERSPVEE
801   SDDEVQHGDE PIDNSASPYF EIKLKHYKVF EGMPVTFTCK VTGNPKPKIY WFKDGKQISK RSDHYRIQRE PDGTCSLHTA
881   ASTLDDDGNY TIMAANPQGR ISCTGRLMVQ AVNQRGRSPW TPPGQPHIRR PRSRSRDSGD ENEPIQERFF RPHFLQAPGD
961   LTVQEGKLCR MDCKVSGLPT PDLSWQLNGR PIRPDSSHKM LVRENGVHSL IIEPVTARDA GIYTCVASNR AGENTFSLEL
1041  IVAAKEVHKA PVFIEKLQNT GVTEGFPVRL ECRISGEPSP QIFWKKENES ITHNTDRVSM HQDNYGYICL LIQGATKEDA
1121  GWYTVSAKNE AGIVSCTARL DVYTQWQQPP QTTKPKKVRP STSRYAALCD QGLDIKAAFQ PEANPVHLTM QSGLVESDDL
```

5.328 PREDICTED: similar to putative acetyltransferase [Gallus gallus]

Protein Accession	gi|118089899
Mean Expression Ratio	0.978
Median Expression Ratio	0.974
Credible Interval	(0.758, 1.27)
Associated Peptides	1
Associated Spectra	1
Coverage	0.0092

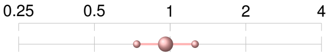

A	2.5	50	97.5	Sequence
**1**	**0.74**	**0.96**	**1.3**	**KAQLEEEKK**

```
1     MPSVSLPPKE NALFKRILVS GGREGGQAAP RGRRSLCCPA EGRGSLGTAG RTAPGLSVRG PLLPAGAAAF LAPWSSDCCA
81    RSRAGLAGGR RLVEEEVRSG GGLGVLVKFS FKAGSRWDFS RLFFRVLAGL QRCYEHKQYR NGLKFCKQIL SNPKFAEHGE
161   TLAMKGLTLN CLGKKEEAYE LVRRGLRNDL KSHVCWHVYG LLQRSDKKYD EAIKCYRNAL KWDKDNLQIL RDLSLLQIQM
241   RDLEGYRETR YQLLQLRPAQ RASWIGYAIA YHLLEDYEMA AKILEEFRKT QQTSPDKVDY EYSELLLYQN QVLREAGLHK
321   EALEHLCTYE KQICDKLAVE ETKGELLLQL GRLEEAVDVY KGLQERNPEN WAYYKGLEKA LKPANMMERL KIYEEAWTKY
401   PKGLVPRRLP LNFLSGEKFK ECLDKFLRMN FSKGCPPVFN TLRSLYKDKE KVAIIEELVV GYETSLRSCR LFNPNDDGKE
481   EPPTTLLWVQ YYLAQHYDKI GQPSLALEYI NAAIESTPTL IELFLVKAKI YKHAGNIKEA ARWMDEAQAL DTADRFINSK
561   CAKYMLKANF IKEAEEMCSK FTREGTSAVE NLNEMQCMWF QTECAQAYKA MNKFGEALKK CHEIERHFVE ITDDQFDFHT
641   YCMRKITLRS YVDLLKLEDV LRQHPFYFKA ARIAIEIYLK LHDNPLTDEN KEHEADTANM SDKELKKLRN KQRRAQKKAQ
721   LEEEKKNAEK EKQQRNQKKK KDDDDEEIGG PKEELIPEKL AKVEAPLEEA IKFLTPLKNL VKNKIETHLF AFEIYFRKEK
801   FLLMLQSVKR AFAIDSSHPW LHECMIHLFS SVSESKDLPD AVRTVLNQEM NRLFGATNPK NFNEAFLKKN YDSLPHRLSA
881   AKMMYYLDPS SQKRAVELAM TLDESLINRN LQTCMEVLEA LCDGSLGDCK EASETYRANC HKLFPYALAF MPPGYEEDMK
961   ITVNGDSSAE PEELANEI
```

5.329 PREDICTED: similar to KIAA0316 protein [Gallus gallus]

Protein Accession	gi|118084148
Mean Expression Ratio	1.02
Median Expression Ratio	1.03
Credible Interval	(0.791, 1.31)
Associated Peptides	1
Associated Spectra	1
Coverage	0.00973

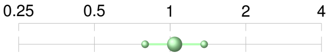

A	2.5	50	97.5	Sequence
**1**	**0.8**	**1.0**	**1.4**	**AKFGTISARDSQR**

```
1     MEEAEMNPSE KLATSFKVIW FTCVGKGAIA MRRRTDHRNK SSGWPHPSGT WSLNQGTPYG WEMTANRDGR DYFINHMTQS
81    ATFEDPRIES CQITPPAPRK VEMRRDPVLG FGFVAGSEKP VVVRSVTPGG PSEGKLIPGD QIIMINDEPV STAPRERVID
161   LVRSCKESIL LTVVQPYPSP KSAFISAAKK ARLKSNPVKV RFSEEVIING QVSETVKDNS LLFMPNVLKV YLENGQTKSF
241   RFDCSTSIKD VILTLQEKLS IKCIEHFSLM LEQRVEGSGT KLLLLHEQET LTQVTQRPSS HKMRCLFRIS FVPKDPIDLL
321   RRDPVAFEYL YVQSCNDVVQ ERFGPELKYD IALRLAALQM YIATVTTKQT QKISLKYIEK EWGLETFLPS AVLQSMKEKN
401   IKKALSHLVK ANQNLVPPGK KLSALQAKVH YLKFLSDLRL YGGRVFKATL VQGEKRSEVT LLVGPRYGIS HVINTKTNLV
481   ALLADFSHVN RIEMFTEDES SVRVELHVLD VKPITLLMES SDAMNLACLT AGYYRLLVDS RRSIFNMANK KNAGNRETGI
561   ENRGKHNLLA SEWNCVPKTT TFLADGDQET QMPFGDPKQK AIDVSESLLC QKEHRHLYIE NTYNSGGFDQ HLTKQDPTDA
641   EESRNFNQHS LLSLSGLESS KKAQDSPRGA KVSFIFGDHN LDGINPQTLG YERLLDESPE VLEKQRAIYI NNANDIKGLE
721   LSPDAESIQF ATNAVYATIN DGKIFGAAEG IEEPLLHDIC YAENTDDAED EDEVSCEEDI MVGEINRPAL LSLSGSSDDI
801   IDLTSLPPPE GDDNEDDFLL HTLNMAIAAP PPGFRDSSDE EDSQNQATQP RDDKEQASNL GSDDIPVSLI DAVPTNTEGK
```

```
881     CEKGLDDAVV STLQALEALA ASEEQQTNDN SGVAILRAYS PESSSDSGNE TNSSEMTESS ELAAAQKHSE NTARMFLTTS
961     EGYQPLVEEQ TEFPIAKSQA GGPGMKSSQP LAGCQAADLQ SKVVPSKQIL HSDNMEMEPE TMETKSVTDY FSKLHMGSLV
1041    YSCTSKRKSK MTESEVKAPS DGNATVKKQQ GTKKAETDEE LKAKFGTISA RDSQRLSTFN VERTAFRQRW YGADDGAADK
1121    PSPETANGKT FPRVPVRGKT ETDCKDEVDP EVDQDDTSGL SQGENFLSDM TPVSSAKDLN DAEDTDLSAD DHPSKLPEAE
1201    QSVARLCEYH LAKRMSSLQS EGHFSLQSSQ CSSVDAGCST GSSTCATPVE SPLCTSDIKH VISDPSMKGI AYIPADERAA
1281    ILSNHGTTYK DLHQQPEAVC HRMTVPVTHS AINAEPLFGT LREGCHRIPK IKETTV
```

5.330 PREDICTED: hypothetical protein [Gallus gallus]

Protein Accession	gi|118095665
Mean Expression Ratio	0.974
Median Expression Ratio	0.975
Credible Interval	(0.794, 1.19)
Associated Peptides	2
Associated Spectra	4
Coverage	0.0154

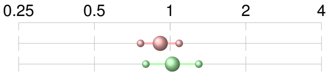

A	2.5	50	97.5	Sequence
**3**	**0.76**	**0.91**	**1.1**	**EISQDSLAAR**
**1**	**0.8**	**1.0**	**1.3**	**YEDQHPVGYDTHAHGR**

```
1       MSARAASGKS ATMEETAIWE QHTVTLHRAP GFGFGIAISG GRDNPHFQSG ETSIVISDVL KGGPAEGLLQ ENDRVAMVNG
81      VSMDNVEHAF AVQQLRKSGK NAKITIRRMK KIQIPVARQE PEPVSENEDD SYDEEIRDPR TSRGASSANR RHEKSWVRDR
161     SASRERSLSP RSDRRSVTSS QPAKPTKVTL VKSRKNEEYG LRLASHIFVK EISQDSLAAR DGNIQEGDVV LKINGTVTEN
241     MSLADAKTLI ERSKGKLKMV VQRDERATLL NVPDLSDSIH SANASERDDI SEIQSLASDH SNRSHDRPRR SRSRSPDQRS
321     EPSDHSRHSP QQPSNGSLRS REDERVTKPG AISTPVKNAD DISKTMEEVS VERTEKQTPP LPEPKPVYAQ GGQPDVDLPV
401     SPSDGPLPNS THEDGMLRPS MKLVKFRKGD SVGLRLAGGN DVGIFVAGVL EDSPAAKEGL EEGDQILRVN NVDFTNIIRE
481     EAVLFLLDLP KGEEVTILAQ KKKDVYRRIV ESDVGDSFYI RTHFEYEKES PYGLSFNKGE VFRVVDTLYN GKLGSWLAIR
561     IGKNHKEVER GIIPNKNRAE QLASVQYTLP KTAGGDRADF WRFRGLRSSK RNLRKSREDL SAQPVQTKFP AYERVVLREA
641     GFLRPVTIFG PIADVAREKL AREEPDIFQI AKSEPRDAGT DQRSSGIIRL HTIKQIIDRD KHALLDVTPN AVDRLNYAQW
721     YPIVVFLNPD SKQGVKTMRM RLCPESRKSA RKLYERAHKL RKNNHHLFTT TINLNSMNEG WYGALKEAIQ QQQNQLVWVS
801     EGKADGATSD DLDLHDDRLS YLSAPGSEYS MYSTDSRHTS DYEDTDTEGG AYTDQELDET LNDEVGTPPE SAITRSSEPV
881     REDSSGMHHE TQTYTSYASQ AQPQPNLRID SSGFKTTASQ PVYRKDPYIS EEASRQSYVL KQPAVNHPVQ RQERDPNLIY
961     ESQTQYAEKQ PSREYEQSTY RYDSTNYVDQ FSRGYDPRLH YEDRAPPYEE HWSYYDEKQP YNQPRTAYES QPPRDLDSRQ
1041    NTEESTERSY YPAQPRFEEP PPMSYDGRPR YEHAPKNFSL PQVRYEDQHP VGYDTHAHGR YKQEAQPYQS AISRSPEPKQ
1121    YFDPHVRGYE QGPPQAYNAK AGQFEPSHST SGVSLPPPAS SQTKPEVLPS NSKPLPTPPS LAEEEEDPAM KPQSVRSRVK
1201    IFERRRSPSL EKMKDPSDTS VVKPPELAPK PTLPAMSGPK PTSQSQYEHD KTTYRAPEPQ RPQVKPPEDI VRSNHYDPEE
1281    DEEYYRKQLS YFDRRNFENK PSAQVPASHH SDSTKPIHSQ NQLNFSNYSK GKPTDAEPMD RSVGDKRYEP IPQVTTPPPA
1361    PSVQYTQPQS INSPVLSLPA HHKPALSEVN SVSDPPPPQN KPAIFRSSRE DTVQSTFYPQ KSFPDKGPVN GTEQVQKTVT
1441    PSYNRFTTKP YTSAARPFER KFESPKFNHN LLPNEAQHKP ELPSKSPNSP QPILKAHSSS QPPEFDSGMD TFTVQVDKPK
1521    YQPNNVNAVP KAIPVSPSAL EDEEEEDGHT VVATARGVFN SNGGVLSSIE TGVSIIIPQG AIPEGIEQEI YFKVCRDNSI
1601    LPPLDKEKGE TLLSPLVMCG PHGLKFLKPV ELRLPHCASM TPDGWSFALK SSDSSSGDPK TWQNKSLPGD PNYLVGANCV
1681    SVLIDHF
```

5.331 PREDICTED: hypothetical protein [Gallus gallus]

Protein Accession	gi|118100576
Mean Expression Ratio	0.974
Median Expression Ratio	0.975
Credible Interval	(0.752, 1.26)
Associated Peptides	1
Associated Spectra	1
Coverage	0.105

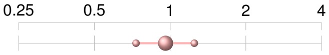

A	2.5	50	97.5	Sequence
**1**	**0.73**	**0.96**	**1.2**	**ASSAAQLSK**

```
1       MAAEVHFSPP EIPEPTLLEN VLRYGLFFGA IFQLLCVLAI ILPVSKSPEA DSVGSESKTL EAVKKPKASS AAQLSKKAKK
81      EGKKRR
```

5.332 matrin 3 [Gallus gallus]

Protein Accession	gi|45383822
Mean Expression Ratio	0.975
Median Expression Ratio	0.975
Credible Interval	(0.78, 1.22)
Associated Peptides	2
Associated Spectra	2
Coverage	0.0277

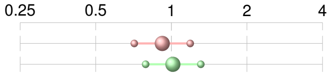

A	2.5	50	97.5	Sequence
1	0.71	0.92	1.2	AKEEKQDDTAEPSGTK
1	0.79	1.0	1.3	KPEGKPDQK

```
1       MSKSFQQSSL SRDSQGHGRE LSAGIGLLAA ATQSLNMPAS LGRMNQGTAR LASLMNLGMS SSLNQQGSHS ALSSGSTSSH
81      NLQSIFNIGS RGPLPLSSQH RGDADQATNI LASFGLSARD LDELSRYPED KITPENLPQI LLQLKRRRAE EGYGRDGRSS
161     TREPPYRVPR DDWEEKRHFR RDSFDDRGPS LNPVVDYDHG SRSQESGYYD RMDYEDDRLR DGERCRDESF YGETSHNYHK
241     FDSEYDRMGR GPGPERSLFE KKRGAPPNSN IEDFHGFLPK GYPHLCSICD MPVHSNKEWN HHINGATHGR RCQLLLEIYP
321     EWNPDSDSGH GMGDPFMLQQ STNPAPGILG PPPPPFHLGG PPVGPRGAGN GNMQGPRHMQ KGRVETSRVV HIMDFQRGKN
401     LRYQLLQLVE PFGIITNHLI LNKINEAFIE MSTTEDAQAA VEYYSTTPAL VFGKPVRVHL SQKYKRIKKP EGKPDQKTEA
481     PKPELGRVIH LSNLPHSGYS DNAVLKLAEP YGKIKNYILM RMKSQAFIEM ETREDALAMV EHCANKALWF QGRCVKVDLS
561     EKYKKLVLRI PNKGVELLKK DKTRKRTYSP DSKDSPSDKK SKTDATQKPE SGTVEDKAKE EKQDDTAEPS GTKSSEQADQ
641     DEPSLLLESE DELLVDEEEA AALLESGSSA GDDADVANLT DVTTEEKKDP ADDVTVKTEG NVVANPATKK KLKKRYVGGF
721     PRSMEGFVTL DEVGDEEDSD HQKLRKSGLA VKSAGKNDDS LAEIKVDKIE EPEQENETLE NGTKTEDNLK AEAVEASDTT
801     AAQDPEKNAH ENIDTQDEQE TKSVQEKALI PDEFRIGPYQ PNVPVGVNYV VPKTGFYCKL CSLFYTNEDV AKKTHCSSLP
881     HYQKLKKILD KMAEDYRQKK EA
```

5.333 PREDICTED: similar to FLJ39378 protein isoform 1 [Gallus gallus]

Protein Accession	gi|118098442
Mean Expression Ratio	1.03
Median Expression Ratio	1.03
Credible Interval	(0.793, 1.33)
Associated Peptides	1
Associated Spectra	1
Coverage	0.034

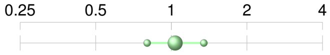

A	2.5	50	97.5	Sequence
1	0.8	1.0	1.3	NEDVEALQQQQNR

```
1       MDGGGGGVPP ALEKNAAELT VMDVYDIASA VGQEFERVID QHGCEAIARL MPKVVRVLEI LEVLVSRNHI NPEMEELRLE
81      LDRLRLERMD RIEKERKHQK ELELVEDVWR GEAQDLLTQI AQLQEENKQL MTNLSHKDIN FTEEEFQKHE GMSERERQVM
161     KKLKEVVDKQ RDEIRAKDRE LGLKNEDVEA LQQQQNRLMK INHDLRHRIT VVEAQGKALI EQKVELEAYL QTKEQEMGTL
241     RAELGKLREK LQGEDSQNGE EIQTEPNNDD CLSESEKLAM DLKDPNRPRF TLQELRDVLH ERNELKSKVF LLQEELLYYK
321     SEETEEETRP PQPTPIIQSK PSTQPESGIK RLIFTAIMPM VAAGLIPDDP TLQPIRRLVS LV
```

5.334 PREDICTED: similar to Ogfr protein [Gallus gallus]

Protein Accession	gi|118100695
Mean Expression Ratio	1.02
Median Expression Ratio	1.03
Credible Interval	(0.792, 1.32)
Associated Peptides	1
Associated Spectra	1
Coverage	0.0110

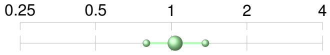

A	2.5	50	97.5	Sequence
1	0.8	1.0	1.4	QETPGKK

```
1       MAAWFGFRAE EDEEGDEASF WRYDSTWEED DDEDEDGDEG QPEGAEAAAG EEPEDSEKSA LSSPREPRQA QGQRRSNSSP
81      LFQRSFKFSG RRNWNAAIDL QRYRHRYPGL IESETDEEED MWNLSFYKNE IAFMPHGLHI EDLLESWWDN YEVLEENHSY
161     IQWLFPLREH GMNFRAKPLT CQEIEALKKS EEVMERFIRA YRLMLRFYGI ILVNQETGEL ERAENWAERF LNLNRFSHNN
241     LRITRILKCL GEMGYEHYQV HLVKFFLTET LVNETLPNVK RSALDYFLFT IRSKRKRREL VHYAWQHYKP QGSFVWGPHD
321     KLLKYRPRST KSKLHQKAEN KQETPGKKID DSVEEGQNQS PEEEQKVGDT ADLQTEANKE DTREKISECV SEGGDDEDDD
401     KTSFAQQEKD CNSEAEVQDA AENDCTKESK KRKLDANIAD NIKSGLLKSP SDIEKISSNL EECAIDAEIP SNPLVQTGQD
481     QETPKEENAN AKETEDEKVG EKKAAVEEMS GKAEGNAIGG ALSSSAASVL PSTAQTSPLS DGMELSADQV AARSDEHHCN
561     TTLLESNSEA GGVEQHKNTA NVSEKEGAKA TDNKQVPGSP EQSFASSCAE QDSAEVAIQR TDSSEHTAEP GEEHIATA
```

5.335 PREDICTED: similar to caspase recruitment domain protein 9 [Gallus gallus]

Protein Accession	gi|118099415
Mean Expression Ratio	1.03
Median Expression Ratio	1.03
Credible Interval	(0.792, 1.33)
Associated Peptides	1
Associated Spectra	1
Coverage	0.0196

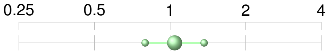

A	2.5	50	97.5	Sequence
**1**	**0.8**	**1.0**	**1.4**	**KAGVLLDILQR**

```
1       MHRKDEAAHC YFSLTTMLEE DNDETCWNSL ENFRVKLISV IDPSRITPYL RQCQVINHDD EEQVLNDPSL VMRKRKAGVL
81      LDILQRTGRK GFEAFMESLE LYYPQLYKKI TGKEPSRVFS LIIDTAGESG LSQLLMNEIT KLQRTVQEER QKAQELTVWL
161     HTKENMIREM WVRDSLLRKH QERVQKMREE RDSLSKELRK CKDENYNLAM SYARQSEEKS SALMKNRDLL LEIDSLKHSL
241     MKAEDDCKLE RKHSMKLKHA IEQRPSHEVM WEIQQEKELL LAKNQELENT LQVGGGDWNL ETSLSHETVQ NDCSQVLERQ
321     DLLNTLYHLR KELRQAEVLR DKYAEEKEIL ELQCTSLRKD SQMYKKRMEA VLEQMEEVAS ERDQALLTRE QFYTQYSKNL
401     VERDTYRKQI RELGERCDEL QLQLFQKEGQ LLATEAKLKR LQLELPALTS DLDDTSSRDS QDLTLHGHLD EDSHLTKKDC
481     CKGQTQQFSM QESNLTAESP TFEECSSAHE ELSEKERRRM KDCFERYRRK RALRRAPAGR RPEADWEPST GSDNTDTEGS
```

5.336 PREDICTED: similar to Pleckstrin homology domain containing, family C (with FERM domain) member 1 i

Protein Accession	gi|118092231
Mean Expression Ratio	1.03
Median Expression Ratio	1.03
Credible Interval	(0.796, 1.33)
Associated Peptides	1
Associated Spectra	1
Coverage	0.0218

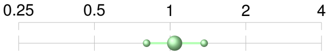

A	2.5	50	97.5	Sequence
**1**	**0.8**	**1.0**	**1.4**	**LLIPVAEGMNEIWLR**

```
1       MTLDGIRMPD GCYADGTWEL SVHVTDLGRD VSLRVTGEIH IGGVLLRLVE KLDVKKDWSD HALWWEKKKT WLLKTHWTLD
81      KYGIQADAKL QFTPQHKLLR LQLPNMKYVK VKVNFSDRVF KAVSDICKTF NIRHPEELSL LRKPRDPSKK KKKKLDDQCD
161     DEAFELEGPL ITPGSGNIYS SPGLYSKTMT PTYDAHDGSP LSPTSAWFGD SALSEGNPGI LAVSQPVTSP ESLAKMYKPQ
241     ALLDKAKINQ GWLDSSRSLM EQEVKENEAL LLRFKYYSFF DLNPKYDAIR INQLYEQSKW AILLEEIECT EEEMMMFAAL
321     QYHINKLSIM SSENHLNNSD KDVDEVDAAL SDLEITLEGG KTSTILGDIT SIPELADYIK VFKPKKLTLK GYKPYWCTFK
401     DTSISCYKSK EEANGTPAHQ MNLRGCEVTP DVNISGQKFN IKLLIPVAEG MNEIWLRCDN ERQYASWMAA CRLASKGKTM
481     ADSSYSMEVQ NILSFLKMQH LNPDPQLIPE QITTDINPEC LVSPRYLKKY KNKQPGYVRD LLTARILEAH QNVAQMSLIE
561     AKMRFIQAWQ SLPEFGITHF IARFQGGKKE ELIGIAYNRL IRMDASTGDA IKTWRFSNMK QWNVNWEIKM VTVEFADDVR
641     VSFICTEVDC KVVHEFIGGY IFLSTRAKDQ NESLDEEMFY KLTSGWV
```

5.337 signal recognition particle 72kDa [Gallus gallus]

Protein Accession	gi|57530084
Mean Expression Ratio	0.976
Median Expression Ratio	0.976
Credible Interval	(0.752, 1.26)
Associated Peptides	1
Associated Spectra	1
Coverage	0.0164

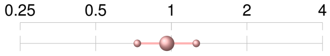

A	2.5	50	97.5	Sequence
1	0.73	0.96	1.3	HQKPAGAPATK

```
1       MAAATAGGPG AATALWSEVN RCGQNGDFAR ALKSVNKILQ INKDDVTALQ CKVVCLIQNG SFKEALSVIN THTKVLSSDV
81      IAFEKAYCEY RLNRIENALK TIQSASQQTD KLKELYGQVL YRLERYDDCL AAYRDLIRNS QDEYEEERKT NLSAVVAAQS
161     TWERVTPENL GLREATYELC YNNACALIGQ GKLNEAMKKL QKAEELCRQS LSEDSDVTEE DIEAELAIIH GQMAYIMQLQ
241     GRTEDALQLY NQIIKLKPTD VGLLAVIANN IITINKDQNV FDSKKKVKLT NAEGVEHKLS KKQLQAIEFN KALLAMYTNQ
321     AEQCRKLSAS LQSQSPEHLL PVLIQAAQLC REKQHTKAIG LLQDFAEQHP ANAAEIKLTM AQLKIAQGSV TKACMILRSI
401     EELQHKPGMV SALVTMYSHE EDIDSAIEVF TQAIQWYQQF QPESPVHLSL IREAANFKLK HGRKKEAISD LEELWKQNPK
481     DVHTLAQLIS AYSLVDPEKA KVLSKHLPSS DTMSLKVDVD ALENSHGATY VRKKAGKLTG DNQQKEQGQG EVKKKKKKKK
561     GKLPKNYDPK VTPDPERWLP MRERSYYRGR KKGKKKDQVG KGTQGSTTAG SSELDASRTA SSPPTSPRPG SAAAVSAASN
641     VIPPRHQKPA GAPATKKKQQ QKKKKGGKGG W
```

5.338 toll interacting protein [Gallus gallus]

Protein Accession	gi|57529999
Mean Expression Ratio	1.02
Median Expression Ratio	1.02
Credible Interval	(0.793, 1.32)
Associated Peptides	1
Associated Spectra	1
Coverage	0.0511

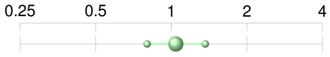

A	2.5	50	97.5	Sequence
1	0.8	1.0	1.4	GPVYVGELPQDFLR

```
1       MATTVSTQRG PVYVGELPQD FLRITPTQQQ QQIQLDAQAA QQLQYGGPMS TVGRLSITVV QAKLAKNYGM TRMDPYCRIR
81      LGYAVYETPT AHNGAKNPRW NKVIQCTVPP GVDSFYLEIF DERAFSMDDR IAWTHITIPE SLKQGNVEDE WYSLSGRQGD
161     DKEGMINLVM SYTSLPAAMM MQPQPVVLMP TVYQQGVGYV PIAGMPAVCN PDMVPVAIPP PAVNPQHLCN EEDLKSIQDM
241     FPNMDREVIR SVLEAQRGNK NAAINSLLQM TEES
```

5.339 high-mobility group box 1 [Gallus gallus]

Protein Accession	gi|45382473
Mean Expression Ratio	0.976
Median Expression Ratio	0.976
Credible Interval	(0.807, 1.18)
Associated Peptides	3
Associated Spectra	4
Coverage	0.126

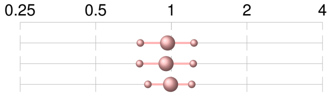

A	2.5	50	97.5	Sequence
1	0.75	0.97	1.2	NYVPPKGETK
1	0.75	0.95	1.2	GKVDAGKK
2	0.81	1	1.2	KFKDPNAPK

```
1       MGKGDPKKPR GKMSSYAFFV QTCREEHKKK HPDASVNFSE FSKKCSERWK TMSSKEKGKF EDMAKADKLR YEKEMKNYVP
81      PKGETKKKFK DPNAPKRPPS AFFLFCSEFR PKIKGEHPGL SIGDVAKKLG EMWNNTAADD KQPYEKKAAK LKEKYEKDIA
161     AYRAKGKVDA GKKVVAKAEK SKKKKEEEED EDEDEEDEED EEEEEEEEED DDDDE
```

5.340 cathepsin B [Gallus gallus]

Protein Accession	gi|46195455
Mean Expression Ratio	1.02
Median Expression Ratio	1.02
Credible Interval	(0.82, 1.28)
Associated Peptides	2
Associated Spectra	2
Coverage	0.0941

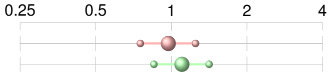

A	2.5	50	97.5	Sequence
1	0.75	0.97	1.2	SGVYQHVSGEQVGGHAIR
1	0.85	1.1	1.4	HCEPGYSPSYKEDK

```
1       MSWSRSILCL LGAFANARSI PYYPPLSSDL VNHINKLNTT GRAGHNFHNT DMSYVKKLCG TFLGGPKAPE RVDFAEDMDL
81      PDTFDTRKQW PNCPTISEIR DQGSCGSCWA FGAVEAISDR ICVHTNAKVS VEVSAEDLLS CCGFECGMGC NGGYPSGAWR
161     YWTERGLVSG GLYDSHVGCR AYTIPPCEHH VNGSRPPCTG EGGETPRCSR HCEPGYSPSY KEDKHYGITS YGVPRSEKEI
241     MAEIYKNGPV EGAFIVYEDF LMYKSGVYQH VSGEQVGGHA IRILGWGVEN GTPYWLAANS WNTDWGITGF FKILRGEDHC
321     GIESEIVAGV PRMEQYWTRV
```

5.341 cell division cycle 37 protein [Gallus gallus]

Protein Accession	gi|45382699
Mean Expression Ratio	0.978
Median Expression Ratio	0.977
Credible Interval	(0.757, 1.26)
Associated Peptides	1
Associated Spectra	1
Coverage	0.0356

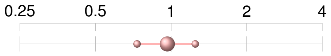

A	2.5	50	97.5	Sequence
1	0.73	0.96	1.3	AEEKEETEEQKEQK

```
1       MVDYSVWDHI EVSDDEDETH PNIDTASLFR WRHQARVERM EQFQKEKEEL DKGCRECKRK LAECQKKLKE LEVAEPGGGS
81      GGGRGERERL QAEAQQLRHE ERNWESKMEE LRKKEKNMPW NVHTLSKDGF SKSVFNVKAE EKEETEEQKE QKHKTFVERH
161     EKQIKHFGML RRWDDSQKYL SDNPHLVCEE TANYLVIWCI DLEVEEKQAL MEQVAHQTIV MQFILELAKS LKVDPRACFR
241     QFFTKIKTAD QQYMEGFNDE LEAFKERVRG RAKARIERAM REYEEEERQK RLGPGGLDPV DVYESLPPEL QKCFDAKDVQ
321     MLQDTISRMD PTEAKYHMQR CIDSGLWVPN AKAAAEGGGQ GGAHGQPGGA DSEALYEEIP KESGEEEGGE GKA
```

5.342 PREDICTED: similar to plasminogen [Gallus gallus]

Protein Accession	gi|118088308
Mean Expression Ratio	0.977
Median Expression Ratio	0.977
Credible Interval	(0.757, 1.26)
Associated Peptides	1
Associated Spectra	1
Coverage	0.0150

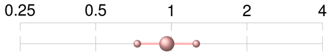

A	2.5	50	97.5	Sequence
1	0.73	0.96	1.3	CTTPPPVPAPGR

```
1       MGISKATLLL LFLLSSVQGS ILDNYVRTEG AWLLNSKKET YKTSSKEECA EQCETETKFP CRAFLFTSKD QQCITLAENT
81      KTAVLFRRTN AVLYEKRIYL LECKKGRGKD YRGTEAKTQK GVACQKWADT APHKPNYTPE KHPSAGLEEN YCRNPDNDEK
161     GPWCYTTDPN TRFDYCNIPE CEVECMHCSG ENYHGVVATT ASGLECQRWD SQLPHSHGYL PENFPEKDLK MNYCRNPDGE
241     PRPWCFTTNP NKRWEFCDIP RCTTPPPVPA PGRQCLSGRG EDYRGKISVT ESGNTCQHWS AQFPHKHART PENYPCKNLE
321     ENYCRNPDGE QMPWCYTTNA TARWEYCTIP SCDGKEPDTP AVDQPEQAQA TEECYQGNGV SYRGTASFTI TGKKCQAWNS
401     MSPHRHNKTS EHFPNADLRQ NYCRNPDADS RPWCYTTDPS VRWEYCNLKR CSDNIQTILP KPPQTTLEPN PDCIHSNGID
481     YRGTVARTAR GRICQEWSSQ TPHKHDYFTP RTHPKSGLEK NYCRNPDGDV NGPWCYTTDP RKAWEYCDIP KCSPAQYECG
561     KAKVRPKLCA QRIVAGCISH PHSWPWQISL RTSYGLHFCG GTLIDPKWVL TAAHCLEKSL RPSSYKVYLG LHEERALESS
641     VQKRDVEKLF KEPHGQDIAL LKLRSPAVIT DQVIPVCLPK ENAVLGGREE CYVTGWGDTK GTPGQGYLKE TGFPVIENKI
721     CNRPEFLNGR VKKHELCAGN IHGGTDTCQG DSGGPLVCLD QDKFVQHGVT SWGLGCALPM KPGVYVRVSA YIPWIKSIME
801     NN
```

5.343 PREDICTED: hypothetical protein [Gallus gallus]

Protein Accession	gi|50753314
Mean Expression Ratio	0.975
Median Expression Ratio	0.977
Credible Interval	(0.757, 1.26)
Associated Peptides	1
Associated Spectra	1
Coverage	0.068

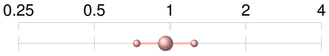

A	2.5	50	97.5	Sequence
1	0.73	0.96	1.3	QPPIQSTAGAVPVR

```
1       MSAPSALVKQ PPIQSTAGAV PVRNEKGELS MEKVKVKRYV SGKRPDYAPM ESSEEEDEEF QFIKKAKEQE VEPEEQEEEV
81      ANDPRLRRLQ NRITEDVEER LARHRKIVEP EVVGESDSEV EGEAWRVERE DTSEEEEEEI DDEEIERRRG MMRQRAQERK
161     TEELEVMELE DEGRSGEESE SESEYEEYTD SEDEMEPRLK PVFIRK
```

5.344 PREDICTED: similar to mKIAA0681 protein [Gallus gallus]

Protein Accession	gi|118100456
Mean Expression Ratio	0.977
Median Expression Ratio	0.977
Credible Interval	(0.757, 1.26)
Associated Peptides	1
Associated Spectra	1
Coverage	0.00966

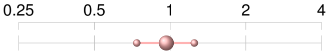

A	2.5	50	97.5	Sequence
1	0.74	0.96	1.3	MPPAEAVK

```
1       MASVNNGGSA DVVYLDLCKA FDMVVHHVLI SSDIPTRPTN ATTINLPTST LEIQRFPREP PRNTGAERPE KGVGSEPITA
81      TVIPQISGVQ TCNTVRVLEW KDGVATLPGS NLRFRINEYG TLKVVSADKM PPAEAVKEGH AKKDGDSDVA PTSRDNTIVA
161     QDVPEQSKLP TADSICHCDT CGRRHVSDGA REGRGFCSEH CHQQFKERSV IVENSASSTS ATEILKPVKK RKRKDYQSPS
241     EEDYESEQME KQEEMKNSVG DSAISNPEAH AWSQHSASEE KKEGWSWASY LEEQKAVAAP LDLFQDYQVA SQHKNGFKVG
321     MKLEGIDPQH PSMYFILTVA EVCGYRMRLH FDGYSECHDF WLNADSPDIH PAGWFEETGH KLQPPKGYKE EEFSWTNYLK
401     ITKAQAAPKH LFVIRNTAPP GFEVGMKLEA VDRMNPSLIC VATVTDVVDD RFLVHFDNWD DTYDYWCDPS SPYIHPVGWC
481     QEHGKPLTPP QDYPDPDNFI WEKYLKETGA SAVPAWAFKV RPPHGFLVNM KLEAVDRRTP SFIRVASVED VEDHRIKIHF
561     DGWSHVYDFW IDADHPDIHP IGWCSKTGHP LQPPLRPKEP ASSAHSGCPT LGCKNIPHTK SSKYSFHHRK CPTPGCDGSG
641     HVTGRFTAHY CLSGCPLAEK NQGKLKADLS DTEASTRKRN LIGFPQRKKS RHHGRGRPPK YRKIQQEDFQ TISSDNMHQS
721     LFMSALSAHP DRSLSLCWEQ HCKLLPGVAG ITATTVAKWT IDEVFSFVQT LTGCEDQAKL FKDEMIDGEA FLLLTQADIV
801     KIMSVKLGPA LKIYNAILMF KNADDTLK
```

5.345 3-oxoacid CoA transferase 1 [Gallus gallus]

Protein Accession	gi|60592998
Mean Expression Ratio	1.02
Median Expression Ratio	1.02
Credible Interval	(0.79, 1.32)
Associated Peptides	1
Associated Spectra	1
Coverage	0.0348

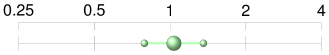

A	2.5	50	97.5	Sequence
1	0.8	1.0	1.4	GLTLVEIWEGLSVDDIKK

```
1       MAAYKFLLPP LGRRILLPAA RAWGAEASGC YFSTSCHRNT KFYTDPVEAV KDIPNGATIL VGGFGLCGIP ENLIGGLLKT
81      GVKGITAVSN NAGVDNFGLG LLLQTKQIKR MVSSYVGENA EFERQYLSGE LEVELTPQGT LAERIRAGGA GIPAFYTSTG
161     YGTLVQEGGA PIKYNSDGTI AIASQPREVR EFDGRHFILE KSITGDFALV KAWKADRAGN IIFRKTARNF NQPMCKAAKT
241     TVVEVEEIVD IGAFAPEDIH VPKIYVDRLI QGEKFEKRIE RLSIRKPEDS KAKQKPGDNV RERIIRRAAL EFEDGMYANL
321     GIGIPLLASN FISPDITVHL QSENGVLGLG PYPLESEVDP DLINAGKETV TVLPGSSYFS SDESFAMIRG GHVDLTMLGA
401     MQVSKYGDLA NWMIPGKMVK GMGGAMDLVS SAQTKVVVTM EHSAKGNVHK ILEKCNLPLT GKQCVNRIIT EKAVFDVDKK
481     KGLTLVEIWE GLSVDDIKKS TGCDFAVSPK LIPMRQI
```

5.346 PREDICTED: similar to axonal transport of synaptic vesicles [Gallus gallus]

Protein Accession	gi|118094941
Mean Expression Ratio	1.02
Median Expression Ratio	1.02
Credible Interval	(0.793, 1.32)
Associated Peptides	1
Associated Spectra	1
Coverage	0.00564

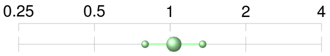

A	2.5	50	97.5	Sequence
**1**	**0.8**	**1.0**	**1.3**	**RPEDEKEPQR**

1 MEQIILSEIP RHVQDNRGIR PSQHGFMTGG SCPTNPIFYD RLTQLVHDGK AVDLIYVDFS KAFGSLPQHS RGEAAAVAGQ
81 VHMLLGNEPE SRAKRVVVDG LRSKWQPVVR WCSPGVAILN PKQPKETPKS FSFDYSYWSH TTPADINYAS QKQVYRDIGE
161 EMLQHAFEGY NVCIFAYGQT GAGKSYTMMG KQEKDQQGII PQLCEDLFSR INDTTNDNMS YSVEVSYMEI YCERVRDLLN
241 PKNKGNLRVR EHPLMGPYVE DLSKLAVTSY NDIQDLMDSG NKARTVAATN MNETSSRSHA VFNIIFTQKR HDAETDITTE
321 KVSKISLVDL AGSERADSTG AKGTRLKEGA NINKSLTTLG KVISALAEMD SGPNKNKKKK KTDFIPYRDS VLTWLLRENL
401 GGNSRTAMVA ALSPADINYD ETLSTLRYAD RAKQIRCNAV INEDPNNKLI RELKDEVARL RDLLYAQGLG DIIDMTNAIA
481 GISPSSSLSA LSSRAASVAS LHERIMFAPG SEEAIERLKE TEKIIAELNE TWEEKLRRTE AIRMEREALL AEMGVAMRED
561 GGTLGVFSPK KTPHLVNLNE DPLMSECLLY YIKDGITRVG REDAEKRQDI VLSGHFIKEE HCLFRSDTRT GGEVIVTLEP
641 CEGADTYVNG KKVTEPSVLR SGNRIIMGKS HVFRFNHPEQ ARQERERTPC AETPAEPVDW AFAQRELLEK QGIDMKQEME
721 QRLQELEDQY RREREEANYL LEQQRLDYES KLEALQKQMD SRYYPEANEE EEEPEDEVQW TEREFELALW AFRKWKWYQF
801 TSLRDLLWGN AIFLKEANAI SVELKKKVQF QFVLLTDTLY SPLPPDLLPP DAAKDREKRP FPRTIVAVEV QDQKNGATHY
881 WTLEKLRQRL DLMREMYDRA AEVPSSVIED CDNVVTGGDP FYDRFPWFRL VGRAFVYLSN LLYPVPLVHR VAIVSEKGEV
961 KGFLRVAVQA ISADEEAPDY GSGVRQSGTA KISFDDQHFE KFQSESCPAV GMSRSGTSQE ELRIVEGQGQ ISDLGPSADE
1041 VNNNTCAVTP EDLLLDSPEK STMDGPLEAA LDHLKLGSIF TFRVTVLQAS SISAEYADIF CQFNFIHRHD EAFSTEPLKN
1121 TGRGPPLGFY HVQNIAVEVT KSFIEYIKSQ PIVFEVFGHY QQHPFPPLCK DVLSPLRPSR RHFPRVMPLS KPVPATKLST
1201 MTRPSAGPCQ CKYDLMVFFE ICELEANGDY IPAVVDHRGG MPCHGTFLLH QGIQRRITVT LVHETGSLIR WKEVRELVVG
1281 RIRNTPEADE SLIDPNILSL NILSSGYIHP SQDDRTFYQF ETAWDSSMHN SLLLNRVTPY REKIYITLSA YIEMENCTQP
1361 AVITKDFCMV FYSRDAKLPA SRSIRNLFGS GSLRASESNR VTGVYELSLC RVADAGSPGM QRRRRRVLDT SVAYVRGEEN
1441 LAGWRPRSDS LILDHQWELE KLSLLQEVEK TRHYLLLREK LETTQRLGLE SLSPCSSEDS ESRSTSCVSS PLSADGAPEG
1521 RTSPPETPSE RQKELAVKCL RLLTHTFNRE YSHSHVCISA SESKLSEMSV TLMRDPSMSA LGVTTLTPSS TCPSLVEGRY
1601 NTMEVRTPQV SSRVESPDLE PVVEGEQKKS PARRPEDEKE PQRQLVPDIQ EIRVSPIVSK KGYLHFLEPH TNGWVKRFVV
1681 VRRPYVVYIYN SDKDSVERAI LNLSKAQVEY SEDQQAMLKT PNTFAVCTEH RGILLQASSD KDMHDWLYAF NPLLAGSIRS
1761 KLSRRRTAQM RI

5.347 hypothetical protein [Gallus gallus]

Protein Accession	gi|53127728
Mean Expression Ratio	0.98
Median Expression Ratio	0.978
Credible Interval	(0.759, 1.26)
Associated Peptides	1
Associated Spectra	1
Coverage	0.184

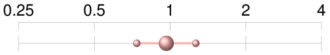

A	2.5	50	97.5	Sequence
**1**	**0.74**	**0.97**	**1.3**	**AQQEDALAQQAFEEAR**

1 MILNGVCVIW KGWIDLQRLD GMGCLEFDEE RAQQEDALAQ QAFEEARRRT REFEDRDRSH REEMEARRQQ DPSPGSNLGS
81 GDDLKLR

5.348 COP9 constitutive photomorphogenic homolog subunit 8 [Gallus gallus]

Protein Accession	gi|114158697
Mean Expression Ratio	1.02
Median Expression Ratio	1.02
Credible Interval	(0.79, 1.32)
Associated Peptides	1
Associated Spectra	1
Coverage	0.101

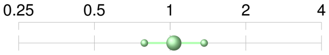

A	2.5	50	97.5	Sequence
1	0.79	1.0	1.4	FIPSSEPAPVPPIPNEQQLAR

```
1       MPVAVMAEGG FGFRKLLEQC ETQELEAPGG IATPLVYGQL LALYLLHNDM NNARYLWKRI PPAIKSANAE LGAVWSVGQR
81      IWQRDFPGIY TTISAHQWSE TVQPIMEAIR DATRRRAFGL VSQAYTSIVA DDFAAFVGLP VEEAVKGVLE QGWQADFSTR
161     MVMPKKPGVL DASFNRFIPS SEPAPVPPIP NEQQLARLTD YVAFLEN
```

5.349 hypothetical protein [Gallus gallus]

Protein Accession	gi|53135040
Mean Expression Ratio	0.978
Median Expression Ratio	0.979
Credible Interval	(0.757, 1.26)
Associated Peptides	1
Associated Spectra	1
Coverage	0.0769

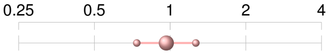

A	2.5	50	97.5	Sequence
1	0.74	0.96	1.3	LAMQEFMILPVGADNFK

```
1       MSILKIHARE IFDSRGNPTV EVDLYTNKGL FRAAVPSGAS TGIYEALELR DNDKTRYLGK GVSKAVEHVN KTIAPALISK
81      NVNVVEQEKI DKLMLEMDGT ENKSKFGANA ILGVSLAVCK AGAAEKGVPL YRHIADLAGN PEVILPVPAF NVINGGSHAG
161     NKLAMQEFMI LPVGADNFKE AMRIGAEVYH NLKNVTKEKY GKDATNVGDE GGFAPNILEN K
```

5.350 PREDICTED: hypothetical protein [Gallus gallus]

Protein Accession	gi|118101152
Mean Expression Ratio	1.02
Median Expression Ratio	1.02
Credible Interval	(0.792, 1.32)
Associated Peptides	1
Associated Spectra	1
Coverage	0.0259

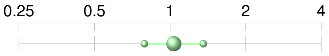

A	2.5	50	97.5	Sequence
1	0.79	1.0	1.4	MEEAQRSALRR

```
1       MEEAQRSALR RGRVRLVEGL RWAAGRGRGS RILSARRXGV VPPQLHDRPX APAPEGSKHD SWLLTWRPVG SRPSQYSSPS
81      XGTPGTVTWL TCWMRAAEAR CPHLWTXGLC SWSCLEKGVT KVXALRNACP SQSNLRVSDF GCLRHQPRVQ LLTRATTDWC
161     TSCEQTLVGT AXSSTMSASA ETLICRLELA LTXTVRSWRS VSGPCASTSG PCGTSKLRKL MWSCGSWRGS TTVPWTAASW
241     SSSPMVARQA IFSFPEGFME QMAKSFQSKG LXTISMGPSA RVXEENPNSS SSRPVEENKR TKDLRWIVNH PKMKLADVPX
321     SRMRFLSRLH QGMRTSQTPS PVCPLLVTSW CPIQLFQVLC PGGTRXVARG TWKPWTAYWN ITPVLKTCXP CYFGCQTSYP
401     PRGGTSRSRA VSTSYVKNSS SCAS
```

5.351 PREDICTED: similar to MGC82793 protein isoform 2 [Gallus gallus]

Protein Accession	gi|118084001
Mean Expression Ratio	1.02
Median Expression Ratio	1.02
Credible Interval	(0.791, 1.32)
Associated Peptides	1
Associated Spectra	1
Coverage	0.0755

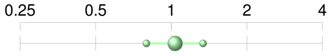

A	2.5	50	97.5	Sequence
**1**	**0.8**	**1.0**	**1.3**	**GDEQCHYYAGGQVYPGEAAR**

```
1       MEAARRGPRA GVGSRALLLA LLLHGAMGAE AEEPRPPRQR GDEQCHYYAG GQVYPGEAAR VPVTDHSLHL SQAKISKPAP
81      YWEGTAVING EFKELKLTDY EGKYLVFFFY PLDFTFVCPT EIIAFSDRIE EFRAINTEVV ACSVDSKFTH LAWINTPRKQ
161     GGLGPMKIPL LSDLTHQISK DYGVYLEDQG HALRGLFIID DKRILRQITM NDLPVGRSVD ETLRLVQAFQ YTDKHGEVCP
241     AGWKPGSETI IPDPAGKLKY FDKLN
```

5.352 PREDICTED: similar to multiple ankyrin repeats single KH domain protein [Gallus gallus]

Protein Accession	gi|118097192
Mean Expression Ratio	1.02
Median Expression Ratio	1.02
Credible Interval	(0.808, 1.29)
Associated Peptides	1
Associated Spectra	2
Coverage	0.00435

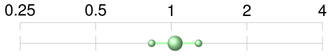

A	2.5	50	97.5	Sequence
**2**	**0.83**	**1.0**	**1.3**	**KQEEDEENKPK**

```
1       MLTDGTAAPL GDEEIDSVAP RAPPAAEPPA AAGPSPLGLR AVRLFGEAGP GGPGGPGAPA AGEAALDFKL AAAVLRSGGG
81      GGGSGSDEDE VSEVESFILD QEDLDNPVLK TTSELFLSGA AEGADLRAVD PETQARLEAL LEAAGIGKLS TADGKAFADP
161     EVLRRLTSSV SCALDEAAAA LTRMRAENNH NAGQVDNRSL AEACSDGDVN AVRKLLDEGR SVNEHTEEGE SLLCLACSAG
241     YYELAQVLLA MHANVEDRGN KGDITPLMAA ASGGYVDIVK LLLVHCADVN AQSSTGNTAL TYACAGGFVD IVKVLLKAGA
321     NIEDHNENGH TPLMEAASAG HVEVARVLLE YGAGINTHSN EFKESALTLA CYKGHLDMVR FLLEAGADQE HKTDEMHTAL
401     MEACMDGHVE VARLLLDSGA QVNMPADSFE SPLTLAACGG HVELAALLIE RGANLEEVND EGYTPLMEAA REGHEEMVAL
481     LLAQGANINA QTEETQETAL TLACCGGFSE VADFLIKAGA DIELGCSTPL MEAAQEGHLE LVKYLLAAGA NVHATTATGD
561     TALTYACENG HTDVADVLLQ AGADLEHESE GGRTPLMKAA RAGHLCTVQF LISKGANVNR ATANNDHTVV SLACAGGHLA
641     VVELLLAHGA DPTHRLKDGS TMLIEAAKGG HTNVVSYLLD YPNNVLPVPT ADLSQLTSPS QDQSQVPRVP VHTLAMVVPP
721     QEPDRTPQEN SPPLVGVVKG ASKQKSSSLQ VADKDLLPPF HPYQPLECIV EETEGKLNEL GQRISAIEKA QLKSLELIQG
801     EPLNKDKIEE LKKNREEQVQ KKKKILKELQ KVERQLQMKT QQQFTKEYLE TKGQTDTPLP LQQQCPLTGV FPEVEADKGL
881     PEDNFSELPQ VDTILSKDDE QQQSPPPAEQ IEFVPIQPLP APQCNFSGNL GYNGTESLEL QKAIGNQQNV GQQQQIAGQG
961     LLVQEPDGLM VATPAQTLTD TLDDLIAAVN SRVPSGSTGS LHTTESPTPE PCSQASSNVA SQSVLPMYPS VDIDAHTESN
1041    HDTALTLACA GGHEELVSVL IARGANIEHR DKKGFTPLIL AATAGHVGVV EILLDKGGDI EAQSERTKDT PLSLACSGGR
1121    QEVVDLLLAR GANKEHRNVS DYTPLSLAAS GGYVNIIKIL LNAGAEINSR TGSKLGISPL MLAAMNGHVP AVKLLLDMGS
1201    DINAQIETNR NTALTLACFQ GRAEVVSLLL DRKANVEHRA KTGLTPLMEA ASGGYAEVGR VLLDKGADVN APPVPSSRDT
1281    ALTIAADKGH YKFCELLINR GAHIDVRNKK GNTPLWLAAN GGHYDVVQLL VQAGADVDAA DNRKITPLMS AFRKGHVKVV
1361    QFLVKEVNQF PSDIECMRYI ATITDKDLLK KCHQCVETIV KAKDQQAAEA NKNATILLKE LDLEKSREES RKQALAAKRE
1441    KRKEKRKKKK EEQKRKQEED EENKPKETLE LHEDDDEEEN DEEVEQEVPI EPPSATTTTT IGISATSATF TNAFGKKRAN
1521    VVTTPSTNRK NKKNKTKETP QNMQIILPDQ HISLAQQKAD KNKINGEPRG GGASGNSDSD NLDSTDCNSE SSSGGKSQEL
1601    NFTMDTNSSE RRYASLLIPS QEEKTSTSAS KAPTRLDGEG NSNSLSTTYK PVSLPLTSPN VKLNLTSPKR GQKREEGWKE
1681    VVRRSKKLSV PASVVSRIMG RGGCNITAIQ DVTGAHIDVD KQKDKNGERM ITIRGGMEST RYAVQLINAL IQDPAKELED
1761    LIPKTHIRTP ASSNKSIHAN FSSGVSTASA SNKNSFPLGA PPLVTSQSST LSTFQPTNKL NKNVPANVRS SFPVSLPLAY
1841    SHPHFALLAA QTMQQIRHPR LPMAQFGGTF SPSPNTWGPF PVRPVNPGST NSSPKHNSSS RVGSQNGNIL QTESPGLATS
1921    SPITVSSVVA STQPLCATSN RTPSSVRKQL FACVPKTSAA ATAISTVTNT CSTLPSASSA PPNNGQVPAA FLPSTAPQTQ
2001    HSALKADSFS AVSAPKEKVS TPDQPVGNTC TPSSIAGSSS MSASSNSGVT EAHPSSSPAP LMNTQDEILP ASMSEMSPSM
2081    SASISSSSEP APLSLASPRS VVADNQDNSN LPQVAVPAPR VTHRMQSRGS FYSVVPNANL HQDPQSIFVT NQVPLTPSQG
2161    PPAAVQLSSA MNVMNGSQMH INPANKSLPP TFGPATLFNH FSSLFDSNQV PSNQGWGDCP LSTRAAADPS YTVQSTFLNN
2241    SMLGHVENVH PDNSKAPGFR PPSQRVSTSP VGLPSIDPSN SSTTSSSGPL TGFSANMQGA RVYLQGPAPV GTPSFNRQHF
2321    SPHPWTSATN SSASAPSNLS QPKTGNTNQD RKVPPPIGTE RLARIRQGGS VTPTPLGTNF TAPVGHSGIW SFGVNSVSEG
2401    LSGWSQPVMG NHPMHQQLSD PGTFSQHQPM ERDDSGIVAP SNIFHQPMPN SFVDFSKGLP ISMYGGTLIP SHPQLADGPG
2481    GPLFNGLHTP DPAWNPMIKV VQNSTECTDA QQIWPGTWAP HIGNMHLKYV N
```

5.353 PREDICTED: hypothetical protein [Gallus gallus]

Protein Accession	gi|118095169
Mean Expression Ratio	1.02
Median Expression Ratio	1.02
Credible Interval	(0.848, 1.23)
Associated Peptides	3
Associated Spectra	5
Coverage	0.146

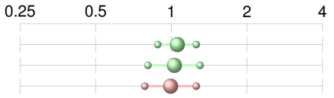

A	2.5	50	97.5	Sequence
3	0.89	1.1	1.3	AAEDDEDDDVDPK
1	0.81	1.0	1.3	AAEDDEDDDVDPKK
1	0.78	1	1.3	RAAEDDEDDDVDPKK

```
1       MKSQYPYDLK EKKEVVEETE NGRDAPANGN AENEENGEQE ADNEVDEEEE EGGEEEDEEE EGDGEEEDGD EDDEAEGATG
81      KRAAEDDEDD DVDPKKQKTD EDD
```

5.354 PREDICTED: similar to KDEL (Lys-Asp-Glu-Leu) containing 1 [Gallus gallus]

Protein Accession	gi|118084661
Mean Expression Ratio	1.02
Median Expression Ratio	1.02
Credible Interval	(0.793, 1.32)
Associated Peptides	1
Associated Spectra	1
Coverage	0.026

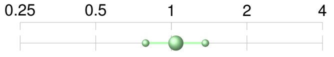

A	2.5	50	97.5	Sequence
1	0.79	1.0	1.4	YFYLQAVDTEGQR

```
1       MCSIWRWCVA LGVLALGVAA GGGGRLSAER SAVWGPGLRA EAVLPARYFY LQAVDTEGQR FTSSPGENAF QVKITAPDEQ
81      FTRVGVQVLD RKDGSFIVRY RMYASYKSLK IEVKTKDKHV AKSPYILKGP VYHENCDCPQ EESSAWLEEM NCPQVFPQIQ
161     RDLANFPVVD PDKIAVEIPQ RFGQRQSLCH YTIKDNEVYI KTYGEHVGFR IFMDAILLSL TRKVKMPDVE FFVNLGDWPL
241     EKKKPPQKLH PIFSWCGSSE SKDIVMPTYD LTDSVLETMG RVSLDMMSVQ ANTGPSWEDK NTTAFWRGRD SRKERLELVK
321     LSRKYPELID AAFTNFFFFK HDENLYGPIV KHISFFDFFK YKYQINIDGT VAAYRLPYLL AGNSVVLKQD SIYYEHFYNE
401     LQPWKHYIPF KSDLSDLLEK LQWAKEHDEE AKKIAKSGQE FARNNLMGDH IFCYYFKLFQ EYSSLQVSEP KIRDGMEKVQ
481     QPDDDLFPCT CHRKKTKDEL
```

5.355 dynamitin [Gallus gallus]

Protein Accession	gi|45382201
Mean Expression Ratio	1.02
Median Expression Ratio	1.02
Credible Interval	(0.792, 1.32)
Associated Peptides	1
Associated Spectra	1
Coverage	0.0274

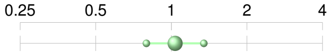

A	2.5	50	97.5	Sequence
1	0.8	1.0	1.3	LGTDGVDFSDR

```
1       MADPKYADLP GIARNEPDVY ETSDLPEDDQ AEFEAELEEL TSTSVEHLII NPNAAFEKFK DKRLGTDGVD FSDRISKSRT
81      TGYESGEYEI LGEGLGAKET PQQRYQRLQH EVQELIRDVE QIQSAVKESA AEEELTPMAL ARQLEGLKQQ LVSCHLQKLL
161     GPTAAIDFAD PEGALAKRLQ QQLEVPSVKK AAPAKSPPKA PGPTTDALTF ELFWRRPEQD QFSQTAKIAE LEKRLAQLEA
241     MVRCEPDSQN PLLVGAEGTS LVETVQILQA KVNILDAAVL DQVEARLQRR PGSKVNEIAK HKAIVQDADT QSKIHQVVYE
321     MMQRWDHMAS SLPDVVQRLL TLRDLHEQAS RFVQVLVHLD TTQQEVDVVQ RLLAEVQKTM KENLAVVEDN FAEVEARIKR
401     LQ
```

5.356 PREDICTED: hypothetical protein, partial [Gallus gallus]

Protein Accession	gi|118105147
Mean Expression Ratio	0.98
Median Expression Ratio	0.98
Credible Interval	(0.76, 1.27)
Associated Peptides	1
Associated Spectra	1
Coverage	0.293

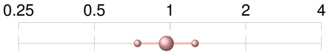

A	2.5	50	97.5	Sequence
**1**	**0.74**	**0.97**	**1.3**	**LNIISNLDCVNEVIGIR**

```
1       CPEVRLNIIS NLDCVNEVIG IRQLSQSLLP AIVELAEDAK WRVRLAIIEY MPLLAGQL
```

5.357 PREDICTED: similar to cyclophilin [Gallus gallus]

Protein Accession	gi|118089782
Mean Expression Ratio	0.98
Median Expression Ratio	0.98
Credible Interval	(0.757, 1.27)
Associated Peptides	1
Associated Spectra	1
Coverage	0.0306

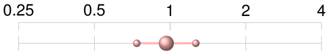

A	2.5	50	97.5	Sequence
**1**	**0.74**	**0.97**	**1.3**	**AFFDVDIGGER**

```
1       MSHPSPVERP GNPRNPRAFF DVDIGGERVG RIVFELFADI VPKTAENFRA LCTGEKGTGA TTGKPLHYKG CPFHRIIKQF
81      MVQGGDFSNQ NGTGGESIYG EKFEDENFHY KHDKPGLLSM ANAGPGTNGS QFFITTVPTS HLDGKHVVFG QVIKGMGVVK
161     ILENVEVNGE NPAKLCVIAE CGELKEGDDW GIVPQDGSGD TYPDFPEDSD IDLKDVLKGV AIAEDIKNIG NMFFVNLFFF
241     RYVEASETVA EEGDKPKLKT VGLSCVLNIG ACKLKLSDWQ GAIESCSEAL QIDPANTKAL YRRAQGWQGI KDLDQALADL
321     KKAHEIAPED KAIQTETLKI KQKIKAQKEK EKAAYAKMFA
```

5.358 hypothetical protein [Gallus gallus]

Protein Accession	gi|53130686
Mean Expression Ratio	1.02
Median Expression Ratio	1.02
Credible Interval	(0.79, 1.32)
Associated Peptides	1
Associated Spectra	1
Coverage	0.109

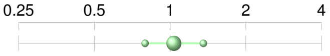

A	2.5	50	97.5	Sequence
**1**	**0.8**	**1.0**	**1.4**	**RSPNKPLPELNDQYK**

```
1       MAENGDGENM SILESKICQQ IEYYFGNHNL PRDKFLKEQI KLDDGWVPLE VMIKFNRLSR LSKDFGVIVE ALRKSKTGLM
81      EINEDKTKIR RSPNKPLPEL NDQYKAAIKN RSVYVKGFPL DATLDDIKEW LEDKGPV
```

5.359 PREDICTED: similar to N-acetylglucosamine-phosphate mutase [Gallus gallus]

Protein Accession	gi|118088855
Mean Expression Ratio	0.979
Median Expression Ratio	0.98
Credible Interval	(0.758, 1.26)
Associated Peptides	1
Associated Spectra	1
Coverage	0.0129

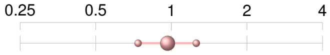

A	2.5	50	97.5	Sequence
1	0.74	0.97	1.3	VHQKPPK

```
1       MDFEGLKRYS ALHPKPAGLT LQYGTAGFRS KAQQLEHVMF RMGLLAALRS RAMGATIGVM VTASHNPEED NGVKLIDPLG
81      EMLHPSWEEY ATQLANAEDQ ELQEIVTEIC QKAAVNQRKD ASVFVGRDTR PSSKELSQAV IDGISVLGGQ YHDYGLVTTP
161     QLHYMVCCQN TQGQYGKPTL EGYYQKLSKA FTELIKKSLS SGEAQRQLKI DCANGIGALK LAEMETYFPK EVQVQVYNDG
241     TKEKLNYLCG ADFVKVHQKP PKGLDMKPNE SCCSFDGDAD RIVYYYKDTA GHFHLIDGDK IATLISIFLK ELLAKMGQTL
321     KMAVVQTAYA NGSSTRYLEE TVKVPVHCVK TGVKHLHHKA QEFDVGIYFE ANGHGTVLFS KAAENTIRQL AKEEKDVAKR
401     EAAKVLENMI DLINQTVGDA ISDMLVIEAI LALKGLTLQQ WDALYTDFPS RLLKVQVADR QVIDTTDAER RAVTPPGLQE
481     KIDALVKKYK LSRAFVRPSG TEDIVRVYAE ADTQENADAL AHEVSLAVYH LAGGKGAPPQ PI
```

5.360 hypothetical protein [Gallus gallus]

Protein Accession	gi|53132882
Mean Expression Ratio	0.98
Median Expression Ratio	0.98
Credible Interval	(0.756, 1.27)
Associated Peptides	1
Associated Spectra	1
Coverage	0.0208

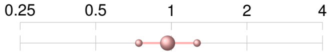

A	2.5	50	97.5	Sequence
1	0.74	0.97	1.3	HPKPDPSEDNSR

```
1       MAAAGPGAAG TARVSSGREL GCVPEVAAAL GAVARQGFDF LCAPLFHPRH RREFRLLPAK ERPGPQTRSD LLLAGRDWNT
81      LIVGKVSPWI RPDSPLEAVR RNSEAALQQE LDFAAYLGVP AVLLSLRGPH CPNLTLCDYN KRVGLALEVG PDLPSAEAID
161     RWLGEPLRAA ILPTSIFLTN KKGFPVLSRP HQRLLGRLLK LEVQVVLWGA PHHEPKPLSA YLQYLEHVGQ NRPPPSAYEL
241     FARGYEDYLQ CPLQPLMDNL ESQTYEVFEK DPIKYSQYQQ AIYKCLLDRV PEEEKETNVQ VVLVLGAGRG PLVNAALRAA
321     RMAQRRIRVY AVEKNPNAVV TLQSWQYEEW GSQVSVVPRD MREWRPPEAA DLLVSELLGS FGDNELAPEC LDGAQPCLKE
401     GGVSIPCSYT SFLAPLSSSK LYNEVRGCRE RDRHPEAQFE TPYVVRLHNF HQLAAPQPCF TFRHPKPDPS EDNSRYRVLS
481     FPVGVTTALH GFAGYFETTL YADVTLSIRP GTHSPGMFSW FPIFFPLKQP MAVQAGQRVV LSFWRRAAPQ KVWYEWAVTE
561     PRCSALHNPA GRSYTIGL
```

5.361 hypothetical protein LOC424451 [Gallus gallus]

Protein Accession	gi|60302834
Mean Expression Ratio	0.98
Median Expression Ratio	0.98
Credible Interval	(0.76, 1.27)
Associated Peptides	1
Associated Spectra	1
Coverage	0.0113

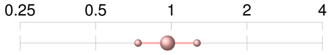

A	2.5	50	97.5	Sequence
1	0.74	0.96	1.3	VHEGSVEEK

```
1       MGGSASGPLD DSKCAYIRGK TEAIIKNFSP HYKRQYAVAF CKHVQDELEQ HRNSQSQFLK TRPKSEAGTV LYETELLHFA
81      EDVKKWKDRY VVIKNDYTVD CFETKEAYQK GLSPKHHTVP AGGKVLTLEE DYNLLSDKHF PDPVGLSEKE TAQAFVQLPR
161     EFPVYLWQPF ARHSYYCFQE PEAQRQFSAV LSDCVRHSNY DFLKQTTYEV EAFLEAIQFF RQEKGHYGTW EMITGSEKEI
241     LSNLVMEELL PNLQTMILPK MKGKRNDRKR AWFGFVEEAY GLVQQQVAEG LSTLKEECRD FAKTLEGTIR SDMDQILNSK
321     NFLAGKIKAT VSEPAQKCCA ENIQPFLTSI LEELMGPVSS GFTEVRSLFD KEVNEIIQNF QKTNDITKLK EDVDQLMSLP
401     FNSVKMEPSY LKVNLLQELL HDLKSRFKVY HIDFVVQKTQ NFMQELMENA VYTFEQLFSP SCQADPVKVT TTLEKVKQRV
481     LKQYDYDSST VRKKIFQEAL VQITLPTMQK TLASTCKPEL QKYEQFIFAD YTSVIQVENV YEEILYQILL EETLKVIKEA
561     AVLKKHNLFE DNLNLPCESV SSLTDLKTPS GSAQTTPAKK PSTTRMEASD TETQSEETLI VTEKILWDKD HEDRKPSDKE
641     VVISVNMDGS QASKSEEVVI TDISEKWESL STTPTKQEFA EGNTPLENES MVKADSVGDT KKKLTTREEA VEEEVASGTE
721     NTLRDVSPEK ELKVHEGSVE EKVTSGKESK VQEDVEIELT SPPNKTAETE ILGSAEMKIE IEKGVTEELS QSEMK
```

5.362 PREDICTED: similar to MGC84609 protein [Gallus gallus]

Protein Accession	gi|118097664
Mean Expression Ratio	0.98
Median Expression Ratio	0.98
Credible Interval	(0.758, 1.27)
Associated Peptides	1
Associated Spectra	1
Coverage	0.0114

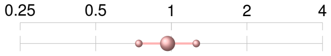

A	2.5	50	97.5	Sequence
**1**	**0.74**	**0.97**	**1.3**	**KVEEENEKK**

```
1       MDATLEKVVD PTLAEMGKNL NEAMKMLEDS QRKVEEENEK KYARKDIPGP LQGSGQDMVS ILQLVQNLMH GEEEEESSQA
81      YRLQNVGEQG HMALLGHSLA AYISVLDKER LRKLTTRILS DTTLWLCRLF RYENGSAYYH EDDREGLLKI CRLVIHTCYE
161     DYTVEGFNVL CTKQPVIYIS SAARTGLGQA LCNQLGLPLS CMCRVPCNTM FGSQHQMDVA LLDRLIKDDV ECGKLPLLLV
241     ANAGTPGAGH TDKLGRLKEL CEQYNMWLHV EGVNLATLAL GYVSSSVLAA TKCDSMTLTL GSWLGLPAVP AVTLYKHEDP
321     SLSLVAGLTS SQPVEKLRAL PLWLSLQYLG HDGIVERIKH ASQLSQRLLE NLKNLDFIKT SVEDELSSPV VVFKFFQKYL
401     NRDLTPHAAQ TSAIQHPVIS NEGHIYDTFN QWLGDQLAQM VPASGIDVVE LEDEGTCVRF SPLMTSAVLG TEIQDVDQLV
481     DCLKMKIPVL TSTLQLREEF EQEVRRTVGL LYIEDLSWPG LGVVRYSYHS DEKNNDNQEK ELEKINTELL KKLNELESDL
561     TFSLGPEFGG QKNCVYIGMV TEDLDVSELV ETIAATGREI EENSRLLENM TEVVRKGIQE AQLQLQKANE ERLLEEGLLR
641     QIPVVGSVLN WFSPFQASPK GRTFNLTAGS LESTESTYVS KAQGTGITPP PTPTSSLTKQ KHLGQKIFKR SLRNSDAFSE
721     TSSVSHCDDM EKMDQRSPTL SPGQEQRPLE PEKTELLPKV VPQATNKDTE DIQSSKSLND CTSVEEQGSQ R
```

5.363 PREDICTED: hypothetical protein [Gallus gallus]

Protein Accession	gi|118090972
Mean Expression Ratio	0.98
Median Expression Ratio	0.98
Credible Interval	(0.758, 1.26)
Associated Peptides	1
Associated Spectra	1
Coverage	0.0346

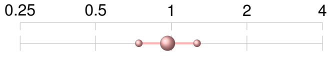

A	2.5	50	97.5	Sequence
**1**	**0.74**	**0.97**	**1.3**	**TMLDALCAAAQALR**

```
1       MCGWWVVGDD CAFASQKKAG RRGLCGTVLV HKVAGAMAEA GASLDEIVTR VSAVTKAMGT LGLSLSPCSV PGSKPTFQLA
81      SDEMELGLGI HGEAGVRRMK VMPADEAVET MLAHMTDPSN ASHLPLSPGA SVVLVVNNLG GLSCLELSIV AGVAVRSLER
161     RGVCIARALV GSFMTALEMA GISLTLLLVD EELLRLIGPG MERAQKVLAR VCSTLLGLQD KLNELDRAAG DGDCGHTHAR
241     AARAIQEWMR AQPLPPSPAH LFSALADLLL DKMGGSSGVL YGLFLTAAAH PLHNRSDLPA WADAVDAGIE AMQRYGGAAP
321     GDRTMLDALC AAAQALRALR SPGADLLTVL ASAVESAEAA AESTRHMEAG AGRASYISSA QLLQPDPGAV AAAAVLRAVL
401     EGLQG
```

5.364 PREDICTED: similar to glutathione synthetase [Gallus gallus]

Protein Accession	gi|118100530
Mean Expression Ratio	1.02
Median Expression Ratio	1.02
Credible Interval	(0.797, 1.33)
Associated Peptides	1
Associated Spectra	1
Coverage	0.0252

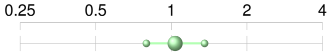

A	2.5	50	97.5	Sequence
1	0.8	1.0	1.4	DVYEQGSLDGSR

```
1      MPPLSGTKPV LLVGEAANRL AFRGGARLLS NRREEGGAAR ERGKQVVSYA PFTLLPSAVP RALFEQAYAV QRDFNLLVDA
81     ISRDKEFLER TLASTIKVDD FTARLFKIHQ QVLEEGLAQS VFLGINRSDY MFDCGAGSLP ALKQIEINTI AASFGGLASR
161    TAAVHGRVLR VLGKPEEAAR LLPNDPARGL AMGIAKAWEL YGSPSAVVMF LVKEAQRNIF DKRCVKVKTC HRNIRVIRRR
241    FRDVYEQGSL DGSRRLYVDG QEVAVVYYRE GYVPSNYNQQ NWEARLLLER SRAVKCPDIA TQLAGTKKVQ QELSCPGTLE
321    KLLPGHAEAV KRIRATFAGL YSLDVGEEGD QIAATAIASP ERFVLKPQRE GGGNNLYGEE LRQVLERIKD SPERTSYILM
401    DKIEPQPAVN YLLRARSPLK ASKCISELGI FGVYVRQGTE LVLNEAAGHL LRTKAVEHAD GGVAAGVAVL DTPYLV
```

5.365 PREDICTED: similar to ATM [Gallus gallus]

Protein Accession	gi|118085051
Mean Expression Ratio	0.98
Median Expression Ratio	0.98
Credible Interval	(0.76, 1.26)
Associated Peptides	1
Associated Spectra	1
Coverage	0.00459

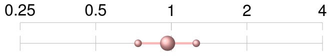

A	2.5	50	97.5	Sequence
1	0.74	0.97	1.3	LDPFPEYPAFEELR

```
1      MSLVLHDLLT CCRRLENERA TERRNEIENF KRLLRDPETV LQLDRNSDSR RGNQLNWDAV FSLLKKSFQK EMENLRLTKP
81     NASASTQTSK QKRMQEIGSL VKYFIRRANR RGPRLECQEL LNYVLHIIKD PASCAAYGSD CSSILLKDIL SVRKYWCEIS
161    QQQWPDLLIL YGKFYLTPSG KMNRVLVARI IHTLMRGYCF QTDQLRSDVF SFFSTAMQYV RQERNTAGLD HIIAAMNIFF
241    NTFAVNCRKR ICKIGEEILP TVLYIWTQYR PKDSLKESII ELFLLQVHIH HPKGATSQEK GAHNSAKWQS ILHNLFDLLV
321    NEINVIGSRG KYSSGSRSVA IKENLIELMA DICHQVFTED TKVLEITQSY TVTQSGGDTV PSKRRRIELG WEVIRDNLQR
401    SQKDFHVVPW LQITARLLSK YPMSFPNCEL PQLLNILHQL LPHQRRGERT PYVLKCLTEI ALCQNQKTDL KSTHKLELQR
481    TWSKIWSLTI RSISFQQIET EIFGLLGAMI QGNLVVTDKE LWKIFSGPAC KPSSSAVCCL ALAMPAYSVP ENLDVGMEQT
561    NRERNLDSVL KEAIMKWALS CNLEEEMEEC SELPPVLCSD FPHLTLQKVL VSLTMKNSRA TMTFFQNDAK CVQHLQGKEE
641    NSFLDVEELY LQTTFDEMDI FTNFAVNVVD KNVTGSRFVI NQSLRETLEC CLLAMSEKLL SSYAPKLVAA EHLVRCVSLL
721    IGVLGCYCYT GVFTEEDACK SELFQKAKSL IHYIGESISN SSKKLNEDAQ ISLLRTLIMQ CTKCLCNCTK NSANKMVSDL
801    FLRLLTSKFI NDLLDVCKNL MTFTGKPSEL GEVDLVGNDP EESIMEADNQ VSDDLFDDHS VTDISDTNES GEMQNVTGAV
881    SPLAEEQLTK QDLLHLEILR FLCICATTVQ IQTMSFRASD IRRKLLMLTD GSIFDSAKPL HLHVYLLLLK ELPVEENLLT
961    VDEVLKLLTP LSNVCSLYRR DQDVCKVILN NLLPIAVSLA QSGTRTEEKR NAQEQFLTVV GAFWTLAQGK RCTAPVRVAL
1041   LNCMKALLEA DPYSKWAILN VKNEDLPVSE VFPHFLADSH HQVSILAAKS ITSLFQDVKQ RDSSGLSTPL PLKLQQKAFE
1121   NVYLRVQEGM GNSVTRPNVL DKQCNRKAVL LMLITMVLCC SPVCEKQALF AICQSVKENG LEPHLVKKVL KKVSDIFGYK
1201   NIEDFMTSHL DYLVVEWLKI KDSGYSLSAF PYVLLNYTGL EEFYRSCYKV LVPHLVIRSQ FDDVKSLANK IGKDWRQVLA
1281   DCFPKILVNI LPHFASQSHG QREVAEQRET ASKVYDVLKD DNCLGKQQID NLSHNNLSEI VVELLMTLHE PPNVNAEKGP
1361   HLSKYIRELD PAPNPPHFPS YVIKATLDHI SNCHKSELKS LVAVLSKSPD SFQKILLALC KHASDMNNIY KKHRVLIIYH
1441   FFVSLLLKEI KDGLGGAWAF VLRDVIYTLI HHISSRPLIF RDVSMRSFLL CCDLLCCVCH TAVKYCSDAL ESHLHVIVGT
1521   LIPLAVDQPE IQPQVLRLLK YLVIDSKDNV HLYQAIKRLD PFPEYPAFEE LRTTQLKIKY SKGPYSLLEE INRFLSVSVC
1601   DSLPLTRLEG LYDLRKQLEQ YKDQMKDLLK TFQENPEDSV MVKLVVSLLQ LSKMAVNHAG EKAVLEAVGS CLGEIGPMDF
1681   STIALQHAEN ALDSKAADLL EDKKLQWVFI MLTQINTALT DNCIDVRAAA VSCLKNILAT NSGSEFWEVY KSQGDPMLIY
1761   LQPFRMPKKK VLAMPANDSE ASSETLDDTN LWIPLGESHE TWIKNLTRSI LDSGGVQNEV LKLMKPLCEV KTDLSQTLLP
1841   YLIHDILLHD SNESWRNILS VHVRKFFTAC CRFASSSRSA TPQNSDSEQE THVLRSVDKV SRRTMLAVVD YLRRQKRSVS
1921   GTVFDDSFWL DLNYLEVAVA AQSCAAHFTA LLYAEIYADK INMDKQQKSL TFEEESEKST IAILNEKSKE TGISLQDLLM
2001   DIYKSIGEPD SLYGCGGGRM LQPLARIRTY EHEAVWDKAL LTYDLEATLS PSTRQAGIIE ALQNFGLCHT LSMYLKGLEH
2081   ENTEWCAELQ EIRYQAAWRN MQWDNISSVK DETGGSGYHE SLYDALQSLR DKEFSTFYDT LKVARVNEVE ELCKGSLESV
2161   YTLYPSLCRL QLIGELENIG LLFSRPATTQ QLNDIYLKWQ RQSQLLEDSD FHFQEPIMAL RTVVLEILLE KENESAKREC
2241   IKDILTKHLV ELSKLARTAN NTQLPERAMF QIKQHNPTQY GVSEWQLEEA QVFWAKKEES LALNILKELI KKLDAIWFQN
2321   DPHLKLMYTE CLRLCGTWLA ETCLENPTVI MQKYLEKAVE IAASHHGDNS DELKKGKTKA FLSLARFSDN QYQRIENYMK
2401   SSEFENKQAL LKKAKEEVGL LRERRVQTNR YTVKVQRELE LDECAIHALT EDRQRFLCKA VENYISCLLS GEEHDMWIFR
2481   LCSLWLENSG VDRVNEMMKK NAEKIPSYKF LPLMYQLAAR MGTKMMGGLG FHEVLNNLMS RISLDHPHHT LFIILALANA
2561   NKDELLTKTD AKRINKLIKN APKEISQLDV DRMEAARNII NIIRKRRAHM VRDVEALCDA YITLANVDAT PWKTQRGGIS
```

2641 IPADQPIIKL KNLKDVVVPT MEIKVDPTGR YENLVTVMSF KPEFHLAGGL NLPKIIDCVG SDGKERRQLV KGRDDLRQDA
2721 VMQQVFQMCN TLLQQNTETR KRKLTIRRYK VVPLSQRSGV LEWCSGTTPI GEFLVNADKG AHKRYRPHDY SGFQCQKIMM
2801 DAQKKHSEEK YNTFMKVCDN FQPVFRYFCM EKFRDPAVWF EKRLAYTRSV ATSSIVGYIL GLGDRHVQNI LIDEQTAELV
2881 HIDLGVAFEQ GKILPTPETV PFRLTRDIVD GMGITGVEGV FRRCCEKTMA VMRNSQEALL TIVEVLLYDP LFDWTMNPLK
2961 ALYLQQGPED EADMSSTLGA DPQACKRKAS SDDQSFNKVA ERVLMRLQEK LKGVEEGTVL SVGGQVNLLI QQAMDPKNLS
3041 RLFPGWKPWV

5.366 heterogeneous nuclear ribonucleoprotein K [Gallus gallus]

Protein Accession gi|71897277
Mean Expression Ratio 1.02
Median Expression Ratio 1.02
Credible Interval (0.838, 1.24)
Associated Peptides 3
Associated Spectra 3
Coverage 0.0913

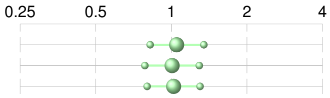

A	2.5	50	97.5	Sequence
1	0.82	1.0	1.3	TDYNASVSVPDSSGPER
1	0.79	1	1.3	IDEPLEGSEDR
1	0.8	1.0	1.3	NLPLPPPPPPR

1 METEQQEETF TNTETNGKRP AEDMEEEQAF KRSRNTDEMV ELRILLQSKN AGAVIGKGGK NIKALRTDYN ASVSVPDSSG
81 PERILSISAD TETIGEILKK IIPTLEEYQH YKGSDFDCEL RLLIHQSLAG GIIGVKGAKI KELRENTQTT IKLFQECCPH
161 STDRVVLIGG KPDRVVECIK IILDLISESP IKGRAQPYDP NFYDETYDYG GFTMMFDDRR GRPVGFPMRG RGGFDRMPPN
241 RGGRPMPPSR RDYDDMSPRR GPPPPPPGRG GRGGSRARNL PLPPPPPPRG GDLMSYDRRG RPGDRYDGMM MQCHVDACDD
321 MQPPELFEGG SGYDYSYAGG RGSYGDLGGP IITTQVTIPK DLAGSIIGKG GQRIKQIRHE SGASIKIDEP LEGSEDRIIT
401 ITGTQDQIQN AQYLLQNSVK QYSGKFF

5.367 adenylosuccinate synthase [Gallus gallus]

Protein Accession gi|71895783
Mean Expression Ratio 0.982
Median Expression Ratio 0.98
Credible Interval (0.765, 1.27)
Associated Peptides 1
Associated Spectra 1
Coverage 0.0310

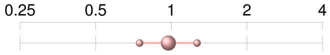

A	2.5	50	97.5	Sequence
1	0.75	0.97	1.3	TFDELPVNAQNYVR

1 MAEHGAPAPA IPNGGCAARL PGNKVTVVLG AQWGDEGKGK VVDLLAQDAD IVCRCQGGNN AGHTVVVDSV EYDFHLLPSG
81 IINPKVTAFI GNGVVIHLPG LFEETEKNLK KGKGLEGWEK RLVISDRAHI VFDFHQAADG IQEQQRQEQA GKNLGTTKKG
161 IGPVYSSKAA RSGLRMCDLV SDFDEFSERF KVLANQYKAI YPTLEIDIEG ELKKLKAYME KVKPMVKDGV YFMYEALHGP
241 PKKILVEGAN AALLDIDFGT YPFVTSSNCT VGGVCTGLGM PPQNVGEVYG VVKAYTTRVG IGAFPTEQDN EIGELLQMRG
321 KEFGVTTGRK RRCGWLDLVQ LRYAYMINGF TALALTKLDI LDVFPEIKVG VAYKLDGEVI PHFPANHEVL SKVEVKYETL
401 PGWDTDISNA RTFDELPVNA QNYVRFIEME LGVPVKWIGV GKSRESMIQL F

5.368 tropomyosin alpha chain, skin fibroblast - Japanese quail

Protein Accession gi|86150
Mean Expression Ratio 1.02
Median Expression Ratio 1.02
Credible Interval (0.819, 1.28)
Associated Peptides 2
Associated Spectra 2
Coverage NaN

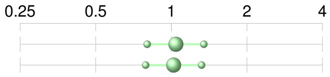

A	2.5	50	97.5	Sequence
1	0.8	1.0	1.3	LDKENALDR
1	0.79	1.0	1.3	AEQAEADKK

5.369 58kDa glucose regulated protein precursor [Gallus gallus]

Protein Accession	gi|45383890
Mean Expression Ratio	0.98
Median Expression Ratio	0.981
Credible Interval	(0.827, 1.17)
Associated Peptides	4
Associated Spectra	5
Coverage	0.119

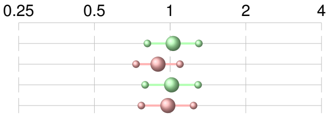

A	2.5	50	97.5	Sequence
**1**	**0.81**	**1.0**	**1.3**	**DGEESGTYDGPR**
**2**	**0.73**	**0.9**	**1.1**	**MDATANDVPSPYEVR**
**1**	**0.8**	**1.0**	**1.3**	**KTFGHELSEFGLDNSVGEAPVVAIR**
**1**	**0.77**	**0.98**	**1.2**	**KFLDAGHK**

```
1      MSVPRPSRAA LLLLVPLLAL SAGASDVVEL SDADFESGLA ERPGLVLVEF FAPWCGHCKR LAPEYEAAAT RLKGIVPLVK
81     VDCTANSNTC NKYGVSGYPT LKIFRDGEES GTYDGPRTAD GIVSHLKKQA GPASVALSSV ADFEKFIGDK DASVVGFFRD
161    ASGDAYSEFM KAANNLRDNY RFAHTSEEQL VQKYEEDGEG VVLYRPSRLA NKFEDSTVKY TEDKITSAKI KKFIQENIFG
241    ICPHMTEDNK DLIQGKDLLV AYYDVDYEKN AKGSNYWRNR VMMIAKKFLD AGHKLSFAVA SRKTFGHELS EFGLDNSVGE
321    APVVAIRTAK GDKFVMQEEF SRDGKALERF LQDYFDGNLK KYLKSEPVPE NNDGPVKVVV AENFDEIVNA EDKDVLIEFY
401    APWCGHCKNL EPKYKELGEK LSKDPNIVIA KMDATANDVP SPYEVRGFPT IYFAPAGKKQ SPKKYEGGRE VSDFISYLKR
481    EATSTPVLQE EDKAKKSKKK AKEDL
```

5.370 PREDICTED: similar to Acidic leucine-rich nuclear phosphoprotein 32 family member A (Leucine-rich a

Protein Accession	gi|118096008
Mean Expression Ratio	1.02
Median Expression Ratio	1.02
Credible Interval	(0.79, 1.32)
Associated Peptides	1
Associated Spectra	1
Coverage	0.0427

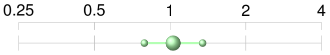

A	2.5	50	97.5	Sequence
**1**	**0.79**	**1.0**	**1.3**	**KREPEDEGDEDD**

```
1      MDMKKRIHLE LRNRTPSDVK ELVLDNCRSY EGKIEGLTDE FEELEFLSTI NVGLASVANL PKLNKLKKLE LSDNRVSGGL
81     EVLAEKCPNL THLNLSGNKI KDLGTIEPLK KLENLKSLDL FNCEVTNLND YRENVFKLLP QLTYLDGYDR DDKEAPDSDA
161    EGYVEGLDDE EEDEDVLSLV KDRDDKEAPD SDAEGYVEGL DDEEDLKGEE EYDDDAQVVE DEEDEEEEEE GEEEDVSGEE
241    EEDEEGYNDG DVDDDEDEEE PDEERGQKRK REPEDEGDED D
```

5.371 peptidylprolyl isomerase F [Gallus gallus]

Protein Accession	gi|71895031
Mean Expression Ratio	1.02
Median Expression Ratio	1.02
Credible Interval	(0.79, 1.32)
Associated Peptides	1
Associated Spectra	1
Coverage	0.0245

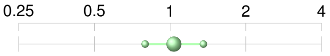

A	2.5	50	97.5	Sequence
**1**	**0.8**	**1.0**	**1.4**	**GFGYK**

```
1      MLVLSAARRL LRPHGELLPP GLACVRAAGA RGCSSDGAGP RNPLVYLDVG ADNQPLGRVV LELKADVVPK TAENFRALCT
81     GEKGFGYKGS TFHRVIPSFM CQGGDFTNHN GTGGKSIYGS RFPDENFLLK HEGPGVLSMA NAGPNTNGSQ FFICTAKTDW
161    LDGKHVVFGH VKEGMDVVKK IESFGSKNGK TSKKIVITDC GQLS
```

5.372 ARP1 actin-related protein 1 homolog A, centractin alpha [Gallus gallus]

Protein Accession	gi|56118984
Mean Expression Ratio	0.98
Median Expression Ratio	0.981
Credible Interval	(0.781, 1.22)
Associated Peptides	2
Associated Spectra	2
Coverage	0.0745

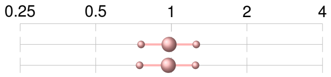

A	2.5	50	97.5	Sequence
1	0.76	0.98	1.3	AQYYLPDGSTIEIGSAR
1	0.75	0.97	1.3	YCFPNYVGRPK

```
1    MESYDVIANQ PVVIDNGSGV IKAGFAGDQI PKYCFPNYVG RPKHVRVMAG ALEGDIFIGP KAEEHRGLLS IRYPMEHGIV
81   KDWNDMERIW QYVYSKDQLQ TFSEEHPVLL TEAPLNPRKN RERAAEVFFE TFNVPALFIS MQAVLSLYAT GRTTGVVLDS
161  GDGVTHAVPI YEGFAMPHSI MRIDIAGRDV SRFLRLYLRK EGYDFHTTSE FEIVKTIKER ACYLSINPQK DETLETEKAQ
241  YYLPDGSTIE IGSARFRAPE LLFRPDLIGE ECEGLHEVLV FAIQKSDMDL RRTLFSNIVL SGGSTLFKGF GDRLLSEVKK
321  LAPKDVKIRI SAPQERLYST WIGGSILASL DTFKKMWVSK KEYEEDGARA IHRKTF
```

5.373 PREDICTED: hypothetical protein [Gallus gallus]

Protein Accession	gi|118095485
Mean Expression Ratio	1.02
Median Expression Ratio	1.02
Credible Interval	(0.815, 1.27)
Associated Peptides	2
Associated Spectra	2
Coverage	0.0584

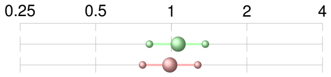

A	2.5	50	97.5	Sequence
1	0.82	1.1	1.4	ASNPAPSLEVAASSSSR
1	0.77	0.99	1.3	KLHAVVETLVNHR

```
1    MASGGAGGSR SPSPGPHLPP PFPPSCGDGD AADSDTEGED IFAGTSKPPA PKRELLLPVN NSSKENGVRV EQDDQDLFAD
81   ATVELSLDST QNNQKKELAK ASNPAPSLEV AASSSSRNPP KSYEELEEEE QEDKFELTVG VSDPEKVGDG MNAYVAYKVS
161  TQTSMPMFRS KQFSVKRRFS DFLGLYEKLS EKHAQNGFIV PPPPEKSLIG MTKVKVGKED SSSAEFLEKR RAALERYLQR
241  VVSHPTMLQD PDVREFLEKE ELPRAIGTQA LSGAGILKMF NKATDAVSKM TIKMNESDIW FEEKLQEVEC EEQRLRKLHA
321  VVETLVNHRK ELALNTAQFA KSLAMLGSSE DNTALSRALS QLAEVEEKIE QLHQEQANND FFVLAELLGD YIRLLSVVRG
401  AFDQRMKTWQ RWQDAQTMLQ KKREMEARLL WANKPDKLQQ AKEEISEWES RVTQYERDFE RISAVIRKEV IRFEKEKSKD
481  FRNHVTKYLE TLLNSQQQLV KYWEAFLPEA KAIS
```

5.374 PREDICTED: similar to Deoxythymidylate kinase (thymidylate kinase) [Gallus gallus]

Protein Accession	gi|118094933
Mean Expression Ratio	1.02
Median Expression Ratio	1.02
Credible Interval	(0.786, 1.30)
Associated Peptides	1
Associated Spectra	1
Coverage	0.066

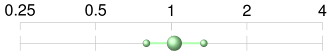

A	2.5	50	97.5	Sequence
1	0.8	1.0	1.3	TTEIGQLISAYLAR

```
1    MAGGRGALIV LEGMDRAGKS TQCRRLVQAL QAAGHRADLL RFPERTTEIG QLISAYLARE RELEDHAVHL LFSANRWEHV
81   TLMKEKLHQG ITLVVDRYAF SGVAFTSAKE NFCLEWCKQT DAGLPKPDLI LFLQLSPEEA AARGNFGGER YENGAFQEKV
161  LQSFRHLMKE KTLNWKTMDA SKSIEDLHRE IKTVAEKTMQ EVKDEPLGEL WK
```

5.375 hypothetical protein LOC426942 [Gallus gallus]

Protein Accession	gi|71897165
Mean Expression Ratio	1.02
Median Expression Ratio	1.02
Credible Interval	(0.79, 1.32)
Associated Peptides	1
Associated Spectra	1
Coverage	0.157

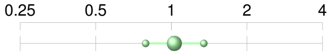

A	2.5	50	97.5	Sequence
**1**	**0.8**	**1.0**	**1.3**	**DQEQVEIEGENSAPPR**

```
1       MADFLKGLPV YNKSNFSRFH ADSVCKASNR RPSVYLPTRE YPSEQIIVTE KTNILLRYLH QQWDKKNAAK KRDQEQVEIE
81      GENSAPPRKI ARTDSQDMNE DT
```

5.376 heat shock 70kDa protein 5 precursor [Gallus gallus]

Protein Accession	gi|45382769
Mean Expression Ratio	1.02
Median Expression Ratio	1.02
Credible Interval	(0.833, 1.24)
Associated Peptides	3
Associated Spectra	3
Coverage	0.0706

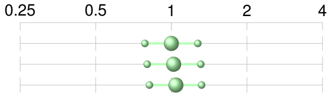

A	2.5	50	97.5	Sequence
**1**	**0.78**	**1**	**1.3**	**LYGSAGPPPTGEEEAAEKDEL**
**1**	**0.8**	**1.0**	**1.3**	**FEELNMDLFR**
**1**	**0.82**	**1.0**	**1.3**	**KSDIDEIVLVGGSTR**

```
1       MRHLLLALLL LGGARADDEE KKEDVGTVVG IDLGTTYSCV GVFKNGRVEI IANDQGNRIT PSYVAFTPEG ERLIGDAAKN
81      QLTSNPENTV FDAKRLIGRT WNDPSVQQDI KYLPFKVVEK KAKPHIQVDV GGGQTKTFAP EEISAMVLTK MKETAEAYLG
161     KKVTHAVVTV PAYFNDAQRQ ATKDAGTIAG LNVMRIINEP TAAAIAYGLD KREGEKNILV FDLGGGTFDV SLLTIDNGVF
241     EVVATNGDTH LGGEDFDQRV MEHFIKLYKK KTGKDVRKDN RAVQKLRREV EKAKRALSSQ HQARIEIESF FEGEDFSETL
321     TRAKFEELNM DLFRSTMKPV QKVLEDSDLK KSDIDEIVLV GGSTRIPKIQ QLVKEFFNGK EPSRGINPDE AVAYGAAVQA
401     GVLSGDQDTG DLVLLDVCPL TLGIETVGGV MTKLIPRNTV VPTKKSQIFS TASDNQPTVT IKVYEGERPL TKDNHLLGTF
481     DLTGIPPAPR GVPQIEVTFE IDVNGILRVT AEDKGTGNKN KITITNDQNR LTPEEIERMV NDAEKFAEED KKLKERIDAR
561     NELESYAYSL KNQIGDKEKL GGKLSSEDKE TIEKAVEEKI EWLESHQDAD IEDFKSKKKE LEEVVQPIVS KLYGSAGPPP
641     TGEEEAAEKD EL
```

5.377 nuclear distribution gene C homolog [Gallus gallus]

Protein Accession	gi|57529473
Mean Expression Ratio	0.982
Median Expression Ratio	0.982
Credible Interval	(0.76, 1.27)
Associated Peptides	1
Associated Spectra	1
Coverage	0.0205

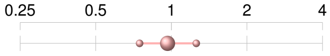

A	2.5	50	97.5	Sequence
**1**	**0.75**	**0.97**	**1.3**	**KQDILKK**

```
1       MGLSAEEAAE QEDRFDGILL AMAQQHQGGV CELVNTFFSF LRRKTDFFTG GEDGVAEKLI TDSFNHHNKL AQKERKEKKA
81      RQEAERREKA ERAAKLAKEA KQEANEPRIK ELTDEEAERL QLEIDQKKEA QKEVNNVPVK SSEDDGDSSD SNKQETDDEE
161     KDENDKGKLK PNAGNGADLP NYRWTQTLSE LDLAIPFKVT FRLKGKDVVV DIQRRRLRVG LKGHPPVIDG ELFNEVKVEE
241     SSWLIEDGKT VTVHLEKINK MEWWNKLVST DPEINTKKIN PENSKLSDLD SETRSMVEKM MYDQRQKSMG LPTSDEQKKQ
321     DILKKFMEQH PEMDFSKAKF N
```

5.378 zyxin [Gallus gallus]

Protein Accession	gi|52138675
Mean Expression Ratio	1.02
Median Expression Ratio	1.02
Credible Interval	(0.814, 1.27)
Associated Peptides	2
Associated Spectra	2
Coverage	0.0554

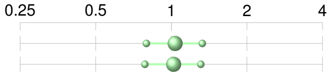

A	2.5	50	97.5	Sequence
**1**	**0.8**	**1.0**	**1.3**	**TGGTSESSQPQPPGTGAQR**
**1**	**0.79**	**1.0**	**1.3**	**KFAPVVAPKPK**

```
1       MASPGTPGTR MTTTVSINIS TPSFYNPQKK FAPVVAPKPK VNPFKTGGTS ESSQPQPPGT GAQRAQIGRV GEIPVSVTAE
81      ELPLPPPPPP GEELSFSSNC AFPPPPPPFE EPFPPAPDEA FPSPPPPPPP MFDEGPALQI PPGSTGSVEK PLAPKAHVEI
161     SSAPRDPTPP FPSKFTPKPS GTLSSKPPGL DSTPAPAPWA APQQRKEPLA SVPPPPSLPS QPTAKFTPPP VASSPGSKPG
241     ATVPMAPSNS TRYPTSLQTQ FTAPSPSGPL SRPQPPNFTY AQQWERPQVQ EKPVPTEKSA AVKDMRRPTA DPPKGNSPLT
321     MKEVEELELL TQKLMKDMDH PPPVEAATSE LCGFCRKPLS RTQPAVRALD CLFHVECFTC FKCEKQLQGQ QFYNVDEKPF
401     CEDCYAGTLE KCSVCKQTIT DRMLKATGNS YHPQCFTCVM CHTPLEGASF IVDQANQPHC VDDYHRKYAP RCSVCSEPIM
481     PEPGKDETVR VVALEKNFHM KCYKCEDCGR PLSIEADENG CFPLDGHVLC MKCHTVRAKT AC
```

5.379 PREDICTED: hypothetical protein [Gallus gallus]

Protein Accession	gi|50758444
Mean Expression Ratio	1.02
Median Expression Ratio	1.02
Credible Interval	(0.79, 1.32)
Associated Peptides	1
Associated Spectra	1
Coverage	0.0902

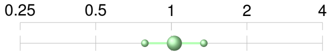

A	2.5	50	97.5	Sequence
**1**	**0.78**	**1.0**	**1.4**	**GYSEFVQTAQR**

```
1       MGWEEKQVRG YSEFVQTAQR YHGRPIFALF CGDKDAEGRS WCPDCVTAEP VVRKELHNMP DESVFIYCLV GDRAYWKDPN
81      NEFRKNLKLT GVPTLLKYGT PQKLVEEECF KADLVRMLFT ED
```

5.380 PREDICTED: similar to Phosphopantothenoylcysteine synthetase [Gallus gallus]

Protein Accession	gi|118101250
Mean Expression Ratio	0.982
Median Expression Ratio	0.983
Credible Interval	(0.758, 1.26)
Associated Peptides	1
Associated Spectra	1
Coverage	0.0221

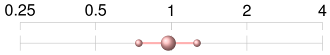

A	2.5	50	97.5	Sequence
**1**	**0.74**	**0.97**	**1.3**	**LTPGPPPGVAADPDALPALLPALR**

```
1       MAAAAKVEAA AAAAEERVRV WAAAQVASGR RVALVTSGGT QVPLETRAVR FLENFSSGRR GAVSAECLVS AGYGVCFLHR
81      ARSVFPWARV LPPPGPALLD AFRLTPGPPP GVAADPDALP ALLPALRAYR EAADAGALLS LEFTDLNEYL ALLRATARAL
161     APFGSGVMFY LAAAVSDFYI PASEMPEHKI QSSEGALQIT MKMVPKMLSP LVKEWAPEAF VISFKLETDP LILIDKSRQP
241     PEMAEDVRRW LQAEGADPGD PAEEQLGLAW RLLWRAEARL GDVDSAAGRG AERSKGRNHG MKRPACPVER YVGHVRSLSQ
321     ERDAVIAECE RDNERLGLEL VRLRLQHDRT ELQKAMEHNS RLDKEILALR ARVQTLDLER KTFLDLKVWR CREVIENIEG
401     RNSQLLHKLH KLEQEHEDLV ERNEELESIL GETQIQTKQD KEQFESEVEG LHRKVSELVR ENEQVNKLKH QREDVSVDGK
481     AYEEQMAKVV FLEEQIRNLT DEQEQLCSEL LESNKKREEL EKQLKESNEE KQSLLEEIAQ LKQDILTTRE QHDSTFEEAL
561     RMNQGTVHRD NRFLTSQSSA GSLDGSIKQS LSEERFQQQE ERLQQLRHDL RRVQNLCSSA ERELRYEREK NIDLQKQNLL
641     LQQECTKVKA ELKQARTKLL DSTETCSSLS AQWEKSQQKV KELEQELLKC SQADKLQSSL QEKLVQEKSK VYEAQKQISK
721     IQQKLKDSQH QLLLAEARVS DKKLLEEELK EARENEARVQ QELREELLKR KLLEQQVEEL RQQLRHSHET EASLAKMHVE
801     LQAKTLHALE DDKKTDSGEH LQCQKESQKL SEQLSLLKEE NKALYEEGVR LLNQKDLYVR KYNEMQLRHK EKIRRAKATF
881     IHEVKQRDSR IKQLENELSE SKLQVEKGKA LIAQITTENE KLLQERRRLL QRITDQEETP WSNRSTVAAL QSRMKILDEE
961     NMRLHESKLH LSGHMHTSQR TLRSIHTPST EDLKSANFSE RQLQSKVSAS SPRASFPPRE PPDSLSSLKP VHDTKPEGGA
1041    ESQDSSFCLS PSQPSEIGYL NVASPGDTTA SQLQEESQSI SSENF
```

5.381 PREDICTED: hypothetical protein isoform 1 [Gallus gallus]

Protein Accession	gi|118083576
Mean Expression Ratio	1.02
Median Expression Ratio	1.02
Credible Interval	(0.788, 1.31)
Associated Peptides	1
Associated Spectra	1
Coverage	0.06

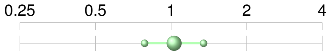

A	2.5	50	97.5	Sequence
**1**	**0.78**	**1.0**	**1.3**	**AVDNQVYVATVSPAR**

```
1       MANFRLALIQ LHVSAVKSDN LQRACGLIRE ASAKGAKVVA LPECFNSPYG TQYFKEYAEK IPGESTQKLS AVAKECSIYL
81      VGGSIPEEDG GKLYNTCTVF GPDGAILAKH RKIHLFDINV PGKIQFKESE TLSPGDSFSM FDTRCQLLIY PGAFNMTTGP
161     AHWELLQRGR AVDNQVYVAT VSPARDEKAS YVAWGHSTVV NPWGEVIAKA GAEETVIYTD IDLKKLAEIR QQIPILSQKR
241     YDLYGIEMKK
```

5.382 phosphofructokinase, platelet [Gallus gallus]

Protein Accession	gi|71895711
Mean Expression Ratio	0.983
Median Expression Ratio	0.983
Credible Interval	(0.761, 1.27)
Associated Peptides	1
Associated Spectra	1
Coverage	0.023

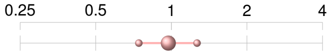

A	2.5	50	97.5	Sequence
**1**	**0.74**	**0.97**	**1.3**	**MFAVIDGFEGFAR**

```
1       MDHQPKFFEN LSGTGKAIGV LTSGGDAQGM NAAVRAVVRM GIYVKAKVYF VYEGYQGMVD GGDNIVEVSW ESVSSILQVG
81      GTVIGSARCK SFRTREGRLQ AAYNLVRRGI TNLCVIGGDG SLTGANLFRE EWSGLLEELA QKGKIDEEAV KKYAYLNIVG
161     MVGSIDNDFC GTDMTIGTDS ALHRIIEVVD AIMTTAQSHQ RTFVLEVMGR HCGYLALVSA LACGADWVFI PEYPPEEGWE
241     DSMCVKLSEN RARKKRLNII IVAEGAIDCH NKPITSEKVK DLVVQRLGFD TRVTILGHVQ RGGTPSAFDR ILASRMGVEA
321     VLALLEATPD TPACVVSLSG NQAVRLPLME CVQMTQEVQK AMDEGRFVEA VRLRGRSFEN NLNTYKLLSQ KKPDAELPKS
401     NFNVAVLNVG APAAGMNAAV RAAVRVGITE GHKMFAVIDG FEGFARGKIK EISWGDVGGW TGQGGSILGT KRTLPAKYLE
481     KIADQMRTNN INALMVIGGF EAYLGLLELS AAREKYDEFC VPMVMVPATV SNNVPGSDFS IGADTALNTI TDLQNMGKNV
561     EKMKI
```

5.383 PREDICTED: similar to CGI-83 protein, partial [Gallus gallus]

Protein Accession	gi|118122584
Mean Expression Ratio	0.984
Median Expression Ratio	0.983
Credible Interval	(0.775, 1.25)
Associated Peptides	1
Associated Spectra	2
Coverage	0.0526

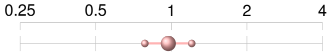

A	2.5	50	97.5	Sequence
**2**	**0.78**	**0.97**	**1.2**	**DGDVIQTEGATLR**

```
1       MASLLPRIER LSSRVVRVLG CNPGPMTLQG TNTYLVGTGL RRILIDTGEP AIPEYIGCLK QALSEFNISI QEILVTHWHR
81      DHTGGIPDIC TNIPNDSEYR ICKLPRVPHC EEIIGDGGHK YLYLKDGDVI QTEGATLRVL YTPGHTDDHM SLHLEEENAI
161     FSGDCILGEG TTVIEDLYDY MKSLKMLLQM KPDLIYPGHG PVVRDANARI QGYISHRNAR EEQILNVFQK NAGKSYTSSE
241     LVKIVYK
```

5.384 PREDICTED: similar to Tsg24 protein [Gallus gallus]

Protein Accession	gi|118087531
Mean Expression Ratio	1.01
Median Expression Ratio	1.02
Credible Interval	(0.782, 1.31)
Associated Peptides	1
Associated Spectra	1
Coverage	0.00514

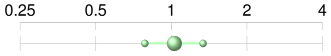

A	2.5	50	97.5	Sequence
**1**	**0.78**	**1.0**	**1.3**	**LGTMDMAITR**

```
1       MSDHSEERAT MIAAGDLQEF IPRGREHCRR HPGAHNLQPR ELQPASELCS SDGAAGLVGS LREVTIHERQ RESWQLRKGV
81      SDFGEEVDYD EELYVASNMV IWSKGSKTEA SAAYKAFSVD SPVLQALWCD FTIQQEKSDK ADGGKEIVQK CVCILQSSCV
161     NVHSIDGKDY VAALPFQVAN IWPTKFGLLL ERSGSSHEVP PSPPREPLPT MFSMLHPLDE ITPLVCKSGG VFGSARVQYV
241     ADYTLRIVFV NAEPSIVMTY DTVQSLHTVW ALRRVKPEEQ NMVLKFSEQL GTPQNVAASS SLTAQLRSLS KGDSPMASPF
321     QNYSSIHSQS RSVSSPSMQS RSPSISNMAA LSRSHSPALG VHSFSGVQRF NSSSNIQSPR RHSITHSPES TCSDSFLVTE
401     TEPIVPELCI DHLWTEAITK MREKNSQASK VFITTDLCGQ KFLCFLVESQ LQLRCVKFQE SNDKSQLIFG SVTNIQAKDA
481     APVQGVDTLL VLEGSGNLVL YSGIVRVGKV FIPGLPAPSL TMSNQMPRPS TPLDSVSTPS KTLNKHLGPL EESMLLSPVP
561     ELRDSSKLHD STYVEDCTFQ QHGTFIHALR DPVHNRVTLE LSSNTMVRIT IPEIATSELV KTCLQGVKAI LPKEIAVQML
641     VKWYNAYNAP GGPSYRLEWN LFVTCLMNMM GYNTEKLTWT RNLNVGGSPS PVSAPKKARP SETGSDEDWE YLLSSDYHRN
721     FESHPLSRVL GLDPLEVLAP KDDFLQSLSL DYSTLLFTHI PAIFFVLHLV YEEFKLNLLM GEENRSLVVL LVQLARDLKL
801     EAYIDYYYRD YPALVKSSGQ TCIIDQGQTG FMHHPAFFSA EPPSIFQWLS SCLKGGSVQP YPYLPGICER SKLVILSVAL
881     YVLGDESAVS NEASPYLYKI TSGQQKQQIE HDDNRCCFGH SASISSLAEK LIIWLTNIGF TLRDLETLPF GVALPIRDAI
961     YYCREQPASD WPEAVCVLIG RQDLSRQACD GNLLKSKSVM LSSDSPSGAE SEEDEDGMNN MNDEVMSLIW SKDLRVQEVR
1041    RLLQSARPVR VNVVQMPEAS DHDFIEEKEN RLLQLCQRTM ALPVGRGMFT LFSYHPIPAE QLPIPKLNLT GRAPPRNSTV
1121    DLNSGNIDVP PNMACWASFH NGVAAGLKIA PASRIDSSWI IYNKPKNAEQ ANEYAGFLMA LGLSGHLTKL ATLNIHDYLT
1201    KGHEMTSIGL LLGVSAAKLG TMDMAITRLL SIHIPALLPP TSTELDVPHN VQVAAVIGIG LVYQGTAHRH TAEVLLAEIG
1281    RPPGPEMEYC TDRESYSLAS GLALGMVCLG HGSNLIGMSD LNVPEQLYQY MVGGHRRFQA GMHREKHKSP SYQIKEGDTI
1361    NVDVTCPGAT LAFAMIYLKT NNRSIADWLQ APDTMYLLDF VKPEFLLLRT LARCLILWDD IMPSTEWVDS NVPQIIRENS
1441    IPLHATELPS SEDLSLETLM QAHVYIIAGA CLSLGFRFAG SENKAAFNCL DKYATDFLKS LSAPTASIIG HYNLETCLSV
1521    LLLSLAMVVA GSGNLKVLQL CRFMHKKTGG EMNYGFHLAH HMALGLLFLG GGRYSLSTSN SSIAALLCAL YPHFPVHSTD
1601    NRYHLQALRH LYVLAADPRL LIPVDVDSNT PCYALIEVTF KGTQWYAETT EEMMAPTLLP ELHLLKQIRV KGPRYWELLI
1681    DLSNETNHLK SILSKGGVLY VKLRAGQLSY KEDPMGWRSF SAQTITHGNE ARSFKPEDIS AFTSDPALLL FAEYFCKPTV
1761    NMGQKQEMLD FFSSILYECV TQEKPEMLPT YIAIDQAVRR LERGEMSETF ELGQIKLVLE FFNSRCHQGR MSRNPNRGLF
1841    MNSEFLPVMK CTIDKALDQW LQAGGDACLR SYLSRQPKDE SQQNMLACFL VYHSVPMPGQ LAAGGLDGIT NFSEMLLKFK
1921    QWNVLVRALL RLAPVLLKNP QSMVL
```

5.385 PREDICTED: hypothetical protein [Gallus gallus]

Protein Accession	gi|118100390
Mean Expression Ratio	0.984
Median Expression Ratio	0.984
Credible Interval	(0.764, 1.27)
Associated Peptides	1
Associated Spectra	1
Coverage	0.0085

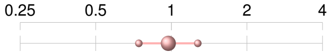

A	2.5	50	97.5	Sequence
**1**	**0.74**	**0.97**	**1.3**	**YLTQQAVALQR**

```
1       MPPAAVPASA QGGEQQEPGP RGGRAAEAEQ RPEHLPGVAL VNGSGPHENL KGEKDARQNG HEEADAGEDG NDQEVIVIQD
81      TGFTVKICAP GIEPFSLQVS PQEMVQEIHQ VLMDREDTCH RTCFSLQLDG NVLDNFAELK TIEGLQEGSL LKVVEEPYTV
161     REARIHVRHI RDLLKSLDPS DAFNGVDCNS LSFLSVFTEG DLGDSGKRKK KGTEMEQIDC TPPEHILPGS KERPLCALQP
241     QNRDWKPLQC LKVLTMSGWN PPPGNRKMHG DLMYLYVITV EDRHVSITAS TRGFYLNQST AYNFNPKPAN PSFLSHSLVE
321     LLNQISPTFK KNFSALQKKR VQRHPFERIA TPFQVYSWTA PQAEHAMDCV RAEDAYTSRL GYEEHIPGQT RDWNEELQTT
401     RELPRKNLPE RLLRERAIFK VHSDFTAAAT RGAMAVIDGN VMAINPSEET KMQMFIWNNI FFSLGFDVRD HYKDFGGDVA
481     AYVAPTNDLN GVRTYNAVDV EGLYTLGTVV VDYRGYRVTA QSIIPGILER EQEQSVIYGS IDFGKTVVSH PKYLELLEKT
561     SRPLKIQKHK VLNDKNEEVE LCSSVECKGI IGNDGRHYIL DLLRTFPPDL NFLPVEGEEM PEECKKMGFP KQHRHKLCCL
641     RQELVDAFVE HRYLLFMKLA ALQLMQQKAN KQESSAALEN GTSPENGTAE SEKSESDDGK TDDSVTGLDQ VKELAETIAS
721     DDGTVDPKSR EVIRNACKAV GSISDTSFDI RFNPDIFSPG VRFPESSREE VQDQKQLLKD AAAFLLSCQI PGLVKDCLDH
801     TVLPMDGATL AEAMHQRGIN MRYLGKVIHF ITKTPGHAQL DHIFKIGISE LITRSAKHIF KTYLQGVELS GLSAAISHFL
881     NCFLSSFPNP IAHLPADELV SKKKNKKRKN RNLGNADNTA WASMTPQELW KNICSEAKSY FDFSLECENA DQAAEMYNLQ
961     KITLLREISL KTGVQILLKE YNFDNRHKPT FTEEDILNIF PVVKHVNPKA SDAFHFFQSG QAKVQQGFLK EGCELINEAL
1041    NLFNNVYGAM HVEICACLRL LARLNYIMGD YSEALSNQQK AVLMSERVLG IEHPNTIQEY MHLALYCFAN SQLSTALNLL
1121    YRARYLMLLV FGEDHPEMAL LDNNIGLVLH GVMEYDLSLR FLENALAISS KYHGSKSLKV ALSHHLVARV YESKAEFRSA
1201    LQHEKEGYTI YKNQLGEHHE KTKESSEYLK YLTQQAVALQ RTMNEIYKNG SNANIVPLKF TAPNMTSVLE QLNIINGILF
1281    IPLRSSCRAT EGC
```

5.386 Retinal dehydrogenase 2 (RalDH2) (RALDH 2) (RALDH(II)) (Retinaldehyde-specific dehydrogenase type 2

Protein Accession	gi|92087020
Mean Expression Ratio	0.983
Median Expression Ratio	0.984
Credible Interval	(0.763, 1.27)
Associated Peptides	1
Associated Spectra	1
Coverage	0.0232

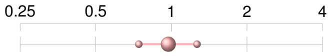

A	2.5	50	97.5	Sequence
**1**	**0.74**	**0.97**	**1.3**	**EEIFGPVQEILR**

```
1     MTSSKIEMPG EVKADPAALM ASLHLLPSPT LNLEIKHTKI FINNEWQNSE SGRVFPVYNP ATGEQICEIQ EADKVDTDKA
81    VRAARLAFSL GSVWRRMDAS ERGQLLDKLA DLVERDRAVL ATMESLNSGK PFLQAFYVDL QGVIKTLRYY AGWADKIHGM
161   TIPVDGDYFT FTRHEPIGVC GQIIPWNFPL LMFAWKIAPA LCCGNTVVIK PAEQTPLSAL YMGALIKEAG FPPGVVNILP
241   GFGPIVGAAI ASHVGIDKIA FTGSTEVGKL IQEAAGRSNL KRVTLELGGK SPNIIFADAD LDYAVEQAHQ GVFFNQGQCC
321   TAGSRIYVEE SIYEEFVRRS VERAKRRVVG SPFDPTTEQG PQIDKKQYNK ILELIQSGIT EGAKLECGGK GLGRKGFFIE
401   PTVFSNVTDD MRIAKEEIFG PVQEILRFKT VDEVIERANN SDFGLVAAVF TNDINKALTV SSAMQAGTVW INCYNALNAQ
481   SPFGGFKMSG NGREMGESGL REYSEVKTVT IKIPQKNS
```

5.387 PREDICTED: similar to TFG protein [Gallus gallus]

Protein Accession	gi|50729668
Mean Expression Ratio	0.984
Median Expression Ratio	0.984
Credible Interval	(0.76, 1.28)
Associated Peptides	1
Associated Spectra	1
Coverage	0.0608

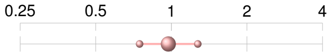

A	2.5	50	97.5	Sequence
**1**	**0.75**	**0.97**	**1.3**	**NVMSAFGLTDDQVAGPPSAPAEER**

```
1     MNGQLDLSGK LIIKAQLGED IRRIPIHNED ITYDELVLMM QRVFRGKLLS NDEVTIKYKD EDGDLITIFD SSDLSFAIQC
81    SRILKLTLFV NGQPRPLESN QVKYLRRELI ELRNKVNRLL DCLEPPAEPG VSTNLPESDA VDGREEKPAA ADSNVKPSTQ
161   VIAASMSAFD PLKNQDEISK NVMSAFGLTD DQVAGPPSAP AEERSGTPDS IASSSSAAHP PGVQPQQAPY PGAQPQTGQQ
241   VEGQMYQQYQ QPGYPAQQPQ AQPQQQYGVQ YPAGYSPQQA ASQPTQQFPA YSQQPAPAAA FPGQGQAQQL PAQQPQQYPA
321   GSFPPQPYTT QASQPAPYSG PPGAQAAPGT FQPRPGFTPP PGSTMTPPPS GPNPYARTRP PFGPQGYAQP GPGYR
```

5.388 PREDICTED: similar to cdc21p, partial [Gallus gallus]

Protein Accession	gi|118126814
Mean Expression Ratio	0.984
Median Expression Ratio	0.984
Credible Interval	(0.761, 1.27)
Associated Peptides	1
Associated Spectra	1
Coverage	0.0553

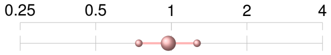

A	2.5	50	97.5	Sequence
**1**	**0.74**	**0.97**	**1.3**	**SQLLQYVYNLVPR**

```
1     IYRAVPIRIN PRVSVVKSVY KTHIDVIHYR KTDSKRLHGV DEETEQKMFT EERVAFLKEL STKADIYERL SSALAPSIYE
81    HEDIKKGILL QLFGGSRKDF THTGRGNFRA EINILLCGDP GTSKSQLLQY VYNLVPRGQY TSGKGSSAVG LTAYVMKDPE
161   TRQLVLQTGA LVLSDNGICC IDEFDKMNES TRSVLHEVME QQTLSIAKVG VCLSPSSLFY TSAQGRRNQE VNQSQ
```

5.389 cystatin C [Gallus gallus]

Protein Accession	gi|45382805
Mean Expression Ratio	1.02
Median Expression Ratio	1.02
Credible Interval	(0.788, 1.32)
Associated Peptides	1
Associated Spectra	1
Coverage	0.122

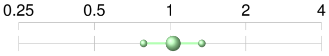

A	2.5	50	97.5	Sequence
**1**	**0.78**	**1.0**	**1.3**	**LLGAPVPVDENDEGLQR**

```
1       MAGARGCVVL LAAALMLVGA VLGSEDRSRL LGAPVPVDEN DEGLQRALQF AMAEYNRASN DKYSSRVVRV ISAKRQLVSG
81      IKYILQVEIG RTTCPKSSGD LQSCEFHDEP EMAKYTTCTF VVYSIPWLNQ IKLLESKCQ
```

5.390 aspartyl-tRNA synthetase [Gallus gallus]

Protein Accession	gi|55741590
Mean Expression Ratio	1.02
Median Expression Ratio	1.02
Credible Interval	(0.792, 1.31)
Associated Peptides	1
Associated Spectra	1
Coverage	0.0199

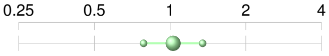

A	2.5	50	97.5	Sequence
**1**	**0.79**	**1.0**	**1.3**	**IYVISLAEPR**

```
1       MPSAAGRGQG KDRRPDNEEQ PAADDYAKER YGVSSMIQSQ EKPDRVLVRI KDQTEEKADE VVWVRGRVHT SRAKGKQCFL
81      VLRQQQFNIQ ALVAVGQHAS KQMVKFAANI NKESIVDVEG VVRKAHQKIG GCTQQDVELH VQRIYVISLA EPRLPLQLDD
161     AVRPEVEGEE DGRATVNQDT RLDNRVIDLR TSTSQAVFCL QSGICQLFRE TLIRKGFVEI QTPKIISAAS EGGANVFTVS
241     YFKSSAYLAQ SPQLYKQMCI CADFEKVFCV GPVFRAEDSN THRHLTEFVG LDIEMAFNYH YHEVVDEIAD TLVQIFKGLQ
321     ERFQAEIQMV NKQFPCEPFK FLEPTLRLEY REAVAMLREA GIEMGDEEDL STPNEKLLGR LVKEKYDTDF YILDKYPLAV
401     RPFYTMPDPV NPKNSNSYDM FMRGEEILSG AQRIHDPQLL TERAKHHGID LEKIKAYIDS FRFGAPPHAG GGIGLERVTM
481     LYLGLHNVRQ TSMFPRDPKR LTP
```

5.391 Ubiquitin

Protein Accession	gi|51701919
Mean Expression Ratio	1.01
Median Expression Ratio	1.02
Credible Interval	(0.786, 1.31)
Associated Peptides	1
Associated Spectra	1
Coverage	0.171

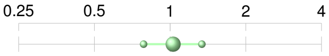

A	2.5	50	97.5	Sequence
**1**	**0.78**	**1.0**	**1.3**	**IQDKEGIPPDQQR**

```
1       MQIFVKTLTG KTITLEVEPS DTIENVKAKI QDKEGIPPDQ QRLIFAGKQL EDGRTLSDYN IQKESTLHLV LRLRGG
```

5.392 PREDICTED: similar to hepatoma-derived growth factor (high-mobility group protein 1-like), partial

Protein Accession	gi|118107483
Mean Expression Ratio	1.02
Median Expression Ratio	1.02
Credible Interval	(0.845, 1.22)
Associated Peptides	3
Associated Spectra	5
Coverage	0.142

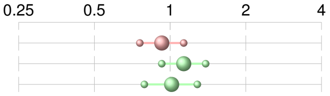

A	2.5	50	97.5	Sequence
2	0.76	0.92	1.1	TCPEDAEPEQEPEGDGEK
2	0.93	1.1	1.4	TCPEDAEPEQEPEGDGEKK
1	0.8	1.0	1.3	FGKPNKR

```
1       MSYHCALLSP HPFLHVVQID EMPEAAVKSS SNKYQVFFFG THETAFLGPK DLFPYEECKE KFGKPNKRKG FSEGLWEIEH
81      NPTVKASGYQ PTQKKTCPED AEPEQEPEGD GEKKGNAEGS SDEEGKLVID EQSKEKNEKA GIKRKAEDVL EDSPKGTKEM
161     EGQEAEKKTD NEEAPKEEPK PNP
```

5.393 PREDICTED: similar to Prdx3 protein [Gallus gallus]

Protein Accession	gi|118093103
Mean Expression Ratio	0.984
Median Expression Ratio	0.984
Credible Interval	(0.76, 1.27)
Associated Peptides	1
Associated Spectra	1
Coverage	0.0490

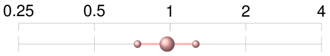

A	2.5	50	97.5	Sequence
1	0.74	0.97	1.3	DYGVLLEGPGIALR

```
1       MTPSFLPRPL RDPPPLVLPP GDGSEPSTAL VPPPVPCIPS PPPGPACCAA LKMAAALRGL LRRAVPAAGR TLTAQPLLCA
81      RRRLTLGASR LAPAVTQHAP FFKGTAVVNG EFKELTLDDF KGKYLVLFFY PLDFTFVCPT EIVAFSNKAN EFHDVNCEVV
161     AVSVDSHFCH LAWINTPRKS GGLGKMNIPV LSDLTKQISR DYGVLLEGPG IALRGLFIID PNGIIKHLSI NDLPVGRSVE
241     ETLRLVKAFQ YVETHGEVCP ANWTPDSPTI KPSPEASKEY FEKVHT
```

5.394 PREDICTED: similar to Band 4.1-like protein 3 (4.1B) (Differentially expressed in adenocarcinoma of

Protein Accession	gi|118086838
Mean Expression Ratio	0.986
Median Expression Ratio	0.984
Credible Interval	(0.763, 1.27)
Associated Peptides	1
Associated Spectra	1
Coverage	0.0139

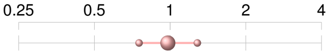

A	2.5	50	97.5	Sequence
1	0.75	0.98	1.3	AEEEKDDEEGK

```
1       MRVRCLSFQD SEGVEIMLGV CASGLLIYRD RLRINRFAWP KVLKISYKRN NFYIKIRPGE FNPFQITFDG NVIHHPMLIP
81      VRSVCFRLLL PEAPPKKFLT LGSKFRYSGR TQAQTRRASA LIDRPAPYFE RSSSKRYTMS RSLDGEVGTG QYATTKGISQ
161     TNLITTVTPE KKAEEEKDDE EGKKKRAEEV TPISAVRHDT KPPFLGTDSH QSSPSPQPGP HASSAKLRRR CKESARKKPL
241     GCESPKEGAS KQGEAEPDSD RKGRVCSTEQ DVAFGYKQKA GKGGTLFSFS LQLPDSFPSL LDDDGYLSLP NLSETNLLPA
321     SVQHYLPIRS PSLVPCFLFI FFFLLSASFS VPYALTLSFP LAMCLCYLEP KAASLSASLA NDLSDSSEEE TDSEQTDTAA
401     DGETTATESD QEEDGDLKAQ DLDKTQEDLM KHQTNISELK RTFLETSTET AVSNEWEKRL STSPVRLAAR QEEAPMIEPL
481     VPEETKQSTG EKTMDGSDIF SLIESARKPT EFIGGVTTTS HSWAQRIDTT TSQEVTSSEL KQAAQPHQDA VTKVVQETVV
561     IEERRGMNVH ASGDPASVAG LADAQAQAAS ASAKGKEGSA VTKGAKEEKR EAHKAVTKQE GIAAATSHEQ AEEHSTTVHV
641     SDSLERKPRF ESPVVKTETI SFSSVSTGGE NLEISTKEVP VVHTETKTIT YESSQVDSGA DSEPGVLMSA QTITSETTST
721     TTTTHITKTV KGGISETRIE KRIVITGDAD IDHDQALAQA IKEAKEQHPD MSVTKVVVHK ETEITPEDGE D
```

5.395 hypothetical protein LOC422173 [Gallus gallus]

Protein Accession	gi|71895327
Mean Expression Ratio	0.983
Median Expression Ratio	0.984
Credible Interval	(0.76, 1.27)
Associated Peptides	1
Associated Spectra	1
Coverage	0.0717

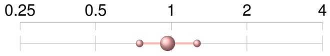

A	2.5	50	97.5	Sequence
1	0.75	0.97	1.3	FYPEDVAEELIQDITQR

```
1       MPKTISVRVT TMDAELEFAI QPNTTGKQLF DQVVKTIGLR EVWFFGLQYQ DTKGFSTWLK LNKKVTAQDV RKESPLLFKF
81      RAKFYPEDVA EELIQDITQR LFFLQVKEAI LNDDIYCPPE TAVLLASYAV QSKYGDFNKD VHKSGYLASD KLLPQRVLEQ
161     HKLNKDQWEE RIQVWHEEHP LPSLTRNRAA LEPLQKSDHG LIYRVILVNA GVRQQKKERH LHIPGVAWES KLVLGQI
```

5.396 PREDICTED: similar to Eukaryotic translation elongation factor 1 delta (guanine nucleotide exchange

Protein Accession	gi|118087445
Mean Expression Ratio	1.01
Median Expression Ratio	1.02
Credible Interval	(0.785, 1.31)
Associated Peptides	1
Associated Spectra	1
Coverage	0.0118

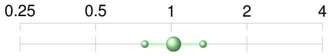

A	2.5	50	97.5	Sequence
1	0.78	1.0	1.3	FKYDDAER

```
1       MRTRKPPCPI EKVWVDKHKY DEAERLHYER EAMLATVAPE ECLELEAVNG VCNDDSAEGE FKGDLKKGKN GKKQRKRKRS
81      PKPKNMVSKV DSVLSGLLAD SVWFDKPLYD HAESLYRQKL VDCQYQEAAE TELPESEAVQ DAPKPWMLPA ALSCSHGNLS
161     ACHHVVQGVW VNKFDFDKAE KAFLERSQFF VPPNILAIPS VCSDSGNVGL GTPDEGYVTA LPTPATPSLA PDVVNSTPFV
241     SSSPPSAEPT VNGKPQASSL QALMSEVWLE KPLYDGAEKS FYENMFDSHP PDSPQASEDH QEVGESPSVE KQEGAEHVEL
321     SSSSQQPPPT SFFLHEDSER VWLNKMTYDS AESRYYEAEA LKMSRAKEST GMPQPTVVKS QPDAEAPSIP APEKKKMAVD
401     FLSNENIWFE KFKYDDAERR FYEQQVNGPA GSSSHQQNGA STILRDIARA RENIQKSLAG SASTSSSGPG DQNELLSRIS
481     HLEVENQNLH SVVADLQKAI FKLESRLNAL EKSTSHQPSP VPPTQHITLM KKVEPFGVPS KKPAAAEEDD DDDDINLFGS
561     DDEEEDQEAA KVREERLRQY AEKKAKKPGL IAKSSILLDV KPWDDETDMA KMEECVRSVQ MDGLVWGASK LVPVGYGIKK
641     LQIQCVVEDD KVGTDILEEE ITKFEDYVQS VDIAAFNKI
```

5.397 hook homolog 1 [Gallus gallus]

Protein Accession	gi|57529629
Mean Expression Ratio	1.02
Median Expression Ratio	1.02
Credible Interval	(0.786, 1.31)
Associated Peptides	1
Associated Spectra	1
Coverage	0.0153

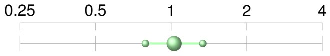

A	2.5	50	97.5	Sequence
1	0.79	1.0	1.3	ERLVIQCDALK

```
1       MEGKAADPLL CDSLILWLQT FNTAAPCRNV QDLTNGVAMA QVLHQIDVAW FDASWLNRIK EDVGDNWRIK SSNLKKILQG
81      IMDYYHEFLD QQISEELIPD LNKISENSDP TELGRLMQLI LGCAVNCERK QEHIQNIMTL EESVQHVVMT AIQELMSKEA
161     MGPSASDVSS EMEQQLKKAL EDLQEAIAEK EELAQRCQEL DLQVAALQDE KNSLVSENEI LNDRLEQLDD SLDDPNTVVA
241     KKYFHAQLQL EQLQEENFRL EAAKDDYRVH CEDLEKQLIE LQHRNNELTS LAEESRALKD ENDILRAAAD KASKLESTVE
321     VYRKKLQDLN DFRRQVKSLQ ETNMMYMHNT VSLEDELREA NAARAQLETY KRQVQELHNK LSEESKRADK LAFEMKRLEE
401     KHEALVKEKE RLVIQCDALK ETNEELRYSQ MQQDHLSRTD ASRIKSHDNL AAELLPVEYR EMFIQLQHEN KMLLLQQEGS
481     ENERIMELQK QLEQKQWTVN ELGTEKRLNK ERIGELQQQI EDLQKTLQEQ GSKTEGSSNL KQKLAAHMEK LSEVHDELQK
561     KEAALAELQP DVSQNPQKIG ELEAALRKKD EDMKAMEERY KMYLEKARNV IKTLDPKLNP ASAEIMLLRK QITERDKKIE
641     ALEAEYKLAK LRDYEENLIV TAWYNKSLTL QKLGMEARLL GSGGACRDGP GRSFLAQQRH VTNTRRNLPV KVPSATSD
```

5.398 tubulin, beta 5 [Mus musculus]

Protein Accession	gi|7106439
Mean Expression Ratio	1.02
Median Expression Ratio	1.02
Credible Interval	(0.923, 1.12)
Associated Peptides	14
Associated Spectra	32
Coverage	0.387

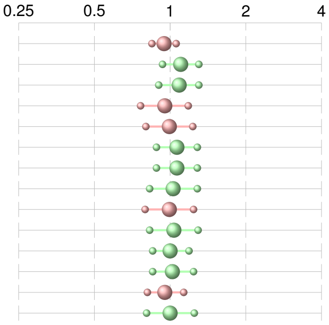

A	2.5	50	97.5	Sequence
9	0.84	0.94	1.1	AILVDLEPGTMDSVR
3	0.93	1.1	1.3	YLTVAAVFR
2	0.9	1.1	1.3	EVDEQMLNVQNK
1	0.76	0.95	1.2	FPGQLNADLR
1	0.8	1	1.2	TAVCDIPPR
2	0.88	1.1	1.3	ALTVPELTQQVFDAK
2	0.88	1.1	1.3	LAVNMVPFPR
1	0.83	1.0	1.3	ISVYYNEATGGK
1	0.8	1	1.2	NSSYFVEWIPNNVK
1	0.83	1.0	1.3	LHFFMPGFAPLTSR
3	0.85	1	1.2	GHYTEGAELVDSVLDVVR
2	0.85	1.0	1.2	LTTPTYGDLNHLVSATMSGVTTCLR
3	0.81	0.96	1.1	IREEYPDR
1	0.8	1	1.2	KLAVNMVPFPR

```
1       MREIVHIQAG QCGNQIGAKF WEVISDEHGI DPTGTYHGDS DLQLDRISVY YNEATGGKYV PRAILVDLEP GTMDSVRSGP
81      FGQIFRPDNF VFGQSGAGNN WAKGHYTEGA ELVDSVLDVV RKEAESCDCL QGFQLTHSLG GGTGSGMGTL LISKIREEYP
161     DRIMNTFSVV PSPKVSDTVV EPYNATLSVH QLVENTDETY CIDNEALYDI CFRTLKLTTP TYGDLNHLVS ATMSGVTTCL
241     RFPGQLNADL RKLAVNMVPF PRLHFFMPGF APLTSRGSQQ YRALTVPELT QQVFDAKNMM AACDPRHGRY LTVAAVFRGR
321     MSMKEVDEQM LNVQNKNSSY FVEWIPNNVK TAVCDIPPRG LKMAVTFIGN STAIQELFKR ISEQFTAMFR RKAFLHWYTG
401     EGMDEMEFTE AESNMNDLVS EYQQYQDATA EEEEDFGEEA EEEA
```

5.399 PREDICTED: similar to hephaestin [Gallus gallus]

Protein Accession	gi|118089267
Mean Expression Ratio	1.02
Median Expression Ratio	1.02
Credible Interval	(0.804, 1.29)
Associated Peptides	1
Associated Spectra	2
Coverage	0.0072

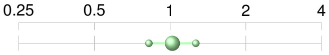

A	2.5	50	97.5	Sequence
2	0.83	1.0	1.3	VCRKGALR

```
1       MPVIVAFSQR KKASVFLQPG KDRIGGTYKK SVYKQYTDST YTTEIPKPAW LGFLGPIIRA EVGDTIKVHL KNFASRPYTI
81      HPHGVFYEKG SEGSLYPDMS PQDQKKDDAV FPGGSYTYTW TVPEDHSPTA DDPNCLTWIY HSHIDAPKDI ASGLIGPLVT
161     CKEGILTGTS QRRQDVDVDF FLMFSVVDEN LSWYLDENIA SFCTDPGSVD KEDEEFQESN KMHAINGFVF GNLPALTMCA
241     GDHVAWHLFG MGNEIDIHTA YFHGETLSIR GHRTDVASLF PATFVTADMI PGNPGRWLLS CQLNDHIQAG MAAIYEVRPC
321     SRQAPEPTLQ GRIRKYYIAA KEVQWDYGPS GRDSSSGKQL SEAGSPAEPF FKRSLYRIGG VYWKVKYVEY TDESFREEKQ
401     HSEEEKHLGI LGPVIKAEVG DTILVTFANK ASWPFSIQPH GVSYGKAWEG MRYHDGVSQN GVSVPPLHNF TYTWTVPSHA
481     GPTSSDPPCL TWMYSSAVDP IRDTSSGLVG PLVICKPGTL DDNNKQKGID KEFYLLFSVF DENLSWYLSA NIKYYLRMEE
561     TSLKKDDGFE ESNRMHAING FMFGNLPGLD ICEGDNVSWH LLGLGSEADV HGAVFQGNTL KMNGMRKDSA NLFPHTFATA
641     FMQPDNKGTF EIYCQTSNHY QAGMREQYSV SNCNRRALSP AVPYTAVRTY YIVAEEMEWD YAPDRSWERE RHNHSAESYA
721     DIFLSNKDGL IGSRYKKAVY REYTDGTFQT PKARINGDEH LGILGPFLWA EVGEILNVVF KNNASRPYSI HAHGVLERQT
801     GQPQVAHPGD IVTYQWEIPE RSGPGPDDSA CVPWIYYSMV DPVKDMYSGL IGPLKVCRKG ALRADGIRKD VKREFALLFL
881     VFDENQSWYL EENVKRYSSG SHKDIDLLDE KFVESNKMHA INGRLYANLP GLTMFEGEWV NWYLLGMGQE IDVHTVHFHA
961     ETFIYKSGKS YRADVVDLFP GTFEMVEMLA GNPGTWLLHC HVSDHIHAGM EILFQVLPRQ EPVPAVLNYN EEAQPEDEDD
1041    SQKVRLFGAK LPLGQVEAAV ITLAVIGLVL LLIAAFLLGV VIYLRRQRKL RLNRRSILDD GFKLMSKKNS GL
```

5.400 PREDICTED: similar to E3 ubiquitin-protein ligase HECTD1 (HECT domain-containing protein 1) (E3 lig

Protein Accession	gi|118091807
Mean Expression Ratio	1.02
Median Expression Ratio	1.02
Credible Interval	(0.786, 1.31)
Associated Peptides	1
Associated Spectra	1
Coverage	0.00383

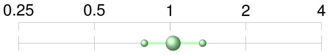

A	2.5	50	97.5	Sequence
1	0.79	1.0	1.3	CPFLIPFETR

```
1      MADVDPDTLL EWLQMGQGDE RDMQLIALEQ LCMLLLMSDN VDRCFETCPP RTFLPALCKI FLDESAPDNV LEVTARAITY
81     YLDVSAECTR RIVGVDGAIK ALCNRLVVVE LNNRTSRDLA EQCVKVLELI CTRESGAVFE AGGLNCVLTF IRDSGHLVHK
161    DTLHSAMAVV SRLCGKMEPQ DSSLEICVES LSSLLKHEDH QVSDGALRCF ASLADRFTRR GVDPAPLAKH GLTEELLSRM
241    AAAGGTASGP SSACKPGRTS TGAPSTAADS KLSNQVSTIV SLLSTLCRGS PVVTHDLLRS ELPDSIESAL QGDERCVLDT
321    MRLVDLLLVL LFEGRKALPK SSAGSTGRIP GLRRLDSSGE RSHRQLIDCI RSKDTDALID AIDTGAFEVN FMDDVGQTLL
401    NWASAFGTQE MVEFLCERGA DVNRGQRSSS LHYAACFGRP QVAKTLLRHG ANPDLRDEDG KTPLDKARER GHSEVVAILQ
481    SPGDWMCPVN KGDEKKKKDA NKDEEECNEP KGDPEMAPIY LKRLLPVFAQ TFQQTMLPSI RKASLALIRK MIHFCSEALL
561    KEVCDSDAGH NLPTILVEIT ATVLDQEDDD DGHLLALQII RDLVDKGGDL FLDQLARLGV ISKVSTLAGP SSDDENEEES
641    KPEKEDEPQE DAKELQQGKP YHWRDWSIIR GRDCLYIWSD AAALELSNGS NGWFRFILDG KLATMYSSGS PEGGSDSSES
721    RSEFLEKLQR ARSQVKPSTA SQPILSAPGP TKLTVGNWSL TCLKEGEIAI HNSDGQQATI LKEDLPGFVF ESNRGTKHSF
801    TAETSLGKYV GMYDAQVGAC IKAVLQLLLS RVRTMARDLY DDHFKAVESM PRGVVVTLRN IATQLESAWE LHTNRQCIEG
881    ENTWRDLMKT ALENLIVLLK DENTISPYEM CSSGLVQSLL TVLNNVSLXN ISLXRKAFEI ERINVFKTAF SENEDDESRP
961    AVALIRKLIA VLESIERLPL HLYDTPGSTY NLQILTRRLR FRLERASGET SLIDRTGRML KMEPLATVES LEQYLLKMVA
1041   KQWYDFDRAS FVFVRKLREG QTFVFRHQHD FDENGIIYWI GTNAKTAYEW VNPAAYGLVV VTSSEGRNLP YGRLEDILSR
1121   DSSALNCHTN DDKNAWFAID LGLWVIPSAY TLRHARGYGR SALRNWVFQV SKDGQNWTTL YTHVDDCSLN EPGSTATWPL
1201   DPPKDEKQGW RHVRIKQMGK NASGQTHYLS LSGFELYGTV NGVCEDQLGK AAKEAEANLR RQRRLVRSQV LKYMVPGARV
1281   IRGIDWKWRD QDGSPQGEGT VTGELHNGWI DVTWDAGGSN SYRMGAEGKF DLKLAPGYDP DSAASPKPVS STVSGTTQSW
1361   SSLVKNNCPD KTTAAAGSSS RKGSSSSVCS VASSSDISLG STKMERRSES VMEQNIVSGT DVHEPIVVLS SADNVPQAEV
1441   GSSSSASTST LTADMGNENT ERKLGPDNSI RTPGESSAIS MGIVSVSSPD VSSVSELTNK EAASQRPLSS SASNRLSVSS
1521   LLAAGAPMSS SASVPNLSSR ETSSLESFVR RVANIARTNA TNNMNLSRSS SDNNTNTLGR NVMSTATSPL MGAQSFPNLT
1601   TTGTTSTVTM STSSVTSSSN VATATTVLSV GQSLSNTLTT SLTSTSSESD TGQEAEYSLY DFLDSCRAST LLAELDDDED
1681   LPEPDEEDDE NEDDNQEDQE YEEVMVRYKT STSXYAEIVS IVTYXMVSXT LVKIPRTRSA KENEEEEYET KGGRRRTWDD
1761   DYVLKRQFSA LVPAFDPRPG RTNVQQTTDL EIPPPGTPHS ELLEEVECMP SPRLALTLKV SGLGTTREVE LPLTNFRFTI
1841   FYYVQKLLQL SCNGSVKSDK LRRIWEPTYT IMYREMKDSD KEKESGKMGC WSVEHVEQYL GTDELPKNDL ITYLQKNADS
1921   AFLRHWKLTG TNKSIRKNRN CSQLIAAYKD FCEHGSKSGL SQGAISTLQN SDILSLAKEQ PQAKAGSGQN SCGVEDVLQL
2001   LRILYIVASD PYTARTSQEE GDEHPQFNFP PDEFTSKKIT TKILQQIEEP LALASGALPD WCEQLTSKCP FLIPFETRQL
2081   YFTCTAFGAS RAIVWLQNRR EATVERTRTT STVRRDDPGE FRVGRLKHER VKVPRGESLM EWAENVMQIH ADRKSVLEVE
2161   FLGEEGTGLG PTLEFYALVA AEFQRTDLGA WLCDDDFPDD ESRQVDIGGG LKPPGYYVQR SCGLFTAPFP QDSDELERIT
2241   KLFHFLGIFL AKCIQDNRLV DLPISKPFFK LMCMGDIKSN MSKLIYESRS DRDLHCTESQ SEASTEEGHD SLSVGSLEED
2321   SKSEFILDPP KPKPPAWFNG ILTWEDFELI NPHRARFLKE IKDLAIKRRQ ILSNKSLSED EKNTKLQELM LKNPSGSGPP
2401   LSIEDLGLNF QFCPSSKVYG FTAVDLKPGG EDETVTMDNA EEYVDLMFDF CMHTGIQKQM EAFRDGFNRV FPMEKLSSFS
2481   HEEVQMILCG NQSPSWAAED IINYTEPKLG YTRDSPGFLR FVRVLCGMSS DERKAFLQFT TGCSTLPPGG LANLHPRLTV
2561   VRKVDATDAS YPSVNTCVHY LKLPEYSSEE IMRERLLAAT MEKGFHLN
```

5.401 nucleophosmin 1 [Gallus gallus]

Protein Accession	gi|45383996
Mean Expression Ratio	0.987
Median Expression Ratio	0.985
Credible Interval	(0.793, 1.23)
Associated Peptides	2
Associated Spectra	2
Coverage	0.0918

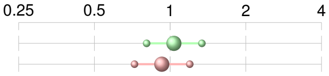

A	2.5	50	97.5	Sequence
1	0.81	1.0	1.3	VIQALWQWR
1	0.72	0.93	1.2	EYQFKVDDEENEHQLSLR

```
1      MEDSAMDMES MGPLRPQTFL FGCELKAEKE YQFKVDDEEN EHQLSLRTVT LGAGAKDELH VVEAEALDYE GNPTKVVLAS
81     LKMSVQPTVS LGGFEITPPF VLRLKCGSGP VYVSGQHLVA LEEEPESEDE EEDTKIGNAS TKRPASGGGA KTPQKKPKLS
161    EDDEDDDEDE DDDEDDEDDL DDDEEEIKTP MKKPAREPAG KNMQKAKQNG KDSKPSTPAS KTKTPDSKKD KSLTPKTPKV
241    PLSLEEIKAK MQASVDKGCS LPKLEPKFAN YVKNCFRTED QKVIQALWQW RQTL
```

5.402 signal transducer and activator of transcription 5B [Gallus gallus]

Protein Accession	gi|45382145
Mean Expression Ratio	0.987
Median Expression Ratio	0.985
Credible Interval	(0.762, 1.29)
Associated Peptides	1
Associated Spectra	1
Coverage	0.0203

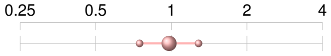

A	2.5	50	97.5	Sequence
1	0.75	0.98	1.3	LQAQFSQLSQLGPQER

```
1       MAVWIQAQQL QGEALRQMQA LYGHHFPIEV RHYLSQWIES QAWDSIDLDN PQENVKATQL LEGLIQELQK KADHQVGEDG
81      FLLKIKLGHY VTQLQNTYDR CPMELVRCIR HILYHEQRLV REANNSPSPA GTLVDAMSQK HLQINQTFEE LRLITQDSEN
161     ELKKLQQTQE YFIIQYQENM RLQAQFSQLS QLGPQERLSR ETTLQQKKAS LEAWLHREAQ TLQQYRVELA EKHQKTLQLL
241     RKQQTTILDD ELIQWKRRQQ LAGNGGPPEG TLDVLQTWCE KLAEIIWQNR QQIRRAEHLC QQLPIPGPVE EMLSELNGTI
321     TDIISALVTS TFIIEKQPPQ VLKTQTKFAA TVRLLVGGKL NVHMNPPQVK ATIISEQQAK ALLKNESTRN ESSGEILNNC
401     CVMEYHQATG TLSAHFRNMS LKRIKRSDRR GAESVTEEKF TILFESQFSV GGNELVFQVK TLSLPVVVIV HGSQDNNATA
481     TVLWDNAFAE PGRVPFAVPE KVQWPQLCEA LNMKFKAEVQ SSRGLTKENL VFLAQKLFNS TSSHLEDYSS TTVSWAQFNR
561     ENLPGRNYTF WQWFDGVMEV LKKHLKPHWN DGAILGFVNK QQAHDLLISK PDGTFLLRFS DSEIGGITIA WKFDSAERMF
641     WNLMPFTTRD FSIRSLADRL GDLSYLIYVF PDRPKDEVFS KYYTPVLCES TPAKAVDGYV KPQIKQVVPE FVSASGDAVP
721     GGGTYMDQAP SPAVCSHPHY NMYTQNPETV LDPEGDFDLD DTMDVAQHVE ELLRRPMDSQ WIPHAQS
```

5.403 tumor protein D52-like 2 [Gallus gallus]

Protein Accession	gi|71894971
Mean Expression Ratio	0.986
Median Expression Ratio	0.985
Credible Interval	(0.76, 1.27)
Associated Peptides	1
Associated Spectra	1
Coverage	0.112

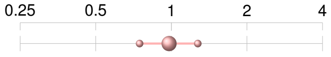

A	2.5	50	97.5	Sequence
1	0.75	0.98	1.3	ENSTEGLQSPSAGDKPPQDNAPF

```
1       MESASQDISL NSPNKGLLSD AMMDVPVDSA AAARTATPEG LSAAEEEELR AEIAKVEEEI GTLRQVLAAK ERHCGELKRK
81      LGLTPLDGLK QNLSKSWHDV QVSNAYVRTS EKLGEWNDKV TQSDFYKKTQ ETLSQAGQKT SAALSNVGSV ISRKLGDMRN
161     SATFKSFEDR VGTIKSRVVG SRENSTEGLQ SPSAGDKPPQ DNAPF
```

5.404 PREDICTED: similar to polyglutamine-containing protein [Gallus gallus]

Protein Accession	gi|118091987
Mean Expression Ratio	0.985
Median Expression Ratio	0.985
Credible Interval	(0.765, 1.27)
Associated Peptides	1
Associated Spectra	1
Coverage	0.0206

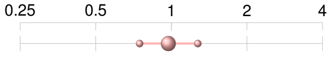

A	2.5	50	97.5	Sequence
1	0.75	0.97	1.3	APEDGPPELNR

```
1       MCIFLQEVKY RKGRESAETE LRADNRTPPQ RGICGNQSHD AKGGRAGRGR RRPSPENSGR APAASGARSG YGPSFQLRPA
81      GEERGDSGSG GVGSPASGTM SAAQVSSSRR QSCYLCDLPR MPWAMIWDFT EPVCRGCVNY EGADRIEFVI ETARQLKRAH
161     GCFQDGRSPG PPPPVGVKAV PLSAKEAAAA AAAAQQQLNH VDASSKAASG LSQSGLERYG LSAAAAEQRG RFEYPPPPGS
241     LGGGHGARLP NGLGGPNGFP KAPEDGPPEL NRQSPNSSSS SSSSRRGAHG GLVSGLPPGA AGAQLNVPPN LLPQTLLNGP
321     AASGVALPPP HGGLSGRGGG GPPAPSASSQ GGACDGGSGS GGGGGPMSVA DTLGNAHSPK DGSSVHSTTS TRRNSSSPVS
401     PASVPGQRRL ASRNGDMNLQ VAPPPPSAHP AMDQVHPQNI PDSPMANSGP LCCTICHERL EDTHFVQCPS VPSHKFCFPC
481     SRESIKAQGA TGEVYCPSGE KCPLVGSNVP WAFMQGEIAT ILAGDVKVKK ERDP
```

5.405 acyl-Coenzyme A binding domain containing 3 [Gallus gallus]

Protein Accession	gi|71895595
Mean Expression Ratio	0.986
Median Expression Ratio	0.985
Credible Interval	(0.765, 1.28)
Associated Peptides	1
Associated Spectra	1
Coverage	0.0443

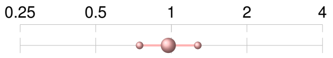

A	2.5	50	97.5	Sequence
1	0.75	0.98	1.3	AGPGCPSQAEAGGEEAAALSPER

```
1      MAAALSAERL EVSVDGLTLS PNAEAPPCEA RPGHGDSAAG RNRAGPGCPS QAEAGGEEAA ALSPERRWGF ALEELYGLAL
81     RFFKEKDGKA FHPTYEEKLR LVALHKQVLL GPYNPDTCPE VGFFDVLGND RRKEWAALGN MSKQEAMTEF VKLLNRCCHL
161    FSTYVTSHKI EKEEQEKKRR EEEERRRREE EELERLQKEE EKRRQEEEER LRREEEERRR IEEERLRMEQ QKQQIMAALN
241    SQTAMQFQQY AAQQYPGNYE QQQILIRQLQ EQHYQQYMQQ LYQVQLAQQQ AALQKQQEAV VAAAGTPLTT ASKVNVPAQG
321    DMPSINGQAS AHTDNPEKEL DPEALEEALE NGPKDSVPVI AAPSMWARPQ IKDFKEKIRQ DADSVITVGR GEVVTVRVPT
401    HEEGSYLFWE FATDSYDIGF GVYFEWTDSP NTAVSVHVSE SSDDEDEEEE NASSEEKAKK NANKPQLDEI VLVYRRDCHE
481    EVYAGSHQYP GRGVYLLKFD NSYSLWRSKT VYYRVYYTR
```

5.406 nucleolin [Gallus gallus]

Protein Accession	gi|45384000
Mean Expression Ratio	0.986
Median Expression Ratio	0.986
Credible Interval	(0.789, 1.24)
Associated Peptides	2
Associated Spectra	2
Coverage	0.0231

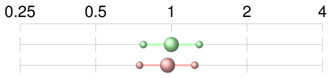

A	2.5	50	97.5	Sequence
1	0.77	1	1.3	AAVTPAKK
1	0.75	0.97	1.2	KAVAPSPK

```
1      MVKLAKTPKN QMKQKKMAPP PKKVEESEEE ESSDLEESSG EEVMVPPKKQ QKAAVTPAKK AATPAKKAAT PAKKAVTPAK
81     KAVATPAKKA VAPSPKKAAV VGKGAKNGKN AKKEESEEED EDDEDDEEDE DEEEESDEEE EPAVPVKPAA KKSAAAVPAK
161    KPAVVPAKQE SEEEEEEDDE EEDEEDDESE DEAMDTTPAP VKKPTPAKAT PAKAKAESED EEDEEDEDED EEDEDDEEED
241    EEESEDEKPV KEAPGKRKKE MANKSAPEAK KKKTETPASA FSLFVKNLTP TKDYEELRTA IKEFFGKKNL QVSEVRIGSS
321    KRFGYVDFLS AEDMDKALQL NGKKLMGLEI KLEKAKSKES LKENKKERDA RTLFVKNLPY RVTEDEMKNV FENALEVRLV
401    LNKEGSSKGM AYIEFKTEAE AEKALEEKQG TEVDGRAMVI DYTGEKSQQE SQKGGGERES KTLIVNNLSY AASEETLQEL
481    FKKATSIKMP QNNQGRPKGY AFVEFPTAED AKEALNSCNN TEIEGRAIRL EFSSPSWQKG NMNARGGFNQ QSKTLFVRGL
561    SEDTTEETLR ESFEGSISAR IVTDRDTGSS KGFGFVDFSS PEDAKAAKEA MEDGEIDGNK VTLDFAKPKG EFQRGGGFGG
641    GFGGRGGRGG RGGGRGGFGG RGGGRGFGGR GGGFRGGRGG GGDHKPQGKK IKFE
```

5.407 heat shock 70kD protein binding protein [Gallus gallus]

Protein Accession	gi|71896903
Mean Expression Ratio	0.987
Median Expression Ratio	0.986
Credible Interval	(0.802, 1.22)
Associated Peptides	2
Associated Spectra	3
Coverage	0.0886

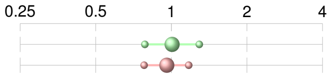

A	2.5	50	97.5	Sequence
1	0.78	1	1.3	QNPGLLHTEELGFLR
2	0.78	0.96	1.2	LLGHWEEAAHDLALACK

```
1      MDSRKLGELR AFVRLCKQNP GLLHTEELGF LREWVESMGG TIPPAPASTS TDETSKGKAE EQPEEPVKSP EPESEESDLE
81     IDNEGVIEPD NDDPQEMGDE NVEVTEEMMD QANEKKMEAI NALSEGDLQK AVNLFTDAIK LNPCLAILYA KRASVFVKLQ
161    KPNAAIRDCD RAIKINPDSA QTYKWRGKAH RLLGHWEEAA HDLALACKLD YDEDASAMLK EVQPRAQKIA EHRRKYERKR
241    EEKEIKERME RVKKAREEHE RAQREEEARR QAGGAQFGGF PGGFPGGFPG AMPGGMPGMA GMPGLNEILS DPEVLAAMQD
321    PEVMAAFQDV AQNPANMSKY QNNPKVMSLI TKLSAKFGSK P
```

5.408 glutathione S-transferase class-alpha [Gallus gallus]

Protein Accession	gi|4959550
Mean Expression Ratio	0.986
Median Expression Ratio	0.986
Credible Interval	(0.789, 1.23)
Associated Peptides	2
Associated Spectra	2
Coverage	0.129

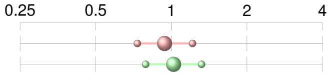

A	2.5	50	97.5	Sequence
1	0.73	0.94	1.2	DVPAVLAIFS
1	0.79	1.0	1.3	VLKDHGHDFLVGNK

```
1      KTKDDLQKLR TDGFLLFQQV PMVEIDGMKL VQTRAILNYI AGKYNLYGKD LKERALIDMY VEGLADLNEL ILHHEFKPAN
81     EMEKDLANIL DKATNRYLPV FEKVLKDHGH DFLVGNKLSK ADVHLLENIL WLEELKPDVL AKFPLLQSFK ARMSNMPNIK
161    KFLQPGSPKK PIVQEKDVPA VLAIFS
```

5.409 eukaryotic translation initiation factor 4A2 [Gallus gallus]

Protein Accession	gi|45383077
Mean Expression Ratio	0.987
Median Expression Ratio	0.986
Credible Interval	(0.777, 1.25)
Associated Peptides	1
Associated Spectra	2
Coverage	0.0344

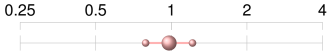

A	2.5	50	97.5	Sequence
2	0.8	0.98	1.2	KGVAINFVTEEDKR

```
1      MSGGSADYSR DHGGPEGMEP DGVIESNWNE IVDNFDDMNL KESLLRGIYA YGFEKPSAIQ QRAIIPCIKG YDVIAQAQSG
81     TGKTATFAIS ILQQLEIDLK ETQALVLAPT RELAQQIQKV ILALGDYMGA TCHACIGGTN VRNEMQKLQA EAPHIVVGTP
161    GRVFDMLNRR YLSPKWIKMF VLDEADEMLS RGFKDQIYEI FQKLSTNIQV VLLSATMPMD VLEVTKKFMR EPIRILVKKE
241    ELTLEGIKQF YINVEREEWK LDTLCDLYET LTITQAVIFL NTRRKVDWLT EKMHARDFTV SALHGDMDQK ERDVIMREFR
321    SGSSRVLITT DLLARGIDVQ QVSLVINYDL PTNRENYIHR IGRGGRFGRK GVAINFVTEE DKRILRDIET FYNTTVEEMP
401    MNVADLI
```

5.410 PREDICTED: similar to cellular apoptosis susceptibility protein [Gallus gallus]

Protein Accession	gi|118100584
Mean Expression Ratio	0.986
Median Expression Ratio	0.986
Credible Interval	(0.82, 1.18)
Associated Peptides	3
Associated Spectra	5
Coverage	0.0381

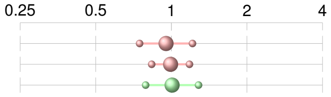

A	2.5	50	97.5	Sequence
1	0.75	0.95	1.2	DLEGSDIDTR
3	0.84	1	1.2	AADEEAFEDNSEEYIR
1	0.79	1	1.3	YDEEFQPYLPR

```
1      MELSDANLQT LTEYLKKTLD PDPAIRRPAE KFLETVEGSQ NYPLLLLTLL EKSQENVIKV CASVTFKNYI KRNWRIVEDE
81     PNKIFESDRI AIKANIVPLM LSSPEQIQKQ LSDAISIIGR EDFPQKWPDL LTEMVNRFQS GDFHVINGVL RTAHSLFKRY
161    RHEFKSNELW TEIKLVLDAF ALPLTNLFKA TIDLCSTHAN GASALKVLFS SLIXIKMHIY AFPSQDLPEF FEDNMETWMT
241    NFHSLLTLDN KLLQTDDEEE AGLLELLKSQ ICDNAALYAQ KYDEEFQPYL PRFVTAIWNL LVTTGQEVKY DLLVSNAIQF
321    LASVCERPHY KHLFEDQNTL TSICEKVIVP NMEFRAADEE AFEDNSEEYI RRDLEGSDID TRRRAACDLV RGLCKFFEGP
401    VTGIFSGYVN SMLQEYAKNP SVNWKHKDAA IYLVTSLASK AQTQKHGITQ ANELVNLTEF FVNHIQPDLK SASVNEFPVL
481    KADGIKYIMI FRNQVPKEQL LLSIPLLINH LQAESIVVHT YAAHALERLF TMRGTNNTTL ITAAEMAPFV EVLLTNLFKA
561    LTLPGSSENE YIMKAIMRSF SLLQESIIPY IPSVITQLTQ KLLAVSKNPS KPHFNHYMFE SICLSIRITC KANPDAVGSF
641    EEALFMVFTE ILQNDVQEFI PYVFQVMSLL LEMHKNEIPS SYMALFPHLL QPVLWERTGN IPPLVRLLQA YLERGANTIA
721    SAAADKIPGL LGVFQKLIAS KANDHQGFYL LNSIIEHMPP ESVDQYRKQI FILLFQRLQN SKTTKFIKSF LVFINLYCVK
801    YGALALQEIF DSIQPKMFGM VLEKIIIPEI QKVSGQVEKK ICAVGITKIL TECPPMMDTE YTKLWTPLLQ ALIGLFELPE
881    DDTIPDEEHF IDIEDTPGYQ TAFSQLAFAG KKEHDPVGQM VNNPRIHLAQ SLHKLSTACP GRVPSMLSTS LNAEALQYLQ
961    GYLQAASVTL L
```

5.411 leucine zipper protein [Gallus gallus]

Protein Accession	gi|517089
Mean Expression Ratio	1.01
Median Expression Ratio	1.01
Credible Interval	(0.783, 1.31)
Associated Peptides	1
Associated Spectra	1
Coverage	0.0194

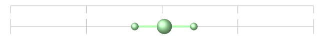

A	2.5	50	97.5	Sequence
**1**	**0.78**	**1.0**	**1.3**	**RVDDFKK**

```
1      MEDTTLQIIE PPTASEASEE QMPEEPIKSD QINGVMVLTL LDKIIGAVDQ IQLTQTQLEE RQQEMDSAVV SIQGELTKLT
81     KAHTTTSNTV NKMLEKVRKV SVNVKTVRQN LEKQAGQIKK LEANEAELLK RRNFKVMIYQ DEVKLPSKLS ISKSLKEGEK
161    QEKEGEGEEV PAGEDHAEED HIQLSSDEEV EIEEIIEESR AERIKRSGMK RVDDFKKAFS KEKMEKTKLK TKENLEKTRH
241    NLEKTRHNLE KRMNKLGTKI VTNERREKMK TSRDKLRKSF TPDHTIYARS KTAVYKVPPF TFHVKKIREG EVEVKATELV
321    EVGGEEGENS DLMRGESPEM HTLLEITEES DAVLVDKSDS E
```

5.412 glutamine-rich 1 [Gallus gallus]

Protein Accession	gi|61098266
Mean Expression Ratio	0.986
Median Expression Ratio	0.986
Credible Interval	(0.791, 1.23)
Associated Peptides	2
Associated Spectra	2
Coverage	0.0269

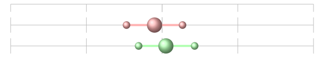

A	2.5	50	97.5	Sequence
**1**	**0.72**	**0.93**	**1.2**	**ATGALLYNAAAR**
**1**	**0.8**	**1.0**	**1.3**	**FHKPGENYK**

```
1      MAAMAATEEA EALDLFTGIG LTEAKARETL RNAALSAQLR QAVLQARAAL GSGLDKATGA LLYNAAARLR DPDRLAFVVG
81     YIGRREIRTD QQLGAALQYL RSHPLEPLEP ADFERACGVG VRVSPEQIEE AVEAVVSRHR EELLAERYHF NTGLLMAEAR
161    SRLQWADGKS IKNEVDLQVL HLLGPKTEAD LEKKPKAAKA RPAPVEKQKA AVVENGEVGA ETKSLLEQLR GEALKFHKPG
241    ENYKTEGYVV TPNTMALLKQ HLALTGGQVR TRFPPEPNGI LHIGHAKAIN FNFGYAKANG GICFLRYDDT NPEKEEEKYF
321    TAIREMVEWL GYQPYAVTHA SDYFDQLYTW ALELIRRGQA YVCHQKVEEI KGHNPPPSPW RDRPVEESLV LFEDMRKGKF
401    GEGEATLRMK LVMEDGKMDP VAYRVKFTPH HRTGDKWCIY PTYDYTHCLC DSIEHITHSL CTKEFQARRS SYFWLCNALD
481    VYCPVQWEYG RLNLLYTVVS KRKIIRLVET GAVRDWDDPR LFTLTALRRR GFPPEAINNF CARVGVTVAQ ATMEPHLLEA
561    CVRDVLNEQA PRAMAVLEPL KVTITNFPAP QAIDVLVPNF PADESRGFHK VPFQSVVYIV ESDFKEEADR GYKRLALGQP
641    VGLRHTGYVI AVQNVIKDAR GRVIELEVTC TKSDAAEKPK AFIHWVSEPR VCEVRLYERL FLHKNPEDPS EVPGGFLSDL
721    NPDSLRVVDD ALVDSSVLGA RPFDKFQFER LGYFSVDPDS TDSKLVFNRT VTLKEDPGKA
```

5.413 protein disulfide isomerase family A, member 4 [Gallus gallus]

Protein Accession	gi|57530768
Mean Expression Ratio	0.987
Median Expression Ratio	0.986
Credible Interval	(0.819, 1.19)
Associated Peptides	3
Associated Spectra	4
Coverage	0.067

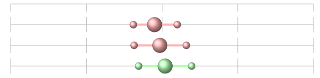

A	2.5	50	97.5	Sequence
**2**	**0.77**	**0.94**	**1.1**	**YAMEPEEFDSDALR**
**1**	**0.77**	**0.98**	**1.3**	**GKPYDYSGPR**
**1**	**0.8**	**1.0**	**1.3**	**NNPIKFEGGDRDLEHLSK**

```
1      MAQLAVLVAQ EDGRESVTQE VDGDDDEEEE DDDDDDNSEV KEENDVLVLN DANFDTFTAD KDTVLLEFYA PWCGHCKQFA
81     PEYEKIAKTL KENDPPIPVA KIDATAATAL ASRFDVSGYP TIKILKKGQP VDYDGSRTED AIVAKVKEIS DPNWTPPPEA
161    TLVLTQDNFD DVVKDADIIL VEFYAPWCGH CKRLAPEYEK AAQELSKRTP PIPLAKVDAT AETELAKKFD VTGYPTLKIF
241    RKGKPYDYSG PREKYGIVDY MIEQAGPPSK QIQATKQVQE FLKDGDDVII IGVFSGETDE VYQLYQEAAN SLREDYKFHH
321    TFSSEIAKLL KVSPGKLVVM QPEKFQSKHE PKMYVLDLKY STSESEIKEH VVKHALPLVG HRKPSNDAKR YAKRPLVVVY
401    YTVDFSFDYR VATQYWRGKV LEVAKDFPEY VFAVSDEEDY SSEIKDLGLL ESGEDVNVAI LDEGGKKYAM EPEEFDSDAL
481    RQFVLAFKKG KLKPIVKSQP VPKNNKGPVK VVVGKTFDTI VMDPKNDVLI EFYAPWCGHC KKLEPVYTEL GKKYKNEKNL
561    VIAKMDATAN DVTNDHYKVE GFPTIYFAPR DKKNNPIKFE GGDRDLEHLS KFIEEHATKL SRTKEEL
```

5.414 PREDICTED: similar to RP11-575L7.5 isoform 1 [Gallus gallus]

Protein Accession	gi|118104129
Mean Expression Ratio	1.01
Median Expression Ratio	1.01
Credible Interval	(0.785, 1.31)
Associated Peptides	1
Associated Spectra	1
Coverage	0.0293

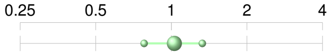

A	2.5	50	97.5	Sequence
1	0.78	1.0	1.3	DVSVDEEGAR

```
1      MEALPSPLES ARFIAGRSRD VSVDEEGARK VAESLFDKAS EAAFGLSGWK SLHELNPRAA SEEAVSWVFL VDTLNFSFWS
81     ESAEQNAWCA TRGRAYSGYW ALCAAVNRAL DDGIPITSAS YYATMTLDQV RQVFRSDTEV PMPLLEERHR VLNESGTVLL
161    EKFGGSFLTC VKMSENSAQK LLRLVVENFP SYRDEAVFEK KKVSFYKRAQ ILVADTWSVL EGKGDGFFGD ISSLTIFADY
241    RIPQVLVHLK AMKYSEDLMK KLREGTVFKS GDREEVEIRG CSIWCCTLIC KHLLDLYEKK GQDMRDQINA VLLDYYLWDY
321    ARDHREDMKD IPFHRVRCIY Y
```

5.415 aconitase 1, soluble [Gallus gallus]

Protein Accession	gi|72535134
Mean Expression Ratio	0.986
Median Expression Ratio	0.987
Credible Interval	(0.78, 1.25)
Associated Peptides	1
Associated Spectra	2
Coverage	0.0112

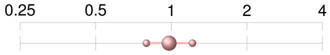

A	2.5	50	97.5	Sequence
2	0.8	0.98	1.2	NVEVPFKPAR

```
1      MSNPFVQIVE PLDAKEPVKK FFNLSKLEDV RYARLPFSIR VLLEAAIRNC DEFLVKKQDV ENILNWKVMQ HKNVEVPFKP
81     ARVILQDFTG VPAVVDFAAM RDAVKKLGGD PEKINPICPA DLVIDHSIQV DFNRRSDSLQ KNQDLEFERN KERFEFLKWG
161    SQAFKNMRII PPGSGIIHQV NLEYLARVVM DQDGYYYPDS VVGTDSHTTM VDGLGVLGWG VGGIEAEAVM LGQPISMVLP
241    EVVGYKLLGN PQPLVTSTDI VLTITKHLRQ VGVVGKFVEF FGPGVAQLSI ADRATIANMC PEYGATAAYF PVDDISIGYL
321    VQTGRDKEKV LCTKKYLEAV GMLRDFKNSS QDPDFTQVVE LDLHTVVPCC SGPKRPQDKV AVSDMKKDFE TCLGAKQGFK
401    GFQIAPDRHN SVIKFNFEGC DFELAHGSVV IAAITSCTNT SNPSVMLGAG LLAKKAVEAG LTVKPYIKTS LSPGSGVVTY
481    YLRESGVMSY LSQLGFDVVG YGCMTCIGNS GPLPDSVVEA ITQGDLVAVG VLSGNRNFEG RVHPNTRANY LASPPLVIAY
561    AIAGTVRIDF EKEPLGISAS GKKIFLKDIW PTRNEIQAVE RQYVIPGMFK EVYQKIETVN EAWNALDAPS DKLYTWNPKS
641    TYIKSPPFFD GLTLALQTPK TIEDAYVLLN FGDSVTTDHI SPAGNIARNS PAARYLTSRG LTPREFNSYG SRRGNDAVMA
721    RGTFANIRLV NKFIDKQGPQ TIHFPSGETL DVFDAAERYK QAGHPLIVLA GKEYGAGSSR DWAAKGPFLL GVKAVLAESY
801    ERIHRSNLVG MGVIPLQYLP GEDARTLGLT GRERYTIIIP ENLKPQMNIQ IKLDTGKTFH AIMRFDTDVE LTYFHNGGIL
881    NYMIRKMAS
```

5.416 hypothetical protein [Gallus gallus]

Protein Accession	gi|53130330
Mean Expression Ratio	0.986
Median Expression Ratio	0.987
Credible Interval	(0.766, 1.27)
Associated Peptides	1
Associated Spectra	1
Coverage	0.0528

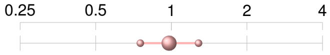

A	2.5	50	97.5	Sequence
1	0.75	0.98	1.3	VVVHPLVLLSVVDHFNR

```
1      MPELAVDRVV VHPLVLLSVV DHFNRIGKVG NQKRVVGVLL GSWQKKILDV SNSFAVPFDE DDKDDTVWFL DHDYLENMYG
81     MFKKVNARER IVGWYHTGPK LHKNDIAINE LMKRYCPNSV LVIIDVKPKD LGLPTEAYIS VEEVHDDGTP TSKTFEHVTS
161    EIGAEEAEEV GVEHLLRDIK DTTVGTLSQR ITNQVHGLKG LNSKLLDIRS YLEKVAMGKL PINHQIIYHL QDVFNLLPDV
241    NLQEFVKAFY LKTNDQMVVV YLASLIRSVV ALHNLINNKI ANRDAEKKEG QEKEESKKER KDEKEKDKEK SDVKKEEKKK
321    KK
```

5.417 PREDICTED: hypothetical protein [Gallus gallus]

Protein Accession	gi|118083310
Mean Expression Ratio	0.987
Median Expression Ratio	0.987
Credible Interval	(0.847, 1.15)
Associated Peptides	6
Associated Spectra	7
Coverage	0.101

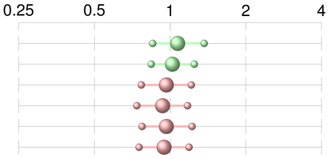

A	2.5	50	97.5	Sequence
1	0.85	1.1	1.4	EEDANSSAGDPPR
2	0.84	1.0	1.2	IFQSAPSDPTQDFSTQVAK
1	0.77	0.97	1.2	IASAVEAILTADSASR
1	0.74	0.94	1.2	IVIFPEHLDIPR
1	0.77	0.97	1.2	YFDGSGGNNHAVEHYR
1	0.75	0.95	1.2	KYVDKLEK

```
1     MAELSEALLS VLPSIRVPKA GDRVHKDECA FSFDTPESDG GLYICMNTFL GFGKQYVEKH YQKTGQRVYL HLKRTRKPKE
81    EDANSSAGDP PRKKPTRLAI GVEGGFDITE EKFEYDEDVK IVIFPEHLDI PRDGLEGLPD MVRDRIASAV EAILTADSAS
161   RKQEVQAWDG EVRRVSKHAF SLHQLQNDVR IPPCGWKCSK CDMRENLWLN MTDGAILCGR RYFDGSGGNN HAVEHYRETG
241   YPLAVKLGTI TPDGADVYSY DEDDMVLDPN LAEHLAHFGI DMLKMQKTDK TMTELEIDMN QRIGEWELIQ ESGVQLKPLY
321   GPGYTGIRNL GNSCYLNSVM QVLFSIPDFQ RKYVDKLEKI FQSAPSDPTQ DFSTQVAKLG HGLLSGEYSK PASAEGEQQP
401   DQKGVQNGIA PRMFKALIGK GHPEFSTNRQ QDAQEFFLHF INMVERNCRS SENPNEVFRF LVEEKLKCLA TEKVKYTQRV
481   DYIMQLPVPM DAALNKDELL EYEEKKRQAE EEKQPLPELV RAKVPFSSCL EAYGAPEQVD DFWSTALQAK SVALKTTRFA
561   SFPDYLVIQI KKFTFGLDWV PKKLDVSIEM PEELDISALQ GTGLQDGEEE MPDIAPPLVT PDEPKAPMLD ESVIIQLVEM
641   GFPMDACRKA VYYTGNSGVE AAMNWVMSHM DDPDFANPLV LPGSSGPGST IACPDPPSED SVATIVSMGF SRDQAMKALR
721   ATNNSLERAV DWIFSHIDDL DAEAAMDISE GRSAAESISE SVPVGPKVRN GPGKYQLFAF ISHMGTSTMC GHYVCHIKKD
801   GRWVIYNDQK VCASEKPPKD LGYIYFYQRI PS
```

5.418 proteasome (prosome, macropain) subunit, beta type, 1 [Gallus gallus]

Protein Accession	gi|56118302
Mean Expression Ratio	0.986
Median Expression Ratio	0.987
Credible Interval	(0.762, 1.28)
Associated Peptides	1
Associated Spectra	1
Coverage	0.0591

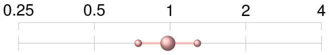

A	2.5	50	97.5	Sequence
1	0.75	0.98	1.3	GAVYSFDPVGSYQR

```
1     MLSAAGYAEL RPEPGCGVGA PVQYRFSPYT FNGGTVLAIA GEDFCIVASD TRLSEGYAIH SRDSPKCYRL TEQTVIGCSG
81    FHGDCLTLTK IIEARLKMYK HSNNKTMTTG AIAAMLSTIL YSRRFFPYYV YNIIGGLDEE GKGAVYSFDP VGSYQRDSFK
161   AGGSASAMLQ PLLDNQIGFK NMQNVEHVPL TLEKALQLVK DVFISAAERD VYTGDALKIC VVTKDGIKEE TIQLRKD
```

5.419 histone H3 [Euperipatoides leuckartii]

Protein Accession	gi|4883735
Mean Expression Ratio	0.987
Median Expression Ratio	0.987
Credible Interval	(0.765, 1.28)
Associated Peptides	1
Associated Spectra	1
Coverage	0.0917

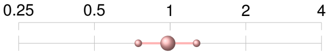

A	2.5	50	97.5	Sequence
1	0.75	0.98	1.3	KSAPATGGVK

```
1     RKSTGGKAPR KQLATKAARK SAPATGGVKK PHRYRPGTVA LREIRRYQKS TELLIRKLPF QRLVREIAQD FKTDLRFQSS
81    AVMALQEASE AYLVGLFEDT NLCAIHAKR
```

5.420 Heat shock protein HSP 90-alpha

Protein Accession	gi|123668
Mean Expression Ratio	0.987
Median Expression Ratio	0.987
Credible Interval	(0.903, 1.08)
Associated Peptides	17
Associated Spectra	36
Coverage	0.228

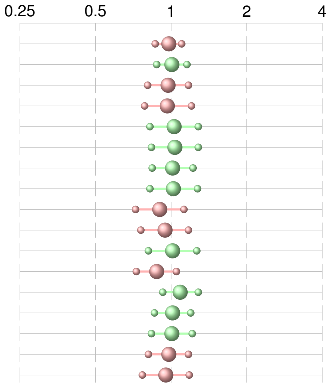

A	2.5	50	97.5	Sequence
7	0.87	0.98	1.1	GVVDSEDLPLNISR
5	0.88	1	1.2	NPDDITNEEYGEFYK
2	0.81	0.97	1.2	YYTSASGDEMVSLK
1	0.78	0.97	1.2	HVYYITGETK
1	0.83	1.0	1.3	EGLELPEDEEEKK
1	0.83	1.0	1.3	HSQFIGYPIR
2	0.84	1.0	1.2	HLEINPDHSIIETLR
1	0.82	1.0	1.3	RAPFDLFENR
1	0.72	0.9	1.1	CLELFTELAEDKENYK
1	0.76	0.94	1.2	EIVKK
1	0.81	1.0	1.3	VILHLKEDQTEYLEER
2	0.73	0.87	1.1	CLELFTELAEDKENYKK
3	0.92	1.1	1.3	QKAEADKNDK
3	0.86	1.0	1.2	INLIPNKHDR
2	0.84	1	1.2	KHSQFIGYPIR
2	0.81	0.98	1.2	KHLEINPDHSIIETLR
1	0.77	0.95	1.2	KNLVKK

```
1      MPEAVQTQDQ PMEEEVETFA FQAEIAQLMS LIINTFYSNK EIFLRELISN SSDALDKIRY ESLTDPSKLD SGKDLKINLI
81     PNKHDRTLTI VDTGIGMTKA DLVNNLGTIA KSGTKAFMEA LQAGADISMI GQFGVGSYSA YLVAEKVTVI TKHNDDEQYA
161    WESSAGGSFT VRLDNGEPLG RGTKVILHLK EDQTEYLEER RIKEIVKKHS QFIGYPIRLF VEKERDKEVS DDEAEEKEEE
241    KEEKEEKTED KPEIEDVGSD EEEEKKDGDK KKKKKIKEKY IDEEELNKTK PIWTRNPDDI TNEEYGEFYK SLTNDWEDHL
321    AVKHFSVEGQ LEFRALLFVP RRAPFDLFEN RKKKNNIKLY VRRVFIMDNC EELIPEYLNF MRGVVDSEDL PLNISREMLQ
401    QSKILKVIRK NLVKKCLELF TELAEDKENY KKFYEQFSKN IKLGIHEDSQ NRKKLSELLR YYTSASGDEM VSLKDYCTRM
481    KENQKHVYYI TGETKDQVAN SAFVERLRKH GLEVIYMIEP IDEYCVQQLK EFEGKTLVSV TKEGLELPED EEEKKKQEEK
561    KAKFENLCKI MKDILEKKVE KVVVSNRLVT SPCCIVTSTY GWTANMERIM KAQALRDNST MGYMAAKKHL EINPDHSIIE
641    TLRQKAEADK NDKSVKDLVI LLYETALLSS GFSLEDPQTH ANRIYRMIKL GLGIDEDDTA AEEASPAVTE EMPPLEGDDD
721    TSRMEEVD
```

5.421 oxysterol binding protein-like 2 [Gallus gallus]

Protein Accession	gi|56605888
Mean Expression Ratio	0.988
Median Expression Ratio	0.987
Credible Interval	(0.766, 1.28)
Associated Peptides	1
Associated Spectra	1
Coverage	0.0187

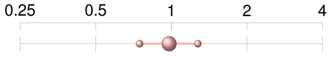

A	2.5	50	97.5	Sequence
1	0.75	0.98	1.3	GTITLELLK

```
1      MNSEEEFYDA VTGFDSDNSS GEFSEANHKV AEMLDLDPHQ SSRTGKHNGE TEIQENGIKR HRTSLPAPMF SRSDFSVWSI
81     LKKCIGLELS KITMPIVFNE PLSFLQRITE YMEHIYLINK ACSHADPLER MQAVAAFAVS AVASQWERTG KPFNPLLGET
161    YELTREDLGF RFISEQVSHH PPISAFYSEG LNKDFIFHGS IYPKLKFWGK SIEAEPRGTI TLELLKHNEA YTWTNPTCCV
241    HNVIIGKLWL EQYGTVEIVN HSTGDKCVLN FKPCGLFGKE LHRVEGYIQD KNRKKLCVIY GKWTECLWCT DPTTYETYKK
321    NEKRGGEQKK LKPSEDVIKS ENDEADDMPE VQDTVQFIPG SKLLWRINCR PPNSSQMYNF TSFAVSLNEL EKGMEAILAP
401    TDCRLRPDIR NMENGNMDVA SKEKERLEEK QRAARKERAK DEVEWQTRWF HQGTNPYTGT SDWLYSGGYF DRNFSDCPDI
481    Y
```

5.422 PREDICTED: similar to putative protein kinase [Gallus gallus]

Protein Accession	gi|118083044
Mean Expression Ratio	0.988
Median Expression Ratio	0.987
Credible Interval	(0.765, 1.27)
Associated Peptides	1
Associated Spectra	1
Coverage	0.00504

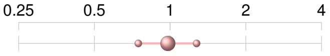

A	2.5	50	97.5	Sequence
**1**	**0.75**	**0.98**	**1.3**	**FQVTTTTDQVGR**

```
1       MSGGAADGAP PAPRFLAPPP PPPKNGSSSD SSVGEKLGAA EHAASGTGGA SGGAGSGGRS EEYRRRRHTM DKDSRGAAAT
81      EHRFFRRSVI CDSNATALEL PSLQPAAPSA PVSGGSAAPL VSPPECVRTS CIAVGASQAP SLLQQPPPPT PLPLEGRSAQ
161     EPPATKDAAP LLPKEEEDEA AALPPTSAAG STVAASREFE ERRAQQEDIE ELETKAVGIS PDGRFLKFDI EIGRGSFKTV
241     YKGLDTDTTV EVAWCELQDR KLSKSERQRF KEEAGMLKGL QHPNIVRFYD SWESTVKGKK CIVLVTELMT SGTLKTYLKR
321     FKVMKIKVLR SWCRQILKGL QFLHTRTPPI IHRDLKCDNI FITGPTGSVK IGDLGLATLK RASFAKSVIG TPEFMAPEMY
401     EEKYDESVDV YAFGMCMLEM ATSEYPYSEC QNAAQIYRRV TSGVKPASFD KVAIPEVKEI IEGCIRQNKG ERYAIKDLLN
481     HAFFQEETGV RVELAEEDDG EKIAIKLWLR IEDIKKLKGK YKDNEAIEFS FDLERDVPED VAQEMVESGY VCEGDHKTMA
561     KAIKDRVSLI KRKREQRQLV REEQEKKLQE EGSQKQQLEQ QQPSSASHAG SKHPVSVSGT TPVPTTSASV STQVEPEEPE
641     ADQHQQLQFQ QPSISILSDG AVDSGQGSSV YTESCVSSQQ TVSYGSQHDQ PISTAAVQGY AASVGQVQSQ QHGGYQPPAV
721     TQVQGQSASS STSVPSQPTQ HTQQNAQQPA SSQQPGQYQL QQPSVSTGAT PTQTVSQTQT SQVMPMPQAA AGTQLPVSQP
801     VSIIQGEPQL PVAAPSLPQP SVAPSVPIGS HFLPVGQPLP TSIVPQFSVS QLPVAAPHVS VAQPGFQSLP ISMAGSMNQP
881     LLTLATSAAA TAVPVGSTVV PSQLPTLMQP VAQLPSQVLP QLLQPAVQSM GLPVSIGQAA DASLPAGDAL YQGFPSRLPP
961     QYPGDSSVAP SSAVASVSIP SAILSPPLPT DVMTQPGYIA PVVQPYVEQS VLVPMGNVGG QVQVPQPTVS LTQQASSASS
1041    QQAVVEGTQG ASQTAPSESL PSTQPAQSTP LASSMDSAHS DVASGMSDGN ENVPASSGRH EGRTTKRHMR RSVRSRSRHE
1121    KTARPKLRIL NVSNKGDRVV ECQLETHNRK MVTFKFDLDG DNPEEIASIM VQNEFILATE RDSFVEQVRE IIEKADEMLS
1201    EDVSVEPEGD QGLESMQTKD DGFIPGSQKL EFKQPDPTSS MPQRIGVPPS SFTQVVHSAG RRFIVSPVPE SRLKEQGFFT
1281    SPIPGGKETS DVVAASPLHG PGMNLSHSAS SLSLQQAFSE IGHGQMTEGP STAPPVFNQT IPPFPPALST MASSGAPPAS
1361    VAAPSISIPS STGVSLPSSV TLSSENAAVG VAPSASVPSS VSPPLASQSG QQLSGGVASS VSAPASFSLT ITSQPAQPVT
1441    GDIAPSISTP ASLALPATQV PGVTGLGVVA PAVTSQSTPQ IVSSLAAPQT SVALSQAQNV ALQLPQLTSS GSVSSLAETT
1521    VVSAPQSLPE SGQSADKSQP SSAAGLSLPI SAPLSSSVAT SLCGSVTQPV IHPLLIPSAI TSTPVLSQIP GATPVLPQVP
1601    LPGVLPQPVT NLPAVQQTLI HSQPQPAPLP NQPHIHCLEA DADAQSKAPG IDDIKTLEEK LRSLFSEHGN VGAAHPSVSL
1681    ETSLIMETTV IPGIPTTVVA PTKPLTSVST CIPPSSLPLG PAGLPVLTPV ATPGQVITPV SYISAPSSVA TAVVKPGTSP
1761    SKPPLSRVPV LPVGSELPAG TPSSEPLPPF PGPSLTQSQQ PLEDLDAKLR RTLSPETVPV TSAPASSTAV TGLVSTAAQS
1841    LKDASCGGES SGTASTTGAG VLKMGRFQVS VAVDDVLKES DKAETKPLQF ETTSSDSSPL SGSSPESTLV KQAGGRKSEA
1921    VAHGSPQPVP VLQLPVDDGQ PTKVGRFQVT TTTDQVGRFS VSKTQDEVTC AEREPMTLPL SVDLEQVASS AAAPRKELES
2001    RQSPHMNGPS SEPEAAFLSG MAKDLDDGSA SPDSLQATAS KISLPVQSLS NSFNSSYMSS DNESDIEDED LKLELRRLRE
2081    KHLKEIQELQ SRQKQEIESL YTKLGKVPPA VIIPPAAPLS GRRRRPTKGK SSKSSRSSSQ GNKSPQLSGN LSAQSAPSVL
2161    PPQQTLHPPG SVPETGQNHL LQPLKPSPSS ENLYSAFTSD GALSVPSLSA PGQGTSSTNA VGATVNSQAP QSQPTAIASS
2241    RKGTFTDDLH KLVDNWARDA MNLSGKKVGK GHSNYEGPGM ARKFSAPGQL CISMTSSLGA TPISAASATS LGPFTKAMCP
2321    PQQYGYPAAS FAAPWSGTSG PAQQPLSQFQ PVGATSLQSF NISGLQKSIS NPPGSNLRTT
```

5.423 fibulin 1 [Gallus gallus]

Protein Accession	gi|45383790
Mean Expression Ratio	0.987
Median Expression Ratio	0.987
Credible Interval	(0.761, 1.27)
Associated Peptides	1
Associated Spectra	1
Coverage	0.0327

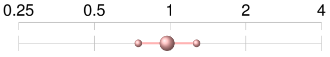

A	2.5	50	97.5	Sequence
**1**	**0.75**	**0.97**	**1.3**	**AITPTYPANQADIIFDITEGNLR**

```
1       MDKLRGARPL RLLLLLLALL PALRGQDLSM EECCDKGVEW ANKNRICTSL PLISESRECS MTQVQCCRSK LEEHYCSDGI
81      EFASVHEECD SHNGENSTCE AEYFKKCCYC CLLGKTAQVQ GQSCEPNLKI GYQCGIVFRA CCVKGQEGTD VSISDDAPKK
161     EQVEISKEEL DQEDPYLHDG CRGGGPCSQQ CRDTGSSYVC SCFVGYQLQP DGVNCEDINE CITGTHSCGI GQTCVNTLGS
241     FRCQRDTSCG TGYELTDDSR CKDIDECETG THNCPPDFIC QNTPGSFRCR PKLQCMNGFI QDALGNCIDI NECLSTNMPC
321     PAGQICINTD GSYTCQRISP SCGRGYHLNE DGTRCVDVDE CSSSDQPCGE GHVCINGPGN YRCECKSGYS FDVISRTCID
401     INECRRYPGR LCAHKCENTP GSYYCTCTMG FKLSSDGRSC EDLNECESSP CSQECANVYG SYQCYCRRGF QLSDIDGISC
481     EDIDECALPT GGHICSFRCI NIPGSFQCTC PSTGYRLAPN ARNCQDIDEC VAETHNCSFN ETCFNIQGGF RCLSLECPEN
561     YRKSGDTVRL EKTDTIRCIK SCRPNDVNCV LDPVHTISHT VISLPTFREF TRPEEIIFLR AITPTYPANQ ADIIFDITEG
641     NLRESFDIIK RYMDGMTVGV VRQVRPIVGP FHAILKLEMN YVMGGVVSHR NIVNVHIFVS EYWF
```

5.424 ras-related GTP-binding protein RAB10 [Gallus gallus]

Protein Accession	gi|71895051
Mean Expression Ratio	1.01
Median Expression Ratio	1.01
Credible Interval	(0.788, 1.31)
Associated Peptides	1
Associated Spectra	1
Coverage	0.055

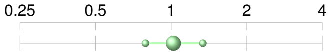

A	2.5	50	97.5	Sequence
1	0.79	1.0	1.3	AFLTLAEDILR

```
1       MAKKTYDLLF KLLLIGDSGV GKTCVLFRFS DDAFNTTFIS TIGIDLKIKT VELQGKKIKL QIWDTAGQER FHTITTSYYR
81      GAMGIMLVYD ITNAKSFENI SKWLRNIDEH ANEDVERMLL GNKCDMEDKR VVPKAKGEQI AREHGIRFFE TSAKANINIE
161     KAFLTLAEDI LRKTPVKEPN SENVDISSGG GVTGWKSKCC
```

5.425 heat shock 27kDa protein 1 [Gallus gallus]

Protein Accession	gi|45384222
Mean Expression Ratio	0.989
Median Expression Ratio	0.989
Credible Interval	(0.878, 1.11)
Associated Peptides	10
Associated Spectra	21
Coverage	0.466

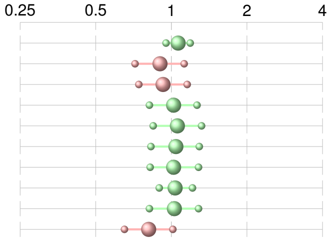

A	2.5	50	97.5	Sequence
9	0.95	1.1	1.2	LLPSESALLPAPGSPYGR
1	0.72	0.9	1.1	YTLPPGVEATAVR
1	0.74	0.93	1.2	VPFTFLTSPSWEPFR
1	0.82	1.0	1.3	WPSGSAWPGYFR
1	0.85	1.1	1.3	TKDNIVEITGK
1	0.83	1.0	1.3	KYTLPPGVEATAVR
1	0.82	1.0	1.3	KEEPAK
4	0.9	1.0	1.2	KEEPAKK
1	0.82	1.0	1.3	HEEKQDEHGFISR
1	0.65	0.81	1.0	DNIVEITGKHEEKQDEHGFISR

```
1       MAERRVPFTF LTSPSWEPFR DWYHGSRLFD QSFGMPHIPE DWYKWPSGSA WPGYFRLLPS ESALLPAPGS PYGRALSELS
81      SGISEIRQSA DSWKVTLDVN HFAPEELVVK TKDNIVEITG KHEEKQDEHG FISRCFTRKY TLPPGVEATA VRSSLSPDGM
161     LTVEAPLPKP AIQSSEITIP VTVEAKKEEP AKK
```

5.426 cytidylate kinase [Gallus gallus]

Protein Accession	gi|71896025
Mean Expression Ratio	1.01
Median Expression Ratio	1.01
Credible Interval	(0.79, 1.30)
Associated Peptides	1
Associated Spectra	1
Coverage	0.0408

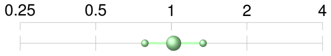

A	2.5	50	97.5	Sequence
1	0.78	1.0	1.3	FLIDGFPR

```
1       MKPVVVFVLG GPGAGKGTQC ARIVEKYGYT HLSAGDLLRD ERKRPGSQYG ELIENYIKEG EIVPVEITIS LLKRAMDQTM
81      AANSQKNKFL IDGFPRNEDN LQGWNKTMDG KADVSFVLFF DCDNEICIGR CLERGKSSGR SDDNRESLEK RIHTYLQSTR
161     PIIDLYERMG KVRRVDASKS VDEVFEKVVQ IFDKEG
```

5.427 PREDICTED: similar to Oxysterol-binding protein 1, partial [Gallus gallus]

Protein Accession	gi|118126565
Mean Expression Ratio	0.99
Median Expression Ratio	0.989
Credible Interval	(0.768, 1.28)
Associated Peptides	1
Associated Spectra	1
Coverage	0.069

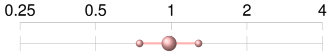

A	2.5	50	97.5	Sequence
**1**	**0.75**	**0.98**	**1.3**	**KKDPVTQELAHVYR**

```
1       TGDKCNLKFV PYSYFSRDVA RKVTGEVMDP AGKVHFLLLG TWDEKMDCYK VAAGSGDNGA EGRQRPHEAE ESRVPLWKRN
81      QLPKYAENMY YFSELALTLN APESGTAPTD SRRRPDQRLM ENGRWDEANA EKQRLEEKQR LARKRREAEA ARATEDGTPY
161     DPYKPLWFER KKDPVTQELA HVYRGGYWES KEKQDWSGCP DIF
```

5.428 PREDICTED: hypothetical protein [Gallus gallus]

Protein Accession	gi|118090272
Mean Expression Ratio	1.01
Median Expression Ratio	1.01
Credible Interval	(0.786, 1.31)
Associated Peptides	1
Associated Spectra	1
Coverage	0.0463

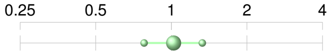

A	2.5	50	97.5	Sequence
**1**	**0.78**	**1.0**	**1.3**	**GLPFDSTEDDIADFFAGLR**

```
1       MAAAVARRGV AALLLGRRPW PLPPCLRPPC ARRRLLSAAA SLGPGGPQAA APLFTAARRY SQVIDSPTQE GQLAEQDPEP
81      PKEENEDSVF LIRAHGFPFA CTKKEMMAFF DSCKIRNGEN GIHFLLNRDG RRRGDALIEL ESKADVQKAL EKNLRYMGTR
161     YVKVHEIHDK DVDGLLQSLR YESEVMSDGV VLLRGLPFDS TEDDIADFFA GLRITDMTFV YRGERKTGEA YVQFAAPEMV
241     AKALLRHKEY MENRYIEVYI STKREMQRHL SLRKEMIRLR RELGSTAEER ELDYTRGSSA EREKEVASEA AESSGLSSQS
321     GSILSSLRTV HVRGFPPQVS AQDIVDFFAP LKPTRILIEY NSDGVATGEA DVHFESYDDA VAAMAKERAQ LQFGTVEVFL
401     KERPKAAGHR
```

5.429 PREDICTED: similar to actin-related protein 3-beta [Gallus gallus]

Protein Accession	gi|50732241
Mean Expression Ratio	1.01
Median Expression Ratio	1.01
Credible Interval	(0.783, 1.31)
Associated Peptides	1
Associated Spectra	1
Coverage	0.0263

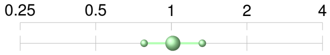

A	2.5	50	97.5	Sequence
**1**	**0.78**	**1.0**	**1.3**	**DITYFIQQLLR**

```
1       MASYLPPCVI DGGTGYTKLG YAGNTEPQFI IPSCIAIRES AKVGDQAQRR VMKGVDDLDF FIGDEAIDKP TYATKWPIRH
81      GIVEDWDLME RFMEQVIFKY LRAEPEDHYF LMTEPPLNTP ENREYLAEIM FESFNIPGLY IAVQAVLALA ASWTSRQVGE
161     RTLTGIVIDS GDGVTHVIPV AEGYVIGSCI KHIPIAGRDI TYFIQQLLRE REVGIPPEQS LETAKAIKEK YCYICPDIVK
241     EFAKYDGDPR KWIKQYTGIN AINKTKFVID VGYERFLGPE IFFHPEFANP DFMESISDVV DEVIQNCPID VRRPLYKNVV
321     LSGGSTMFRD FGRRLQRDLK RVVDARLRLS EELSGGRIKP KPVEVQVITH HMQRYAVWFG GSMLASTPEF FQVCHTKKDY
401     EEYGPSICRH NPVFGVMS
```

5.430 PREDICTED: similar to guanine nucleotide exchange factor Lbc [Gallus gallus]

Protein Accession	gi|118095912
Mean Expression Ratio	1.01
Median Expression Ratio	1.01
Credible Interval	(0.78, 1.31)
Associated Peptides	1
Associated Spectra	1
Coverage	0.0143

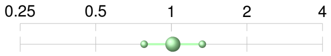

A	2.5	50	97.5	Sequence
**1**	**0.78**	**1.0**	**1.3**	**APGQQQVEDGNAEIPPTR**

```
1       MLIRVYRYRS LRFVSEPPER RPLLAFPTFG AEAFWGSEVV RRVIATGPGL IAWYGRVKQG DDIWHEFRPV HVLCTYGMSV
81      ALPVLSRGDF LLNAPGAAPM VLFDDEEGVW LQCPSLFYSV ADDDDERCYR DKAGDSVITV QLTEEDKVED DVVFYLIFTG
161     STVQHCTSTR KINPGSLETI SPGHDCCETV KVALCASKEG RPVLVVAEES FKFIQDEAYD AAQFLATYAG NQQALNFTRF
241     LDQSRPPAAD VDILDEKVAL AFRHLKLPSE WNVLGTDQSL NENIPRETLM HFAVRQGLLR LTWFLLQQPG GRGALSIHNR
321     DGATPVSLAL ERGYQKLHQL LTEEEAAEPD SWSTLSHTVH TGDYCVKHHQ QLDVYTLTAE TKERTKASLE SDIRQLRMHM
401     QSHQHMSSRK RTKTVLGASG NSCGNGAEPV GHSTASSQSL GEMPGEGRKE SPSDVLHLDI GQLSDQTHQE DECSSGSLIT
481     RVINAPNGSL GLEVVESPEC SCESKNTVTL CEKEEEGPAS AQSSGTLSDE NGCTVSPCAG INGAVNLPSC GNTNEEAGMT
561     SAGVVLDQAG LCSKDSTHQE VGAAEERAAE STVAAVETAG EAPASREGVD VAMGQTECRN CAGAADRAPG QQQVEDGNAE
641     IPPTRTADPE EPLPAEEEPT SAAQPLLDGF NGTECAGGED ANVGVVPVCD STNANVDTDV SVGLCKSGDN HEAGLSGTDL
721     VNNGVLESQG GACVKERQRV TASTRAPLPA VNGVKVPACA PMFPDVESQD SSESVEPEGQ AKVGSTNGKS SSPFLEDSLE
801     TDGLDAKAEN GNIGGTEQSS EKPTHCQGSS DIAECTGEAM EGSGAHNEEP VGTDTSSSRE GGSVDSADRA QEVASCCPPV
881     DQTTVNDEMG DKLKLDGTQG SVQEPRLLPL EDASTFSAGK EPPKAEERAE SASGQGMPHP DAGDLAVGRT GDAAVAPPGQ
961     EAPLCSNDPV LPPCTKGADC TEDNFVGTPF VIRNEESKQK QEVAAAGSAE LAAPHGQERG GVAGVLEDYA GAPGPPKQSL
1041    QPRAEGDTAK PEREASDSSQ EQVLSENLLA TVVKSFVGSA ERPAASSDVG AGLKELGPNQ AGTAGAGDSI MQGTGPAAGS
1121    NPCPDAGLPQ ECGPSLQHVS PRSSTPELKD AVEMEAPSLA FEAEEALQIA AITSITGYEE EQGEMESVLP RATLQPIAEE
1201    PTCDSDSSSH TSCLANIPED PKVKSRCKTN WQQRSQQIFS GHGGNLLQNC LLKCEKVNAS AHL
```

5.431 hypothetical protein [Gallus gallus]

Protein Accession	gi|53130600
Mean Expression Ratio	1.01
Median Expression Ratio	1.01
Credible Interval	(0.779, 1.31)
Associated Peptides	1
Associated Spectra	1
Coverage	0.0276

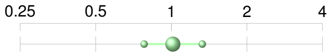

A	2.5	50	97.5	Sequence
**1**	**0.78**	**1.0**	**1.3**	**IQRPPEDSIQPYEK**

```
1       MSSFAKDFSK AMSQAGPSQF QEVIRQELEY SMKVELDKIL ATAPSNELEH TKKDLEGFKK LFHRFLQEKG PSVDWGKIQR
81      PPEDSIQPYE KIKARGLPDN IASVLNKLVV VKLNGGLGTS MGCKGPKSLI GVRNENTFLD LTVQQIEHLN KTYNTDVPLV
161     LMNSFNTDED TKKILQKYSL SRVKIYTFNQ SRYPRINKET LLPIAKDVSY SGENTECWYP PGHGDIYGSF YNSGLLDNLI
241     AEGKEYIFVS NIDNLGATVD LYILNHLMNP PNGKRCEFVM EVTNKTRADV KGGTLTQYEN KLRLVEIAQV PKAHVDEFKS
321     VSKFKIFNTN NLWIALSAIK RLQEKNAIDM EIIVNPKTLD GGLNVIQLET AVGAAIKSFE NSLGINVPRS RFLPVKTTSD
401     LLLVMSNLYS LNAGSLTMSE KREFPAVPLV KLGSSFTKVQ DYLRRFESIP DMLELDHLTV SGDVTFGKNV SLKGTVIIIA
481     NHGDRIDIPA GAVLENKIVS GNLRILDH
```

5.432 SH3 domain binding glutamic acid-rich protein like [Gallus gallus]

Protein Accession	gi|60302796
Mean Expression Ratio	1.01
Median Expression Ratio	1.01
Credible Interval	(0.807, 1.27)
Associated Peptides	1
Associated Spectra	3
Coverage	0.105

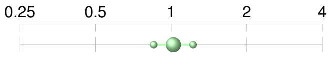

A	2.5	50	97.5	Sequence
**3**	**0.85**	**1.0**	**1.2**	**YLGDYEAFFEAR**

```
1       MVIKVYIASS SGSTAIKKQQ QDVLGFLEAN KIEFEEKDIA ANEENRKWMR ENVPEDRRPA SGNPLPPRLF NDSRYLGDYE
81      AFFEARENNA VYAFLGLTAP PGSKEAEALA KQQA
```

5.433 methionyl aminopeptidase 2 [Gallus gallus]

Protein Accession	gi|57525368
Mean Expression Ratio	1.01
Median Expression Ratio	1.01
Credible Interval	(0.779, 1.30)
Associated Peptides	1
Associated Spectra	1
Coverage	0.0271

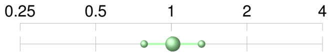

A	2.5	50	97.5	Sequence
1	0.78	1.0	1.3	GEECQYPPTQDGR

```
1       MAGVGQADGE AEKHLNGELP PEPEDRDGAA GPGAEDGAKK KRKKKKKGKV GPAGQETDKE MEGALDDVAK QLDKQALEEK
81      DKDDDDEDGE GEGDGAAGKK KKKKKKKGP  KVQTDPPSIP ICDLFPSNIY PKGEECQYPP TQDGRTAAWR ITSEEKKALD
161     QASEEIWNDF REAAEAHRQV RKYVMSWIKP GMTMIEICEK LEDCSRKLIK ENGLNAGLAF PTGCSLNNCA AHYTPNAGDP
241     TVLHYDDICK IDFGTYYSGR IIDCAFTVTF NPKYDRLLEA VKDATNTGIK CAGIDVRLCD VGEAIQEVME SYEVEIDGKT
321     YQVKPIRNLN GHSIGPYRIH AGKTVPIVKG GEATRMEEGE VYAIETFGST GKGVVHDDME CSHYMKNFDV GHVPIRLPRA
401     KHLLNVINEN FGTLAFCRRW LDRLGESKYL MALKNLCDLG IVDPYPPLCD IKGSYTAQFE HTILLRPTCK EVVSRGDDY
```

5.434 PREDICTED: similar to Peptidylprolyl isomerase domain and WD repeat containing 1 [Gallus gallus]

Protein Accession	gi|50761531
Mean Expression Ratio	1.01
Median Expression Ratio	1.01
Credible Interval	(0.784, 1.31)
Associated Peptides	1
Associated Spectra	1
Coverage	0.0272

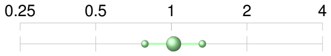

A	2.5	50	97.5	Sequence
1	0.78	1.0	1.3	ASGAAENEEEDEEDGER

```
1       MASSEPERKR RASGAAENEE EDEEDGERWV GPLPGEAAQT KKRRVLEFEH VYLENLPCAS MYERSYMHRD VITHVACTKT
81      DFIITASHDG HVKFWKKIEE GIEFVKHFRS HLGVIESIAV SSEGALFCSV GDDKAMKVFD VVNFDMINML KLGYSPGQCE
161     WVYCPGDAIS SVATSEKTTG KIFIYDGRGN NQPLHVFDKL HTSSLTQIRL NPVYKVVVSS DKSGMIEYWT GTPHEYKFPK
241     NVNWEYKTDT DLYEFAKCKA YPSSISFSPD GKKMATLGSD RKVRIFRFLT GKLMRVFDES LSMFTELQQM RQQLPDMEFG
321     RRMAVERELE KVDAVRLINI IFDETGHFVL YGTMLGIKVI NVETNRCIRI LGKQENIRVM QLALFQGVAK KHRAAITIEM
401     KASENPVLQN IQADPTVICT AFKKNRFYMF TKREPEDTKS ADSDRDVFNE KPSKEEVMAA TQAEGPKRVS DSAIIHTSMG
481     DIHIKLFPVE CPKTVENFCV HSRNGYYNGH IFHRIIKGFM IQTGDPTGTG MGGESIWGGE FEDEFHSTLR HDRPYTLSMA
561     NAGPNTNGSQ FFITVVPTPW LDNKHSVFGR VTKGMEVVQR ISNVKVNPKT DKPYEDISII NITVK
```

5.435 archain 1 [Gallus gallus]

Protein Accession	gi|118405190
Mean Expression Ratio	1.01
Median Expression Ratio	1.01
Credible Interval	(0.781, 1.31)
Associated Peptides	1
Associated Spectra	1
Coverage	0.0255

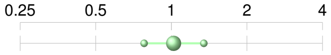

A	2.5	50	97.5	Sequence
1	0.78	1.0	1.3	GVQLQTHPNVDKK

```
1       MVLLAAAVCT KAGKAIVSRQ FVEMTRTRIE GLLAAFPKLM NTGKQHTFVE TESVRYVYQP MEKLYMVLIT TKNSNILEDL
81      ETLRLFSRVI PEYCRALEEN EISEHCFDLI FAFDEIVALG YRENVNLAQI RTFTEMDSHE EKVFRAVRET QEREAKAEMR
161     RKAKELQQAR RDAERLGKKA PGFGGFGSSA VSGGTTAAMI TETIIETEKP KVAPAPSRPS GPSKALKLGA KGKEVDNFVD
241     KLKSEGENIM TSVGKRSTEA AKVLAPPINM ESVHMKIEEK ISLTCGRDGG LQNMELHGMI MLHISDEKFA RIRLHVENED
321     KRGVQLQTHP NVDKKLFTAE SQIGLKNPEK SFPINSDVGV LKWRLQTTEE SFIPLTINCW PSESGNSCDV NIEYELQEES
401     LELNDVVIMI PLPSGVGAPV IGEIDGEYRH DSRRNLLEWC LPVIDAKNKS GSLEFSIAGQ PNDFFPVQVS FISKKNYCNI
481     QVTKVTQVDG NSPVRFSTET TFLVDKYEIL
```

5.436 PREDICTED: similar to P1725 [Gallus gallus]

Protein Accession	gi|118101506
Mean Expression Ratio	0.992
Median Expression Ratio	0.99
Credible Interval	(0.77, 1.28)
Associated Peptides	1
Associated Spectra	1
Coverage	0.140

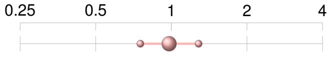

A	2.5	50	97.5	Sequence
**1**	**0.75**	**0.98**	**1.3**	**YELVDISQDNALR**

```
1       MSTLKVYSTS VTGSREIKSQ QSEVTRILDG KNIKYELVDI SQDNALREEM RAKAGNPKAI PPQIVNGDHY CGDYELFVEA
81      VEQNTLQEFL KLA
```

5.437 eukaryotic translation initiation factor 5B [Gallus gallus]

Protein Accession	gi|84105269
Mean Expression Ratio	1.01
Median Expression Ratio	1.01
Credible Interval	(0.779, 1.31)
Associated Peptides	1
Associated Spectra	1
Coverage	0.0156

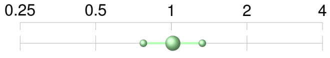

A	2.5	50	97.5	Sequence
**1**	**0.78**	**1.0**	**1.3**	**EGDTIIVPGVEGPIVTQIR**

```
1       MGKKQKNKSE DSAKDDIDID ALAAEIEGAG AAKEQEPQKS KGKKKKEKKK QDFDEDDILK ELEELSIEAQ GGKADREPST
81      GKVENDNEDS LSKQDKKRKG KSKKANLEND YDSEEMEDKD RKSKKTQKAK QDVLSGSDDD DLEIQPKKNK GKTQKSNKKH
161     ELSEDEANVK KSKERVGTLS TGESGDESDE FSQPRKGQKK NQKPKSTAAL GSGDEEEESS FKVKTVAQKK AEKKERERKK
241     REEEKAKLRK LKEKEELEGG KEAAKPKEAP KKAEEKASPD VAAAPGPGGK GEIPAGAEAD DNEGDKKKKD KKKKKGEKEE
321     KEKEKKKGPS KATVKAMQEA LAKMKEEEER AKREEEERIR RLEELEAKRK EEERLEQERK ERKKQKEKER KERLKKEGKL
401     LTKAQREARA RAEATLKLLQ AQGVEVPSKD SVPKKRPIYE DKKKKKQQQP ENKEVSESLE VTSPAEDAVE LETLVKEEPP
481     LPAEPEEKEE EEEDDGGLDD WEAMVSDEDE EKESKPVHIE VKEQNEVDEE EEEEEEDEEE EEEESEESDD AESEGSEDED
561     EKTSDERDAD SQATGKQSVE KKPSKEISSD SEYDSDDDRT KEERAYDKAK RRIEKRRAEN SKNANTEKLR APVICVLGHV
641     DTGKTKILDK LRHTHVQDSE AGGITQQIGA TNVPLEAINE QTKMVKNFDR ENIKIPGMLI IDTPGHESFS NLRNRGSSLC
721     DIAILVVDIM HGLEPQTIES INLLKSKKCP FIVALNKIDR LYDWKKSPDT DVAVTLKKQK KNTKDEFEER AKAIIVEFAK
801     QGLNAALFYE NKDPRTFVSL VPTSAHTGDG MGSLIALLVE LTQTMLTKRL AECQELRAQV MEVKALPGMG TTIDVILING
881     RLREGDTIIV PGVEGPIVTQ IRGLLLPPPM KELRVKNQYE KHKEVVAAQG VKILGKDLEK TLAGLPLLVA HKEDEVPVLK
961     DELIHELKQT LNAIKLEEKG VYVQASTLGS LEALLEFLKT SEVPYAGINI GPVHKKDVMK ASVMLEHDPQ YAVILAFDVR
1041    IERDAQEMAD SLGVRIFSAE IIYHLFDAFT KYRQDYKKQK QEEFKHIAVF PCKMKILPQF IFNSRDPIVM GVVVEAGQVK
1121    QGTPMCVPSK NFVDIGIVTS IEINHKPVEV AKKGQEVCVK IEPIPGESPK MYGRHFEATD ILVSKISRQS IDALKDWFRD
1201    EMQKSDWQLI VELKKVFEII
```

5.438 non-histone chromosomal protein [Gallus gallus]

Protein Accession	gi|45382755
Mean Expression Ratio	0.99
Median Expression Ratio	0.99
Credible Interval	(0.768, 1.28)
Associated Peptides	1
Associated Spectra	1
Coverage	0.0435

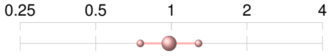

A	2.5	50	97.5	Sequence
1	0.75	0.98	1.3	GKGDPNKPR

```
1       MGKGDPNKPR GKMSSYAYFV QTCREEHKKK HPDSSVNFAE FSRKCSERWK TMSSKEKGKF EEMAKGDKAR YDREMKNYVP
81      PKGEKKGKKK DPNAPKRPPS AFFLFCSEHR PKIKNDHPGL SIGDTAKKLG EMWSEQLAKD KQPYEQKAAK LKEKYEKDIA
161     AYRAKSKSDA GKKGPGRPAG SKKKAEPEEE EEEEEDEEEE EEEEDEE
```

5.439 hsp 108 [Gallus gallus]

Protein Accession	gi|63509
Mean Expression Ratio	1.01
Median Expression Ratio	1.01
Credible Interval	(0.826, 1.23)
Associated Peptides	3
Associated Spectra	3
Coverage	0.0492

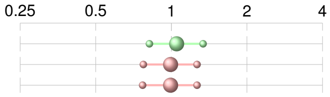

A	2.5	50	97.5	Sequence
1	0.82	1.0	1.3	GVVDSDDLPLNVSR
1	0.77	1	1.3	TVSDLAVVLFETATLR
1	0.77	1	1.3	FAFQAEVNR

```
1       MKSAWALALA CTLLLAASVT AEEVDVDATV EEDLGKSREG SRTDDEVVQR EEEAIQLDGL NASQIKEIRE KSEKFAFQAE
81      VNRMMKLIIN SLYKNKEIFL RELISNASDA LDKIRLISLT DENALAGNEE LTVKIKCDKE KNMLHVTDTG IGMTKEELIK
161     NLGTIAKSGT SEFLNKMTEM QDDSQSTSEL IGQFGVGFYS AFLVAERVIV TSKHNNDTQH IWESDSNEFS VIDDPRGNTL
241     GRGTTITLVL KEEASDYLEL DTVKNLVKKY SQFINFPIYV WSSKTETVEE PVEEEEAKEE KEETDDDEAA VEEEEEEKKP
321     KTKKVEKTVW DWELMNDIKP IWQRPSKEVE EDEYKAFYKT FSKEHDDPMA YIHFTAEGEV TFKSILFVPN SAPRGLFDEY
401     GSKKSDFIKL YVRRVFITDD FHDMMPKYLN FVKGVVDSDD LPLNVSRETL QQHKLLKVIR KKLVRKTLDM IKKIAEEKYN
481     DTFWKEFGTN VKLGVIEDHS NRTRLAKLLR FQSSHHESNL TSLDQYVERM KEKQDKIYFM AGASRKEAES SPFVERLLKK
561     GYEVIYLTEP VDEYCIQALP EFDGKRFQNV AKEGVKFEES EKSKESREAL EKEFEPLLNW MKDKALKDKI EKAVLSQRLT
641     QSPCALVASQ YGWSGNMERI MKAQAYQTVF SSYYASQKKT FEINPRHPLI KDMLRRVKEN EDDKTVSDLA VVLFETATLR
721     SGYMLPDTKE YGDRIERMLR LSLNIDLDAK VEEEPEEPED AAEEAEQDEE EVDADAEDSE TQKESTDVKD EL
```

5.440 GTP-binding protein PTD004 [Gallus gallus]

Protein Accession	gi|71895183
Mean Expression Ratio	0.99
Median Expression Ratio	0.99
Credible Interval	(0.765, 1.27)
Associated Peptides	1
Associated Spectra	1
Coverage	0.048

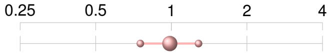

A	2.5	50	97.5	Sequence
1	0.75	0.98	1.3	SQAAAENFPFCTIDPNESR

```
1       MAPKKAGDGV KAHPIIGRFG TSLKIGIVGL PNVGKSTFFN VLTKSQAAAE NFPFCTIDPN ESRVPVPDDR FDFLCQYHKP
81      PSKIPAFLNV VDIAGLVKGA HTGQGLGNSF LSHINACDGI FHLMRAFEDD DITHVEGSVD PVRDIEIIHE ELRLKDEELI
161     TQSIDKLEKV AVRGGDKKLK PEYDVMCKIK TWVIDEKKAV RFYHDWNDKE IDVLNKHLFF TSKPMIYLVN LSEKDYIRKK
241     NKWLIKIKEW VDKHDPGALV IPFSGALELK LQDMSAEEKQ KYLEENMTQS ALPKIIKAGY AALQLEYFFT AGPDEVRAWT
321     IRKGTKAPQA AGKIHTDFEK GFIMAEVMKY EDFKEGGSEA AVKAAGKYRQ QGRNYIVEDG DIIFFKFNTP QQPKKK
```

5.441 alpha 1 type II procollagen [Gallus gallus]

Protein Accession	gi|31340542
Mean Expression Ratio	0.99
Median Expression Ratio	0.991
Credible Interval	(0.781, 1.26)
Associated Peptides	1
Associated Spectra	2
Coverage	0.0142

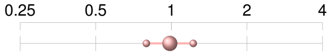

A	2.5	50	97.5	Sequence
2	0.8	0.98	1.2	GETGAQGPMGPSGPAGAR

```
1      GPQGFQGNPG EPGEPGAAGP MGPRGPPGPP GKPGDDGETG KPGKSGERGP PGPQGARGFP GTPGLPGVKG HRGYPGLDGA
81     KGEAGAPGAK GESGSPGENG SPGPMGPRGL PGERGRPGPS GAAGARGNDG LPGPAGPPGP VGPAGAPGFP GAPGSKGEAG
161    PTGARGPEGA QGPRGESGTP GSPGPAGAPG NPGTDGIPGA KGSAGAPGIA GAPGFPGPRG PPGPQGATGP LGPKGQTGEP
241    GIAGFKGEQG PKGETGPAGP QGAPGPAGEE GKRGARGEPG AAGPVGPPGE RGAPGNRGFP GQDGLAGPKG APGERGPAGL
321    AGPKGATGDP GRPGEPGLPG ARGLTGRPGD AGPQGKVGPT GAPGEDGRPG PPGPQGARGQ PGVMGFPGPK GANGEPGKAG
401    EKGLPGAPGL RGLPGKDGET GAAGPPGPAG PVGERGEQGA PGPSGFQGLP GPPGPPGESG KPGDQGVPGE AGAPGLVGPR
481    GERGFPGERG SPGAQGLQGP RGLPGTPGTD GPKGATGPAG PNGAQGPPGL QGMPGERGAA GIAGPKGDRG DVGEKGPEGA
561    PGKDGARGLT GPIGPPGPAG PNGEKGESGP PGPSGAAGAR GAPGERGEPG APGPAGFAGP PGADGQPGAK GEQGEPGQKG
641    DAGAPGPQGP SGAPGPQGPT GVTGPKGARG AQGPPGATGF PGAAGRVGPP GPNGNPGPPG PPGSAGKDGP KGVRGDAGPP
721    GRAGDPGLQG PAGPPGEKGE PGEDGPAGPD GPPGPQGLAG QRGIVGLPGQ RGERGFPGLP GPSGEPGKQG APGSAGDRGP
801    PGPVGPPGLT GPAGEPGREG NPGADGPPGR DGAAGVKGDR GETGPVGAPG APGAPGAPGP VGPTGKQGDR GETGAQGPMG
881    PSGPAGARGM PGPQGPRGDK GETGEAGERG LKGHRGFTGL QGLPGPPGPS GDQGAAGPAG PSGPRGPPGP VGPSGKDGSN
961    GMPGPIGPPG PRGRSGEPGP AGPPGNPGPP GPPGPPGTGI DMSAFAGLGQ TEKGPDPIRY MGADEAAGGL RQHDVEVDAT
1041   LKSLNNQIES IRSPEGSKKN PARTCRDIKL CHPEWKSGDY WIDPNQGCTL DAIKVFCNME TGETCVYPTP SSIPRKNWWT
1121   SKTKDKKHVW FAETINGGFH FSYGDENLSP NTASIQMTFL RLLSTEGSQN VTYHCKNSIA YMDEETGNLK KAILIQGSND
1201   VEIRAEGNSR FTYSVLEDGC TKHTGKWGKT VIEYRSQKTS RLPIVDIAPM DIGGADQEFG VDIGPVCFL
```

5.442 taste receptor, type 2, member 7 [Gallus gallus]

Protein Accession	gi|123959734
Mean Expression Ratio	1.01
Median Expression Ratio	1.01
Credible Interval	(0.782, 1.31)
Associated Peptides	1
Associated Spectra	1
Coverage	0.0280

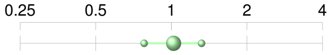

A	2.5	50	97.5	Sequence
1	0.78	1.0	1.3	ILLFLGCFR

```
1      MAEQHNTTSS SSAFVVIFAF QALAGMGINA FIVAASCMAW FKKKGMNSNE KILLFLGCFR FWYLCATWIY LIISVLFPQN
81     LLDTGISLTF AIFMCFLSSS NLWTSTCLYA FYLMKIANFR HHFFIYLKAR IDRIVPWLLL SSVVLSLLNC SPFLKVIDEE
161    NRTSPNFTTQ GIFWKTNEEI RKHFNSIISI CTCGFSMAFI LVTLFAFFLL FSLCRHKHKM QTSSTRSLSM DAHIKAMKSL
241    LSFFFTFSIH YILLITTVYY SSKENFLVLL LLVLQYSFPV IHSLILIFSN PRLERIALRI LPCAKCKECA RQPTETPMLC
321    S
```

5.443 PREDICTED: hypothetical protein [Gallus gallus]

Protein Accession	gi|118096653
Mean Expression Ratio	0.99
Median Expression Ratio	0.992
Credible Interval	(0.766, 1.28)
Associated Peptides	1
Associated Spectra	1
Coverage	0.0365

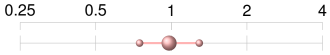

A	2.5	50	97.5	Sequence
1	0.75	0.98	1.3	AGPGRAGRGGARPSSGALCPR

```
1      MTVPRPVKAA KPNAPRGVTE PSDGAVQRSG PRLPLRPRGR EKRSPSPPKA PRRRRLTARS TEWRQQERPR PSEGGRSARS
81     RGRAAAAGRG LGGTAGGAPG AARRGSRSRA EGSCGPRAAP TESPPSRRRE EGWYRRAERR REDGRAVRAG QMGAAGERRG
161    PTHRAGPWSG GSSRSQAFKM AAARPQHPIP QAEGSARRNA PLSLPTSAGV GPGERPSSLC RGGAEKERDG QRRGGKGPAH
241    YFSRRKHTRR WLSRSGASAP WQSLMTSRAG RSRGALPRGA AVRAARSQAA FRAAPARARG GDLKGRGGRR RIPERRSARE
321    PPRAAIRTAR TGVSRAPCSW DGASPNGRLT RPRAARSRPR SLPRRSGLRR RSAPVLSAGA RSSPSESRGS RSPEVAAATG
401    AAGAPRRHRH SPRRPVRRFR RAGPCVITAR SSAGRGRAGG GRGRRWGRWG SRRRSRAMAG EPRAAAGAGR AYGGGAWLRR
481    LEAGRAGPGR AGRGGARPSS GALCPRGWSC GAGSGPARRF SPSRRGAARL VPCRWLGPGS AAGPGLTAPS VPGAGEAAAG
561    GGDPGAGVRQ PLRVPP
```

5.444 PREDICTED: hypothetical protein [Gallus gallus]

Protein Accession	gi|50755411
Mean Expression Ratio	1.01
Median Expression Ratio	1.01
Credible Interval	(0.777, 1.31)
Associated Peptides	1
Associated Spectra	1
Coverage	0.0413

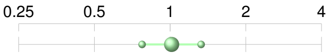

A	2.5	50	97.5	Sequence
**1**	**0.78**	**1.0**	**1.3**	**HIIVFQHIPLFLR**

```
1      MSVMAAVGDV FRRARGRTLT AFRQGDEYQW KGPFYFIQGA DPQFGLIKAW AVGNTGSGDD EWGEEIKLTE QAVQAINKLN
81     PKPKFFVLCG DLIHGMPGTQ WRKDQEQDLK NVLKNTDQDI PLVFVSGNHD IGNTPTRETI DNYCKSWGDD YFSFWVGGVF
161    FLVLNSQLYF DSSKCPELKQ AQDAWLNGQL AAAEKRKCKH IIVFQHIPLF LRKPDEDHDY FNLEKSVRQE IMEKFQNAGV
241    KAVFSGHYHR NAGGWYRGLE MVVSSAIGCQ LGEDKHGLRV VLVTDEKIVH RYYSLDELSS QGLEKEMVDM LAKQN
```

5.445 PREDICTED: hypothetical protein [Gallus gallus]

Protein Accession	gi|50732684
Mean Expression Ratio	0.992
Median Expression Ratio	0.992
Credible Interval	(0.764, 1.27)
Associated Peptides	1
Associated Spectra	1
Coverage	0.0474

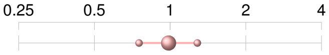

A	2.5	50	97.5	Sequence
**1**	**0.75**	**0.99**	**1.3**	**QATDASLPK**

```
1      MWRALLGGCR LLRSRSAAPV VGAPRAGGSA AVRNAGGGGA AAAAPEWRQA TDASLPKFSI DFVVNLLRQE NAKDICVIQV
81     PPEIKYCHYF IVVSGSSTRH LHAMAQYMLK MYKHQKEESD PHTRIEGKET HDWLCIDFGS IVVHFMLPET RETYELEKLW
161    TLGSYDDQLA QMTPQLLPED FILGLTSEEQ
```

5.446 adaptor-related protein complex 2, alpha 2 subunit [Gallus gallus]

Protein Accession	gi|61097989
Mean Expression Ratio	1.01
Median Expression Ratio	1.01
Credible Interval	(0.782, 1.30)
Associated Peptides	1
Associated Spectra	1
Coverage	0.0192

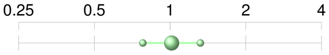

A	2.5	50	97.5	Sequence
**1**	**0.78**	**1.0**	**1.3**	**VGGYILGEFGNLIAGDPR**

```
1      MPAVSKGDGM RGLAVFISDI RNCKSKEAEI KRINKELANI RSKFKGDKAL DGYSKKKYVC KLLFIFLLGH DIDFGHMEAV
81     NLLSSNRYTE KQIGYLFISV LVNSNSELIR LINNAIKNDL ASRNPTFMGL ALHCIANVCS REMAEAFAGE IPKILVAGDT
161    MDSVKQSAAL CLLRLYRTSP DLVPMGDWTS RVVHLLNDQH LGVVTAATSL ITTLAQKNPE EFKTSVSLAV SRLSRIVTSA
241    STDLQDYTYY FVPAPWLSVK LLRLLQCYPP PDPAVRGRLT ECLETILNKA QEPPKSKKVQ HSNAKNAVLF EAISLIIHHD
321    SEPNLLVRAC NQLGQFLQHR ETNLRYLALE SMCTLASSEF SHEAVKTHIE TVINALKTER DVSVRQRAVD LLYAMCDRSN
401    AQQIVAEMLN YLETADYSIR EEIVLKVAIL AEKYAVDYTW YVDTILNLIR IAGDYVSEEV WYRVIQIVIN RDDVQGYAAK
481    TVFEALQAPA CHENLVKVGG YILGEFGNLI AGDPRSSPLI QFNLLHSKFH LCSVPTRALL LSTYIKFVNL FPEIKTTIQD
561    VLRSDSQLKN ADVELQQRAV EYLRLSTIAS TDILATVLEE MPPFPERESS ILAKLKKKKG PGTVTDLEEI KKERSSDMNG
641    SAEPASVNAS AVSTPSPSAD LLGLGAAPLT NSAPPPSSSG SLLVDVFSDS ASAVAPLAPG SDDNFARFVC KNNGVLFENQ
721    LLQIGLKSEF RQNLGRMFIF YGNKTSTQFL NFTPTVICSD DLQPSLNLQT KPVDPTVDGG AQVQQVVNIE CVSDFMEAPI
801    LNIQFRYGGT FQNLSVKLPI TLNKFFQPTE MSSQDFFQRW KQLSNPKQEV QNIFKAKHPM DAEITKAKII GFGSALLEEV
881    DPNPANFVGA GIIHTKTTQI GCLLRLEPNL QAQMYRLTLR TSKEAVSQRL CELLSEQF
```

5.447 nuclear calmodulin-binding protein [Gallus gallus]

Protein Accession	gi|3822553
Mean Expression Ratio	1.01
Median Expression Ratio	1.01
Credible Interval	(0.783, 1.30)
Associated Peptides	1
Associated Spectra	1
Coverage	0.0198

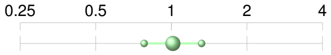

A	2.5	50	97.5	Sequence
**1**	**0.78**	**1.0**	**1.3**	**SQLGEDEFSYGYDGR**

```
1      GGGGGGGGGS AGGSGPCGGD AAGTAALRAG DEEEEEEEEE EEEEEEEEDE EALLAEEDPQ GGGGGRRAAP GTAEEEEEEE
81     EEGGEEGPAE PGKAAQPPVK DEEEVEEEAA VPEQPQAEGP EVGGGVNGAQ RAAEPEEGPS GDEAAAAADT DQKEEEEEEE
161    EAAPAADEAK AAGSEAERRG VKRQRDEKDE HGRAYYEFRE EAYNSRSKSP PPPEEEPREG EEDESVVILD TYTSDLHFRA
241    TKDRYGGQPL FFERFPALWS GARSTHGVTA GKVCFEAKVA QHLPLKEGST EVPLFRVGWS VDFSRSQLGE DEFSYGYDGR
321    GLKVESGRFE EFGQPFGEGD VIGCFADFEA SEEEVELSFS KNGEALGVAF RVPKEALGGR ALLPHVLCKG CAVQLNFGQT
401    EPLCPPAPPG YVFIHSVPPE QRVRTPPAPA HLQQCEVLLM VGLPGSGKTQ WAQKHSAENQ EKRYNMVGTE LVLHQMRTRG
481    PEVEELDAKS KDLLIQQAAQ CLSKLVQIAP RAKRNFILDQ VPPGGVSQTL LGRPPMSLFN FFPPFPPRFG QCNVYNSGQR
561    RKLLAFKGFC RKVVVVVPTE DDWKKRLELR KEAEGEDVPE SVMLEMKANY SLPEKSEYLD EVQYGELPKE EAHVGHQIQG
641    GGPETSAPLR ETRQPPQQPQ QAQPPEPQPR QGYVGGQRRG YDNRIYGQQQ YWGPQPGNRG GYRNFYSRYR GDYDRFYGRD
721    YEYNRYRDYY RHYNREWQNY YQDRDRYYRN YGYQGYR
```

5.448 heat shock 90kDa protein 1, beta [Gallus gallus]

Protein Accession	gi|47604960
Mean Expression Ratio	0.99
Median Expression Ratio	0.992
Credible Interval	(0.762, 1.28)
Associated Peptides	1
Associated Spectra	1
Coverage	0.0207

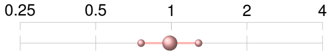

A	2.5	50	97.5	Sequence
**1**	**0.76**	**0.99**	**1.3**	**NPDDITQEEYGEFYK**

```
1      MPEQVQHGED EVETFAFQAE IAQLMSLIIN TFYSNKEIFL RELISNASDA LDKIRYESLT DPSKLDTGKD LKIDIVPNPR
81     DPTLTLLDTG IGMTKADLVN NLGTIAKSGT KAFMEALQAG ADISMIGQFG VGFYSAYLVA EKVVVITKHN DDEQYAWESS
161    AGGSFTVRTD HGEPIGRGTK VILYLKEDQT EYLEERRVKE VVKKHSQFIG YPITLYVEKE REKEVSDDEA EEEKVEKEEE
241    ESKDEEKPKI EDVGSDEEEE EGEKSKKKKT KKIKEKYIDQ EELNKTKPIW TRNPDDITQE EYGEFYKSLT NDWEDHLAVK
321    HFSVEGQLEF RALLFIPRRA PFDLFENKKK KNNIKLYVRR VFIMDSCDEL IPEYLNFIRG VVDSEDLPLN ISREMLQQSK
401    ILKVIRKNIV KKCLELFTEL AEDKENYKKF YEAFSKNLKL GIHEDSTNRK RLSELLRYHT SQSGDEMTSL SEYVSRMKES
481    QKSIYYITGE SKEQVANSAF VERVRKRGFE VVYMTEPIDE YCVQQLKEFD GKTLVSVTKE GLELPEDEEE KKNMEESKAK
561    FETLCKLMKE ILDKKVEKVT ISNRLVSSPC CIVTSTYGWT ANMERIMKAQ ALRDNSTMGY MMAKKHLEIN PDHPIVETLR
641    QKADANKNDK AVKDLVVLLF ETALLSSGFS LEDPQTHSNR IYRMIKLGLG IDEDEVIAEE SSIAPPDEIP PLEGDEDTSR
721    MEEVD
```

5.449 spectrin, alpha, non-erythrocytic 1 (alpha-fodrin) [Gallus gallus]

Protein Accession	gi|110227609
Mean Expression Ratio	0.992
Median Expression Ratio	0.992
Credible Interval	(0.869, 1.13)
Associated Peptides	9
Associated Spectra	9
Coverage	0.048

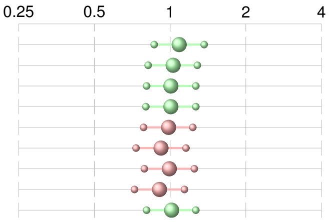

A	2.5	50	97.5	Sequence
1	0.86	1.1	1.4	ALINADELANDVAGAEALLDR
1	0.82	1.0	1.3	DIEDEETWIR
1	0.8	1	1.3	AGTFQAFEQFGQQLLAR
1	0.8	1	1.3	LNHQEFK
1	0.78	0.98	1.2	HQAFEAELHANADR
1	0.73	0.92	1.2	KFEEFQTDLAAHEER
1	0.79	1	1.2	KHEDFDK
1	0.72	0.9	1.1	KHQALQAEIAGHEPR
1	0.8	1.0	1.3	KHEAFETDFTVHK

```
1       MDPSGVKVLE TAEDIQERRQ QVLDRYHRFK ELSSLRRQKL EDSYRFQFFQ RDADELGKWI QEKLQIASDE NYKDPSNLQG
81      KLQKHQAFEA EVQANSGAIV KLDETGNQMI NEGHFASETI RTRLQELHRL WELLLEKMRE KGVKLLQAQK LVQFLRECED
161     VMDWINDKEA IVTSEELGQD LEHVEVLQKK FEEFQTDLAA HEERVNEVNQ FAGKLIQEQH PEEELIKSKQ DEVNASWQRL
241     KGLAQQRQGK LFGAAEVQRF NRDVDETISW IKEKGQLMAS DDFGRDLASV QALLRKHEGL ERDLAAFHHK VKALCAEADR
321     LQQSHPINAS QIQVKREELI ANWEQIRTLA AERHARLNDS YRLQRFLADF RDLTSWVTEM KALINADELA NDVAGAEALL
401     DRHQEHKGEI DAHEDSFRSA DESGQALLAA GHYASDEVKE KLTILSDERS ALLELWELRR QQYEQCMDLQ LFYRDTEQVD
481     NWMSKQEAFL LNEDLGDSLD SVEALLKKHE DFEKSLSAQE EKITALDEFA TKLIQNNHYA MDDVATRRDA LLSRRNALHE
561     RAMKRRAQLA DSFHLQQFFR DSDELKSWVN EKMKTATDEA YKDPSNLQGK VQKHQAFEAE LSANQSRIDA LEKAGQKLID
641     VNHYASDEVA ARMNEVISLW KKLLEATELK GIKLREANQQ QQFNRNVEDI ELWLYEVEGH LASDDYGKDL TSVQNLQKKH
721     ALLEADVAAH QDPIDGITIQ ARQFQDAGHF DADNIKKKQE ALVARYEALK DPMVARKQKL ADSLRLQQLF RDIEDEETWI
801     REKEPIAAST NRGKDLIGVQ NLLKKHQALQ AEIAGHEPRI KAVTQKGNAM VEEGHFAAED VKIKLNELNQ KWDSLKAKAS
881     QRRQDLEDSL QAQQYFADAN EAQSWMREKE PIVGSTDYGK DEDSAEALLK KHEALMSDLS AYGSSIQALR EQAQSCRQQV
961     APTDDETGKE LVLALYDYQE KSPREVTMKK GDILTLLNST NKDWWKVEVN DRQGFVPAAY VKKLDPAQSA SRENLLEEQG
1041    SIALRQEQID NQTLITKEVG SVSLRMKQVE ELYHSLLELG EKRKGMLEKS CKKFMLFREA NELQQWINEK EAALTNEEVG
1121    ADLEQVEVLQ KKFDDFQKDL KANESRLKDI NKVANDLESE GLMAEEVQAV EHQEVYGMMP RDETDSKTVS PWKSARMMVH
1201    TVATFNSIKE LNERWRSLQQ LAEERSQLLG SADEVQRFHR DADETKEWIE EKNQALNTDN YGHDLASVQA LQRNDEGFER
1281    DLAALGDKVN SLGETAQRLI QSHPELAEDL QEKCTELNQA WSSLGKRADQ RKEKLGDSHD LQRFLSDFRD LMSWINGIRG
1361    LVSSDELAKD VTGAEALLER HQEHRTEIDA RAGTFQAFEQ FGQQLLARGH YASPEIKEKL DILDQERTDL EKAWVQRRMM
1441    LDQCLELQLF HRDCEQAENW MAAREAFLNT EDKGDSLDSV EALIKKHEDF DKAINVQEEK IAVLQSFADQ LIAADHYAKG
1521    VIANRRNEVL DRWRRLKAQM IEKRSKLGES QTLQQFSRDV DEIEAWISEK LQTASDESYK DPTNIQLSKL LSKHQKHQAF
1601    EAELHANADR IRGVIEMGNP LIERGACAGS EDAVKARLAA LADQWEFLVQ KSSEKSQKLK EANKQQNFNT GIKDFDFWLS
1681    EVEALLASED YGKDLASVNN LLKKHQLLEA DISAHEDRLK DLNSQADSLM TSSAFDTSQV KDKRETINGR FQRIKSMAAA
1761    RRAKLNESHR LHQFFRDMDD EESWIKEKKL LVSSEDYGRD LTGVQNLRKK HKRLEAELAA HEPAIQGVLD TGKKLSDDNT
1841    IGKEEIQQRL AQFVDHWKEL KQLAAARGQR LEESLEYQQF VANVEEEEAW INEKMTLVAS EDYGDTLAAI QGLLKKHEAF
1921    ETDFTVHKDR VNDVCANGED LIKKNNHHVE NITAKMKGLK GKVSDLEKAA AQRKAKLDEN SAFLQFNWKA DVVESWIGEK
2001    ENSLKTDDYG RDLSSVQTLL TKQETFDAGL QAFQQEGIAN ITALKDQLLA AKHIQSKAIE VRHASLMKRW NQLLANSAAR
2081    KKKLLEAQEH FRKVEDLFLT FAKKASAFNS WFENAEEDLT DPVRCNSLEE IKALREAHDA FRSSLSSAQA DFNQLAELDR
2161    QIKSFRVASN PYTWFTMEAL EETWRNLQKI IKERELELQK EQRRQEENDK LRQEFAQHAN AFHQWIQETR TYLLDGSCMV
2241    EESGTLESQL EATKRKHQEI RAMRSQLKKI EDLGAAMEEA LILDNKYTEH STVGLAQQWD QLDQLGMRMQ HNLEQQIQAR
2321    NTTGVTEEAL KEFSMMFKHF DKDKSGRLNH QEFKSCLRSL GYDLPMVEEG EPDPEFESIL DTVDPNRDGH VSLQEYMAFM
2401    ISRETENVKS SEEIESAFRA LSSERKPYVT KEELYQNLTR EQADYCISHM KPYMDGKGRE LPSAYDYIEF TRSLFVN
```

5.450 6-phosphogluconolactonase [Gallus gallus]

Protein Accession	gi|71896147
Mean Expression Ratio	1.01
Median Expression Ratio	1.01
Credible Interval	(0.778, 1.3)
Associated Peptides	1
Associated Spectra	1
Coverage	0.056

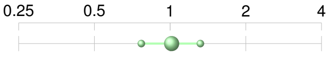

A	2.5	50	97.5	Sequence
1	0.77	1.0	1.3	ILEGNEENPLPAAR

```
1       MPGRISVFPS PQELGSALAQ LVVQRAAGSD GRFSLGLSGG SLVGLLAREL PLAATTGAAT GAGPWLVAFC DERLVPPEHP
81      ESTFGAYSAQ LLPWLPAPAP TVLAVAPGLS PNAAAADYAE RLREAFQGDA VPVFDLLLLG VGPDGHTCSL FPNHPLLQEK
161     EKIVAAITDS PKPPPQRITL TLPVLNAART VVFVATGEGK AAVLKRILEG NEENPLPAAR VRPHTGQLLW FLDEAAAKEL
241     TVPFEKHSIL
```

5.451 PREDICTED: similar to p80-coilin [Gallus gallus]

Protein Accession	gi|118099722
Mean Expression Ratio	0.991
Median Expression Ratio	0.993
Credible Interval	(0.765, 1.29)
Associated Peptides	1
Associated Spectra	1
Coverage	0.015

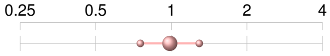

A	2.5	50	97.5	Sequence
**1**	**0.75**	**0.99**	**1.3**	**GFRGMVRGR**

```
1       MAAAGGAPVR LRLQFDFPPP GSPGCALCWL LLEPGQARLV TDLLSIIRHR FGFSRRARLS LFLDGALLPP TESARLVRDN
81      DALRVTLEEI AEEGCEEADD DFYAPKDDKK RHKRRQKVQG LSRSGEEKHQ REKKKNKYSP ECSSGREGTS GDVSDTRKKS
161     KKRKRREENS GTVPRPSEGK KGRADRPPKL QKAQKEERPA AKAKDGRRAA LPAAKAAHGR VGGSAPLMAA KSTSKNLTAE
241     AQKQRVGTSE SSSTSSDSDS SESNVKQSKS SHRPAAAALP RDKAQTAVTA AACNQMANAE HCSKAAVSKN AKKSQSSSSD
321     SDSSSEDERA AAAQGSTTEQ VLPNSAAAAQ TGPTGAPKAR SSSSSSDSSD SDTIVIKKPA ANSGLSNSIA RNGSKQSPAG
401     IQGPAAGLGR GRGRAVGEGN FWRGPRGRGF RGMVRGRGRG ESPGFFYSYS SEGQKQRQLH EAVTNTSVPV QNAVDVPKRD
481     YSVLPLLAAP PQVGERIAFK RLELNENYSP EVSSYKEGRI ISWNADKKQI ELEILSSPAS QIAKEPGKFD LVYQSADGAE
561     LIEYAVPQDT KITESWDSLI EPRLIVEPPM NGSGIENGAV
```

5.452 homeotic protein Hoxa-2 - chicken

Protein Accession	gi|422705
Mean Expression Ratio	1.01
Median Expression Ratio	1.01
Credible Interval	(0.775, 1.3)
Associated Peptides	1
Associated Spectra	1
Coverage	NaN

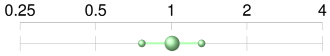

A	2.5	50	97.5	Sequence
**1**	**0.77**	**1**	**1.3**	**HSGGGRPKASPR**

5.453 WD repeat domain 1 [Gallus gallus]

Protein Accession	gi|52138693
Mean Expression Ratio	0.995
Median Expression Ratio	0.993
Credible Interval	(0.768, 1.29)
Associated Peptides	1
Associated Spectra	1
Coverage	0.0131

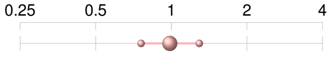

A	2.5	50	97.5	Sequence
**1**	**0.76**	**0.99**	**1.3**	**IKIPDAHR**

```
1       MRMPYEIKKV FASLPQVERG VSKIIGGDPK GNNFLYTNGK CVVIRNIDNP AIADIYTEHA HQVVVAKYAP SGFYIASGDV
81      SGKLRIWDTT QKEHLLKYEY QPFAGKIKDL AWTEDSKRIA VVGEGREKFG AVFLWDSGSS VGEITGHNKV INSVDIKQTR
161     PYRLATGSDD NCAAFFEGPP FKFKFTLSDH TRFVNCVRFS PDGNRFATAS ADGQIFIYDG KTGEKVCALG GGKAHDGGIY
241     AISWSPDSSQ LLSASGDKTA KIWDVGANSV VSTFNMGSNV LDQQLGCLWQ KDHLLSLSLS GYINYLDKNN PDKPLRVIKG
321     HSKSIQCLTV HKNGGKSYIY SGSNDGHINY WDSDTGENDG FSGKGHTNQV SRMAVDEMDQ LVTCSMDDTV RYTNLSKRDY
401     SGQDAVKMDV QPKCLAVGPG GYTVVLCIGQ IVLMKDKKKC FAIDDLGYEP EAVAVHPGGG SVAVGGTDGN VRLYSIQGTS
481     LKSDDKTLEA KGPVTDLAYS HDGAFLAVCD ANKVVTVFSV PDGYVEHNVF YGHHAKVVCI AWSPDNEHFA SGGMDMMVYV
561     WTVSDPETRI KIPDAHRLHH VSGLAWLDEH TLVTTSHDAS VKEWSISYN
```

5.454 PREDICTED: similar to ATP-dependent Lon protease [Gallus gallus]

Protein Accession	gi|118103080
Mean Expression Ratio	0.992
Median Expression Ratio	0.993
Credible Interval	(0.77, 1.27)
Associated Peptides	1
Associated Spectra	1
Coverage	0.0127

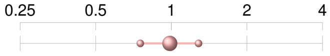

A	2.5	50	97.5	Sequence
1	0.75	0.99	1.3	KEAEEEPGAK

```
1     MTALTPLMVP EHFPNVPLIA VTRNPVFPRF IKIIEVKNKK LVELLRRKVR LAQPYAGVFL KKDDNNESDV VEDLNEIYQM
81    GTFVQIHEMQ DLGDKLRMIV MGHRRIRINR QLEVEPEEPE GKQKVRRKQK RPKKEAEEEP GAKEQAVEVV LDPVAASSQE
161   VLMVEVENVV HEDFQITEEV KALTAEIVKT IRDIIALNPL YRESVLQMMQ AGQRVVDNPI YLSDMGAALT GAESHELQDI
241   LEETSIPKRL YKALSLLKKE YELSKLQQRL GREVEEKIKQ THRKYLLQEQ LKIIKKELGL EKEDKDAIEE KFRERLKELV
321   VPKHVMDVID EELNKLSLLD NHSSEFNVTR NYLDWLTSIP WGKCSEENLE LSRAQAVLEE DHYGMDDVKK RILEFIAVSQ
401   LRGSTQGKIL CFYGPPGVGK TSIARSIARA LNREYFRFSV GGMTDVAEIK GHRRTYVGAM PGKIIQCLKK TKTENPLILI
481   DEVDKIGRGY QGDPSSALLE LLDPEQNSNF LDHYLDVPVD LSKVLFICTA NVTETIPEPL RDRMEVINVS GYVAEEKLAI
561   AERYLVPQAR VLCGLDENKA KITSDVLTVL IKQYCRESGV RNLQKQVEKV LRKSAYKIVS GEAETVQVTP ENLQDFVGKP
641   IFTVDRMYET TPPGVVMGLA WTAMGGSTLF VETSLKRPKD TEGKDGSLEV TGQLGDVMKE SAKIAYTFAR AFLMQKEPNN
721   DFLMSSHIHL HVPEGATPKD GPSAGCTIVT ALLSLAMNCP VRQNVAMTGE VSLTGKILPV GGIKEKTIAV
```

5.455 polymerase basic protein 1 [Influenza A virus (A/chicken/Thailand/PC-170/2006(H5N1))]

Protein Accession	gi|116664707
Mean Expression Ratio	1.01
Median Expression Ratio	1.01
Credible Interval	(0.78, 1.30)
Associated Peptides	1
Associated Spectra	1
Coverage	0.00793

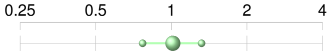

A	2.5	50	97.5	Sequence
1	0.77	1.0	1.3	QKLNKK

```
1     MDVNPTLLFL KVPVQNAIST TFPYTGDPPY SHGTGTGYTM DTVNRTHQYS EKGKWTTNTE TGAPQLNPID GPLPEDNEPS
81    GYAQTDCVLE AMAFLEESHP GIFENSCLET MEIVQQTRVD KLTQGRQTYD WTLNRNQPAA TALANTIEIF RSNGLTANES
161   GRLIDFLKDV MESMDKEEMD ITTHFQRKRR VRDNMTKKMV TQRTIGKKKQ KLNKKSYLIR ALTLNTMTKD AERGKLKRRA
241   IATPGMQIRG FVYFVETLAR SICEKLEQSG LPVGGNEKKA KLANVVRKMM TNSQDTELSF TITGDNTKWN ENQNPRVFLA
321   MITYITRNQP EWFRNVLSIA PIMFSNKMAR LGKGYMFESK SMKLRTQIPA EMLANIDLKY FNEITKKKIE KIRPLLIDGT
401   ASLSPGLMMG MFNMLSTVLG VSILNLGQKR YTKTTYWWDG LQSSDDFALI VNAPNHEGIQ AGVDRFYRTC KLVGINMSKK
481   KSYINRTGTF EFTSFFYRYG FVANFSMELP SFGVSGINES ADMSIGVTVI KNNMINNDLG PATAQMALQL FIKDYRYTYR
561   CHRGDTQIQT RRSFELKKLW EQTRSKAGLL VSDGGPNLYN IRNLHIPEVC LKWELMDEDY QGRLCNPLNP FVSHKEIESV
641   NNSVVMPAHG PAKSMEYDAV ATTHSWIPKR NRSILNTSQR GILEDEQMYQ KCCNLFEKFF PSSSYRRPVG ISSMVEAMVS
721   RARIDARIDF ESGRIKKEEF AEIMKICSTI EELRRQK
```

5.456 vimentin [Gallus gallus]

Protein Accession	gi|114326309
Mean Expression Ratio	1.01
Median Expression Ratio	1.01
Credible Interval	(0.888, 1.14)
Associated Peptides	9
Associated Spectra	18
Coverage	0.215

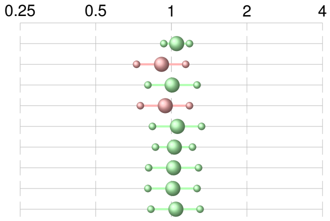

A	2.5	50	97.5	Sequence
8	0.93	1.0	1.2	EMEENFAVEAANYQDTIGR
1	0.73	0.92	1.1	INMPIPTFASLNLR
1	0.8	1	1.3	FADLSEAANR
1	0.75	0.94	1.2	LGDLYEEEMR
1	0.84	1.1	1.3	NLQEAEEWYK
3	0.86	1.0	1.2	ILLAELEQLK
1	0.81	1.0	1.3	QQYESVAAK
1	0.8	1.0	1.3	KLLEGEESR
1	0.83	1.0	1.3	KLHDEEIR

1 MSFTSSKNSS YRRMFGGGSR PSSGTRYITS STRYSLGSAL RPSSARYVSA SPGGVYRTKA TSVRLRSSMP PMRMHDAVDF
81 TLADAINTEF KANRTNEKVE LQELNDRFAN YIDKVRFLEQ QNKILLAELE QLKGKGTSRL GDLYEEEMRD VRRQVDQLTN
161 DKARVEVERD NLADDIMRLR EKLQEEMLQR EEAESTLQSF RQDVDNASLA GLDLERPVES LQEEIVFLKK LHDEEIRELQ
241 AQLQEQHIQI DMDVSKPDLT AALRDVRQQY ESVAAKNLQE AEEWYKSKFA DLSEAANRNN DALRQAKQEA NEYRRQIQSL
321 TCEVDALKGS NESLERQMRE MEENFAVEAA NYQDTIGRLQ DEIQNMKEEM ARHLREYQDL LNVKMALDIE IATYRKLLEG
401 EESRINMPIP TFASLNLRET NIESQPIVDT HSKRTLLIKT VETRDGQVIN ETSQHHDDLE

5.457 dihydropyrimidinase-like 2 [Gallus gallus]

Protein Accession	gi|45383177
Mean Expression Ratio	1.01
Median Expression Ratio	1.01
Credible Interval	(0.809, 1.25)
Associated Peptides	2
Associated Spectra	2
Coverage	0.0281

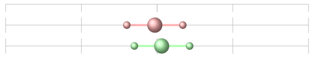

A	2.5	50	97.5	Sequence
1	0.76	0.98	1.3	SAAEVIAQAR
1	0.81	1.0	1.4	KPFPDFVYK

1 MAEKKQSGRQ GEEEEVPAFF KNLGSGSPKP RQKFCGMFCP VEGSLENKTI DFDSLSVGRG SNKVVAKQKD VAHLGPDAQS
81 VYSRQSGKGG EPSVDFGRKV EIRSATGKEA LQNLNDKSDR LLIKGGKIVN DDQSFYADIY MEDGLIKQIG ENLIVPGGVK
161 TIEAHGRMVI PGGIDVHTRF QMPEQGMTSA DDFFQGTKAA LAGGTTMIID HVVPEPGTSL LTAFDQWREW ADSKSCCDYS
241 LHVDITEWHK GVQEEMEALV KDHGVNSFLV YMAFKDRFQL SDSQIYEVLS VIRDIGATAQ VHAENGDIIA EEQQRILELG
321 ITGPEGHVLS RPEEVEAEAV NRAITIANQT NCPLYITKVM SKSAAEVIAQ ARKKGTVVYG EPITASLGTD GSHYWSKNWA
401 KAAAFVTSPP LSPDPTTPDF LNSLLSCGDL QVTGSAHCTF NTAQKAVGKD NFTLIPEGTN GTEERMSIIW DKAVVTGKMD
481 ENQFVAVTST NAAKIFNLYP RKGRIAVGSD ADLVIWDPDS VKTISAKTHN ISLEYNIFEG MECRGSPLVV ISQGKIVLED
561 GNLHVTEGSG RYIPRKPFPD FVYKRIKARS RLAELRGVPR GLYDGPVCEV SVTPKTVTPA SSAKTSPAKQ QAPPVRNLHQ
641 SGFSLSGAQI DDNIPRRTTQ RIVAPPGGRA NITSLG

5.458 aminoimidazole-4-carboxamide ribonucleotidetransformylase/IMP cyclohydrolase [Gallus gallus]

Protein Accession	gi|53778215
Mean Expression Ratio	1.01
Median Expression Ratio	1.01
Credible Interval	(0.828, 1.23)
Associated Peptides	3
Associated Spectra	3
Coverage	0.0708

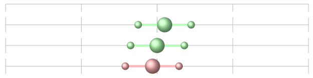

A	2.5	50	97.5	Sequence
1	0.84	1.1	1.4	DGQVIGIGAGQQSR
1	0.78	1	1.3	LTAVSLSSDAFFPFR
1	0.75	0.96	1.2	TLHPAVHAGILAR

1 MAARQQLALL SVSEKAGLVE FARSLNALGL GLIASGGTAT ALRDAGLPVR DVSDLTGFPE MLGGRVKTLH PAVHAGILAR
81 NIPEDNADMN KQDFSLVRVV VCNLYPFVKT VSSPGVTVPE AVEKIDIGGV ALLRAAAKNH ARVTVVCDPA DYSSVAKEMA
161 ASKDKDTSVE TRRHLALKAF THTAQYDAAI SDYFRKEYSK GVSQLPLRYG MNPHQSPAQL YTTRPKLPLT VVNGSPGFIN
241 LCDALNAWQL VKELKQALGI PAAASFKHVS PAGAAVGIPL SEEEAQVCMV HDLHKTLTPL ASAYARSRGA DRMSSFGDFI
321 ALSDICDVPT AKIISREVSD GVVAPGYEEE ALKILSKKKN GAYCVLQMDP NYEPDDNEIR TLYGLQLMQK RNNAVIDRSL
401 FKNIVTKNKT LPESAVRDLI VASIAVKYTQ SNSVCYAKDG QVIGIGAGQQ SRIHCTRLAG DKANSWWLRH HPRVLSMKFK
481 AGVKRAEVSN AIDQYVTGTI GEDEDLVKWQ AMFEEVPAQL TEAEKKQWIA KLTAVSLSSD AFFPFRDNVD RAKRSGVQFI
561 VAPSGSAADE VVIEACNELG ITLIHTNLRL FHH

5.459 trimethyllysine hydroxylase, epsilon [Gallus gallus]

Protein Accession	gi|60302798
Mean Expression Ratio	1.01
Median Expression Ratio	1.01
Credible Interval	(0.785, 1.31)
Associated Peptides	1
Associated Spectra	1
Coverage	0.0359

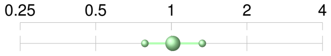

A	2.5	50	97.5	Sequence
**1**	**0.78**	**1.0**	**1.3**	**TLLVDGFYAAEQVLR**

```
1      MWCRRLACLL SVPCQHTRHR LLGPSCGRRT FTAAVARWHH TAPESLSCAW QLHGDHLELR YADTLMRFDF VWLRDHCRSA
81     SCYNAKTNQR SLDTASVDLS IKPKAVRVDE TTLFLTWPDG HVTRYGLQWL VKNSYEGQKQ QVMHPRILWN AEIYRQAQVP
161    SVDCQSFLET DEGLKEFLQN FLLYGIAFVE NVTPTKEDTQ ILAERISLIR ETIYGRMWYF TSDFSRGDTA YTKLALDRHT
241    DTTYFQEPCG IQVFHCLKHE GTGGRTLLVD GFYAAEQVLR QAPDQFELLS KVPLKHEYIE NVGDCHNHMI GVGPVLNVYP
321    WNNELYLIRY NNYDRAVINT VPYDVVNRWY TAHRTLTTEL RRPENELWVK LKPGKALFID NWRVLHGREA FTGYRQLCGC
401    YLTRDDVLNT ARLLGLQA
```

5.460 alanyl-tRNA synthetase [Gallus gallus]

Protein Accession	gi|57524852
Mean Expression Ratio	0.993
Median Expression Ratio	0.993
Credible Interval	(0.768, 1.28)
Associated Peptides	1
Associated Spectra	1
Coverage	0.0131

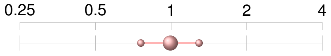

A	2.5	50	97.5	Sequence
**1**	**0.76**	**1**	**1.3**	**AVFDETYPDPVR**

```
1      MESTLTASQI RQRFIDFFKE NQHTYVHSSS TIPLDDPTLL FANAGMNQFK PIFLNTIDPS HPLAKLSRAT NTQKCIRAGG
81     KHNDLDDVGK DVYHHTFFEM LGSWSFGDYF KELACKLALD LLTKEFGIPA ERLYVTYFGG NEAAGLQPDL ECKQIWLDLG
161    LAEGRILPGN MKDNFWEMGD TGPCGPCSEI HYDRIGDRDA SHLVNQDDPN VLEIWNLVFI QFNREADGSL KPLPKKSIDT
241    GMGLERLVSV LQNKMSNYDT DLFLPYFEAI QKGTGARPYM GQVGAEDADG IDMAYRVLAD HARTITLALS DGGRPDNTGR
321    GYVLRRILRR AVRYSHEKLN APKGFFATLV DVVVQSLGDA FPELKKDPDM VKDIINEEED QFLKTLSRGR RILDRKIQSM
401    GDSKTIPGDT AWLLYDTYGF PVDLTGLIAE EKGLVVDMEG FEEERKNAQL KSQGKGAGGE DLLMLDIYAI EELRARGLEV
481    TDDSPKYGYT SDPSGTYDFG SLVATVKAIR REKKFVEEVS TGQECGIVLD RTCFYAEQGG QIYDQGYMVK DDDSKEDKTE
561    FTVKNVQVRG GYVLHIGTLY GSLKVGDQVH LSIDETRRRP VMSNHTATHI LNFALRSVLG EADQRGSLVA PDRLRFDFTA
641    KGALSTQEIK KVEGIANQMI EEAKPVYARD CPLAAAKAIQ GLRAVFDETY PDPVRVVSIG IPVEELLADP SGPAGSITSI
721    EFCGGTHLQN SGHAGPFVIV SEEAIAKGIR RIVAVTGAEA RKALRKVESL KKVLSALDAK VKVQTAPNKD VQKEITDLSE
801    MLATAVIPQW QKDELREAVK ALKKVMDDLD RASKADIQKR VLEKTKQVIE SHPNQPLVIM EMENGASAKA LNESLKLFKT
881    HSPQTATMLF AVDNEAGRIT CLCQVPRRRC RRA
```

5.461 restin [Gallus gallus]

Protein Accession	gi|45384404
Mean Expression Ratio	1.01
Median Expression Ratio	1.01
Credible Interval	(0.776, 1.3)
Associated Peptides	1
Associated Spectra	1
Coverage	0.00628

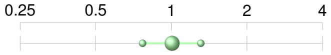

A	2.5	50	97.5	Sequence
**1**	**0.77**	**1**	**1.3**	**EKDAIHQEK**

```
1       MSMLKPSGLK APSKTIKHGS TLLKAPASVA TAPAEKAPSS EKSSSTTTAD AHDDFVDDFR VGERVWVNGN KPGFIQFLGE
81      TQFAPGQWAG IVLDEPIGKN DGSVAGVRYF QCEPLRGIFT RPSKLSRKVL TEDEANGTQT AHASRATSPT STSTASAVSA
161     SPAALLPSGI PQKTSPLAAK EHSTPSQFSN LSKTASGSVS NLSEAGSLKK GERELKIGDR VLVGGTKAGV VRFLGETDFA
241     KGEWCGVELD EPLGKNDGAV AGTRYFQCQP RYGLFAPVHK VTKIGFPSTT PAKAKTTVRK VVATPAALKR SPSASSLSSL
321     SSVASSVSSK PSRTGLLTET SSRYARKISG TTALQEALKE KQQHIEQLLA ERDLERAEVA KATSHVGEIE QELALVRDGH
401     DRHVLEMEAK MDQLRAMVEA ADREKVELLN QLEEEKRKVE DLQFRVEEES ITKGDLETQT KLEHARIKEL EQSLLFEKTK
481     ADKLQRELED TRVATVSEKS RIMELERDLA LRVKEVAELR GRLESSKHID DVDTSLSLLQ EISSLQEKMA AAGKEHQREM
561     SSLKEKFESS EEALRKEIKT LSASNERMGK ENESLKTKLD HANKENSDVI ELWKSKLESA IASHQQAMEE LKVSFNKGVG
641     AQTAEFAELK TQMEKVKLDY ENEMSNLKLK QENEKSQHLK EIEALKAKLL EVTEEKEQTL ENLKAKLESV EDQHLVEMED
721     TLNKLQEAEI KVKELDVLQA KCNEQTKLIG SLTQQIRASE EKLLDLAALQ KANSEGKLEI QKLSEQLQAA EKQIQNLETE
801     KVSNLTKELQ GKEQKLLDLE KNLSAVNQVK DSLEKELQLL KEKFTSAVDG AENAQRAMQE TINKLNQKEE QFALMSSELE
881     QLKSNLTVME TKLKEREERE QQLTEAKVKL ENDIAEIMKS SGDSSAQLMK MNDELRLKER QLEQIQLELT KANEKAVQLQ
961     KNVEQTAQKA EQSQQETLKT HQEELKKMQD QLTDMKKQME TSQNQYKDLQ AKYEKETSEM ITKHDADIKG FKQNLLDAEE
1041    ALKAAQKKND ELETQAEELK KQAEQAKADK RAEEVLQTME KVTKEKDAIH QEKIETLASL ENSRQTNEKL QNELDMLKQN
1121    NLKNEEELTK SKELLNLENK KVEELKKEFE ALKLAAAQKS QQLAALQEEN VKLAEELGRS RDEVTSHQKL EEERSVLNNQ
1201    LLEMKKREST LKKEIDEERA SLQKSISDTS ALITQKDEEL EKLRNEITVL RGENASAKTL QSVVKTLESD KLKLEEKVKN
1281    LEQKLKAKSE QPLTVTSPSG DIAANLLQDE SAEDKQQEID FLNSVIVDLQ RRNEELNLKI QRMCEAALNG NEEETINYDS
1361    EEEGLSKKTP RLFCDICGCF DLHDTEDCPT QAQMLEEPPH STYHGSRREE RPYCDTCEMF GHWTADCNDD ETF
```

5.462 PDZ and LIM domain 5 [Gallus gallus]

Protein Accession	gi|71896681
Mean Expression Ratio	0.995
Median Expression Ratio	0.993
Credible Interval	(0.77, 1.29)
Associated Peptides	1
Associated Spectra	1
Coverage	0.0235

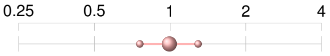

A	2.5	50	97.5	Sequence
**1**	**0.76**	**1**	**1.3**	**ENQGDNKQQNGPPR**

```
1       MSNYSVSLVG PAPWGFRLQG GKDFNMPLSI SRLNDGGKAA RANVGIGDVV LTIDGISTDG MTHLEAQNKI KACTGNLNMT
81      LQRVASATKP DIIHVPKEEP KEVVRPVPVA SAAAPKVTAM ASVAYNKTPR PFGAVASSKV TSIPSPSSAF TPAQAAPMLP
161     TPAPFAAPGL HVNAKPNADG WPPAPSTAKP AVNVPRQPAT HNAAPGGHVG DSTQELAEGL RRGFRENQGD NKQQNGPPRK
241     HIVDTYTEFY HTPTHSDASK KRLIEDTEDW HPRTGTTQSR SFRILAQITG TDHMKEPEHE GAKKSNDSME PTPPAAYTPT
321     SVRNVSASHE SPDPRPIPTN NATSPSAPKP AGPSSAAKYS GWQPPSQAAP ASGWTSNSNK TSGTTAPSQK LAAASQLTEQ
401     DTLVQRAEHI PAGKRTPMCA QCNQVIRGPF LVALGKSWHP EEFNCAHCKT SMAYIGFVEE KGALYCEVCY EKFFAPECSK
481     CQRKILGEVI NALKQTWHVS CFVCVACHNP IRNNVFHLED GDPYCETDYY ALFGTMCHGC EFPIEAGDRF LEALGHTWHD
561     TCFVCSVCND SLEGQTFFSK KDKPLCKKHA HSINI
```

5.463 PREDICTED: similar to cytosolic NADP-dependent isocitrate dehydrogenase [Gallus gallus]

Protein Accession	gi|118093509
Mean Expression Ratio	0.995
Median Expression Ratio	0.994
Credible Interval	(0.806, 1.23)
Associated Peptides	2
Associated Spectra	3
Coverage	0.0434

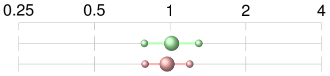

A	2.5	50	97.5	Sequence
**1**	**0.79**	**1.0**	**1.3**	**FKDIFQEIYDR**
**2**	**0.8**	**0.97**	**1.2**	**KYHVGIK**

```
1       MSKKIHGGSV VEMQGDEMTR VIWELIKEKL IFPYVDLDLH SYDLGIEHRD ATNDKVTVEA AEAIKKYHVG IKCATITPDE
81      KRVEEFKLKQ MWKSPNGTIR NILGGTVFRE AIICKNIPRL VSGWVKPIVI GRHAYGDQYR ATDFVVPGPG KVEMTYTPGD
161     GGKPVTYLVH NFESCGGVAM GMYNLDQSIK DFAHSSFQMA LSKGWPLYMS TKNTILKRYD GRFKDIFQEI YDREYKSQFE
241     AKKIWYEHRL IDDMVAQALK SEGGFVWACK NYDGDVQSDS VAQGYGSLGM MTSVLICPDG KTVEAEAAHG TVTRHYRMHQ
321     KGQETSTNPI ASIFAWTRGL AHRAKLDNNT SLKTFATALE EVCIETIESG FMTKDLAACI KGLPNVTRSD YLNTFEFMDK
401     LAANLKGKLA SLPKL
```

5.464 Parkinson disease (autosomal recessive, early onset) 7 [Gallus gallus]

Protein Accession	gi|45383015
Mean Expression Ratio	1.01
Median Expression Ratio	1.01
Credible Interval	(0.776, 1.3)
Associated Peptides	1
Associated Spectra	1
Coverage	0.074

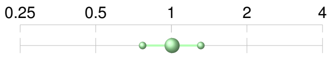

A	2.5	50	97.5	Sequence
1	0.77	1	1.3	DVLICPDASLEDAR

```
1       MASKRALVIL AKGAEEMETV IPTDVMRRAG IKVTVAGLTG KEPVQCSRDV LICPDASLED ARKEGPYDVI VLPGGNLGAQ
81      NLSESAAVKD ILKDQESRKG LIAAICAGPT ALLAHGIGFG SKVITHPLAK DKMMNGAHYC YSESRVEKDG NILTSRGPGT
161     SFEFGLAIVE ALMGKEVAEQ VKAPLILKD
```

5.465 cgABP260 [Gallus gallus]

Protein Accession	gi|45383033
Mean Expression Ratio	1.01
Median Expression Ratio	1.01
Credible Interval	(0.904, 1.12)
Associated Peptides	14
Associated Spectra	18
Coverage	0.0854

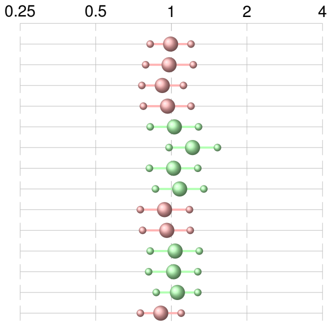

A	2.5	50	97.5	Sequence
2	0.82	1	1.2	CDGDPLPGSPLQFYVDAVDAR
1	0.79	0.98	1.2	DVSTEFTVDAR
2	0.77	0.92	1.1	DAGEAELNIEIR
1	0.77	0.96	1.2	AALDVTLLGPTGTPEPVEVR
1	0.83	1.0	1.3	VLASGPGLSPSGVPASLPVEFAIDAR
1	0.98	1.2	1.5	VSEPVDASQVR
1	0.82	1.0	1.3	GAGSGALGLTVEGPR
1	0.87	1.1	1.3	LENVSVALEFLER
1	0.75	0.94	1.2	AEVAQSGDGTVAVR
1	0.77	0.96	1.2	VHAFGPGLHGGVAGSPATFTISTR
1	0.83	1.0	1.3	GQHVPGSPFPFTVGPLGEGGAHR
1	0.81	1.0	1.3	LIALLEVLSQKK
2	0.87	1.1	1.3	KGGQHVTNSPFK
2	0.75	0.9	1.1	YGGPHHIVGSPFK

```
1       MNSSGYEETA VPEDAEELPA AEKELAEDAP WKKIQQNTFT RWCNEHLKCV NKSISDLQRD LGDGLRLIAL LEVLSQKKMG
81      RKHHPRPNFR QMKLENVSVA LEFLERERIK LVFIDSKAIV DGNLKLILGL VWTLILHYSI SLPMWEEEEE GARQQTPKQR
161     LLGWVQHRVP QLRITNFNRD WNDGRALGAL VDNCAPGLCP DWQSWDPAQP VQNAREAMQQ ADDWLGVPQV IAPEEIADPN
241     VDEHSVMTYL SQFPKAKLKP GAPLRPRAVR PERVRAYGPG LEPQGNVVLQ PARFTVETWT RARQVLENAR DHRAHHEEAQ
321     VVANNDEKRT FSVTYVPKVA GLHKVTVLFG GQNIPGSPFV GVAMAHGDAS KVSARGPGIE PSGNVANKPT YFDIYTAGAG
401     SGDVGVVLED PAGPRDTVEG DMEDRGDSTF RCSYRPTLPG PHRVAVTFAG AHIPNSTFCV NVAEACNPSA CRAWGRGLQP
481     KGLRVHETAD FRVHTNAPAE LRVTVPDRGT EVPVSVRPTA DGVYECEYRP TVAGTHSVSI TWGGYSIPRS PLEVEVSPAA
561     GAQKVRAWGP GLHGGVVGDS ADFVVEAIGD DVRTLGFSIE GPSQPKIESD DPGDGSCDVR YWPTEAGPYP VHVVCDDEDI
641     ARSPFMAHIR PAAPDCSPDK VKVWGPGLEP TGVIVNRPTE FCVDARAAGK GGLHIYAQDA DGCPVDIAVK DNGDGTFSCH
721     YVPSKAIKHT VIVTWGGVNV PRSPFRVAVG KGSHPGRVRV YGPGVERAGL TAGEPTYFTV DCSDAGQGDV SIGIKCCPAL
801     PGAAEADIDF TIIKNDNDTF TVKYTPPCAG LLTVMVLFGT QELPCSPFRI TVAPAHDSSK VRAEGPGISR TGVESGRPTH
881     FTVQTRGAGK APLDVQFGGA AQSGVSDVQI IDNGDYSHTV KYTAMRQGEL SVGVTYGGDP IPKSPFPVTV APPLQLDKVS
961     VQGLSSKVPV GAPQSFSVLS SGAGGQGALS VTAMGAGRRS VPVAVESAGP GLHRARFTAA EEGQLRFDVT YDGHPVPGSP
1041    FVVDAVLPPD PSKVHAFGPG LHGGVAGSPA TFTISTRGAG SGALGLTVEG PREAELECHD NGDGSCSVRY VPPEPGDYSL
1121    NILFAGTHVP GSPFRAAVRP RFDATKVRAD GPGLRSGRVG QIATFAVDCR DAGEAELNIE IRHELGARPE VRIHDNGDGT
1201    YGISYTPARA GAYTVTVSYG GVPVPHFPAC VTVEPDVDLS AIAVYGTGVE PRGPLRDVST EFTVDARAVV PSGGPHVGAR
1281    VTAPSGATTD CSVSDGGDGT YRVRYTPREE GVHQVEVTFQ EVALPQSPFA VAVAEGCDPT RVSAHGPGLE SGLVGRANCF
1361    TVQTRGAGTG GLGLAIEGPS EAKMSCQDNK DGSCSVEYVP FAAGDYDVNI TFGGRPIPGS PFRVRVSEPV DASQVRCSGA
1441    GLGPTVRARL PHSVNVDCSA AGRAALDVTL LGPTGTPEPV EVRDNGDGTH SVTYSPTAVG PHSLSIAYGG QEVPRTFKLQ
1521    AVPHDASRVL ASGPGLSPSG VPASLPVEFA IDARRAGQGV LTVQILDPEG RPTEASIEDH GDGTFTVRYL PRLVGRYSIT
1601    VRYGGDDIPA SPFCIHGAPS GNASKCHVTG GCAFPTLRPG ERTLLAVDAR AAGPGKVTCS VLSPDGAEVD VDVAENPDGT
1681    FHVSYTAPEP GSYGLTVRFG GQHVPNSPFR IVATAEPPRP RDLRPFSLDL PCAPLKGDIT AEVRTPSGRT ARAEVAQSGD
1761    GTVAVRFEPS EKGRHLMELR CDGDPLPGSP LQFYVDAVDA RHVSAYGAGL SHGVVNKACS FTVITKGAGE GGLSLAVEGP
1841    SKAELSCHDN HDGTCTVSYV PTAPGDYSVI VRFDDEHIPG SPFTAKITGD DSLRTSQLNV GTATDVSLKI SETDLSQLTA
1921    SIRAPSGSEE PCLLKRLPNR HIGISFTPKE VGEHVVSVRK GGQHVTNSPF KIVVGPSEIG NAERVKVWGT GLSEGRTFQL
2001    AHFVVDTRSA GYGGLGLSVE GPSKVDIHCE DAEDGTCKVS YCPTEPGSYR LSVKFAEQHV PGSPFTVKVS GEGRVKESIT
2081    RARQAASIAA IGSACDLNLK IPGNWFQMVS AQERLTRTFT RSSHTYTRTE RTEISKTRGG ETTREVRVQE STRVGAEPFH
2161    GVFGGFLGGF GAAARGQEDA APPELTAQVL SPSGQHSEAE VQAGPGSAYR VRFVPEELGP HSVAVKFRGQ HVPGSPFPFT
2241    VGPLGEGGAH RARAGGPGLQ RGVAGVPAEF SIWTREAGAG GLSIAVEGPS KAEINFEDRK DGSCGVTYLV QEPGDYEVSI
2321    KFNEEHIPDS PFVVPVASRS DAAQHSTVSS AQEALRVNHP AALSVHLNGA RRPFDAKVLS PTGTVSECSV SEIDPDLYSI
2401    GFLPVENGVH AVEVRLNGRH VPGSPFNVRV GEQSHDADPG LVTAYGAGLQ GGVTGVRSEF VVKTANAGSG ALSVTIDGPS
2481    KVTLDCVECA EGHRVTYTPM APGNYLISIK YGGPHHIVGS PFKAKVTGPR LSGGHSLHET SSVLVETVTR GALGPPGFGT
2561    LPKFSSDASK VVARGPGLST AFVGQKNHFT VDCSKAGSNM LMVGVHGPKT PCEEVYVKHV GNRLYNVTYT VKEKGEYVLI
```

```
2641     LRWGDDSVPG SPFRVTVP
```

5.466 PREDICTED: similar to translocated promoter region (to activated MET oncogene) [Gallus gallus]

Protein Accession	gi|118094253
Mean Expression Ratio	0.995
Median Expression Ratio	0.994
Credible Interval	(0.767, 1.29)
Associated Peptides	1
Associated Spectra	1
Coverage	0.00633

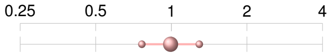

A	2.5	50	97.5	Sequence
1	0.76	1	1.3	NLQEQISQLQSELAR

```
1        MAAVLQQVLE RAELAKLSKP VQGKLERFLA DQQSEIDGLR ARHERFKVDS EQQYFEVEKR LAQSQERLVN ETQQCQTLRE
81       ELKKLHEQLK LLNEKNKELE AAQDRNAAIQ SHLSREKEEL EAEKRDLVRT SERRSQEVEH LNEDVKRLNE KLTEANTEKV
161      KLQLKLDELQ TSDVTVKYRE KRLEQEKELL QSQNTWLNSE LKAKTDELLH TAREKGSEIL ELKCNLENKK EEVSRMEEQI
241      NSLKQSNENL QKHVEDLLNK LKEAKEQQAS MEERFHNELN AHIKLSNLYK SAADDSEAKS NELTGAVEEL HKLLKEAGEA
321      NKATQEHLAE VEESKAVMEK ELREKISKLE KELENANDLL SATKRKGAVL SEEELAAMSP TAAAVAKVVK PGMKLTELYN
401      AYVETQDQLL LEKLENKRIN KYLDEIVQEV EAKAPILKRQ REEFERSQKA VASLSAKLEQ AMKEIQRLQE DADKANKQTS
481      MLERENQRLE IQVKDLSQQI RVLLMELEEA RGNHVIRDEE VSSADISSSS EVISQHLVSY RNIEELQQQN QRLLVALREL
561      GEAREKEEQE TTSSKISELQ SQLDEALNEL EKLRESRHHQ LQLVESIVRQ RDMFRILLAQ TTGAIIPLQA SGMLPEEISL
641      TSTPKRSSIP QAMSTPAPVS MSESVETAEA KAALKQLQEV FENYKKEKAE NDKLLNEQNE KLQEQVTELR SQNAKISTQL
721      EFASKRYEML QDNVEGYRRE ITSLHERTQK LSATTQKQEQ IINTMTQDLR GANEKLAVAE VRAENLKKEK DILKMSDVRL
801      TQQRESLLVE QRGQNLLLTN LRTIQGILER SETETKQRLN NQVEKLEREI SQLKKKLESE VEQRHALTKN QEVHILDLKR
881      QLETETNRHI NTKELLKNAQ KEATVLKQQL NNTEAQLASQ SSQRAPGKGQ PGTSEDVDDL VSRLRQADEQ VNDLRERLRT
961      SSSNVEQYRA MVLSLEESLN KEKQVTEEVR ATVEARLKES SEYQAQLEKK LMESEKEKQE LQEEKRKAVE NMEQQLSELK
1041     KSLSTLQSEV QEALQRASTA LSNEQQARRD CQEQAKMASE AQNKYERELM LHAADVEALQ AIKEQVAKNT AVKQQLEEAA
1121     QKAESALLEC KASWEERERM MKDEASTLAS RCEDLEKQNR LLHEQLESLS DKMVTSMKEA MPAALNVSLN EEGKSQEQIL
1201     EILRFIRREK EIAETRFEVA QVESLRFRQR VEHLERELQE VQDSLNAERE KVQVTAKTIA QHEELMKKTE TMNVLIETNK
1281     MLREEKERLE QELQQMQAKV RKLEADILPL QESNAELSEK SGMLQAEKKL LEEDVKRWKT RTQHLLSQQK DTDLEEYRKL
1361     LSEKEANTKR IQQMSEETGR LKAEIARTTA SLTTSQNLLQ NLKDEVAKIR TEKETLQKEL DAKVADIQEK VKTITQVKKI
1441     GRRYKTQYEE LKAQHDKMVA EAATQSFVEQ QEEQVSVQEV QELKDSLSQA EGKTKTLENQ VENLQKTVAE KETEARNLQE
1521     QISQLQSELA RFRQDLQEKT TQEEQLRQQI TEKEEKTRKT FLAAKQKIAQ LAGTKEQLTK ENEEWKQKSN SLEEQKTELE
1601     VRMSALKSQY EGRICRLERE LREQQERHHE QRDEPPESTN KVPEQQRQIS LKSTPASGER GIASTSDPPT ANIKPTPVVS
1681     TPSKVTAAAI AGNKSTPRAS IRPMVTPATV TNPTTTPTAT VMPTTQVETQ EAMQSEGPVE HVPVFGSTSG SVRSTSPNVQ
1761     TSLSQPILTV QQQTQATAFV QPTQQSHPQI EPANQEPSPT IVEVVQSSQI ERPSTSTAVF GTVSATPSSS LSKRPREEEE
1841     DNTVENSDQI SEETVDVPLP KKMRSIQRVG LEEEVTAEES TDGEVEAQTY NQDSQDSIGE GVTQGEYTPM EDSEETSQSI
1921     PIDLGPLQSD QQNTTSSQDG QSKRDDVIVI DSDDEDDDDD ENEGEQEDYE DEEEEDEDDD EDTGMGDEGD DSNEGTGSAD
2001     GNDGYEADDA EGADGTDPGT ETEESLGGGE SNQRAADSQN SCEGSTSTAE STFPHESSRE QQPSSASERQ APRPPQSPRR
2081     PPHPLPPRLT IHAPPQELGP PVQRIQMTRR QSVGRGLQLT PGIGGMQQHF FDDEDRTVPS TPTLVVPHRT DGFAEAIHSP
2161     QVAGVPRFRF GPPEDMPQTS SSHSDLGQLA SQGGLGMYET PLFLAHEEES GGRSVPTTPL QVAAPVTVFT ESTSADASEH
2241     ASQSVPMVTT STGNLSTTTE AGAGDDGDEV FAEAESEGIT SEAGLEIDSQ QEEESVQASD ESDLPSTSQD PPSSSSADTS
2321     SNQLKLFRRV RLQPPTLRTG VRGRQFNRQR GVTHAMGGRG GLNRGNIS
```

5.467 PREDICTED: similar to MGC84017 protein [Gallus gallus]

Protein Accession	gi|118096735
Mean Expression Ratio	1.00
Median Expression Ratio	1.01
Credible Interval	(0.777, 1.31)
Associated Peptides	1
Associated Spectra	1
Coverage	0.0273

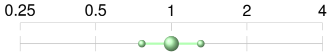

A	2.5	50	97.5	Sequence
1	0.76	1	1.3	YGVVVSEPDSGR

```
1        MEVGKYGKNA TKSGDRGVLL EPFIHQVGGH SSMMRYDDHT VCKPLITREQ RFYESLPPEM KEFTPEYKGV VSVCFEGDSD
81       GYINLVAYPY MENEALEQDD MPERDQPRRK HSRRSLHRSS SGTEHKEEKP GLASDSTESV QQEKEQEQRL GIRISLSHEK
161      EPLGTAGPLA LARDLLAEGG EPFFVLNSDV ICEFPFAALA RFHRQHGGEG SIVVTRVEEP AKYGVVVSEP DSGRICRFVE
241      KPRVFVSNKI NAGLYIFSPG ILQRIQLRPT SIEKEIFPAM AQDGQLYAME LQGFWMDIGQ PKDFLTGMCM YLQALRAQHP
321      EKLHSGPGVV GNVLVDPSAK IGANCVIGPN VTIGAGVVVE DGVRIKRCTV LQGARIRSHS WLESCIVGWS CSVGQWVRME
401      NVTVLGEDVI VNDELYLNGA NVLPHKSIAE SVPEPRIIM
```

5.468 PREDICTED: hypothetical protein, partial [Gallus gallus]

Protein Accession	gi|118123201
Mean Expression Ratio	1.01
Median Expression Ratio	1.01
Credible Interval	(0.781, 1.30)
Associated Peptides	1
Associated Spectra	1
Coverage	0.146

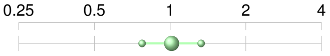

A	2.5	50	97.5	Sequence
**1**	**0.77**	**1.0**	**1.3**	**CPQIVIAFYEER**

1 SNDIARGFER GLEPEKIIGA TDSCGDLMFL MKWKDTDEAD LVLAKEANLK CPQIVIAFYE ERLTWHAYPE DTGSKEREAA
81 KS

5.469 PREDICTED: G elongation factor, mitochondrial 1 [Gallus gallus]

Protein Accession	gi|118095339
Mean Expression Ratio	1.01
Median Expression Ratio	1.01
Credible Interval	(0.78, 1.3)
Associated Peptides	1
Associated Spectra	1
Coverage	0.0149

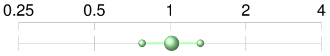

A	2.5	50	97.5	Sequence
**1**	**0.78**	**1.0**	**1.3**	**KQTGGAGQYGK**

1 MLRAVRAARG RGQRQFLLNA CRRCSSAVLP NERIRNIGIS AHIDSGKTTL TERVLFYTGR IAQMHEVRGK DGVGAVMDSM
81 ELERQRGITI QSAATYTMWK DTNINIIDTP GHVDFTIEVE RSLRVLDGAI LVLCAVGGVQ CQTITVNRQM KRYNVPFLTF
161 INKLDRMGSS PSRAVQQMRS KLKHNAAFVQ IPMGLEGNFK GIIDLIEERA IYFDGAFGQT VRYDEIPAEF RAEAAERRSE
241 LIECVANSDD RLGELFLEEK IPTVADIKLA IRRATLKKSF TPVLVGSALK NKGVQPLLDA VLEYLPNPSE VENYAILNQG
321 DSEDKAKFLL NSARDNSQPF VGLAFKLEAG RFGQLTYIRV YQGMLKKSDY IYNTRTGKRV RVQRLVRMHS DNMEDVNEVY
401 AGDICALFGI DCASGDTFTD KTSTDISMES IHVPDPVISV AMKPSNKNDF DKFSKGLSRF TREDPTFRVH FDDESKETIV
481 SGMGELHLEI YSQRMEREYS CPCTMGKPKV AFRENISAPV HFEYTHKKQT GGAGQYGKVI GVLEPLDPED YTKVEFEDRT
561 IGTNIPKQFV PAVEKGFRDA CEKGPVSGHK ISGVRFVLED GAHHMVDSNE FAFIRAGEGA LKQAMENAAV RLLEPIMAVE
641 VMAPTEFQGA VIAGINRRHG VITGQDGTEG YFTLYAEVPL NDMFGYASEL RSCTEGKGEY TMEYSKYHPC LPSTQEEIIN
721 KYLEATGRLP AKKGKAKS

5.470 PREDICTED: similar to Leucine rich repeat containing 33 [Gallus gallus]

Protein Accession	gi|50752132
Mean Expression Ratio	0.994
Median Expression Ratio	0.994
Credible Interval	(0.769, 1.28)
Associated Peptides	1
Associated Spectra	1
Coverage	0.0157

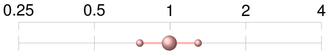

A	2.5	50	97.5	Sequence
**1**	**0.76**	**0.99**	**1.3**	**EALSGDLGWLR**

1 MRRPSPRRAA AEMEALLPVL SLLLVLLAVG WGDGAGAAWA MSPGGCEHVQ STADCKRKWL SSVPGNLQGD IEELLLDDNT
81 IRVLGRASLL SYHQLKHLSL TKNRMELLEP GAFLGSRGLH ALSLADNLLF TNYSLTAAAL SVLPALRVLD LSGNQLNEDM
161 ASVLLSNLSS LESLSMARNV IMRLDSSVFT NLTQLLELNL ERNYIFEIDQ AFEGLQRLQR LNIAYNYMTC IVDFNLTQLR
241 VLNVSYNIIE WFLALESDDL FELEVLDLSH NQLLFFPVLP RQSKLHSLLL KDNKMSFYQL LPNGTSLENV TVQFLLIDGN
321 STNITTVRLW DEVCYSNLSS LRLLDMSQNQ FWYLPKGFLA KMPALTHLKL NQNCLETFQL SERDPLAMLT DLDLSQNLLM
401 ELWEDVGAGP TLPSLQLFNL STNMLQALPA GIFTHTKQIT TVDLSYNRLD LCPQLAVLGR SAPCVDIRGL ATLTRLSLAG
481 CGLRDLGGHP FRGTALTHLD LSDNREALSG DLGWLRDVAV TLQVLSLRNT NLSSASVDFS AFQSLAGLDL SGNALTTLPA
561 SLGALKLRSL DLRDNSLTAL PADVAWRPLG RSLQELYLSR NPYNCCTLGW WEALQRGERL RVPDRHEVTC NYDSQRLSAL
641 VLPEPVLRDC RWRTADTALL YLVLALPTCL TLLVAFTVVF LTLKPKLLKM VKRRCGVSSP Y

5.471 PREDICTED: hypothetical protein [Gallus gallus]

Protein Accession	gi|118086240
Mean Expression Ratio	1.00
Median Expression Ratio	1.01
Credible Interval	(0.773, 1.30)
Associated Peptides	1
Associated Spectra	1
Coverage	0.00565

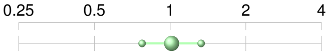

A	2.5	50	97.5	Sequence
**1**	**0.78**	**1.0**	**1.3**	**TLXTTPGAR**

```
1       MPEREKEPLK MDSYQMEEWI EVERPAAGQA YYRHIAFRQP REKYFPEPSR RAEGLIESYP NKKWRPIGRL CTNGPIYLAL
81      LDSDGEPMDV DPQLEELTEL EPPARGEAVA ESQAHCSQAV TTAEIPFWLK KQRRAEAETI LTTPPRHLVL TRTTPRERGG
161     GTDKGSRELP GGGKGKEDLA RAGRCARLRA CERGGQTGAR LAGRGFLSSP ASRMRCAPPG RVPGACVCVC QPRVALPAGG
241     HTAPGTWEPP RRVSGLGASA GPRATPDTPP RGGPARPDPA ARPGSGCGRR RKRRRQRRRR RPAGGLTPRS AARRRARPRR
321     PGMDEAASSS SKSRTLXTTP GARTGRDSRA AEAAAATAAG RAPSPRLVAA GKSGRVKRIA AGLKRPKSRR RLTRTRRRPT
401     ATTSQSPRPT GGSAHPPALA GTTAPRCIAP RRACGAANRX APWAAARPSA PTAAAAPPAT AGTVPTSAAE MCRPAPTAAG
481     GGHGAPTVPL SGDQQNLEAE VHTHQGTQDL VADTGCLDQE VVTQVFLLVH XLXRVAWLLS XTKTKKQEQL RQLKPVQKLP
561     TLQHQRRETL TLLQVHLNXT MXRTXRKXKL SIHLLLRAVQ IXRMIRQKQR SYFPKLKEKI IXSQKKILNR KLQQXKRIKX
641     LLXNSSQSLX KRKQNRVHQV XXQRRRSELL HYQPPHCHPC LCLPRCLKIM KLIAXKRILQ XSQLKKQRRN FAICLQTCHF
721     HLSCLEELSX AKVLKKRNQQ FSYTARGDQK YVDHDMVKQR KKKXTGENAV WINLISLVLL VKALMDKFTK PGIKTQGNWW
801     HXRKYDWIMK RKGFQLQLFE RLKFFDSLIT KALSTXRKXX QIRKMLWISR RTKVHFIWYL NIWTMTXWDC WNLVWFILMK
881     IIXNHLXDSX WRVWPIAIRR TFCTEIXNVQ IFYXIIGGRX SLQILGLLGC TTQKKADHIP TKLLHYGIGL LNFCLEKKDT
961     RQLSMSGAVA ASWVNFLQKS PFFKQIKNLL SWNLXVEFVG VLVQQCGLML XNXLISTRXN QRSSIVENXE KSLHSSHQLH
1041    XICLITCLLW IPASAVQLNK LFSVSFCEMW NHLRCLPQTF LCGRIAMSCG VKNAEDRSKW AXLMMQQQLK SLGRICLXAW
1121    MRAEPTPHKA CKLLPNSKLR ATLVXHLQKQ ALDSSXTRMK WQSCXTCYSL KQVLVWHSLP KCXILRXTQR LSSNXIKXIF
1201    LLEFCQQVKN SQNSSRRRLH HHRRNRSPXN SRPSHSLLYH LLSXVSLKWR LTLPRQLYRV HLLFCCLSXX RLSKQNKKIW
1281    CWRRRKTDQE MKCHYNSGSL QSPPLLHQVK RTCLGSPRXG SXTERRNRNA LGSCLQTSVP QSPQSPRLSL TKTLIIGQRT
1361    SICLXLTLLQ EQILTQEXKR HFCSCLLSIK LRQQQRSPYR RVWITRLETP MXQAQTTRTT LDHLPSPLLT MVVVMAXEVG
1441    HREHXNEGAS SETQIFSLWI TTVLLHLTRV VPLLRLPFQS PFPAQXLVME TFTSTLAPCY LVEIKIIGLN TATAQSQSWG
1521    TAGTLLWGRR VLIPCLLRCT TIAMEVTCRK TLVASATCMD RPGHLLHKDL ATPKDIGGTL VHLQXEGEAE DYHF
```

5.472 PREDICTED: hypothetical protein [Gallus gallus]

Protein Accession	gi|50740296
Mean Expression Ratio	0.996
Median Expression Ratio	0.995
Credible Interval	(0.774, 1.29)
Associated Peptides	1
Associated Spectra	1
Coverage	0.0321

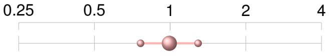

A	2.5	50	97.5	Sequence
**1**	**0.76**	**1**	**1.3**	**EFESHLDKLDNEKR**

```
1       MRSEAVPQPG GLERFASPGK GRGLRALRRY AVGELLFSCP AYTAVLTVSE RGSHCDGCFA RKEGLSKCGR CKQAFYCNVE
81      CQKEDWPMHK LECAAMCAFG QNWNPSETVR LTARILAKQK IHPERTQSEK LLAVKEFESH LDKLDNEKRE LIQNDIAALH
161     HFYSKHMEYP DNAALVVLFA QVNCNGFTIE DEELSHLGSA IFPDVALMNH SCCPNVIVTY KGTLAEVRAV KEIEPGEEVF
241     TSYIDLLYPT EDRNDRLRDS YFFTCDCREC TMKEKDKEKL KIRKLNDPPS AEAVRDMIKY ARNVIEEFRR AKHYKSPSEL
321     LEICELSLDK MGAVFEDSNV YMLHMMYQAM GVCLYVQDWE GALRYGQKII RPYSKHYPSY SLNVASMWLK LGRLYMALEN
401     RPAGDKALKK AIAIMEVAHG KDHPYISEIK KELEDH
```

5.473 PREDICTED: similar to blood island enriched kruppel like factor [Gallus gallus]

Protein Accession	gi|118094511
Mean Expression Ratio	0.993
Median Expression Ratio	0.995
Credible Interval	(0.764, 1.28)
Associated Peptides	1
Associated Spectra	1
Coverage	0.0251

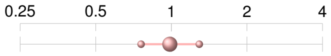

A	2.5	50	97.5	Sequence
1	0.76	0.99	1.3	RSLSGLASRR

```
1      MAAAVDESAL PSISTFTNLL PLEERPPDMM HRMLQHDGSA AAVKREEDEL GKFVDLDFIL AHTSSGGYPL PETPRAAAPR
81     TTFTAPTPRR ASSRRRTPTA RATATWPSFS PRTCPRTATC SGGSTRSCGC PAAPTTTRPC TRPCPARCPW TRRSLSGLAS
161    RRSSRRSAPA CWGYLAASTW DREVVNTSPP AWRRARCRGH RCRTLVSPTA AWAPPRSRCP VPTRTCWPTC RPTMPWPRIA
241    TRPTSPPSRP LSFMDTSVFS GSPXRGRGRA RRGCPGCWSR RPTPRCWSIF RPGAPPRTAS PNAAAARGRA SGRRRTTVNT
321    RAAARRTPRA PTSRRTCGRT RVRSPTTAPG KAAAGSSPVP MSXPATTAST RGTGPSSATS ASGPSPAPTT WPCTXSGTC
```

5.474 rabaptin, RAB GTPase binding effector protein 1 [Gallus gallus]

Protein Accession	gi|45384236
Mean Expression Ratio	0.996
Median Expression Ratio	0.995
Credible Interval	(0.773, 1.28)
Associated Peptides	1
Associated Spectra	1
Coverage	0.0128

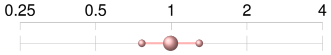

A	2.5	50	97.5	Sequence
1	0.76	1	1.3	KKDQEEDDQQR

```
1      MAQPGPSAPT DVALLQRVAE LEKVNAEFLR TKQQLEQEFN QKRAKFKELY LAKEEDLKRQ NAVLQAAQDD LGQLRTQLME
81     AHAEMENIKA IATVSENTKQ EAIDEVKRQW QEEVASLQAI MKETVRDYEL QYHHRMEQER AQWNQYRENV EREIAELRRR
161    LSEAQEEENL ENEMKKAQED AEKLRSVVMP MEKEIAALKE KLIEAEEKIK ELEASKVKEL NHYLEAEKSC RTDLEMYVAV
241    LNTQKSVLQE DAEKLRKELH EVCHLLEQER QQHNQLKHTW QKANDQFLES QRLLMRDMQR MEIVLTSEQL RQVEELKKKD
321    QEEDDQQRLS KRKEQKQKDS DDETKASCSL AHEESLTQFS NEEVHLNSTH SSVHSLDADL LLSSGESFNK SENDMFKDGL
401    RRAQSTDSLG TSGSLQSKAL GYNNKAKSAG NLDESDFGPL VGADSVSENY DTASLGSLQM PSGFMLTKDQ EKAIKAMTPE
481    QEETASLLSS VTQGVESAYV SPSGYRLVSE TEWNLLQKEV QNAGNKLGRR CDMCSNYEKQ LQGIQIQEAE TRDQVKKLQV
561    MLRQANDQLE KTMKDKQELE EYMKQSAEDS SNQISLLMVK CQKSENFLNE LQQAFSQAKR SVQEQMAVLT QSREQVSEEL
641    VRLQKDNESL QGKHSLHVSL QQAEDFILPE AAEELRELIL KYREDIISVR TAADHLEEKL KAEILFLKEQ IQAEQYLQEN
721    IEETLQLEIE NCKEEIASIS SLKAELERIK VEKEQLESSS QEYLQQLESL QETKNTLEEQ LKKETAAKAN LEQLVFEEKN
801    KAQRLQTELD VSEQVQRDFV KLSQTLQVQL ERIRQADSLE RIRAILNDTK LTDINQLPET
```

5.475 hypothetical protein FLJ10656 [Gallus gallus]

Protein Accession	gi|71896588
Mean Expression Ratio	1.01
Median Expression Ratio	1.01
Credible Interval	(0.777, 1.29)
Associated Peptides	1
Associated Spectra	1
Coverage	0.0261

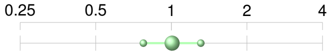

A	2.5	50	97.5	Sequence
1	0.77	1	1.3	EHKLEVR

```
1      MSAFSEAALE RKLSELSNSQ QSVQTLSLWL IHHRKHSALI VSVWERELRK AKPNRKLTFL YLANDVIQNS KRKGPEFTKD
81     FAPVIVEAFK HVSSESDESC KKHPGRVLSI WEERSVYEND VLEQLRQALY GDRKVRKRTY EQIKVDENNC SPRSSPTDPP
161    QTMDLIRALQ ELENAASGDA AVHQRIASLP IEVQDVSLLD RITDKESGEQ LSKMVDDACM LLADYNGRLA AEIDDRKQLT
241    RMLSDFLRCQ KEFLAEKEHK LEVRIVLF
```

5.476 Vinculin (Metavinculin)

Protein Accession	gi|50403716
Mean Expression Ratio	0.994
Median Expression Ratio	0.995
Credible Interval	(0.85, 1.16)
Associated Peptides	4
Associated Spectra	9
Coverage	0.0617

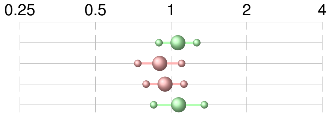

A	2.5	50	97.5	Sequence
3	0.9	1.1	1.3	DPNAPPGDAGEQAIR
2	0.74	0.9	1.1	GILSGTSDLLLTFDEAEVR
3	0.8	0.95	1.1	AIPDLTAPVSAVQAAVSNLVR
1	0.85	1.1	1.4	AAQMLQADPYSVPAR

```
1       MPVFHTRTIE SILEPVAQQI SHLVIMHEEG EVDGKAIPDL TAPVSAVQAA VSNLVRVGKE TVQTTEDQIL KRDMPPAFIK
81      VENACTKLVR AAQMLQADPY SVPARDYLID GSRGILSGTS DLLLTFDEAE VRKIIRVCKG ILEYLTVAEV VETMEDLVTY
161     TKNLGPGMTK MAKMIDERQQ ELTHQEHRVM LVNSMNTVKE LLPVLISAMK IFVTTKNTKS QGIEEALKNR NFTVEKMSAE
241     INEIIRVLQL TSWDEDAWAS KDTEAMKRAL ALIDSKMNQA KGWLRDPNAP PGDAGEQAIR QILDEAGKAG ELCAGKERRE
321     ILGTCKTLGQ MTDQLADLRA RGQGATPMAM QKAQQVSQGL DLLTAKVENA ARKLEAMTNS KQAIAKKIDA AQNWLADPNG
401     GSEGEEHIRG IMSEARKVAE LCEEPKERDD ILRSLGEISA LTAKLSDLRR HGKGDSPEAR ALAKQIATSL QNLQSKTNRA
481     VANTRPVKAA VHLEGKIEQA QRWIDNPTVD DRGVGQAAIR GLVAEGRRLA NVMMGPYRQD LLAKCDRVDQ LAAQLADLAA
561     RGEGESPQAR AIAAQLQDSL KDLKARMQEA MTQEVSDVFS DTTTPIKLLA VAATAPSDTP NREEVFEERA ANFENHAARL
641     GATAEKAAAV GTANKTTVEG IQATVKSARE LTPQVVSAAR ILLRNPGNQA AYEHFETMKN QWIDNVEKMT GLVDEAIDTK
721     SLLDASEEAI KKDLDKCKVA MANMQPQMLV AGATSIARRA NRILLVAKRE VENSEDPKFR EAVKAASDEL SKTISPMVMD
801     AKAVAGNISD PGLQKSFLDS GYRILGAVAK VREAFQPQEP DFPPPPPDLE HLHLTDELAP PKPPLPEGEV PPPRPPPPEE
881     KDEEFPEQKA GEAINQPMMM AARQLHDEAR KWSSKPVTVI NEAAEAGVDI DEEDDADVEF SLPSDIEDDY EPELLLMPTN
961     QPVNQPILAA AQSLHREATK WSSKGNDIIA AAKRMALLMA EMSRLVRGGS GNKRALIQCA KDIAKASDEV TRLAKEVAKQ
1041    CTDKRIRTNL LQVCERIPTI STQLKILSTV KATMLGRTNI SDEESEQATE MLVHNAQNLM QSVKETVREA EAASIKIRTD
1121    AGFTLRWVRK TPWYQ
```

5.477 PREDICTED: similar to Zinc finger SWIM domain-containing protein 5 [Gallus gallus]

Protein Accession	gi|118094551
Mean Expression Ratio	0.996
Median Expression Ratio	0.995
Credible Interval	(0.775, 1.29)
Associated Peptides	1
Associated Spectra	1
Coverage	0.0090

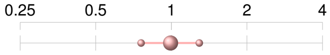

A	2.5	50	97.5	Sequence
1	0.76	1	1.3	LMSTDKAPLR

```
1       MAEGGEGEEL LRPPLPAGAP GPKRLCSRTC PAGGTGAAHP RPGAAGAGSG PGAGGSGAPA ASAGCCPAAG AAAGPAGAAA
81      SGAAAAAAAP QKPLFKCSLN EVPGAWRKVG FLCEEESQAA WAPRFHLSGT VTEPATATEP EMTYKVAISF DRCKITSVTC
161     GCGNKDIFYC AHVVALSLYR IRKPDQVKLR LPISETLFQM NRDQLQKFVQ YLITAHHTEV LPTAQKLADE ILSSNSEINQ
241     VHGAPDPTAG ASIDDENCWH LDEEQVREQV KLFLSQGGYY GSGKQLNSMF AKVREMLRMR DSNGARMLTL ITEQFMADPR
321     LSLWRQQGTG ITEKCRQLWD ELGALWVCVV LNPHCKLEEK SSWLRQLRKW GEMDVCPLED GNYGNELPNI TNALTQSSSH
401     SQDSLARPRR TVFTRAIEGC DLHWQDSHLQ RIISSDFYVS PAYQREGESL LFNSQGQPLW LEHVPTACAR VDALRSHGYP
481     REALRLTVAI INTLRLQQQR QLEIYKHQKK ELLQRGATTI TNLEGWVGHP LNPIGCLFLT LTEACRLEEE NCLEISDTGD
561     TKPPVYQHVP VTTGSQDSGE SYLSLALEVA LMGMGQQRVM PEGLYAQDKV CRNEEQIIAR LQDLELDPVL VQTLRKQCIL
641     LLEGGPFSGL GEVIHRESVP MHTFAKYLFS ALLPHDADLA YKLALRAMRL PVLETTAPSG DVTHPHHLVS VVPSRYPRWF
721     TLGHLESQQC ELASTMLTAA KGDMLRLRTV LEAIQKNIHS SSLIFKLAQD AFKIATPADS NSDTTLLNVA LELGLQVMRM
801     TLSTLNWRRR EMVRWLVTCA TEVGVRALVS ILQSWYSLFT PTEATSIVAA TVMSHNTILR LSLDYPQREE LASCARTLAL
881     QCAMKDPQNC ALSALTLCEK DHIAFETAYQ IVIDAASTGM TYTQLFTIAR YMEHRGYPLR AFKLASLAMT HLNLAYNQDT
961     HPAINDVLWA CALSHSLGKN ELAAIIPLVV KSVHCATVLS DILRRCTMTA PGLAGIPGRR NSGKLMSTDK APLRQLLDAT
1041    ISAYINTTHS RLTHISPRHY GEFIEFLSKA RETFLLAQDG HIQFAQFIDN LKQIYKGKKK LMLLVRERFG
```

5.478 PREDICTED: hypothetical protein [Gallus gallus]

Protein Accession	gi|118097162
Mean Expression Ratio	0.996
Median Expression Ratio	0.995
Credible Interval	(0.77, 1.29)
Associated Peptides	1
Associated Spectra	1
Coverage	0.0175

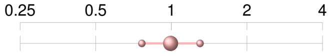

A	2.5	50	97.5	Sequence
1	0.76	1	1.3	YDLTVPFAR

```
1      MLRTLSVAAA VRCPLRLSRH CPLPGPLLLG LLSRQVREAA GSAGAECGSV LKTPKGTRDH HPAQMALRER LFGTVVACFK
81     RHGAAAIDTP VLELRETLME KYGDNSKLIY ELQDQGGELL ALRYDLTVPF ARYLAMNKIT NIKRYHIAKV YRRDNPAMTR
161    GRYREFYQCD FDIAGQFDPM IPDAECLKIV HEILSELQIG DFLIKVNDRR ILNGMFAICG IPESKLVTMC STLDKLDKMP
241    WEEVRNEMVG EKGLSPEAAD RIGEYVQLHG GMDLIEQLLQ DPKLSQNKLA KEGLGDMKLL FEYLTLFGIT GKISFDLSLA
321    RGLDYYTGVI IEAVLLQQEN DHGEESVSVG SVAGGGRYDG LVATFDPKGR KVPCVGISIG IERIFSILEQ RMKASGEKVR
401    TTETQVMVAT PQKHLLSARL KLISELWDAG IKAEMMYRKD PKLLKQLQYC EDMGIPLVAI IKKQELRDGV VKLRDAATRE
481    EVDIPREELA AEIRRRLETC TTGNPAAPQP LPSC
```

5.479 actin type 5, cytosolic - chicken

Protein Accession	gi|86169
Mean Expression Ratio	0.995
Median Expression Ratio	0.995
Credible Interval	(0.883, 1.12)
Associated Peptides	9
Associated Spectra	22
Coverage	NaN

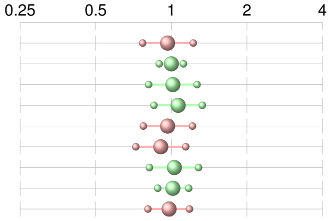

A	2.5	50	97.5	Sequence
1	0.77	0.97	1.2	DSYVGDEAQSK
9	0.9	1	1.1	SYELPDGQVITIGNER
1	0.81	1.0	1.3	DLYANTVLSGGTTMYPGIADR
1	0.85	1.1	1.3	LCYVALDFEQEMATAASSSSLEK
1	0.77	0.97	1.2	AGFAGDDAPR
1	0.72	0.9	1.1	GYSFTTTAER
1	0.82	1.0	1.3	VAPEEHPVLLTEAPLNPK
5	0.88	1.0	1.2	HQGVMVGMGQK
2	0.8	0.98	1.2	IWHHTFYNELR

5.480 PREDICTED: similar to cadherin-8 [Gallus gallus]

Protein Accession	gi|118096459
Mean Expression Ratio	0.996
Median Expression Ratio	0.995
Credible Interval	(0.77, 1.29)
Associated Peptides	1
Associated Spectra	1
Coverage	NaN

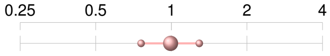

A	2.5	50	97.5	Sequence
1	0.76	1	1.3	TGDIHAMKRLDR

5.481 enolase 1 [Gallus gallus]

Protein Accession	gi|46048768
Mean Expression Ratio	0.996
Median Expression Ratio	0.996
Credible Interval	(0.889, 1.12)
Associated Peptides	9
Associated Spectra	18
Coverage	0.288

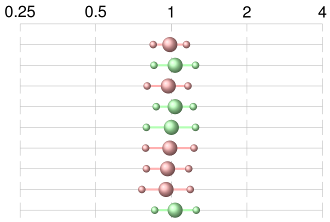

A	2.5	50	97.5	Sequence
4	0.85	0.99	1.2	DATNVGDEGGFAPNILENK
2	0.85	1.0	1.2	NYPVVSIEDPFDQDDWAAWK
2	0.8	0.97	1.2	VVIGMDVAASEFYR
3	0.87	1.0	1.2	AAVPSGASTGIYEALELR
1	0.8	1	1.2	FGANAILGVSLAVCK
1	0.79	0.99	1.2	GNPTVEVDLYTNK
2	0.8	0.97	1.2	IGAEVYHNLK
1	0.76	0.95	1.2	AVEHVNK
2	0.86	1.0	1.3	DGKYDLDFK

```
1       MSILKIHARE IFDSRGNPTV EVDLYTNKGL FRAAVPSGAS TGIYEALELR DNDKTRYLGK GVSKAVEHVN KTIAPALISK
81      NVNVVEQEKI DKLMLEMDGT ENKSKFGANA ILGVSLAVCK AGAAEKGVPL YRHIADLAGN PEVILPVPAF NVINGGSHAG
161     NKLAMQEFMI LPVGADTFKE AMRIGAEVYH NLKNVIKEKY GKDATNVGDE GGFAPNILEN KEALELLKTA IGKAGYSDKV
241     VIGMDVAASE FYRDGKYDLD FKSPDDPSRY ISPDQLADLY LGFVKNYPVV SIEDPFDQDD WAAWKKFTAS VGIQVVGDDL
321     TVTNPKRIAK AVEEKSCNCL LLKVNQIGSV TESLQACKLA QSNGWGVMVS HRSGETEDTF IADLVVGLCT GQIKTGAPCR
401     SERLAKYNQL LRIEEELGSK ARFAGRNFRN PRIN
```

5.482 UBP [Gallus gallus]

Protein Accession	gi|34541984
Mean Expression Ratio	1.00
Median Expression Ratio	1.00
Credible Interval	(0.775, 1.30)
Associated Peptides	1
Associated Spectra	1
Coverage	0.0192

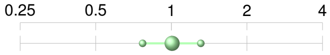

A	2.5	50	97.5	Sequence
1	0.77	1	1.3	LSESVLSPPCFVR

```
1       MNHHQQQQHQ KPGEQQLSEP EDMEMEAGDA DDPPRITQNP VINGNVAMAD GHNNTEEDME DDTSWRSEAT FQFTVERFNR
81      LSESVLSPPC FVRNLPWKIM VMPRLYPDRP HQKSVGFFLQ CNAESDSTSW SCHAQAVLKI INYKDDEKSF SRRISHLFFH
161     KENDWGFSNF MAWSEVTDPE KGFIEEDKVT FEVYVQADAP HGVAWDSKKH TGYVGLKNQG ATCYMNSLLQ TLFFTNQLRK
241     AVYMMPTEGD DSSKSVPLAL QRVFYELQHS DKPVGTKKLT KSFGWETLDS FMQHDVQELC RVLLDNVENK MKGTCVEGTI
321     PKLFRGKMVS YIQCKHVDYR SERIEDYYDI QLSIKGKKNI FESFIDYVAV EQLDGDNKYD AGEHGLQEAE KGVKFLTLPP
401     VLHLQLMRFM YDPQTDQNIK INDRFEFPEQ LPLDEFLQKT DPKDPANYIL HAVLVHSGDN HGGHYVVYLN PKGDGKWCKF
481     DDDVVSRCTK EEAIEHNYGG HDDDLSVRHC TNAYMLVYIR ESKLSEVLQP VTDHDIPQQL VERLQEEKRI EAQKRKERQE
561     AHLYMQVQIV AEDQFCGHQG NDMYDEEKVK YTVFKVLKNS TLTEFVQNLS QTMGFPQDQI RLWPMQARSN GKRNSSSSSS
641     ITWSTGIVAK DKMLIILIST PVGTIYSQLV FLESGHS
```

5.483 glutathione transferase (EC 2.5.1.18) mu2 - chicken

Protein Accession	gi|104677
Mean Expression Ratio	0.996
Median Expression Ratio	0.996
Credible Interval	(0.769, 1.29)
Associated Peptides	1
Associated Spectra	1
Coverage	NaN

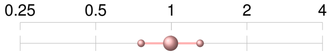

A	2.5	50	97.5	Sequence
1	0.76	1	1.3	LLLEYTETPYQER

5.484 Tubulin alpha-5 chain

Protein Accession	gi|135423
Mean Expression Ratio	1.00
Median Expression Ratio	1.00
Credible Interval	(0.773, 1.29)
Associated Peptides	1
Associated Spectra	1
Coverage	0.0335

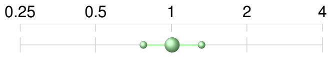

A	2.5	50	97.5	Sequence
**1**	**0.77**	**1**	**1.3**	**AIFVDLEPTVIDEVR**

```
1       MRECISVHVG QAGVQMGNTC WELYCLEHGI QPDGQMPSDK TIGGGDDSFT TFFCETGAGK HVPRAIFVDL EPTVIDEVRA
81      GIYRQLFHPE QLITGKEDGA NNYARGHYTI GKEIIDQVLD RIRKLADQCT GLQGFLCFHS FGGGTGSGFT SLLMERLSGD
161     YGKKSKLEFS IYPAPQVSTA VVEPYNSILT THTTLEHSDC AFMVDNEAIY DICRRNLDIE RPTYTNLNRL ISQIVSSITA
241     SLRFDGALNV DLTEFQTNLV PYPRIHFPLA TYAPVISAEK AYHEQLSVAE ITNSCFEPAN QMVKCDPRHG KYMACCLLYR
321     GDVVPKDVNA AIATIKTKRS IQFVDWCPTG FKVGINYQPP TVVAGGDLAK VQRIVCMLSN TTAIAEAWAR LDHKFDLMYA
401     KRAFVHWYVG EGMEEGEFSE AREDIAALEK DYEEVGLDSY EDEEEGEE
```

5.485 vimentin [Gallus gallus]

Protein Accession	gi|57240090
Mean Expression Ratio	1.00
Median Expression Ratio	1.00
Credible Interval	(0.803, 1.26)
Associated Peptides	1
Associated Spectra	4
Coverage	0.0504

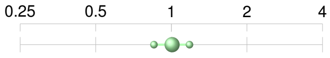

A	2.5	50	97.5	Sequence
**4**	**0.85**	**1**	**1.2**	**KVESLQEEIVFLK**

```
1       DVDNASLARL DLERKVESLQ EEIVFLKKLH DEEIRELQAQ LQEQHIQIDM DVSKPDLTAA LRDVRQQYES VAAKNLQEAE
81      EWYKSKFADL SEAANRNNDA LRQAKQEANE YRRQIQSLTC EVDALKGSNE SLERQMREME ENFAVEAANY QDTIGRLQDE
161     IQNMKEEMAR HLREYQDLLN VKMALDIEIA TYRKLLEGEE SRINMPIPTF ASLNLRETNI ESQPIVDTHS KRTLLIKTVE
241     TRDGQVINET SQHHDDLE
```

5.486 PREDICTED: similar to Adprhl1-prov protein [Gallus gallus]

Protein Accession	gi|50730512
Mean Expression Ratio	1.00
Median Expression Ratio	1.00
Credible Interval	(0.775, 1.3)
Associated Peptides	1
Associated Spectra	1
Coverage	0.0424

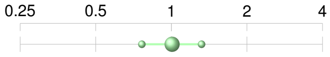

A	2.5	50	97.5	Sequence
**1**	**0.77**	**1**	**1.3**	**AALVLAGVGDALGYR**

```
1       MDKFKAALVL AGVGDALGYR NFSRQDNALG AKIQQELKEI GGLENLVLSP DKWPVSDNTL MHMATAEAVI TDYWCLEDLY
81      RELVKRYVDA VDKLSGRRPD PATIEGCREL KPDNYLLAWH TPFNEKGSGF GASTKAMCLG MRYWKPERLE SLIEVSIECG
161     RMTHNHPTGF LGSLCTALFV AYAIQGKPLV QWGREMMKVV PMAEEYCKKT IRHMAEYQEH WFYFEAKWQF YLEEREINEE
241     NQNKAVFPDN YDAEEREKTY RRWSSEGRGG RRGHDAPMIA YDALLGCGGD WTELCNRSMF HGGESAATGS IAGCLYGLVY
321     GLSKVPKGMY QDLEQRERLE YLGENLYRLS MEEK
```

5.487 PREDICTED: similar to Phosphoribosyl pyrophosphate synthetase-associated protein 1 [Gallus gallus]

Protein Accession	gi|118099635
Mean Expression Ratio	0.995
Median Expression Ratio	0.997
Credible Interval	(0.771, 1.28)
Associated Peptides	1
Associated Spectra	1
Coverage	0.0478

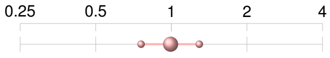

A	2.5	50	97.5	Sequence
**1**	**0.76**	**1**	**1.3**	**ASPFLIQYIQEEIPDYR**

```
1     MNAARSGYRV FSANSTAACT ELAKRITERL GAELGKSVVY QETNGETRVE IKESVRGQDI FIIQTIPRDV NTAVMELLIM
81    AYALKTSCAR NIIGVIPYFP YSKQSKMRKR GSIVCKLLAS MLAKAGLTHI ITMDLHQKEI QGFFSFPVDN LRASPFLIQY
161   IQEEIPDYRN AVIVAKSPDA AKRAQSYAER LRLGLAVIHG EAQCTEQDMD DGRHSPPMVK NATVHPGLEL PLMMAKEKPP
241   ITVVGDVGGR IAIIVDDIID DVESFVAAAE ILKERGAYKI FVMATHGLLS ADAPRLIEES SIDEVVVTNT VPHEVQKLQC
321   PKIKTVDISL ILSEAIRRIH NGESMAYLFR NITVDD
```

5.488 vacuolar protein sorting 35 [Gallus gallus]

Protein Accession	gi|57524880
Mean Expression Ratio	1.00
Median Expression Ratio	1.00
Credible Interval	(0.779, 1.30)
Associated Peptides	1
Associated Spectra	1
Coverage	0.0176

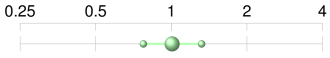

A	2.5	50	97.5	Sequence
**1**	**0.77**	**1**	**1.3**	**HFHPLFEYFDYESR**

```
1     MPTTQQSPQD EQEKLLDEAI QAVKVQSFQM KRCLDKNKLM DALKHASNKL GELRTSMLSP KSYYELYMAI SDELHYLEVY
81    LTDEFAKGRK VADLYELVQY AGNIIPRLYL LITVGVVYVK SFPQSRKDIL KDLVEMCRGV QHPLRGLFLR NYLLQCTRNI
161   LPDEGEQADE ETTGDISDSM DFVLLNFAEM NKLWVRMQHQ GHSRDREKRE RERQELRILV GTNLVRLSQL EGVNVERYKQ
241   IVLPGILEQV VNCRDALAQE YLMECIIQVF PDEFHLQTLN PFLRACAELH QNVNVKNIII ALIDRLALLA HREDGPGIPA
321   DIKLFDIFSQ QVATVIQSRQ DMPSEDVVSL QVSLINLAMK CYPDRVDYVD KVLETTVEIF NKLNLEHIAT SSAVSKELTR
401   LLKIPVDTYN NILTVLRLKH FHPLFEYFDY ESRKSMSCYV LSNVLDYNTE IVSQEQVDAI MNLVSTLIQD QPDQPAEDPD
481   PEDFADEQSL VGRFIHLLHS DDPDQQYKIL NTARKHFGAG GNQRIRFTLP PLVFAAYQLA FRYKENSKVD DKWEKKCQKI
561   FSFAHQTISA LIKAELAELP LRLFLQGALA AGEIGFENHE TVAYEFMSQA FSLYEDEISD SKAQLAAITL IIGTFERMKC
641   FSEENHEPLR TQCALAASKL LKKPDQCRAV STCAHLFWSG RNTDKNGEEL HGGKRVMECL KKALKIANQC MDPSLQVQLF
721   IEILNRYIYF YEKENEAVTI QVLNQLIQKI REDLPNLEST EETEQINKHF HNTLEHLRLR RESPESEGPI YEGLVL
```

5.489 PREDICTED: similar to phosphofructokinase isoform 1 [Gallus gallus]

Protein Accession	gi|118094885
Mean Expression Ratio	0.997
Median Expression Ratio	0.997
Credible Interval	(0.772, 1.29)
Associated Peptides	1
Associated Spectra	1
Coverage	0.0141

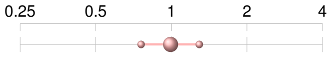

A	2.5	50	97.5	Sequence
**1**	**0.76**	**1**	**1.3**	**LLAHQKPAQEK**

```
1       MAAAELERLR MAGAGMAIAV LTSGGDAQGM NAAVRAVTRM GIYVGAKVFL IYEGYEGLVE GGDNIKQATW LSVSNIIQLG
81      GTVIGSARCK AFTTRQGRLR AARNLVEHGI TNLCVIGGDG SLTGADVFRA EWAGLLDELL RDGLISEEVA KANGRLNVVG
161     LVGSIDNDFC GTDMTIGTDS ALHRIMEVID AITTTAQSHQ RTFVLEVMGR HCGYLALVSG LASGADWLFI PESPPEDGWE
241     DLMCERLGET RSRGSRLNII IIAEGAIDRN GKPISSNYVK DLVVQRLGFD TRVTVLGHVQ RGGTPSAFDR VLSSKMGMEA
321     VMALLEATPD TPACVVSLSG NQSVRLPLME CVQVTKDVQK AMDEKRFDEA IQLRGRSFEN NWNIYKLLAH QKPAQEKSPF
401     SLAILNVGAP AAGMNAAVRS AVRISICRGH TIYAVSDGFE GLAKGQIREV GWHDVAGWLG RGGSMLGTKR TLPKTCMEKI
481     VENVRKFNIQ GLLVIGGFEA YEGVLQLVEA RGQYEELCII MCVIPATISN NVPGTDFSLG SDTAVNAAME SCDRIKQSAS
561     GTKRRVFIVE TMGGYCGYLS TVTGIAVGAD AAYVYEDPFT IHDLKANVEH LTDKMKTDIQ RGLVLRNEKC HEHYTTEFLY
641     NLYSSEGKGI FDCRINVLGH LQQGGAPTPF DRNYGTKLGV KAVLWMSEKL QEAYRKGRVF ANSADSACVI GLRKKVVAFS
721     PVTELKKVTD FEHRLPQEQW WLNLRLMLKM LANYQISLTE YISGKMEHVT RRTLSIEKGF
```

5.490 GDP dissociation inhibitor 2 [Gallus gallus]

Protein Accession	gi|45384364
Mean Expression Ratio	0.998
Median Expression Ratio	0.997
Credible Interval	(0.859, 1.16)
Associated Peptides	6
Associated Spectra	7
Coverage	0.194

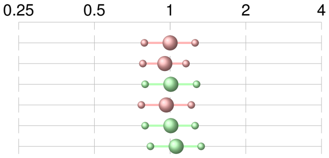

A	2.5	50	97.5	Sequence
1	0.79	1	1.3	NDIYGEEEQQ
2	0.78	0.95	1.2	TDDYLDQPCQETINR
1	0.8	1	1.3	FVSISDLFAPTDLGTESQIFISR
1	0.77	0.97	1.2	SPYLYPLYGLGELPQGFAR
1	0.8	1	1.3	FDLGQDVIDFTGHALALYR
1	0.84	1.1	1.3	KFDLGQDVIDFTGHALALYR

```
1       MNEEYDVIVL GTGLTECILS GIMSVNGKKV LHMDRNSYYG GESASITPLE DLYKRFNLPG TPPESMGRGR DWNVDLIPPF
81      LMANGQLVKM LLYTEVTRYL DFKVIEGSFV YKGGKIYKVP STEAEALASS LMGLFEKRRF RKFLVYVANF DENDPRTFEG
161     VDPKKTTMRD VYKKFDLGQD VIDFTGHALA LYRTDDYLDQ PCQETINRIK LYSESLARYG KSPYLYPLYG LGELPQGFAR
241     LSAIYGGTYM LNKPIEEIVI ENGKVVGVKS EGEVARCKQL ICDPSYVSDR VTKVGQVIRV ICILSHPIKN TNDANSCQII
321     IPQNQVNRKS DIYVCMISSA HNVAAQGKYI AIASTTVETA DPEKEIKPAL DLLEPIEQKF VSISDLFAPT DLGTESQIFI
401     SRTYDATTHF ETTCDDIKDI YKRMMGSEFD FEEMKRKKND IYGEEEQQ
```

5.491 troponin T type 2, cardiac [Gallus gallus]

Protein Accession	gi|45382073
Mean Expression Ratio	1.00
Median Expression Ratio	1.00
Credible Interval	(0.854, 1.18)
Associated Peptides	5
Associated Spectra	6
Coverage	0.205

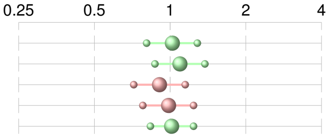

A	2.5	50	97.5	Sequence
1	0.81	1.0	1.3	DLNELQALIEAHFESR
1	0.87	1.1	1.4	DLEAEKFDLQEK
1	0.72	0.91	1.1	LDFDDIHRK
1	0.78	0.99	1.2	KKEEEELISLK
2	0.84	1.0	1.2	KPLNIDHLSEDKLR

```
1       MSDSEEVVEE YEQEQEEEYV EEEEEEWLEE DDGQEDQVDE EEEETEETTA EEQEDETKAP GEGGEGDREQ EPGEGESKPK
81      PKPFMPNLVP PKIPDGERLD FDDIHRKRME KDLNELQALI EAHFESRKKE EEELISLKDR IEQRRAERAE QQRIRSEREK
161     ERQARMAEER ARKEEEEARK KAEKEARKKK AFSNMLHFGG YMQKSEKKGG KKQTEREKKK KILSERRKPL NIDHLSEDKL
241     RDKAKELWQT IRDLEAEKFD LQEKFKRQKY EINVLRNRVS DHQKVKGSKA ARGKTMVGGR WK
```

5.492 PREDICTED: similar to pleckstrin homology domain containing, family H (with MyTH4 domain) member 2

Protein Accession	gi|118087949
Mean Expression Ratio	1.00
Median Expression Ratio	1.00
Credible Interval	(0.802, 1.26)
Associated Peptides	1
Associated Spectra	3
Coverage	0.006

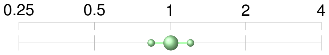

A	2.5	50	97.5	Sequence
3	0.84	1	1.2	QTIQLTTEK

```
1       MADLSEPEGT VNWKERCLTL ESQLMKFRLQ ASKIRELLAE KMQQLERQVI DADRQAERAF QQVQVMEEKL KAANVQTSES
81      ENRLYKRCQD LETLIQDKND IIQKLEQQLE EQKQIRLQEA KIIEEKAAKI KEWVTVKLHE LEVENQNLRL INQKQIKEMR
161     TVQSKLQEAT GKKSVTSTQK PGECQRLSGL TFGCFSNRAR SPQPSPKFEE ISKSSSKELD YAESKNTLEK DILETTSAGS
241     AHESNKGQKS LQQSSSGSEQ NKNVRTSRSS KDVDSDMSKN SCTTGSDWSS DEDGGDNKGL KSRCASTLSS HTSEENARYS
321     RVGSEMYLTA SDDSSSLFEE ESLGVQRTQH KKLYSWQQGS QRKGQNNTGG RCNSDFTNMK KDLDSSSDEL NKKFQSQRLD
401     YSSSSSEANT PSPILTPALA HKHAIRATSA DSQWTTPSDS PSNLPPPKLR TPNVFSINSS LAKKYLSQPQ LSSDRMFGRN
481     RNAISMIRPW RPQETDIDQV DGEDTAILEK MEIGCDEGVF TYDCTEAQDA EAQEPCDMTK KVASNKPPTP PLHRFPSWES
561     RIYAVAKSGL RMSEAFTTES LNKSAASLTS YSISGLYTSL IYKNMTTPVY TTLKGKATQI SNSPFIDESS GSEEEESSRS
641     SSRTSESDSR CRSGPGSPRA MKRGVSLSSV TSDCDYAIPP DAYSVDTDYS EPEQKLPKTS SSSSDNGKNE PLEKSGYLLK
721     MSGKVKTWKR RWFVLKGGEL LYYKSPSDVI RKPQGQIELN ASSHIERGDG KQTIQLTTEK RTYYLTADSP NILEEWIKVL
801     QNVLKIQAAS PLFIQPEIKP TMKGLLTKVK HGYSKRVWCT LVGKTLYYFR NQEDKFPLGQ IKLFEAKVEE VDRSCDSDED
881     YEASGRSLLS THYTVVIHPK DQGPTYLLIG SKHEKDTWLY HLTVAAGSTS VNVGSEFEQL ICKLLNVEGD TTSQIWRHPT
961     LCHSKEGITC PLTTLPSEAL QTEAIKLFKT CQLFINAAVD SPAIDYHVSL AQSALQICLT HPELQNEICC QLIKQTRRRH
1041    PQNQTGPIQG LQLLALCIGL FLPQHPFLWL LKLHLKKNAD SRTEFGKYAI YCQRCVERTQ QNGDREARPS RMEILSTLLR
1121    NPYHHSLPFS IPVHFMNGIY QVVGFDASTT VDEFLNTLNQ DTGMRKPAQS GFALFSDDPS GKDIEHCLQG NIKICDIISK
1201    WEQASKEQHP GKCEGTRTVR LTYKNRLYFS VQVHGETDRE KLLLVYQTND QIVNGLFPVN KELAMELSAL LAQVEIGDFE
1281    RPFSTPAGQV TSQSKSNQTL KQVLERFYPK RYRLGCSEEQ LRQLCQRLST RWMALRGHSA ADCVRIYLTV ARKWSFFGAK
1361    LFAAKPLATS SLEKSFIWFA VHEDGISILD YSTMRLTVTY TYKSLMTFGG YQDDFMLVVN DAQTKDKATE KLLFAMTKPK
1441    ILEITLLIAS YINNFHQQKG AAHHLSAPAL LTPQSGQKLK EMGSQPLLTN NRPTKCPTLL
```

5.493 fibronectin [Gallus gallus]

Protein Accession	gi|295716
Mean Expression Ratio	1
Median Expression Ratio	0.997
Credible Interval	(0.773, 1.30)
Associated Peptides	1
Associated Spectra	1
Coverage	0.066

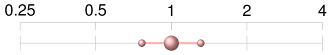

A	2.5	50	97.5	Sequence
1	0.76	1	1.3	EESVPLVGQQTTVSDVPR

```
1       LDSPTGLDFS DITANSFTVH WIAPRATITG YKIRHHPEHG VGRPKEDRVP PSRNSITLTN LLPGTEYVVS IIAVNGREES
81      VPLVGQQTTV SDVPRDLEVN PTSPTSLEIS WDAPAVTVRY YRITYGETGG SSPVQEFTVP GTMSRATITG LKPGVDYTIT
161     VYAVTGRGDS PASSKPVTVT YKTEIDTPSP MQVTDVQDNS ISIRWLPSSS PVTGYRVTAV PKKGHGPTKT KNVPPDQTQV
241     TIQGLEPTVE YMVSVYAQNQ NGESLPLVET AVT
```

5.494 PREDICTED: similar to serine palmitoyltransferase, long chain base subunit 1 [Gallus gallus]

Protein Accession	gi|118104215
Mean Expression Ratio	0.998
Median Expression Ratio	0.998
Credible Interval	(0.771, 1.29)
Associated Peptides	1
Associated Spectra	1
Coverage	0.0208

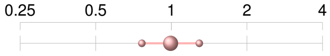

A	2.5	50	97.5	Sequence
1	0.76	1	1.3	IHRALQGISGLR

```
1       MVQAFYEVRR VCRGGMLGGG EPRPGVATGS EQTAAGPRPS RSDCGLSRRL PAALAVAVGI SWHRQRKAGF RLSGSQEESG
81      RELERCASPA GPPAPCALVY LQFLHSILNV WLLLKLLNSV YQAPAYHLIL EGILILWIIR LIFSKTYKLQ ERSDLTPKEK
161     EELIEEWQPE PLVPPVSKDH PALNYNIVSG PPTHKIIVNG KECVNFASFN FLGLLDNEKV KSAAQASLKK YGVGTCGPRG
241     FYGTFDVHLE LEDRLAKFMR TEEAIIYSYG FATIASAIPA YSKRGDIVFV DEAACFAIQK GLQASRSNIK LFKHNDMTDL
321     ERLLKEQETE DQKNPRKARV TRRFIVVEGL YMNTGDICPL PELIKLKYKY KVRIFLEESL SFGVLGEHGR GITEHFGINI
401     DDIDLISANM ENSLASIGGF CCGRSFIIDH QRLSGQGYCF SASLPPLLAA AAIEALNIME DNPEIFQTLR AKCERIHRAL
481     QGISGLRVVG ESFSPALHLR LEDSYGSREN DVKLLKRIVD YCINSGIALT QARYLEKEEK CLPSPSIRVV VTVEQTEQEL
561     DKAASLIKEA AKSVLN
```

5.495 capping protein (actin filament) muscle Z-line, beta [Gallus gallus]

Protein Accession	gi|45382141
Mean Expression Ratio	0.996
Median Expression Ratio	0.998
Credible Interval	(0.794, 1.24)
Associated Peptides	2
Associated Spectra	2
Coverage	0.101

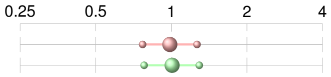

A	2.5	50	97.5	Sequence
1	0.77	0.98	1.3	YDPPLEDGAMPSAR
1	0.78	1	1.3	KLEVEANNAFDQYR

```
1     MSDQQLDCAL DLMRRLPPQQ IEKNLSDLID LVPSLCEDLL SSVDQPLKIA RDKVVGKDYL LCDYNRDGDS YRSPWSNKYD
81    PPLEDGAMPS ARLRKLEVEA NNAFDQYRDL YFEGGVSSVY LWDLDHGFAG VILIKKAGDG SKKIKGCWDS IHVVEVQEKS
161   SGRTAHYKLT STVMLWLQTN KTGSGTMNLG GSLTRQMEKD ETVSDSSPHI ANIGRLVEDM ENKIRSTLNE IYFGKTKDIV
241   NGLRSIDAIP DNQKYKQLQR ELSQVLTQRQ IYIQPDN
```

5.496 PREDICTED: similar to Adaptor-related protein complex 2, beta 1 subunit [Gallus gallus]

Protein Accession	gi|118100169
Mean Expression Ratio	0.998
Median Expression Ratio	0.999
Credible Interval	(0.77, 1.29)
Associated Peptides	1
Associated Spectra	1
Coverage	0.0116

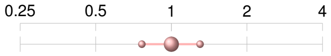

A	2.5	50	97.5	Sequence
1	0.76	1	1.3	GLEISGTFSHR

```
1     MTDSKYFTTN KKGEIFELKA ELNNEKKEKR KEAVKKVIAA MTVGKDVSSL FPDVVNCMQT DNLELKKLVY LYLMNYAKSQ
81    PDMAIMAVNS FVKDCEDPNP LIRALAVRTM GCIRVDKITE YLCEPLRKCL KDEDPYVRKT AAVCVAKLHD INAQMVEDQG
161   FLDSLRDLIA DSNPMVVANA VAALSEISES HPNSNLLDLN PQNINKLLTA LNECTEWGQI FILDCLSNYN PKDDREAQSI
241   CERVTPRLSH ANSAVVLSAV KVLMKFLELL PKDSDYYNML LKKLAPPLVT LLSGEPEVQY VALRNINLIV QKRPEILKQE
321   IKVFFVKYND PIYVKLEKLD IMIRLASQAN IAQVLAELKE YATEVDVDFV RKAVRAIGRC AIKVEQSAER CVSTLLDLIQ
401   TKVNYVVQEA IVVIRDIFRK YPNKYESIIA TLCENLDSLD EPDARAAMIW IVGEYAERID NADELLESFL EGFHDESTQV
481   QLTLLTAIVK LFLKKPSETQ ELVQQVLSLA TQDSDNPDLR DRGYIYWRLL STDPVTAKEV VLSEKPLISE ETDLIEPTLL
561   DELICHIGSL ASVYHKPPNA FVEGSHGIHR KHLPIHHGST DAGDSPVGTT ATTNLEQPQV IPSQGDLLGD LLNLDLGPPV
641   NVPQVSSMQM GAVDLLGGGL DSLLGSDLGG GIGGSPAVGQ TYIPSSVPAT FAPSPTPAVV SSGLNDLFEL SSGIGMAPGG
721   YVAPKSVWLP AVKAKGLEIS GTFSHRQGHI YMEMNFTNKA LQHMTDFAIQ FNKNSFGVIP STPLAIHTPL MPNQSIDVSL
801   PLNTLGPVMK MEPLNNLQVA VKNNIDVFYF SCLIPLNVLF VEDGKMERQV FLATWKDIPN ENELQFQIKD CHLNADTVSS
881   KLQNNNVYTI AKRNVEGQDM LYQSLKLTNG IWILAELRIQ PGNPNYTLSL KCRAPEVSQY IYQAYDAILK N
```

5.497 PREDICTED: similar to Human Diff6,H5,CDC10 homologue [Gallus gallus]

Protein Accession	gi|50752104
Mean Expression Ratio	0.999
Median Expression Ratio	0.999
Credible Interval	(0.772, 1.29)
Associated Peptides	1
Associated Spectra	1
Coverage	0.0442

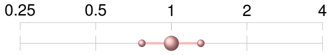

A	2.5	50	97.5	Sequence
1	0.76	1	1.3	STLINSLFLTDLYPER

```
1     MSKQQPAQFT NPETPGYVGF ANLPNQVHRK SVKKGFEFTL MVVGESGLGK STLINSLFLT DLYPERIIPG AAEKIERTVQ
81    IEASTVEIEE RGVKLRLTVV DTPGYGDAIN CRDCFKTIIS YIDEQFERYL HDESGLNRRH IIDNRVHCCF YFISPFGHGL
161   KPLDVEFMKA IHNKVNIVPV IAKADTLSLK ERERLKKRIL DEIEEHGIKI YHLPDAESDE DEDFKEQTRL LKASIPFCVV
241   GSNQLIEAKG KKVRGRLYPW GVVEVENPEH NDFLKLRTML ITHMQDLQEV TQDLHYENFR SERLKRGGRK IEDEEVNKDQ
321   ILLEKEAELR RMQEMIARMQ AQMQMQMQGG EGESSAVQGH NV
```

5.498 PREDICTED: similar to KIAA1734 protein [Gallus gallus]

Protein Accession	gi|118099629
Mean Expression Ratio	0.998
Median Expression Ratio	0.999
Credible Interval	(0.771, 1.29)
Associated Peptides	1
Associated Spectra	1
Coverage	0.0194

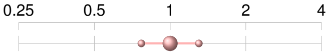

A	2.5	50	97.5	Sequence
**1**	**0.77**	**1**	**1.3**	**GAGGAASSGGSESGGPGAGGTEEGR**

```
1       MAELPAEAAP APAAPTPAPA LPLPPSAPPG GSQRLLFSHD LVSGRYRGSV RFGLVRLIHG EDSDSEEEET AAAPGGGGRG
81      AGGAASSGGS ESGGPGAGGT EEGRASPLRR GYVRVQWYPE GIKQHVKETK LKLEDRSVVP RDVVRHMSSS DSQCGTVIDV
161     NIECAVKLVG TNCILYPVNS KDLQHIWPFM YGDYIAYDCW LGKVYDLKNQ VILKLSNGAR CSMSTEDGAK LYDVCPHVSD
241     SGLFFDDSYG FYPGQVLIGP SKVFSSVQWL SGVKPVLSTK SKFRVVVEEV QVVELKVTWI TKSFCPGGTD SVSPPPSIIS
321     QENLSRVKRL GCFDHAQRQL GERCLYVFPD KVEPAKITCE CPERNCVLGE GSVAKKVRRL LKKQIVKIMS CSPESQGTTE
401     APDKEPAPAA DQKAEDLKPG SKCELEDSSN GAPSAGERKE EQPEETGKDK GGAAVRQQQP PFLLKDEGSA DYRLQSMEQD
481     ADDEAADDTD DTSSVTSSAS STASSQSGSG TGRKKSIPLS IKNLKRKHKK KKTKISREFK PGDRVAVEVV TTMTSADVMW
561     QDGTVETNIR SNELIPVHHL DNNEFCPGDF VVDKRAQSSQ DPGVYGVVQS GDHIGRTCVV KWFKLKSSGD DVELIGEEED
641     VSVYDIADHP DFRFRTTDIV IRIGNSDGAT ANEDEPSVGQ VARVDVSSKV EVVWADNSKT IILPQHLYNI ESEIEESDYD
721     SVDGSTSGAS SEWEDESDSW ETDNGLMEDD HPRIEELEPE EPAAEEVKKV EEQVQGAVAM AVADLAAEKA GKDGAPKSFR
801     ELKEAIKILE SLKNMTVEQL LTGSPTSPTV ELEKPTREKK FLDDIKKLQE NLKKTLDNVA IAEEEKMEAM VETEKKEEKA
881     EAQTPVRSEW PSETPVLCQQ SGGKPGVTFT SAKGEVFSVL ECAPDSHAFK KMEFQPPEAK KFFSTVRKEM ALLATSLPDG
961     IMVKTFEDRM DLFSALIKGP TRTPYEDGLF LFDIQLPNIY PAVPPLFRYL SQCSGRLNPN LYDNGKVCVS LLGTWIGKGT
1041    ERWTSKSSLL QVLISIQGLI LVNEPYYNEA GFDSDRGLQE GYENSRCYNE MTLIRVVQSM MQLLRRPVEV FEHEIREHFR
1121    CNGWRLVSRI ESWLETNELV ERSHEQSNGA DPLSGSAEQG AAAELSDSAL DGKEELDEAE FATSAPNAGD VRQHSDSEGA
1201    GQGRGVGLAR DRTDEGRAAQ DSASQPSVKP KKRRKSYRSF LPEKSGYPDI GFPLFPLSKG FIKSIRGVLQ QYRAALAGAN
1281    IPEWTEDK
```

5.499 signal recognition particle 14kDa (homologous Alu RNA binding protein) [Gallus gallus]

Protein Accession	gi|57529956
Mean Expression Ratio	0.999
Median Expression Ratio	1
Credible Interval	(0.774, 1.29)
Associated Peptides	1
Associated Spectra	1
Coverage	0.127

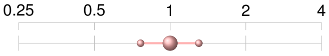

A	2.5	50	97.5	Sequence
**1**	**0.76**	**1**	**1.3**	**VLLESEQFLTELTR**

```
1       MVLLESEQFL TELTRLFQKC RTSGSVFITL KKYDGRTKPV PRKGHVESFE PADNKCLLRA TDGKKKISTV VSSKEVNKFQ
81      MAYSNLLRAN MDGLKKKDKK SKNKKSKATQ
```

5.500 PREDICTED: polymerase (DNA directed), epsilon [Gallus gallus]

Protein Accession	gi|118098794
Mean Expression Ratio	1.00
Median Expression Ratio	1
Credible Interval	(0.774, 1.29)
Associated Peptides	1
Associated Spectra	1
Coverage	0.00395

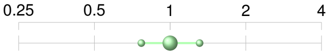

A	2.5	50	97.5	Sequence
**1**	**0.77**	**1**	**1.3**	**QLVRHLTGR**

```
1     MVLRGGGGGD RDPEGTRDDG PALSALKRLE RSQRTDQLDA LFGFERPAEP CERTGWLINM HPTEVLDEDR RAVSAVDYYF
81    IQEDGSRFKV ALPYRPYFYV ATQQGCEREV SSFLAKKFQG KIAKLETVPK EDLDLPNHLV GLKRHYLKLS FSTVDELVKV
161   RKEISPAVRK NRERDQANDV YTSMLSSALA GGSLTTSEEG ASKKVTNQMD NIVDMREYDV PYHMRLSIDL KIHVAHWYNV
241   RYRGSTFPPE ITRRDDLVER PDPVVLAFDI ETTKLPLKFP DPETDQIMMI SYMIDGQGYL ITNREIVSED IEDFEFTPKP
321   EYEGPFCVFN EPDEAHLIQR WFEHVQETKP TIIVTYNGDF FDWPFVEARA AAHGINMYQE IGFQKDSQGE YKASQCIHMD
401   CLRWVKRDSY LPVGSHNLKA AAKAKLGYDP VELDPEEMCR MATEEPQTLA TYSVSDAVAT YYMYMKYVHP FIFALCTIIP
481   MEPDEVLRKG SGTLCEALLM VQAYHANIIF PNKQEQEFNK LTEDGHVLDS ETYVGGHVEA LESGVFRSDI PCRFKMNPAA
561   FDYLVQNVEK TLRHAIEEEE RLPLDQVTNF QEVCDEIKVK LNSLKDVPNR IECPLIYHLD VGAMYPNIIL TNRLQPSAMV
641   DEATCAACDF NKPGANCQRR MTWQWRGEFM SASRSEYHRI QQQLESEKFP PLYPDGAPRA FHELSREEQA KYEKKRLADY
721   CRKAYKKIHV TKVEERVTTI CQRENSFYVD TVRAFRDRRY EFKGLHKVWK KKLSAAMETG DASEVKRCKN MEILYDSLQL
801   AHKCILNSFY GYVMRKGARW YSMEMAGIVC FTGANIITQA RELIEQIGRP LELDTDGIWC VLPNSFPENF VIKSTNAKKP
881   KVTISYPGAM LNILVKEGFT NDQYQELQDP ASLTYVTRSE NSIFFEVDGP YLAMILPASK EEGKKLKKRY AVFNEDGSLA
961   ELKGFEVKRR GELQLVKIFQ SSVFEAFLKG TTLEEVYASV AKVADYWLDV LYSKAANMPD SELFELISEN RSMSRKLEDY
1041  GEQKSTSIST AKRLAEFLGD QMVKDAGLSC RFIISKKPEG SPVTERAIPL AIFQAEPSVR KHYLRKWLKS PSLQDFNIRA
1121  ILDWDYYIER LGSTIQKIIT IPAALQQVKN PVPRVRHPDW LHKKLLEKND VYKQKKINEL FTSEGKRQPQ EGTPSSQVGD
1201  IEDFGAAKSL HPSVPIANKR KRVPTAEESQ QMSQSLELSQ SWREILGPPP SMGTTKEELV AWLRFHQKKW ELQARQRRER
1281  QKRRRLEDGG TAAGGGVVRD ALSRGLGSYL RHTARSILDL PWQIVQIAET SQPGLFRLWA VIGSDLHCIK LSIPRVFYVN
1361  QRVAKPEEGA VYRKVNRILP RSNLVYNLYE YSVPEDMYQE HINEINADLS APDIEGVYET QVPLLLRALI QLGCVCMVSR
1441  QLVRHLTGRE ADTFDIEHLE MRSLAQFPYL EPGEAAGSIR HIYLYHSSQG SRALFGLFIP AQRKAAVFVL DTVRSNQMPN
1521  LTTMFSAERS ALLERVGEEL LPPDKHTFEV RAETDPKAIC RAIQRLLLGY KDERRGPTLI AVQSNWELKR LASGIPILDE
1601  FPLVPIRVTD DISYSVLDWQ RHAARRMIRH YLNLDTCLSQ AFEMSRYYHI PIGNLPDDIS TFGSDLFFSR HLRRHNHLLW
1681  LSPTARPDLG GKEADDNRLV MEFDERASVE INNPGCYSTV CLELDIQSLA VNTVLQSHHV NDMEGCSSMS ISFDVIQQAS
1761  LEDMVTGNQA ANIPASYDET ALCSNTFRIL KSMVVSWVKE ITQYHNIYAD NQVIHFYRWL RSPSSLLYDP ALHRTLHNMM
1841  KKLFLQLVAE FRRLGSSVVY ANFNRIILCT KKRRIEDAIS YMEYIINSIH SKEIFHSLTI SFSRCWEFLL WMDPANYGGI
1921  KGKVHSRIHC GEDTSKKQAP EEGDSEEEEE AGEDDEEEGE SNVEELLENS WNIVQFLPQA ASCQNYFLMI VSAYIVAVYH
2001  SMKEELRRNA PGSTPVKRRG TSQVTQEAQG ETGAMPGMIT FSQDYVANEL TQSFFTITQK IQKKMSGSRH TTEPSDLFPV
2081  LPGSYLPLNN PALEFIKYVC QVLSLDANVT NQVNKLRRDL LRLIEVGEFS DEAQFRDPCR SYVLPEVICR NCNFCRDLDL
2161  CKDPALSQDG SLLPSWVCSN CQAQYDSDSI EMALVEALQK KLMAFMLQDL VCKKCHGVKD THMPLYCSCA GDFALTISSQ
2241  TFMEHITVFQ NIARHYGMAH LLETIEWLLH TNPQLQQ
```

5.501 hypoxia up-regulated 1 [Gallus gallus]

Protein Accession	gi|57528712
Mean Expression Ratio	1
Median Expression Ratio	1
Credible Interval	(0.801, 1.25)
Associated Peptides	2
Associated Spectra	2
Coverage	0.0180

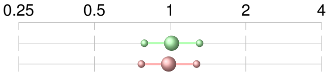

A	2.5	50	97.5	Sequence
1	0.79	1.0	1.3	EKKPEAGGESR
1	0.77	0.99	1.3	FAKPKPK

```
1     MARAPRWMLG WLLLACCVPH TEPLAVMSVD MGSESMKIAI VKPGVPMEIV LNKESRRKTP VAVALKENER LFGDSALGMS
81    IKTPKVAFRY FQDLLGKQID NPQVALYQSR FPEHELVKDE KRQTVIFKLS QTLQYSPEEM LGMVLNYSRG LAEEFAEQPI
161   KDAVITVPAY FNQAERRAVL HAARMADLKV LQLINDNTAV ALNYGVSGGK TSMPLHSFSF QNIMFYDMGA GSTVCTIVTY
241   QTVKTKDSGT QPQLQIQGIG FDRTLGGLEM ELRLRDYLAK LFNDQHPSKD VRKNPRAMAK LLKEANRLKT VLSANADHMA
321   QIEGLLDDID FKAKVSRQEF EDLCSDLFQR VPGPVQQALS SAEMNLDGID QVILVGGATR VPKVQEVLLK AVGKEELGKN
401   INADEAAAMG AVYQAAALSK AFKVKPFMVR DAAMFPIQVE FTREVEEDDK SKSLKHNKRI LFQRMAPYPQ RKVITFNRYT
481   DDFEFYVNYG DLSFLNQDDL RIFGSLNLTT VRLKGVGESF KKHSDYESKG IKAHFNMDES GVLSLDRVES VFETLVEDKL
561   EEESTLTKLG NTISSLFGGG GHTPEAGENL TDSVQEEEES LAEAAKEEQG VKQGQKSSAE DAGEEQGEEK QQSPHPDQAE
641   AVPPKEESQK NEEGEKSEAR DPKEDKETVN EEELSKSSGA GTAAKAEEEK KIKAPKKQKL VHEITMELDV NDVPDLLEDE
721   LKSSMKKLQD LTIRDLEKQE REKSANSLES FIFETQDKLY QEEYLFVSTE EEREEISKKL SEASNWMEEE GYAAATKELK
801   DKLAELKKLC RNLFFRVEER RKWPERLAAL ESLLNHSNIF LKGARMIPES DQIFTEVELG TLEKAINETT VWKNETLAEQ
881   NKLSPAEKPV LLSKDIELKI AGLDREVQYL LDKAKFAKPK PKKEKNATKS DSGKNATGTS ESENTIPPTE GKQEEKPEDI
961   SPAKEPPTTE KVVTDDEPGS DSSSKKEKKP EAGGESRKND EL
```

5.502 PREDICTED: hypothetical protein [Gallus gallus]

Protein Accession	gi|118086389
Mean Expression Ratio	1
Median Expression Ratio	1
Credible Interval	(0.774, 1.30)
Associated Peptides	1
Associated Spectra	1
Coverage	0.0208

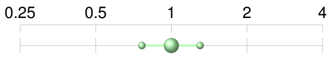

A	2.5	50	97.5	Sequence
**1**	**0.76**	**1**	**1.3**	**SHTPSPTSPK**

```
1       MAENHGLMER LEKAVTRLES LFSDSQRSGG MECDAMNGVN GSIAPYVEAF DRLLNGSVAE FLRYSKILEG DVKTHAEMVR
81      AAFQAQRSFL VLASQCQEPQ ENEVAVLLKP ISEKIQEIQN FRERNRGSKM FNHLSAVSES IPALGWIAVS PKPGPYVKEM
161     NDAATFYTNR VLKDYKHSDT RHVDWVKSYL NIWSELQAYI KEHHTTGLTW SKTGPVASPT SMRSVLTSGS CLSSPPPPPP
241     PPPGPPPIFD TETPPKDEGT ASRSALFAQL NQGEAITKGL RHVSDDQKTH KNPSLRAQGP PARSPTKSHT PSPTSPKNSP
321     QQSHAPVLEL EGKKWRVEYQ EDKNDLVITN TELKQVAYIF KCNKSTLQIK GKINSITIDN CKKFGLVFDN VVGIVEVINS
401     RDIQIQVMGK VPTISINKTE GCHIYLSEES LDCEIVSAKS SEMNILIPQD GDYKEFPVPE QFKTAWDGSK LVTEPAEIVG
```

5.503 hexokinase 2 [Gallus gallus]

Protein Accession	gi|45383696
Mean Expression Ratio	1
Median Expression Ratio	1
Credible Interval	(0.775, 1.29)
Associated Peptides	1
Associated Spectra	1
Coverage	0.0131

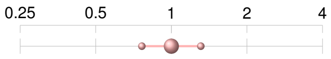

A	2.5	50	97.5	Sequence
**1**	**0.76**	**1**	**1.3**	**SANLCGATLAAR**

```
1       MIASHLLAYF FTELNHDQAQ KVDKYLYHMR LSEDTLQEVS ERFRKEMEKG LGADTNPTAS VKMLPSFVRS TPDGTEDGDF
81      LALDLGGTNF RVLRVKVSDN GLQKVEMESQ IYEIHEDLMR GSGMQLFDHI AECLGNFMEK LKIKDKKLPL GFTFSFPCHQ
161     TKLDESILVN WTKGFKCSSV EGKDVVSLLR RAIKKRGDFD IDIVAVVNDT VGTMMSCGYD DQNCEVGLIV GTGTNACYME
241     EMRHIDLVEG DEGRMCINME WGAFGDDGAL NDIRTEFDHE IDMGSLNPGK QLFEKMISGM YMGELVRLIL VKMAKEGLLF
321     QGKLSSDLRT TGHFETRFVS AIEKEKEGLQ KAHEILTKLG LEPSHEDCLA THRICQIVST RSANLCGATL AARLRRIKEY
401     KGVDPLRSTV GVDGSVYKKY PHFARRLHKT VRKLLPDCEI RFVRSEDGSG KGAAMVTAVA YRLAAQHKAR QKILEALKLS
481     HEQLLEVKRR MRVEMEKGLG KETHAEATVK MLPTYVCSTP DGTEKGDFLA LDLGGTNFRV LLVRVRNGMR RGVEMHNKIY
561     SIPLEVMQGT GEELFDHIVH CISDFLEYMG MKGVSLPLGF TFSFPCKQTN LDEGILLKWT KGFKATGCEG EDVVSLLKEA
641     IHRREEFDLD VVAVVNDTVG TMMTCGYEDP YCEVGLIVGT GSNACYMEEM RNVELVEGDE GRMCVNMEWG AFGDNGCLDD
721     IQTEFDLAVD ELSLNPGKQR FEKMISGMYL GEIVRNILMD FTKRGLLFRG RISERLKTRG IFETKFLSQI ESDCLALLQV
801     RSILQHLGLE STCDDSIIVK EVCTVVARRA AQLCGAGMAA VVDKIRENRG LDFLKVTVGV DGTLYKLHPH FSAIMQDTVR
881     QLSPCCEVTF LQSEDGSGKG AALITAVACR IREAGQ
```

5.504 PREDICTED: hypothetical protein, partial [Gallus gallus]

Protein Accession	gi|118118644
Mean Expression Ratio	1
Median Expression Ratio	1
Credible Interval	(0.773, 1.29)
Associated Peptides	1
Associated Spectra	1
Coverage	0.0342

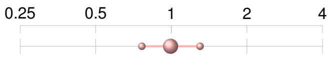

A	2.5	50	97.5	Sequence
**1**	**0.76**	**1**	**1.3**	**DEIDLAGLHR**

```
1       EHIQRRQEVH VVMGNEACDL DSTVSALALA YFLAKTSVPP KAAFIPVLNI PRTDFALRTE TTFLLREHSI PDSSLIFRDE
81      IDLAGLHRAG LLSLTLVDHH VLPSTDAALE EAVVDVLDHR PLEREWAPSC QLTVELVGSC ATLVTERIAQ GPPGVLDRTT
161     AALLHGTILL DSVNLSPAAG KVTPRDVCRA LEEATTPALH LQALPSPWPC ISAYAQGNTL ASRKKVLPIL RAALGGGPGT
241     AAGPEEEAAP PPTPMNSLVE ESPLAQAVPP VCPQAVLERV SRMAAEQPDS DK
```

5.505 myosin, heavy polypeptide 9, non-muscle [Gallus gallus]

Protein Accession	gi|45382693
Mean Expression Ratio	1
Median Expression Ratio	1
Credible Interval	(0.822, 1.22)
Associated Peptides	3
Associated Spectra	3
Coverage	0.0235

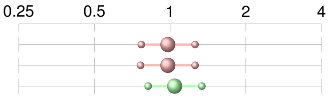

A	2.5	50	97.5	Sequence
**1**	**0.77**	**0.98**	**1.3**	**ELESQITELQEDLESER**
**1**	**0.76**	**0.98**	**1.3**	**NTDQASMPDNTAAQK**
**1**	**0.82**	**1.0**	**1.3**	**KQELEEICHDLEAR**

```
1     MAQRDADKYL YVDKNIINNP LTQADWAAKK LVWVPSEKSG FEAASLKEEV GDEAIVELAE NGKKVKVNKD DIQKMNPPKF
81    SKVEDMAELT CLNEASVLHN LKERYYSGLI YTYSGLFCVV INPYKNLPIY SEEIVEMYKG KKRHEMPPHI YAITDTAYRS
161   MMQDREDQSI LCTGESGAGK TENTKKVIQY LAHVASSHKS KKDQGELERQ LLQANPILEA FGNAKTVKND NSSRFGKFIR
241   INFDVNGYIV GANIETYLLE KSRAIRQAKE ERTFHIFYYL LSGAGEHLKT DLLLEPYNKY RFLSNGHVTI PGQQDKDMFQ
321   ETMEAMRIMG IPDEEQIGLL KVISGVLQLG NIVFKKERNT DQASMPDNTA AQKVSHLLGI NVTDFTRGIL TPRIKVGRDY
401   VQKAQTKEQA DFAIEALAKA TYEQMFRWLV MRINKALDKT KRQGASFIGI LDIAGFEIFE LNSFEQLCIN YTNEKLQQLF
481   NHTMFILEQE EYQNEGIEWN FIDFGLDLQP CIDLIEKPAG PPGILALLDE ECWFPKATDK SFVEKVVQEQ GTHPKFQKPK
561   QLKDKADFCI IHYAGKVDYK ADEWLMKNMD PLNDNIATLL HQSSDKFVSE LWKDVDRIVG LDQVAGMSET ALPGAFKTRK
641   GMFRTVGQLY KEQLAKLMAT LRNTNPNFVR CIIPNHEKKA GKLDPHLVLD QLRCNGVLEG IRICRQGFPN RVVFQEFRQR
721   YEILTPNAIP KGFMDGKQAC VLMIKALELD SNLYRIGQSK VFFRAGVLAH LEEERDLKIT DVIIGFQACC RGYLARKAFA
801   KRQQQLTAMK VLQRNCAAYL KLRNWQWWRL FTKVKPLLQV SRQEEEMMAK EEELIKVKEK QLAAENRLSE METFQAQLMA
881   EKMQLQEQLQ AEAELCAEAE EIRARLTAKK QELEEICHDL EARVEEEEER CQHLQAEKKK MQQNIQELEE QLEEEESARQ
961   KLQLEKVTTE AKLKKLEEDV IVLEDQNLKL AKEKKLLEDR MSEFTTNLTE EEEKSKSLAK LKNKHEAMIT DLEERLRREE
1041  KQRQELEKTR RKLEGDSSDL HDQIAELQAQ IAELKIQLSK KEEELQAALA RVEEEAAQKN MALKKIRELE SQITELQEDL
1121  ESERASRNKA EKQKRDLGEE LEALKTELED TLDSTAAQQE LRSKREQEVT VLKKTLEDEA KTHEAQIQEM RQKHSQAIEE
1201  LAEQLEQTKR VKANLEKAKQ ALESERAELS NEVKVLLQGK GDAEHKRKKV DAQLQELQVK FTEGERVKTE LAERVNKLQV
1281  ELDNVTGLLN QSDSKSIKLA KDFSALESQL QDTQELLQEE TRLKLSFSTK LKQTEDEKNA LKEQLEEEEE AKRNLEKQIS
1361  VLQQQAVEAR KKMDDGLGCL EIAEEAKKKL QKDLESLTQR YEEKIAAYDK LEKTKTRLQQ ELDDIAVDLD HQRQTVSNLE
1441  KKQKKFDQLL AEEKNISAKY AEERDRAEAE AREKETKALS LARALEEAIE QKAELERVNK QFRTEMEDLM SSKDDVGKSV
1521  HELEKAKRAL EQQVEEMKTQ LEELEDELQA TEDAKLRLEV NQQAMKAQFD RDLLGRDEQN EEKRKQLIRQ VREMEVELED
1601  ERKQRSIAVA ARKKLELDLK DLESHIDTAN KNRDEAIKHV RKLQAQMKDY MRELEDTRTS REEILAQAKE NEKKLKSMEA
1681  EMIQLQEELA AAERAKRQAQ QERDELADEI ANSSGKGALA MEEKRRLEAR IAQLEEELEE EQGNTEIIND RLKKANLQID
1761  QMNADLNAER SNAQKNENAR QQMERQNKEL KLKLQEMESA VKSKYKATIT ALEAKIVQLE EQLDMETKER QAASKQVRRA
1841  EKKLKDILLQ VDDERRNAEQ FKDQADKANM RLKQLKRQLE EAEEEAQRAN VRRKLQRELD DATETADAMN REVSSLKSKL
1921  RRGDLPFVVT RRLVRKGTGE CSDEEVDGKA EAGDAKATE
```

5.506 ubiquitin-conjugating enzyme E2L 3 isoform 1 [Homo sapiens]

Protein Accession	gi|4507789
Mean Expression Ratio	1
Median Expression Ratio	1
Credible Interval	(0.778, 1.29)
Associated Peptides	1
Associated Spectra	1
Coverage	0.0974

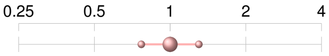

A	2.5	50	97.5	Sequence
**1**	**0.77**	**1**	**1.3**	**IEINFPAEYPFKPPK**

```
1     MAASRRLMKE LEEIRKCGMK NFRNIQVDEA NLLTWQGLIV PDNPPYDKGA FRIEINFPAE YPFKPPKITF KTKIYHPNID
81    EKGQVCLPVI SAENWKPATK TDQVIQSLIA LVNDPQPEHP LRADLAEEYS KDRKKFCKNA EEFTKKYGEK RPVD
```
