# Supplementary material for: iQuantitator: A tool for protein expression inference using iTRAQ
Source: BMC Bioinformatics. 2009 Oct 18;10:342. doi: 10.1186/1471-2105-10-342 (PMC2770557; doi:10.1186/1471-2105-10-342)
Supplement: Additional File 3 — iQuantitator Installation and Usage Manual. A step-by-step introduction to the installation and use of iQuantitator based upon the sample data and scripts provided here. [file 1471-2105-10-342-S3.PDF]

iQuantitator Overview

John Schwacke

September 18, 2009

1 Introduction

This document provides an introduction to the iQuantitator software using the sample dataset and scripts supplied with the package. iQuantitator facilitates the analysis of quantitative proteomic data from experiments using the Applied Biosystems iTRAQ reagents.

2 Installation

2.1 Installing R

iQuantitator is a package that operates within the R Statistical Computing Environment. A copy of R must, therefore, be installed on your computer. Download the most recent version of R from the Comprehensive R Archive Network (CRAN). The R Installation and Administration manual contains details on installing R for your operating system.

2.2 Installing iQuantitator

To install iQuantitator, use the R `INSTALL` command at the command line with the iQuantitator package file name as the command argument. R should respond with something similar to that shown below. This command will compile the iQuantitator C code on your computer and install the R code and associated documentation.

```
> R CMD INSTALL iquantitator_1.0.tar.gz
* Installing to library '<yourdirectory>/R/x86_64-pc-linux-gnu-library/2.6'
* Installing *source* package 'iquantitator' ...
** libs
gcc -std=gnu99 -I/usr/share/R/include -I/usr/share/R/include      -fpic  -g -O2 -c iTRAQSampler.c -o iT
gcc -std=gnu99 -I/usr/share/R/include -I/usr/share/R/include      -fpic  -g -O2 -c scanFASTA2.c -o scan
gcc -std=gnu99 -shared  -o iquantitator.so iTRAQSampler.o scanFASTA2.o   -L/usr/lib64/R/lib -lR
** R
** inst
** help
 >>> Building/Updating help pages for package 'iquantitator'
     Formats: text html latex example
  buildModelTable                   text    html    latex
```

```
  getFASTASequences                 text    html    latex
  iquantitator-package              text    html    latex
  loadMSMSSummary                   text    html    latex
  processiTRAQ                      text    html    latex
  sampleAOVModel                    text    html    latex
  seqcoverage                       text    html    latex
  summarizeRow                      text    html    latex
  summarizeSamples                  text    html    latex
  summarizeiTRAQ                    text    html    latex
  trimFactor                        text    html    latex
** building package indices ...
* DONE (iquantitator)
```

3 Elements of the R Script

The iQuantitator package does not provide a graphical user interface and was designed primarily for use by researchers and statisticians familiar with R programming. To make use of this software, a short R script is needed. Throughout this section we provide an annotated description of one such script, designed to analyze the sample dataset. The iQuantitator package must first be loaded into the R environment.

```
> library(iquantitator)
```

3.1 Study Name

The study name is defined by setting the variable `studyname` to a suitably descriptive string. This string will be placed in the title and the header of each page of the resulting report.

```
> studyname <- "Krug, HH36 vs HH39"
```

3.2 Experiment Description

The experiment layout is then described in an R data frame containing four columns; `experiment`, `treatment`, `channel`, and `sample`. Each row of this data frame corresponds to one channel from the collection of experiments being analyzed. A single 4-plex experiment, for example, would have 4 rows and two 8-plex experiments would have 16 rows. The `experiment` column identifies the iTRAQ run from which the data came. Here we have have an experiment with a single 4-plex iTRAQ run which has been given the label 'A'. The `treatment` column gives the treatment group associated with the channel specified in the `channel` column. In cases where paired tests are to be performed, the `sample` column can be used.

```
> edesign <- data.frame(experiment = c("A", "A", "A", "A"), treatment = c("HH39",
+     "HH36", "HH36", "HH39"), channel = c("114", "115", "116",
+     "117"), sample = c("S1", "S1", "S2", "S2"))
```

3.3 Input Data

Input data is read from a set of tab-delimited text files, one for each iTRAQ run. Each file must contain one line for each MS/MS spectrum collected in the run. These are typically generated by exporting the

spreadsheets produced by the vendor-supplied software (MSMS summary reports) as text files. An array of filenames, indexed by the identifier given in the `experiment` column of the experiment layout data frame, must be specified.

```
> mlist <- vector(mode = "character")
> mlist["A"] <- "krug3639MSMSSummary.txt"
```

3.4 Loading and Preprocessing Data

Loading data into R for analysis is accomplished using the `loadMSMSSummary` function. This function has many arguments that can be used to tailor the mapping of the data columns in the input files to the input data needed by iQuantitator. Use the help command or review the iQuantitator function documentation for more details. In this example, the default values are suitable for our file format and we only specify a filter to remove spectra from peptides containing iTRAQ-modified tyrosines. The resulting structure (`msmsload` in this example) has a number of lists and data frames that summarize the loaded data, list the proteins and peptides, provide an identifier to protein name cross-reference, give summary statistics, and provide a data frame of observations that is the basis for the following steps.

```
> modfilters <- c("Tyrosine\\(Y\\)_iTRAQ")
> msmsload <- loadMSMSSummary(edesign, mlist, modfilters = modfilters)

krug3639MSMSSummary.txt
Eliminated 0 low confidence spectra.
Eliminated 14488 unknown peptides.
Eliminated 0  spectra with missing data.
Eliminated 0  degenerate peptides.
Eliminated 42  peptides with disallowed modifications.
Eliminated 0  contaminating proteins.

> msmsload$observations[1:5, -6]

  LogIntensity Experiment Channel Treatment     Protein             Peptide
1     8.752767          A     114      HH39 gi|45382679        GSDVNEAQPTPAE
2     8.523557          A     114      HH39   gi|211372    EGEGEGEGEEDEEEEEE
3     9.441787          A     114      HH39 gi|71895319             AIENLMQK
4    10.293329          A     114      HH39   gi|465508 TAEPPAENTSAPEAEQGGAE
5    10.500407          A     114      HH39 gi|45382723    QEAPSESSPEGPAEPAE
  Spot Sample Spectrum
1  358     S1        1
2  363     S1        2
3  374     S1        3
4  378     S1        4
5  385     S1        5
```

We note that the data in `observations` is organized in a manner that would facilitate the application of other regression methods for the inference of treatment-dependent changes in expression.

3.5 Describing the Model

The statistical model is described in a restricted form of the R formula grammar. The model must be specified in terms of the columns found in the `observations` data frame. Additional columns can be added to this data frame as long as they are categorical. There is currently no support for continuous covariates in the following steps. In this example, log intensity is described by channel, spectrum, peptide, protein and peptide-treatment and protein-treatment interactions.

```
> model <- formula(LogIntensity ~ Channel + Spectrum + Protein +
+     Peptide + Protein:Treatment + Peptide:Treatment)
```

iQuantitator uses Monte Carlo Markov methods for inferring parameters of the given model. While the default prior specifications may be appropriate in some cases, users can modify these settings through the prior specification structure. In this example, an intercept term is included in the model and the protein, peptide and spectrum terms will be overparameterized (see next section). We therefore, constrain the mean value of the prior distributions for channel, spectrum, protein, peptide, and the interaction terms to 0.

```
> priors <- list(Channel = list(nomeanprior = 1, mu = 0), Spectrum = list(nomeanprior = 1,
+     mu = 0), Protein = list(nomeanprior = 1, mu = 0), Peptide = list(nomeanprior = 1,
+     mu = 0), Protein.Treatment = list(nomeanprior = 1, mu = 0),
+     Peptide.Treatment = list(nomeanprior = 1, mu = 0))
```

3.6 Executing the Sampler

The Gibbs sampler included in the iQuantitator package is executed by calling the `processiTRAQ` function. The loaded data, model, and prior specifications established previously are used here. Additionally, the user specifies the number of MCMC samples to be collected, the thinning factor, and the burn-in period. In this example, 10,000 samples are collected from 20×10000 draws from the sampler after a burn-in period of 2,000 samples.

```
> samples <- processiTRAQ(msmsload, model, nsamples = 10000, thin = 20,
+     skip = 2000, overparameterize = list(Protein = TRUE, Peptide = TRUE,
+         Spectrum = TRUE), priors = priors)
```

```
             Channel Spectrum Protein Peptide Protein:Treatment
LogIntensity       0        0       0       0                 0
Channel            1        0       0       0                 0
Spectrum           0        1       0       0                 0
Protein            0        0       1       0                 2
Peptide            0        0       0       1                 0
Treatment          0        0       0       0                 1
             Peptide:Treatment
LogIntensity                 0
Channel                      0
Spectrum                     0
Protein                      0
Peptide                      1
Treatment                    1
```

```
Staring sampler...
5.366927
```

Each row of the resulting structure contains the samples drawn for one of the model parameters. The row name gives the factor (or interaction) and associated level(s). In the plots below (Figures 1 and 2), we examine the sequence of 10,000 samples from a protein-treatment interaction that shows an increase in protein expression for treatment group HH39 relative to HH36.

3.7 Summarizing the Results

The results of the analysis are next summarized using the `summarizeiTRAQ` function. This function builds a hierarchical data structure from proteins down to peptides, computes summary statistics, and acquires the protein sequence from a FASTA file using the protein identifier. This structure contains the information needed to construct the final report using the supplied Sweave files. Users may develop their own scripts for analyzing the samples collected from the Gibbs sampler as an alternative.

```
> summary <- summarizeiTRAQ(msmsload, samples, "HH39", "krug3639.fasta")
```

```
> summary[["gi|118085057"]]
```

```
$stats
      Mean         SD       2.5%        50%      97.5%       npep     scount
0.23736486 0.10113119 0.03564845 0.23693953 0.43871358 3.00000000 3.00000000
  coverage
0.05938242

$pepstats
$pepstats$EIVSVTVSK
       Mean          SD        2.5%         50%       97.5%           A
 0.22555110  0.12567468 -0.01755487  0.22649170  0.47036427  1.00000000

$pepstats$GKPDTEVTEDEEYKR
      Mean         SD       2.5%        50%      97.5%          A
0.30451442 0.12540329 0.05783792 0.30481491 0.54899335 1.00000000

$pepstats$KGKPDTEVTEDEEYKR
     Mean        SD      2.5%       50%     97.5%         A
0.3469077 0.1254668 0.1021040 0.3474682 0.5935522 1.0000000


$sequence

"MAAVAMLGRRRAAAGLLRALKYASRGYASQRTLNEVVIASAARTPIGSFQGSLSSLPATKLGSIAIKGAIDRAGIPAEEVKEAYMGNVLQAGQGQAPARQAVI
```

3.8 Generating the Final Report

iQuantitator uses the Sweave function, a part of the R utils package, to create a LaTex document. The template for that document is given in the `summaryReport.Rnw` document included in the distribution.

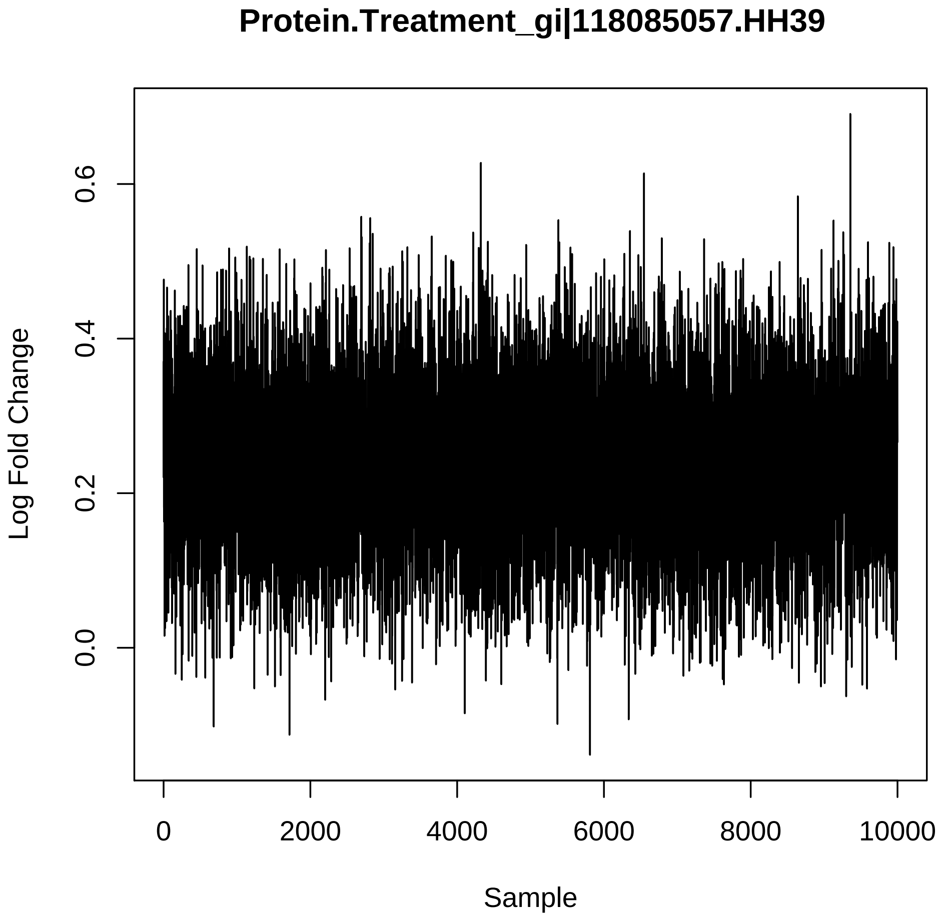

Figure 1: Example of sampler output. Created from the MCMC samples using `plot(samples[i,],type="l",ylab="Log Fold Change",xlab="Sample",main=rownames(samples)[i])`

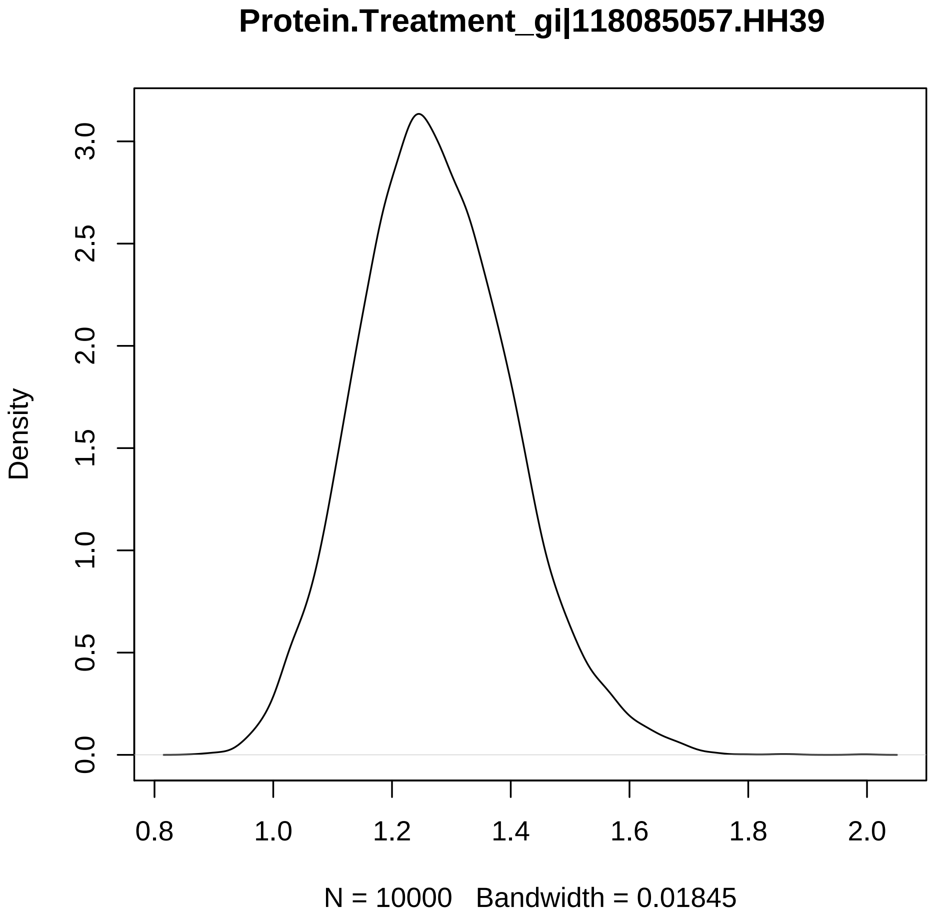

Figure 2: Density plot of sampler output showing fold change. Created from the MCMC samples using `plot(density(exp(samples[i,])),main=rownames(samples)[i])`

Sweave is quite general and can be used to create HTML as well as LaTex documents. The template can be customized or replaced as desired. The resulting file (`krug3639.tex` in this example) can then be converted to pdf using `pdflatex`.

```
> Sweave(system.file("summaryreport2.Rnw", package = "iquantitator"),
+     output = "krug3639.tex")
```

4 Summary

This document summarizes a simple application of the iQuantitator package. For additional information, see the documentation for each of the iQuantitator methods used here.
